# Catalytic, Regioselective 1,4‐Fluorodifunctionalization of Dienes

**DOI:** 10.1002/anie.202214906

**Published:** 2022-12-01

**Authors:** You‐Jie Yu, Michael Schäfer, Constantin G. Daniliuc, Ryan Gilmour

**Affiliations:** ^1^ Organisch-Chemisches Institut Westfälische Wilhelms-Universität Münster Corrensstraße 36 48149 Münster Germany

**Keywords:** Conformation, Difunctionalization, Fluorination, Hypervalent Iodine, Regioselectivity

## Abstract

A catalysis‐based regioselective 1,4‐fluorofunctionalization of trifluoromethyl substituted 1,3‐dienes has been developed to access compact, highly functionalized products. The process allows *E*,*Z*‐mixed dienes to be processed to a single *E*‐alkene isomer, and leverages an inexpensive and operationally convenient I(I)/I(III) catalysis platform. The first example of catalytic 1,4‐difluorination is disclosed and subsequently evolved to enable 1,4‐hetero‐difunctionalization, which allows δ‐fluoro‐alcohol and amine derivatives to be forged in a single operation. The protocol is compatible with a variety of nucleophiles including fluoride, nitriles, carboxylic acids, alcohols and even water thereby allowing highly functionalized products, with a stereocenter bearing both C(sp^3^)−F and C(sp^3^)−CF_3_ groups, to be generated rapidly. Scalability (up to 3 mmol), and facile post‐reaction modifications are demonstrated to underscore the utility of the method in expanding organofluorine chemical space.

## Graphical abstract

A catalysis‐based regioselective 1,4‐fluorofunctionalization of trifluoromethyl substituted 1,3‐dienes has been developed to access compact, highly functionalized products. The platform has been extended to enable 1,4‐hetero‐difunctionalization, which allows δ‐fluoro‐alcohol and amine derivatives to be forged in a single operation.

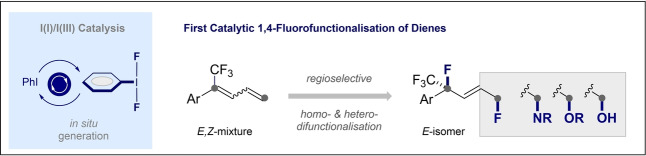

Advancing catalysis‐based platforms to enable the regioselective homo‐ and hetero‐difunctionalization of π‐systems is a powerful approach to improve *molecular literacy*,^[1]^ and expand chemical space.^[2]^ This reflects the accessibility of unsaturated substrates and the intrinsic atom economy that is associated with forging two new σ bonds in a single operation.^[3]^ The intervention of small molecule catalysts continues to enrich this arena by mitigating conventional reactivity limitations (e.g. functionalization sites) that would otherwise compromise efficiency.^[4]^ Sustained innovation in the site‐selective addition of nucleophile/electrophile combinations across simple alkenes is testimony to this success, and has logically stimulated interest in the activation of larger π‐systems, beginning with dienes.[^5^, ^6^]

Motivated by the ubiquity of fluorinated motifs in functional small molecule discovery,[^7^, ^8^] and cognizant that direct, I(I)/I(III) catalysis‐based [1,*n*]‐difluorination is restricted to *n*=3,^[9]^ it was envisaged that a direct 1,4‐difluorination of dienes would be highly enabling (Scheme 1A). This fundamental advance would not only augment the existing 1,1‐^[10]^ 1,2‐^[11]^ and 1,3‐difluorination[^12^, ^13^] series, but it would also facilitate access to novel discovery modules for contemporary medicinal chemistry.^[14]^ With this latter point in mind, and to demonstrate preliminary proof of concept, trifluoromethyl substituted 1,3‐dienes were conceived to be promising substrates. An appealing feature of the 1,4‐difluorination product is the stable tertiary fluoride in which the C(sp^3^)‐CF_3_ substituent confers a high degree of stability.^[15]^ It was envisaged that upon exposure to difluorination conditions, orchestrated by an I(I)/I(III) manifold,^[16]^ the ArIF_2_ species generated in situ^[17]^ would activate the less sterically hindered terminal alkene of the diene (**I**) (Scheme 1B). This would induce a stepwise mechanism in which a stabilizing impact of the allylic/benzylic substituents of the cation would off‐set the‐I_π_ effect of the CF_3_ group (Scheme 1B, **II**↔**III**). Concomitant generation of the thermodynamically favored *E*‐alkene, and displacement of the iodonium intermediate, would liberate the product (**IV**). However, (asynchronous) concerted character, in which intramolecular fluorination leads to installation of the benzylic C(sp^3^)‐F center, cannot be discounted. This mechanistic spectrum affords the latitude to rationalize 1,4‐difunctionalization and expand the process to regioselective intermolecular hetero‐difunctionalization with *O*‐ and *N*‐based nucleophiles to access δ‐fluoroalcohol and amine derivatives, respectively.

**Scheme 1:**
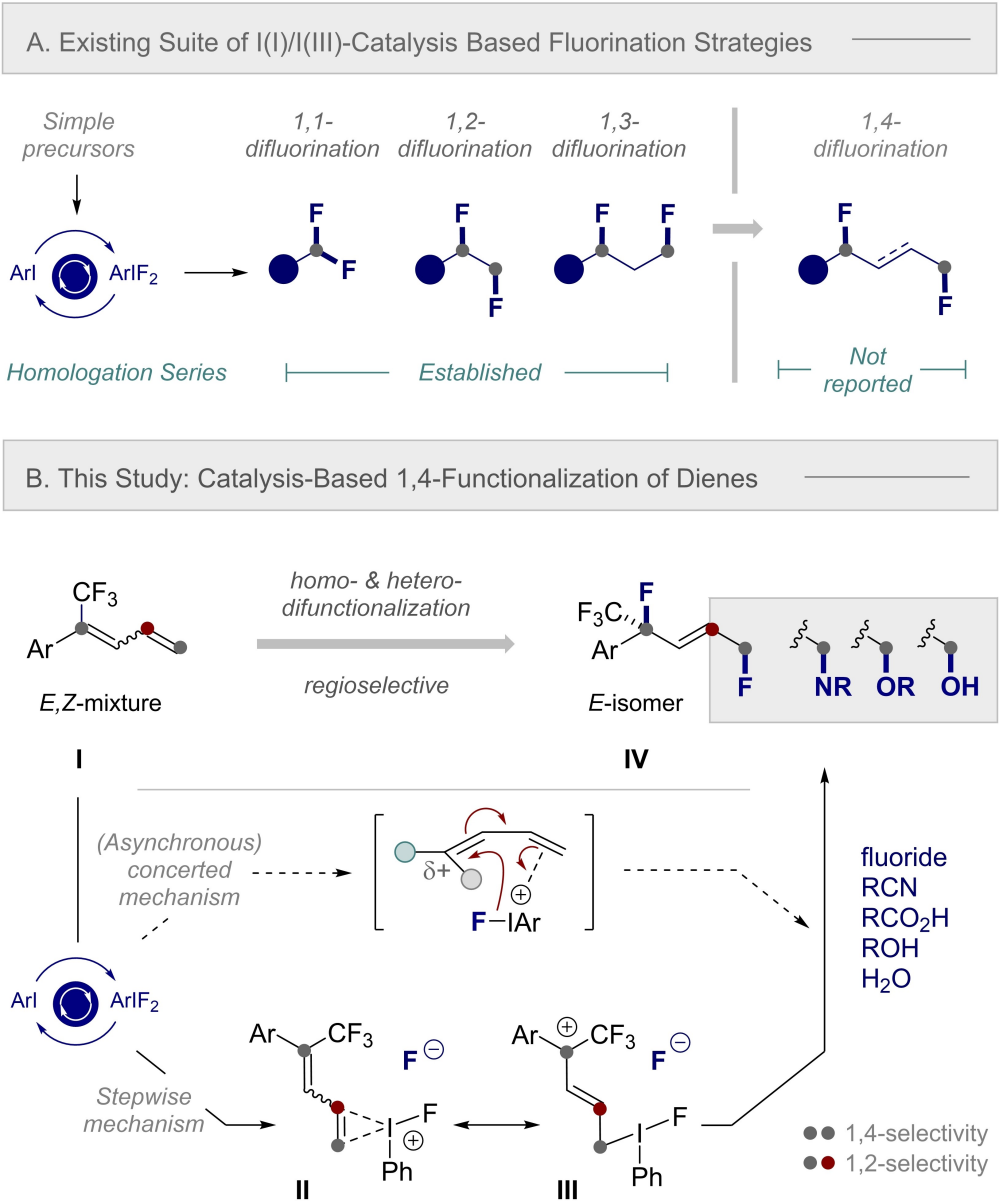
**A**. Difluorination motifs that are currently accessible via I(I)/I(III) catalysis. **B**. Reaction blueprint to enable catalysis‐based 1,4‐difluorination and fluorofunctionalization.

To establish proof of concept, the conversion of diene **1 a** to 1,4‐difluoride **2 a** was selected as a model transformation (Scheme 2). Initially, a screen of common aryl iodide catalysts was conducted in the presence of an amine⋅HF complex and Selectfluor® as the terminal oxidant. As reaction medium, CHCl_3_ was employed and full details of the solvent screen are provided in the Supporting Information. Gratifyingly, the addition of *p*‐TolI **3** (20 mol%) and an amine:HF ratio of 1 : 7.5 led to formation of the desired 1,4‐difluoride **2 a** in 92 % yield. Regioselectivity in favor of the desired 1,4‐product, over the 1,2‐difluoride, was >20 : 1, which is in‐line with the working hypothesis (Scheme 1B). A short process of catalyst structural editing revealed the following trend: **5** (*p*‐H)>**4** (*p*‐Me)>**6** (*p*‐CO_2_Me)>**3** (*p*‐OMe), thereby identifying PhI (**5**) to be most effective. Attention was then turned to further refining the process by modification of key reaction parameters (see ESI). Whereas increasing and decreasing the amine:HF ratio had little impact on the reaction outcome (entries 1 and 2), substitution of Selectfluor® for either Oxone® or *m‐*CPBA led to a reduction in yield (entries 3 and 4). Interestingly, the catalyst loading could be reduced to 10 mol% without drastically impacting the process (entry 5). Conducting the reaction at lower temperatures was found to be detrimental (entry 6) and control reactions in the absence of the catalyst and oxidant (entries 7 and 8, respectively) support the involvement of an I(I)/I(III) catalysis cycle.

**Scheme 2:**
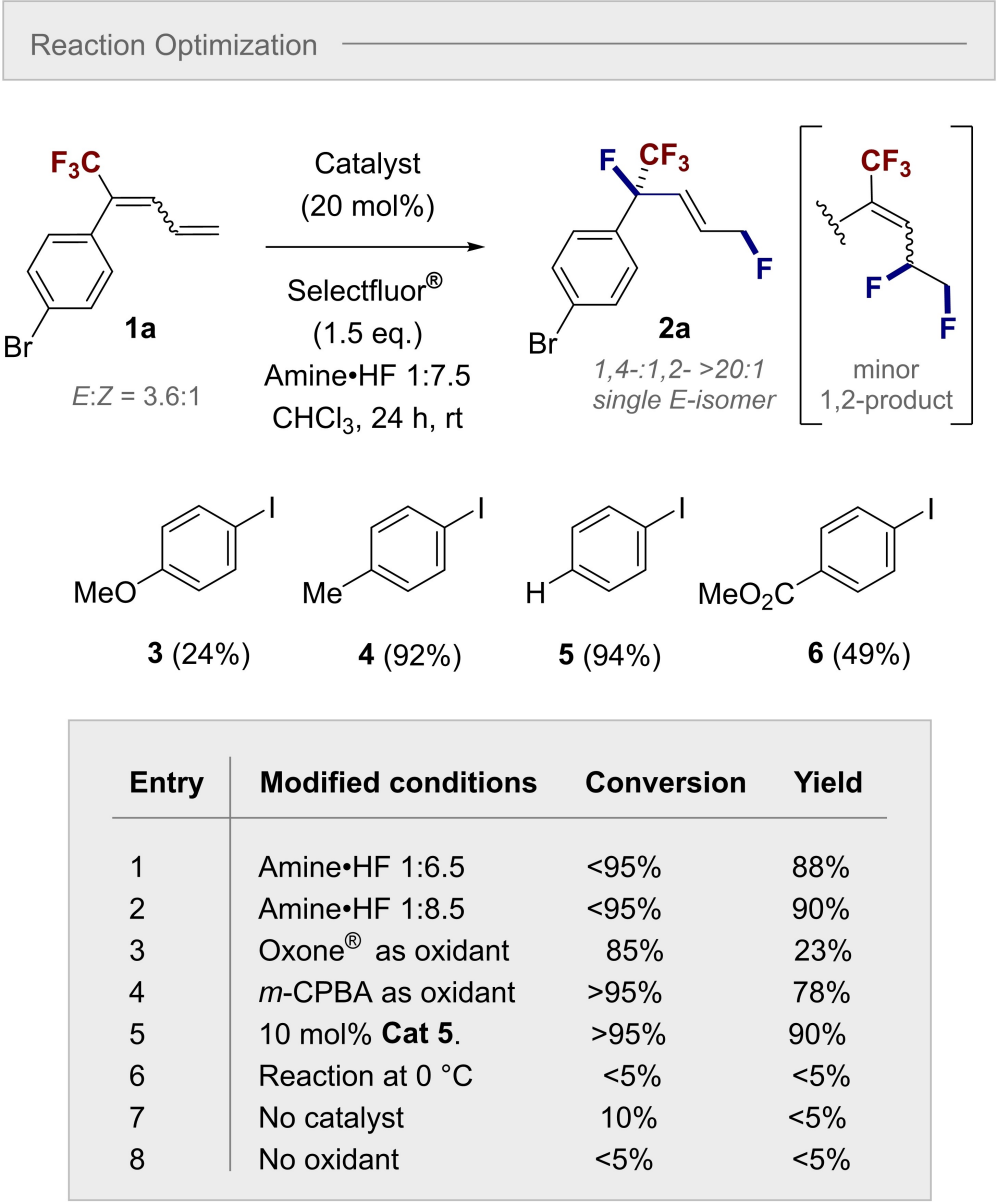
Optimization of reaction conditions. Standard reaction conditions: diene **1 a** (0.2 mmol, 3.6 : 1 *E : Z*), catalyst **3**–**6** (20 mol%), amine⋅HF 1 : 7.5 (0.5 mL), CHCl_3_ (0.5 mL) and Selectfluor® (0.3 mmol). For full optimization details, see Supporting Information. Yields and conversions determined by ^19^F NMR using trifluorotoluene as internal standard. The regioselectivity 1,4‐ versus 1,2‐ was >20 : 1 in all cases. The enantiomer of the product shown was arbitrarily chosen.

With optimized reaction conditions having been identified, attention was then turned to the scope and limitations of the 1,4‐difluorination (Scheme 3). To investigate the impact of the diene configuration on the outcome, and establish stereoconvergence with respect to the alkene component, the ***E*** **‐1 a** and ***Z*** **‐1 a** were independently exposed to the catalysis conditions: the *E*‐alkene **2 a** was produced in all cases between 89–94 % yield. For operational simplicity, the reminder of the scope study was performed with *E/Z* mixtures. Furthermore, control experiments in which the CF_3_ group was replaced by H and Me proved unsuccessful.

**Scheme 3:**
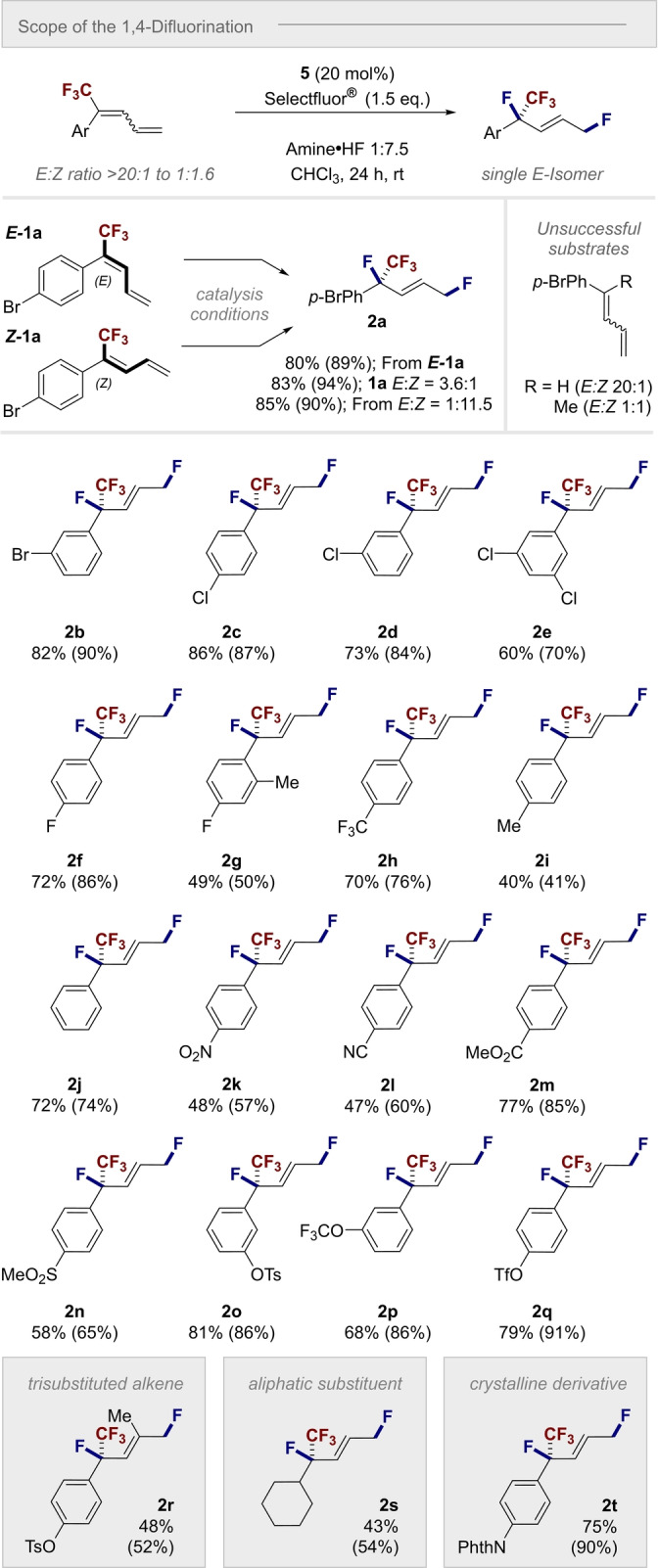
Scope of the 1,4‐difluorination. Conditions: diene **1** (0.2 mmol, *E : Z* mixture, see Supporting Information for the detailed *E : Z* ratio), catalyst **5** (20 mol%), amine ⋅ HF 1 : 7.5 (0.5 mL), CHCl_3_ (0.5 mL) and Selectfluor® (0.3 mmol). Isolated yields provided with ^19^F NMR yields given in parentheses and determined by ^19^F NMR using trifluorotoluene as internal standard. The enantiomer of the products shown was arbitrarily chosen.

The introduction of halogenated aryl groups was generally compatible (**2 b**–**2 f**) and a yield range of 70–90 % was achieved. Disubstitution was also tolerated (**2 e** and **2 g**), as was electronic modulation of the aryl ring. It is interesting to note that the electron‐deficient *p*‐CF_3_ derivative **2 h** was formed in 76 %, compared to 41 % for the *p*‐Me species **2 i**. To further aid with this evaluation, the unsubstituted derivative was investigated and found to generate **2 j** in 74 % yield. A range of electron‐deficient dienes, many of which bear handles for subsequent functionalization, were then exposed to the catalysis conditions. The 1,4‐difluorides **2 k**–**2 r** were prepared in up to 91 % yield and as a single *E*‐isomer. A valuable addition to the scope of the transformation was the finding that non‐aromatic dienes were also compatible: this enabled the cyclohexyl derived product **2 s** to be prepared in 54 % yield. Finally, phthalimide derivative **2 t** was prepared (90 %) in the hope that it might be a suitable candidate for single crystal analysis. Gratifyingly, it was possible to unequivocally establish the molecular connectivity of the product (Figure 1).^[18]^ Pertinent features include the *E*‐configured alkene and the highly pre‐organized benzylic group in which 1,3‐allylic strain is minimized:^[19]^ this closely mimics the preferred conformation of the heptafluoroisopropyl group.[^11j^, ^11k^, ^20^]

**Figure 1:**
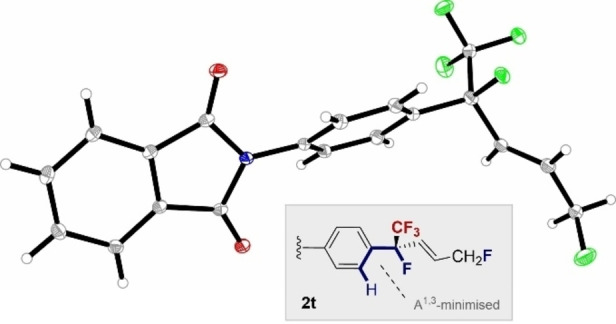
X‐ray crystal structure of compound **2 t** (CCDC 2194476).^[17]^ showing minimization of 1,3‐allylic strain, and structural pre‐organization around the benzylic region.

To rationalize the regioselectivity of the reaction, and provide support for the involvement of a transient cation (Scheme 1B), the ^13^C NMR shifts of the *ipso* positions of selected substrates in Scheme 3 were plotted against the log_10_(regioselectivity) (Figure 2). This revealed a clear correlation linking the electronic nature of the substituent with experimentally observed regioselectivity. Substrates in which more electron rich aryl substituents stabilize the benzylic cation display higher levels of 1,4‐regioselectivity: this supports the stepwise hypothesis delineated in Figure 1, although a concerted component to the mechanism cannot be fully excluded.^[21]^

**Figure 2:**
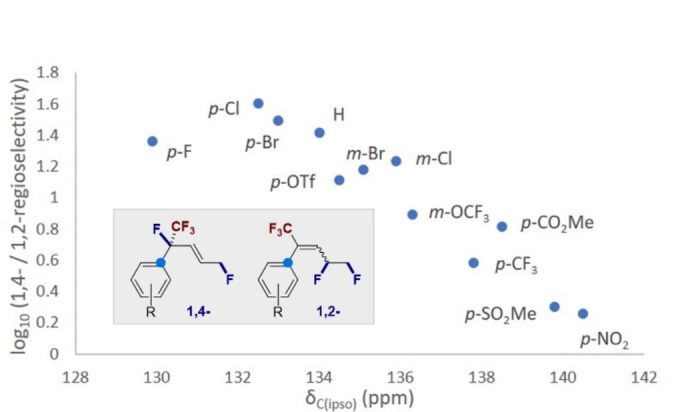
A plot of substrate regioselectivity versus δ_C(ipso)_ (ppm).

To further expand the capabilities of the methodology, and enable regiocontrolled heterodifunctionalization, the conditions were modified to include an exogenous nucleophile (Scheme 4). It was envisaged that this would facilitate direct access to highly decorated, 1,4‐difunctionalised products. Inspired by the compatibility of the Ritter reaction with hypervalent iodine catalysis,^[15]^ this led us to explore nitriles as reaction partners.

**Scheme 4:**
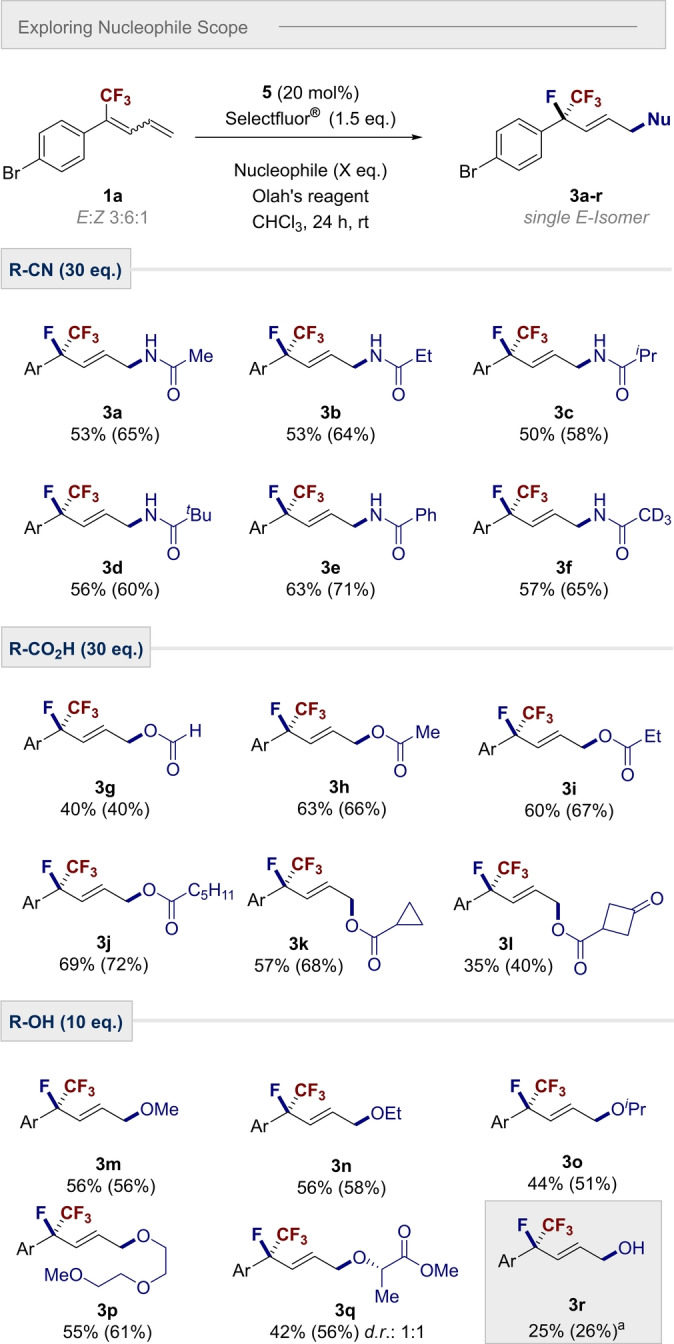
Scope of different nucleophiles: Conditions: diene **1 a** (0.2 mmol), catalyst **5** (20 mol%), Olah's reagent (0.5 mL), CHCl_3_ (0.5 mL), nucleophile (as indicated) and Selectfluor® (0.3 mmol). Isolated yields are given, ^19^F NMR yield is given in parentheses and determined by ^19^F NMR using trifluorotoluene as internal standard. ^a^ H_2_O (20 equiv) was used as nucleophile. The enantiomer of the products shown was arbitrarily chosen.

As an initial proof of concept, acetonitrile was employed and this led to the formation of the desired 1,4‐aminofluorinated product **3 a** (65 %). Substituting acetonitrile for various other alkyl nitriles proved successful, enabling products **3 b** (Et), **3 c** (^*i*^Pr), **3 d** (^*t*^Bu) and **3 f** (CD_3_) to be prepared in up to 71 % yield. Moreover, benzonitrile was also well‐tolerated, to generate product **3 e**. Replacing nitriles by carboxylic acids as coupling partners was also successful, enabling a regioselective 1,4‐oxyfluorination of dienes. The initial transformation with formic acid, to generate product **3 g** (40 %), was generalized to enable a series of highly functionalized building blocks as is evident from products **3 h**–**k** (up to 72 %). Finally, the generation of ether products was realised by leveraging alcohols as nucleophiles in the title reaction. The introduction of simple alcohols such as methanol, ethanol and ^*i*^PrOH furnished ethers **3 m**, **3 n** and **3 o**, respectively (up to 58 %). Furthermore, the process was compatible with polyoxygenated alcohol **3 p**, and no loss of stereochemical integrity was observed with the chiral pool derived lactate **3 q** (56 %). In pushing the boundaries of the process, it was noted that water could be used directly to enable a 1,4‐fluorohydroxylation (**3 r**), albeit in a moderate yield.

Finally, the generation of building block **2 a** was scaled to 3 mmol without loss of catalysis efficiency (85 % yield). To demonstrate the synthetic utility of the transformation, the products of homo‐ and hetero‐difuctionalization (**2 a** and **3 h**/**3 r**, respectively) were further derivatized (Scheme 5). Treatment of **2 a** with bromine in refluxing CH_2_Cl_2_ resulted in the formation of the allyl bromide **4 a** in 61 % isolated yield. An ozonolysis/reductive quench of the alkene moiety of **2 a**, enabled the alcohol **4 b** to be generated in 58 % yield. Alkene epoxidation also proved facile in the presence of *m*‐CPBA to generate **4 c**. To access the allylic bromide **4 d**, compound **3 h** was processed via a two‐step saponification, Appel reaction sequence (98 % yield over two steps). Finally, formation of the α,β‐unsaturated carboxylic acid was achieved by exposing **3 r** to MnO_2_ followed by Pinnick oxidation of the intermediate aldehyde (80 %, 2 steps).

**Scheme 5:**
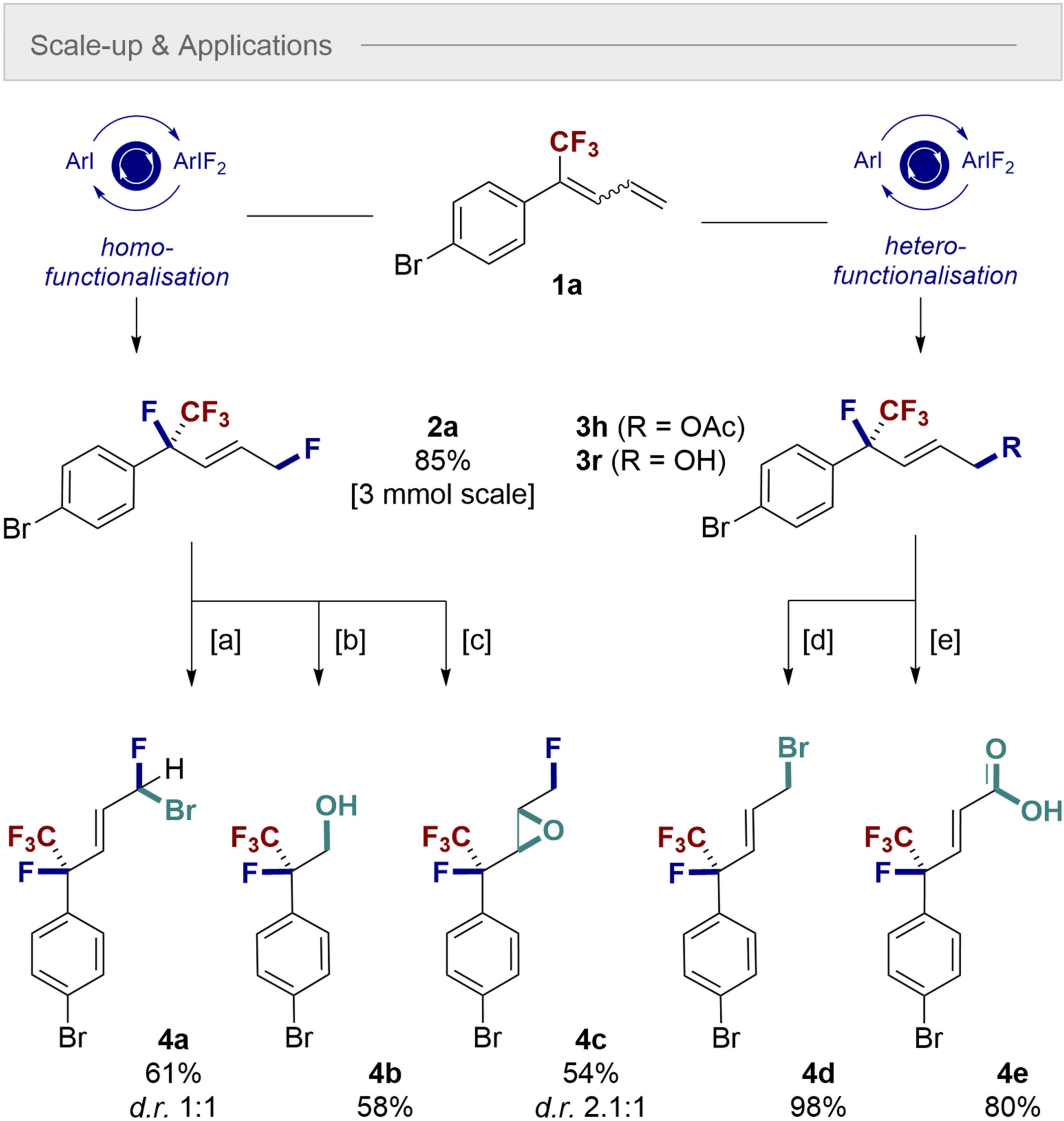
Scale‐up experiment and selective derivatization of **2 a**, **3 h** and **3 r**. [a] Br_2_ (2.0 equiv), DCM, reflux, 24 h; [b] 1) O_3_; 2) NaBH_4_ (20 equiv), DCM:MeOH (1 : 1), −78 °C to rt, 27 h; [c] *m*‐CPBA (6 equiv), DCE, 90 °C, 48 h; [d] K_2_CO_3_ (2.0 equiv), MeOH, rt, 1 h; 2) CBr_4_ (1.5 equiv), PPh_3_ (1.5 equiv), DCM, 0 °C to rt, 0.5 h; [e] 1) MnO_2_ (10 equiv), DCM, reflux, 24 h; 2) NaClO_2_ (10 equiv), NaH_2_PO_4_⋅2H_2_O (7 equiv), 2‐methyl‐2‐butene/^*t*^BuOH/H_2_O, rt, 20 h.

In conclusion, the first catalysis‐based regioselective 1,4‐difluorination of dienes has been achieved under the auspices of iodine(I)/iodine(III) catalysis. The platform has been further developed to encapsulate 1,4‐heterodifunctionalization and enable the direct formation of δ‐fluoro‐alcohol and amine derivatives in a single operation. High levels of regiocontrol are observed (up to 1,4‐:1,2‐>20 : 1), and the presence of an α‐CF_3_ group in the product locks in a stable tertiary C(sp^3^)‐F center. In addition to addressing a strategic gap in the fluorination arsenal, it is envisaged that this enabling transformation will serve an important role in expanding organofluorine chemical space. Efforts to render the process enantioselective are ongoing and will be reported in due course.

## Conflict of interest

The authors declare no conflict of interest.

## Supporting information

As a service to our authors and readers, this journal provides supporting information supplied by the authors. Such materials are peer reviewed and may be re‐organized for online delivery, but are not copy‐edited or typeset. Technical support issues arising from supporting information (other than missing files) should be addressed to the authors.

Supporting Information

Supporting Information

## Data Availability

The data that support the findings of this study are available from the corresponding author upon reasonable request.
