# Supplementary material for: Catalytic, Regioselective 1,4‐Fluorodifunctionalization of Dienes
Source: Angew Chem Int Ed Engl. 2022 Dec 1;62(1):e202214906. doi: 10.1002/anie.202214906 (PMC10107283; doi:10.1002/anie.202214906)
Supplement: Supplementary file 1 — Supporting Information [file ANIE-62-0-s002.pdf]

Angewandte Chemie
Eine Zeitschrift der Gesellschaft Deutscher Chemiker

Supporting Information

Catalytic, Regioselective 1,4-Fluorodifunctionalization of Dienes

*Y.-J. Yu, M. Schäfer, C. G. Daniliuc, R. Gilmour**

Table of Contents

1. General information....3
2. Synthesis of starting materials....5
3. 1,4-Difluorination reactions....53
    3.1 Optimization of reaction conditions....53
    3.2 Catalytic 1,4-difluorination reactions....54
    3.3 Raw data for the plot of substrate regioselectivity versus $\delta_{C(ipso)}$ (ppm)....75
4. 1,4-Fluorofunctionalization reactions....75
    4.1 Optimization of reaction conditions....75
    4.2 Catalytic 1,4-fluorofunction reactions....80
5. Synthetic applications....99
    5.1 Large scale reaction....99
    5.2 Transformation of allylic fluorides....100
6. X-ray crystallographic data....109
7. NMR-Spectra of key compounds....111
8. References....269

1. General information

All commercially available reagents were purchased as reagent grade from *Sigma Aldrich, Merck, Alfa Aesar, TCI, Fluorochem* or *abcr* and were used without further purification unless otherwise stated. Solvents for extractions or chromatographic purifications were bought as technical grade and distilled on a rotary evaporator prior to use. All reactions with HF were run in Teflon® vials.. For analytical thin layer chromatography, glass plates coated with SiO_2-60 F254 were used from *Merck*. They were visualized with UV-light (254 nm) or with $KMnO_4$ or CAM solution. Column chromatography was performed using silica gel (40-63 μm, *VWR Chemicals*). For preparative thin layer chromatography, glass plates coated with SiO_2-60 F254 and 2 mm thickness were used from *Merck*. The obtained products are often volatile and care must be taken in the isolation. The NMR measurements were performed on a *Bruker AV300, AV400, Agilent DD2 500* or an *Agilent DD2 600* by the NMR service department of the Organisch-Chemisches Institut, Westfälische Wilhelms-Universität Münster. The chemical shifts were referenced to the residual solvent peak as the internal standard (7.26 ppm for $CDCl_3$, 2.50 ppm for DMSO-d_6 for ^{1}H-NMR, 77.16 ppm for $CDCl_3$, 39.52 ppm for DMSO-d_6 for ^{13}C-NMR). The multiplicity is abbreviated as follows: s (singlet), d (doublet), t (triplet), q (quartet), p (pentet), h (heptet), m (multiplet) and br (broad). The given assignments are supported by additional 1D and 2D NMR experiments. The melting points were determined on a *Büchi* B-545 melting point apparatus with open glass capillaries. The IR measurements were performed on a *Perkin-Elmer 100 FT-IR* spectrometer and the intensities of the bands are assigned as follows: w (weak), m (medium), s (strong). High resolution mass spectrometry was performed by the MS service of the Organisch-Chemisches Institut, Westfälische Wilhelms-Universität Münster on a Bruker Daltonics MicroTof (HRMS-ESI), a Triplequad TSQ 7000 (MS-EI), Triplequad Quattro Micro GC (GC-EI-MS), a Qp5050 Single Quad (GC-EI-MS) or a LTQ Orbitap LTQ XL (HRMS-APCI).

Preparation of various amine•HF mixtures

Amine·HF sources:

NEt_3·3HF; Supplier: abcr; (MW: 161.21g/mol, ρ = 0.990 g/mL)
Olah's Reagent (70wt% HF: Py·9.23HF); Supplier: Sigma Aldrich; (MW: 263.79 g/mol, ρ = 1.1 g/mL)

Procedure for calculating compositions of amine·HF mixtures:

The amine•HF mixtures based on NEt_3•3HF and Olah´s reagent were prepared following the procedure previously described by this group.[1]

2. Synthesis of starting materials

General procedure A

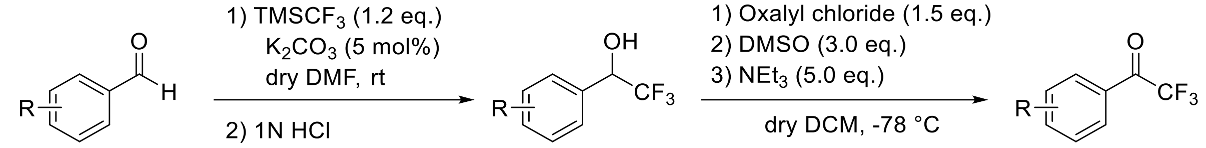

According to a modified literature procedure,[2] aldehyde (1.0 eq.), $TMSCF_3$ (1.2 eq.) and dry DMF (0.5 M) were added to a dry round bottom flask. K_2CO_3 (0.05 eq.) was added in one portion then the reaction mixture was allowed to stir at room temperature until TLC indicated consumption of the aldehyde. The mixture was quenched with aqueous 1 N HCl and stirred vigorously until full conversion of the silyl ether as judged by TLC. EtOAc and water were added and the organics were extracted with EtOAc (3x) and washed with brine (1x). The combined organic layers were dried over Na_2SO_4, the solvent was evaporated under reduced pressure and the crude residue was directly used in next step without further purification.

According to a modified literature procedure,[3] oxalyl chloride (1.5 eq.) was added dropwise to a -78 °C solution of DMSO (3.0 eq.) in DCM (0.5 M). The resulting solution was stirred for an additional 10 minutes and a solution of the alcohol (1.0 eq.) in DCM was added dropwise. The resulting solution was stirred for 30 minutes and triethylamine (5.0 eq.) was added. The resulting solution was stirred for 30 minutes at -78 °C and the cooling bath was removed. After an additional 30 minutes, the reaction mixture was quenched with saturated aqueous NH_4Cl and extracted with DCM (3x). The combined organic layers were dried over Na_2SO_4, the solvent was evaporated under reduced pressure and the crude residue was directly used in next step without further purification.

General procedure B

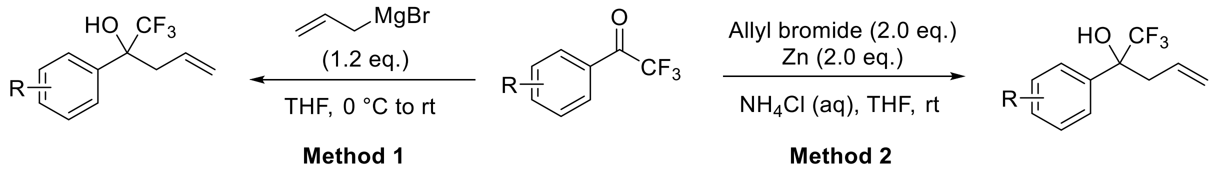

Method 1: According to a modified literature procedure,[4] the corresponding phenyl-2,2,2-trifluoroethan-1-one derivative (1.0 eq.) was dissolved in dry THF in a dry round bottom flask under an argon atmosphere. Allylmagnesium bromide (1.2 eq.) was added

dropwise at 0 °C, and then the reaction mixture was allowed to stir at room temperature until TLC indicated consumption of the ketone. After completion of the reaction, 1 N HCl was added. The reaction mixture was extracted with EtOAc (3x) and washed with brine (1x). The combined organic layers were dried over Na_2SO_4, the solvent was evaporated under reduced pressure and the crude residue was purified by column chromatography (SiO_2, specified combination of solvents) to give the allyl alcohol.

Method 2: According to a modified literature procedure,[5] the corresponding phenyl-2,2,2-trifluoroethan-1-one derivative (1.0 eq.), allyl bromide (2.0 eq.), NH_4Cl (sat.) (45 mL) and THF (9 mL) were added to a round bottom flask. Zn dust (2.0 eq.) was added in one portion, then the reaction mixture was allowed to stir at room temperature until TLC indicated consumption of the ketone. After work up as described above, the residue was purified by flash chromatography (SiO_2, specified combination of solvents) to give the allyl alcohol.

General procedure C

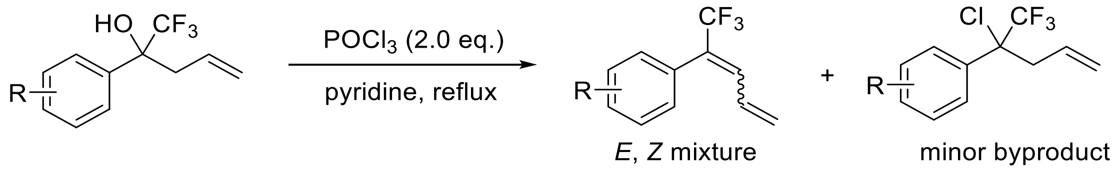

According to a modified literature procedure,[4] the allyl alcohol was dissolved in pyridine (0.2 M), and $POCl_3$ (2.0 eq.) was added. The reaction mixture was stirred at 110 °C. Upon completion of the reaction, the resulting mixture was cooled to room temperature, and then treated with a saturated solution of $NaHCO_3$, extracted with EtOAc (2x) and washed with 1 N HCl (2x). The crude material was dried over Na_2SO_4, filtered and evaporated followed by silica gel column chromatography (SiO_2, specified combination of solvents) to give the diene as a mixture of isomers (*E*/*Z*, the ratio of which was determined by ^{19}F NMR), which could not be separated by silica gel chromatography or preparative TLC.

Caution: For some substrates, a small amount of chlorinated product was isolated together with the diene products, which cannot be separated by silica gel chromatography. In these cases, the mixture was dissolved in dry THF (0.2 M), *t*-BuOK (2.0 eq. relative to the

chlorinated product) was added, and the reaction mixture was allowed to stir at room temperature for 5 h. After usual work up, the residue was purified by flash chromatography to give the pure diene product.

Synthesis of pure (*E*)-1a and pure (*Z*)-1a

According to a modified literature procedure,[6] pure *E* or *Z* diene was synthesized from the corresponding aryl ketones by a four-step sequence: Horner-Wadsworth-Emmons olefination / DIBAL-H reduction / MnO_2 oxidation / Wittig reaction.

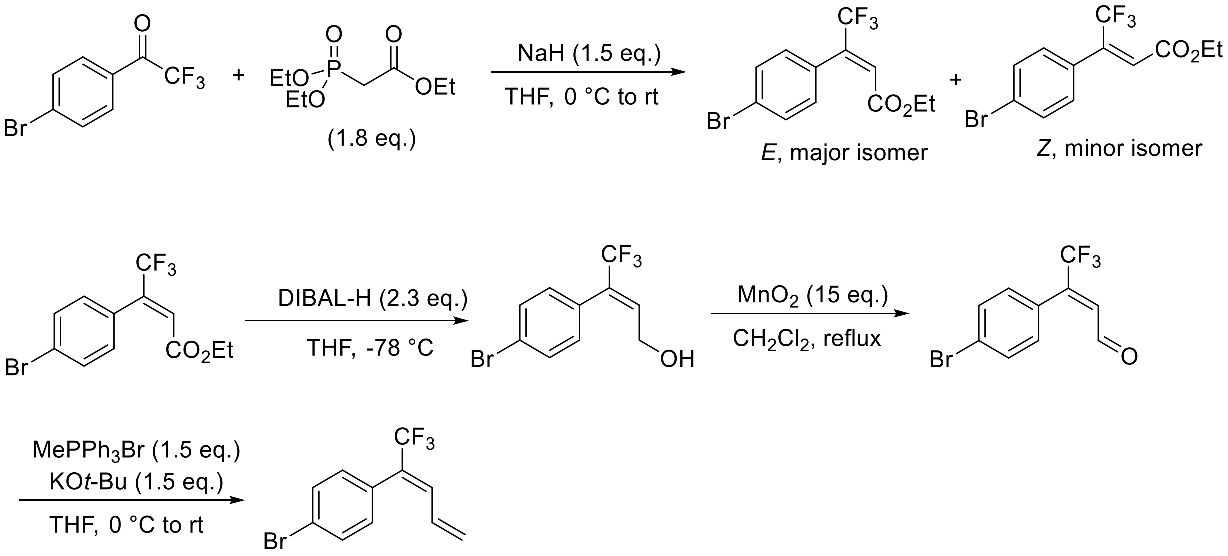

Step 1: To a 100-mL round bottom flask containing NaH (60 mmol, 60% mineral dispersion, 1.5 eq.) and anhydrous THF (40 mL) at 0 °C was added triethyl phosphonoacetate (72 mmol, 1.8 eq.) dropwise. The reaction mixture was allowed to stir for 30 min at the same temperature, followed by a dropwise addition of a solution of 4'-bromo-2,2,2-trifluoroacetophenone (40 mmol, in 20 mL anhydrous THF, 1 eq.). The reaction mixture was stirred while monitored by TLC until the ketone was consumed. Water (40 mL) was slowly added and the reaction mixture was extracted with EtOAc (3x). The organic layers were combined and dried over Na_2SO_4, filtered, and concentrated under reduced pressure. The crude residue was subjected to flash chromatography (SiO_2, specified combination of solvents) to afford the corresponding α,β-unsaturated ester in an isomerically pure form.

Step 2: The unsaturated ester (1.0 eq.) was dissolved in dry THF (0.5 M) under argon before being cooled to −78 °C. DIBAL-H (1.0 M in toluene, 2.3 eq.) was added dropwise and the resulting mixture was stirred at −78 °C for 30 min, and then the reaction mixture was allowed to stir at 0 °C until TLC indicated consumption of the ester. The reaction was quenched by dropwise addition of a saturated aqueous solution of Rochelle's salt. The organic layer was separated and the aqueous layer was extracted with EtOAc (3x). The combined organic layers were dried over Na_2SO_4, filtered, and concentrated under reduced pressure. The crude residue was directly used in the next step without further purification.

Step 3: To a 100-mL round bottom flask containing the allylic alcohol (1.0 eq.) obtained above, was added activated MnO_2 (15 eq.) and anhydrous CH_2Cl_2 (25 mL) at room temperature. The reaction mixture was then heated to reflux and stirred for at least 24 h, while being monitored by TLC. After complete consumption of the starting material, the reaction mixture was filtered through a pad of celite and rinsed by CH_2Cl_2. The resulting filtrate was concentrated under reduced pressure. The crude residue was directly used in the next step without further purification.

Step 4: To a 100-mL round bottom flask containing anhydrous THF (0.3 M) at 0 °C was added Ph_3PMeBr (1.5 eq.). *t*-BuOK (1.5 eq.) was added portion-wise and the resulting yellow solution was stirred for 0.5 h before cooling to 0 °C. The β-aryl-β-CF_3 enal (1.0 eq.) obtained above was added dropwise and the reaction mixture was allowed to stir at ambient temperature until full conversion of the silyl ether, as shown by TLC. Et_2O and water were added and the organics were extracted with Et_2O (3x) and washed with brine (1x). The combined organic layers were dried over Na_2SO_4, filtered, the solvent was evaporated under reduced pressure and the crude residue was purified by column chromatography (SiO_2, specified combination of solvents) to afford the corresponding diene in an isomerically pure form.

Ethyl (*E*)-3-(4-bromophenyl)-4,4,4-trifluorobut-2-enoate

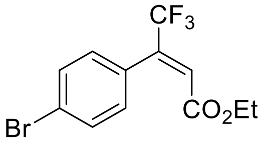

Ethyl (*E*)-3-(4-bromophenyl)-4,4,4-trifluorobut-2-enoate was prepared according to Step 1 and was obtained as a colorless liquid (9.66 g, 29.90 mmol, 75%) after column chromatography on silica gel (*n*-pentane:EtOAc 400:1).

R$_f$ = 0.61 (*n*-pentane:EtOAc 20:1).

^{1}H NMR (599 MHz, $CDCl_3$): δ [ppm] = 8.00 – 7.46 (m, 2H), 7.22 – 7.12 (m, 2H), 6.62 (q, *J* = 1.4 Hz, 1H), 4.07 (q, *J* = 7.2 Hz, 2H), 1.11 (t, *J* = 7.1 Hz, 3H).

^{19}F NMR (564 MHz, $CDCl_3$): δ [ppm] = -67.61 (s, 3F).

^{19}F{^{1}H} NMR (564 MHz, $CDCl_3$): δ [ppm] = -67.61 (s, 3F).

GC-EI-MS: (*m/z*) requires: $[(C_{12}H_{10}BrF_3O_2)^+]$ = 323.9791, (*m/z*) found: $[(C_{12}H_{10}BrF_3O_2)^+]$ = 323.9793.

The analytical data were in good agreement with the literature.[7]

(*E*)-1-Bromo-4-(1,1,1-trifluoropenta-2,4-dien-2-yl)benzene (*E*-1a)

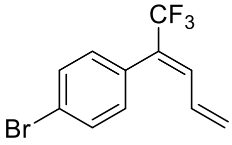

Compound ***E*-1a** was prepared according Steps 2-4 using ethyl (*E*)-3-(4-bromophenyl)-4,4,4-trifluorobut-2-enoate (4.91 g, 14.89 mmol, 1.0 eq.). The crude residue was purified by column chromatography (*n*-pentane) to yield the title compound as a colorless oil (1.32 g, 4.76 mmol, 32% over three steps).

R$_f$ = 0.72 (100% *n*-pentane).

^{1}H NMR (500 MHz, $CDCl_3$): δ [ppm] = 7.67 – 7.44 (m, 2H), 7.24 – 7.09 (d, *J* = 8.6 Hz, 2H), 6.87 (dqd, *J* = 11.2, 1.7, 0.9 Hz, 1H), 6.40 – 6.09 (m, 1H), 5.61 (ddq, *J* = 16.9, 2.0, 1.0 Hz, 1H), 5.42 (ddq, *J* = 10.2, 1.6, 0.8 Hz, 1H).

^{19}F NMR (470 MHz, $CDCl_3$): δ [ppm] = -65.26 (s, 3F).

^{19}F{^{1}H} NMR (470 MHz, $CDCl_3$): δ [ppm] = -65.26 (s, 3F).

GC-EI-MS: (*m/z*) requires: [$(C_{11}H_8BrF_3)^+$] = 275.9756, (*m/z*) found: [$(C_{11}H_8BrF_3)^+$] = 275.9758.

The analytical data were in good agreement with the literature.[8]

Ethyl (*Z*)-3-(4-bromophenyl)-4,4,4-trifluorobut-2-enoate

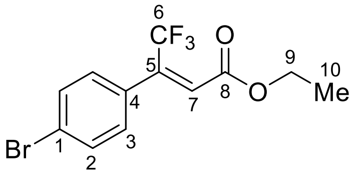

Ethyl (*Z*)-3-(4-bromophenyl)-4,4,4-trifluorobut-2-enoate was obtained as the minor product using Step 1 and isolated as a colorless liquid (1.86 g, 5.77 mmol, 14%) after column chromatography on silica gel (*n*-pentane:EtOAc 100:1).

R$_f$ = 0.51 (*n*-pentane:EtOAc 20:1).

FT-IR ($\tilde{\nu}$ = cm^{-1})**:** 2986 (w), 1733 (m), 1651 (w), 1589 (w), 1492 (m), 1447 (w), 1367 (m), 1276 (m), 1258 (m), 1167 (s), 1132 (s), 1076 (m), 1026 (m), 1013 (m), 984 (m), 920 (m), 887 (m), 823 (m), 749 (m), 720 (m), 693 (m), 659 (m).

^{1}H NMR (599 MHz, $CDCl_3$) δ [ppm] = 7.57 – 7.51 (m, 2H, H-C3), 7.34 – 7.26 (m, 2H, H-C2), 6.33 (s, 1H, H-C7), 4.31 (q, $^3J_{HH}$ = 7.1 Hz, 2H, H-C9), 1.34 (t, 1H, $^3J_{HH}$ = 7.1 Hz, 3H, H-C10).

^{13}C NMR (151 MHz, $CDCl_3$): δ [ppm] = 164.5 (C8), 136.8 (q, $^2J_{CF}$ = 32.3 Hz, C5), 132.5 (q, $^3J_{CF}$ = 1.4 Hz, C4), 132.1 (C2), 129.5 (C3), 128.1 (q, $^3J_{CF}$ = 3.5 Hz, C7), 124.2 (C1), 122.1 (q, $^1J_{CF}$ = 275.8 Hz, C6), 62.0 (C9), 14.1 (C10).

^{19}F NMR (564 MHz, $CDCl_3$): δ [ppm] = -60.26 (s, 3F, F-C6).

^{19}F{^{1}H} NMR (564 MHz, $CDCl_3$): δ [ppm] = -60.26 (s, 3F, F-C6).

GC-EI-MS: (*m/z*) requires: [$(C_{12}H_{10}BrF_3O_2)^+$] = 321.9811, (*m/z*) found: [$(C_{12}H_{10}BrF_3O_2)^+$] = 321.9811.

(*Z*)-1-Bromo-4-(1,1,1-trifluoropenta-2,4-dien-2-yl)benzene (*Z* -1a)

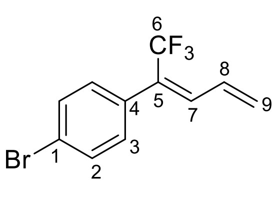

Compound ***Z*-1a** was prepared according to Steps 2-4 using ethyl (*Z*)-3-(4-bromophenyl)-4,4,4-trifluorobut-2-enoate (0.64 g, 1.98 mmol,

1.0 eq.). The crude residue was purified by column chromatography (*n*-pentane) to yield the title compound as a colorless oil (0.18 g, 0.65 mmol, *E*:*Z* = 1:11.5, 33% over three steps).

Cautions: Due to the isomerization of allyl alcohol during oxidation step (Step 3), the product was mixed with a small amount of ***E*-1a**.

R_f = 0.72 (100% *n*-pentane).

FT-IR ($\tilde{\nu}$ = cm^{-1})**:** 3457 (w), 3016 (w), 2970 (m), 1739 (s), 1586 (w), 1488 (m), 1425 (w), 1365 (s), 1271 (m), 1228 (m), 1216 (m), 1202 (s), 1173 (m), 1149 (s), 1114 (s), 1073 (m), 1011 (m), 988 (m), 964 (m), 930 (m), 914 (s), 823 (s), 771 (m), 730 (w), 718 (m), 696 (w), 651 (w), 623 (m), 520 (m).

^{1}H NMR (599 MHz, $CDCl_3$): δ [ppm] = 7.53 – 7.47 (m, 2H, H-C3), 7.23 (d, $^{3}J_{HH}$ = 8.2 Hz, 2H, H-C2), 6.93 (dddq, $^{3}J_{HH}$ = 16.6 Hz, $^{3}J_{HH}$ = 12.0 Hz, $^{3}J_{HH}$ = 10.0 Hz, $^{4}J_{HF}$ = 2.3 Hz, 1H, H-C8), 6.51 (d, $^{3}J_{HH}$ = 11.3 Hz, 1H, H-C7), 5.56 (d, 1H, $^{3}J_{HH}$ = 16.6 Hz, 1H, H^a-C9), 5.55 (d, 1H, $^{3}J_{HH}$ = 10.0 Hz, 1H, H^b-C9).

^{13}C NMR (151 MHz, $CDCl_3$): δ [ppm] = 138.7 (q, $^{3}J_{CF}$ = 2.9 Hz, C7), 135.1 (q, $^{3}J_{CF}$ = 1.7 Hz, C4), 131.7 (C3), 131.5 (q, $^{4}J_{CF}$ = 2.8 Hz, C8), 129.9 (C2), 129.3 (q, $^{2}J_{CF}$ = 30.6 Hz, C5), 126.4 (C9), 122.8 (q, $^{1}J_{CF}$ = 275.4 Hz, C6), 122.8 (C1).

^{19}F NMR (564 MHz, $CDCl_3$): δ [ppm] = -56.71 (s, 3F, F-C6, ***Z*-1a**).

^{19}F{^{1}H} NMR (564 MHz, $CDCl_3$): δ [ppm] = -56.71 (s, 3F, F-C6, ***Z*-1a**).

GC-EI-MS: (*m/z*) requires: [$(C_{11}H_8BrF_3)^+$] = 275.9756, (*m/z*) found: [$(C_{11}H_8BrF_3)^+$] = 275.9756.

2-(4-Bromophenyl)-1,1,1-trifluoropent-4-en-2-ol (1a-1)

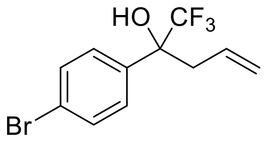

Compound **1a-1** was prepared according to **General Procedure B** (**Method 1**) using 1-(4-bromophenyl)-2,2,2-trifluoroethan-1-one (7.40 g, 29.25 mmol, 1.0 eq.). The crude residue was purified by column chromatography (*n*-pentane:DCM 6:1) to yield the title compound as a colorless oil (7.68 g, 26.02 mmol, 89%).

R_f = 0.66 (*n*-pentane:EtOAc 6:1).

^{1}H NMR (400 MHz, $CDCl_3$): δ [ppm] = 7.58 – 7.49 (m, 2H), 7.45 (d, *J* = 8.6 Hz, 2H), 5.66 – 5.45 (m, 1H), 5.34 – 5.17 (m, 2H), 2.93 (dd, *J* = 14.4, 6.6 Hz, 1H), 2.83 (dd, *J* = 14.3, 7.9 Hz, 1H), 2.65 (s, 1H).

^{19}F NMR (377 MHz, $CDCl_3$): δ [ppm] = -79.30 (s, 3F).

^{19}F{^{1}H} NMR (377 MHz, $CDCl_3$): δ [ppm] = -79.30 (s, 3F).

GC-EI-MS: (*m/z*) requires: $[(C_{11}H_{10}BrF_3O)^+]$ = 293.9862, (*m/z*) found: $[(C_{11}H_{10}BrF_3O)^+]$ = 293.9848.

The analytical data were in good agreement with the literature.[9]

1-Bromo-4-(1,1,1-trifluoropenta-2,4-dien-2-yl)benzene (1a)

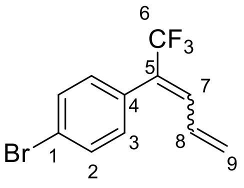

E/*Z* = 3.6:1

Compound **1a** was prepared according to **General Procedure C** using 2-(4-bromophenyl)-1,1,1-trifluoropent-4-en-2-ol **(1a-1)** (3.54 g, 12.00 mmol, 1.0 eq.). The crude residue was purified by column chromatography (*n*-pentane) to yield the title compound as a colorless oil (2.32 g, 8.37 mmol, 70%, *E*:*Z* = 3.6:1).

R_f = 0.72 (*n*-pentane)

FT-IR ($\tilde{\nu}$ = cm^{-1})**:** 1868 (w), 1648 (w), 1588 (w), 1491 (m), 1424 (w), 1395 (w), 1365 (m), 1275 (m), 1202 (m), 1172 (m), 1144 (m), 1114 (s), 1073 (m), 1144 (m), 1114 (s), 1072 (m), 1013 (m), 993 (m), 955 (m), 928 (s), 916 (m), 825 (s), 760 (m), 730 (m), 700 (w), 660 (m).

^{1}H NMR (500 MHz, $CDCl_3$):

E-diene: δ [ppm] = 7.58 – 7.53 (m, 2H, H-C3), 7.20 – 7.08 (m, 2H, H-C2), 6.87 (dqd, $^3J_{HH}$ = 11.0 Hz, $^4J_{HF}$ = 1.6 Hz, $^4J_{HH}$ = 0.8 Hz, 1H, H-C7), 6.33 – 6.14 (m, 1H, H-C8), 5.61 (ddq, $^3J_{HH}$ = 16.9 Hz, $^2J_{HH}$ = 1.7 Hz, $^6J_{HF}$ = 1.6 Hz, 1H, H^a-C9), 5.42 (dddq, $^3J_{HH}$ = 10.2 Hz, $^2J_{HH}$ = 1.6 Hz, $^4J_{HH}$ = 0.8 Hz, $^6J_{HF}$ = 0.8 Hz, 1H, H^b-C9).

Z-diene: δ [ppm] = 7.52 – 7.45 (m, 2H, H-C3), 7.23 (dq, $^3J_{HH}$ = 7.7 Hz, $^5J_{HF}$ = 0.9 Hz, 2H, H-C2), 7.01 – 6.88 (m, 1H, H-C8), 6.51 (dq, $^3J_{HH}$ = 11.5 Hz, $^4J_{HF}$ = 0.8 Hz, 1H, H-C7), 5.59 – 5.56 (d, 1H, $^3J_{HH}$ = 16.6 Hz, 1H, H^a-C9), 5.55 (d, 1H, $^3J_{HH}$ = 10.0 Hz, 1H, H^b-C9).

^{13}C NMR (126 MHz, $CDCl_3$):

E-diene: δ [ppm] = 134.3 (q, $^3J_{CF}$ = 5.9 Hz, C7), 131.9 (C3), 131.7 (C2), 131.4 (C8), 131.0 (q, $^3J_{CF}$ = 0.8 Hz, C4), 130.0 (q, $^2J_{CF}$ = 30.2 Hz, C5), 125.7 (q, $^5J_{CF}$ = 1.2 Hz, C9), 123.6 (q, $^1J_{CF}$ = 272.4 Hz, C6), 123.3 (C1).

Note: As the limited quantity of *Z* isomer prohibits ^{13}C NMR analysis, only the *E*-diene is characterized. This applies to all *E*,*Z*-mixed starting materials.

^{19}F NMR (470 MHz, $CDCl_3$): *E*-diene: δ [ppm] = -65.26 (s, 3F, F-C6); *Z*-diene: δ [ppm] = -56.69 (d, $^4J_{HF}$ = 2.1 Hz, 3F, F-C6)

^{19}F{^{1}H} NMR (470 MHz, $CDCl_3$): *E*-diene: δ [ppm] = -65.26 (s, 3F, F-C6); *Z*-diene: δ [ppm] = -56.69 (s, 3F, F-C6)

GC-EI-MS: (*m/z*) requires: [$(C_{11}H_8BrF_3)^+$] = 275.9756, (*m/z*) found: [$(C_{11}H_8BrF_3)^+$] = 275.9755.

2-(3-Bromophenyl)-1,1,1-trifluoropent-4-en-2-ol (1b-1)

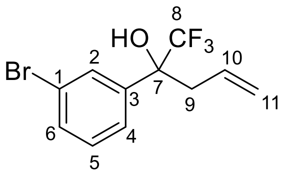

Compound **1b-1** was prepared according to **General Procedure A and B** (**Method 1**) using 3-bromobenzaldehyde (1.85 g, 10.00 mmol, 1.0 eq.). The crude residue was purified by column chromatography (*n*-pentane:EtOAc 50:1) to yield the title compound as a colorless oil (1.88 g, 6.48 mmol, 65%).

R_f = 0.69 (*n*-pentane:EtOAc 6:1).

FT-IR ($\tilde{\nu}$ = cm^{-1})**:** 3543 (w), 3079 (w), 1642 (w), 1596 (w), 1569 (w), 1476 (w), 1445 (w), 1421 (w), 1369 (w), 1265 (m), 1230 (m), 1156 (s), 1076 (m), 1018 (m), 996 (m), 954 (m), 924 (m), 890 (m), 786 (m), 725 (m), 698 (m), 688 (m), 674 (m).

^{1}**H NMR** (599 MHz, $CDCl_3$): δ [ppm] = 7.76 (dd, $^{3}J_{HH}$ = 2.0 Hz, $^{3}J_{HH}$ = 2.0 Hz, 1H, H-C2), 7.52 – 7.48 (m, 2H, H-C4, H-C6,), 7.28 (ddq, $^{3}J_{HH}$ = 8.0 Hz, $^{3}J_{HH}$ = 7.9 Hz, $^{5}J_{HF}$ = 0.3 Hz, 1H, H-C5), 5.55 (ddddq, $^{3}J_{HH}$ = 16.9 Hz, $^{3}J_{HH}$ = 10.1 Hz, $^{3}J_{HH}$ = 7.8 Hz, $^{3}J_{HH}$ = 6.7 Hz, $^{5}J_{HF}$ = 2.0 Hz, 1H, H-C10), 5.25 – 5.29 (m, 2H, H-C11), 2.92 (ddq, $^{2}J_{HH}$ = 14.3 Hz, $^{2}J_{HH}$ = 6.6 Hz, $^{4}J_{HF}$ = 0.9 Hz, 1H, H^{a}-C9), 2.84 (ddq, $^{2}J_{HH}$ = 14.3 Hz, $^{2}J_{HH}$ = 8.0 Hz, $^{4}J_{HF}$ = 0.9 Hz, 1H, H^{b}-C9), 2.62 (s, 1H, OH).

^{13}**C NMR** (151 MHz, $CDCl_3$): δ [ppm] = 134.9 (C3), 131.9 (C6), 130.03 (C5), 130.00 (C10), 129.9 (q, $^{4}J_{CF}$ = 1.3 Hz, C2), 125.3 (q, $^{4}J_{CF}$ = 1.4 Hz, C4), 125.2 (q, $^{1}J_{CF}$ = 285.7 Hz, C8), 122.8 (C1), 122.7 (C11), 75.6 (q, $^{2}J_{CF}$ = 28.6 Hz, C7), 40.5 (q, $^{3}J_{CF}$ = 1.3 Hz, C9).

^{19}**F NMR** (564 MHz, $CDCl_3$): δ [ppm] = -79.07 (s, 3F, F-C8).

^{19}**F{^{1}H} NMR** (564 MHz, $CDCl_3$): δ [ppm] = -79.07 (s, 3F, F-C8).

GC-EI-MS: (*m/z*) requires: [$(C_{11}H_{10}BrF_3O)^+$] = 293.9862, (*m/z*) found: [$(C_{11}H_{10}BrF_3O)^+$] = 293.9862.

1-Bromo-3-(1,1,1-trifluoropenta-2,4-dien-2-yl)benzene (1b)

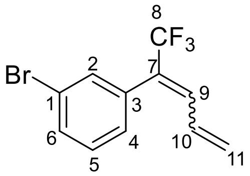

E/*Z* = 4:1

Compound **1b** was prepared according to **General Procedure C** using 2-(3-bromophenyl)-1,1,1-trifluoropent-4-en-2-ol (**1b-1**) (1.09 g, 3.75 mmol, 1.0 eq.). The crude residue was purified by column chromatography (*n*-pentane) to yield the title compound as a colorless oil (0.72 g, 2.60 mmol, 69%, *E*:*Z* = 4:1).

R*f* = 0.78 (*n*-pentane).

FT-IR ($\tilde{\nu}$ = cm^{-1})**:** 3093 (w), 1870 (w), 1651 (w), 1604 (w), 1559 (m), 1475 (w), 1425 (w), 1363 (m), 1278 (s), 1200 (m), 1174 (s), 1147 (m), 1112 (s), 1074 (m), 994 (m), 962 (m), 931 (m), 886 (m), 796 (m), 774 (m), 716 (s), 697 (m), 682 (m), 654 (m).

^{1}**H NMR** (599 MHz, $CDCl_3$):

E-diene: δ [ppm] = 7.54 (ddd, $^{3}J_{HH}$ = 8.0 Hz, $^{4}J_{HH}$ = 2.0 Hz, $^{4}J_{HH}$ = 1.1 Hz, 1H, H-C6), 7.45 (dd, $^{4}J_{HH}$ = 2.0 Hz, $^{4}J_{HH}$ = 1.9 Hz, 1H, H-C2), 7.29 (dd, $^{3}J_{HH}$ = 8.0 Hz, $^{3}J_{HH}$ = 7.7 Hz, 1H,

H-C5), 7.23 (d, $^{3}J_{HH}$ = 7.7 Hz, 1H, H-C4), 6.88 (dqd, $^{3}J_{HH}$ = 11.1 Hz, $^{4}J_{HF}$ = 1.6 Hz, $^{4}J_{HH}$ = 0.8 Hz, 1H, H-C9), 6.29 – 6.19 (m, 1H, H-C10), 5.62 (ddq, $^{3}J_{HH}$ = 16.9 Hz, $^{2}J_{HH}$ = 1.8 Hz, $^{6}J_{HF}$ = 1.0 Hz, 1H, H^{a}-C11), 5.44 (dddq, $^{3}J_{HH}$ = 10.1 Hz, $^{2}J_{HH}$ = 1.5 Hz, $^{4}J_{HH}$ = 0.8 Hz, $^{6}J_{HF}$ = 0.8 Hz, 1H, H^{b}-C11).

Z-diene: δ [ppm] = 7.51 (ddt, $^{4}J_{HH}$ = 2.0 Hz, $^{4}J_{HH}$ = 1.3 Hz, $^{4}J_{HF}$ = 0.6 Hz, 1H, H-C2), 7.49 (ddd, $^{3}J_{HH}$ = 7.8 Hz, $^{4}J_{HH}$ = 2.0 Hz, $^{4}J_{HH}$ = 1.1 Hz, 1H, H-C6), 7.30 (d, $^{3}J_{HH}$ = 7.8 Hz, 1H, H-C4), 7.24 (dd, $^{3}J_{HH}$ = 7.8 Hz, $^{3}J_{HH}$ = 7.8 Hz, 1H, H-C5), 6.93 (dddq, $^{3}J_{HH}$ = 13.8 Hz, $^{3}J_{HH}$ = 10.1 Hz, $^{3}J_{HH}$ = 4.5 Hz, $^{5}J_{HF}$ = 1.3 Hz, 1H, H-C10), 6.52 (dq, $^{3}J_{HH}$ = 11.5 Hz, $^{4}J_{HF}$ = 0.8 Hz, 1H, H-C9), 5.59 (d, 1H, $^{3}J_{HH}$ = 16.6 Hz, 1H, H^{a}-C11), 5.57 (d, 1H, $^{3}J_{HH}$ = 10.0 Hz, 1H, H^{b}-C11).

^{13}C NMR (151 MHz, $CDCl_3$):

E-diene: δ [ppm] = 134.6 (q, $^{3}J_{CF}$ = 5.8 Hz, C9), 134.2 (q, $^{3}J_{CF}$ = 0.7 Hz, C3), 132.9 (C2), 132.0 (C6), 131.4 (C10), 130.2 (C5), 129.6 (q, $^{4}J_{CF}$ = 30.4 Hz, C7), 128.8 (C4), 126.0 (q, $^{5}J_{CF}$ = 1.4 Hz, C11), 123.6 (q, $^{1}J_{CF}$ = 272.6 Hz, C8), 123.3 (C1).

^{19}F NMR (564 MHz, $CDCl_3$): *E*-diene: δ [ppm] = -65.19 (d, $^{4}J_{HF}$ = 1.7 Hz, 3F, F-C8); *Z*-diene: δ [ppm] = -56.65 (d, $^{4}J_{HF}$ = 2.1 Hz, 3F, F-C8)

^{19}F{^{1}H} NMR (564 MHz, $CDCl_3$): *E*-diene: δ [ppm] = -65.19 (s, 3F, F-C8); *Z*-diene: δ [ppm] = -56.65 (s, 3F, F-C8)

GC-EI-MS: (*m/z*) requires: [$(C_{11}H_8BrF_3)^+$] = 275.9756, (*m/z*) found: [$(C_{11}H_8BrF_3)^+$] = 275.9756.

2-(4-Chlorophenyl)-1,1,1-trifluoropent-4-en-2-ol (1c-1)

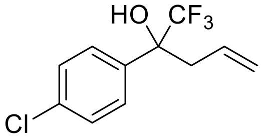

Compound **1c-1** was prepared according to **General Procedure B** (**Method 1**) using 1-(4-chlorophenyl)-2,2,2-trifluoroethan-1-one (1.00 g, 4.79 mmol, 1.0 eq.). The crude residue was purified by column chromatography (*n*-pentane:DCM 10:1) to yield the title compound as a colorless oil (0.99 g, 3.95 mmol, 82%).

R_f = 0.66 (*n*-pentane:EtOAc 6:1).

^{1}H NMR (400 MHz, $CDCl_3$): δ[ppm] = 7.59 – 7.46 (m, 2H), 7.44 – 7.32 (m, 2H), 5.67 – 5.42 (m, 1H), 5.31 – 5.20 (m, 2H), 2.94 (dd, J = 14.4, 6.6 Hz, 1H), 2.83 (dd, J = 14.3, 8.0 Hz, 1H), 2.60 (s, 1H).

^{19}F NMR (376 MHz, $CDCl_3$): δ[ppm] = -79.31 (s, 3F).

^{19}F{^{1}H} NMR (376 MHz, $CDCl_3$): δ[ppm] = -79.31 (s, 3F).

ESI-MS: (*m/z*) requires: [$(C_{11}H_{10}ClF_3O\text{-}H)^-$] = 249.0300, (*m/z*) found: [$(C_{11}H_{10}ClF_3O\text{-}H)^-$] = 249.0298.

The analytical data were in good agreement with the literature.[10]

1-Chloro-4-(1,1,1-trifluoropenta-2,4-dien-2-yl)benzene (1c)

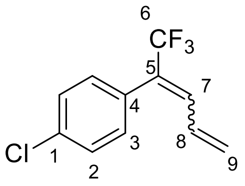

E/*Z* = 3.2:1

Compound **1c** was prepared according to **General Procedure C** using 2-(4-chlorophenyl)-1,1,1-trifluoropent-4-en-2-ol **(1c-1)** (0.95 g, 3.79 mmol, 1.0 eq.). The crude residue was purified by column chromatography (*n*-pentane) to yield the title compound as a colorless oil (0.47 g, 2.02 mmol, 53%, *E*:*Z* = 3.2:1).

R_f = 0.73 (*n*-pentane).

FT-IR ($\tilde{\nu}$ = cm^{-1})**:** 3094 (w), 1649 (w), 1593 (w), 1494 (m), 1425 (w), 1401 (w), 1366 (m), 1276 (s), 1200 (m), 1172 (m), 1144 (m), 1115 (s), 1089 (s), 1017 (m), 993 (m), 956 (m), 929 (s), 917 (m), 829 (s), 768 (m), 732 (m), 703 (w).

^{1}H NMR (599 MHz, $CDCl_3$):

E-diene: δ[ppm] = 7.58 – 7.53 (m, 2H, H-C3), 7.20 – 7.08 (m, 2H, H-C2), 6.87 (dqd, $^3J_{HH}$ = 11.0 Hz, $^4J_{HF}$ = 1.6 Hz, $^4J_{HH}$ = 0.8 Hz, 1H, H-C7), 6.33 – 6.14 (m, 1H, H-C8), 5.61 (ddq, $^3J_{HH}$ = 16.9 Hz, $^2J_{HH}$ = 1.7 Hz, $^6J_{HF}$ = 1.6 Hz, 1H, H^a-C9), 5.42 (dddq, $^3J_{HH}$ = 10.2 Hz, $^2J_{HH}$ = 1.6 Hz, $^4J_{HH}$ = 0.8 Hz, $^6J_{HF}$ = 0.8 Hz, 1H, H^b-C9).

Z-diene: δ [ppm] = 7.52 – 7.45 (m, 2H, H-C3), 7.23 (dq, $^3J_{HH}$ = 7.7 Hz, $^5J_{HF}$ = 0.9 Hz, 2H, H-C2), 7.01 – 6.88 (m, 1H, H-C8), 6.51 (dq, $^3J_{HH}$ = 11.5 Hz, $^4J_{HF}$ = 0.8 Hz, 1H, H-C7), 5.59 – 5.56 (d, 1H, $^3J_{HH}$ = 16.6 Hz, 1H, H^a-C9), 5.55 (d, 1H, $^3J_{HH}$ = 10.0 Hz, 1H, H^b-C9).

^{13}C NMR (151 MHz, $CDCl_3$):

E-diene: δ [ppm] = 135.1 (C1), 134.3 (q, $^3J_{CF}$ = 5.8 Hz, C7), 131.5 (C8), 131.4 (C2), 130.6 (q, $^3J_{CF}$ = 0.6 Hz, C4), 130.0 (q, $^2J_{CF}$ = 30.2 Hz, C5), 128.9 (C3), 125.6 (q, $^5J_{CF}$ = 1.4 Hz, C9), 123.6 (q, $^1J_{CF}$ = 272.6 Hz, C6).

^{19}F NMR (564 MHz, $CDCl_3$): *E*-diene: δ [ppm] = -65.29 (s, 3F, F-C6); *Z*-diene: δ [ppm] = -56.71 (d, $^4J_{HF}$ = 2.1 Hz, 3F, F-C6)

^{19}F{^{1}H} NMR (564 MHz, $CDCl_3$): *E*-diene: δ [ppm] = -65.29 (s, 3F, F-C6); *Z*-diene: δ [ppm] = -56.71 (s, 3F, F-C6)

GC-EI-MS: (*m/z*) requires: [$(C_{11}H_8ClF_3)^+$] = 232.0261, (*m/z*) found: [$(C_{11}H_8ClF_3)^+$] = 232.0261.

2-(3-Chlorophenyl)-1,1,1-trifluoropent-4-en-2-ol (1d-1)

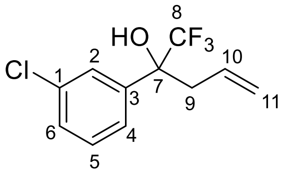

Compound **1d-1** was prepared according to **General Procedure A and B** (**Method 1**) using 3-chlorobenzaldehyde (1.40 g, 9.96 mmol, 1.0 eq.). The crude residue was purified by column chromatography (*n*-pentane:EtOAc 50:1) to yield the title compound as a colorless oil (1.61 g, 6.42 mmol, 64%).

R_f = 0.60 (*n*-pentane:EtOAc 10:1).

FT-IR ($\tilde{\nu}$ = cm^{-1})**:** 2941 (w), 1600 (w), 1496 (m), 1456 (w), 1406 (w), 1385 (w), 1271 (m), 1221 (w), 1177 (s), 1162 (s), 1110 (m), 1095 (m), 1014 (m), 954 (s), 942 (m), 915 (m), 828 (m), 796 (w), 772 (w), 737 (m), 730 (m), 692 (m).

^{1}H NMR (599 MHz, $CDCl_3$): δ [ppm] = 7.61 (dd, $^3J_{HH}$ = 8.1 Hz, $^3J_{HH}$ = 2.1 Hz, 1H, H-C2), 7.45 (ddq, $^3J_{HH}$ = 6.7 Hz, $^4J_{HH}$ = 1.9 Hz, $^5J_{HF}$ = 0.9 Hz, 1H, H-C4), 7.39 – 7.30 (m, 2H, H-C5, H-C6), 5.55 (ddddq, $^3J_{HH}$ = 17.0 Hz, $^3J_{HH}$ = 10.1 Hz, $^3J_{HH}$ = 7.8 Hz, $^3J_{HH}$ = 6.7 Hz, $^5J_{HF}$ = 0.9

Hz, 1H, H-C10), 5.36 – 5.19 (m, 2H, H-C11), 2.93 (ddq, $^{2}J_{HH}$ = 14.4 Hz, $^{3}J_{HH}$ = 6.7 Hz, $^{4}J_{HF}$ = 1.0 Hz, 1H, H^{a}-C9), 2.89 – 2.79 (m, 1H, H^{b}-C9), 2.64 (s, 1H, OH).

^{13}C NMR (151 MHz, $CDCl_3$): δ [ppm] = 139.1 (C3), 134.7 (C1), 130.0 (C10), 129.8 (C6), 129.0 (C5), 127.1 (q, $^{4}J_{CF}$ = 1.4 Hz, C2), 125.2 (q, $^{1}J_{CF}$ = 285.3 Hz, C8), 124.8 (q, $^{4}J_{CF}$ = 1.4 Hz, C4), 122.7 (C11), 75.6 (q, $^{2}J_{CF}$ = 28.6 Hz, C7), 40.5 (q, $^{3}J_{CF}$ = 1.3 Hz, C9).

^{19}F NMR (564 MHz, $CDCl_3$): δ [ppm] = -79.09 (s, 3F, F-C8).

^{19}F{^{1}H} NMR (564 MHz, $CDCl_3$): δ [ppm] = -79.09 (s, 3F, F-C8).

GC-EI-MS: (*m/z*) requires: [$(C_{11}H_{10}ClF_3O)^{+}$] = 250.0367, (*m/z*) found: [$(C_{11}H_{10}ClF_3O)^{+}$] = 250.0366.

1-Chloro-3-(1,1,1-trifluoropenta-2,4-dien-2-yl)benzene (1d)

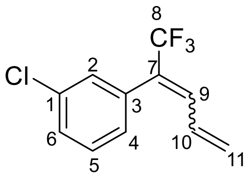

E/*Z* = 4.4:1

Compound **1d** was prepared according to **General Procedure C** using 2-(3-chlorophenyl)-1,1,1-trifluoropent-4-en-2-ol (**1d-1**) (1.49 g, 5.95 mmol, 1.0 eq.). The crude residue was purified by column chromatography (*n*-pentane) to yield the title compound as a colorless oil (0.82 g, 3.52 mmol, 59%, *E*:*Z* = 4.4:1).

R$_f$ = 0.78 (*n*-pentane).

FT-IR ($\tilde{\nu}$ = cm^{-1})**:** 3072 (w), 1652 (w), 1604 (w), 1565 (m), 1477 (w), 1425 (w), 1364 (m), 1279 (s), 1200 (m), 1176 (s), 1147 (m), 1112 (s), 1081 (m), 993 (m), 965 (m), 931 (m), 886 (m), 808 (m), 785 (m), 717 (s), 706 (m), 698 (m), 658 (m).

^{1}H NMR (500 MHz, $CDCl_3$):

E-diene: δ [ppm] = 7.39 (ddd, $^{3}J_{HH}$ = 8.0 Hz, $^{4}J_{HH}$ = 2.1 Hz, $^{4}J_{HH}$ = 1.3 Hz, 1H, H-C6), 7.35 (dd, $^{3}J_{HH}$ = 8.1 Hz, $^{3}J_{HH}$ = 7.4 Hz, 1H, H-C5), 7.31 – 7.29 (m, 1H, H-C2), 7.18 (ddq, $^{3}J_{HH}$ = 7.4 Hz, $^{4}J_{HH}$ = 1.5 Hz, $^{5}J_{HF}$ = 0.8 Hz, 1H, H-C4), 6.88 (dqd, $^{3}J_{HH}$ = 11.0 Hz, $^{4}J_{HF}$ = 1.6 Hz, $^{4}J_{HH}$ = 0.8 Hz, 1H, H-C9), 6.31 – 6.18 (m, 1H, H-C10), 5.62 (ddq, $^{3}J_{HH}$ = 17.0 Hz, $^{2}J_{HH}$ = 1.9 Hz, $^{6}J_{HF}$ = 1.0 Hz, 1H, H^{a}-C11), 5.44 (dddq, $^{3}J_{HH}$ = 10.1 Hz, $^{2}J_{HH}$ = 1.5 Hz, $^{4}J_{HH}$ = 0.7 Hz, $^{6}J_{HF}$ = 0.7 Hz, 1H, H^{b}-C11).

Z-diene: δ [ppm] = 7.37 – 7.33 (m, 2H, H-C2, H-C6), 7.32 – 7.28 (m, 1H, H-C5), 7.27 – 7.23 (m, 1H, H-C4), 6.99 – 6.89 (m, 1H, H-C10), 6.52 (dq, $^{3}J_{HH}$ = 11.5 Hz, $^{4}J_{HF}$ = 0.8 Hz, 1H, H-C9), 5.60 – 5.54 (m, 2H, H-C11).

^{13}C NMR (126 MHz, $CDCl_3$):

E-diene: δ [ppm] = 134.59 (C1), 134.56 (q, $^{3}J_{CF}$ = 6.0 Hz, C9), 133.7 (q, $^{3}J_{CF}$ = 0.7 Hz, C3), 131.4 (C10), 130.1 (C2), 129.9 (C5), 129.7 (q, $^{4}J_{CF}$ = 30.1 Hz, C7), 129.1 (C6), 128.3 (C4), 125.9 (q, $^{5}J_{CF}$ = 1.7 Hz, C11), 123.6 (q, $^{1}J_{CF}$ = 272.5 Hz, C8).

^{19}F NMR (470 MHz, $CDCl_3$): *E*-diene: δ [ppm] = -64.89 – -65.58 (m, 3F, F-C8); *Z*-diene: δ [ppm] = -56.67 (d, $^{4}J_{HF}$ = 2.2 Hz, 3F, F-C8).

^{19}F{^{1}H} NMR (470 MHz, $CDCl_3$): *E*-diene: δ [ppm] = -65.20 (s, 3F, F-C8); *Z*-diene: δ [ppm] = -56.67 (s, 3F, F-C8)

GC-EI-MS: (*m/z*) requires: [$(C_{11}H_8ClF_3)^+$] = 232.0261, (*m/z*) found: [$(C_{11}H_8ClF_3)^+$] = 232.0261.

2-(3,5-Dichlorophenyl)-1,1,1-trifluoropent-4-en-2-ol (1e-1)

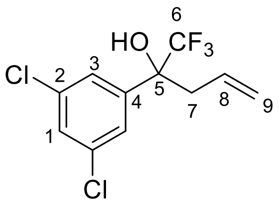

Compound **1e-1** was prepared according to **General Procedure B** (**Method 1**) using 1-(3,5-dichlorophenyl)-2,2,2-trifluoroethan-1-one (1.15 g, 4.73 mmol, 1.0 eq.). The crude residue was purified by column chromatography (*n*-pentane:EtOAc 40:1) to yield the title compound as a colorless oil (1.20 g, 4.21 mmol, 89%).

R_f = 0.54 (*n*-pentane:EtOAc 10:1).

FT-IR ($\tilde{\nu}$ = cm^{-1})**:** 3546 (w), 3087 (w), 2985 (w), 1713 (w), 1643 (w), 1591 (w), 1568 (m), 1421 (m), 1388 (w), 1272 (m), 1231 (w), 1163 (s), 1143 (s), 1109 (m), 1023 (m), 995 (m), 968 (w), 927 (m), 897 (w), 861 (m), 801 (s), 729 (m), 696 (m), 680 (m), 667 (m).

^{1}H NMR (500 MHz, $CDCl_3$): δ [ppm] = 7.47 (dq, $^{4}J_{HH}$ = 1.8 Hz, $^{5}J_{HF}$ = 0.7 Hz, 2H, H-C3), 7.37 (t, $^{4}J_{HH}$ = 1.9 Hz, 1H, H-C1), 5.66 – 5.45 (m, 1H, H-C8), 5.37 – 5.22 (m, 2H, H-C9), 2.97 – 2.78 (m, 2H, H-C7), 2.68 (s, 1H, OH).

^{13}**C NMR** (126 MHz, $CDCl_3$): δ [ppm] = 140.5 (C4), 135.4 (C3), 129.6 (C8), 129.0 (C1), 125.4 (q, $^4J_{CF}$ = 1.5 Hz, C2), 124.9 (q, $^1J_{CF}$ = 285.6 Hz, C6), 123.2 (C9), 75.5 (q, $^2J_{CF}$ = 28.7 Hz, C5), 40.5 (q, $^3J_{CF}$ = 1.2 Hz, C7).

^{19}**F NMR** (470 MHz, $CDCl_3$): δ [ppm] = -78.95 (s, 3F, F-C6).

^{19}**F{^{1}H} NMR** (470 MHz, $CDCl_3$): δ [ppm] = -78.95 (s, 3F, F-C6).

GC-EI-MS: (*m/z*) requires: $[(C_{11}H_9Cl_2F_3O)^+]$ = 283.9977, (*m/z*) found: $[(C_{11}H_9Cl_2F_3O)^+]$ = 283.9973.

1-Chloro-3-(1,1,1-trifluoropenta-2,4-dien-2-yl)benzene (1e)

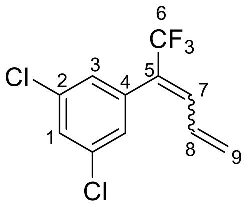

E/*Z* = 4.7:1

Compound **1e** was prepared according to **General Procedure C** using 2-(3,5-dichlorophenyl)-1,1,1-trifluoropent-4-en-2-ol (**1e-1**) (1.15 g, 4.03 mmol, 1.0 eq.). The crude residue was purified by column chromatography (*n*-pentane) to yield the title compound as a colorless oil (0.61 g, 2.28 mmol, 57%, *E*:*Z* = 4.7:1).

R*f* = 0.76 (*n*-pentane).

FT-IR ($\tilde{\nu}$ = cm^{-1})**:** 3080 (w), 1651 (w), 1586 (m), 1560 (m), 1445 (w), 1415 (w), 1389 (w), 1360 (m), 1279 (s), 1237 (w), 1202 (m), 1182 (s), 1151 (m), 1113 (s), 992 (m), 948 (m), 934 (m), 885 (w), 863 (m), 836 (w), 819 (m), 804 (s), 721 (s), 686 (m), 669 (m), 659 (m).

^{1}**H NMR** (599 MHz, $CDCl_3$):

E-diene: δ [ppm] = 7.42 (t, $^4J_{HH}$ = 1.9 Hz, 1H, H-C1), 7.23 – 7.16 (m, 2H, H-C3), 6.90 (dqd, $^3J_{HH}$ = 11.0 Hz, $^4J_{HF}$ = 2.1 Hz, $^4J_{HH}$ = 0.9 Hz, 1H, H-C7), 6.23 (ddq, $^3J_{HH}$ = 16.9 Hz, $^3J_{HH}$ = 10.2 Hz, $^5J_{HF}$ = 1.1 Hz, 1H, H-C8), 5.66 (dq, $^3J_{HH}$ = 16.9 Hz, $^6J_{HF}$ = 1.1 Hz, 1H, H^a-C9), 5.49 (dddq, $^3J_{HH}$ = 10.2 Hz, $^2J_{HH}$ = 1.5 Hz, $^4J_{HH}$ = 0.8 Hz, $^6J_{HF}$ = 0.8 Hz, 1H, H^b-C9).

Z-diene: δ [ppm] = 7.36 (t, $^4J_{HH}$ = 1.9 Hz, 1H, H-C1), 7.25 (dq, $^4J_{HH}$ = 1.9 Hz, $^5J_{HF}$ = 0.8 Hz, 2H, H-C3), 6.97 – 6.91 (m, 1H, H-C8), 6.54 (dq, $^3J_{HH}$ = 11.6 Hz, $^4J_{HF}$ = 0.8 Hz, 1H, H-C7), 5.64 – 5.59 (m, 2H, H-C9).

^{13}**C NMR** (151 MHz, $CDCl_3$):

E-diene: δ [ppm] = 135.4 (C2), 135.2 (q, $^3J_{CF}$ = 5.8 Hz, C7), 134.9 (q, $^3J_{CF}$ = 0.9 Hz, C4), 130.9 (C8), 129.2 (C1), 128.5 (C3), 128.5 (q, $^4J_{CF}$ = 30.7 Hz, C5), 126.8 (q, $^5J_{CF}$ = 1.2 Hz, C9), 123.3 (q, $^1J_{CF}$ = 272.4 Hz, C6).

^{19}F NMR (564 MHz, $CDCl_3$): *E*-diene: δ [ppm] = -65.13 (s, 3F, F-C6); *Z*-diene: δ [ppm] = -56.74 (d, $^4J_{HF}$ = 2.1 Hz, 3F, F-C6).

^{19}F{^{1}H} NMR (564 MHz, $CDCl_3$): *E*-diene: δ [ppm] = -65.13 (s, 3F, F-C6); *Z*-diene: δ [ppm] = -56.74 (s, 3F, F-C6)

GC-EI-MS: (*m/z*) requires: [$(C_{11}H_7Cl_2F_3)^+$] = 265.9871, (*m/z*) found: [$(C_{11}H_7Cl_2F_3)^+$] = 265.9872.

1,1,1-Trifluoro-2-(4-fluorophenyl)pent-4-en-2-ol (1f-1)

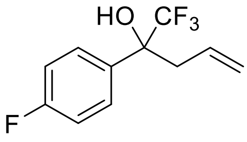

Compound **1f-1** was prepared according to **General Procedure B** (**Method 1**) using 2,2,2-trifluoro-1-(4-fluorophenyl)ethan-1-one (1.21 g, 6.30 mmol, 1.0 eq.). The crude residue was purified by column chromatography (*n*-pentane: EtOAc 50:1) to yield the title compound as a colorless oil (1.30 g, 5.55 mmol, 88%).

R_f = 0.68 (*n*-pentane:EtOAc 6:1).

^{1}H NMR (400 MHz, $CDCl_3$): δ [ppm] = 7.55 (dddq, *J* = 8.4, 5.2, 1.4, 0.7 Hz, 2H), 7.16 – 7.03 (m, 2H), 5.56 (ddddq, *J* = 16.9, 10.0, 7.7, 6.7, 0.9 Hz, 1H), 5.42 – 5.18 (m, 2H), 3.02 – 2.90 (m, 1H), 2.88 – 2.77 (m, 1H), 2.61 (s, 1H).

^{19}F NMR (377 MHz, $CDCl_3$): δ [ppm] = -113.72 (s, 1F), -79.45 (tt, *J* = 8.4, 5.4 Hz, 3F).

^{19}F{^{1}H} NMR (377 MHz, $CDCl_3$): δ [ppm] = -113.72 (s, $^3J_{CF}$ = 5.8 Hz,1F), -79.45 (s, 3F).

ESI-MS: (*m/z*) requires: [$(C_{11}H_{10}F_4O\text{-}H)^-$] = 233.0595, (*m/z*) found: [$C_{11}H_{10}F_4O\text{-}H)^-$] = 233.0594.

The analytical data were in good agreement with the literature.[10]

1-Fluoro-4-(1,1,1-trifluoropenta-2,4-dien-2-yl)benzene (1f)

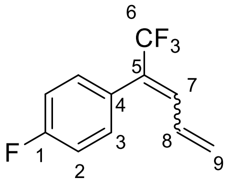

E/*Z* = 2.9:1

Compound **1f** was prepared according to **General Procedure C** using 1,1,1-trifluoro-2-(4-fluorophenyl)pent-4-en-2-ol **(1f-1)** (1.25 g, 5.34 mmol, 1.0 eq.). The crude residue was purified by column chromatography (*n*-pentane) to yield the title compound as a colorless oil (0.56 g, 2.59 mmol, 49%, *E*:*Z* = 2.9:1).

$\mathbf{R}_f$ = 0.73 (*n*-pentane).

FT-IR ($\tilde{\nu}$ = cm^{-1})**:** 1653 (w), 1602 (w), 1511 (m), 1425 (w), 1367 (m), 1278 (m), 1237 (m), 1200 (m), 1172 (s), 1143 (m), 1112 (s), 1017 (w), 994 (m), 957 (m), 948 (m), 928 (m), 836 (s), 817 (m), 737 (m), 725 (w), 696 (w), 667 (m).

^{1}H NMR (599 MHz, $CDCl_3$):

E-diene: δ [ppm] = 7.29 – 7.23 (m, 2H, H-C3), 7.12 – 7.07 (m, 2H, H-C2), 6.86 (dqd, $^3J_{HH}$ = 11.0 Hz, $^4J_{HF}$ = 1.7 Hz, $^4J_{HH}$ = 0.9 Hz, 1H, H-C7), 6.29 – 6.17 (m, 1H, H-C8), 5.59 (ddq, $^3J_{HH}$ = 17.0 Hz, $^2J_{HH}$ = 1.8 Hz, $^6J_{HF}$ = 1.0 Hz, 1H, H^a-C9), 5.40 (dddq, $^3J_{HH}$ = 10.1 Hz, $^2J_{HH}$ = 1.5 Hz, $^4J_{HH}$ = 0.8 Hz, $^6J_{HF}$ = 0.8 Hz, 1H, H^b-C9).

Z-diene: δ [ppm] = 7.36 – 7.29 (m, 2H, H-C3), 7.07 – 7.03 (m, 2H, H-C2), 6.98 – 6.88 (m, 1H, H-C8), 6.48 (dq, $^3J_{HH}$ = 11.4 Hz, $^4J_{HF}$ = 0.8 Hz, 1H, H-C7), 5.57 – 5.50 (m, 2H, H-C9).

^{13}C NMR (151 MHz, $CDCl_3$):

E-diene: δ [ppm] = 163.1 (d, $^1J_{CF}$ = 248.5 Hz, C1), 134.2 (q, $^3J_{CF}$ = 5.9 Hz, C7), 132.0 (d, $^3J_{CF}$ = 8.2 Hz, C3), 131.6 (C8), 130.1 (q, $^2J_{CF}$ = 30.1 Hz, C5), 128.1 (d, $^4J_{CF}$ = 3.5 Hz, C4), 125.4 (q, $^5J_{CF}$ = 1.4 Hz, C9), 123.7 (q, $^1J_{CF}$ = 272.6 Hz, C6), 115.7 (d, $^2J_{CF}$ = 21.6 Hz, C2).

^{19}F NMR (564 MHz, $CDCl_3$): *E*-diene: δ [ppm] = -65.51 (s, 3F, F-C6), -112.66 (tt, $^3J_{HF}$ = 8.6 Hz, $^4J_{HF}$ = 5.4 Hz, 1F, F-C1); *Z*-diene: δ [ppm] = -56.87 (d, $^4J_{HF}$ = 2.2 Hz, 3F, F-C6), -113.32 (tt, $^3J_{HF}$ = 8.6 Hz, $^4J_{HF}$ = 5.3 Hz, 1F, F-C1).

^{19}F{^{1}H} NMR (376 MHz, $CDCl_3$): *E*-diene: δ [ppm] = -65.47 (s, 3F, F-C6), -112.61 (s, 1F, F-C1); *Z*-diene: δ [ppm] = -56.81 (s, 3F, F-C6), -113.28 (s, 1F, F-C1).

GC-EI-MS: (*m/z*) requires: [$(C_{11}H_8F_4)^+$] = 216.0557, (*m/z*) found: [$(C_{11}H_8F_4)^+$] = 216.0557.

1,1,1-Trifluoro-2-(4-fluoro-2-methylphenyl)pent-4-en-2-ol (1g-1)

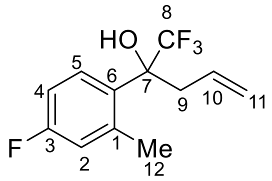

Compound **1g-1** was prepared according to **General Procedure A and B** (**Method 1**) using 4-fluoro-2-methylbenzaldehyde (1.37 g, 9.93 mmol, 1.0 eq.). The crude residue was purified by column chromatography (*n*-pentane:EtOAc 100:1) to yield the title compound as a colorless oil (1.18 g, 4.75 mmol, 48%).

$\mathbf{R_f}$ = 0.61 (*n*-pentane:EtOAc 10:1).

FT-IR ($\tilde{\nu}$ = cm^{-1})**:** 3536 (w), 3087 (w), 2985 (w), 1720 (w), 1612 (w), 1589 (m), 1499 (m), 1456 (w), 1367 (w), 1266 (m), 1229 (m), 1154 (s), 1123 (m), 1037 (w), 1012 (m), 993 (m), 966 (m), 935 (m), 865 (m), 812 (m), 764 (w), 739 (m), 726 (m), 698 (w), 681 (m).

^{1}H NMR (500 MHz, $CDCl_3$): δ [ppm] = 7.44 (dd, $^{4}J_{HF}$ = 9.3 Hz, $^{3}J_{HH}$ = 5.9 Hz, 1H, H-C5), 6.99 – 6.82 (m, 2H, H-C2, H-C4), 5.66 (ddddq, $^{3}J_{HH}$ = 17.0 Hz, $^{3}J_{HH}$ = 10.2 Hz, $^{3}J_{HH}$ = 7.8 Hz, $^{3}J_{HH}$ = 6.8 Hz, $^{5}J_{HF}$ = 1.1 Hz, 1H, H-C10), 5.38 – 5.25 (m, 2H, H-C11), 3.16 (dd, $^{2}J_{HH}$ = 14.8 Hz, $^{2}J_{HH}$ = 6.7 Hz, 1H, H^{a}-C9), 2.85 (dd, $^{2}J_{HH}$ = 14.7 Hz, $^{2}J_{HH}$ = 7.8 Hz, 1H, H^{b}-C9), 2.59 (s, 1H, OH), 2.58 (s, 3H, H-C12).

^{13}C NMR (126 MHz, $CDCl_3$): δ [ppm] = 162.4 (d, $^{1}J_{CF}$ = 248.0 Hz, C3), 141.5 (d, $^{4}J_{CF}$ = 7.5 Hz, C6), 130.64 (C5), 130.58 (q, $^{5}J_{CF}$ = 1.5 Hz, C10), 130.2 (d, $^{3}J_{CF}$ = 3.3 Hz, C1), 125.8 (q, $^{1}J_{CF}$ = 285.7 Hz, C8), 122.6 (C11), 119.8 (d, $^{2}J_{CF}$ = 20.8 Hz, C2), 112.5 (d, $^{2}J_{CF}$ = 20.8 Hz, C4), 77. 6 (q, $^{2}J_{CF}$ = 28.8 Hz, C7), 40.8 (q, $^{3}J_{CF}$ = 1.5 Hz, C9), 23.35 – 23.06 (m, C12).

^{19}F NMR (470 MHz, $CDCl_3$): δ [ppm] = -78.85 (s, 3F, F-C6), -114.73 – -114.93 (m, 1F, F-C3).

^{19}F{^{1}H} NMR (470 MHz, $CDCl_3$): δ [ppm] = -78.85 (s, 3F, F-C6), -114.83 (s, 1F, F-C3).

ESI-MS: (*m/z*) requires: [$(C_{12}H_{12}F_4ONa)^+$] = 271.0717, (*m/z*) found: [$(C_{12}H_{12}F_4ONa)^+$] = 271.0710.

4-Fluoro-2-methyl-1-(1,1,1-trifluoropenta-2,4-dien-2-yl)benzene (1g)

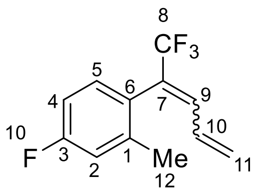

E/*Z* = 1.3:1

Compound **1g** was prepared according to **General Procedure C** using 1,1,1-trifluoro-2-(4-fluoro-2-methylphenyl)pent-4-en-2-ol (**1g-1**) (1.14 g, 4.59 mmol, 1.0 eq.). The crude residue was purified by column chromatography (*n*-pentane) to yield the title compound as a colorless oil (0.33 g, 1.43 mmol, 31%, *E*:*Z* = 1.3:1).

R$_f$ = 0.75 (*n*-pentane)

FT-IR ($\tilde{\nu}$ = cm^{-1})**:** 2932 (w), 1651 (w), 1601 (w), 1587 (m), 1498 (m), 1448 (w), 1426 (w), 1360 (m), 1272 (m), 1232 (m), 1196 (m), 1173 (m), 1152 (s), 1113 (s), 1038 (w), 994 (m), 963 (m), 932 (m), 894 (w), 865 (m), 918 (m), 745 (w), 733 (w), 698 (w), 669 (m).

^{1}H NMR (500 MHz, $CDCl_3$):

E-diene: δ [ppm] = 7.10 – 7.14 (m, 1H, H-C5), 7.01 – 6.96 (m, 1H, H-C4), 6.96 – 6.87 (m, 2H, H-C2, H-C9), 6.07 – 5.83 (m, 1H, H-C10), 5.76 – 5.55 (m, 1H, H^a-C11), 5.38 (ddq, $^3J_{HH}$ = 10.2 Hz, $^2J_{HH}$ = 1.6 Hz, $^6J_{HF}$ = 0.9 Hz, 1H, H^b-C11), 2.20 (s, 3H, H-C12).

Z-diene: δ [ppm] = 7.10 – 7.14 (m, 1H, H-C5), 6.96 – 6.87 (m, 2H, H-C4, H-C2), 6.32 (dq, $^3J_{HH}$ = 11.6 Hz, $^4J_{HF}$ = 0.8 Hz, 1H, H-C9), 5.55 – 5.45 (m, 2H, H-C11), 2.28 (s, 3H, H-C12).

^{13}C NMR (126 MHz, $CDCl_3$):

E-diene: δ [ppm] = 163.0 (d, $^2J_{CF}$ = 248.2 Hz, C3), 140.6 (d, $^3J_{CF}$ = 8.1 Hz, C1), 135.0 (q, $^3J_{CF}$ = 5.6 Hz, C9), 132.0 (d, $^3J_{CF}$ = 8.6 Hz, C5), 131.3 (C10), 129.4 (q, $^2J_{CF}$ = 30.5 Hz, C7), 127.2 (C6), 125.3 (q, $^5J_{CF}$ = 1.4 Hz, C11), 123.7 (q, $^1J_{CF}$ = 272.2 Hz, C8), 117.1 (d, $^2J_{CF}$ = 21.3 Hz, C2), 113.0 (d, $^2J_{CF}$ = 21.3 Hz, C4), 19.9 (C12).

^{19}F NMR (470 MHz, $CDCl_3$): *E*-diene: δ [ppm] = -66.31 ($^4J_{HH}$ = 1.6 Hz, 3F, F-C8), -113.53 (ddd, $^3J_{HF}$ = 9.7 Hz, $^3J_{HF}$ = 8.3 Hz, $^4J_{HF}$ = 5.9 Hz, 1F, F-C3); *Z*-diene: δ [ppm] = -57.63 ($^4J_{HH}$ = 2.3 Hz, 3F, F-C8), -113.94 (ddd, $^3J_{HF}$ = 9.6 Hz, $^3J_{HF}$ = 8.3 Hz, $^4J_{HF}$ = 5.8 Hz, 1F, F-C3).

^{19}F{^{1}H} NMR (376 MHz, $CDCl_3$): *E*-diene: δ [ppm] = -66.31 (s, 3F, F-C8), -113.53 (s, 1F, F-C3); *Z*-diene: δ [ppm] = -57.63 (s, 3F, F-C8), -113.94 (s, 1F, F-C3).

GC-EI-MS: (*m/z*) requires: [$(C_{12}H_{10}F_4)^+$] = 270.0713, (*m/z*) found: [$(C_{12}H_{10}F_4)^+$] = 270.0714.

1,1,1-Trifluoro-2-(4-(trifluoromethyl)phenyl)pent-4-en-2-ol (1h-1)

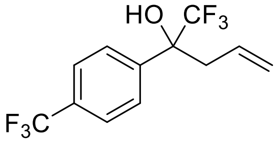

Compound **1h-1** was prepared according to **General Procedure A and B** (**Method 1**) using 4-(trifluoromethyl) benzaldehyde (1.78 g, 10.21 mmol, 1.0 eq.). The crude residue was purified by column chromatography (*n*-pentane: EtOAc 50:1) to yield the title compound as a colorless oil (1.39 g, 5.22 mmol, 51%).

R_f = 0.50 (*n*-pentane:EtOAc 10:1).

^{1}H NMR (400 MHz, $CDCl_3$): δ [ppm] = 7.75 – 7.69 (m, 2H), 7.69 – 7.63 (m, 2H), 5.54 (ddddd, *J* = 16.9, 10.0, 7.8, 6.8, 0.9 Hz, 1H), 5.41 – 5.19 (m, 2H), 3.01 – 2.92 (m, 1H), 2.92 – 2.83 (m, 1H), 2.69 (s, 1H).

^{19}F NMR (377 MHz, $CDCl_3$): δ [ppm] = -62.79 (s, 3F), -78.99 (s, 3F).

^{19}F{^{1}H} NMR (377 MHz, $CDCl_3$): δ [ppm] = -62.79 (s, 3F), -78.99 (s, 3F).

GC-EI-MS: (*m/z*) requires: [$(C_{12}H_{10}F_6O)^+$] = 284.1630, (*m/z*) found: [$(C_{11}H_{10}ClF_3O)^+$] = 284.1630.

The analytical data were in good agreement with the literature.[9]

1-(Trifluoromethyl)-4-(1,1,1-trifluoropenta-2,4-dien-2-yl)benzene (1h)

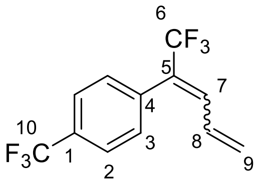

E/*Z* = 6.4:1

Compound **1h** was prepared according to **General Procedure C** using 1,1,1-trifluoro-2-(4-(trifluoromethyl)phenyl)pent-4-en-2-ol (**1h-1**) (1.28 g, 3.33 mmol, 1.0 eq.). The crude residue was purified by column chromatography (*n*-pentane) to yield the title compound as a colorless oil (0.48 g, 1.80 mmol, 54%, *E*:*Z* = 6.4:1).

R_f = 0.75 (*n*-pentane).

FT-IR ($\tilde{\nu}$ = cm^{-1})**:** 1652 (w), 1622 (w), 1427 (w), 1410 (w), 1368 (m), 1323 (s), 1276 (s), 1202 (m), 1170 (m), 1107 (s), 1067 (s), 1021 (m), 994 (m), 958 (m), 933 (s), 843 (s), 788 (w), 756 (w), 735 (m), 701 (m), 601 (m).

^{1}H NMR (500 MHz, $CDCl_3$):

E-diene: δ [ppm] = 7.76 – 7.65 (m, 2H, H-C2), 7.43 (d, $^3J_{HH}$ = 8.0 Hz, 2H, H-C3), 6.92 (ddq, $^3J_{HH}$ = 11.1 Hz, $^4J_{HF}$ = 1.6 Hz, $^4J_{HH}$ = 0.8 Hz, 1H, H-C7), 6.34 – 6.08 (m, 1H, H-C8), 5.73 – 5.61 (m, 1H, H^a-C9), 5.45 (dddq, $^3J_{HH}$ = 10.1 Hz, $^2J_{HH}$ = 1.5 Hz, $^4J_{HH}$ = 0.8 Hz, $^6J_{HF}$ = 0.8 Hz, 1H, H^b-C9).

Z-diene: δ [ppm] = 7.66 – 7.61 (m, 2H, H-C2), 7.44 (ddd, $^3J_{HH}$ = 8.0 Hz, $^5J_{HF}$ = 1.6 Hz, $^5J_{HF}$ = 0.8 Hz, 2H, H-C3), 7.01 – 6.92 (m, 1H, H-C8), 6.56 (dq, $^3J_{HH}$ = 11.6 Hz, $^4J_{HF}$ = 0.8 Hz, 1H, H-C7), 5.63 – 5.57 (m, 2H, H-C9).

^{13}C NMR (126 MHz, $CDCl_3$):

E-diene: δ [ppm] = 135.9 (C4), 134.8 (q, $^3J_{CF}$ = 5.8 Hz, C7), 131.2 (C8), 131.1 (q, $^2J_{CF}$ = 32.7 Hz, C1), 130.6 (C3), 130.0 (q, $^2J_{CF}$ = 30.3 Hz, C5), 126.3 (q, $^5J_{CF}$ = 1.3 Hz, C9), 125.6 (q, $^3J_{CF}$ = 3.8 Hz, C2), 124.0 (q, $^1J_{CF}$ = 272.4 Hz, C10), 123.5 (q, $^1J_{CF}$ = 272.4 Hz, C6).

^{19}F NMR (470 MHz, $CDCl_3$): *E*-diene: δ [ppm] = -62.89 (t, $^4J_{HF}$ = 0.8 Hz, 3F, F-C10), -65.07 – -65.14 (m, 3F, F-C6); *Z*-diene: δ [ppm] = -56.63 (dd, $^4J_{HF}$ = 2.1 Hz, $^5J_{HF}$ = 1.0 Hz, 3F, F-C6), -62.81 (t, $^4J_{HF}$ = 0.9 Hz, 3F, F-C10).

^{19}F{^{1}H} NMR (470 MHz, $CDCl_3$): *E*-diene: δ [ppm] = -62.89 (s, 3F, F-C10), -65.10 (s, 3F, F-C6); *Z*-diene: δ [ppm] = -56.63 (s, 3F, F-C6), -62.81 (s, 3F, F-C10).

GC-EI-MS: (*m/z*) requires: $[(C_{12}H_8F_6)^+]$ = 266.0525, (*m/z*) found: $[(C_{12}H_{11}F_3)^+]$ = 266.0526.

1,1,1-Trifluoro-2-(*p*-tolyl)pent-4-en-2-ol (1i-1)

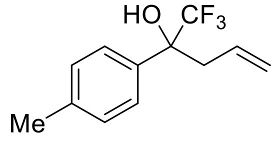

Compound **1i-1** was prepared according to **General Procedure B** (**Method 1**) using 2,2,2-trifluoro-1-(*p*-tolyl)ethan-1-one (1.21 g, 6.45 mmol, 1.0 eq.). The crude residue was purified by column chromatography (*n*-pentane:DCM 10:1) to yield the title compound as a colorless oil (1.29 g, 5.60 mmol, 87%).

R_f = 0.51 (*n*-pentane:EtOAc 10:1).

^{1}H NMR (400 MHz, $CDCl_3$): δ[ppm] = 7.45 (d, J = 8.0 Hz, 2H), 7.24 – 7.17 (m, 2H), 5.66 – 5.48 (m, 1H), 5.29 – 5.19 (m, 2H), 2.98 (dddq, J = 14.3, 6.5, 1.6, 0.8 Hz, 1H), 2.83 (ddq, J = 14.2, 8.0, 1.0 Hz, 1H), 2.57 (s, 1H), 2.37 (s, 3H),.

^{19}F NMR (377 MHz, $CDCl_3$): δ[ppm] = -79.40 (s, 3F).

^{19}F{^{1}H} NMR (377 MHz, $CDCl_3$): δ[ppm] = -79.40 (s, 3F).

ESI-MS: (m/z) requires: [$(C_{12}H_{13}F_3O\text{-}H)^-$] = 229.0846, ($m/z$) found: [$(C_{12}H_{13}F_3O\text{-}H)^-$] = 229.0845.

The analytical data were in good agreement with the literature.[9]

1-Methyl-4-(1,1,1-trifluoropenta-2,4-dien-2-yl)benzene (1i)

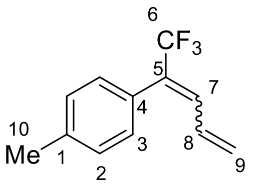

E/*Z* = 2.6:1

Compound **1i** was prepared according to **General Procedure C** using 1,1,1-trifluoro-2-(*p*-tolyl)pent-4-en-2-ol (**1i-1**) (1.23 g, 5.35 mmol, 1.0 eq.). The crude residue was purified by column chromatography (*n*-pentane) to yield the title compound as a colorless oil (0.73 g, 3.44 mmol, 64%, *E*:*Z* = 2.6:1).

R$_f$ = 0.78 (*n*-pentane).

FT-IR ($\tilde{\nu}$ = cm^{-1})**:** 2926 (w), 1650 (w), 1601 (w), 1515 (w), 1424 (w), 1366 (m), 1277 (m), 1217 (w), 1199 (m), 1689 (m), 1143 (m), 1109 (s), 1024 (w), 995 (m), 956 (m), 927 (s), 819 (m), 800 (w), 764 (w), 732 (m), 722 (m), 694 (w), 670 (m), 659 (m).

^{1}H NMR (599 MHz, $CDCl_3$):

E-diene: δ[ppm] = 7.24 – 7.22 (m, 2H, H-C3), 7.21 – 7.19 (m, 2H, H-C2), 6.85 (dqd, $^3J_{HH}$ = 11.0 Hz, $^4J_{HF}$ = 1.6 Hz, $^4J_{HH}$ = 0.9 Hz, 1H, H-C7), 6.31 (m, dddq, $^3J_{HH}$ = 15.8 Hz, $^3J_{HH}$ = 11.1 Hz, $^3J_{HH}$ = 10.0 Hz, $^4J_{HF}$ = 1.1 Hz, 1H, H-C8), 5.58 (ddq, $^3J_{HH}$ = 16.9 Hz, $^2J_{HH}$ = 1.8 Hz, $^6J_{HF}$ = 1.0 Hz, 1H, H^a-C9), 5.38 (dddq, $^3J_{HH}$ = 10.1 Hz, $^2J_{HH}$ = 1.6 Hz, $^4J_{HH}$ = 0.8 Hz, $^6J_{HF}$ = 0.8 Hz, 1H, H^b-C9), 2.40 (s, 3H, H-C10).

Z-diene: δ [ppm] = 7.29 – 7.26 (m, 2H, H-C3), 7.20 – 7.17 (m, 2H, H-C2), 7.02 – 6.91 (m, 1H, H-C8), 6.52 (dq, $^3J_{HH}$ = 11.5 Hz, $^4J_{HF}$ = 0.9 Hz, 1H, H-C7), 5.55 – 5.47 (m, 2H, H-C9), 2.38 (s, 3H, H-C10).

^{13}C NMR (151 MHz, $CDCl_3$):

E-diene: δ [ppm] = 138.7 (C1), 133.6 (q, $^3J_{CF}$ = 5.9 Hz, C7), 132.0 (C8), 131.2 (q, $^2J_{CF}$ = 29.7 Hz, C5), 129.9 (C2), 129.3 (C3), 129.2 (C4), 124.6 (q, $^5J_{CF}$ = 1.4 Hz, C9), 123.9 (q, $^1J_{CF}$ = 272.6 Hz, C6), 21.4 (C10).

^{19}F NMR (564 MHz, $CDCl_3$): *E*-diene: δ [ppm] = -65.27 (s, 3F, F-C6); *Z*-diene: δ [ppm] = -56.60 (d, $^4J_{HF}$ = 2.2 Hz, 3F, F-C6).

^{19}F{^{1}H} NMR (564 MHz, $CDCl_3$): *E*-diene: δ [ppm] = -65.27 (s, 3F, F-C6); *Z*-diene: δ [ppm] = -56.60 (s, 3F, F-C6).

GC-EI-MS: (*m/z*) requires: [$(C_{12}H_{11}F_3)^+$] = 212.0807, (*m/z*) found: [$(C_{12}H_{11}F_3)^+$] = 212.0807.

1,1,1-Trifluoro-2-phenylpent-4-en-2-ol (1j-1)

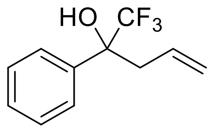

Compound **1j-1** was prepared according to **General Procedure B** (**Method 1**) using 2,2,2-trifluoro-1-phenylethan-1-one (1.24 g, 7.12 mmol, 1.0 eq.). The crude residue was purified by column chromatography (*n*-pentane: EtOAc 40:1) to yield the title compound as a colorless oil (1.50 g, 6.94 mmol, 97%).

R_f = 0.70 (*n*-pentane:EtOAc 6:1).

^{1}H NMR (400 MHz, $CDCl_3$): δ [ppm] = 7.64 – 7.50 (m, 2H), 7.47 – 7.31 (m, 3H), 5.77 – 5.49 (m, 1H), 5.40 – 5.18 (m, 2H), 2.99 (dd, *J* = 14.3, 6.6 Hz, 1H), 2.85 (dd, *J* = 14.2, 8.1 Hz, 1H), 2.61 (s, 1H).

^{19}F NMR (377 MHz, $CDCl_3$): δ [ppm] = -79.21 (s, 3F).

^{19}F{^{1}H} NMR (377 MHz, $CDCl_3$): δ [ppm] = -79.21 (s, 3F).

GC-EI-MS: (*m/z*) requires: [$(C_{11}H_{11}F_3O)^+$] = 216.0756, (*m/z*) found: [$(C_{11}H_{11}F_3O)^+$] = 216.0758.

The analytical data were in good agreement with the literature.[10]

(1,1,1-Trifluoropenta-2,4-dien-2-yl)benzene (1j)

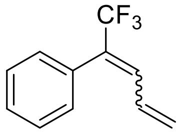

E/*Z* = 3:1

Compound **1j** was prepared according to **General Procedure C** using 1,1,1-trifluoro-2-phenylpent-4-en-2-ol (**1j-1**) (1.50 g, 6.94 mmol, 1.0 eq.). The crude residue was purified by column chromatography (*n*-pentane) to yield the title compound as a colorless oil (0.82 g, 4.14 mmol, 60%, *E*:*Z* = 3:1).

$\mathbf{R}_f$ = 0.75 (*n*-pentane).

^{1}H NMR (500 MHz, $CDCl_3$):

E-diene: δ [ppm] = 7.43 – 7.40 (m, 2H), 7.37 – 7.38 (m, 1H), 7.33 – 7.29 (m, 2H), 6.87 (ddq, *J* = 11.0, 1.7, 0.8 Hz, 1H), 6.39 – 6.17 (m, 1H), 5.65 – 5.56 (m, 1H), 5.39 (ddq, *J* = 10.1, 1.6, 0.8 Hz, 1H).

Z-diene: δ [ppm] = 7.45 – 7.39 (m, 3H), 7.38 – 7.36 (m, 2H), 7.04 – 6.90 (m, 1H), 6.54 (dq, *J* = 11.5, 0.8 Hz, 1H), 5.57 – 5.52 (m, 2H).

^{19}F NMR (470 MHz, $CDCl_3$): *E*-diene: -63.56 – -67.58 (m, 3F); *Z*-diene: δ [ppm] = -56.56 (d, *J* = 2.1 Hz, 3F).

^{19}F{^{1}H} NMR (470 MHz, $CDCl_3$): *E*-diene: -65.23 (s, 3F); *Z*-diene: δ [ppm] = -56.56 (s, 3F).

GC-EI-MS: (*m/z*) requires: $[(C_{11}H_9F_3)^+]$ = 198.0651, (*m/z*) found: $[(C_{11}H_9F_3)^+]$ = 198.0650.

The analytical data were in good agreement with the literature.[11]

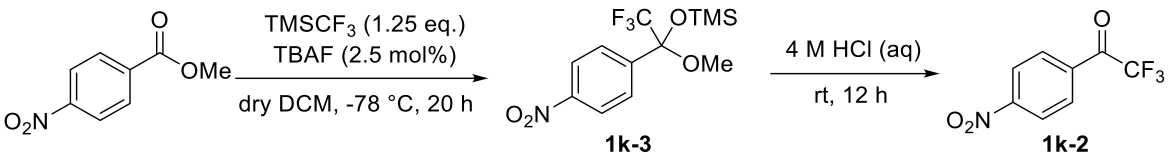

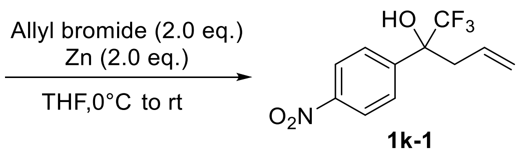

Trimethyl(2,2,2-trifluoro-1-methoxy-1-(4-nitrophenyl)ethoxy)silane (1k-3)

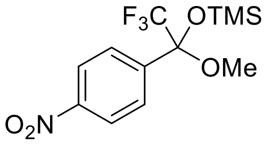

Methyl 4-nitrobenzoate (3.89 g, 21.50 mmol, 1.0 eq.) and $TMSCF_3$ (4.1 mL, 26.70 mmol, 1.25 eq.) were dissolved in dry DCM (0.5 M) under argon and cooled to −78 °C. Tetrabutylammonium fluoride (1.0 M in THF, 2.5 mol%) was added dropwise and the resulting mixture was stirred at −78 °C for 30 min. The reaction mixture was then allowed to stir at 0 °C until TLC indicated consumption of the ester. The reaction was quenched by dropwise addition of 1N HCl. The organic layer was separated and the aqueous layer was extracted with DCM (3 × 100 mL). The organic layers were combined and dried over Na_2SO_4, filtered, and concentrated under reduced pressure. The crude residue was subjected to flash chromatography (*n*-pentane:EtOAc 250:1) to yield the title compound as a colorless oil (4.40 g, 13.61 mmol, 32%).

$\mathbf{R_f}$ = 0.78 (*n*-pentane:EtOAc 10:1).

^{1}H NMR (400 MHz, $CDCl_3$): δ[ppm] = 8.43 – 8.10 (m, 2H), 7.78 (d, *J* = 8.6 Hz, 2H), 3.23 (s, 3H), 0.29 (s, 9H).

^{19}F NMR (376 MHz, $CDCl_3$): δ[ppm] = -80.44 (s, 3F).

^{19}F{^{1}H} NMR (376 MHz, $CDCl_3$): δ[ppm] = -80.44 (s, 3F).

ESI-MS: (*m/z*) requires: [$(C_{12}H_{16}NO_4SiF_3Na)^+$] = 346.0693, (*m/z*) found: [$(C_{12}H_{16}NO_4SiF_3Na)^+$] = 346.0694.

The analytical data were in good agreement with the literature.[12]

1,1,1-Trifluoro-2-(4-nitrophenyl)pent-4-en-2-ol (1k-1)

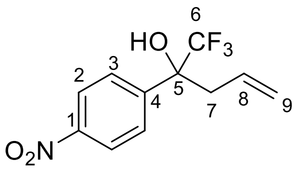

To a stirred solution of aqueous HCl (4 M) was added trimethyl (2,2,2-trifluoro-1-methoxy-1-(4-nitrophenyl)ethoxy)silane **(1k-3)** (2.10 g, 6.50 mmol, 1.0 eq.). After stirring for 12 h at room temperature, H_2O was added and the aqueous layer was extracted with EtOAc (3 × 50 mL). The combined organic layers were dried over Na_2SO_4, filtered, and concentrated under reduced pressure. The crude residue was directly used in the next step without further purification.

2,2,2-Trifluoro-1-(4-nitrophenyl)ethan-1-one **(1k-2)** (6.50 mmol, 1.0 eq.), allyl bromide (2.0 eq.) and THF (20 mL) were added to a round bottom flask. Zn dust (2.0 eq.) was added in one portion at 0 ˚C and the reaction mixture was allowed to stir at room temperature for 24 h. H_2O was added and the aqueous layer was extracted with EtOAc (3 × 50 mL). The combined organic layers were dried over Na_2SO_4, filtered, and concentrated under reduced pressure. The crude residue was purified by column chromatography (*n*-pentane:EtOAc 10:1) to yield the title compound as a yellow solid (0.69 g, 2.6 mmol, 41%).

$\mathbf{R_f}$ = 0.21 (*n*-pentane:EtOAc 10:1).

Melting Point: 43-45 ˚C.

FT-IR ($\tilde{\nu}$ = cm^{-1})**:** 3480 (m), 3015 (w), 2970 (m), 2943 (w), 1739 (s), 1604 (w), 1515 (s), 1451 (m), 1416 (m), 1348 (s), 1302 (w), 1264 (m), 1231 (s), 1217 (m), 1203 (m), 1159 (s), 1111 (m), 1094 (m), 1021 (m), 995 (m), 971 (m), 937 (m), 893 (m), 850 (s), 761 (m), 746 (m), 717 (s), 675 (m), 595 (w), 572 (w), 527 (m), 516 (m).

^{1}H NMR (500 MHz, $CDCl_3$): δ [ppm] = 8.31 – 8.20 (m, 2H, H-C2), 7.92 – 7.69 (m, 2H, H-C3), 5.62 – 5.44 (m, 1H, H-C8), 5.34 – 5.20 (m, 2H, H-C9), 3.01 – 2.88 (m, 2H, H-C7), 2.62 (d, $^4J_{FH}$ = 3.2 Hz, 1H, OH).

^{13}**C NMR** (126 MHz, $CDCl_3$): δ [ppm] = 148.2 (C1), 144.0 (C4), 129.4 (C8), 127.9 (q, $^4J_{CF}$ = 1.5 Hz, C3), 125.0 (q, $^1J_{CF}$ = 285.8 Hz, C6), 123.6 (C2), 123.0 (C9), 76.0 (q, $^2J_{CF}$ = 28.7 Hz, C5), 10.6 (q, $^3J_{CF}$ = 1.2 Hz, C7).

^{19}**F NMR** (470 MHz, $CDCl_3$): δ [ppm] = -78.77 (q, $^4J_{HF}$ = 0.8 Hz, 3F, F-C6).

^{19}**F{^{1}H} NMR** (470 MHz, $CDCl_3$): δ [ppm] = -78.77 (s, 3F, F-C6).

GC-EI-MS: (*m/z*) requires: [$(C_{11}H_{10}F_3NO_3)^+$] = 261.0607, (*m/z*) found: [$(C_{11}H_{10}F_3NO_3)^+$] = 261.0607.

1-Nitro-4-(1,1,1-trifluoropenta-2,4-dien-2-yl)benzene (1k)

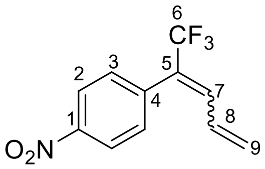

E/*Z* = 14.3:1

Compound **1k** was prepared according to **General Procedure C** 1,1,1-trifluoro-2-(4-nitrophenyl)pent-4-en-2-ol (**1k-1**) (0.68 g, 2.16 mmol, 1.0 eq.). The crude residue was purified by column chromatography (*n*-pentane:Et_2O 20:1) to yield the title compound as a yellow oil (0.31 g, 1.27 mmol, 59%, *E*:*Z* = 14.3:1).

R*f* = 0.39 (*n*-pentane: EtOAc 10:1).

FT-IR ($\tilde{\nu}$ = cm^{-1})**:** 3459 (w), 3073 (w), 3026 (w), 2970 (m), 1884 (w), 1739 (s), 1648 (w), 1598 (m), 1522 (m), 1426 (m), 1364 (s), 1348 (s), 1276 (s), 1229 (m), 1216 (m), 1200 (s), 1171 (m), 1142 (m), 1095 (s), 1016 (w), 1004 (m), 970 (m), 957 (m), 935 (s), 850 (s), 768 (w), 751 (m), 708 (s), 671 (m), 643 (m), 621 (m), 519 (m).

^{1}**H NMR** (500 MHz, $CDCl_3$):

E-diene: δ [ppm] = 8.33 – 8.25 (m, 2H, H-C2), 7.56 – 7.45 (m, 2H, H-C3), 6.96 (ddq, $^3J_{HH}$ = 11.1 Hz, $^4J_{HH}$ = 1.6 Hz, $^4J_{HF}$ = 0.8 Hz, 1H, H-C7), 6.28 – 6.08 (m, 1H, H-C8), 5.69 (ddq, $^3J_{HH}$ = 16.9 Hz, $^2J_{HH}$ = 1.5 Hz, $^6J_{HF}$ = 1.1 Hz, 1H, H^a-C9), 5.50 (ddq, $^3J_{HH}$ = 10.2 Hz, $^2J_{HH}$ = 1.5 Hz, $^6J_{HF}$ = 0.8 Hz, 1H, H^b-C9).

Z-diene: δ [ppm] = 8.25 – 8.20 (m, 2H, H-C2), 7.57 – 7.51 (m, 2H, H-C3), 6.99 – 6.97 (m, 1H, H-C8), 6.63 (dq, $^3J_{HH}$ = 11.5 Hz, $^4J_{HF}$ = 0.8 Hz, 1H, H-C7), 5.67 – 5.63 (m, 2H, H-C9).

^{13}**C NMR** (126 MHz, $CDCl_3$):

E-diene: δ [ppm] = 148.2 (C1), 138.8 (q, $^{3}J_{CF}$ = 1.0 Hz, C4), 135.4 (q, $^{3}J_{CF}$ = 5.8 Hz, C7), 131.2 (C3), 130.7 (C8), 128.9 (q, $^{2}J_{CF}$ = 30.6 Hz, C5), 127.2 (q, $^{5}J_{CF}$ = 1.3 Hz, C9), 123.8 (C2), 123.3 (q, $^{1}J_{CF}$ = 272.5 Hz, C6).

^{19}F NMR (470 MHz, $CDCl_3$): *E*-diene: δ [ppm] = -64.81 (s, 3F, F-C6); *Z*-diene: δ [ppm] = -56.50 (d, $^{4}J_{HF}$ = 2.1 Hz, 3F, F-C6).

^{19}F{^{1}H} NMR (470 MHz, $CDCl_3$): *E*-diene: δ [ppm] = -64.81 (s, 3F, F-C6); *Z*-diene: δ [ppm] = -56.50 (s, 3F, F-C6).

GC-EI-MS: (*m/z*) requires: [$(C_{11}H_8F_3NO_2)^+$] = 243.0502, (*m/z*) found: [$(C_{11}H_8F_3NO_2)^+$] = 243.0502.

4-(1,1,1-Trifluoro-2-hydroxypent-4-en-2-yl)benzonitrile (1l-1)

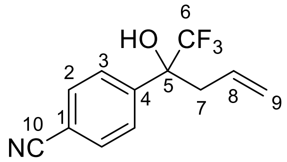

Compound **1l-1** was prepared according to **General Procedure A and B** (**Method 2**) using 4-formylbenzonitrile (1.32 g, 10.09 mmol, 1.0 eq.). The crude residue was purified by column chromatography (*n*-pentane:EtOAc 20:1) to yield the title compound as a white solid (0.36 g, 1.49 mmol, 15%).

R_f = 0.34 (*n*-pentane:EtOAc 10:1).

Melting Point: 103-104 °C.

FT-IR ($\tilde{\nu}$ = cm^{-1})**:** 3380 (w), 2982 (w), 2244 (w), 1645 (w), 1611 (w), 1507 (w), 1414 (w), 1264 (w), 1238 (w), 1150 (m), 1118 (w), 1100 (w), 1020 (w), 1002 (w), 974 (w), 944 (w), 918 (w), 894 (w), 835 (m), 788 (w), 744 (w), 714 (w), 697 (w).

^{1}H NMR (599 MHz, $CDCl_3$): δ [ppm] = 7.72 – 7.69 (m, 4H, H-C2, H-C3), 5.58 – 5.47 (m, 1H, H-C8), 5.29 – 5.21 (m, 2H, H-C9), 2.94 – 2.87 (m, 2H, H-C7), 2.76 (s, 1H, OH).

^{13}C NMR (151 MHz, $CDCl_3$): δ [ppm] = 142.2 (C4), 132.3 (C2), 129.5 (C8), 127.6 (q, $^{4}J_{CF}$ = 1.5 Hz, C3), 125.0 (q, $^{1}J_{CF}$ = 285.8 Hz, C6), 123.0 (C9), 118.5 (C10), 112.8 (C1), 75.9 (q, $^{2}J_{CF}$ = 28.6 Hz, C5), 40.5 (q, $^{3}J_{CF}$ = 1.3 Hz, C7).

^{19}F NMR (564 MHz, $CDCl_3$): δ [ppm] = -78.82 (s, 3F, F-C6).

^{19}F{^{1}H} NMR (564 MHz, $CDCl_3$): δ [ppm] = -78.82 (s, 3F, F-C6).

GC-EI-MS: (*m/z*) requires: [($C_{12}H_{10}F_3NO$)$^+$] = 241.0709, (*m/z*) found: [($C_{12}H_{10}F_3NO$)$^+$] = 241.0709.

4-(1,1,1-Trifluoropenta-2,4-dien-2-yl)benzonitrile (1l)

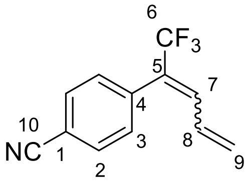

E/*Z* = 5.7:1

Compound **1l** was prepared according to **General Procedure C** using 4-(1,1,1-trifluoro-2-hydroxypent-4-en-2-yl)benzonitrile (**1l-1**) (0.30 g, 1.24 mmol, 1.0 eq.). The crude residue was purified by column chromatography (*n*-pentane:EtOAc 20:1) to yield the title compound as a colorless oil (0.13 g, 0.60 mmol, 48%, *E*:*Z* = 5.7:1).

R*f* = 0.45 (*n*-pentane:EtOAc 10:1).

FT-IR ($\tilde{\nu}$ = cm^{-1})**:** 2232 (w), 1653 (w), 1610 (w), 1506 (w), 1426 (w), 1367 (w), 1277 (m), 1200 (m), 1184 (m), 1169 (m), 1144 (m), 1109 (s), 1023 (w), 993 (m), 957 (m), 933 (m), 841 (m), 806 (w), 789 (w), 771 (w), 744 (m), 726 (w), 710 (w), 691 (w), 681 (w), 663 (m).

^{1}H NMR (500 MHz, $CDCl_3$):

E-diene: δ [ppm] = 7.77 – 7.68 (m, 2H, H-C2), 7.45 – 7.38 (m, 2H, H-C3), 6.97 – 6.89 (m, 1H, H-C7), 6.29 – 6.08 (m, 1H, H-C8), 5.67 (dd, $^{3}J_{HH}$ = 16.8 Hz, $^{2}J_{HH}$ = 1.2 Hz, 1H, H^a-C9), 5.54 – 5.43 (m, 1H, H^b-C9).

Z-diene: δ [ppm] = 7.68 – 7.63 (m, 2H, H-C2), 7.53 – 7.45 (m, 2H, H-C3), 7.00 – 6.94 (m, 1H, H-C8), 6.58 (dq, $^{3}J_{HH}$ = 11.6 Hz, $^{4}J_{HF}$ = 0.8 Hz, 1H, H-C7), 5.64 – 5.60 (m, 2H, H-C9).

^{13}C NMR (126 MHz, $CDCl_3$):

E-diene: δ [ppm] = 136.8 (C4), 135.0 (q, $^{3}J_{CF}$ = 5.8 Hz, C7), 132.2 (C2), 130.8 (C3), 130.7 (C8), 129.1 (q, $^{2}J_{CF}$ = 30.4 Hz, C5), 126.8 (q, $^{5}J_{CF}$ = 1.4 Hz, C9), 123.2 (q, $^{1}J_{CF}$ = 272.5 Hz, C6), 118.2 (C10), 112.8 (C1).

^{19}F NMR (470 MHz, $CDCl_3$): *E*-diene: δ [ppm] = -64.85 (s, 3F, F-C6); *Z*-diene: δ [ppm] = -56.51 (d, $^{4}J_{HF}$ = 2.1 Hz, 3F, F-C6).

^{19}F{^{1}H} NMR (470 MHz, $CDCl_3$): *E*-diene: δ [ppm] = -64.85 (s, 3F, F-C6); *Z*-diene: δ [ppm] = -56.51 (s, 3F, F-C6).

GC-EI-MS: (*m/z*) requires: [$(C_{12}H_8F_3N)^+$] = 223.0603, (*m/z*) found: [$(C_{12}H_8F_3N)^+$] = 223.0602.

Methyl 4-(1,1,1-trifluoro-2-hydroxypent-4-en-2-yl)benzoate (1m-1)

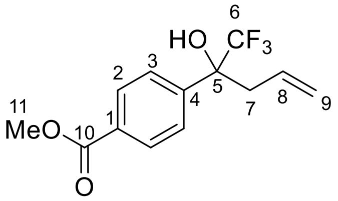

Compound **1m-1** was prepared according to **General Procedure A and B** (**Method 2**) using methyl 4-formylbenzoate (1.62 g, 9.88 mmol, 1.0 eq.). The crude residue was purified by column chromatography (*n*-pentane:EtOAc 25:1) to yield the title compound as a colorless liquid (1.37 g, 5.00 mmol, 51%).

R$_f$ = 0.31 (*n*-pentane:EtOAc 10:1).

FT-IR ($\tilde{\nu}$ = cm^{-1})**:** 3460 (w), 2956 (w), 1704 (m), 1644 (w), 1614 (w), 1577 (w), 1439 (m), 1412 (m), 1373 (w), 1283 (m), 1268 (m), 1234 (m), 1156 (s), 1111 (s), 1019 (m), 995 (m), 973 (m), 946 (m), 920 (m), 893 (w), 855 (m), 829 (w), 775 (m), 747 (m), 723 (s), 671 (m).

^{1}H NMR (500 MHz, $CDCl_3$): δ [ppm] = 8.12 – 8.02 (m, 2H, H-C2), 7.73 – 7.61 (m, 2H, H-C3), 5.61 – 5.46 (m, 1H, H-C8), 5.32 – 5.17 (m, 2H, H-C9), 3.92 (s, 3H, H-C11), 3.03 – 2.84 (m, 2H, H-C7), 2.81 (dd, $^3J_{HH}$ = 6.6 Hz, $^4J_{HF}$ = 3.3 Hz, 1H, OH).

^{13}C NMR (126 MHz, $CDCl_3$): δ [ppm] = 166.8 (C10), 141.9 (C4), 130.5 (C1), 130.0 (C8), 129.7 (C2), 126.8 (q, $^4J_{CF}$ = 1.3 Hz, C3), 125.2 (q, $^1J_{CF}$ = 285.8 Hz, C6), 122.5 (q, $^5J_{CF}$ = 2.1 Hz, C9), 76.0 (q, $^2J_{CF}$ = 28.4 Hz, C5), 52.4 (C11), 40.5 (q, $^3J_{CF}$ = 1.3 Hz, C7).

^{19}F NMR (470 MHz, $CDCl_3$): δ [ppm] = -78.93 (s, 3F, F-C6).

^{19}F{^{1}H} NMR (470 MHz, $CDCl_3$): δ [ppm] = -78.93 (s, 3F, F-C6).

ESI-MS: (*m/z*) requires: [$(C_{13}H_{13}F_3O_3Na)^+$] = 297.0709, (*m/z*) found: [$(C_{13}H_{13}F_3O_3Na)^+$] = 297.0710.

Methyl 4-(1,1,1-trifluoropenta-2,4-dien-2-yl)benzoate (1m)

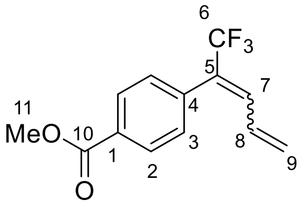

Compound **1m** was prepared according to **General Procedure C** using methyl 4-(1,1,1-trifluoro-2-hydroxypent-4-en-2-yl)benzoate (**1m-1**) (1.17 g, 4.27 mmol, 1.0 eq.). The crude residue was purified by column chromatography (*n*-pentane:EtOAc 50:1) to yield the title compound as a colorless oil (0.34 g, 1.33 mmol, 31%, *E*:*Z* = 14.2:1)

R_f = 0.61 (*n*-pentane:EtOAc 10:1).

FT-IR ($\tilde{\nu}$ = cm^{-1})**:** 2955 (w), 1725 (s), 1647 (w), 1614 (w), 1568 (w), 1437 (m), 1407 (w), 1367 (m), 1312 (w), 1271 (s), 1200 (m), 1172 (m), 1143 (m), 1103 (s), 1022 (m), 994 (m), 957 (m), 933 (s), 859 (m), 828 (m), 779 (m), 753 (m), 716 (s), 661 (m).

^{1}H NMR (599 MHz, $CDCl_3$):

E-diene: δ [ppm] = 8.22 – 7.94 (m, 2H, H-C2), 7.37 (d, $^3J_{HH}$ = 8.2 Hz, 2H, H-C3), 7.00 – 6.79 (m, 1H, H-C7), 6.37 – 6.11 (m, 1H, H-C8), 5.78 – 5.58 (m, 1H, H^a-C9), 5.43 (ddq, $^3J_{HH}$ = 10.1 Hz, $^2J_{HH}$ = 1.5 Hz, $^6J_{HF}$ = 0.8 Hz, 1H, H^b-C9), 3.94 (s, 3H, H -C11).

Z-diene: δ [ppm] = 8.05 – 8.02 (m, 2H, H-C2), 7.47 – 7.41 (m, 2H, H-C3), 7.00 – 6.92 (m, 1H, H-C8), 6.58 (dq, $^3J_{HH}$ = 11.6 Hz, $^4J_{HF}$ = 1.0 Hz, 1H, H-C7), 5.60 – 5.55 (m, 2H, H-C9), 3.93 (s, 3H, H -C11).

^{13}C NMR (151 MHz, $CDCl_3$):

E-diene: δ [ppm] = 166.7 (C10), 136.8 (C4), 134.5 (q, $^3J_{CF}$ = 5.9 Hz, C7), 131.3 (C8), 130.6 (C1), 130.2 (q, $^2J_{CF}$ = 30.3 Hz, C5), 130.2 (C3), 130.0 (C2), 126.0 (q, $^5J_{CF}$ = 1.4 Hz, C9), 123.6 (q, $^1J_{CF}$ = 272.5 Hz, C6), 52.4 (C11).

^{19}F NMR (564 MHz, $CDCl_3$): *E*-diene: δ [ppm] = -64.96 (s, 3F, F-C6); *Z*-diene: δ [ppm] = -56.49 (d, $^4J_{HF}$ = 2.1 Hz, 3F, F-C6).

^{19}F{^{1}H} NMR (564 MHz, $CDCl_3$): *E*-diene: δ [ppm] = -64.96 (s, 3F, F-C6); *Z*-diene: δ [ppm] = -56.49 (s, 3F, F-C6).

ESI-MS: (*m/z*) requires: $[(C_{13}H_{11}O_2F_3Na)^+]$ = 279.0603, (*m/z*) found: $[(C_{13}H_{11}O_2F_3Na)^+]$ = 279.0604.

1,1,1-Trifluoro-2-(4-(methylsulfonyl)phenyl)pent-4-en-2-ol (1n-1)

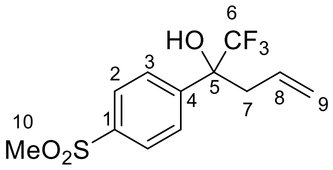

Compound **1n-1** was prepared according to **General Procedure A and B** (**Method 1**) using 4-(methylsulfonyl)benzaldehyde (1.85 g, 10.04 mmol, 1.0 eq.). The crude residue was purified by column chromatography (*n*-pentane:EtOAc 5:1) to yield the title compound as a white solid (0.43 g, 1.46 mmol, 15%).

R_f = 0.30 (*n*-pentane:EtOAc 4:1).

Melting Point: 94-95 ˚C.

FT-IR ($\tilde{\nu}$ = cm^{-1})**:** 3412 (w), 1644 (w), 1603 (w), 1408 (w), 1283 (m), 1268 (m), 1243 (w), 1185 (m), 1146 (m), 1090 (m), 1020 (w), 1001 (w), 979 (w), 952 (m), 927 (w), 876 (w), 834 (m), 781 (m), 750 (m), 735 (m), 704 (w), 683 (m).

^{1}H NMR (500 MHz, $CDCl_3$): δ [ppm] = 8.02 – 7.90 (m, 2H, H-C2), 7.82 – 7.75 (m, 2H, H-C3), 5.59 – 5.42 (m, 1H, H-C8), 5.32 – 5.16 (m, 2H, H-C9), 3.07 (s, 3H, H-C10), 3.00 (s, 1H, OH), 2.98 – 2.84 (m, 2H, H-C7).

^{13}C NMR (126 MHz, $CDCl_3$): δ [ppm] = 143.2 (C4), 140.8 (C1), 129.5 (C8), 127.9 (q, $^{4}J_{CF}$ = 1.4 Hz, C3), 127.5 (C2), 125.0 (q, $^{1}J_{CF}$ = 285.9 Hz, C6), 122.8 (C9), 76.0 (q, $^{2}J_{CF}$ = 28.6 Hz, C5), 44.5 (C10), 40.4 (q, $^{3}J_{CF}$ = 1.2 Hz, C7).

^{19}F NMR (470 MHz, $CDCl_3$): δ [ppm] = -78.79 (s, 3F, F-C6).

^{19}F{^{1}H} NMR (470 MHz, $CDCl_3$): δ [ppm] = -78.79 (s, 3F, F-C6).

ESI-MS: (*m/z*) requires: [$(C_{12}H_{13}SF_3O_3Na)^+$] = 317.0430, (*m/z*) found: [$(C_{12}H_{13}SF_3O_3Na)^+$] = 317.0428.

(E)-1-(Methylsulfonyl)-4-(1,1,1-trifluoropenta-2,4-dien-2-yl)benzene (1n)

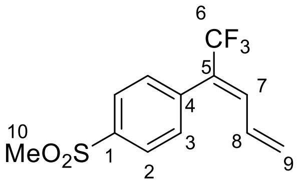

Compound **1n** was prepared according to **General Procedure C** using 1,1,1-trifluoro-2-(4-(methylsulfonyl)phenyl)pent-4-en-2-ol (**1n-1**) (0.41 g, 1.39 mmol, 1.0 eq.). The crude residue was purified by column chromatography (n-pentane:EtOAc 5:1) to yield the title compound as a white solid (0.12 g, 0.43 mmol, 31%, pure E isomer).

R_f = 0.21 (n-pentane:EtOAc 5:1).

Melting Point: 71-72 ˚C.

FT-IR ($\tilde{\nu}$ = cm^{-1})**:** 3067 (w), 1651 (w), 1603 (w), 1411 (w), 1369 (w), 1276 (m), 1209 (w), 1177 (m), 1158 (w), 1136 (w), 1104 (m), 1091 (m), 1020 (w), 1001 (m), 965 (w), 956 (m), 946 (m), 931 (m), 860 (w), 847 (w), 779 (m), 760 (m), 733 (m), 704 (w), 678 (w).

^{1}H NMR (599 MHz, $CDCl_3$): δ [ppm] = 8.16 – 7.91 (m, 2H, H-C2), 7.51 (d, $^3J_{HH}$ = 8.1 Hz, 2H, H-C3), 6.94 (ddqd, $^3J_{HH}$ = 11.1 Hz, $^4J_{HH}$ = 1.7 Hz, $^3J_{HF}$ = 1.7 Hz, $^4J_{HH}$ = 0.9 Hz, 1H, H-C7), 6.18 (dddq, $^3J_{HH}$ = 16.9 Hz, $^3J_{HH}$ = 11.2 Hz, $^3J_{HH}$ = 10.1 Hz, $^5J_{HF}$ = 1.1 Hz, 1H, H-C8), 5.67 (dq, $^3J_{HH}$ = 16.9 Hz, $^6J_{HF}$ = 1.1 Hz, 1H, H^a-C9), 5.48 (ddq, $^3J_{HH}$ = 10.2 Hz, $^2J_{HH}$ = 1.5 Hz, $^6J_{HF}$ = 0.8 Hz, 1H, H^b-C9), 3.10 (s, 3H, H -C10).

^{13}C NMR (151 MHz, $CDCl_3$): δ [ppm] = 141.0 (C1), 137.9 (C4), 135.3 (q, $^3J_{CF}$ = 5.8 Hz, C7), 131.2 (C3), 130.8 (C8), 129.2 (q, $^2J_{CF}$ = 30.4 Hz, C5), 127.7 (C2), 126.9 (q, $^5J_{CF}$ = 1.1 Hz, C9), 123.4 (q, $^1J_{CF}$ = 272.5 Hz, C6), 44.5 (C10).

^{19}F NMR (564 MHz, $CDCl_3$): δ [ppm] = -64.86 (s, 3F, F-C6).

^{19}F{^{1}H} NMR (564 MHz, $CDCl_3$): δ [ppm] = -64.86 (s, 3F, F-C6).

ESI-MS: (m/z) requires: [$(C_{12}H_{11}O_2SF_3Na)^+$] = 299.0324, ($m/z$) found: [$(C_{12}H_{11}O_2SF_3Na)^+$] = 299.0322.

3-(1,1,1-Trifluoro-2-hydroxypent-4-en-2-yl)phenyl 4-methylbenzenesulfonate (1o-1)

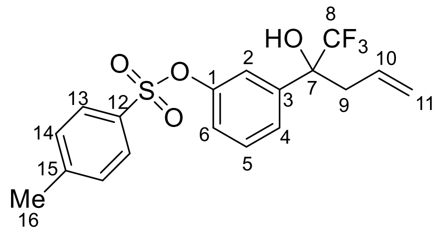

Compound **1o-1** was prepared according to **General Procedure A and B** (**Method 2**) using 3-formylphenyl 4-methylbenzenesulfonate (3.31 g, 12.00 mmol, 1.0 eq.). The crude residue was purified by column chromatography (*n*-pentane:EtOAc 6:1) to yield the title compound as a colorless oil (2.73 g, 7.49 mmol, 62%).

R_f = 0.21 (*n*-pentane:EtOAc 6:1).

FT-IR ($\tilde{\nu}$ = cm^{-1})**:** 3470 (w), 3015 (m), 2970 (m), 2946 (m), 1739 (s), 1598 (w), 1585 (w), 1486 (w), 1440 (m), 1367 (s), 1286 (w), 1266 (m), 1228 (s), 1216 (s), 1164 (s), 1092 (s), 1020 (w), 992 (w), 907 (m), 887 (m), 863 (m), 800 (m), 763 (s), 729 (m), 703 (m), 690 (m), 664 (m), 630 (m), 550 (s).

^{1}H NMR (500 MHz, $CDCl_3$): δ [ppm] = 7.72 – 7.62 (m, 2H, H-C13), 7.45 (ddd, $^3J_{HH}$ = 7.9 Hz, $^4J_{HH}$ = 1.8 Hz, $^4J_{HH}$ = 0.8 Hz, 1H, H-C4), 7.34 (dd, $^3J_{HH}$ = 8.0 Hz, $^3J_{HH}$ = 8.0 Hz, 1H, H-C5), 7.32 – 7.27 (m, 2H, H-C14), 7.10 (ddd, $^3J_{HH}$ = 8.2 Hz, $^4J_{HH}$ = 2.4 Hz, $^4J_{HH}$ = 1.0 Hz, 1H, H-C6), 7.04 (dd, $^4J_{HH}$ = 2.1 Hz, $^4J_{HH}$ = 2.1 Hz, 1H, H-C2), 5.47 – 5.35 (m, 1H, H-C10), 5.26 – 5.16 (m, 2H, H-C11), 2.83 – 2.70 (m, 2H, H-C9), 2.69 – 2.63 (m, 1H, OH), 2.42 (s, 3H, H-C16).

^{13}C NMR (126 MHz, $CDCl_3$): δ [ppm] = 149.7 (C1), 145.6 (C15), 139.0 (C3), 132.0 (C12), 129.84 (C14), 129.80 (C10), 129.7 (C5), 128.6 (C13), 125.3 (q, $^4J_{CF}$ = 1.3 Hz, C4), 125.0 (q, $^1J_{CF}$ = 285.4 Hz, C8), 122.8 (C6), 122.4 (C11), 121.0 (q, $^4J_{CF}$ = 1.4 Hz, C2), 75.5 (q, $^2J_{CF}$ = 28.6 Hz, C7), 40.2 (q, $^3J_{CF}$ = 1.3 Hz, C9), 21.7 (C16).

^{19}F NMR (470 MHz, $CDCl_3$): δ [ppm] = -79.27 (s, 3F, F-C8).

^{19}F{^{1}H} NMR (470 MHz, $CDCl_3$): δ [ppm] = -79.27 (s, 3F, F-C8).

ESI-MS: (*m/z*) requires: [$(C_{18}H_{17}O_4SF_3Na)^+$] = 409.0692, (*m/z*) found: [$(C_{18}H_{17}O_4SF_3Na)^+$] = 409.0690.

3-(1,1,1-Trifluoropenta-2,4-dien-2-yl)phenyl 4-methylbenzenesulfonate (1o)

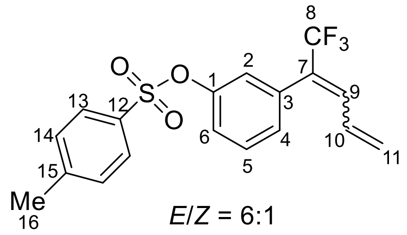

Compound **1o** was prepared according to **General Procedure C** using 3-(1,1,1-trifluoro-2-hydroxypent-4-en-2-yl)phenyl 4-methylbenzenesulfonate (**1o-1**) (2.70 g, 7.41 mmol, 1.0 eq.). The crude residue was purified by column chromatography (*n*-pentane:Et_2O 20:1) to yield the title compound as a colorless oil (0.92 g, 2.66 mmol, 36%, *E*:*Z* = 6:1).

R*f* = 0.31 (*n*-pentane:Et_2O 10:1).

FT-IR ($\tilde{\nu}$ = cm^{-1})**:** 3458 (w), 3015 (m), 2970 (m), 1739 (s), 1598 (w), 1576 (w), 1486 (w), 1434 (m), 1373 (s), 1281 (m), 1228 (m), 1216 (s), 1177 (s), 1152 (m), 1113 (s), 1091 (s), 981 (m), 932 (m), 896 (m), 872 (s), 802 (s), 766 (s), 726 (m), 701 (m), 680 (m), 660 (m), 628 (m), 561 (s), 551 (s).

^{1}H NMR (500 MHz, $CDCl_3$):

E-diene: δ [ppm] = 7.72 – 7.63 (m, 2H, H-C13), 7.36 (dd, $^3J_{HH}$ = 8.0 Hz, $^3J_{HH}$ = 8.0 Hz, 1H, H-C5), 7.32 – 7.27 (m, 2H, H-C14), 7.20 – 7.11 (m, 2H, H-C4, H-C6), 6.80 (dqd, $^3J_{HH}$ = 11.0 Hz, $^4J_{HF}$ = 1.6 Hz, $^4J_{HH}$ = 0.8 Hz, 1H, H-C9), 6.77 (dd, $^4J_{HH}$ = 2.0 Hz, $^4J_{HH}$ = 2.0 Hz, 1H, H-C2), 6.17 – 5.99 (m, 1H, H-C10), 5.58 (ddq, $^3J_{HH}$ = 17.7 Hz, $^2J_{HH}$ = 1.8 Hz, $^6J_{HF}$ = 0.9 Hz, 1H, H^a-C11), 5.39 (ddq, $^3J_{HH}$ = 10.1 Hz, $^2J_{HH}$ = 1.6 Hz, $^6J_{HF}$ = 0.8 Hz, 1H, H^b-C11), 2.43 (s, 3H, H-C16).

Z-diene: δ [ppm] = 7.72 – 7.69 (m, 2H, H-C13), 7.34 – 7.27 (m, 3H, H-C14, H-C5), 7.23 (ddd, $^3J_{HH}$ = 7.8 Hz, $^4J_{HH}$ = 1.8 Hz, $^4J_{HH}$ = 0.9 Hz, 1H, H-C6), 7.03 (ddd, $^3J_{HH}$ = 8.1 Hz, $^4J_{HH}$ = 2.3 Hz, $^4J_{HH}$ = 1.1 Hz, 1H, H-C4), 6.94 – 6.83 (m, 2H, H-C10, H-C2), 6.40 (dq, $^3J_{HH}$ = 11.5 Hz, $^4J_{HF}$ = 0.9 Hz, 1H, H-C9), 5.57 – 5.51 (m, 2H, H-C11), 2.44 (s, 3H, H-C16)..

^{13}C NMR (126 MHz, $CDCl_3$):

E-diene: δ [ppm] = 149.7 (C1), 145.7 (C15), 134.5 (q, $^3J_{CF}$ = 5.8 Hz, C9), 133.6 (C3), 132.1 (C12), 131.2 (C10), 129.93 (C14), 129.89 (C5), 129.3 (q, $^2J_{CF}$ = 30.2 Hz, C7), 128.8 (C4), 128.6 (C13), 126.0 (q, $^5J_{CF}$ = 1.4 Hz, C11), 124.1 (C2), 123.5 (q, $^1J_{CF}$ = 240.1 Hz, C8), 123.2 (C6), 21.8 (C16).

^{19}F NMR (470 MHz, $CDCl_3$): *E*-diene: δ [ppm] = -65.25 (s, 3F, F-C8); *Z*-diene: δ [ppm] = -56.76 (d, $^4J_{HF}$ = 2.1 Hz, 3F, F-C8).

^{19}F{^{1}H} NMR (470 MHz, $CDCl_3$): *E*-diene: δ [ppm] = -65.25 (s, 3F, F-C8); *Z*-diene: δ [ppm] = -56.76 (s, 3F, F-C8).

ESI-MS: (*m/z*) requires: [$(C_{18}H_{15}O_3SF_3Na)^+$] = 391.0586, (*m/z*) found: [$(C_{18}H_{15}O_3SF_3Na)^+$] = 391.0585.

1,1,1-Trifluoro-2-(3-(trifluoromethoxy)phenyl)pent-4-en-2-ol (1p-1)

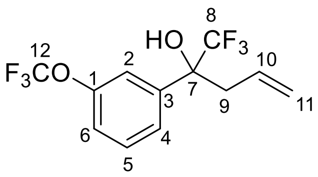

Compound **1p-1** was prepared according to **General Procedure A and B** (**Method 1**) using 3-(trifluoromethoxy)benzaldehyde (1.89 g, 9.94 mmol, 1.0 eq.). The crude residue was purified by column chromatography (*n*-pentane:EtOAc 40:1) to yield the title compound as a colorless oil (1.36 g, 4.53 mmol, 46%).

R_f = 0.36 (*n*-pentane:EtOAc 10:1).

FT-IR ($\tilde{\nu}$ = cm^{-1})**:** 3549 (w), 1612 (w), 1592 (w), 1492 (w), 1446 (w), 1256 (m), 1214 (m), 1149 (s), 1082 (m), 1022 (m), 996 (m), 933 (m), 885 (w), 836 (w), 794 (m), 729 (m), 703 (m).

^{1}H NMR (500 MHz, $CDCl_3$): δ [ppm] = 7.50 (dqdd, $^3J_{HH}$ = 8.0 Hz, $^5J_{HF}$ = 1.8 Hz, $^4J_{HH}$ = 0.9 Hz, $^4J_{HH}$ = 0.9 Hz, 1H, H-C6), 7.49 – 7.46 (m, 1H, H-C2), 7.46 – 7.41 (m, 1H, H-C5), 7.24 (dqdd, $^3J_{HH}$ = 8.1 Hz, $^5J_{HF}$ = 2.3 Hz, $^4J_{HH}$ = 1.1 Hz, $^4J_{HH}$ = 1.1 Hz, 1H, H-C4), 5.56 (ddddq, $^3J_{HH}$ = 17.5 Hz, $^3J_{HH}$ = 9.6 Hz, $^3J_{HH}$ = 7.8 Hz, $^3J_{HH}$ = 6.7 Hz, $^5J_{HF}$ = 0.9 Hz, 1H, H-C10), 5.36 – 5.22 (m, 2H, H-C11), 2.99 – 2.90 (m, 1H, H^a-C9), 2.87 (ddq, $^2J_{HH}$ = 14.3 Hz, $^3J_{HH}$ = 7.9 Hz, $^4J_{HF}$ = 1.0 Hz, 1H, H^b-C9), 2.67 (s, 1H, OH).

^{13}C NMR (126 MHz, $CDCl_3$): δ [ppm] = 149.5 (q, $^3J_{CF}$ = 1.9 Hz, C1), 139.5 (C3), 129.9 (C10, C5), 125.2 (q, $^1J_{CF}$ = 285.5 Hz, C8), 125.0 (q, $^4J_{CF}$ = 1.4 Hz, C6), 122.8 (C11), 121.1 (q, $^4J_{CF}$ = 1.0 Hz, C4), 120.6 (q, $^1J_{CF}$ = 257.4 Hz, C12), 119.8 (m, C2), 75.7 (q, $^2J_{CF}$ = 28.5 Hz, C7), 40.6 (q, $^3J_{CF}$ = 1.2 Hz, C9).

^{19}F NMR (470 MHz, $CDCl_3$): δ [ppm] = -57.92 (t, $^5J_{HF}$ = 1.0 Hz, 3F, F-C12), -79.15 (s, 3F, F-C8).

^{19}F{^{1}H} NMR (470 MHz, $CDCl_3$): δ [ppm] = -57.92 (s, 3F, F-C12), -79.15 (s, 3F, F-C8).

GC-EI-MS: (*m/z*) requires: $[(C_{12}H_{10}F_6O_2)^+]$ = 300.0580, (*m/z*) found: $[(C_{12}H_{10}F_6O_2)^+]$ = 300.0577.

1-(Trifluoromethoxy)-3-(1,1,1-trifluoropenta-2,4-dien-2-yl)benzene (1p)

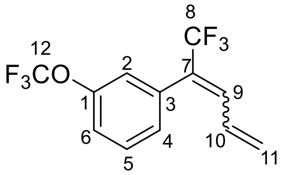

E/*Z* = 4:1

Compound **1p** was prepared according to **General Procedure C** using 1,1,1-trifluoro-2-(3-(trifluoromethoxy)phenyl)pent-4-en-2-ol (**1p-1**) (1.33 g, 4.40 mmol, 1.0 eq.). The crude residue was purified by column chromatography (*n*-pentane) to yield the title compound as a colorless oil (0.58 g, 2.06 mmol, 47%, *E*:*Z* = 4:1).

R*f* = 0.62 (*n*-pentane).

FT-IR ($\tilde{\nu}$ = cm^{-1})**:** 1650 (w), 1611 (w), 1582 (w), 1491 (w), 1439 (w), 1367 (w), 1254 (s), 1212 (s), 1197 (s), 1153 (s), 1114 (s), 993 (m), 951(m), 933 (m), 888 (w), 843 (w), 795 (m), 721 (m), 701 (m), 660 (m).

^{1}H NMR (500 MHz, $CDCl_3$):

E-diene: δ [ppm] = 7.39 (ddd, $^3J_{HH}$ = 8.0 Hz, $^4J_{HH}$ = 2.1 Hz, $^4J_{HH}$ = 1.3 Hz, 1H, H-C6), 7.35 (dd, $^3J_{HH}$ = 8.1 Hz, $^3J_{HH}$ = 7.4 Hz, 1H, H-C5), 7.31 – 7.29 (m, 1H, H-C2), 7.18 (ddq, $^3J_{HH}$ = 7.4 Hz, $^4J_{HH}$ = 1.5 Hz, $^5J_{HF}$ = 0.8 Hz, 1H, H-C4), 6.88 (dqd, $^3J_{HH}$ = 11.0 Hz, $^4J_{HF}$ = 1.6 Hz, $^4J_{HH}$ = 0.8 Hz, 1H, H-C9), 6.31 – 6.18 (m, 1H, H-C10), 5.62 (ddq, $^3J_{HH}$ = 17.0 Hz, $^2J_{HH}$ = 1.9 Hz, $^6J_{HF}$ = 1.0 Hz, 1H, H^a-C11), 5.44 (ddq, $^3J_{HH}$ = 10.1 Hz, $^2J_{HH}$ = 1.5 Hz, $^6J_{HF}$ = 0.7 Hz, 1H, H^b-C11).

Z-diene: δ [ppm] = 7.41 (dd, $^3J_{HH}$ = 8.9 Hz, $^3J_{HH}$ = 7.8 Hz, 1H, H-C5), 7.34 – 7.20 (m, 3H, H-C2, H-C4, H-C6), 7.01 – 6.93 (m, 1H, H-C10), 6.56 (dq, $^3J_{HH}$ = 11.5 Hz, $^4J_{HF}$ = 0.8 Hz, 1H, H-C9), 5.63 – 5.55 (m, 2H, H-C11).

^{13}C NMR (126 MHz, $CDCl_3$):

E-diene: δ [ppm] = 149.4 (q, $^3J_{CF}$ = 1.9 Hz, C1), 134.7 (q, $^3J_{CF}$ = 5.9 Hz, C9), 134.0 (C3), 131.2 (C10), 130.1 (C5), 129.5 (q, $^2J_{CF}$ = 30.3 Hz, C7), 128.5 (C4), 126.2 (q, $^5J_{CF}$ = 1.4 Hz, C11), 123.6 (q, $^1J_{CF}$ = 272.4 Hz, C8), 122.8 (q, $^4J_{CF}$ = 1.0 Hz, C2), 121.3 (q, $^4J_{CF}$ = 1.1 Hz, C6), 120.6 (q, $^1J_{CF}$ = 255.7 Hz, C12).

^{19}F NMR (470 MHz, $CDCl_3$): *E*-diene: δ [ppm] = -57.97 (t, $^5J_{HF}$ = 1.0 Hz, 3F, F-C12), -65.22 (d, $^4J_{HF}$ = 1.6 Hz, 3F, F-C8); *Z*-diene: δ [ppm] = -56.73 (d, $^4J_{HF}$ = 2.1 Hz, 3F, F-C8), -57.91 (t, $^5J_{HF}$ = 1.0 Hz, 3F, F-C12).

^{19}F{^{1}H} NMR (470 MHz, $CDCl_3$): *E*-diene: δ [ppm] = -57.97 (s, 3F, F-C12), -65.22 (s, 3F, F-C8); *Z*-diene: δ [ppm] = -56.73 (s, 3F, F-C8), -57.91 (s, 3F, F-C12).

GC-EI-MS: (*m/z*) requires: [$(C_{12}H_8F_6O)^+$] = 282.0474, (*m/z*) found: [$(C_{12}H_8F_6O)^+$] = 282.0475.

4-(1,1,1-Trifluoro-2-hydroxypent-4-en-2-yl)phenyl trifluoromethanesulfonate (1q-1)

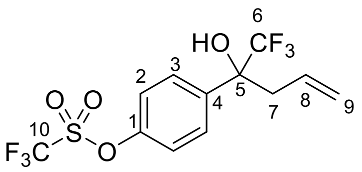

Compound **1q-1** was prepared according to **General Procedure A and B** (**Method 2**) using 4-formylphenyl trifluoromethanesulfonate (2.69 g, 10.58 mmol, 1.0 eq.). The crude residue was purified by column chromatography (*n*-pentane:EtOAc 40:1) to yield the title compound as a colorless liquid (1.35 g, 3.71 mmol, 35%).

R_f = 0.49 (*n*-pentane:EtOAc 10:1).

FT-IR ($\tilde{\nu}$ = cm^{-1})**:** 3553 (w), 3015 (m), 2970 (m), 1739 (s), 1503 (m), 1424 (m), 1366 (m), 1271 (w), 1216 (s), 1161 (m), 1136 (s), 1093 (m), 1016 (m), 995 (w), 883 (s), 838 (s), 784 (m), 763 (m), 742 (m), 708 (m), 606 (s), 575 (m), 528 (m), 504 (m).

^{1}H NMR (500 MHz, $CDCl_3$): δ [ppm] = 7.73 – 7.64 (m, 2H, H-C3), 7.36 – 7.29 (m, 2H, H-C2), 5.63 – 5.49 (m, 1H, H-C8), 5.32 – 5.21 (m, 2H, H-C9), 2.99 – 2.84 (m, 2H, H-C7), 2.72 (s, 1H, OH).

^{13}**C NMR** (126 MHz, $CDCl_3$): δ [ppm] = 149.7 (C1), 137.6 (C4), 129.7 (C8), 128.9 (q, $^{5}J_{CF}$ = 1.4 Hz, C3), 125.1 (q, $^{1}J_{CF}$ = 285.5 Hz, C6), 122.9 (C9), 121.4 (C2), 118.9 (q, $^{1}J_{CF}$ = 320.7 Hz, C10), 75.6 (q, $^{2}J_{CF}$ = 28.7 Hz, C5), 40.6 (q, $^{3}J_{CF}$ = 1.2 Hz, C7).

^{19}**F NMR** (470 MHz, $CDCl_3$): δ [ppm] = -72.92 (s, 3F, F-C10), -79.08 (d, $^{4}J_{HF}$ = 0.7 Hz, 3F, F-C6).

^{19}**F{^{1}H} NMR** (470 MHz, $CDCl_3$): δ [ppm] = -72.92 (s, 3F, F-C10), -79.08 (s, 3F, F-C6).

ESI-MS: (*m/z*) requires: [$(C_{12}H_{10}SF_6O_4Na)^+$] = 387.0096, (*m/z*) found: [$(C_{12}H_{10}SF_6O_4Na)^+$] = 387.0098.

(*E*)-4-(1,1,1-Trifluoropenta-2,4-dien-2-yl)phenyl trifluoromethanesulfonate (1q)

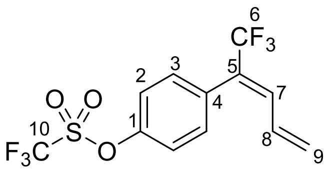

Pure *E*

Compound **1q** was prepared according to **General Procedure C** using 4-(1,1,1-trifluoro-2-hydroxypent-4-en-2-yl)phenyl trifluoro methanesulfonate (**1q-1**) (1.20 g, 3.29 mmol, 1.0 eq.). The crude residue was purified by column chromatography (*n*-pentane:Et_2O 200:1) to yield the title compound as a colorless oil (0.25 g, 0.72 mmol, 22%, pure *E* isomer).

$\mathbf{R_f}$ = 0.41 (*n*-pentane: Et_2O 10:1).

FT-IR ($\tilde{\nu}$ = cm^{-1})**:** 3459 (w), 3015 (m), 2970 (m), 2946 (m), 1739 (s), 1503 (m), 1425 (s), 1366 (s), 1281 (m), 1250 (m), 1216 (s), 1175 (m), 1136 (s), 1117 (s), 1020 (w), 994 (m), 931 (m), 879 (s), 843 (s), 788 (m), 765 (m), 743 (m), 710 (m), 664 (m), 628 (m), 605 (s), 574 (m), 537 (m), 527 (m), 513 (m).

^{1}**H NMR** (500 MHz, $CDCl_3$): δ [ppm] = 7.41 – 7.37 (m, 2H, H-C3), 7.36 – 7.31 (m, 2H, H-C2), 6.91 (ddq, $^{3}J_{HH}$ = 10.9 Hz, $^{4}J_{HH}$ = 1.6 Hz, $^{3}J_{HF}$ = 0.8 Hz, 1H, H-C7), 6.20 (dddq, $^{3}J_{HH}$ = 16.9 Hz, $^{3}J_{HH}$ = 11.2 Hz, $^{3}J_{HH}$ = 10.1 Hz, $^{5}J_{HF}$ = 1.1 Hz, 1H, H-C8), 5.65 (dq, $^{3}J_{HH}$ = 16.9 Hz, $^{6}J_{HF}$ = 1.1 Hz, 1H, H^a-C9), 5.47 (ddq, $^{3}J_{HH}$ = 10.1 Hz, $^{2}J_{HH}$ = 1.5 Hz, $^{6}J_{HF}$ = 0.8 Hz, 1H, H^b-C9).

^{13}**C NMR** (126 MHz, $CDCl_3$): δ[ppm] = 149.8 (C1), 135.0 (q, $^3J_{CF}$ = 5.8 Hz, C7), 132.6 (C4), 132.2 (C3), 131.1 (C8), 129.2 (q, $^2J_{CF}$ = 30.4 Hz, C5), 126.4 (q, $^5J_{CF}$ = 1.3 Hz, C9), 123.5 (q, $^1J_{CF}$ = 272.4 Hz, C6), 121.7 (C2), 118.9 (q, $^1J_{CF}$ = 320.7 Hz, C10).

^{19}**F NMR** (470 MHz, $CDCl_3$): δ [ppm] = -63.30 – -67.41 (m, 3F, F-C6), -72.86 (s, 3F, F-C10).

^{19}**F{^{1}H} NMR** (470 MHz, $CDCl_3$): δ[ppm] = -65.20 (s, 3F, F-C6), -72.86 (s, 3F, F-C10).

ESI-MS: (*m/z*) requires: [$(C_{12}H_8O_3SF_6Na)^+$] = 368.9991, (*m/z*) found: [$(C_{12}H_8O_3SF_6Na)^+$] = 368.9991.

4-(1,1,1-Trifluoro-2-hydroxy-4-methylpent-4-en-2-yl)phenyl 4-methylbenzenesulfonate (1r-1)

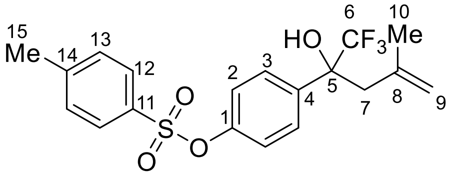

Compound **1r-1** was prepared according to **General Procedure A and B** (**Method 2**) using 4-formylphenyl 4-methylbenzenesulfonate (4.17 g, 15.08 mmol, 1.0 eq.) and 3-bromo-2-methylprop-1-ene (1.2 eq.). The crude residue was purified by column chromatography (*n*-pentane:EtOAc 10:1) to yield the title compound as a white solid (4.12 g, 10.66 mmol, 71%).

$\mathbf{R_f}$ = 0.41 (*n*-pentane:EtOAc 5:1).

Melting Point: 94-96 ˚C.

FT-IR ($\tilde{\nu}$ = cm^{-1})**:** 3474 (w), 3015 (m), 2970 (m), 1739 (s), 1596 (w), 1502 (m), 1443 (m), 1364 (s), 1295 (w), 1263 (m), 1228 (s), 1216 (s), 1152 (s), 1092 (m), 1037 (w), 1015 (m), 976 (w), 939 (w), 905 (m), 871 (m), 849 (m), 815 (m), 767 (m), 752 (m), 715 (m), 670 (m), 650 (m), 636 (m), 623 (w), 568 (m), 549 (m), 518 (m).

^{1}**H NMR** (500 MHz, $CDCl_3$): δ[ppm] = 7.70 – 7.63 (m, 2H, H-C12), 7.57 – 7.48 (m, 2H, H-C3), 7.28 (d, $^3J_{HH}$ = 8.1 Hz, 2H, H-C13), 7.06 – 6.97 (m, 2H, H-C2), 4.97 (dq, $^2J_{HH}$ = 1.6 Hz, $^4J_{HH}$ = 1.6 Hz, 1H, H^a-C9), 4.97 (dt, $^2J_{HH}$ = 1.6 Hz, $^4J_{HH}$ = 0.9 Hz, 1H, H^b-C9), 2.93 – 2.83 (m, 2H, H-C7), 2.81 – 2.78 (m, 1H, OH) 2.43 (s, 3H, H-C15), 1.34 (s, 3H, H-C10).

^{13}**C NMR** (126 MHz, $CDCl_3$): δ [ppm] = 149.8 (C1), 145.6 (C14), 139.2 (C8), 136.5 (C4), 132.3 (C11), 129.8 (C13), 128.7 (C12), 128.1 (q, $^4J_{CF}$ = 1.5 Hz, C3), 125.2 (q, $^1J_{CF}$ = 285.4 Hz, C6), 122.2 (C2), 118.5 (C9), 75.1 (q, $^2J_{CF}$ = 28.3 Hz, C5), 43.6 (C7), 23.8 (C10), 21.8 (C15).

^{19}**F NMR** (470 MHz, $CDCl_3$): δ [ppm] = -79.57 (s, 3F, F-C6).

^{19}**F{^{1}H} NMR** (470 MHz, $CDCl_3$): δ [ppm] = -79.57 (s, 3F, F-C6).

ESI-MS: (*m/z*) requires: [$(C_{19}H_{19}F_3O_4SNa)^+$] = 423.0854, (*m/z*) found: [$(C_{19}H_{19}F_3O_4SNa)^+$] = 423.0841.

(*E*)-4-(1,1,1-Trifluoro-4-methylpenta-2,4-dien-2-yl)phenyl 4-methylbenzenesulfonate (1r)

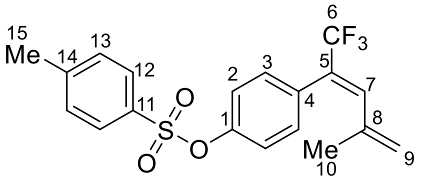

Pure *E*

Compound **1r** was prepared according to **General Procedure C** using 4-(1,1,1-trifluoro-2-hydroxy-4-methyl pent-4-en-2-yl)phenyl 4-methylbenzenesulfonate (**1r-1**) (4.02 g, 6.40 mmol, 1.0 eq.). The crude residue was purified by column chromatography (*n*-pentane:EtOAc 40:1) to yield the title compound as a colorless oil (0.83 g, 2.17 mmol, 34%, pure *E* isomer).

R*f* = 0.41 (*n*-pentane: EtOAc 15:1).

FT-IR ($\tilde{\nu}$ = cm^{-1})**:** 3456 (w), 2970 (m), 2925 (w), 1739 (m), 1645 (w), 1598 (w), 1503 (m), 1440 (w), 1373 (s), 1276 (m), 1229 (m), 1216 (m), 1200 (m), 1175 (m), 1156 (s), 1111 (s), 1092 (s), 1034 (w), 1019 (m), 986 (m), 954 (m), 927 (m), 860 (s), 836 (s), 814 (s), 771 (s), 754 (m), 707 (m), 681 (m), 655 (m), 625 (s), 571 (s), 551 (s), 517 (m).

^{1}**H NMR** (500 MHz, $CDCl_3$): δ [ppm] = 7.71 – 7.63 (m, 2H, H-C12), 7.36 – 7.26 (m, 2H, H-C13), 7.24 – 7.14 (m, 2H, H-C3), 7.04 – 6.95 (m, 2H, H-C2), 6.91 – 6.72 (m, 1H, H-C7), 5.40 – 4.99 (m, 2H, H-C9), 2.44 (s, 3H, H-C15), 1.31 (dd, $^4J_{HH}$ = 1.5 Hz, $^4J_{HH}$ = 0.8 Hz, 3H, H-C10).

^{13}**C NMR** (126 MHz, $CDCl_3$): δ [ppm] = 150.0 (C1), 145.7 (C14), 139.3 (C8), 136.3 (q, $^3J_{CF}$ = 5.7 Hz, C7), 132.2 (C11), 131.8 (C4), 131.7 (C3), 129.9 (C13), 128.7 (C12), 128.2 (q, $^2J_{CF}$ = 29.5 Hz, C5), 124.7 (C9), 123.6 (q, $^1J_{CF}$ = 273.3 Hz, C6), 122.2 (C2), 21.8 (C15), 21.4 (C10).

^{19}**F NMR** (470 MHz, $CDCl_3$): δ [ppm] = -65.99 (d, $^4J_{HF}$ = 1.5 Hz, 3F, F-C6).

^{19}**F{^{1}H} NMR** (470 MHz, $CDCl_3$): δ [ppm] = -65.99 (s, 3F, F-C6).

ESI-MS: (*m/z*) requires: [$(C_{17}H_{17}O_3SF_3Na)^+$] = 405.0748, (*m/z*) found: [$(C_{17}H_{17}O_3SF_3Na)^+$] = 405.0741.

2-Cyclohexyl-1,1,1-trifluoropent-4-en-2-ol (1s-1)

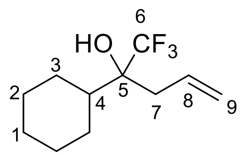

Compound **1s-1** was prepared according to **General Procedure A and B** (**Method 1**) using cyclohexanecarbaldehyde (1.18 g, 10.52 mmol, 1.0 eq.). The crude residue was purified by column chromatography (*n*-pentane:EtOAc 60:1) to yield the title compound as a colorless liquid (0.77 g, 3.46 mmol, 33%).

R$_f$ = 0.69 (*n*-pentane:EtOAc 10:1).

FT-IR ($\tilde{\nu}$ = cm^{-1})**:** 3543 (w), 3079 (w), 1642 (w), 1596 (w), 1569 (w), 1476 (w), 1445 (w), 1421 (w), 1369 (w), 1265 (m), 1230 (m), 1156 (s), 1176 (m), 1018 (m), 996 (m), 954 (m), 924 (m), 890 (m), 786 (m), 768 (m), 725 (m), 698 (m), 688 (m), 674 (m).

^{1}**H NMR** (500 MHz, $CDCl_3$): δ [ppm] = 5.94 – 5.71 (m, 1H, H-C8), 5.26 (ddq, $^2J_{HH}$ = 10.1 Hz, $^1J_{HH}$ = 1.9 Hz, $^4J_{HH}$ = 0.9 Hz, 1H, H^a-C9), 5.20 (dd, $^2J_{HH}$ = 14.3 Hz, $^1J_{HH}$ = 1.9 Hz, 1H, H^b-C9), 2.67 – 2.31 (m, 2H, H-C7), 2.23 (s, 1H, OH), 2.06 – 1.61 (m, 6H, H-C1, H-C2, H-C3, H-C4), 1.39 – 1.03 (m, 5H, H-C1, H-C2, H-C3).

^{13}**C NMR** (126 MHz, $CDCl_3$): δ [ppm] = 131.5 (q, $^4J_{CF}$ = 1.3 Hz, C8), 126.8 (q, $^1J_{CF}$ = 288.6 Hz, C6), 121.8 (C9), 76.5 (q, $^2J_{CF}$ = 25.4 Hz, C5), 44.2 (C4), 27.0 (q, $^3J_{CF}$ = 1.5 Hz, C7), 27.0 (m, C3), 26.83 (C2), 26.78 (C2'), 26.5 (C1).

^{19}**F NMR** (470 MHz, $CDCl_3$): δ [ppm] = -74.20 (s, 3F, F-C6).

$^{19}F\{^1H\}$ NMR (470 MHz, $CDCl_3$): δ [ppm] = -74.20 (s, 3F, F-C6).

ESI-MS: (*m/z*) requires: [($C_{11}H_{17}F_3O$-H)$^-$] = 221.1159, (*m/z*) found: [($C_{11}H_{17}F_3O$-H)$^-$] = 221.1159.

(1,1,1-Trifluoropenta-2,4-dien-2-yl)cyclohexane (1s)

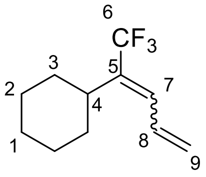

E/*Z* = 1:1.6

Compound **1s** was prepared according to **General Procedure C** using 2-cyclohexyl-1,1,1-trifluoropent-4-en-2-ol (**1s-1**) (0.71 g, 1.95 mmol, 1.0 eq.). The crude residue was purified by column chromatography (*n*-pentane) to yield the title compound as a colorless liquid (0.17 g, 0.84 mmol, 43%, *E*:*Z* = 1:1.6).

R*f* = 0.60 (*n*-pentane).

FT-IR ($\tilde{\nu}$ = cm^{-1})**:** 3093 (w), 1870 (w), 1651 (w), 1604 (w), 1559 (m), 1473 (w), 1425 (w), 1363 (m), 1278 (s), 1200 (m), 1174 (s), 1147 (m), 1112 (s), 1074 (m), 993 (m), 962 (m), 931 (m), 886 (m), 796 (m), 777 (m), 716 (s), 697 (m), 682 (m), 654 (m).

^1H NMR (500 MHz, $CDCl_3$):

E-diene: δ [ppm] = 6.74 – 6.64 (m, 1H, H-C8), 6.55 – 6.46 (m, 1H, H-C7), 5.51 – 5.40 (m, 2H, H-C9), 2.59 – 2.53 (m, 1H, H-C4), 1.85 – 1.78 (m, 2H, H-C2), 1.77 – 1.66 (m, 3H, H-C3, H-C1), 1.62 – 1.48 (m, 2H, H-C3), 1.41 – 1.11 (m, 3H, H-C3, H-C4).

Z-diene: δ [ppm] = 6.84 – 6.73 (m, 1H, H-C8), 6.20 (d, $^3J_{HH}$ = 11.4 Hz, 1H, H-C7), 5.40 – 5.28 (m, 2H, H-C9), 2.32 – 2.06 (m, 1H, H-C4), 1.85 – 1.78 (m, 4H, H-C2, H-C3), 1.44 – 1.09 (m, 5H, H-C2, H-C3, H-C4).

^{13}C NMR (126 MHz, $CDCl_3$):

Z-diene: δ [ppm] = 135.3 (q, $^2J_{CF}$ = 26.9 Hz, C5), 132.8 (q, $^3J_{CF}$ = 3.5 Hz, C7), 132.0 (q, $^4J_{CF}$ = 2.1 Hz, C8), 124.8 (q, $^1J_{CF}$ = 276.5 Hz, C6), 122.8 (q, $^5J_{CF}$ = 1.7 Hz, C9), 38.2 (C4), 33.1 (C3), 26.8 (C2), 26.1 (C1).

^{19}F NMR (470 MHz, $CDCl_3$): *E*-diene: δ [ppm] = -63.62 (s, 3F, F-C6); *Z*-diene: δ [ppm] = -58.83 (d, $^4J_{HF}$ = 2.3 Hz, 3F, F-C6).

^{19}F{^{1}H} NMR (470 MHz, $CDCl_3$): *E*-diene: δ [ppm] = -63.62 (s, 3F, F-C6); *Z*-diene: δ [ppm] = -58.83 (s, 3F, F-C6).

GC-EI-MS: (*m/z*) requires: $[(C_{11}H_{15}F_3)^+]$ = 204.1120, (*m/z*) found: $[(C_{11}H_{15}F_3)^+]$ = 204.1121.

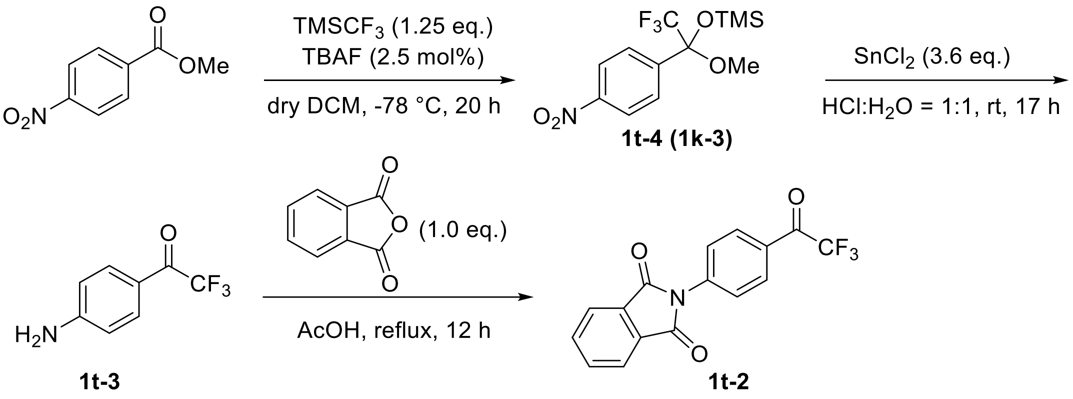

2-(4-(1,1,1-Trifluoro-2-hydroxypent-4-en-2-yl)phenyl)isoindoline-1,3-dione (1t-1)

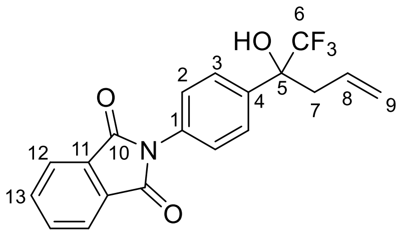

According to a modified literature procedure,[12] a solution of trimethyl(2,2,2-trifluoro-1-methoxy-1-(4-nitrophenyl) ethoxy)silane (**1t-4)** (4.40 g, 13.61 mmol, 1.0 eq.) in 10 mL ethanol was added to $SnCl_2$ (9.27 g, 48.90 mmol, 3.6 eq.) in 20.0 mL of aqueous HCl solution (1:1). After stirring for 17 h at room temperature, 2 M NaOH was added until pH = 10, and the aqueous layer was extracted with EtOAc (3 × 100 mL). The combined organic layers were dried over Na_2SO_4, filtered, and concentrated under reduced pressure. The crude residue was directly used in the next step without further purification.

To a solution of 1-(4-aminophenyl)-2,2,2-trifluoroethan-1-one (**1t-4**) (1.57 g, 8.30 mmol, 1.0 eq.) in AcOH (20 mL), phthalic anhydride (1.23 g, 8.30 mmol, 1.0 eq.) was added as a solid. The suspension was heated to reflux with stirring for 12 h. After completion, the reaction mixture was cooled to ambient temperature and diluted with water (20 mL). The obtained suspension was poured carefully into a saturated solution of $NaHCO_3$ (200 mL), and extracted with EtOAc (3 × 100 mL). The combined organic layers were dried over Na_2SO_4, filtered, and concentrated under reduced pressure. The crude residue was directly used in the next step

without further purification.

Compound **1t-1** was then prepared according to **General Procedure B** (**Method 2**) using 2-(4-(2,2,2-trifluoroacetyl)phenyl)isoindoline-1,3-dione (**1t-2**) (8.30 mmol, 1.0 eq.). The crude residue was purified by column chromatography (*n*-pentane:EtOAc 5:1) to yield the title compound as a white solid (1.75 g, 4.84 mmol, 58%).

R_f = 0.49 (*n*-pentane:EtOAc 10:1).

Melting Point: 186-187 ˚C.

FT-IR ($\tilde{\nu}$ = cm^{-1})**:** 3465 (m), 3015 (m), 2970 (m), 1739 (s), 1607 (w), 1515 (m), 1423 (m), 1372 (s), 1262 (m), 1228 (s), 1216 (s), 1156 (s), 1120 (m), 1082 (m), 1019 (m), 992 (m), 945 (m), 918 (m), 888 (m), 852 (w), 842 (m), 823 (m), 795 (m), 750 (w), 711 (m), 699 (m), 646 (w), 567 (w), 527 (m).

^{1}H NMR (500 MHz, DMSO-d_6): δ [ppm] = 8.01 – 7.95 (m, 2H, H-C12), 7.94 – 7.89 (m, 2H, H-C13), 7.78 – 7.67 (m, 2H, H-C3), 7.53 – 7.43 (m, 2H, H-C2), 6.73 (s, 1H, OH), 5.64 – 5.47 (m, 1H, H-C8), 5.17 (dq, $^{3}J_{HH}$ = 17.2 Hz, $^{6}J_{HF}$ = 1.5 Hz, 1H, H^a-C9), 5.03 (ddq, $^{3}J_{HH}$ = 10.2 Hz, $^{2}J_{HH}$ = 2.3 Hz, $^{6}J_{HH}$ = 1.1 Hz, 1H, H^b-C9), 3.06 (dd, $^{2}J_{HH}$ = 14.7 Hz, $^{3}J_{HH}$ = 7.5 Hz, 1H, H^a-C7), 2.84 (ddq, $^{2}J_{HH}$ = 14.6 Hz, $^{3}J_{HH}$ = 6.2 Hz, $^{4}J_{HF}$ = 1.5 Hz, 1H, H^b-C7).

^{13}C NMR (126 MHz, DMSO-d_6): δ [ppm] = 166.9 (C10), 136.8 (C4), 134.7 (C13), 131.6 (C1), 131.5 (C11), 131.2 (C8), 127.5 (C3), 126.6 (C2), 125.8 (q, $^{1}J_{CF}$ = 287.2 Hz, C6), 123.4 (C12), 119.5 (C9), 75.6 (q, $^{2}J_{CF}$ = 26.9 Hz, C5), 38.2 (C7).

^{19}F NMR (470 MHz, DMSO-d_6): δ [ppm] = -78.34 (s, 3F, F-C6).

^{19}F{^{1}H} NMR (470 MHz, DMSO-d_6): δ [ppm] = -78.34 (s, 3F, F-C6).

ESI-MS: (*m/z*) requires: [$(C_{19}H_{14}NF_3O_3Na)^+$] = 384.0818, (*m/z*) found: [$(C_{19}H_{14}NF_3O_3Na)^+$] = 384.0815.

2-(4-(1,1,1-Trifluoropenta-2,4-dien-2-yl)phenyl)isoindoline-1,3-dione (1t)

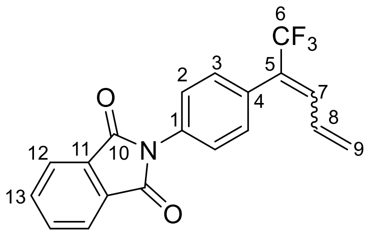

Compound **1t** was prepared according to **General Procedure C** 2-(4-(1,1,1-trifluoro-2-hydroxypent-4-en-2-yl)phenyl) isoindoline-1,3-dione (**1t-1**) (1.10 g, 3.04 mmol, 1.0 eq.). The crude residue was purified by column chromatography (*n*-pentane:EtOAc 10:1) to yield the title compound as a white solid (0.41 g, 1.19 mmol, 39%, *E*:*Z* = 2.3:1).

R*f* = 0.19 (*n*-pentane: EtOAc 10:1).

Melting Point: 152-154 ˚C.

FT-IR ($\tilde{\nu}$ = cm^{-1})**:** 3459 (w), 3015 (m), 2970 (m), 2946 (w), 1739 (s), 1609 (w), 1516 (w), 1422 (m), 1366 (s), 1270 (w), 1228 (s), 1216 (s), 1204 (s), 1167 (w), 1136 (w), 1105 (m), 1001 (w), 954 (w), 930 (m), 909 (w), 887 (w), 832 (m), 787 (w), 742 (w), 715 (m), 703 (m), 688 (m), 664 (w), 631 (w), 527 (m).

^{1}H NMR (500 MHz, $CDCl_3$):

E-diene: δ [ppm] = 8.01 – 7.90 (m, 2H, H-C12), 7.82 – 7.79 (m, 2H, H-C13), 7.58 – 7.51 (m, 2H, H-C2), 7.46 – 7.38 (m, 2H, H-C3), 7.00 – 6.81 (m, 1H, H-C7), 6.45 – 6.24 (m, 1H, H-C8), 5.75 – 5.57 (m, 1H, H^{a}-C9), 5.50 – 5.35 (m, 1H, H^{b}-C9).

Z-diene: δ [ppm] = 8.01 – 7.90 (m, 2H, H-C12), 7.82 – 7.79 (m, 2H, H-C13), 7.52 – 7.46 (m, 4H, H-C2, H-C3), 7.01 – 6.92 (m, 1H, H-C8), 6.58 (dq, $^{3}J_{HH}$ = 11.6 Hz, $^{4}J_{HF}$ = 0.8 Hz, 1H, H-C9), 5.60 – 5.51 (m, 2H, H-C11).

^{13}C NMR (126 MHz, $CDCl_3$):

E-diene: δ [ppm] = 167.1 (C10), 134.6 (C13), 134.3 (q, $^{3}J_{CF}$ = 5.9 Hz, C7), 132.2 (C1), 131.7 (C4), 131.7 (C11), 131.6 (C8), 130.7 (C3), 130.1 (q, $^{2}J_{CF}$ = 30.1 Hz, C5), 126.2 (C2), 125.4 (C9), 123.9 (C12), 123.6 (q, $^{1}J_{CF}$ = 272.7 Hz, C6).

^{19}F NMR (470 MHz, $CDCl_3$): *E*-diene: δ [ppm] = -64.96 (s, 3F, F-C6); *Z*-diene: δ [ppm] = -56.51 (d, $^{4}J_{HF}$ = 2.1 Hz, 3F, F-C6).

$^{19}F\{^{1}H\}$ NMR (470 MHz, $CDCl_3$): *E*-diene: δ [ppm] = -64.96 (s, 3F, F-C6); *Z*-diene: δ [ppm] = -56.51 (s, 3F, F-C6).

ESI-MS: (*m/z*) requires: $[(C_{19}H_{12}O_2NF_3Na)^+]$ = 366.0712, (*m/z*) found: $[(C_{19}H_{12}O_2NF_3Na)^+]$ = 366.0712.

3. 1,4-Difluorination reactions

3.1 Optimization of reaction conditions

Table S1 Optimization of 1,4-Difluorination reaction conditions. [a]

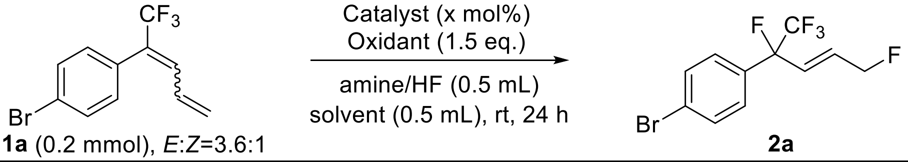

1a (0.2 mmol), *E*:*Z*=3.6:1 **2a**

Entry	Catalyst (x mol%)	Amine/HF ratio	Oxidant	Solvent	Conversion(%)	Yield(%)[b]
1	A (20%)	1 : 4.5	Selectfluor®	$CHCl_3$	<5	0
2	A (20%)	1 : 5.5	Selectfluor®	$CHCl_3$	50	40
3	A (20%)	1 : 6.5	Selectfluor®	$CHCl_3$	>95	88
4	A (20%)	1 : 7.5	Selectfluor®	$CHCl_3$	>95	94(83)[c]
5	A (20%)	1 : 8.5	Selectfluor®	$CHCl_3$	>95	90
6	A (20%)	1 : 9.2	Selectfluor®	$CHCl_3$	>95	88
7	B (20%)	1 : 7.5	Selectfluor®	$CHCl_3$	>95	92
8	C (20%)	1 : 7.5	Selectfluor®	$CHCl_3$	64	24
9	D (20%)	1 : 7.5	Selectfluor®	$CHCl_3$	58	49
10	E (20%)	1 : 7.5	Selectfluor®	$CHCl_3$	>95	57
11	F (20%)	1 : 7.5	Selectfluor®	$CHCl_3$	>95	46
12	G (20%)	1 : 7.5	Selectfluor®	$CHCl_3$	>95	88
13	A (20%)	1 : 7.5	Selectfluor®	DCM	>95	92
14	A (20%)	1 : 7.5	Selectfluor®	DCE	>95	91
15	A (20%)	1 : 7.5	Selectfluor®	*n*-heptane	>95	93
16	A (20%)	1 : 7.5	Selectfluor®	toluene	22	<5
17	A (20%)	1 : 7.5	*m*-CPBA	$CHCl_3$	>95	78
18	A (20%)	1 : 7.5	Oxone	$CHCl_3$	85	23
19	A (10%)	1 : 7.5	Selectfluor®	$CHCl_3$	>95	90
20	none	1 : 7.5	Selectfluor®	$CHCl_3$	10	0
21	A (20%)	1 : 7.5	none	$CHCl_3$	<5	0
22[d]	A (20%)	1 : 7.5	Selectfluor®	$CHCl_3$	<5	0
23[e]	A (20%)	1 : 7.5	Selectfluor®	$CHCl_3$	81	66

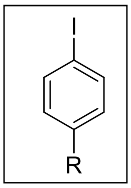

R = H, Catalyst A
R = Me, Catalyst B
R = OMe, Catalyst C
R = CO_2Me, Catalyst D

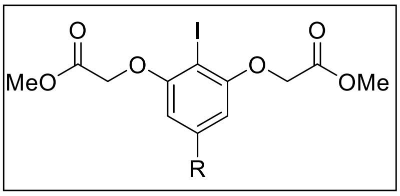

R = H, Catalyst E
R = Me, Catalyst F
R = CO_2Me, Catalyst G

[a] Reaction conditions: **1a** (0.2 mmol), catalyst (20 mol%), oxidant (1.5 eq.), amine:HF (0.5 mL) and solvent (0.5 mL). [b] Yields were determined by ^{19}F NMR analysis of the crude reaction mixture using trifluoro toluene as internal standard. [c] Isolated yield. [d] Reaction was performed at 0 °C. [e] Reaction was performed at 50 °C.

3.2 Catalytic 1,4-difluorination reactions

General procedure D for the 1,4-difluorination

Unless otherwise stated, a Teflon® vial was equipped with a 1 cm stirring bar followed by the addition of 1,3-diene (0.2 mmol, 1.0 eq.), iodobenzene (8.2 mg, 0.04 mmol, 20 mol%) and $CHCl_3$ (0.5 mL). A mixture of amine:HF 1:7.5 (0.5 mL, prepared from NEt_3•3HF and Olah´s reagent) was added via syringe. After stirring for 1 min, Selectfluor® (106 mg, 0.3 mmol, 1.5 eq.) was added in one portion. The reaction vessel was then sealed with a Teflon® screw cap. After stirring (350 rpm) at ambient temperature for 24 h, the reaction mixture was poured into 100 mL of a saturated solution of $NaHCO_3$ (CAUTION, generation of CO_2!). The Teflon® vial was rinsed with DCM and dropped into another flask of saturated aqueous solution of $NaHCO_3$ to guarantee the removal of excess HF. The organics were extracted with DCM (3x 30 mL), the combined organic layers were dried over Na_2SO_4 filtered and the solvent was carefully removed under reduced pressure. The yield was determined by ^{19}F NMR analysis of the crude reaction mixture using trifluoro toluene as an internal standard. The crude reaction mixture was purified by column chromatography to afford the desired product.

(*E*)-1-Bromo-4-(1,1,1,2,5-pentafluoropent-3-en-2-yl)benzene (2a)

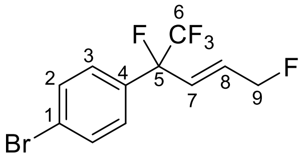

The reaction was performed according to **general procedure D** using 1-bromo-4-(1,1,1-trifluoropenta-2,4-dien-2-yl)benzene (**1a**, *E*:*Z* = 3.6:1) (55.3 mg, 0.2 mmol, 1.0 eq.). ^{19}F NMR analysis of the crude reaction mixture showed the formation of 94% 1,4-difluorinated product and <5% 1,2-difluorinated product. Column chromatography (*n*-pentane:DCM 150:1) afforded product **2a** as a colorless oil (52.3 mg, 0.17 mmol, 83%).

R*f* = 0.45 (*n*-pentane:DCM 10:1).

FT-IR ($\tilde{\nu}$ = cm^{-1})**:** 2938 (w), 1594 (w), 1490 (w), 1400 (w), 1386 (w), 1269 (m), 1221 (w), 1177 (s), 1162 (s), 1110 (m), 1188 (m), 1176 (m), 1011 (m), 953 (s), 941 (m), 915 (m), 823 (m), 795 (w), 768 (w), 730 (m), 684 (m).

^{1}H NMR (599 MHz, $CDCl_3$): δ [ppm] = 7.62 – 7.55 (m, 2H, H-C2), 7.42 – 7.35 (m, 2H, H-C3), 6.32 – 6.14 (m, 2H, H-C7, H-C8), 5.10 – 4.91 (m, 2H, H-C9).

^{13}C NMR (151 MHz, $CDCl_3$): δ [ppm] = 133.0 (d, $^{2}J_{CF}$ = 22.1 Hz, C4), 132.0 (d, $^{5}J_{CF}$ = 1.3 Hz, C2), 131.4 (dd, $^{2}J_{CF}$ = 16.6 Hz, $^{3}J_{CF}$ = 12.1 Hz, C8), 127.8 (dd, $^{3}J_{CF}$ = 9.1 Hz, $^{6}J_{CF}$ = 1.2 Hz, C3), 124.3 (d, $^{5}J_{CF}$ = 1.7 Hz, C1), 123.8 (dd, $^{2}J_{CF}$ = 18.3 Hz, $^{3}J_{CF}$ = 12.2 Hz, C7), 122.5 (dq, $^{1}J_{CF}$ = 285.4 Hz, $^{2}J_{CF}$ = 30.2 Hz, C6), 93.5 (dq, $^{1}J_{CF}$ = 190.3 Hz, $^{2}J_{CF}$ = 31.7 Hz, C5), 81.5 (d, $^{1}J_{CF}$ = 170.2 Hz, C9).

^{19}F NMR (564 MHz, $CDCl_3$): δ [ppm] = -79.43 (d, $^{3}J_{FF}$ = 8.5 Hz, 3F, F-C6), -168.67 (dqd, $^{3}J_{HF}$ = 17.0 Hz, $^{3}J_{FF}$ = 8.5 Hz, $^{5}J_{FF}$ = 2.7 Hz, 1F, F-C5), -221.19 – -221.54 (m, 1F, F-C9).

^{19}F{^{1}H} NMR (564 MHz, $CDCl_3$): δ [ppm] = -79.43 (d, $^{3}J_{FF}$ = 8.5 Hz, 3F, F-C6), -168.7 (dq, $^{3}J_{FF}$ = 8.5 Hz, $^{5}J_{FF}$ = 2.7 Hz, 1F, F-C5), -221.38 (d, $^{5}J_{FF}$ = 2.8 Hz, 1F, F-C9).

GC-EI-MS: (*m/z*) requires: [$(C_{11}H_8BrF_5)^+$] = 313.9724, (*m/z*) found: [$(C_{11}H_8BrF_5)^+$] = 313.9726.

(*E*)-1-Bromo-3-(1,1,1,2,5-pentafluoropent-3-en-2-yl)benzene (2b)

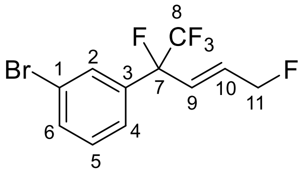

The reaction was performed according to **general procedure D** using 1-bromo-3-(1,1,1-trifluoropenta-2,4-dien-2-yl)benzene (**1b**, *E*:*Z* = 4:1) (55.4 mg, 0.2 mmol, 1.0 eq.). ^{19}F NMR analysis of the crude reaction mixture showed the formation of 90% 1,4-difluorinated product and 6% 1,2-difluorinated product. Column chromatography (*n*-pentane:DCM 150:1) afforded product **2b** as a colorless oil (51.8 mg, 0.16 mmol, 82%).

R_f = 0.45 (*n*-pentane:DCM 10:1).

FT-IR ($\tilde{\nu}$ = cm^{-1})**:** 2936 (w), 1571 (w), 1477 (w), 1423 (w), 1386 (w), 1269 (m), 1180 (s), 1164 (s), 1116 (m), 1088 (m), 1078 (m), 1020 (m), 997 (m), 964 (m), 920 (w), 888 (w), 785 (m), 732 (m), 717 (m), 693 (m).

^{1}H NMR (500 MHz, $CDCl_3$): δ [ppm] = 7.67 (dd, $^{4}J_{HH}$ = 1.9 Hz, $^{4}J_{HH}$ = 1.8 Hz, 1H, H-C2), 7.57 (ddd, $^{3}J_{HH}$ = 8.0 Hz, $^{4}J_{HH}$ = 1.9 Hz, $^{4}J_{HH}$ = 1.0 Hz, 1H, H-C6), 7.44 (ddt, $^{3}J_{HH}$ = 7.9 Hz,

$^{4}J_{HH}$ = 1.8 Hz, $^{4}J_{HH}$ = 0.9 Hz, 1H, H-C4), 7.32 (dd, $^{3}J_{HH}$ = 7.9 Hz, $^{3}J_{HH}$ = 8.0 Hz, 1H, H-C5), 6.33 – 6.14 (m, 2H, H-C9, H-C10), 5.13 – 4.89 (m, 2H, H-C11).

^{13}C NMR (126 MHz, $CDCl_3$): δ [ppm] = 135.1 (dd, $^{2}J_{CF}$ = 22.2 Hz, $^{5}J_{CF}$ = 0.9 Hz, C3), 132.0 (d, $^{5}J_{CF}$ = 1.2 Hz, C6), 130.7 (dd, $^{2}J_{CF}$ = 16.4 Hz, $^{3}J_{CF}$ = 11.1 Hz, C10), 129.3 (d, $^{4}J_{CF}$ = 1.5 Hz, C5), 128.2 (dq, $^{3}J_{CF}$ = 10.0 Hz, $^{4}J_{CF}$ = 1.3 Hz, C2), 123.7 (dq, $^{4}J_{CF}$ = 8.8 Hz, $^{5}J_{CF}$ = 1.4 Hz, C4), 122.7 (dd, $^{2}J_{CF}$ = 18.3 Hz, $^{3}J_{CF}$ = 12.3 Hz, C9), 121.9 (d, $^{4}J_{CF}$ = 1.8 Hz, C1), 121.5 dq, $^{1}J_{CF}$ = 286.0 Hz, $^{2}J_{CF}$ = 30.2 Hz, C8), 92.1 (dq, $^{1}J_{CF}$ = 191.5 Hz, $^{2}J_{CF}$ = 31.7 Hz, C7), 81.5 (d, $^{1}J_{CF}$ = 170.4 Hz, C11).

^{19}F NMR (470 MHz, $CDCl_3$): δ [ppm] = -79.28 (d, $^{3}J_{FF}$ = 8.3 Hz, 3F, F-C8), -169.02 (dqdd, $^{3}J_{HF}$ = 16.8 Hz, $^{3}J_{FF}$ = 8.4 Hz, $^{4}J_{HF}$ = 5.6 Hz, $^{5}J_{FF}$ = 2.6 Hz, 1F, F-C7), -221.24 – -221.61 (m, 1F, F-C11).

^{19}F{^{1}H} NMR (470 MHz, $CDCl_3$): δ [ppm] = -79.28 (d, $^{3}J_{FF}$ = 8.4 Hz, 3F, F-C8), -169.02 (dq, $^{3}J_{FF}$ = 8.4 Hz, $^{5}J_{FF}$ = 2.7 Hz, 1F, F-C7), -221.44 (d, $^{5}J_{FF}$ = 2.8 Hz, 1F, F-C11).

GC-EI-MS: (*m/z*) requires: [$(C_{11}H_8BrF_5)^+$] = 313.9724, (*m/z*) found: [$(C_{11}H_8BrF_5)^+$] = 313.9725.

(*E*)-1-Chloro-4-(1,1,1,2,5-pentafluoropent-3-en-2-yl)benzene (2c)

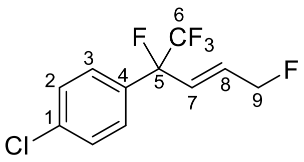

The reaction was performed according to **general procedure D** using 1-chloro-4-(1,1,1-trifluoropenta-2,4-dien-2-yl)benzene (**1c**, *E*:*Z* = 3.2:1) (46.1 mg, 0.2 mmol, 1.0 eq.). ^{19}F NMR analysis of the crude reaction mixture showed the formation of 87% 1,4-difluorinated product and < 5% 1,2-difluorinated product. Column chromatography (*n*-pentane:DCM 150:1) afforded product **2c** as a colorless oil (45.9 mg, 0.17 mmol, 86%).

R*f* = 0.45 (*n*-pentane:DCM 10:1).

FT-IR ($\tilde{\nu}$ = cm^{-1})**:** 2941 (w), 1600 (w), 1496 (m), 1456 (w), 1406 (w), 1385 (w), 1271 (m), 1221 (w), 1177 (s), 1162 (s), 1110 (m), 1014 (m), 954 (s), 942 (m), 915 (m), 828 (m), 796 (w), 772 (w), 737 (m), 729 (m), 692 (m).

^{1}H NMR (500 MHz, $CDCl_3$): δ [ppm] = 7.47 – 7.44 (m, 2H, H-C3), 7.42 – 7.35 (m, 2H, H-C2), 6.34 – 6.14 (m, 2H, H-C7, H-C8), 5.12 – 4.94 (m, 2H, H-C9).

^{13}C NMR (126 MHz, $CDCl_3$): δ [ppm] = 136.1 (d, $^{5}J_{CF}$ = 1.8 Hz, C1), 132.5 (dq, $^{2}J_{CF}$ = 22.3 Hz, $^{5}J_{CF}$ = 0.9 Hz, C4), 131.6 (dd, $^{2}J_{CF}$ = 16.4 Hz, $^{3}J_{CF}$ = 11.2 Hz, C8), 129.0 (d, $^{5}J_{CF}$ = 1.4 Hz, C2), 127.6 (dq, $^{2}J_{CF}$ = 9.1 Hz, $^{3}J_{CF}$ = 1.4 Hz, C3), 123.9 (dd, $^{2}J_{CF}$ = 18.2 Hz, $^{3}J_{CF}$ = 12.3 Hz, C7), 122.6 (dqd, $^{1}J_{CF}$ = 284.9 Hz, $^{2}J_{CF}$ = 30.9 Hz, $^{4}J_{CF}$ = 1.2 Hz, C6), 93.5 (dq, $^{1}J_{CF}$ = 189.6 Hz, $^{2}J_{CF}$ = 32.2 Hz, C5), 81.5 (d, $^{1}J_{CF}$ = 170.2 Hz, C9).

^{19}F NMR (470 MHz, $CDCl_3$): δ [ppm] = -79.45 (d, $^{3}J_{FF}$ = 8.4 Hz, 3F, F-C6), -168.43 (dqdd, $^{3}J_{HF}$ = 16.9 Hz, $^{4}J_{HF}$ = 13.8 Hz, $^{3}J_{FF}$ = 8.5 Hz, $^{5}J_{FF}$ = 3.6 Hz, 1F, F-C5), -221.22 – -221.54 (m, 1F, F-C9).

^{19}F{^{1}H} NMR (376 MHz, $CDCl_3$): δ [ppm] = -79.45 (d, $^{3}J_{FF}$ = 8.5 Hz, 3F, F-C6), -168.43 (dq, $^{3}J_{FF}$ = 9.2 Hz, $^{5}J_{FF}$ = 5.6 Hz, 1F, F-C5), -221.37 (d, $^{5}J_{FF}$ = 2.5 Hz, 1F, F-C9).

GC-EI-MS: (*m/z*) requires: $[(C_{11}H_8ClF_5)^+]$ = 270.0229, (*m/z*) found: $[(C_{11}H_8ClF_5)^+]$ = 270.0230.

(*E*)-1-Chloro-3-(1,1,1,2,5-pentafluoropent-3-en-2-yl)benzene (2d)

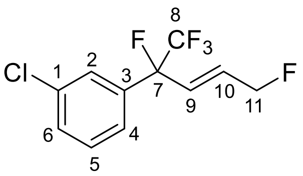

The reaction was performed according to **general procedure D** using 1-chloro-3-(1,1,1-trifluoropenta-2,4-dien-2-yl)benzene (**1d**, *E*:*Z* = 4.4:1) (46.3 mg, 0.2 mmol, 1.0 eq.). ^{19}F NMR analysis of the crude reaction mixture showed the formation of 84% 1,4-difluorinated product and 5% 1,2-difluorinated product. Column chromatography (*n*-pentane:DCM 150:1) afforded product **2d** as a colorless oil (39.4 mg, 0.15 mmol, 73%).

R*f* = 0.45 (*n*-pentane:DCM 10:1).

FT-IR ($\tilde{\nu}$ = cm^{-1})**:** 2938 (w), 1600 (w), 1578 (w), 1479 (w), 1472 (w), 1386 (w), 1270 (m), 1180 (s), 1163 (s), 1116 (m), 1085 (m), 1021 (m), 1000 (m), 967 (m), 920 (w), 888 (m), 784 (m), 740 (m), 724 (m), 705 (m), 686 (m).

^{1}H NMR (500 MHz, $CDCl_3$): δ [ppm] = 7.56-7.49 (m, 1H, H-C2), 7.47-7.35 (m, 3H, H-C4, H-C5, H-C6), 6.33 – 6.15 (m, 2H, H-C9, H-C10), 5.11 – 4.91 (m, 2H, H-C11).

^{13}C NMR (126 MHz, $CDCl_3$): δ [ppm] = 135.9 (dd, $^{2}J_{CF}$ = 22.2 Hz, $^{5}J_{CF}$ = 0.7 Hz, C3), 135.0 (d, $^{5}J_{CF}$ = 1.9 Hz, C6), 131.6 (ddq, $^{2}J_{CF}$ = 16.4 Hz, $^{3}J_{CF}$ = 11.2 Hz, $^{4}J_{CF}$ = 0.9 Hz, C10), 130.7 (d, $^{4}J_{CF}$ = 1.4 Hz, C1), 130.0 (d, $^{4}J_{CF}$ = 1.2 Hz, C5), 126.4 (dq, $^{3}J_{CF}$ = 10.1 Hz, $^{4}J_{CF}$ = 1.4 Hz, C2), 124.2 (dq, $^{3}J_{CF}$ = 8.9 Hz, $^{4}J_{CF}$ = 1.5 Hz, C4), 123.8 (dd, $^{2}J_{CF}$ = 18.3 Hz, $^{3}J_{CF}$ = 12.3 Hz, C9), 122.5 (dq, $^{1}J_{CF}$ = 285.0 Hz, $^{2}J_{CF}$ = 30.8 Hz, C8), 93.3 (dq, $^{1}J_{CF}$ = 190.7 Hz, $^{2}J_{CF}$ = 32.4 Hz, C7), 81.5 (d, $^{1}J_{CF}$ = 170.3 Hz, C11).

^{19}F NMR (470 MHz, $CDCl_3$): δ [ppm] = -79.30 (d, $^{3}J_{FF}$ = 8.4 Hz, 3F, F-C8), -168.99 (dqdt, $^{3}J_{HF}$ = 14.7 Hz, $^{3}J_{FF}$ = 8.5 Hz, $^{4}J_{HF}$ = 5.9 Hz, $^{5}J_{FF}$ = 3.2 Hz, 1F, F-C7), -221.25 – -221.58 (m, 1F, F-C11).

^{19}F{^{1}H} NMR (470 MHz, $CDCl_3$): δ [ppm] = -79.28 (d, $^{3}J_{FF}$ = 8.4 Hz, 3F, F-C8), -168.99 – -169.16 (m, 1F, F-C7), -221.4 (d, $^{5}J_{FF}$ = 2.2 Hz, 1F, F-C11).

GC-EI-MS: (*m/z*) requires: $[(C_{11}H_8ClF_5)^+]$ = 270.0229, (*m/z*) found: $[(C_{11}H_8ClF_5)^+]$ = 270.0230.

(*E*)-1,3-Dichloro-5-(1,1,1,2,5-pentafluoropent-3-en-2-yl)benzene (2e)

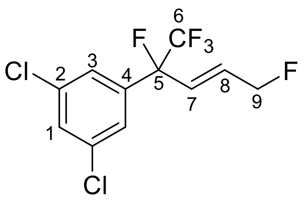

The reaction was performed according to **general procedure D** using 1,3-dichloro-5-(1,1,1-trifluoropenta-2,4-dien-2-yl)benzene (**1e**, *E*:*Z* = 4.7:1) (53.3 mg, 0.2 mmol, 1.0 eq.). ^{19}F NMR analysis of the crude reaction mixture showed the formation of 70% 1,4-difluorinated product and 25% 1,2-difluorinated product. Column chromatography (*n*-pentane:DCM 150:1) afforded product **2e** as a white solid (36.5 mg, 0.12 mmol, 60%) .

R*f* = 0.59 (*n*-pentane:DCM 10:1).

Melting Point: 45-46 ˚C.

FT-IR ($\tilde{\nu}$ = cm^{-1})**:** 3099 (w), 2952 (w), 1593 (w), 1572 (m), 1458 (w), 1423 (w), 1392 (w), 1288 (w), 1270 (m), 1226 (w), 1182 (m), 1163 (m), 1081 (m), 1029 (m), 998 (m), 986 (m), 965 (m), 919 (m), 885 (w), 862 (m), 803 (m), 782 (m), 740 (m), 691 (m), 674 (m).

^{1}H NMR (500 MHz, $CDCl_3$): δ [ppm] = 7.44 (t, $^4J_{HH}$ = 1.9 Hz, 1H, H-C1), 7.39 (dq, 2H, $^4J_{HH}$ = 1.9 Hz, $^5J_{HF}$ = 0.7 Hz, 2H, H-C3), 6.29 – 6.15 (m, 2H, H-C7, H-C8), 5.15 – 4.91 (m, 2H, H-C9).

^{13}C NMR (126 MHz, $CDCl_3$): δ [ppm] = 137.2 (d, $^2J_{CF}$ = 23.1 Hz, C4), 135.7 (d, $^5J_{CF}$ = 1.8 Hz, C2), 132.2 (dd, $^2J_{CF}$ = 16.4 Hz, $^3J_{CF}$ = 11.0 Hz, C7), 130.1 (d, $^5J_{CF}$ = 1.4 Hz, C1), 124.7 (dq, $^3J_{CF}$ = 9.7 Hz, $^4J_{CF}$ = 1.4 Hz, C3), 123.0 (dd, $^2J_{CF}$ = 18.2 Hz, $^3J_{CF}$ = 12.4 Hz, C8), 122.3 (dq, $^1J_{CF}$ = 285.1 Hz, $^2J_{CF}$ = 30.4 Hz, C6), 93.0 (dq, $^1J_{CF}$ = 192.2 Hz, $^2J_{CF}$ = 32.7 Hz, C5), 81.4 (d, $^1J_{CF}$ = 171.0 Hz, C9).

^{19}F NMR (470 MHz, $CDCl_3$): δ [ppm] = -79.22 (d, $^3J_{FF}$ = 8.3 Hz, 3F, F-C6), -168.97 (dddd, $^3J_{HF}$ = 16.4 Hz, $^4J_{HF}$ = 10.7 Hz, $^3J_{FF}$ = 8.1 Hz, $^5J_{FF}$ = 5.4 Hz, 1F, F-C5), -221.70 – -221.11 (m, 1F, F-C9).

^{19}F{^{1}H} NMR (376 MHz, $CDCl_3$): δ [ppm] = -79.22 (d, $^3J_{FF}$ = 8.3 Hz, 3F, F-C6), -168.97 (dq, $^3J_{FF}$ = 8.4 Hz, $^5J_{FF}$ = 2.5 Hz, 1F, F-C5), -221.91 (d, $^5J_{FF}$ = 2.5 Hz, 1F, F-C9).

GC-EI-MS: (*m/z*) requires: [$(C_{11}H_7Cl_2F_5)^+$] = 303.9840, (*m/z*) found: [$(C_{11}H_7Cl_2F_5)^+$] = 303.9842.

(*E*)-1-Fluoro-4-(1,1,1,2,5-pentafluoropent-3-en-2-yl)benzene (2f)

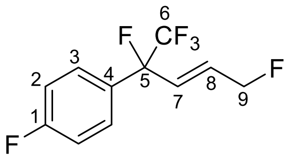

The reaction was performed according to **general procedure D** using 1-fluoro-4-(1,1,1-trifluoropenta-2,4-dien-2-yl)benzene (**1f**, *E*:*Z* = 2.9:1) (43.0 mg, 0.2 mmol, 1.0 eq.). ^{19}F NMR analysis of the crude reaction mixture showed the formation of 86% 1,4-difluorinated product and < 5% 1,2-difluorinated product. Column chromatography (*n*-pentane:DCM 150:1) afforded product **2f** as a colorless oil (36.2 mg, 0.14 mmol, 72%).

R*f* = 0.45 (*n*-pentane:DCM 10:1).

FT-IR ($\tilde{\nu}$ = cm^{-1})**:** 2943 (w), 1606 (w), 1513 (m), 1458 (w), 1415 (w), 1386 (w), 1302 (w), 1274 (m), 1340 (m), 1179 (s), 1164 (s), 1016 (m), 1100 (m), 1088 (m), 1014 (m), 955 (m), 939 (m), 915 (w), 835 (s), 818 (m), 794 (w), 763 (w), 729 (m), 700 (m).

^{1}H NMR (500 MHz, $CDCl_3$): δ [ppm] = 7.50 (dd, $^3J_{HH}$ = 8.7 Hz, $^4J_{HF}$ = 5.1 Hz, 2H, H-C3), 7.22 – 7.00 (m, 2H, H-C2), 6.42 – 6.10 (m, 2H, H-C7, H-C8), 5.19 – 4.84 (m, 2H, H-C9).

^{13}C NMR (126 MHz, $CDCl_3$): δ [ppm] = 163.5 (dd, $^1J_{CF}$ = 246.9 Hz, $^5J_{CF}$ = 1.7 Hz, C1), 131.3 (ddd, $^2J_{CF}$ = 16.5 Hz, $^3J_{CF}$ = 11.5 Hz, $^4J_{CF}$ = 0.9 Hz, C8), 129.9 (dd, $^2J_{CF}$ = 22.4 Hz, $^4J_{CF}$ = 3.5 Hz, C4), 128.3 (ddq, $^3J_{CF}$ = 8.7 Hz, $^3J_{CF}$ = 8.7 Hz, $^4J_{CF}$ = 1.4 Hz, C3), 124.2 (dd, $^2J_{CF}$ = 18.1 Hz, $^3J_{CF}$ = 12.3 Hz, C7), 122.7 (qdd, $^1J_{CF}$ = 285.0 Hz, $^2J_{CF}$ = 31.1 Hz, C6), 115.8 (dd, $^2J_{CF}$ = 21.9 Hz, $^4J_{CF}$ = 1.3 Hz, C2), 93.5 (dq, $^1J_{CF}$ = 189.6 Hz, $^2J_{CF}$ = 32.3 Hz, C5), 81.6 (d, $^1J_{CF}$ = 170.0 Hz, C9).

^{19}F NMR (470 MHz, $CDCl_3$): δ [ppm] = -79.45 (d, $^3J_{FF}$ = 8.5 Hz, 3F, F-C6), -111.53 (dddq, $^3J_{HF}$ = 13.4 Hz, $^4J_{HF}$ = 5.1 Hz, $^6J_{FF}$ = 8.3 Hz, $^7J_{FF}$ = 1.7 Hz, 1F, F-C1), -167.40 (ddq, $^3J_{HF}$ = 17.4 Hz, $^4J_{HF}$ = 2.4 Hz, $^3J_{FF}$ = 8.9 Hz, 1F, F-C5), -221.03 – -221.43 (m, 1F, F-C9).

^{19}F{^{1}H} NMR (376 MHz, $CDCl_3$): δ [ppm] = -79.45 (d, $^3J_{FF}$ = 8.5 Hz, 3F, F-C6), -111.53 (d, $^6J_{FF}$ = 1.6 Hz, 1F, F-C1), -168.43 (qdd, $^3J_{FF}$ = 8.6 Hz, $^5J_{FF}$ = 2.9 Hz, $^6J_{FF}$ = 1.6 Hz, 1F, F-C5), -221.24 (d, $^5J_{FF}$ = 2.8 Hz, 1F, F-C9).

GC-EI-MS: (*m/z*) requires: [$(C_{11}H_8F_6)^+$] = 254.0525, (*m/z*) found: [$(C_{11}H_8F_6)^+$] = 254.0526.

(*E*)-4-Fluoro-2-methyl-1-(1,1,1,2,5-pentafluoropent-3-en-2-yl)benzene (2g)

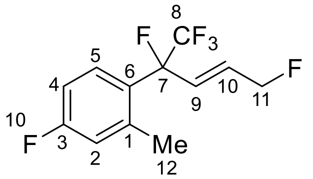

The reaction was performed according to **general procedure D** using 4-fluoro-2-methyl-1-(1,1,1-trifluoropenta-2,4-dien-2-yl) benzene (**1g**, *E*:*Z* = 1.3:1) (46.3 mg, 0.2 mmol, 1.0 eq.). ^{19}F NMR analysis of the crude reaction mixture showed the formation of 50% 1,4-difluorinated product and < 5% 1,2-difluorinated product. Column chromatography (*n*-pentane:DCM 100:1) afforded product **2g** as a colorless oil (26.3 mg, 0.10 mmol, 49%).

R$_f$ = 0.52 (*n*-pentane:DCM 10:1).

FT-IR ($\tilde{\nu}$ = cm^{-1})**:** 2970 (w), 1739 (m), 1594 (w), 1503 (m), 1425 (m), 1367 (m), 1280 (m), 1251 (m), 1202 (s), 1174 (s), 1136 (s), 1116 (s), 1020 (m), 994 (m), 932 (m), 879 (s), 843 (s), 788 (m), 765 (m), 743 (m), 710 (m), 663 (m), 628 (m), 603 (s), 574 (m), 536 (m), 509 (m).

^{1}H NMR (500 MHz, $CDCl_3$): δ [ppm] = 7.40 (ddd, $^{3}J_{HH}$ = 8.5 Hz, $^{4}J_{HF}$ = 4.0 Hz, $^{5}J_{HF}$ = 2.8 Hz, 1H, H-C5), 7.05 – 6.87 (m, 2H, H-C2, H-C4), 6.42 – 6.23 (m, 1H, H-C9), 6.17 – 5.97 (m, 1H, H-C10), 5.11 – 4.89 (m, 2H, H-C11), 2.44 (d, $^{5}J_{HF}$ = 4.8 Hz, 3H, H-C12).

^{13}C NMR (126 MHz, $CDCl_3$): δ [ppm] = 163.0 (dd, $^{1}J_{CF}$ = 249.7 Hz, $^{5}J_{CF}$ = 1.2 Hz, C3), 141.0 (d, $^{3}J_{CF}$ = 8.1 Hz, C1), 131.9 (dd, $^{2}J_{CF}$ = 16.7 Hz, $^{3}J_{CF}$ = 10.5 Hz, C10), 129.6 (ddq, $^{3}J_{CF}$ = 8.5 Hz, $^{3}J_{CF}$ = 8.5 Hz, $^{4}J_{CF}$ = 2.3 Hz, C5), 127.7 (dq, $^{2}J_{CF}$ = 21.2 Hz, $^{3}J_{CF}$ = 3.4 Hz, C6), 125.5 (dd, $^{2}J_{CF}$ = 20.6 Hz, $^{3}J_{CF}$ = 12.3 Hz, C9), 123.2 (qd, $^{1}J_{CF}$ = 285.5 Hz, $^{2}J_{CF}$ = 30.5 Hz, C8), 119.8 (dd, $^{2}J_{CF}$ = 21.2 Hz, $^{4}J_{CF}$ = 1.1 Hz, C2), 112.7 (dd, $^{2}J_{CF}$ = 21.2 Hz, $^{4}J_{CF}$ = 1.0 Hz, C4), 95.5 (dq, $^{1}J_{CF}$ = 188.6 Hz, $^{2}J_{CF}$ = 31.8 Hz, C7), 81.6 (d, $^{1}J_{CF}$ = 169.7 Hz, C11), 22.1 (dq, $^{4}J_{CF}$ = 8.9 Hz, $^{5}J_{CF}$ = 1.4 Hz, C12).

^{19}F NMR (470 MHz, $CDCl_3$): δ [ppm] = -77.58 (d, $^{3}J_{FF}$ = 8.5 Hz, 3F, F-C8), -112.65 (dddd, $^{3}J_{HF}$ = 9.6 Hz, $^{3}J_{HF}$ = 7.6 Hz, $^{4}J_{HF}$ = 5.6 Hz, $^{6}J_{FF}$ = 1.9 Hz, 1F, F-C3), -157.37 – -162.29 (m, 1F, F-C7), -220.87 (tddd, $^{2}J_{HF}$ = 46.2 Hz, $^{3}J_{HF}$ = 19.1 Hz, $^{4}J_{HF}$ = 3.0 Hz, $^{5}J_{FF}$ = 3.0 Hz, 1F, F-C11).

^{19}F{^{1}H} NMR (470 MHz, $CDCl_3$): δ [ppm] = -77.58 (d, $^{3}J_{FF}$ = 8.5 Hz, 3F, F-C8), -112.65 (d, $^{6}J_{FF}$ = 1.9 Hz, 1F, F-C3), -157.37 – -162.29 (m, 1F, F-C7), -220.87 (d, $^{5}J_{FF}$ = 3.4 Hz, 1F, F-C11).

GC-EI-MS: (*m/z*) requires: [$(C_{12}H_{10}F_6)^+$] = 268.0681, (*m/z*) found: [$(C_{12}H_{10}F_6)^+$] = 268.0682.

(*E*)-1-(1,1,1,2,5-Pentafluoropent-3-en-2-yl)-4-(trifluoromethyl)benzene (2h)

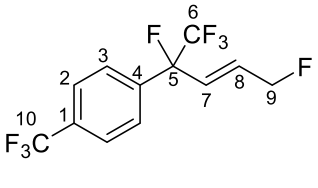

The reaction was performed according to **general procedure D** using 1-(trifluoromethyl)-4-(1,1,1-trifluoropenta-2,4-dien-2-yl) benzene (**1h**, *E*:*Z* = 6.4:1) (53.6 mg, 0.2 mmol, 1.0 eq.). ^{19}F NMR analysis of the crude reaction mixture showed the formation of 76% 1,4-difluorinated product and 20% 1,2-difluorinated product. Column chromatography

(n-pentane:DCM 150:1) afforded product **2h** as a colorless oil (42.8 mg, 0.14 mmol, 70%).

R*f* = 0.56 (n-pentane:DCM 10:1).

FT-IR ($\tilde{\nu}$ = cm^{-1})**:** 2943 (w), 1622 (w), 1458 (w), 1416 (w), 1387 (w), 1325 (s), 1274 (m), 1223 (w), 1166 (s), 1129 (s), 1111 (s), 1070 (s), 1017 (m), 959 (m), 950 (m), 916 (w), 841 (m), 800 (w), 759 (w), 712 (m), 700 (m), 675 (w), 653 (w).

^{1}H NMR (500 MHz, $CDCl_3$): δ [ppm] = 7.75 – 7.68 (m, 2H, H-C2), 7.65 (d, $^{3}J_{HH}$ = 7.6 Hz, 2H, H-C3), 6.40 – 6.11 (m, 2H, H-C7, H-C8), 5.31 – 4.85 (m, 2H, H-C9).

^{13}C NMR (126 MHz, $CDCl_3$): δ [ppm] = 137.8 (d, $^{2}J_{CF}$ = 22.2 Hz, C4), 132.1 (qd, $^{2}J_{CF}$ = 32.4 Hz, $^{5}J_{CF}$ = 1.2 Hz, C1), 131.9 (dd, $^{2}J_{CF}$ = 16.2 Hz, $^{3}J_{CF}$ = 11.3 Hz, C8), 126.6 (dq, $^{3}J_{CF}$ = 9.6 Hz, $^{4}J_{CF}$ = 1.4 Hz, C3), 125.8 (qd, $^{3}J_{CF}$ = 3.7 Hz, $^{4}J_{CF}$ = 1.6 Hz, C2), 123.8 (q, $^{1}J_{CF}$ = 272.4 Hz, C10), 123.6 (dd, $^{2}J_{CF}$ = 18.3 Hz, $^{3}J_{CF}$ = 12.3 Hz, C7), 122.5 (qd, $^{1}J_{CF}$ = 285.4 Hz, $^{2}J_{CF}$ = 30.0 Hz, C6), 93.5 (dq, $^{1}J_{CF}$ = 190.7 Hz, $^{2}J_{CF}$ = 32.4 Hz, C5), 81.4 (d, $^{1}J_{CF}$ = 170.6 Hz, C9).

^{19}F NMR (470 MHz, $CDCl_3$): δ [ppm] = -63.0 (s, 3F, F-C10), -79.28 (d, $^{3}J_{FF}$ = 8.4 Hz, 3F, F-C6), -169.43 (dqd, $^{3}J_{HF}$ = 16.9 Hz, $^{4}J_{HF}$ = 2.6 Hz, $^{3}J_{FF}$ = 8.2 Hz, 1F, F-C5), -221.52 – -221.94 (m, 1F, F-C9).

^{19}F{^{1}H} NMR (470 MHz, $CDCl_3$): δ [ppm] = -63.0 (s, 3F, F-C10), -79.28 (d, $^{3}J_{FF}$ = 8.4 Hz, 3F, F-C6), -168.43 (qd, $^{3}J_{FF}$ = 8.4 Hz, $^{5}J_{FF}$ = 2.7 Hz, 1F, F-C5), -221.73 (d, $^{5}J_{FF}$ = 2.8 Hz, 1F, F-C9).

GC-EI-MS: (m/z) requires: [$(C_8H_8F_8)^+$] = 304.0493, (m/z) found: [$(C_8H_8F_8)^+$] = 304.0496.

(*E*)-1-Methyl-4-(1,1,1,2,5-pentafluoropent-3-en-2-yl)benzene (2i)

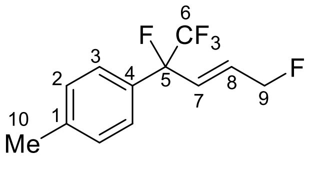

The reaction was performed according to **general procedure D** using 1-methyl-4-(1,1,1-trifluoropenta-2,4-dien-2-yl)benzene (**1i**, E:Z = 2.6:1) (42.2 mg, 0.2 mmol, 1.0 eq.). ^{19}F NMR analysis of the crude reaction mixture showed the formation of 41% 1,4-difluorinated product and 9% 1,2-difluorinated product. Column chromatography (n-pentane:DCM 100:1) afforded product **2i** as a colorless oil (20.1 mg, 0.08 mmol, 40%).

R$_f$ = 0.48 (*n*-pentane:DCM 10:1).

FT-IR ($\tilde{\nu}$ = cm^{-1})**:** 2930 (w), 1615 (w), 1516 (w), 1457 (w), 1382 (w), 1299 (w), 1273 (m), 1174 (s), 1111 (m), 1087 (m), 1013 (m), 955 (m), 942 (m), 914 (w), 816 (m), 753 (w), 724 (m), 701 (w).

^{1}H NMR (500 MHz, $CDCl_3$): δ [ppm] = 7.40 (d, $^{3}J_{HH}$ = 8.1 Hz, 2H, H-C3), 7.32 – 7.18 (m, 2H, H-C2), 6.49 – 6.07 (m, 2H, H-C7, H-C8), 5.25 – 4.81 (m, 2H, H-C9), 2.39 (s, 3H, H-C10).

^{13}C NMR (126 MHz, $CDCl_3$): δ [ppm] = 139.8 (d, $^{5}J_{CF}$ = 1.5 Hz, C1), 131.1 (dq, $^{2}J_{CF}$ = 22.2 Hz, $^{5}J_{CF}$ = 0.9 Hz, C4), 130.8 (dd, $^{2}J_{CF}$ = 16.4 Hz, $^{3}J_{CF}$ = 11.2 Hz, C8), 129.4 (d, $^{4}J_{CF}$ = 1.4 Hz, C2), 126.0 (dq, $^{3}J_{CF}$ = 8.9 Hz, $^{4}J_{CF}$ = 1.3 Hz, C3), 124.8 (dd, $^{2}J_{CF}$ = 18.2 Hz, $^{3}J_{CF}$ = 12.1 Hz, C7), 122.9 (qd, $^{1}J_{CF}$ = 285.2 Hz, $^{2}J_{CF}$ = 30.7 Hz, C6), 93.8 (dq, $^{1}J_{CF}$ = 188.7 Hz, $^{2}J_{CF}$ = 31.7 Hz, C5), 81.6 (d, $^{1}J_{CF}$ = 169.5 Hz, C9), 21.8 (C10).

^{19}F NMR (470 MHz, $CDCl_3$): δ [ppm] = -79.43 (d, $^{3}J_{FF}$ = 8.5 Hz, 3F, F-C6), -168.54 (dqd, $^{3}J_{HF}$ = 16.9 Hz, $^{4}J_{HF}$ = 2.3 Hz, $^{3}J_{FF}$ = 8.3 Hz, 1F, F-C5), -220.48 – -220.91 (m, 1F, F-C9).

^{19}F{^{1}H} NMR (376 MHz, $CDCl_3$): δ [ppm] = -79.43 (d, $^{3}J_{FF}$ = 8.5 Hz, 3F, F-C6), -168.54 (qd, $^{3}J_{FF}$ = 8.6 Hz, $^{5}J_{FF}$ = 2.9 Hz, 1F, F-C5), -220.71 (d, $^{5}J_{FF}$ = 2.9 Hz, 1F, F-C9).

GC-EI-MS: (*m/z*) requires: [$(C_{12}H_{11}F_5)^+$] = 250.0775, (*m/z*) found: [$(C_{12}H_{11}F_5)^+$] = 250.0777.

(*E*)-(1,1,1,2,5-Pentafluoropent-3-en-2-yl)benzene (2j)

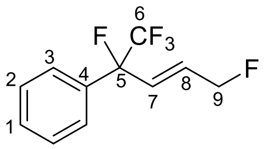

The reaction was performed according to **general procedure D** using (1,1,1-trifluoropenta-2,4-dien-2-yl)benzene (**1j**, *E*:*Z* = 3:1) (40.1 mg, 0.2 mmol, 1.0 eq.). ^{19}F NMR analysis of the crude reaction mixture showed the formation of 74% 1,4-difluorinated product and < 5% 1,2-difluorinated product. Column chromatography (*n*-pentane:DCM 100:1) afforded product **2j** as a colorless oil (33.9 mg, 0.14 mmol, 72%).

R$_f$ = 0.50 (*n*-pentane:DCM 10:1).

FT-IR ($\tilde{\nu}$ = cm^{-1})**:** 3458 (w), 3066 (w), 3026 (m), 3016 (m), 3005 (m), 2970 (m), 2946 (m), 1739 (s), 1452 (m), 1366 (s), 1296 (w), 1275 (m), 1228 (s), 1217 (s), 1203 (s), 1176 (s), 1112 (m), 1088 (m), 1015 (m), 1003 (m), 953 (m), 910 (m), 849 (w), 823 (w), 798 (w), 769 (m), 755 (m), 703 (m), 653 (m), 626 (w), 601 (m), 568 (w), 539 (m), 527 (m), 515 (m).

^{1}H NMR (599 MHz, $CDCl_3$): δ [ppm] = 7.54 – 7.49 (m, 2H, H-C3), 7.47 – 7.40 (m, 3H, H-C1, H-C2), 6.37 – 6.27 (m, 1H, H-C7), 6.26 – 6.14 (m, 1H, H-C8), 5.09 – 4.92 (m, 2H, H-C9).

^{13}C NMR (151 MHz, $CDCl_3$): δ [ppm] = 134.0 (d, $^2J_{CF}$ = 22.1 Hz, C4), 131.0 (dd, $^2J_{CF}$ = 16.4 Hz, $^3J_{CF}$ = 11.3 Hz, C8), 129.7 (d, $^5J_{CF}$ = 1.4 Hz, C1), 128.7 (d, $^4J_{CF}$ = 1.5 Hz, C2), 126.0 (dq, $^3J_{CF}$ = 9.1 Hz, $^4J_{CF}$ = 1.3 Hz, C3), 124.6 (dd, $^2J_{CF}$ = 18.3 Hz, $^3J_{CF}$ = 12.2 Hz, C7), 122.9 (qd, $^1J_{CF}$ = 285.4 Hz, $^2J_{CF}$ = 30.9 Hz, C6), 93.8 (dq, $^1J_{CF}$ = 189.1 Hz, $^2J_{CF}$ = 32.1 Hz, C5), 81.6 (d, $^1J_{CF}$ = 169.5 Hz, C9).

^{19}F NMR (564 MHz, $CDCl_3$): δ [ppm] = -79.34 (d, $^3J_{FF}$ = 8.4 Hz, 3F, F-C6), -169.11 (dqd, $^3J_{HF}$ = 17.2 Hz, $^3J_{FF}$ = 8.5 Hz, $^4J_{HF}$ = 2.6 Hz, 1F, F-C5), -220.89 (tddd, $^2J_{HF}$ = 46.2 Hz, $^3J_{HF}$ = 19.1 Hz, $^4J_{HF}$ = 2.6 Hz, $^5J_{FF}$ = 2.6 Hz, 1F, F-C9).

^{19}F{^{1}H} NMR (376 MHz, $CDCl_3$): δ [ppm] = -79.34 (d, $^3J_{FF}$ = 8.5 Hz, 3F, F-C6), -169.11 (qd, $^3J_{FF}$ = 9.7 Hz, $^5J_{FF}$ = 2.6 Hz, 1F, F-C5), -220.95 (d, $^5J_{FF}$ = 2.5 Hz, 1F, F-C9).

GC-EI-MS: (*m/z*) requires: [$(C_{11}H_9F_5)^+$] = 236.0619, (*m/z*) found: [$(C_{11}H_9F_5)^+$] = 236.0618.

(*E*)-1-Nitro-4-(1,1,1,2,5-pentafluoropent-3-en-2-yl)benzene (2k)

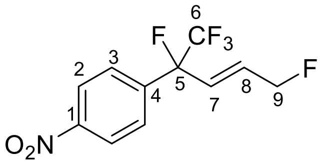

The reaction was performed according to **general procedure D** using 1-nitro-4-(1,1,1-trifluoropenta-2,4-dien-2-yl)benzene (**1k**, *E*:*Z* = 14.3:1) (48.0 mg, 0.2 mmol, 1.0 eq.). ^{19}F NMR analysis of the crude reaction mixture showed the formation of 57% 1,4-difluorinated product and 31% 1,2-difluorinated product. Column chromatography (*n*-pentane: DCM 5:1) afforded product **2k** as a colorless liquid (26.8 mg, 0.10 mmol, 48%).

R$_f$ = 0.31 (*n*-pentane: EtOAc 10:1).

FT-IR ($\tilde{\nu}$ = cm^{-1})**:** 3458 (w), 3004 (m), 2970 (m), 2946 (m), 1739 (s), 1611 (w), 1525 (m), 1498 (w), 1454 (w), 1351 (s), 1307 (w), 1272 (m), 1227 (m), 1217 (m), 1182 (s), 1110 (m), 1088 (m), 1015 (m), 988 (m), 952 (m), 915 (m), 893 (m), 851 (s), 797 (m), 770 (m), 752 (m), 721 (m), 712 (m), 695 (m), 626 (m), 602 (m), 568 (w), 527 (m).

^{1}H NMR (500 MHz, $CDCl_3$): δ [ppm] = 8.48 – 8.18 (m, 2H, H-C2), 7.88 – 7.60 (m, 2H, H-C3), 6.43 – 6.09 (m, 2H, H-C7, H-C8), 5.24 – 4.87 (m, 2H, H-C9).

^{13}C NMR (126 MHz, $CDCl_3$): δ [ppm] = 148.8 (C1), 140.5 (d, $^{2}J_{CF}$ = 21.7 Hz, C4), 132.4 (dd, $^{2}J_{CF}$ = 16.3 Hz, $^{3}J_{CF}$ = 11.2 Hz, C7), 127.3 (dq, $^{3}J_{CF}$ = 9.5 Hz, $^{4}J_{CF}$ = 1.4 Hz, C3), 123.9 (d, $^{4}J_{CF}$ = 1.7 Hz, C2), 122.8 (dd, $^{2}J_{CF}$ = 18.2 Hz, $^{3}J_{CF}$ = 12.4 Hz, C8), 122.3 (qd, $^{1}J_{CF}$ = 285.4 Hz, $^{2}J_{CF}$ = 31.0 Hz, C6), 93.5 (dq, $^{1}J_{CF}$ = 191.9 Hz, $^{2}J_{CF}$ = 32.6 Hz, C5), 81.3 (d, $^{1}J_{CF}$ = 171.2 Hz, C9).

^{19}F NMR (470 MHz, $CDCl_3$): δ [ppm] = -79.10 (d, $^{3}J_{FF}$ = 8.4 Hz, 3F, F-C6), -169.24 (dqd, $^{3}J_{HF}$ = 16.4 Hz, $^{3}J_{FF}$ = 8.1 Hz, $^{4}J_{HF}$ = 4.4 Hz, 1F, F-C5), -217.34 – -225.58 (m, 1F, F-C9).

^{19}F{^{1}H} NMR (470 MHz, $CDCl_3$): δ [ppm] = -79.10 (d, $^{3}J_{FF}$ = 8.4 Hz, 3F, F-C6), -169.24 (dq, $^{3}J_{FF}$ = 8.4 Hz, $^{5}J_{FF}$ = 2.7 Hz, 1F, F-C5), -215.53 – -226.28 (m, 1F, F-C9).

ESI-MS: (*m/z*) requires: [($C_{11}H_8O_2NF_5$-H)$^-$] = 280.0402, (*m/z*) found: [($C_{11}H_8O_2NF_5$-H)$^-$] = 280.0401.

(*E*)-4-(1,1,1,2,5-Pentafluoropent-3-en-2-yl)benzonitrile (2l)

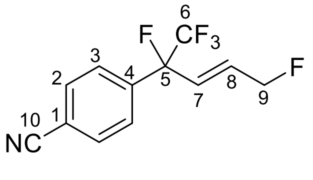

The reaction was performed according to **general procedure D** using 4-(1,1,1-trifluoropenta-2,4-dien-2-yl)benzonitrile (**1l**, *E*:*Z* = 5.7:1) (44.3 mg, 0.2 mmol, 1.0 eq.). ^{19}F NMR analysis of the crude reaction mixture showed the formation of 60% 1,4-difluorinated product and < 5% 1,2-difluorinated product. Column chromatography (*n*-pentane:Et_2O 30:1) afforded product **2l** as a colorless oil (24.5 mg, 0.09 mmol, 47%).

R$_f$ = 0.23 (*n*-pentane: Et_2O 20:1).

FT-IR ($\tilde{\nu}$ = cm^{-1})**:** 2952 (w), 2234 (w), 1613 (w), 1508 (w), 1456 (w), 1412 (w), 1386 (w), 1270 (m), 1178 (s), 1164 (s), 1113 (m), 1087 (m), 1017 (m), 989 (m), 950 (m), 915 (m), 837 (m), 804 (w), 746 (w), 735 (w), 699 (m).

^{1}H NMR (500 MHz, $CDCl_3$): δ [ppm] = 7.79 – 7.70 (m, 2H, H-C2), 7.68 – 7.59 (m, 2H, H-C3), 6.35 – 6.11 (m, 2H, H-C7, H-C8), 5.13 – 4.89 (m, 2H, H-C9).

^{13}C NMR (126 MHz, $CDCl_3$): δ [ppm] = 138.8 (d, $^{2}J_{CF}$ = 22.4 Hz, C4), 132.6 (d, $^{4}J_{CF}$ = 1.8 Hz, C2), 132.4 (dd, $^{2}J_{CF}$ = 16.2 Hz, $^{3}J_{CF}$ = 11.2 Hz, C8), 126.9 (dq, $^{3}J_{CF}$ = 9.5 Hz, $^{4}J_{CF}$ = 1.4 Hz, C3), 122.9 (dd, $^{2}J_{CF}$ = 18.1 Hz, $^{3}J_{CF}$ = 12.3 Hz, C7), 122.3 (qd, $^{1}J_{CF}$ = 285.6 Hz, $^{2}J_{CF}$ = 30.4 Hz, C6), 118.0 (C10), 114.0 (d, $^{5}J_{CF}$ = 1.5 Hz, C1), 93.4 (dq, $^{1}J_{CF}$ = 191.5 Hz, $^{2}J_{CF}$ = 32.5 Hz, C5), 81.3 (d, $^{1}J_{CF}$ = 171.0 Hz, C9).

^{19}F NMR (470 MHz, $CDCl_3$): δ [ppm] = -79.16 (d, $^{3}J_{FF}$ = 8.3 Hz, 3F, F-C6), -169.87 (dqdd, $^{3}J_{HF}$ = 13.9 Hz, $^{3}J_{FF}$ = 8.4 Hz, $^{4}J_{HF}$ = 5.2 Hz, $^{5}J_{FF}$ = 2.8 Hz, 1F, F-C5), -221.79 – -222.26 (m, 1F, F-C9).

^{19}F{^{1}H} NMR (376 MHz, $CDCl_3$): δ [ppm] = -79.16 (d, $^{3}J_{FF}$ = 8.3 Hz, 3F, F-C6), -164.21 – -173.88 (m, 1F, F-C5), -220.02 (d, $^{5}J_{FF}$ = 2.8 Hz, 1F, F-C9).

GC-EI-MS: (*m/z*) requires: [$(C_{12}H_8F_5N)^+$] = 261.0571, (*m/z*) found: [$(C_{12}H_8F_5N)^+$] = 261.0567.

Methyl (*E*)-4-(1,1,1,2,5-pentafluoropent-3-en-2-yl)benzoate (2m)

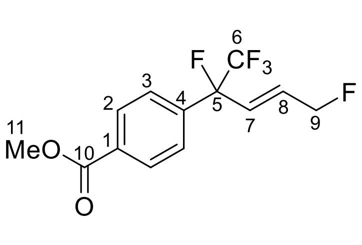

The reaction was performed according to **general procedure D** using methyl 4-(1,1,1-trifluoropenta-2,4-dien-2-yl)benzoate (**1m**, *E*:*Z* = 14.2:1) (51.0 mg, 0.2 mmol, 1.0 eq.). ^{19}F NMR analysis of the crude reaction mixture showed the formation of 85% 1,4-difluorinated product and 13% 1,2-difluorinated product. Column chromatography (*n*-pentane:Et_2O 20:1) afforded product **2m** as a colorless oil (44.9 mg, 0.15 mmol, 77%).

R*f* = 0.45 (*n*-pentane: EtOAc 10:1).

FT-IR ($\tilde{\nu}$ = cm^{-1})**:** 3459 (w), 3004 (m), 2970 (m), 1737 (s), 1438 (m), 1413 (m), 1366 (s), 1279 (s), 1228 (s), 1217 (s), 1202 (s), 1109 (s), 1089 (m), 1016 (m), 951 (m), 914 (m), 857 (m), 827 (m), 774 (m), 762 (m), 716 (m), 686 (w), 625 (m), 602 (m), 568 (w), 526 (m).

^{1}H NMR (500 MHz, $CDCl_3$): δ [ppm] = 8.19 – 7.95 (m, 2H, H-C2), 7.59 (d, $^{3}J_{HH}$ = 8.4 Hz, 2H, H-C3), 6.48 – 6.05 (m, 2H, H-C7, H-C8), 5.22 – 4.82 (m, 2H, H-C9), 3.94 (s, 3H, H-C11).

^{13}C NMR (126 MHz, $CDCl_3$): δ [ppm] = 166.4 (C10), 138.5 (dd, $^{2}J_{CF}$ = 21.9 Hz, $^{3}J_{CF}$ = 0.9 Hz, C4), 131.7 (dd, $^{2}J_{CF}$ = 16.4 Hz, $^{3}J_{CF}$ = 11.3 Hz, C8), 131.5 (d, $^{5}J_{CF}$ = 1.3 Hz, C1), 129.9 (d, $^{4}J_{CF}$ = 1.6 Hz, C2), 126.1 (dq, $^{3}J_{CF}$ = 9.4 Hz, $^{4}J_{CF}$ = 1.4 Hz, C3), 123.8 (dd, $^{2}J_{CF}$ = 18.4 Hz, $^{3}J_{CF}$ = 12.2 Hz, C7), 122.5 (qd, $^{1}J_{CF}$ = 284.2 Hz, $^{2}J_{CF}$ = 30.9 Hz, C6), 93.7 (dq, $^{1}J_{CF}$ = 190.5 Hz, $^{2}J_{CF}$ = 32.4 Hz, C5), 81.5 (d, $^{1}J_{CF}$ = 170.3 Hz, C9), 52.5 (C11).

^{19}F NMR (470 MHz, $CDCl_3$): δ [ppm] = -79.20 (d, $^{3}J_{FF}$ = 8.5 Hz, 3F, F-C6), -169.51 (dqdd, $^{3}J_{HF}$ = 16.9 Hz, $^{3}J_{FF}$ = 8.1 Hz, $^{4}J_{HF}$ = 14.4 Hz, $^{5}J_{FF}$ = 2.5 Hz, 1F, F-C5), -211.45 – -227.86 (m, 1F, F-C9).

^{19}F{^{1}H} NMR (376 MHz, $CDCl_3$): δ [ppm] = -79.20 (d, $^{3}J_{FF}$ = 8.5 Hz, 3F, F-C6), -169.51 (dq, $^{3}J_{FF}$ = 8.4 Hz, $^{5}J_{FF}$ = 2.7 Hz, 1F, F-C5), -221.46 (d, $^{5}J_{FF}$ = 2.8 Hz, 1F, F-C9).

ESI-MS: (*m/z*) requires: [$(C_{13}H_{11}O_2F_5Na)^+$] = 317.0571, (*m/z*) found: [$(C_{13}H_{11}O_2F_5Na)^+$] = 317.0572.

(*E*)-1-(Methylsulfonyl)-4-(1,1,1,2,5-pentafluoropent-3-en-2-yl)benzene (2n)

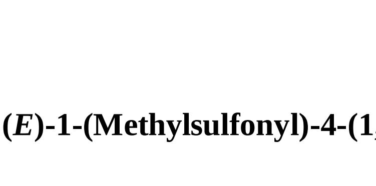

The reaction was performed according to **general procedure D** using (*E*)-1-(methylsulfonyl)-4-(1,1,1-trifluoropenta-2,4-dien-2-yl)benzene (**1n**, pure *E*) (55.1 mg, 0.2 mmol, 1.0 eq.). ^{19}F NMR analysis of the crude reaction mixture showed the formation of 65% 1,4-difluorinated product and 32% 1,2-difluorinated product. Column chromatography (*n*-pentane:EtOAc 4:1) afforded product **2n** as a colorless oil (36.3 mg, 0.12 mmol, 58%).

R$_f$ = 0.24 (*n*-pentane: EtOAc 3:1).

FT-IR ($\tilde{\nu}$ = cm^{-1})**:** 2933 (w), 1601 (w), 1405 (w), 1385 (w), 1307 (m), 1272 (m), 1181 (s), 1151 (s), 1116 (m), 1190 (m), 1015 (m), 987 (m), 947 (s), 914 (m), 838 (m), 778 (m), 760 (m), 731 (m), 692 (m).

^{1}H NMR (500 MHz, $CDCl_3$): δ [ppm] = 8.14 – 7.89 (m, 2H, H-C2), 7.81 – 7.66 (m, 2H, H-C3), 6.44 – 6.08 (m, 2H, H-C7, H-C8), 5.18 – 4.84 (m, 2H, H-C9), 3.08 (s, 3H, H-C10).

^{13}C NMR (126 MHz, $CDCl_3$): δ [ppm] = 142.0 (d, $^5J_{CF}$ = 1.3 Hz, C1), 139.8 (d, $^2J_{CF}$ = 21.7 Hz, C4), 132.3 (dd, $^2J_{CF}$ = 16.3 Hz, $^3J_{CF}$ = 11.1 Hz, C8), 127.9 (d, $^4J_{CF}$ = 1.7 Hz, C2), 127.2 (dq, $^3J_{CF}$ = 9.5 Hz, $^4J_{CF}$ = 1.3 Hz, C3), 123.0 (dd, $^2J_{CF}$ = 18.3 Hz, $^3J_{CF}$ = 12.4 Hz, C7), 122.4 (qd, $^1J_{CF}$ = 285.2 Hz, $^2J_{CF}$ = 29.9 Hz, C6), 93.5 (dq, $^1J_{CF}$ = 191.6 Hz, $^2J_{CF}$ = 32.5 Hz, C5), 81.3 (d, $^1J_{CF}$ = 170.9 Hz, C9), 44.5 (C10).

^{19}F NMR (470 MHz, $CDCl_3$): δ [ppm] = -79.09 (d, $^3J_{FF}$ = 8.3 Hz, 3F, F-C6), -165.08 – -174.63 (m, 1F, F-C5), -216.31 – -228.12 (m, 1F, F-C9).

^{19}F{^{1}H} NMR (376 MHz, $CDCl_3$): δ [ppm] = -79.09 (d, $^3J_{FF}$ = 8.4 Hz, 3F, F-C6), -169.44 (dq, $^3J_{FF}$ = 8.4 Hz, $^5J_{FF}$ = 2.7 Hz, 1F, F-C5), -213.71 – -230.20 (m, 1F, F-C9).

ESI-MS: (*m/z*) requires: [$(C_{12}H_{11}O_2SF_5Na)^+$] = 337.0292, (*m/z*) found: [$(C_{12}H_{11}O_2SF_5Na)^+$] = 337.0291.

(*E*)-3-(1,1,1,2,5-Pentafluoropent-3-en-2-yl)phenyl 4-methylbenzenesulfonate (2o)

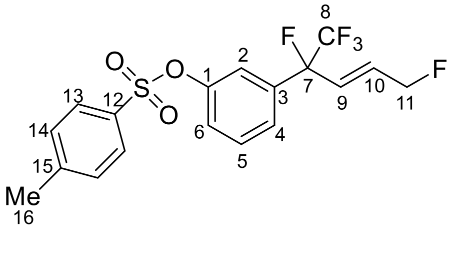

The reaction was performed according to **general procedure D** using 3-(1,1,1-trifluoropenta-2,4-dien-2-yl) phenyl 4-methylbenzenesulfonate (**1o**, *E*:*Z* = 6:1) (69.3 mg, 0.19 mmol, 1.0 eq.). ^{19}F NMR analysis of the crude reaction mixture showed the formation of 86% 1,4-difluorinated product and 8% 1,2-difluorinated product. Column chromatography (*n*-pentane:DCM 2:1) afforded product **2o** as a white solid (62.3 mg, 0.15 mmol, 81%).

R$_f$ = 0.31 (*n*-pentane: EtOAc 10:1).

Melting Point: 58-59 ˚C.

FT-IR ($\tilde{\nu}$ = cm^{-1})**:** 3458 (w), 3015 (m), 2970 (m), 2949 (m), 3458 (w), 1739 (s), 1596 (w), 1585 (m), 1487 (m), 1439 (m), 1370 (s), 1286 (m), 1269 (m), 1228 (s), 1215 (s), 1179 (m), 1158 (m), 1144 (m), 1092 (m), 1075 (m), 1014 (m), 975 (m), 917 (m), 892 (m), 868 (m), 805 (m), 793 (m), 771 (m), 741 (m), 721 (m), 691 (m), 660 (m), 643 (m), 597 (w), 550 (s), 529 (s).

^{1}H NMR (500 MHz, $CDCl_3$): δ [ppm] = 7.76 – 7.61 (m, 2H, H-C13), 7.43 – 7.36 (m, 2H, H-C4, H-C5), 7.34 – 7.28 (m, 2H, H-C14), 7.18 (ddd, $^{3}J_{HH}$ = 6.4 Hz, $^{4}J_{HH}$ = 3.1 Hz, $^{4}J_{HH}$ = 2.4 Hz, 1H, H-C6), 6.97 (dd, $^{4}J_{HH}$ = 2.9 Hz, $^{4}J_{HH}$ = 1.1 Hz, 1H, H-C2), 6.24 – 5.98 (m, 2H, H-C9, H-C10), 5.17 – 4.83 (m, 2H, H-C11), 2.44 (s, 3H, H-C16).

^{13}C NMR (126 MHz, $CDCl_3$): δ [ppm] = 149.8 (d, $^{4}J_{CF}$ = 1.9 Hz, C1), 145.9 (C15), 135.7 (d, $^{2}J_{CF}$ = 22.9 Hz, C3), 132.0 (C12), 131.8 (dd, $^{2}J_{CF}$ = 16.4 Hz, $^{3}J_{CF}$ = 11.1 Hz, C10), 130.1 (d, $^{4}J_{CF}$ = 1.4 Hz, C5), 130.0 (C14), 128.7 (C13), 124.7 (dq, $^{3}J_{CF}$ = 9.3 Hz, $^{4}J_{CF}$ = 1.3 Hz, C4), 124.1 (d, $^{5}J_{CF}$ = 1.1 Hz, C6), 123.4 (dd, $^{2}J_{CF}$ = 18.3 Hz, $^{3}J_{CF}$ = 12.3 Hz, C9), 127.4 (dq, $^{1}J_{CF}$ = 285.3 Hz, $^{2}J_{CF}$ = 30.8 Hz, C8), 120.4 (dq, $^{3}J_{CF}$ = 9.9 Hz, $^{4}J_{CF}$ = 1.4 Hz, C2), 93.1 (dq, $^{1}J_{CF}$ = 190.7 Hz, $^{2}J_{CF}$ = 32.3 Hz, C7), 81.4 (d, $^{1}J_{CF}$ = 170.4 Hz, C11), 21.7 (C16).

^{19}F NMR (470 MHz, $CDCl_3$): δ [ppm] = -79.42 (d, $^{3}J_{FF}$ = 8.4 Hz, 3F, F-C8), -168.66 (dqdd, $^{3}J_{HF}$ = 11.1 Hz, $^{3}J_{FF}$ = 8.1 Hz, $^{4}J_{HF}$ = 5.1 Hz, $^{5}J_{FF}$ = 2.7 Hz, 1F, F-C7), -210.93 – -229.42 (m, 1F, F-C11).

^{19}F{^{1}H} NMR (470 MHz, $CDCl_3$): δ [ppm] = -79.42 (d, $^{3}J_{FF}$ = 8.4 Hz, 3F, F-C8), -168.66 (qd, $^{3}J_{FF}$ = 8.4 Hz, $^{5}J_{FF}$ = 2.7 Hz, 1F, F-C7), -221.45 (d, $^{5}J_{FF}$ = 2.7 Hz, 1F, F-C11).

ESI-MS: (*m/z*) requires: [$(C_{18}H_{15}O_3SF_5Na)^+$] = 429.0554, (*m/z*) found: [$(C_{18}H_{15}O_3SF_5Na)^+$] = 429.0548.

(*E*)-1-(1,1,1,2,5-Pentafluoropent-3-en-2-yl)-3-(trifluoromethoxy)benzene (2p)

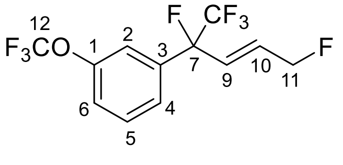

The reaction was performed according to **general procedure D** using 1-(trifluoromethoxy)-3-(1,1,1-trifluoropenta-2,4-dien-2-yl) benzene (**1p**, *E*:*Z* = 4:1) (56.0 mg, 0.2 mmol, 1.0 eq.). ^{19}F NMR analysis of the crude reaction mixture showed the formation of 86% 1,4-difluorinated product and 11% 1,2-difluorinated product. Column chromatography

(n-pentane:DCM 100:1) afforded product **2p** as a colorless oil (43.4 mg, 0.14 mmol, 68%).

R$_f$ = 0.43 (n-pentane:DCM 10:1).

FT-IR ($\tilde{\nu}$ = cm^{-1})**:** 2949 (w), 1593 (w), 1492 (w), 1446 (w), 1387 (w), 1253 (s), 1203 (s), 1156 (s), 1115 (m), 1088 (m), 1026 (m), 992 (m), 961 (m), 931 (m), 890 (w), 838 (w), 795 (m), 762 (w), 721 (m), 703 (m), 659 (m).

^{1}H NMR (500 MHz, $CDCl_3$): δ [ppm] = 7.49 (dd, $^3J_{HH}$ = 8.3 Hz, $^3J_{HH}$ = 7.6 Hz, 1H, H-C5), 7.44 (d, $^3J_{HH}$ = 7.9 Hz, 1H, H-C4), 7.37 (s, 1H, H-C2), 7.31 (dqdd, $^3J_{HH}$ = 8.0 Hz, $^5J_{HF}$ = 2.4 Hz, $^4J_{HH}$ = 1.2 Hz, $^4J_{HH}$ = 1.2 Hz, 1H, H-C6), 6.36 – 6.14 (m, 2H, H-C9, H-C10), 5.17 – 4.89 (m, 2H, H-C11).

^{13}C NMR (126 MHz, $CDCl_3$): δ [ppm] = 149.6 (qd, $^3J_{CF}$ = 1.9 Hz, $^4J_{CF}$ = 1.9 Hz, C1), 136.3 (d, $^2J_{CF}$ = 22.4 Hz, C3), 131.9 (ddq, $^2J_{CF}$ = 16.4 Hz, $^3J_{CF}$ = 11.2 Hz, $^4J_{CF}$ = 0.8 Hz, C10), 130.3 (d, $^4J_{CF}$ = 1.4 Hz, C5), 124.5 (dq, $^3J_{CF}$ = 8.6 Hz, $^4J_{CF}$ = 1.4 Hz, C4), 123.6 (dd, $^2J_{CF}$ = 18.3 Hz, $^3J_{CF}$ = 12.2 Hz, C9), 122.5 (dq, $^1J_{CF}$ = 285.3 Hz, $^2J_{CF}$ = 30.9 Hz, C8), 122.2 (dq, $^4J_{CF}$ = 1.2 Hz, $^5J_{CF}$ = 1.2 Hz, C6), 120.6 (q, $^1J_{CF}$ = 257.9 Hz, C12), 119.1 (d, $^3J_{CF}$ = 10.5 Hz, C2), 93.3 (dq, $^1J_{CF}$ = 190.7 Hz, $^2J_{CF}$ = 32.4 Hz, C7), 81.4 (d, $^1J_{CF}$ = 170.4 Hz, C11).

^{19}F NMR (470 MHz, $CDCl_3$): δ [ppm] = -52.72 – -61.23 (m, 3F, F-C12), -79.41 (dd, $^3J_{FF}$ = 8.4 Hz, $^6J_{FF}$ = 2.3 Hz, 3F, F-C8), -168.76 (dqd, $^3J_{HF}$ = 18.4 Hz, $^3J_{FF}$ = 10.6 Hz, $^4J_{HF}$ = 5.4 Hz, 1F, F-C7), -221.25 – -221.58 (m, 1F, F-C11).

^{19}F{^{1}H} NMR (470 MHz, $CDCl_3$): δ [ppm] = -50.46 – -64.01 (m, 3F, F-C12), -79.1 (dd, $^3J_{FF}$ = 8.4 Hz, $^6J_{FF}$ = 2.0 Hz, 3F, F-C8), -168.76 (qd, $^3J_{FF}$ = 8.4 Hz, $^5J_{FF}$ = 4.6 Hz, 1F, F-C7), -221.66 (d, $^5J_{FF}$ = 2.6 Hz, 1F, F-C11).

GC-EI-MS: (m/z) requires: [$(C_{12}H_8F_8O)^+$] = 320.0442, (m/z) found: [$(C_{12}H_8F_8O)^+$] = 320.0442.

(*E*)-4-(1,1,1,2,5-Pentafluoropent-3-en-2-yl)phenyl trifluoromethanesulfonate (2q)

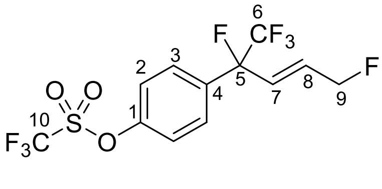

The reaction was performed according to **general procedure D** using (*E*)-4-(1,1,1-trifluoropenta-2,4-dien-2-yl)phenyl trifluoromethanesulfonate (**1q**, pure *E*) (69.6 mg, 0.2 mmol,

1.0 eq.). ^{19}F NMR analysis of the crude reaction mixture showed the formation of 91% 1,4-difluorinated product and 7% 1,2-difluorinated product. Column chromatography (*n*-pentane: DCM 30:1) afforded product **2q** as a colorless oil (60.7 mg, 0.16 mmol, 79%).

R$_f$ = 0.31 (*n*-pentane: DCM 10:1).

FT-IR ($\tilde{\nu}$ = cm^{-1})**:** 3459 (w), 3015 (m), 2970 (m), 2946 (m), 1739 (s), 1504 (m), 1427 (m), 1366 (s), 1274 (w), 1251 (m), 1216 (s), 1138 (s), 1107 (m), 1089 (m), 1017 (m), 957 (m), 881 (s), 841 (s), 799 (m), 783 (m), 745 (m), 698 (m), 606 (s), 575 (m), 527 (m).

^{1}H NMR (500 MHz, $CDCl_3$): δ [ppm] = 7.74 – 7.56 (m, 2H, H-C3), 7.46 – 7.29 (m, 2H, H-C2), 6.44 – 6.01 (m, 2H, H-C7, H-C8), 5.25 – 4.81 (m, 2H, H-C9).

^{13}C NMR (126 MHz, $CDCl_3$): δ [ppm] = 150.4 (d, $^{5}J_{CF}$ = 1.8 Hz, C1), 134.5 (d, $^{2}J_{CF}$ = 22.1 Hz, C4), 132.0 (dd, $^{2}J_{CF}$ = 16.3 Hz, $^{3}J_{CF}$ = 11.2 Hz, C8), 128.4 (dq, $^{3}J_{CF}$ = 9.3 Hz, $^{4}J_{CF}$ = 1.4 Hz, C3), 123.4 (dd, $^{2}J_{CF}$ = 18.2 Hz, $^{3}J_{CF}$ = 12.5 Hz, C7), 122.6 (qd, $^{1}J_{CF}$ = 285.0 Hz, $^{2}J_{CF}$ = 30.4 Hz, C6), 121.8 (d, $^{4}J_{CF}$ = 1.4 Hz, C2), 118.9 (q, $^{1}J_{CF}$ = 320.7 Hz, C10), 93.4 (dq, $^{1}J_{CF}$ = 190.8 Hz, $^{2}J_{CF}$ = 32.6 Hz, C5), 81.4 (d, $^{1}J_{CF}$ = 170.7 Hz, C9).

^{19}F NMR (470 MHz, $CDCl_3$): δ [ppm] = -72.87 (s, 3F, F-C10), -79.39 (d, $^{3}J_{FF}$ = 8.4 Hz, 3F, F-C6), -168.52 (dqdd, $^{3}J_{HF}$ = 17.0 Hz, $^{3}J_{FF}$ = 8.6 Hz, $^{4}J_{HF}$ = 7.0 Hz, $^{5}J_{FF}$ = 2.7 Hz, 1F, F-C5), -216.22 – -224.57 (m, 1F, F-C9).

^{19}F{^{1}H} NMR (470 MHz, $CDCl_3$): δ [ppm] = -72.87 (s, 3F, F-C10), -79.39 (d, $^{3}J_{FF}$ = 8.4 Hz, 3F, F-C6), -168.51 (dq, $^{3}J_{FF}$ = 8.5 Hz, $^{5}J_{FF}$ = 2.7 Hz, 1F, F-C5), -221.86 (d, 1F, $^{5}J_{FF}$ = 2.7 Hz, F-C9).

ESI-MS: (*m/z*) requires: [$(C_{12}H_8O_3SF_8Na)^+$] = 406.9959, (*m/z*) found: [$(C_{12}H_8O_3SF_8Na)^+$] = 406.9961.

(*E*)-4-(1,1,1,2,5-Pentafluoro-4-methylpent-3-en-2-yl)phenyl 4-methylbenzenesulfonate (2r)

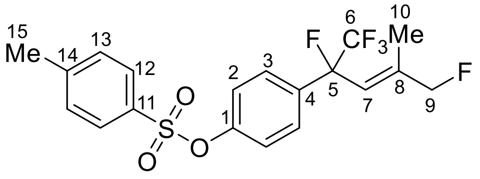

The reaction was performed according to **general procedure D** using (*E*)-4-(1,1,1-trifluoro-4-methyl penta-2,4-dien-2-yl)phenyl 4-methylbenzenesulfonate

(**1r**, Pure *E*) (76.5 mg, 0.2 mmol, 1.0 eq.). ^{19}F NMR analysis of the crude reaction mixture showed the formation of 52% 1,4-difluorinated product and < 5% 1,2-difluorinated product. Column chromatography (*n*-pentane: EtOAc 30:1) afforded product **2r** as a colorless liquid (40.3 mg, 0.10 mmol, 48%).

R*f* = 0.41 (*n*-pentane: EtOAc 10:1).

FT-IR ($\tilde{\nu}$ = cm^{-1})**:** 3456 (w), 3015 (m), 2970 (m), 2945 (m), 1739 (s), 1598 (w), 1503 (m), 1441 (m), 1372 (s), 1297 (s), 1228 (s), 1216 (s), 1203 (s), 1177 (s), 1157 (s), 1092 (m), 1054 (m), 1011 (m), 960 (m), 861 (s), 843 (m), 814 (m), 768 (m), 751 (m), 708 (m), 664 (m), 624 (m), 569 (s), 551 (s), 518 (m).

^{1}H NMR (500 MHz, $CDCl_3$): δ [ppm] = 7.73 – 7.68 (m, 2H, H-C12), 7.49 – 7.40 (m, 2H, H-C3), 7.33 – 7.29 (m, 2H, H-C13), 7.10 – 7.03 (m, 2H, H-C2), 6.08 (dq, $^{3}J_{HF}$ = 16.7 Hz, $^{4}J_{Hf}$ = 1.6 Hz, 1H, H-C7), 4.88 – 4.65 (m, 2H, H-C9), 2.45 (s, 3H, H-C15), 1.51 (dq, $^{4}J_{HF}$ = 3.6 Hz, $^{6}J_{HF}$ = 0.8 Hz, 3H, H-C10).

^{13}C NMR (126 MHz, $CDCl_3$): δ [ppm] = 150.5 (d, $^{5}J_{CF}$ = 1.8 Hz, C1), 145.8 (C14), 143.5 (dd, $^{2}J_{CF}$ = 13.5 Hz, $^{3}J_{CF}$ = 3.4 Hz, C8), 134.0 (d, $^{2}J_{CF}$ = 24.1 Hz, C4), 132.4 (C11), 130.0 (C13), 128.6 (C12), 128.2 (dq, $^{3}J_{CF}$ = 7.4 Hz, $^{4}J_{CF}$ = 1.2 Hz, C3), 122.5 (d, $^{4}J_{CF}$ = 1.1 Hz, C2), 122.9 (qd, $^{1}J_{CF}$ = 284.7 Hz, $^{2}J_{CF}$ = 31.0 Hz, C6), 117.8 (dd, $^{2}J_{CF}$ = 15.2 Hz, $^{3}J_{CF}$ = 12.6 Hz, C7), 93.5 (dq, $^{1}J_{CF}$ = 190.1 Hz, $^{2}J_{CF}$ = 31.8 Hz, C5), 85.4 (dd, $^{1}J_{CF}$ = 175.4 Hz, $^{4}J_{CF}$ = 1.8 Hz, C9), 21.8 (C15), 14.0 (t, $^{3}J_{CF}$ = 3.6 Hz, C10).

^{19}F NMR (470 MHz, $CDCl_3$): δ [ppm] = -81.29 (d, $^{3}J_{FF}$ = 9.4 Hz, 3F, F-C6), -161.56 (ddqd, $^{3}J_{HF}$ = 17.7 Hz, $^{4}J_{HF}$ = 13.4 Hz, $^{3}J_{FF}$ = 9.2 Hz, $^{5}J_{FF}$ = 4.7 Hz, 1F, F-C5), -216.38 – -216.89 (m, 1F, F-C9).

^{19}F{^{1}H} NMR (470 MHz, $CDCl_3$): δ [ppm] = -81.29 (d, $^{3}J_{FF}$ = 9.5 Hz, 3F, F-C6), -161.56 (qd, $^{3}J_{FF}$ = 9.4 Hz, $^{5}J_{FF}$ = 4.2 Hz, 1F, F-C5), -216.63 (d, $^{5}J_{FF}$ = 4.2 Hz, 1F, F-C9).

ESI-MS: (*m/z*) requires: [$(C_{19}H_{17}O_3SF_5Na)^+$] = 443.0711, (*m/z*) found: [$(C_{19}H_{17}O_3SF_5Na)^+$] = 443.0706.

(*E*)-(1,1,1,2,5-Pentafluoropent-3-en-2-yl)cyclohexane (2s)

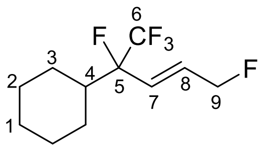

The reaction was performed according to **general procedure D** using (1,1,1-trifluoropenta-2,4-dien-2-yl)cyclohexane (**1s**, *E*:*Z* = 1:1.6) (40.3 mg, 0.2 mmol, 1.0 eq.). ^{19}F NMR analysis of the crude reaction mixture showed the formation of 53% 1,4-difluorinated product and 8% 1,2-difluorinated product. Column chromatography (*n*-pentane) afforded product **2s** as a colorless liquid (20.4 mg, 0.08 mmol, 43%).

R*f* = 0.50 (*n*-pentane).

FT-IR ($\tilde{\nu}$ = cm^{-1})**:** 3458 (w), 3015 (m), 2970 (m), 2937 (m), 2858 (m), 1739 (s), 1453 (m), 1366 (s), 1297 (m), 1228 (s), 1216 (s), 1205 (s), 1173 (m), 1156 (m), 1071 (m), 986 (m), 894 (m), 823 (w), 711 (w), 595 (w), 540 (m), 527 (m), 513 (m).

^{1}H NMR (500 MHz, $CDCl_3$): δ [ppm] = 6.23 – 6.00 (m, 1H, H-C7), 5.90 – 5.68 (m, 1H, H-C8), 5.15 – 4.85 (m, 2H, H-C9), 1.98 (dq, $^{3}J_{HF}$ = 12.3 Hz, $^{4}J_{HF}$ = 3.2 Hz, 1H, H-C4), 1.93 – 1.75 (m, 4H, H-C2, H-C3), 1.71 – 1.66 (m, 1H, H-C2), 1.37 – 1.20 (m, 2H, H-C1, H-C2), 1.71 – 1.66 (m, 3H, H-C1, H-C3).

^{13}C NMR (126 MHz, $CDCl_3$): δ [ppm] = 130.0 (dd, $^{2}J_{CF}$ = 16.3 Hz, $^{3}J_{CF}$ = 11.5 Hz, C7), 123.5 (ddd, $^{1}J_{CF}$ = 285.9 Hz, $^{2}J_{CF}$ = 29.9 Hz, $^{5}J_{CF}$ = 1.8 Hz, C6), 123.3 (ddd, $^{2}J_{CF}$ = 18.3 Hz, $^{3}J_{CF}$ = 12.2 Hz, $^{4}J_{CF}$ = 1.2 Hz, C8), 96.2 (dd, $^{1}J_{CF}$ = 190.1 Hz, $^{2}J_{CF}$ = 29.7 Hz, C5), 81.9 (d, $^{1}J_{CF}$ = 162.8 Hz, C9), 41.7 (dq, $^{2}J_{CF}$ = 29.7 Hz, $^{3}J_{CF}$ = 1.1 Hz, C4), 26.9 (dq, $^{3}J_{CF}$ = 6.6 Hz, $^{4}J_{CF}$ = 1.8 Hz, C3), 26.1 (C2), 26.0 (C2’), 25.8 (dq, $^{3}J_{CF}$ = 4.9 Hz, $^{4}J_{CF}$ = 1.0 Hz, C3’), 25.8 (C1).

^{19}F NMR (470 MHz, $CDCl_3$): δ [ppm] = -76.67 (d, $^{3}J_{FF}$ = 6.3 Hz, 3F, F-C6), -176.61 (ddq, $^{3}J_{HF}$ = 20.2 Hz, $^{3}J_{HF}$ = 13.2 Hz, $^{3}J_{FF}$ = 6.6 Hz, 1F, F-C5), -219.11 – -219.63 (m, 1F, F-C9).

^{19}F{^{1}H} NMR (377 MHz, $CDCl_3$): δ [ppm] = -76.67 (d, $^{3}J_{FF}$ = 6.3 Hz, 3F, F-C6), -171.90 – -178.33 (m, 1F, F-C5), -219.34 (d, $^{5}J_{FF}$ =1.6 Hz,1F, F-C9).

GC-EI-MS: (*m/z*) requires: [$(C_{11}H_{15}F_5)^+$] = 242.1088, (*m/z*) found: [$(C_{11}H_{15}F_5)^+$] = 242.1088.

(*E*)-2-(4-(1,1,1,2,5-Pentafluoropent-3-en-2-yl)phenyl)isoindoline-1,3-dione (2t)

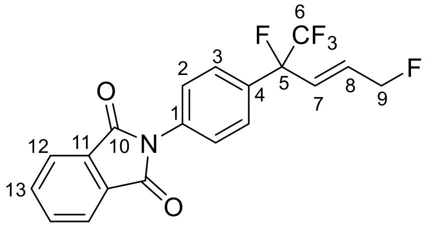

The reaction was performed according to **general procedure D** using 2-(4-(1,1,1-trifluoropenta-2,4-dien-2-yl)phenyl)isoindoline-1,3-dione (**1t**, *E*:*Z* = 2.3:1) (69.0 mg, 0.2 mmol, 1.0 eq.). ^{19}F NMR analysis of the crude reaction mixture showed the formation of 90% 1,4-difluorinated product and < 5% 1,2-difluorinated product. Column chromatography (*n*-pentane:EtOAc 8:1) afforded product **2t** as a white solid (57.7 mg, 0.15 mmol, 75%).

R*f* = 0.40 (*n*-pentane: EtOAc 4:1).

Melting Point: 132-133 ˚C.

FT-IR ($\tilde{\nu}$ = cm^{-1})**:** 3465 (m), 3015 (m), 2970 (m), 2946 (m), 1739 (s), 1607 (w), 1515 (m), 1423 (m), 1372 (s), 1262 (m), 1228 (s), 1216 (s), 1156 (s), 1120 (m), 1082 (m), 1019 (m), 992 (m), 945 (m), 918 (m), 888 (m), 852 (w), 842 (m), 823 (m), 795 (m), 750 (w), 711 (s), 699 (m), 646 (w), 567 (w), 527 (s).

^{1}H NMR (500 MHz, $CDCl_3$): δ [ppm] = 7.97 (dd, $^{3}J_{HF}$ = 5.5 Hz, $^{3}J_{HF}$ = 3.0 Hz, 2H, H-C12), 7.86 – 7.77 (m, 2H, H-C13), 7.65 (d, $^{3}J_{HF}$ = 8.7 Hz, 2H, H-C3), 7.62 – 7.53 (m, 2H, H-C2), 6.47 – 6.10 (m, 2H, H-C7, H-C8), 5.19 – 4.84 (m, 2H, H-C9).

^{13}C NMR (126 MHz, $CDCl_3$): δ [ppm] = 167.1 (C10), 134.8 (C13), 133.5 (dq, $^{2}J_{CF}$ = 22.3 Hz, $^{3}J_{CF}$ = 0.9 Hz, C4), 133.1 (d, $^{5}J_{CF}$ = 1.6 Hz, C1), 131.7 (C11), 131.5 (dd, $^{2}J_{CF}$ = 16.5 Hz, $^{3}J_{CF}$ = 11.1 Hz, C7), 127.0 (dq, $^{3}J_{CF}$ = 9.2 Hz, $^{4}J_{CF}$ = 1.4 Hz, C3), 126.4 (d, $^{4}J_{CF}$ = 1.4 Hz, C2), 124.03 (C12), 124.04 (dd, $^{2}J_{CF}$ = 18.0 Hz, $^{3}J_{CF}$ = 12.4 Hz, C8), 122.7 (qd, $^{1}J_{CF}$ = 285.1 Hz, $^{2}J_{CF}$ = 30.7 Hz, C6), 93.6 (dq, $^{1}J_{CF}$ = 189.8 Hz, $^{2}J_{CF}$ = 32.3 Hz, C5), 81.5 (d, $^{1}J_{CF}$ = 170.1 Hz, C9).

^{19}F NMR (470 MHz, $CDCl_3$): δ [ppm] = -79.16 (d, $^{3}J_{FF}$ = 8.5 Hz, 3F, F-C6), -168.62 (dqd, $^{3}J_{HF}$ = 16.9 Hz, $^{3}J_{FF}$ = 8.3 Hz, $^{4}J_{HF}$ = 11.0 Hz, 1F, F-C5), -216.23 – -224.68 (m, 1F, F-C9).

^{19}F{^{1}H} NMR (470 MHz, $CDCl_3$): δ [ppm] = -79.16 (d, $^{3}J_{FF}$ = 8.5 Hz, 3F, F-C6), -168.62 (dq, $^{3}J_{FF}$ = 8.5 Hz, $^{5}J_{FF}$ = 2.7 Hz, 1F, F-C5), -221.25 (d, $^{5}J_{FF}$ = 2.7 Hz, 1F, F-C9).

ESI-MS: (*m/z*) requires: [($C_{19}H_{12}O_2NF_5Na)^+$] = 404.0680, (*m/z*) found: [($C_{19}H_{12}O_2NF_5Na)^+$] = 404.0679.

3.3 Raw data for the plot of substrate regioselectivity versus $\delta_{C(ipso)}$ (ppm).

Substrates	Substituent	ipso C (ppm)	1,4-VS 1.2-	log 10 (1,4-VS 1.2-)
2a	*p*-Br	133	31	1.491
2b	*m*-Br	135.1	15	1.176
2c	*p*-Cl	132.5	40	1.602
2d	*m*-Cl	135.9	17	1.230
2f	*p*-F	129.9	23	1.362
2h	*p*-CF_3	137.8	3.8	0.580
2j	H	134	26	1.415
2k	*p*-NO_2	140.5	1.8	0.255
2m	*p*-CO_2Me	138.5	6.5	0.813
2n	*p*-SO_2Me	139.8	2	0.301
2p	*m*-OCF_3	136.3	7.8	0.892
2q	*p*-OTf	134.5	13	1.114

4. 1,4-Fluorofunctionalization reactions

4.1 Optimization of reaction conditions

Table S2 Optimization of 1,4-aminofluorination reaction conditions

S2-1 Screening of HF ratio. [a]

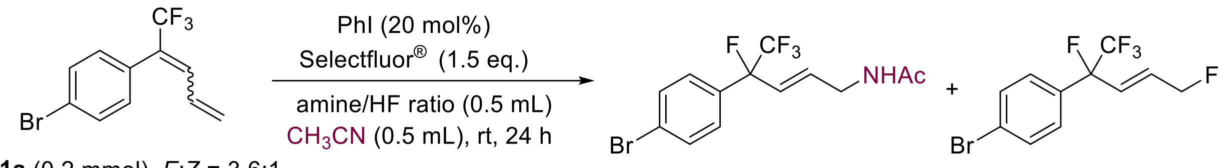

1a (0.2 mmol), *E*:*Z* = 3.6:1 **3a** **2a**

Entry	Amine/HF ratio	Yield of **3a** [b]	Yield of **2a** [b]	SM recovery [b]
1	1 : 9.2<br>Olah's reagent (70% HF)	62%	26%	0
2	1 : 8.5	50%	32%	0
3	1 : 7.5	39%	32%	0
4	1 : 6.5	8%	6%	52%
5	1 : 5.5	trace	trace	57%
6	DMPU-HF (65% HF)	64%	9%	0

[a] Reaction conditions: **1a** (0.2 mmol), PhI (20 mol%), Selectfluor® (1.5 eq.), amine:HF (0.5 mL) and CH_3CN (0.5 mL). [b] Yields were determined by ^{19}F NMR analysis of the crude reaction mixture using trifluoro toluene as an internal standard.

S2-2 Screening of concentration. [a]

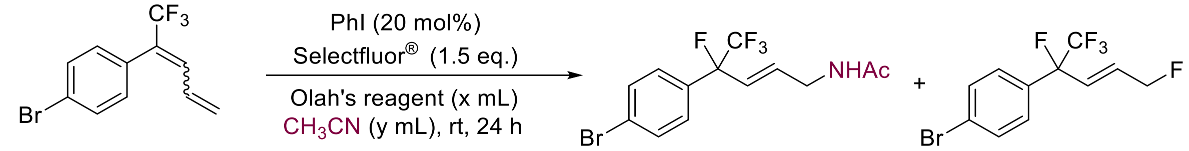

1a (0.2 mmol), *E*:*Z* = 3.6:1 **3a** **2a**

Entry	Olah's reagent (x mL)	CH_3CN (y mL)	Yield of **3a** [b]	Yield of **2a** [b]	SM recovery [b]
1	0.7	0.3	55%	28%	0
2	0.6	0.4	61%	25%	0
3	0.5	0.5	63%	23%	0
4	0.4	0.6	62%	23%	0
5	0.3	0.7	50%	20%	0
6	0.5	1.5	16%	5%	57%

[a] Reaction conditions: **1a** (0.2 mmol), PhI (20 mol%), Selectfluor® (1.5 eq.), Olah's reagent (x mL) and CH_3CN (y mL). [b] Yields were determined by ^{19}F NMR analysis of the crude reaction mixture using trifluoro toluene as an internal standard.

S2-3 Screening of catalyst. [a]

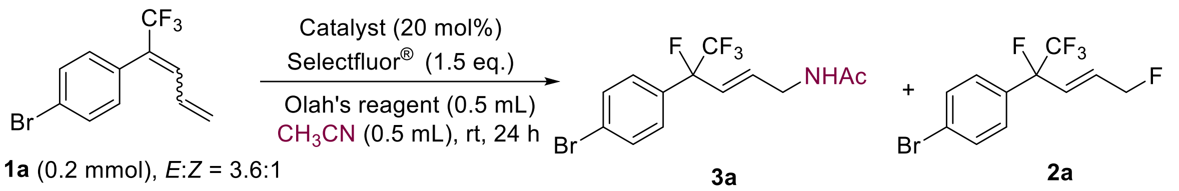

Entry	Catalyst	Yield of **3a** [b]	Yield of **2a** [b]	SM recovery [b]
1	I	62%	25%	0
2	II	55%	22%	0
3	III	59%	22%	0
4	IV	44%	17%	0
5	V	35%	15%	0
6	VI	57%	21%	0

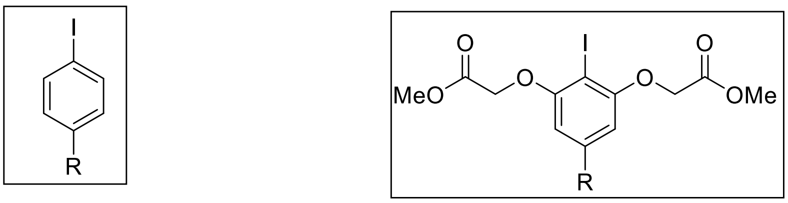

R = H, Catalyst I
R = Me, Catalyst II
R = CO_2Me, Catalyst III

R = H, Catalyst IV
R = Me, Catalyst V
R = CO_2Me, Catalyst VI

[a] Reaction conditions: **1a** (0.2 mmol), catalyst (20 mol%), Selectfluor® (1.5 eq.), Olah's reagent (0.5 mL) and CH_3CN (0.5 mL). [b] Yields were determined by ^{19}F NMR analysis of the crude reaction mixture using trifluoro toluene as an internal standard.

S2-4 Screening of nucleophile stoichiometry[a]

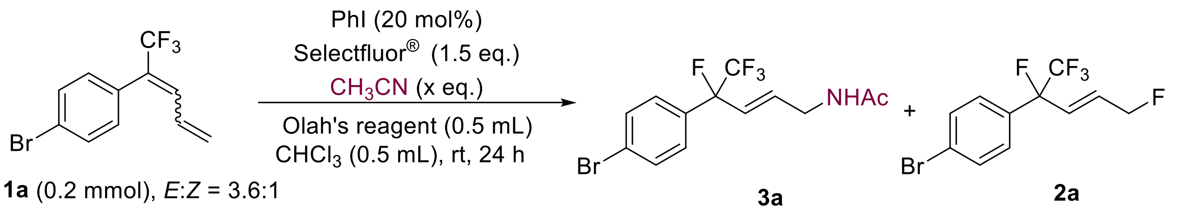

Entry	CH_3CN (x eq.)	Yield of **4a** [b]	Yield of **2a** [b]	SM recovery [b]
1	2	15%	72%	0
2	5	27%	54%	0
3	10	44%	43%	0
4	15	51%	35%	0
5	20	59%	30%	0
6	30	65%(53%) [c]	27%	0
7	40	64%	25%	0
8	50	64%	25%	0
9	60	62%	23%	0

[a] Reaction conditions: **1a** (0.2 mmol), PhI (20 mol%), Selectfluor® (1.5 eq.), CH_3CN (x mol%), Olah's reagent (0.5 mL) and $CHCl_3$ (0.5 mL). [b] Yields were determined by ^{19}F NMR analysis of the crude reaction

mixture using trifluoro toluene as an internal standard. [c] Isolated yield.

Table S3 Optimization of 1,4-oxyfluorination reaction conditions (AcOH as nucleophile)

S3-1 Screening of catalyst. [a]

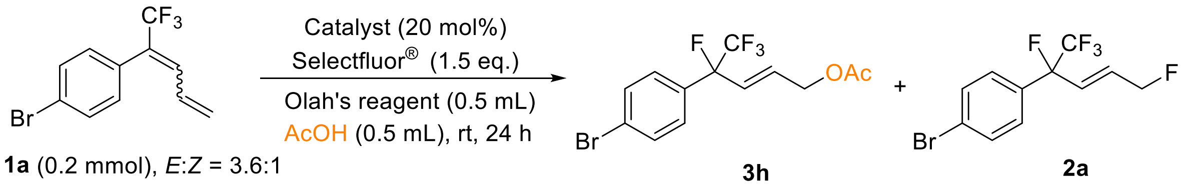

Entry	Catalyst	Yield of **3h** [b]	Yield of **2a** [b]	SM recovery [b]
1	I	56%	7%	0
2	II	53%	6%	0
3	III	52%	8%	0
4	IV	21%	4%	0
5	V	12%	3%	0
6	VI	44%	5%	0
7 [c]	I	29%	trace	0

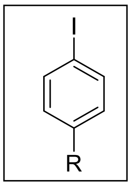

R = H, Catalyst I
R = Me, Catalyst II
R = CO_2Me, Catalyst III

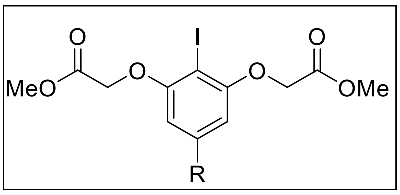

R = H, Catalyst IV
R = Me, Catalyst V
R = CO_2Me, Catalyst VI

[a] Reaction conditions: **1a** (0.2 mmol), catalyst (20 mol%), Selectfluor® (1.5 eq.), Olah's reagent (0.5 mL) and AcOH (0.5 mL). [b] Yields were determined by ^{19}F NMR analysis of the crude reaction mixture using trifluoro toluene as an internal standard. [c] DMPU·HF (0.5 mL) was used instead of Olah's reagent.

S3-2 Screening of nucleophile stoichiometry. [a]

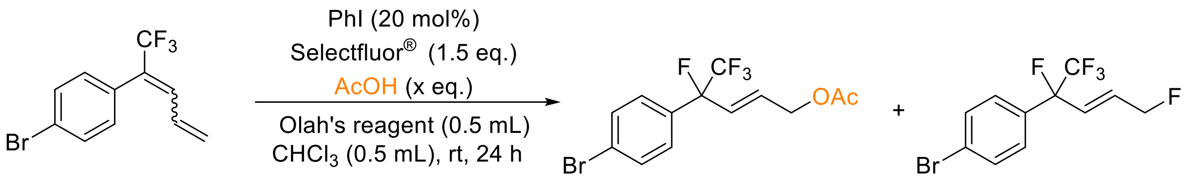

1a (0.2 mmol), *E*:*Z* = 3.6:1 **3h** **2a**

Entry	AcOH (x eq.)	Yield of **3h** [b]	Yield of **2a** [b]	SM recovery [b]
1	2	26%	62%	0
2	5	41%	40%	0
3	10	58%	28%	0
4	15	59%	20%	0
5	20	62%	16%	0
6	30	66%(63%) [c]	11%	0
7	40	61%	8%	0
8	50	60%	7%	0
9	60	61%	6%	0

[a] Reaction conditions: **1a** (0.2 mmol), PhI (20 mol%), Selectfluor® (1.5 eq.), AcOH (x mol%), Olah's reagent (0.5 mL) and $CHCl_3$ (0.5 mL). [b] Yields were determined by ^{19}F NMR analysis of the crude reaction mixture using trifluoro toluene as an internal standard. [c] Isolated yield.

Table S4 Optimization of 1,4-oxyfluorination reaction conditions (MeOH as nucleophile)

S4-1 Screening of nucleophile stoichiometry. [a]

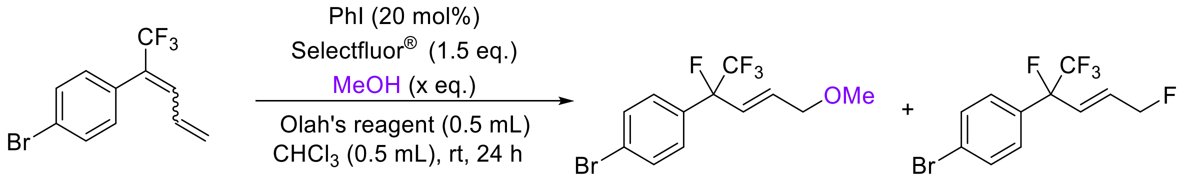

1a (0.2 mmol), *E*:*Z* = 3.6:1 **3m** **2a**

Entry	MeOH (x eq.)	Yield of **3m** [b]	Yield of **2a** [b]	SM recovery [b]
1	5	45%	45%	0
2	10	56%(56%) [c]	21%	0
3	15	54%	12%	0
4	20	55%	8%	0
5	30	48%	<5%	0
6	40	43%	<5%	0

[a] Reaction conditions: **1a** (0.2 mmol), PhI (20 mol%), Selectfluor® (1.5 eq.), MeOH (x mol%), Olah's reagent (0.5 mL) and $CHCl_3$ (0.5 mL). [b] Yields were determined by ^{19}F NMR analysis of the crude reaction mixture using trifluoro toluene as an internal standard. [c] Isolated yield.

Table S5 Optimization of 1,4-oxyfluorination reaction conditions (H_2O as nucleophile)

S5-1 Screening of nucleophile stoichiometry. [a]

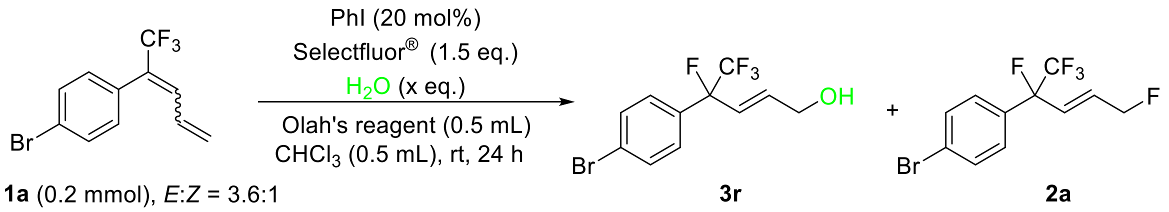

Entry	H_2O (x eq.)	Yield of **3r** [b]	Yield of **2a** [b]	SM recovery [b]
1	10	17%	59%	13%
2	20	26%(25%) [c]	31%	23%
3	30	22%	16%	46%
4	40	11%	5%	73%
5	50	trace	trace	84%

[a] Reaction conditions: **1a** (0.2 mmol), PhI (20 mol%), Selectfluor® (1.5 eq.), H_2O (x mol%), Olah's reagent (0.5 mL) and $CHCl_3$ (0.5 mL). [b] Yields were determined by ^{19}F NMR analysis of the crude reaction mixture using trifluoro toluene as an internal standard. [c] Isolated yield.

4.2 Catalytic 1,4-fluorofunctionalization reactions

General procedure E for the fluorofunctionalization

Unless otherwise stated, a Teflon® vial was equipped with a 1 cm stirring bar followed by the addition of 1,3-diene (0.2 mmol, 1.0 eq.), iodobenzene (8.2 mg, 0.04 mmol, 20 mol%), $CHCl_3$ (0.5 mL) and nucleophile (10 to 30 eq.). Olah's reagent (0.5 mL) was added via syringe. After stirring for 1 min, Selectfluor® (106 mg, 0.3 mmol, 1.5 eq.) was added in one portion. The reaction vessel was then sealed with a Teflon® screw cap. After stirring (350 rpm) at ambient temperature for 24 h, the reaction mixture was poured into 100 mL of a saturated solution of $NaHCO_3$ (CAUTION, strong generation of CO_2!). The Teflon® vial was rinsed with DCM and dropped into another flask with a saturated aqueous solution of $NaHCO_3$ to guarantee the removal of excess HF. The organics were extracted with DCM (3x 30 mL), the combined organic layers were dried over Na_2SO_4 and the solvent was carefully removed under reduced pressure. The yield was determined by ^{19}F NMR analysis of the crude reaction mixture using trifluoro toluene as an internal standard. The crude reaction mixture was purified by column

chromatography to afford the desired product.

(*E*)-*N*-(4-(4-bromophenyl)-4,5,5,5-tetrafluoropent-2-en-1-yl)acetamide (3a)

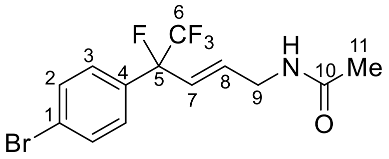

The reaction was performed according to **general procedure E** using 1-bromo-4-(1,1,1-trifluoropenta-2,4-dien-2-yl) benzene (**1a**, *E*:*Z* = 3.6:1) (55.4 mg, 0.2 mmol, 1.0 eq.) and CH_3CN (0.32 mL, 6.0 mmol, 30.0 eq.). ^{19}F NMR analysis of the crude reaction mixture showed the formation of 65% 1,4-aminofluorinated product and 22% 1,4-difluorinated product **2a**. Column chromatography (*n*-pentane:EtOAc 1:1) afforded product **3a** as a colorless oil (37.5 mg, 0.11 mmol, 53%).

R*f* = 0.38 (*n*-pentane:EtOAc 1:2).

FT-IR ($\tilde{\nu}$ = cm^{-1})**:** 3458 (m), 3283 (m), 3015 (m), 2970 (m), 2946 (m), 2850 (m), 2133 (w), 1739 (s), 1650 (m), 1549 (m), 1490 (m), 1435 (m), 1366 (s), 1287 (m), 1271 (m), 1228 (s), 1216 (s), 1092 (m), 1076 (m), 1031 (w), 1011 (m), 954 (m), 934 (m), 897 (m), 822 (m), 728 (m), 686 (m), 605 (m), 540 (m), 527 (m), 514 (m).

^{1}H NMR (599 MHz, $CDCl_3$): δ [ppm] = 7.63 – 7.52 (m, 2H, H-C2), 7.44 – 7.30 (m, 2H, H-C3), 6.12 – 5.95 (m, 2H, H-C7, H-C8), 5.78 (broad s, 1H, NH), 3.98 – 3.96 (m, 2H, H-C9), 2.00 (s, 3H, H-C11).

^{13}C NMR (151 MHz, $CDCl_3$): δ [ppm] = 170.1 (C10), 134.0 (d, $^3J_{CF}$ = 10.5 Hz, C8), 133.1 (d, $^2J_{CF}$ = 22.5 Hz, C4), 131.9 (d, $^4J_{CF}$ = 1.4 Hz, C2), 127.9 (dq, $^3J_{CF}$ = 8.7 Hz, $^4J_{CF}$ = 1.3 Hz, C3), 124.2 (d, $^5J_{CF}$ = 1.8 Hz, C1), 124.2 (d, $^2J_{CF}$ = 18.4 Hz, C7), 122.6 (dq, $^1J_{CF}$ = 285.0 Hz, $^2J_{CF}$ = 30.9 Hz, C6), 93.6 (dq, $^1J_{CF}$ = 188.7 Hz, $^2J_{CF}$ = 32.1 Hz, C5), 40.7 (C9), 23.3 (C11).

^{19}F NMR (564 MHz, $CDCl_3$): δ [ppm] = -79.28 (d, $^3J_{FF}$ = 8.8 Hz, 3F, F-C6), -165.93 (dqdd, $^3J_{HF}$ = 14.7 Hz, $^3J_{FF}$ = 8.5 Hz, $^4J_{HF}$ = 5.6 Hz, $^4J_{HF}$ = 3.1 Hz, 1F, F-C5).

^{19}F{^{1}H} NMR (564 MHz, $CDCl_3$): δ [ppm] = -79.28 (d, $^3J_{FF}$ = 8.8 Hz, 3F, F-C6), -165.93 (q, $^3J_{FF}$ = 8.5 Hz, 1F, F-C5).

ESI-MS: (*m/z*) requires: $[(C_{13}H_{12}NOBrF_4Na)^+]$ = 375.9931, (*m/z*) found: $[(C_{13}H_{12}NOBrF_4Na)^+]$ = 375.9929.

(*E*)-*N*-(4-(4-bromophenyl)-4,5,5,5-tetrafluoropent-2-en-1-yl)propionamide (3b)

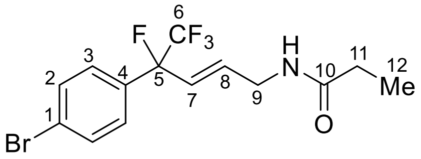

The reaction was performed according to **general procedure E** using 1-bromo-4-(1,1,1-trifluoropenta-2,4-dien-2-yl)benzene (**1a**, *E*:*Z* = 3.6:1) (55.1 mg, 0.2 mmol, 1.0 eq.) and EtCN (0.43 mL, 6.0 mmol, 30.0 eq.). ^{19}F NMR analysis of the crude reaction mixture showed the formation of 64% 1,4-aminofluorinated product and 28% 1,4-difluorinated product **2a**. Column chromatography (*n*-pentane:EtOAc 1:1) afforded product **3b** as a colorless oil (38.9 mg, 0.11 mmol, 53%).

$\mathbf{R_f}$ = 0.25 (*n*-pentane:EtOAc 1:1).

FT-IR ($\tilde{\nu}$ = cm^{-1})**:** 3457 (w), 3284 (m), 3015 (m), 2970 (m), 2945 (m), 2133 (w), 1739 (s), 1645 (m), 1593 (w), 1546 (m), 1490 (m), 1435 (m), 1366 (s), 1269 (m), 1271 (m), 1228 (s), 1216 (s), 1176 (s), 1108 (m), 1075 (m), 1028 (w), 1011 (m), 953 (m), 909 (m), 822 (m), 767 (w), 728 (m), 686 (m), 627 (w), 540 (m), 527 (m), 512 (m).

^{1}H NMR (500 MHz, $CDCl_3$): δ [ppm] = 7.61 – 7.49 (m, 2H, H-C2), 7.40 – 7.29 (m, 2H, H-C3), 6.22 – 5.91 (m, 2H, H-C7, H-C8), 5.77 (broad s, 1H, NH), 3.99 – 3.96 (m, 2H, H-C9), 2.22 (q, $^3J_{HH}$ = 7.6 Hz, 2H, H-C11), 1.15 (t, $^3J_{HH}$ = 7.6 Hz, 3H, H-C12).

^{13}C NMR (126 MHz, $CDCl_3$): δ [ppm] = 173.8 (C10), 134.2 (d, $^3J_{CF}$ = 10.4 Hz, C8), 133.1 (d, $^2J_{CF}$ = 22.5 Hz, C4), 131.9 (d, $^4J_{CF}$ = 1.4 Hz, C2), 127.9 (dq, $^3J_{CF}$ = 8.8 Hz, $^4J_{CF}$ = 1.3 Hz, C3), 124.2 (d, $^5J_{CF}$ = 1.8 Hz, C1), 124.1 (d, $^2J_{CF}$ = 18.4 Hz, C7), 122.6 (dq, $^1J_{CF}$ = 285.0 Hz, $^2J_{CF}$ = 31.0 Hz, C6), 93.5 (dq, $^1J_{CF}$ = 188.6 Hz, $^2J_{CF}$ = 32.0 Hz, C5), 40.5 (C9), 29.7 (C11), 9.9 (C12).

^{19}F NMR (470 MHz, $CDCl_3$): δ [ppm] = -79.31 (d, $^3J_{FF}$ = 8.8 Hz, 3F, F-C6), -165.72 – -165.98 (m, 1F, F-C5).

^{19}F{^{1}H} NMR (470 MHz, $CDCl_3$): δ [ppm] = -79.31 (d, $^3J_{FF}$ = 8.8 Hz, 3F, F-C6), -165.85 (q, $^3J_{FF}$ = 8.8 Hz, 1F, F-C5).

ESI-MS: (m/z) requires: $[(C_{14}H_{14}NOBrF_4Na)^+]$ = 390.0087, (m/z) found: $[(C_{14}H_{14}NOBrF_4Na)^+]$ = 390.0087.

(*E*)-*N*-(4-(4-bromophenyl)-4,5,5,5-tetrafluoropent-2-en-1-yl)isobutyramide (3c)

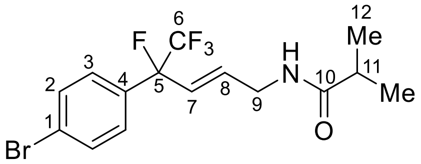

The reaction was performed according to **general procedure E** using 1-bromo-4-(1,1,1-trifluoropenta-2,4-dien-2-yl)benzene (**1a**, *E*:*Z* = 3.6:1) (55.5 mg, 0.2 mmol, 1.0 eq.) and *i*-PrCN (0.55 mL, 6.0 mmol, 30.0 eq.). ^{19}F NMR analysis of the crude reaction mixture showed the formation of 58% 1,4-aminofluorinated product and 21% 1,4-difluorinated product **2a**. Column chromatography (*n*-pentane:EtOAc 3:1) afforded product **3c** as a white solid (38.1 mg, 0.10 mmol, 50%).

R_f = 0.39 (*n*-pentane:EtOAc 2:1).

Melting Point: 68-69 ˚C.

FT-IR ($\tilde{\nu}$ = cm^{-1})**:** 3459 (w), 3285 (w), 3015 (m), 2970 (m), 2927 (m), 2871 (w), 1739 (s), 1659 (m), 1633 (m), 1593 (w), 1543 (m), 1488 (m), 1447 (m), 1401 (m), 1367 (s), 1308 (m), 1267 (m), 1230 (s), 1216 (s), 1200 (s), 1182 (s), 1159 (m), 1113 (m), 1094 (m), 1074 (m), 1028 (m), 1010 (m), 969 (m), 957 (m), 929 (m), 821 (m), 789 (m), 760 (w), 726 (m), 702 (m), 680 (m), 596 (w), 541 (m), 527 (m), 504 (m).

^{1}H NMR (500 MHz, $CDCl_3$): δ [ppm] = 7.61 – 7.49 (m, 2H, H-C2), 7.39 – 7.30 (m, 2H, H-C3), 6.18 – 5.92 (m, 2H, H-C7, H-C8), 5.75 (broad s, 1H, NH), 4.07 – 3.88 (m, 2H, H-C9), 2.27 (hept, $^3J_{HH}$ = 6.9 Hz, 1H, H-C11), 1.15 (dd, $^3J_{HH}$ = 6.9 Hz, $^8J_{HF}$ = 2.7 Hz, 6H, H-C12).

^{13}C NMR (126 MHz, $CDCl_3$): δ [ppm] = 177.1 (C10), 134.2 (d, $^3J_{CF}$ = 11.0 Hz, C8), 133.1 (d, $^2J_{CF}$ = 22.6 Hz, C4), 131.9 (d, $^4J_{CF}$ = 1.4 Hz, C2), 127.9 (dq, $^3J_{CF}$ = 8.8 Hz, $^4J_{CF}$ = 1.2 Hz, C3), 124.2 (d, $^5J_{CF}$ = 1.8 Hz, C1), 124.0 (d, $^2J_{CF}$ = 18.3 Hz, C7), 122.6 (dq, $^1J_{CF}$ = 285.0 Hz, $^2J_{CF}$ = 31.1 Hz, C6), 93.5 (dq, $^1J_{CF}$ = 188.6 Hz, $^2J_{CF}$ = 32.0 Hz, C5), 40.4 (C9), 35.7 (C11), 19.71 (d, $^7J_{CF}$ = 5.5 Hz, C12).

^{19}F NMR (470 MHz, $CDCl_3$): δ [ppm] = -79.35 (d, $^3J_{FF}$ = 8.8 Hz, 3F, F-C6), -165.83 (dqd, $^3J_{HF}$ = 16.9 Hz, $^3J_{FF}$ = 9.6 Hz, $^4J_{HF}$ = 6.6 Hz, 1F, F-C5).

^{19}F{^{1}H} NMR (470 MHz, $CDCl_3$): δ [ppm] = -79.35 (d, $^{3}J_{FF}$ = 8.8 Hz, 3F, F-C6), -165.83 (q, $^{3}J_{FF}$ = 8.8 Hz, 1F, F-C5).

ESI-MS: (*m/z*) requires: $[(C_{15}H_{16}NOBrF_4Na)^+]$ = 404.0244, (*m/z*) found: $[(C_{15}H_{16}NOBrF_4Na)^+]$ = 404.0244.

(*E*)-*N*-(4-(4-bromophenyl)-4,5,5,5-tetrafluoropent-2-en-1-yl)pivalamide (3d)

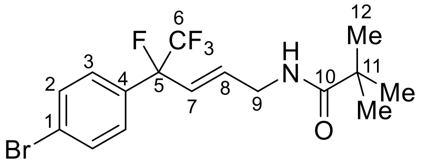

The reaction was performed according to **general procedure E** using 1-bromo-4-(1,1,1-trifluoropenta-2,4-dien-2-yl)benzene (**1a**, *E*:*Z* = 3.6:1) (55.3 mg, 0.2 mmol, 1.0 eq.) and *t*-BuCN (0.66 mL, 6.0 mmol, 30.0 eq.). ^{19}F NMR analysis of the crude reaction mixture showed the formation of 60% 1,4-aminofluorinated product and 22% 1,4-difluorinated product **2a**. Column chromatography (*n*-pentane:EtOAc 5:1) afforded product **3d** as a white solid (44.4 mg, 0.11 mmol, 56%).

R$_f$ = 0.22 (*n*-pentane:EtOAc 4:1).

Melting Point: 75-76 ˚C.

FT-IR ($\tilde{\nu}$ = cm^{-1})**:** 3459 (w), 3306 (w), 3015 (m), 2970 (m), 1739 (s), 1639 (m), 1593 (w), 1537 (m), 1479 (m), 1441 (m), 1367 (s), 1286 (w), 1259 (m), 1228 (s), 1216 (s), 1165 (m), 1115 (m), 1075 (m), 1035 (m), 1011 (m), 946 (m), 919 (m), 828 (w), 808 (m), 796 (m), 743 (w), 727 (w), 703 (w), 665 (m), 616 (w), 567 (w), 527 (m), 503 (m).

^{1}H NMR (500 MHz, $CDCl_3$): δ [ppm] = 7.68 – 7.49 (m, 2H, H-C2), 7.44 – 7.31 (m, 2H, H-C3), 6.19 – 5.93 (m, 2H, H-C7, H-C8), 5.83 (broad s, 1H, NH), 4.05 – 3.89 (m, 2H, H-C9), 1.20 (s, 9H, H-C12).

^{13}C NMR (126 MHz, $CDCl_3$): δ [ppm] = 178.5 (C10), 134.4 (d, $^{3}J_{CF}$ = 10.2 Hz, C8), 133.1 (d, $^{2}J_{CF}$ = 22.5 Hz, C4), 131.9 (d, $^{4}J_{CF}$ = 1.4 Hz, C2), 127.9 (dq, $^{3}J_{CF}$ = 8.9 Hz, $^{4}J_{CF}$ = 1.3 Hz, C3), 124.2 (d, $^{5}J_{CF}$ = 1.7 Hz, C1), 123.9 (d, $^{2}J_{CF}$ = 18.3 Hz, C7), 122.6 (dq, $^{1}J_{CF}$ = 284.4 Hz, $^{2}J_{CF}$ = 31.2 Hz, C6), 93.5 (dq, $^{1}J_{CF}$ = 188.7 Hz, $^{2}J_{CF}$ = 32.1 Hz, C5), 40.5 (C9), 38.9 (C11), 27.7 (C12).

^{19}F NMR (470 MHz, $CDCl_3$): δ [ppm] = -79.37 (d, $^3J_{FF}$ = 8.8 Hz, 3F, F-C6), -165.71 (dqd, $^3J_{HF}$ = 17.7 Hz, $^3J_{FF}$ = 9.9 Hz, $^4J_{HF}$ = 7.1 Hz, 1F, F-C5).

^{19}F{^{1}H} NMR (470 MHz, $CDCl_3$): δ [ppm] = -79.37 (d, $^3J_{FF}$ = 8.8 Hz, 3F, F-C6), -165.71 (q, $^3J_{FF}$ = 8.8 Hz, 1F, F-C5).

ESI-MS: (*m/z*) requires: [$(C_{16}H_{18}NOBrF_4H)^+$] = 396.0581, (*m/z*) found: [$(C_{16}H_{18}NOBrF_4H)^+$] = 396.0581.

(*E*)-*N*-(4-(4-bromophenyl)-4,5,5,5-tetrafluoropent-2-en-1-yl)benzamide (3e)

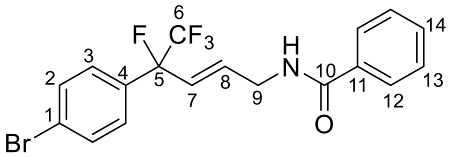

The reaction was performed according to **general procedure E** using 1-bromo-4-(1,1,1-trifluoropenta-2,4-dien-2-yl)benzene (**1a**, *E*:*Z* = 3.6:1) (55.3 mg, 0.2 mmol, 1.0 eq.) and PhCN (0.61 mL, 6.0 mmol, 30.0 eq.). ^{19}F NMR analysis of the crude reaction mixture showed the formation of 71% 1,4-aminofluorinated product and 22% 1,4-difluorinated product **2a**. Column chromatography (*n*-pentane:EtOAc 5:1) afforded product **3e** as a colorless liquid (52.4 mg, 0.13 mmol, 63%).

R_f = 0.31 (*n*-pentane:EtOAc 4:1).

FT-IR ($\tilde{\nu}$ = cm^{-1})**:** 3458 (w), 3307 (m), 3015 (m), 2970 (m), 2946 (m), 2133 (w), 1739 (s), 1638 (m), 1603 (m), 1578 (m), 1536 (m), 1488 (m), 1435 (m), 1366 (s), 1290 (m), 1270 (m), 1228 (s), 1216 (s), 1204 (s), 1099 (m), 1075 (m), 1029 (w), 1011 (m), 953 (m), 907 (m), 822 (m), 804 (m), 727 (m), 692 (m), 628 (m), 539 (m), 527 (m), 511 (m).

^{1}H NMR (500 MHz, $CDCl_3$): δ [ppm] = 7.85 – 7.71 (m, 2H, H-C12), 7.59 – 7.53 (m, 2H, H-C2), 7.53 – 7.48 (m, 1H, H-C14), 7.47 – 7.38 (m, 2H, H-C13), 7.35 (d, $^3J_{HH}$ = 8.4 Hz, 2H, H-C3), 6.46 (broad s, 1H, NH), 6.30 – 5.98 (m, 2H, H-C7, H-C8), 4.20 – 4.17 (m, 2H, H-C9).

^{13}C NMR (126 MHz, $CDCl_3$): δ [ppm] = 167.6 (C10), 134.1 (C11), 133.9 (d, $^3J_{CF}$ = 10.4 Hz, C8), 133.0 (d, $^2J_{CF}$ = 22.4 Hz, C4), 131.9 (d, $^4J_{CF}$ = 1.1 Hz, C2), 131.9 (C14), 128.8 (C13), 127.9 (dq, $^3J_{CF}$ = 8.8 Hz, $^4J_{CF}$ = 1.4 Hz, C3), 127.1 (C12), 124.4 (d, $^2J_{CF}$ = 18.4 Hz, C7), 124.2

(d, $^5J_{CF}$ = 1.8 Hz, C1), 122.6 (dq, $^1J_{CF}$ = 285.5 Hz, $^2J_{CF}$ = 31.0 Hz, C6), 93.6 (dq, $^1J_{CF}$ = 188.8 Hz, $^2J_{CF}$ = 32.1 Hz, C5), 41.8 (C9).

^{19}F NMR (470 MHz, $CDCl_3$): δ [ppm] = -79.23 (d, $^3J_{FF}$ = 8.7 Hz, 3F, F-C6), -165.96 (ddqd, $^3J_{HF}$ = 17.7 Hz, $^4J_{HF}$ = 11.5 Hz, $^3J_{FF}$ = 8.8 Hz, $^4J_{FH}$ = 4.8 Hz, 1F, F-C5).

^{19}F{^{1}H} NMR (470 MHz, $CDCl_3$): δ [ppm] = -79.23 (d, $^3J_{FF}$ = 8.8 Hz, 3F, F-C6), -165.96 (q, $^3J_{FF}$ = 8.8 Hz, 1F, F-C5).

ESI-MS: (*m/z*) requires: [($C_{18}H_{14}NOBrF_4Na)^+$] = 438.0087, (*m/z*) found: [($C_{18}H_{14}NOBrF_4Na)^+$] = 438.0082.

(*E*)-*N*-(4-(4-bromophenyl)-4,5,5,5-tetrafluoropent-2-en-1-yl)acetamide-2,2,2-*d*$_3$ (3f)

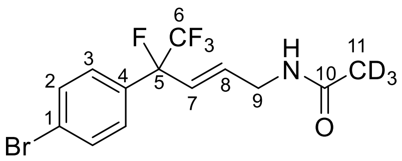

The reaction was performed according to **general procedure E** using 1-bromo-4-(1,1,1-trifluoropenta-2,4-dien-2-yl)benzene (**1a**, *E*:*Z* = 3.6:1) (55.9 mg, 0.2 mmol, 1.0 eq.) and CH_3CN-***d*$_3$** (0.31 mL, 6.0 mmol, 30.0 eq.). ^{19}F NMR analysis of the crude reaction mixture showed the formation of 65% 1,4-aminofluorinated product and 28% 1,4-difluorinated product **2a**. Column chromatography (*n*-pentane:EtOAc 1:1) afforded product **3f** as a colorless liquid (41.1 mg, 0.12 mmol, 57%).

R*f* = 0.19 (*n*-pentane:EtOAc 1:1).

FT-IR ($\tilde{\nu}$ = cm^{-1})**:** 3455 (w), 3282 (m), 3015 (m), 2970 (m), 2945 (m), 2135 (w), 1739 (s), 1642 (m), 1593 (w), 1548 (m), 1490 (m), 1435 (m), 1399 (m), 1366 (s), 1291 (m), 1270 (m), 1228 (s), 1217 (s), 1203 (s), 1176 (s), 1093 (m), 1076 (m), 1031 (w), 1011 (m), 954 (m), 938 (m), 907 (m), 822 (m), 728 (m), 686 (m), 626 (m), 538 (m), 527 (m), 516 (m).

^{1}H NMR (500 MHz, $CDCl_3$): δ [ppm] = 7.60 – 7.49 (m, 2H, H-C2), 7.38 – 7.30 (m, 2H, H-C3), 6.09 – 6.00 (m, 2H, H-C7, H-C8), 5.98 (broad s, 1H, NH), 4.03 – 3.88 (m, 2H, H-C9).

^{13}C NMR (126 MHz, $CDCl_3$): δ [ppm] = 170.3 (C10), 134.0 (d, $^3J_{CF}$ = 10.6 Hz, C8), 133.1 (d, $^2J_{CF}$ = 22.5 Hz, C4), 131.9 (d, $^4J_{CF}$ = 1.3 Hz, C2), 127.9 (dq, $^3J_{CF}$ = 8.8 Hz, $^4J_{CF}$ = 1.4 Hz, C3), 124.2 (d, $^5J_{CF}$ = 1.8 Hz, C1), 124.1 (d, $^2J_{CF}$ = 13.3 Hz, C7), 122.6 (dq, $^1J_{CF}$ = 285.0 Hz, $^2J_{CF}$ =

31.0 Hz, C6), 93.6 (dq, ${}^{1}J_{CF}$ = 188.7 Hz, ${}^{2}J_{CF}$ = 32.1 Hz, C5), 40.3 (C9), 22.5 (t, ${}^{2}J_{CD}$ = 19.3 Hz, C11).

^{19}F NMR (564 MHz, $CDCl_3$): δ [ppm] = -79.28 (d, ${}^{3}J_{FF}$ = 8.8 Hz, 3F, F-C6), -165.82 – -166.07 (m, 1F, F-C5).

^{19}F{^{1}H} NMR (564 MHz, $CDCl_3$): δ [ppm] = -79.28 (d, ${}^{3}J_{FF}$ = 8.8 Hz, 3F, F-C6), -165.93 (q, ${}^{3}J_{FF}$ = 8.8 Hz, 1F, F-C5).

ESI-MS: (*m/z*) requires: [$(C_{13}H_{19}NOBrD_3F_4Na)^+$] = 379.0119, (*m/z*) found: [$(C_{13}H_{19}NOBrD_3F_4Na)^+$] = 379.0118.

(*E*)-4-(4-Bromophenyl)-4,5,5,5-tetrafluoropent-2-en-1-yl formate (3g)

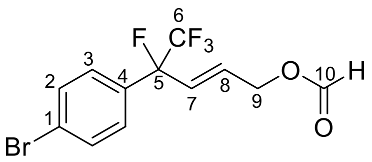

The reaction was performed according to **general procedure E** using 1-bromo-4-(1,1,1-trifluoropenta-2,4-dien-2-yl) benzene (**1a**, *E*:*Z* = 3.6:1) (55.2 mg, 0.2 mmol, 1.0 eq.) and HCO_2H (0.21 mL, 6.0 mmol, 30.0 eq.). ^{19}F NMR analysis of the crude reaction mixture showed the formation of 40% 1,4-oxyfluorinated product and 5% 1,4-difluorinated product **2a**. Column chromatography (*n*-pentane:Et_2O 10:1) afforded product **3g** as a colorless liquid (27.1 mg, 0.08 mmol, 40%).

R_f = 0.24 (*n*-pentane: Et_2O 15:1).

FT-IR ($\tilde{\nu}$ = cm^{-1})**:** 3457 (m), 3015 (m), 2970 (m), 2946 (m), 1737 (s), 1593 (w), 1490 (m), 1438 (m), 1366 (s), 1270 (m), 1228 (s), 1216 (s), 1203 (s), 1157 (s), 1092 (m), 1076 (m), 1011 (m), 953 (m), 894 (m), 822 (s), 727 (m), 685 (m), 627 (m), 526 (m), 514 (m).

^{1}H NMR (500 MHz, $CDCl_3$): δ [ppm] = 8.10 (s. 1H, H-C10), 7.83 – 7.49 (m, 2H, H-C2), 7.47 – 7.31 (m, 2H, H-C3), 6.42 – 6.01 (m, 2H, H-C7, H-C8), 4.93 – 4.67 (m, 2H, H-C9).

^{13}C NMR (126 MHz, $CDCl_3$): δ [ppm] = 160.3 (C10), 132.9 (d, ${}^{2}J_{CF}$ = 22.4 Hz, C4), 132.0 (d, ${}^{4}J_{CF}$ = 1.4 Hz, C2), 130.4 (dq, ${}^{3}J_{CF}$ = 11.4 Hz, ${}^{4}J_{CF}$ = 1.1 Hz, C8), 127.8 (dq, ${}^{3}J_{CF}$ = 9.0 Hz, ${}^{4}J_{CF}$ = 1.3 Hz, C3), 125.6 (d, ${}^{2}J_{CF}$ = 18.2 Hz, C7), 124.3 (d, ${}^{5}J_{CF}$ = 1.8 Hz, C1), 122.5 (dq, ${}^{1}J_{CF}$ = 285.1 Hz, ${}^{2}J_{CF}$ = 30.8 Hz, C6), 93.4 (dq, ${}^{1}J_{CF}$ = 189.9 Hz, ${}^{2}J_{CF}$ = 32.3 Hz, C5), 62.4 (C9).

^{19}F NMR (470 MHz, $CDCl_3$): δ [ppm] = -79.33 (d, $^3J_{FF}$ = 8.6 Hz, 3F, F-C6), -160.22 – -179.49 (m, 1F, F-C5).

^{19}F{^{1}H} NMR (376 MHz, $CDCl_3$): δ [ppm] = -79.34 (d, $^3J_{FF}$ = 8.6 Hz, 3F, F-C6), -167.15 (q, $^3J_{FF}$ = 8.6 Hz, 1F, F-C5).

ESI-MS: (*m/z*) requires: [$(C_{12}H_9O_2BrF_4Na)^+$] = 362.9614, (*m/z*) found: [$(C_{12}H_9O_2BrF_4Na)^+$] = 362.9614.

(*E*)-4-(4-Bromophenyl)-4,5,5,5-tetrafluoropent-2-en-1-yl acetate (3h)

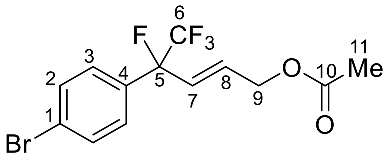

The reaction was performed according to **general procedure E** using 1-bromo-4-(1,1,1-trifluoropenta-2,4-dien-2-yl) benzene (**1a**, *E*:*Z* = 3.6:1) (55.4 mg, 0.2 mmol, 1.0 eq.) and AcOH (0.34 mL, 6.0 mmol, 30.0 eq.). ^{19}F NMR analysis of the crude reaction mixture showed the formation of 66% 1,4-oxyfluorinated product and 11% 1,4-difluorinated product **2a**. Column chromatography (*n*-pentane:Et_2O 30:1) afforded product **3h** as a colorless liquid (44.5 mg, 0.13 mmol, 63%).

R_f = 0.27 (*n*-pentane: Et_2O 15:1).

FT-IR ($\tilde{\nu}$ = cm^{-1})**:** 3049 (m), 3015 (m), 2970 (m), 2946 (m), 2134 (w), 1739 (s), 1593 (w), 1491 (m), 1437 (m), 1366 (s), 1228 (s), 1217 (s), 1091 (m), 1076 (m), 1029 (m), 1011 (m), 954 (m), 938 (m), 897 (m), 822 (m), 787 (m), 768 (m), 728 (m), 687 (w), 628 (m), 606 (m), 526 (m), 514 (m).

^{1}H NMR (500 MHz, $CDCl_3$): δ [ppm] = 7.64 – 7.50 (m, 2H, H-C2), 7.46 – 7.31 (m, 2H, H-C3), 6.31 – 5.99 (m, 2H, H-C7, H-C8), 4.85 – 4.56 (m, 2H, H-C9), 2.10 (s, 3H, H-C11).

^{13}C NMR (126 MHz, $CDCl_3$): δ [ppm] = 170.5 (C10), 133.0 (d, $^2J_{CF}$ = 22.5 Hz, C4), 132.0 (d, $^4J_{CF}$ = 1.4 Hz, C2), 131.4 (d, $^3J_{CF}$ = 11.2 Hz, C8), 127.9 (dq, $^3J_{CF}$ = 9.0 Hz, $^4J_{CF}$ = 1.3 Hz, C3), 125.0 (d, $^2J_{CF}$ = 18.2 Hz, C7), 124.3 (d, $^5J_{CF}$ = 1.7 Hz, C1), 122.6 (dq, $^1J_{CF}$ = 285.1 Hz, $^2J_{CF}$ = 30.8 Hz, C6), 93.5 (dq, $^1J_{CF}$ = 189.3 Hz, $^2J_{CF}$ = 32.3 Hz, C5), 62.1 (C9), 20.9 (C11).

^{19}F NMR (470 MHz, $CDCl_3$): δ [ppm] = -79.30 (d, $^3J_{FF}$ = 8.7 Hz, 3F, F-C6), -167.13 – -167.46 (m, 1F, F-C5).

^{19}F{^{1}H} NMR (470 MHz, $CDCl_3$): δ [ppm] = -79.30 (d, $^3J_{FF}$ = 8.7 Hz, 3F, F-C6), -167.30 (q, $^3J_{FF}$ = 8.6 Hz, 1F, F-C5).

ESI-MS: (*m/z*) requires: [$(C_{13}H_{11}O_2BrF_4Na)^+$] = 376.9771, (*m/z*) found: [$(C_{13}H_{11}O_2BrF_4Na)^+$] = 376.9773.

(*E*)-4-(4-Bromophenyl)-4,5,5,5-tetrafluoropent-2-en-1-yl propionate (3i)

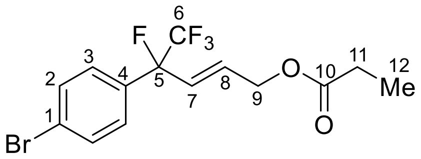

The reaction was performed according to **general procedure E** using 1-bromo-4-(1,1,1-trifluoropenta-2,4-dien-2-yl)benzene (**1a**, *E*:*Z* = 3.6:1) (55.3 mg, 0.2 mmol, 1.0 eq.) and EtCOOH (0.45 mL, 6.0 mmol, 30.0 eq.). ^{19}F NMR analysis of the crude reaction mixture showed the formation of 67% 1,4-oxyfluorinated product and 10% 1,4-difluorinated product **2a**. Column chromatography (*n*-pentane:Et_2O 50:1) afforded product **3i** as a colorless liquid (44.3 mg, 0.12 mmol, 60%).

R_f = 0.20 (*n*-pentane: Et_2O 50:1).

FT-IR ($\tilde{\nu}$ = cm^{-1})**:** 3456 (w), 2970 (m), 2945 (m), 1739 (s), 1593 (w), 1491 (w), 1454 (w), 1400 (w), 1366 (m), 1353 (m), 1271 (m), 1228 (m), 1217 (m), 1176 (s), 1011 (m), 1503 (m), 989 (w), 953 (m), 937 (m), 887 (m), 822 (m), 768 (m), 728 (m), 687 (w), 627 (m), 509 (m).

^{1}H NMR (500 MHz, $CDCl_3$): δ [ppm] = 7.60 – 7.52 (m, 2H, H-C2), 7.41 – 7.32 (m, 2H, H-C3), 6.24 – 6.07 (m, 2H, H-C7, H-C8), 4.73 – 4.60 (m, 2H, H-C9), 2.82 (q, 2H, $^3J_{HH}$ = 7.6 Hz, H-C11), 1.16 (t, 3H, $^3J_{HH}$ = 7.6 Hz, H-C11).

^{13}C NMR (126 MHz, $CDCl_3$): δ [ppm] = 173.9 (C10), 133.0 (d, $^2J_{CF}$ = 22.4 Hz, C4), 132.0 (d, $^4J_{CF}$ = 1.4 Hz, C2), 131.6 (d, $^3J_{CF}$ = 11.6 Hz, C8), 127.9 (dq, $^3J_{CF}$ = 8.9 Hz, $^4J_{CF}$ = 1.3 Hz, C3), 124.8 (d, $^2J_{CF}$ = 18.2 Hz, C7), 124.3 (d, $^5J_{CF}$ = 1.8 Hz, C1), 122.6 (dq, $^1J_{CF}$ = 285.0 Hz, $^2J_{CF}$ = 30.9 Hz, C6), 93.5 (dq, $^1J_{CF}$ = 189.4 Hz, $^2J_{CF}$ = 32.3 Hz, C5), 63.0 (C9), 27.5 (C11), 9.2 (C12).

^{19}F NMR (470 MHz, $CDCl_3$): δ [ppm] = -79.32 (d, $^3J_{FF}$ = 8.6 Hz, 3F, F-C6), -167.22 – -167.46 (ddqd, $^3J_{HF}$ = 14.4 Hz, $^3J_{FF}$ = 8.9 Hz, $^4J_{HF}$ = 5.3 Hz, $^4J_{FH}$ = 2.5 Hz, 1F, F-C5).

^{19}F{^{1}H} NMR (470 MHz, $CDCl_3$): δ [ppm] = -79.32 (d, $^3J_{FF}$ = 8.6 Hz, 3F, F-C6), -167.22 (q, $^3J_{FF}$ = 8.6 Hz, 1F, F-C5).

ESI-MS: (*m/z*) requires: [$(C_{14}H_{13}O_2BrF_4Na)^+$] = 390.9927, (*m/z*) found: [$(C_{14}H_{13}O_2BrF_4Na)^+$] = 390.9928.

(*E*)-4-(4-Bromophenyl)-4,5,5,5-tetrafluoropent-2-en-1-yl hexanoate (3j)

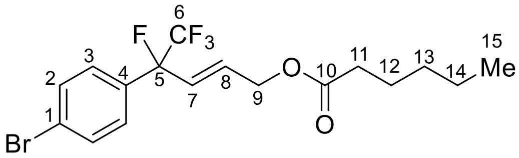

The reaction was performed according to **general procedure E** using 1-bromo-4-(1,1,1-trifluoro penta-2,4-dien-2-yl)benzene (**1a**, *E*:*Z* = 3.6:1) (55.9 mg, 0.2 mmol, 1.0 eq.) and $C_5H_{11}CO_2H$ (0.75 mL, 6.0 mmol, 30.0 eq.). ^{19}F NMR analysis of the crude reaction mixture showed the formation of 72% 1,4-oxyfluorinated product and 13% 1,4-difluorinated product **2a**. Column chromatography (*n*-pentane:Et_2O 50:1) afforded product **3j** as a colorless liquid (57.5 mg, 0.14 mmol, 69%).

R*f* = 0.35 (*n*-pentane: Et_2O 20:1).

FT-IR ($\tilde{\nu}$ = cm^{-1})**:** 3457 (w), 3015 (m), 2970 (m), 2946 (m), 2131 (w), 1789 (s), 1739 (s), 1592 (w), 1487 (m), 1449 (m), 1364 (s), 1285 (m), 1271 (m), 1259 (m), 1228 (s), 1216 (s), 1177 (s), 1159 (s), 1106 (s), 1073 (m), 1055 (m), 1009 (m), 1011 (m), 978 (m), 952 (m), 931 (m), 815 (m), 791 (w), 766 (m), 726 (m), 688 (m), 644 (w), 629 (w), 594 (w), 514 (m).

^{1}H NMR (500 MHz, $CDCl_3$): δ [ppm] = 7.62 – 7.51 (m, 2H, H-C2), 7.45 – 7.32 (m, 2H, H-C3), 6.25 – 6.06 (m, 2H, H-C7, H-C8), 4.73 – 4.61 (m, 2H, H-C9), 2.35 (t, $^3J_{HH}$ = 7.5 Hz, 2H, H-C11), 1.79 – 1.55 (m, 2H, H-C12), 1.42 – 1.21 (m, 4H, H-C13, H-C14), 0.89 (t, $^3J_{HH}$ = 7.0 Hz, 3H, H-C15).

^{13}C NMR (126 MHz, $CDCl_3$): δ [ppm] = 173.3 (C10), 133.0 (d, $^2J_{CF}$ = 22.5 Hz, C4), 131.9 (d, $^4J_{CF}$ = 1.4 Hz, C2), 131.6 (d, $^3J_{CF}$ = 11.1 Hz, C8), 127.9 (dq, $^3J_{CF}$ = 8.9 Hz, $^4J_{CF}$ = 1.4 Hz, C3), 124.9 (d, $^2J_{CF}$ = 18.3 Hz, C7), 124.3 (d, $^5J_{CF}$ = 1.7 Hz, C1), 122.6 (dq, $^1J_{CF}$ = 285.1 Hz, $^2J_{CF}$ =

30.9 Hz, C6), 93.5 (dq, $^{1}J_{CF}$ = 189.6 Hz, $^{2}J_{CF}$ = 32.2 Hz, C5), 62.9 (C9), 34.2 (C11), 31.4 (C12), 24.7 (C13), 22.4 (C14), 14.0 (C15).

^{19}F NMR (470 MHz, $CDCl_3$): δ [ppm] = -79.32 (d, $^{3}J_{FF}$ = 8.7 Hz, 3F, F-C6), -167.22 – -167.12 (m, 1F, F-C5).

^{19}F{^{1}H} NMR (377 MHz, $CDCl_3$): δ [ppm] = -79.30 (d, $^{3}J_{FF}$ = 8.7 Hz, 3F, F-C6), -167.15 (q, $^{3}J_{FF}$ = 8.6 Hz, 1F, F-C5).

ESI-MS: (*m/z*) requires: $[(C_{17}H_{19}O_2BrF_4Na)^+]$ = 433.0397, (*m/z*) found: $[(C_{17}H_{19}O_2BrF_4Na)^+]$ = 433.0396.

(*E*)-4-(4-Bromophenyl)-4,5,5,5-tetrafluoropent-2-en-1-yl cyclopropanecarboxylate (3k)

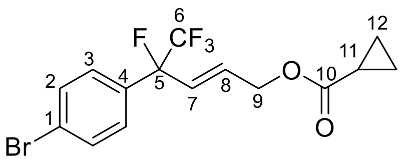

The reaction was performed according to **general procedure E** using 1-bromo-4-(1,1,1-trifluoro penta-2,4-dien-2-yl)benzene (**1a**, *E*:*Z* = 3.6:1) (55.2 mg, 0.2 mmol, 1.0 eq.) and cyclopropanecarboxylic acid (0.48 mL, 6.0 mmol, 30.0 eq.). ^{19}F NMR analysis of the crude reaction mixture showed the formation of 68% 1,4-oxyfluorinated product and 10% 1,4-difluorinated product **2a**. Column chromatography (*n*-pentane:Et_2O 40:1) afforded product **3k** as a colorless liquid (43.6 mg, 0.11 mmol, 57%).

R*f* = 0.31 (*n*-pentane: EtOAc 20:1).

FT-IR ($\tilde{\nu}$ = cm^{-1})**:** 3457 (w), 3015 (m), 2970 (m), 2947 (m), 1738 (s), 1593 (w), 1490 (w), 1447 (m), 1399 (m), 1365 (s), 1268 (m), 1228 (s), 1216 (s), 1203 (s), 1160 (s), 1106 (s), 1093 (m), 1076 (m), 1011 (m), 953 (m), 893 (m), 854 (m), 822 (m), 728 (m), 687 (w), 627 (m), 538 (m), 514 (m).

^{1}H NMR (500 MHz, $CDCl_3$): δ [ppm] = 7.66 – 7.54 (m, 2H, H-C2), 7.48 – 7.32 (m, 2H, H-C3), 6.29 – 6.05 (m, 2H, H-C7, H-C8), 4.74 – 4.63 (m, 2H, H-C9), 1.66 (tt, $^{3}J_{HH}$ = 8.0 Hz, $^{3}J_{HH}$ = 8.0 Hz, 1H, H-C11), 1.06 – 0.98 (m, 2H, H-C12), 0.94 – 0.86 (m, 2H, H-C12).

^{13}C NMR (126 MHz, $CDCl_3$): δ[ppm] = 174.4 (C10), 133.1 (d, $^2J_{CF}$ = 22.4 Hz, C4), 131.9 (d, $^4J_{CF}$ = 1.3 Hz, C2), 131.6 (d, $^3J_{CF}$ = 11.3 Hz, C8), 127.9 (dq, $^3J_{CF}$ = 8.9 Hz, $^4J_{CF}$ = 1.4 Hz, C3), 124.8 (d, $^2J_{CF}$ = 18.3 Hz, C7), 124.3 (d, $^5J_{CF}$ = 1.8 Hz, C1), 122.6 (dq, $^1J_{CF}$ = 285.1 Hz, $^2J_{CF}$ = 30.8 Hz, C6), 93.5 (dq, $^1J_{CF}$ = 189.4 Hz, $^2J_{CF}$ = 32.2 Hz, C5), 63.1 (C9), 12.9 (C11), 8.9 (C12).

^{19}F NMR (470 MHz, $CDCl_3$): δ [ppm] = -79.30 (d, $^3J_{FF}$ = 8.7 Hz, 3F, F-C6), -167.02 – -167.31 (m, 1F, F-C5).

^{19}F{^{1}H} NMR (377 MHz, $CDCl_3$): δ[ppm] = -79.30 (d, $^3J_{FF}$ = 8.7 Hz, 3F, F-C6), -167.14 (q, $^3J_{FF}$ = 8.7 Hz, 1F, F-C5).

ESI-MS: (*m/z*) requires: [$(C_{15}H_{13}O_2BrF_4Na)^+$] = 402.9927, (*m/z*) found: [$(C_{15}H_{13}O_2BrF_4Na)^+$] = 402.9929.

(*E*)-4-(4-Bromophenyl)-4,5,5,5-tetrafluoropent-2-en-1-yl 3-oxocyclobutane-1-carboxylate (3l)

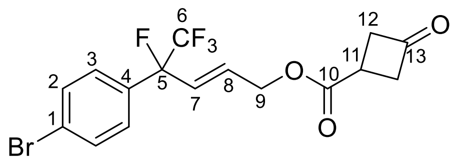

The reaction was performed according to **general procedure E** using 1-bromo-4-(1,1,1-trifluoro penta-2,4-dien-2-yl)benzene (**1a**, *E*:*Z* = 3.6:1) (55.7 mg, 0.2 mmol, 1.0 eq.) and 3-oxocyclobutane-1-carboxylic acid (0.69 g, 6.0 mmol, 30.0 eq.). ^{19}F NMR analysis of the crude reaction mixture showed the formation of 40% 1,4-oxyfluorinated product and 6% 1,4-difluorinated product **2a**. Column chromatography (*n*-pentane: EtOAc 10:1) afforded product **3l** as a white solid (28.6 mg, 0.07 mmol, 35%).

R*f* = 0.20 (*n*-pentane: EtOAc 10:1).

Melting Point: 42-43 ˚C.

FT-IR ($\tilde{\nu}$ = cm^{-1})**:** 3457 (w), 3015 (m), 2970 (m), 2946 (m), 2131 (w), 1789 (s), 1739 (s), 1592 (w), 1487 (m), 1449 (m), 1399 (m), 1364 (s), 1285 (m), 1271 (m), 1259 (m), 1228 (s), 1216 (s), 1203 (s), 1177 (s), 1159 (s), 1106 (s), 1073 (m), 1055 (m), 1009 (m), 978 (m), 931 (m), 815 (m), 791 (w), 766 (m), 726 (m), 688 (m), 644 (w), 629 (w), 594 (w), 514 (m).

^{1}**H NMR** (500 MHz, $CDCl_3$): δ [ppm] = 7.59 – 7.55 (m, 2H, H-C2), 7.40 – 7.32 (m, 2H, H-C3), 6.25 – 6.11 (m, 2H, H-C7, H-C8), 4.80 – 4.70 (m, 2H, H-C9), 3.45 – 3.37 (m, 2H, H-C12), 3.35 – 3.23 (m, 3H, H-C11, H-C12).

^{13}**C NMR** (126 MHz, $CDCl_3$): δ [ppm] = 203.1 (C13), 173.6 (C10), 133.8 (d, $^{2}J_{CF}$ = 22.4 Hz, C4), 131.9 (d, $^{4}J_{CF}$ = 1.3 Hz, C2), 130.7 (d, $^{3}J_{CF}$ = 11.4 Hz, C8), 127.8 (dq, $^{3}J_{CF}$ = 9.0 Hz, $^{4}J_{CF}$ = 1.4 Hz, C3), 125.8 (d, $^{2}J_{CF}$ = 18.3 Hz, C7), 124.3 (d, $^{5}J_{CF}$ = 1.7 Hz, C1), 122.5 (dq, $^{1}J_{CF}$ = 285.1 Hz, $^{2}J_{CF}$ = 30.8 Hz, C6), 93.4 (dq, $^{1}J_{CF}$ = 189.8 Hz, $^{2}J_{CF}$ = 32.3 Hz, C5), 63.9 (C9), 51.8 (d, $^{7}J_{CF}$ = 1.1 Hz, C1), 27.4 (C12).

^{19}**F NMR** (470 MHz, $CDCl_3$): δ [ppm] = -79.29 (d, $^{3}J_{FF}$ = 8.6 Hz, 3F, F-C6), -167.69 – -167.97 (m, 1F, F-C5).

^{19}**F{^{1}H} NMR** (470 MHz, $CDCl_3$): δ [ppm] = -79.29 (d, $^{3}J_{FF}$ = 8.6 Hz, 3F, F-C6), -167.82 (q, $^{3}J_{FF}$ = 8.6 Hz, 1F, F-C5).

ESI-MS: (*m/z*) requires: [$(C_{16}H_{13}O_3BrF_4Na)^+$] = 430.9876, (*m/z*) found: [$(C_{16}H_{13}O_3BrF_4Na)^+$] = 430.9876.

(*E*)-1-Bromo-4-(1,1,1,2-tetrafluoro-5-methoxypent-3-en-2-yl)benzene (3m)

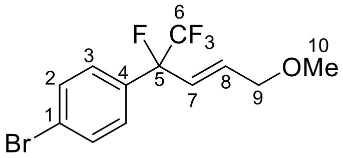

The reaction was performed according to **general procedure E** using 1-bromo-4-(1,1,1-trifluoro penta-2,4-dien-2-yl)benzene (**1a**, *E*:*Z* = 3.6:1) (55.7 mg, 0.2 mmol, 1.0 eq.) and methanol (0.08 mL, 2.0 mmol, 10.0 eq.). ^{19}F NMR analysis of the crude reaction mixture showed the formation of 56% 1,4-oxyfluorinated product and 17% 1,4-difluorinated product **2a**. Column chromatography (*n*-pentane: Et_2O 100:1) afforded product **3m** as a colorless liquid (33.4 mg, 0.11 mmol, 56%).

$\mathbf{R_f}$ = 0.58 (*n*-pentane: EtOAc 10:1).

FT-IR ($\tilde{\nu}$ = cm^{-1})**:** 3457 (w), 3015 (m), 2970 (m), 2945 (m), 2574 (w), 2129 (w), 1739 (s), 1593 (w), 1488 (m), 1438 (m), 1366 (s), 1270 (m), 1228 (s), 1216 (s), 1124 (m), 1075 (m), 1053 (w), 1011 (m), 952 (m), 939 (m), 910 (m), 822 (m), 790 (m), 768 (m), 729 (m), 685 (m), 627 (m), 598 (m), 572 (w), 527 (s), 515 (m).

^{1}**H NMR** (400 MHz, $CDCl_3$): δ [ppm] = 7.76 – 7.49 (m, 2H, H-C2), 7.38 (d, $^{3}J_{HH}$ = 8.5 Hz, 2H, H-C3), 6.46 – 5.95 (m, 2H, H-C7, H-C8), 4.21 – 3.87 (m, 2H, H-C9), 3.88 (s, 3H, H-C10).

^{13}**C NMR** (101MHz, $CDCl_3$): δ [ppm] = 133.9 (d, $^{3}J_{CF}$ = 10.6 Hz, C8), 133.4 (d, $^{2}J_{CF}$ = 22.5 Hz, C4), 131.8 (d, $^{4}J_{CF}$ = 1.4 Hz, C2), 127.9 (dq, $^{3}J_{CF}$ = 9.0 Hz, $^{4}J_{CF}$ = 1.4 Hz, C3), 124.1 (d, $^{5}J_{CF}$ = 1.8 Hz, C1), 123.3 (d, $^{2}J_{CF}$ = 18.1 Hz, C7), 122.7 (dq, $^{1}J_{CF}$ = 285.0 Hz, $^{2}J_{CF}$ = 31.0 Hz, C6), 93.7 (dq, $^{1}J_{CF}$ = 188.8 Hz, $^{2}J_{CF}$ = 32.1 Hz, C5), 71.4 (C9), 58.7 (C10).

^{19}**F NMR** (470 MHz, $CDCl_3$): δ [ppm] = -79.42 (d, $^{3}J_{FF}$ = 8.7 Hz, 3F, F-C6), -167.19 (ddqd, $^{3}J_{HF}$ = 17.4 Hz, $^{4}J_{HF}$ = 11.9 Hz, $^{3}J_{FF}$ = 8.7 Hz, $^{4}J_{FH}$ = 3.0 Hz, 1F, F-C5).

^{19}**F{^{1}H} NMR** (470 MHz, $CDCl_3$): δ [ppm] = -79.42 (d, $^{3}J_{FF}$ = 8.6 Hz, 3F, F-C6), -167.19 (q, $^{3}J_{FF}$ = 8.6 Hz, 1F, F-C5).

GC-EI-MS: (*m/z*) requires: [$(C_{12}H_{11}BrOF_4)^+$] = 325.9929, (*m/z*) found: [$(C_{12}H_{11}BrOF_4)^+$] = 325.9917.

(*E*)-1-Bromo-4-(5-ethoxy-1,1,1,2-tetrafluoropent-3-en-2-yl)benzene (3n)

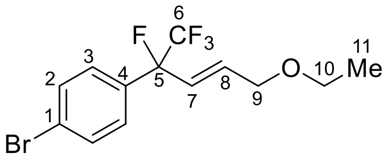

The reaction was performed according to **general procedure E** using 1-bromo-4-(1,1,1-trifluoro penta-2,4-dien-2-yl) benzene (**1a**, *E*:*Z* = 3.6:1) (55.8 mg, 0.2 mmol, 1.0 eq.) and ethanol (0.12 mL, 2.0 mmol, 10.0 eq.). ^{19}F NMR analysis of the crude reaction mixture showed the formation of 58% 1,4-oxyfluorinated product and 27% 1,4-difluorinated product **2a**. Column chromatography (*n*-pentane: DCM 7:1) afforded product **3n** as a colorless liquid (38.6 mg, 0.11 mmol, 56%).

$\mathbf{R}_f$ = 0.22 (*n*-pentane: DCM 5:1).

FT-IR ($\tilde{\nu}$ = cm^{-1})**:** 3457 (m), 3015 (m), 2970 (s), 2946 (m), 2132 (w), 1739 (s), 1593 (w), 1440 (m), 1366 (s), 1269 (m), 1228 (s), 1216 (s), 1204 (m), 1076 (m), 1011 (m), 954 (m), 895 (m), 822 (m), 787 (m), 768 (m), 729 (m), 685 (w), 627 (m), 539 (m), 526 (m), 515 (m).

^{1}**H NMR** (500 MHz, $CDCl_3$): δ [ppm] = 7.60 – 7.53 (m, 2H, H-C2), 7.45 – 7.35 (m, 2H, H-C3), 6.28 – 6.02 (m, 2H, H-C7, H-C8), 4.11 – 4.03 (m, 2H, H-C9), 3.52 (q, $^{3}J_{CF}$ = 7.0 Hz, 2H, H-C10), 1.23 (t, $^{3}J_{CF}$ = 7.0 Hz, 3H, H-C11).

^{13}**C NMR** (126MHz, $CDCl_3$): δ [ppm] = 134.5 (d, $^{3}J_{CF}$ = 10.5 Hz, C8), 133.5 (d, $^{2}J_{CF}$ = 22.4 Hz, C4), 131.8 (d, $^{4}J_{CF}$ = 1.5 Hz, C2), 128.0 (dq, $^{3}J_{CF}$ = 9.2 Hz, $^{4}J_{CF}$ = 1.4 Hz, C3), 124.0 (d, $^{5}J_{CF}$ = 1.8 Hz, C1), 123.0 (d, $^{2}J_{CF}$ = 18.0 Hz, C7), 122.7 (dq, $^{1}J_{CF}$ = 285.0 Hz, $^{2}J_{CF}$ = 31.0 Hz, C6), 93.8 (dq, $^{1}J_{CF}$ = 188.5 Hz, $^{2}J_{CF}$ = 32.0 Hz, C5), 69.5 (C9), 66.6 (C10), 15.2 (C11).

^{19}**F NMR** (470 MHz, $CDCl_3$): δ [ppm] = -79.38 (d, $^{3}J_{FF}$ = 8.7 Hz, 3F, F-C6), -166.76 (ddqd, $^{3}J_{HF}$ = 16.4 Hz, $^{4}J_{HF}$ = 12.1 Hz, $^{3}J_{FF}$ = 8.7 Hz, $^{4}J_{FH}$ = 3.1 Hz, 1F, F-C5).

^{19}**F{^{1}H} NMR** (470 MHz, $CDCl_3$): δ [ppm] = -79.38 (d, $^{3}J_{FF}$ = 8.7 Hz, 3F, F-C6), -167.76 (q, $^{3}J_{FF}$ = 8.7 Hz, 1F, F-C5).

ESI-MS: (*m/z*) requires: [($C_{13}H_{13}OBrF_4Na)^+$] = 362.9978, (*m/z*) found: [($C_{13}H_{13}OBrF_4Na)^+$] = 362.9977.

(*E*)-1-Bromo-4-(1,1,1,2-tetrafluoro-5-isopropoxypent-3-en-2-yl)benzene (3o)

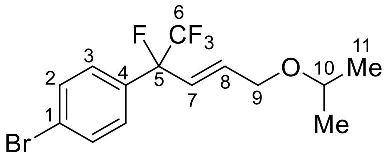

The reaction was performed according to **general procedure E** using 1-bromo-4-(1,1,1-trifluoro penta-2,4-dien-2-yl) benzene (**1a**, *E*:*Z* = 3.6:1) (55.6 mg, 0.2 mmol, 1.0 eq.) and isopropyl alcohol (0.15 mL, 2.0 mmol, 10.0 eq.). ^{19}F NMR analysis of the crude reaction mixture showed the formation of 51% 1,4-oxyfluorinated product and 41% 1,4-difluorinated product **2a**. Column chromatography (*n*-pentane: DCM 6:1) afforded product **3o** as a colorless liquid (30.9 mg, 0.09 mmol, 44%).

R*f* = 0.23 (*n*-pentane: DCM 5:1).

FT-IR ($\tilde{\nu}$ = cm^{-1})**:** 3457 (m), 3015 (m), 2970 (s), 2946 (m), 2574 (w), 2128 (w), 1739 (s), 1593 (w), 1437 (m), 1367 (s), 1269 (m), 1228 (s), 1216 (s), 1093 (m), 1075 (m), 1029 (m), 1011 (m), 953 (m), 935 (m), 897 (m), 821 (m), 782 (m), 729 (m), 686 (w), 627 (m), 539 (m), 527 (m), 513 (m).

^{1}H NMR (500 MHz, $CDCl_3$): δ [ppm] = 7.63 – 7.50 (m, 2H, H-C2), 7.46 – 7.31 (m, 2H, H-C3), 6.33 – 6.00 (m, 2H, H-C7, H-C8), 4.08 – 4.06 (m, 2H, H-C9), 3.61 (hept, $^{3}J_{HH}$ = 6.1 Hz, 1H, H-C10), 1.18 (dd, $^{3}J_{HH}$ = 6.1 Hz, $^{8}J_{HF}$ = 1.9 Hz, 6H, H-C11).

^{13}C NMR (126MHz, $CDCl_3$): δ [ppm] = 135.7 (d, $^{3}J_{CF}$ = 10.4 Hz, C8), 133.5 (d, $^{2}J_{CF}$ = 22.5 Hz, C4), 131.8 (d, $^{4}J_{CF}$ = 1.4 Hz, C2), 128.0 (dq, $^{3}J_{CF}$ = 9.0 Hz, $^{4}J_{CF}$ = 1.3 Hz, C3), 124.0 (d, $^{5}J_{CF}$ = 1.8 Hz, C1), 122.7 (d, $^{2}J_{CF}$ = 18.2 Hz, C7), 122.8 (dq, $^{1}J_{CF}$ = 285.3 Hz, $^{2}J_{CF}$ = 31.3 Hz, C6), 93.8 (dq, $^{1}J_{CF}$ = 188.3 Hz, $^{2}J_{CF}$ = 32.0 Hz, C5), 71.9 (C10), 67.2 (C9), 22.2 (d, $^{7}J_{CF}$ = 2.3 Hz, C11).

^{19}F NMR (470 MHz, $CDCl_3$): δ [ppm] = -79.38 (d, $^{3}J_{FF}$ = 8.7 Hz, 3F, F-C6), -166.45 (ddqd, $^{3}J_{HF}$ = 21.5 Hz, $^{4}J_{HF}$ = 13.2 Hz, $^{3}J_{FF}$ = 8.8 Hz, $^{4}J_{FH}$ = 3.9 Hz, 1F, F-C5).

^{19}F{^{1}H} NMR (470 MHz, $CDCl_3$): δ [ppm] = -79.38 (d, $^{3}J_{FF}$ = 8.7 Hz, 3F, F-C6), -166.45 (q, $^{3}J_{FF}$ = 8.7 Hz, 1F, F-C5).

ESI-MS: (*m/z*) requires: $[(C_{14}H_{15}OBrF_4Na)^+]$ = 377.0135, (*m/z*) found: $[(C_{14}H_{15}OBrF_4Na)^+]$ = 377.0135.

(*E*)-1-Bromo-4-(1,1,1,2-tetrafluoro-5-(2-(2-methoxyethoxy)ethoxy)pent-3-en-2-yl)benzene (3p)

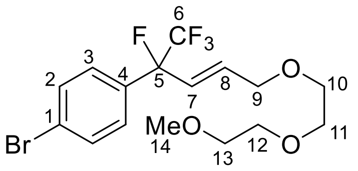

The reaction was performed according to **general procedure E** using 1-bromo-4-(1,1,1-trifluoro penta-2,4-dien-2-yl) benzene (**1a**, *E*:*Z* = 3.6:1) (55.8 mg, 0.2 mmol, 1.0 eq.) and methoxypoly(ethylene glycol) (0.24 mL, 2.0 mmol, 10.0 eq.). ^{19}F NMR analysis of the crude reaction mixture showed the formation of 61% 1,4-oxyfluorinated product and 15% 1,4-difluorinated product **2a**. Column chromatography (*n*-pentane: Et_2O 4:1) afforded product **3p** as a colorless liquid (46.2 mg, 0.11 mmol, 55%).

R_f = 0.48 (*n*-pentane: EtOAc 2:1).

FT-IR ($\tilde{\nu}$ = cm^{-1})**:** 3457 (w), 3015 (m), 2970 (s), 2945 (m), 2574 (w), 2130 (w), 1739 (s), 1592 (w), 1441 (m), 1366 (s), 1269 (m), 1228 (s), 1216 (s), 1104 (m), 1075 (m), 1011 (m),

953 (m), 939 (m), 896 (m), 822 (m), 787 (m), 768 (m), 730 (m), 685 (w), 621 (m), 539 (m), 527 (m), 514 (m).

^{1}H NMR (500 MHz, $CDCl_3$): δ [ppm] = 7.59 – 7.50 (m, 2H, H-C2), 7.43 – 7.31 (m, 2H, H-C3), 6.29 – 6.16 (m, 1H, H-C7), 6.11 (dtd, $^3J_{HH}$ = 15.7 Hz, $^3J_{HH}$ = 4.4 Hz, $^4J_{HF}$ = 1.2 Hz, 1H, H-C8), 4.17 – 4.09 (m, 2H, H-C9), 3.74 – 3.60 (m, 6H, H-C10, H-C11, H-C12), 3.59 – 3.52 (m, 2H, H-C13), 3.37 (s, 3H, H-C14).

^{13}C NMR (126MHz, $CDCl_3$): δ [ppm] = 134.0 (d, $^3J_{CF}$ = 10.5 Hz, C8), 133.4 (d, $^2J_{CF}$ = 22.5 Hz, C4), 131.8 (d, $^4J_{CF}$ = 1.4 Hz, C2), 127.9 (dq, $^3J_{CF}$ = 9.2 Hz, $^4J_{CF}$ = 1.4 Hz, C3), 124.0 (d, $^5J_{CF}$ = 1.7 Hz, C1), 123.2 (d, $^2J_{CF}$ = 18.1 Hz, C7), 122.7 (dq, $^1J_{CF}$ = 285.0 Hz, $^2J_{CF}$ = 31.1 Hz, C6), 93.7 (dq, $^1J_{CF}$ = 188.7 Hz, $^2J_{CF}$ = 32.0 Hz, C5), 72.0 (C13), 70.8 (C11), 70.7 (C12), 70.2 (C9), 70.1 (C10), 59.1 (C14).

^{19}F NMR (470 MHz, $CDCl_3$): δ [ppm] = -79.40 (d, $^3J_{FF}$ = 8.7 Hz, 3F, F-C6), -167.04 (dqd, $^3J_{HF}$ = 14.3 Hz, $^3J_{FF}$ = 8.3 Hz, $^4J_{FH}$ = 3.9 Hz, 1F, F-C5).

^{19}F{^{1}H} NMR (470 MHz, $CDCl_3$): δ [ppm] = -79.40 (d, $^3J_{FF}$ = 8.7 Hz, 3F, F-C6), -167.03 (q, $^3J_{FF}$ = 8.7 Hz, 1F, F-C5).

ESI-MS: (*m/z*) requires: $[(C_{16}H_{19}O_3BrF_4Na)^+]$ = 437.0350, (*m/z*) found: $[(C_{16}H_{19}O_3BrF_4Na)^+]$ = 437.0355.

Methyl (2*S*)-2-(((*E*)-4-(4-bromophenyl)-4,5,5,5-tetrafluoropent-2-en-1-yl)oxy)propanoate (3q)

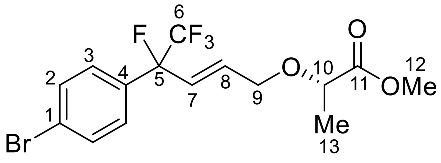

d.r. = 1:1

The reaction was performed according to **general procedure E** using 1-bromo-4-(1,1,1-trifluoro penta-2,4-dien-2-yl) benzene (**1a**, *E*:*Z* = 3.6:1) (55.2 mg, 0.2 mmol, 1.0 eq.) and methyl (*S*)-(-)-lactate (0.19 mL, 2.0 mmol, 10.0 eq.). ^{19}F NMR analysis of the crude reaction mixture showed the formation of 56% 1,4-oxyfluorinated product and 34% 1,4-difluorinated product **2a**. Column chromatography (*n*-pentane: Et_2O 10:1) afforded product **3q** as a colorless liquid (33.1 mg, 0.08 mmol, 42%, *d.r.* = 1:1).

$\mathbf{R}_f$ = 0.25 (*n*-pentane: EtOAc 10:1).

FT-IR ($\tilde{\nu}$ = cm^{-1})**:** 3457 (m), 3015 (m), 2970 (s), 2947 (m), 2574 (w), 2131 (w), 1739 (s), 1592 (w), 1437 (s), 1367 (s), 1270 (m), 1228 (s), 1216 (s), 1145 (m), 1092 (m), 1076 (m), 1011 (m), 955 (m), 896 (m), 822 (m), 789 (m), 729 (m), 686 (w), 627 (m), 539 (m), 527 (m), 515 (m).

^{1}H NMR (500 MHz, $CDCl_3$): δ [ppm] = 7.59 – 7.54 (m, 2H, H-C2), 7.38 – 7.35 (m, 2H, H-C3), 6.30 – 6.03 (m, 2H, H-C7, H-C8), 4.28 – 4.22 (m, 1H, H-C9), 4.13 – 3.96 (m, 2H, H-C9, H-C10), 3.74 (s, 3H, H-C12), 1.44 (d, 3H, $^3J_{HH}$ = 3.9 Hz, H-C13), 1.43 (d, 3H, $^3J_{HH}$ = 3.9 Hz, H-C13).

^{13}C NMR (126MHz, $CDCl_3$): δ [ppm] = 173.41 (C11), 173.40 (C11), 133.54 (d, $^3J_{CF}$ = 10.7 Hz, C8), 133.54 (d, $^3J_{CF}$ = 10.7 Hz, C8), 133.6 (d, $^2J_{CF}$ = 22.5 Hz, C4), 133.3 (d, $^2J_{CF}$ = 22.3 Hz, C4), 131.87 (d, $^4J_{CF}$ = 1.4 Hz, C2), 131.85 (d, $^4J_{CF}$ = 1.4 Hz, C2), 128.9 (dq, $^3J_{CF}$ = 9.0 Hz, $^4J_{CF}$ = 1.2 Hz, C3), 127.9 (dq, $^3J_{CF}$ = 9.0 Hz, $^4J_{CF}$ = 1.2 Hz, C3), 124.10 (C1), 124.11 (C1), 123.8 (d, $^2J_{CF}$ = 18.1 Hz, C7), 123.6 (d, $^2J_{CF}$ = 18.0 Hz, C7), 122.66 (dq, $^1J_{CF}$ = 285.3 Hz, $^2J_{CF}$ = 31.4 Hz, C6), 122.64 (dq, $^1J_{CF}$ = 285.2 Hz, $^2J_{CF}$ = 31.4 Hz, C6), 93.69 (dq, $^1J_{CF}$ = 189.0 Hz, $^2J_{CF}$ = 32.2 Hz, C5), 93.66 (dq, $^1J_{CF}$ = 189.3 Hz, $^2J_{CF}$ = 32.0 Hz, C5), 74.92 (C10), 74.89 (C10), 69.13 (C9), 69.08 (C9), 52.1 (C12), 18.7 (C13).

^{19}F NMR (470 MHz, $CDCl_3$): δ [ppm] = -79.31 (d, $^3J_{FF}$ = 8.7 Hz, 3F, F-C6), -79.37 (d, $^3J_{FF}$ = 8.7 Hz, 3F, F-C6), -166.70 – -166.90 (m, 1F, F-C5), -166.93 – -167.17 (m, 1F, F-C5).

^{19}F{^{1}H} NMR (470 MHz, $CDCl_3$): δ [ppm] = -79.31 (d, $^3J_{FF}$ = 8.6 Hz, 3F, F-C6), -79.37 (d, $^3J_{FF}$ = 8.8 Hz, 3F, F-C6), -166.85 (q, $^3J_{FF}$ = 8.6 Hz, 1F, F-C5), -167.08 (q, $^3J_{FF}$ = 8.8 Hz, 1F, F-C5).

ESI-MS: (*m/z*) requires: [$(C_{15}H_{15}O_3BrF_4Na)^+$] = 421.0038, (*m/z*) found: [$(C_{15}H_{15}O_3BrF_4Na)^+$] = 421.0032.

(*E*)-4-(4-Bromophenyl)-4,5,5,5-tetrafluoropent-2-en-1-ol (3r)

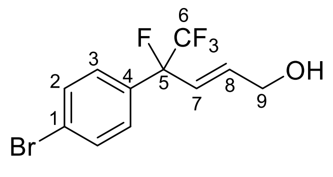

The reaction was performed according to **general procedure E** using 1-bromo-4-(1,1,1-trifluoro penta-2,4-dien-2-yl)benzene (**1a**,

E:Z = 3.6:1) (54.9 mg, 0.2 mmol, 1.0 eq.) and H_2O (0.07 mL, 4.0 mmol, 20.0 eq.). ^{19}F NMR analysis of the crude reaction mixture showed the formation of 26% 1,4-oxyfluorinated product and 31% 1,4-difluorinated product **2a**. Column chromatography (*n*-pentane: Et_2O 4:1) afforded product **3r** as a colorless liquid (15.5 mg, 0.05 mmol, 25%).

R*f* = 0.31 (*n*-pentane: Et_2O 3:1).

FT-IR ($\tilde{\nu}$ = cm^{-1})**:** 3456 (w), 3015 (m), 2970 (m), 2946 (m), 2133 (w), 1739 (s), 1593 (w), 1489 (m), 1437 (m), 1366 (s), 1269 (m), 1228 (s), 1216 (s), 1174 (s), 1094 (m), 1075 (m), 1010 (m), 953 (m), 907 (m), 822 (m), 795 (m), 768 (m), 728 (m), 685 (m), 648 (w), 628 (w), 601 (m), 540 (m), 527 (m), 511 (m).

^{1}H NMR (500 MHz, $CDCl_3$): δ [ppm] = 7.58 – 7.55 (m, 2H, H-C2), 7.44 – 7.33 (m, 2H, H-C3), 6.32 – 6.12 (m, 2H, H-C7, H-C8), 4.38 – 4.20 (m, 2H, H-C9), 1.68 (broad s, 1H, OH).

^{13}C NMR (126 MHz, $CDCl_3$): δ [ppm] = 136.4 (dd, $^3J_{CF}$ = 10.1 Hz, $^4J_{CF}$ = 1.0 Hz, C8), 133.4 (d, $^2J_{CF}$ = 22.5 Hz, C4), 131.9 (d, $^4J_{CF}$ = 1.4 Hz, C2), 127.9 (dq, $^3J_{CF}$ = 9.0 Hz, $^4J_{CF}$ = 1.4 Hz, C3), 124.1 (d, $^5J_{CF}$ = 1.7 Hz, C1), 122.7 (dq, $^1J_{CF}$ = 285.0 Hz, $^2J_{CF}$ = 31.1 Hz, C6), 122.3 (d, $^2J_{CF}$ = 18.3 Hz, C7), 93.8 (dq, $^1J_{CF}$ = 188.8 Hz, $^2J_{CF}$ = 32.1 Hz, C5), 62.1 (C9).

^{19}F NMR (470 MHz, $CDCl_3$): δ [ppm] = -79.41 (d, $^3J_{FF}$ = 8.6 Hz, 3F, F-C6), -167.01 – -167.25 (m, 1F, F-C5).

^{19}F{^{1}H} NMR (470 MHz, $CDCl_3$): δ [ppm] = -79.41 (d, $^3J_{FF}$ = 8.6 Hz, 3F, F-C6), -167.12 (q, $^3J_{FF}$ = 8.6 Hz, 1F, F-C5).

ESI-MS: (*m/z*) requires: [$(C_{11}H_{10}OBrF_4)^+$] = 312.9846, (*m/z*) found: [$(C_{11}H_{10}OBrF_4)^+$] = 312.9844.

5. Synthetic applications

5.1 Large scale reaction

The reaction was performed according to **general procedure D:** To a 100 mL Teflon® vial equipped with a stirring bar was added 1-bromo-4-(1,1,1-trifluoropenta-2,4-dien-2-yl)

benzene (**1a**, *E*:*Z* = 3.6:1) (832.6 mg, 3.0 mmol, 1.0 eq.), iodobenzene (121.9 mg, 0.6 mmol, 20 mol%) and $CHCl_3$ (7.5 mL). A mixture of amine:HF 1:7.5 (7.5 mL) was added via syringe. After stirring for 1 min, Selectfluor® (1.6 g, 4.5 mmol, 1.5 eq.) was added in one portion. The reaction vessel was then sealed with a Teflon® screw cap. After stirring (350 rpm) at ambient temperature for 24 h,the reaction mixture was quenched with a saturated solution of $NaHCO_3$ (CAUTION, generation of CO_2!). The organics were extracted with DCM (3x 60 mL), the combined organic layers were dried over Na_2SO_4 and the solvent was carefully removed under reduced pressure. Column chromatography (*n*-pentane:DCM 150:1) afforded product **2a** as colorless oil (807.5 mg, 2.56 mmol, 85%).

5.2 Transformation of allylic fluorides

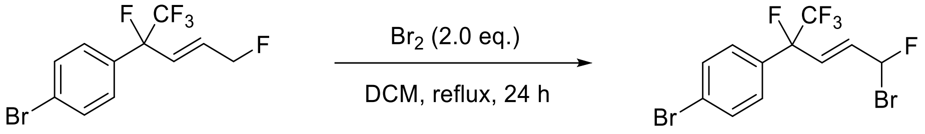

2a **4a** 61%, *d.r.* = 1:1

(*E*)-1-Bromo-4-(5-bromo-1,1,1,2,5-pentafluoropent-3-en-2-yl)benzene (4a)

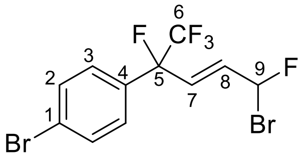

According to a modified literature procedure,[13] bromine (20 µL, 0.4 mmol, 2.0 eq. was slowly added to a solution of (*E*)-1-bromo-4-(1,1,1,2,5-pentafluoropent-3-en-2-yl)benzene (**2a)** (63.0 mg, 0.2 mmol, 1.0 eq.) in anhydrous DCM (2 mL). The reaction mixture was heated to reflux and stirred under an argon atmosphere for 24 h. The reaction was quenched with saturated sodium thiosulfate solution and the aqueous layer was extracted with DCM (3 × 30 mL). The combined organic layers were dried over Na_2SO_4, filtered, and concentrated under reduced pressure. The crude residue was purified by column chromatography (*n*-pentane:DCM 150:1) to yield the title compound as a colorless liquid (48.0 mg, 0.12 mmol, 61%, *d.r.* = 1:1.1).

R*f* = 0.61 (*n*-pentane:DCM 10:1).

FT-IR ($\tilde{\nu}$ = cm^{-1})**:** 3457 (w), 3015 (m), 2970 (s), 2946 (m), 2574 (w), 2131 (w), 1739 (s), 1593 (m), 1490 (m), 1436 (m), 1366 (s), 1267 (m), 1228 (s), 1216 (s), 1092 (m), 1077 (m),

1023 (m), 1011 (m), 956 (m), 931 (m), 895 (m), 821 (s), 735 (m), 696 (m), 628 (m), 527 (s), 514 (s).

^{1}H NMR (500 MHz, $CDCl_3$): δ [ppm] = 7.69 – 7.54 (m, 2H, H-C2), 7.44 – 7.31 (m, 2H, H-C3), 7.11 – 6.81 (m, 1H, H-C9), 6.54 – 6.28 (m, 2H, H-C7, H-C8).

^{13}C NMR (126 MHz, $CDCl_3$): δ [ppm] = 133.9 – 132.2 (m, C8, C4), 133.4 – 132.0 (m, C8), 132.22 (d, $^4J_{CF}$ = 1.1 Hz, C2), 132.21 (d, $^4J_{CF}$ = 1.2 Hz, C2), 128.0 – 127.5 (m, C3), 125.2 – 124.8 (m, C7), 124.8 – 124.6 (m, C1), 122.44 (dq, $^1J_{CF}$ = 285.1 Hz, $^2J_{CF}$ = 30.5 Hz, C6), 122.41 (dq, $^1J_{CF}$ = 285.6 Hz, $^2J_{CF}$ = 30.6 Hz, C6), 93.1 (dq, $^1J_{CF}$ = 191.9 Hz, $^2J_{CF}$ = 32.2 Hz, C5), 88.24 (d, $^1J_{CF}$ = 253.8 Hz, C9), 88.15 (d, $^1J_{CF}$ = 254.2 Hz, C9).

^{19}F NMR (470 MHz, $CDCl_3$): δ [ppm] = -79.08 (d, $^3J_{FF}$ = 8.4 Hz, 3F, F-C6), -79.21 (d, $^3J_{FF}$ = 8.4 Hz, 3F, F-C6), -139.07 – -139.44 (m, 1F, F-C5), -139.69 – -140.18 (m, 1F, F-C5), -169.64 – -169.75 (m, 2F, F-C9).

^{19}F{^{1}H} NMR (470 MHz, $CDCl_3$): δ [ppm] = -79.08 (d, $^3J_{FF}$ = 8.4 Hz, 3F, F-C6), -79.21 (d, $^3J_{FF}$ = 8.4 Hz, 3F, F-C6), -139.24 (d, $^3J_{FF}$ = 2.0 Hz, 1F, F-C5), -139.97 (d, $^3J_{FF}$ = 2.1 Hz, 1F, F-C5), -169.66 – -169.73 (m, 2F, F-C9).

GC-EI-MS: (*m/z*) requires: $[(C_{11}H_7Br_2F_5)^+]$ = 391.8829, (*m/z*) found: $[(C_{11}H_7Br_2F_5)^+]$ = 391.8829.

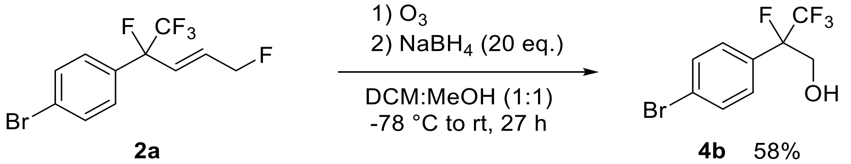

2a **4b** 58%

2-(4-Bromophenyl)-2,3,3,3-tetrafluoropropan-1-ol (4b)

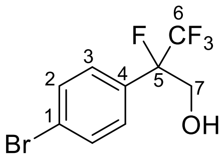

According to a modified literature procedure,[14] ozone was passed through a solution of (*E*)-1-bromo-4-(1,1,1,2,5-pentafluoropent-3-en-2-yl)benzene (**2a)** (63.0 mg, 0.2 mmol, 1.0 eq.) in a mixture of DCM (2 mL) and MeOH (2 mL) at -78 °C until the solution turned blue. The excess of O_3 was removed by an oxygen flow. Then, $NaBH_4$ (153.3 mg, 4.0 mmol, 20.0 eq.) was added as a

solid. The reaction was stirred at -78 °C for 5 minutes before warming to room temperature and further stirring for 27 hours. H_2O was then added and the mixture was extracted with DCM (3 × 30 mL). The combined organic layers were dried over Na_2SO_4, filtered, and concentrated under reduced pressure. The crude residue was purified by column chromatography (*n*-pentane:Et_2O 30:1) to yield the title compound as a colorless liquid (33.2 mg, 0.12 mmol, 58%).

R*f* = 0.42 (*n*-pentane:EtOAc 10:1).

FT-IR ($\tilde{\nu}$ = cm^{-1})**:** 3727 (w), 3311 (m), 2970 (m), 2942 (w), 2360 (w), 1739 (s), 1596 (w), 1495 (m), 1454 (m), 1402 (m), 1366 (m), 1313 (m), 1297 (m), 1271 (m), 1216 (s), 1197 (s), 1174 (s), 1138 (m), 1110 (m), 1077 (s), 1052 (m), 1011 (m), 972 (s), 946 (m), 900 (m), 820 (s), 745 (m), 728 (s), 698 (m), 631 (s), 580 (m), 542 (m).

^{1}H NMR (500 MHz, $CDCl_3$): δ [ppm] = 7.67 – 7.53 (m, 2H, H-C2), 7.44 – 7.33 (m, 2H, H-C3), 4.37 – 4.08 (m, 2H, H-C7), 1.93 (t, $^3J_{HH}$ = 7.1 Hz, 1H, OH).

^{13}C NMR (126 MHz, $CDCl_3$): δ [ppm] = 132.2 (C2), 130.7 (d, $^2J_{CF}$ = 22.4 Hz, C4), 127.8 (d, $^3J_{CF}$ = 8.7 Hz, C3), 124.4 (C1), 122.5 (dq, $^1J_{CF}$ = 285.5 Hz, $^2J_{CF}$ = 29.2 Hz, C6), 95.5 (dq, $^1J_{CF}$ = 189.0 Hz, $^2J_{CF}$ = 30.1 Hz, C5), 63.2 (d, $^2J_{CF}$ = 22.1 Hz, C7).

^{19}F NMR (470 MHz, $CDCl_3$): δ [ppm] = -77.71 (d, $^3J_{FF}$ = 7.4 Hz, 3F, F-C6), -179.71 (tq, $^3J_{HF}$ = 18.1 Hz, $^3J_{FF}$ = 7.3 Hz, 1F, F-C5).

^{19}F{^{1}H} NMR (470 MHz, $CDCl_3$): δ [ppm] = -77.71 (d, $^3J_{FF}$ = 7.4 Hz, 3F, F-C6), -179.71 (q, $^3J_{FF}$ = 7.3 Hz, 1F, F-C5).

GC-EI-MS: (*m/z*) requires: [$(C_9H_7OBrF_4)^+$] = 285.9611, (*m/z*) found: [$(C_{11}H_7Br_2F_5)^+$] = 285.9612.

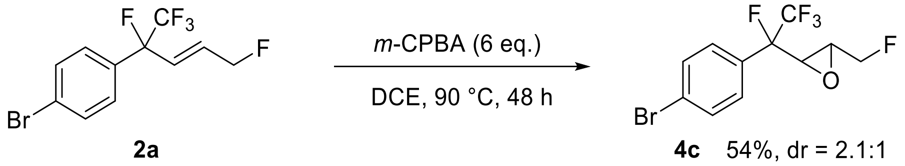

2a **4c** 54%, dr = 2.1:1

2-(1-(4-Bromophenyl)-1,2,2,2-tetrafluoroethyl)-3-(fluoromethyl)oxirane (4c)

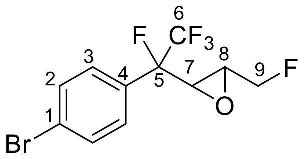

According to a modified literature procedure,[15] 3-chloroperoxybenzoic acid (268.2 mg, 1.2 mmol, 6.0 eq.) was added to a solution of (*E*)-1-bromo-4-(1,1,1,2,5-pentafluoropent-3-en-2-yl)benzene (**2a)** (63.0 mg, 0.2 mmol, 1.0 eq.) in 1,2-dichloroethane. The reaction mixture was heated to reflux and stirred under an argon atmosphere for 48 h. The reaction was quenched with saturated sodium thiosulfate solution and the aqueous layer was extracted with DCM (3 × 30 mL). The combined organic layers were dried over Na_2SO_4, filtered, and concentrated under reduced pressure. The crude residue was purified by column chromatography (*n*-pentane:DCM 15:1) to yield the title compound as a colorless liquid (35.7 mg, 0.11 mmol, 54%, *d.r.* = 2.1:1).

R*f* = 0.19 (*n*-pentane:DCM 15:1).

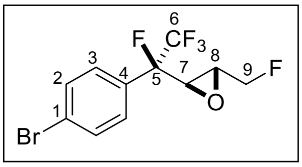

FT-IR ($\tilde{\nu}$ = cm^{-1})**:** 3457 (m), 3015 (s), 2970 (s), 2947 (m), 2574 (w), 2131 (w), 1739 (s), 1594 (m), 1492 (m), 1437 (s), 1367 (s), 1298 (m), 1216 (s), 1089 (m), 1011 (m), 959 (m), 938 (m), 916 (m), 878 (m), 822 (m), 789 (m), 746 (m), 732 (m), 696 (m), 653 (w), 619 (m), 587 (w), 539 (s), 527 (s), 514 (s).

^{1}H NMR (500 MHz, $CDCl_3$): δ [ppm] = 7.64 – 7.58 (m, 2H, H-C2), 7.45 – 7.39 (m, 2H, H-C3), 4.71 (ddd, $^{2}J_{HF}$ = 47.8 Hz, $^{2}J_{HH}$ = 11.3 Hz, $^{3}J_{HH}$ = 2.1 Hz, 1H, H^{a}-C9), 4.71 (ddd, $^{2}J_{HF}$ = 46.4 Hz, $^{2}J_{HH}$ = 11.3 Hz, $^{3}J_{HH}$ = 4.2 Hz, 1H, H^{b}-C9), 3.73 (dd, $^{3}J_{HF}$ = 16.6 Hz, $^{3}J_{HH}$ = 1.9 Hz, 1H, H-C7), 3.42 (dtd, $^{3}J_{HF}$ = 16.6 Hz, $^{3}J_{HH}$ = 4.2 Hz, $^{3}J_{HH}$ = 2.0 Hz, 1H, H-C8).

^{13}C NMR (126 MHz, $CDCl_3$): δ [ppm] = 132.1 (d, $^{4}J_{CF}$ = 1.6 Hz, C2), 129.7 (d, $^{2}J_{CF}$ = 22.5 Hz, C4), 127.8 (dq, $^{3}J_{CF}$ = 9.5 Hz, $^{4}J_{CF}$ = 1.4 Hz, C3), 125.0 (d, $^{5}J_{CF}$ = 1.7 Hz, C5), 122.4 (dq, $^{1}J_{CF}$ = 285.4 Hz, $^{2}J_{CF}$ = 29.5 Hz, C6), 91.8 (dq, $^{1}J_{CF}$ = 192.6 Hz, $^{2}J_{CF}$ = 32.0 Hz, C5), 80.5 (dd, $^{1}J_{CF}$ = 174.2 Hz, $^{4}J_{CF}$ = 0.9 Hz, C9), 53.47 – 53.24 (m, C7), 53.22 – 53.00 (m, C8).

^{19}F NMR (470 MHz, $CDCl_3$): δ [ppm] = -78.55 (d, $^3J_{FF}$ = 8.4 Hz, 3F, F-C6), -181.63 (dq, $^2J_{FF}$ = 16.8 Hz, $^3J_{FF}$ = 8.4 Hz, 1F, F-C5), -231.45 – -231.87 (m, 1F, F-C9).

^{19}F{^{1}H} NMR (470 MHz, $CDCl_3$): δ [ppm] = -78.55 (d, $^3J_{FF}$ = 8.4 Hz, 3F, F-C6), -181.63 (q, $^3J_{FF}$ = 8.5 Hz, 1F, F-C5), -231.67 (s, 1F, F-C9).

GC-EI-MS: (*m/z*) requires: $[(C_{11}H_8BrOF_5)^+]$ = 329.9673, (*m/z*) found: $[(C_{11}H_8BrOF_5)^+]$ = 329.9673.

The diastereomers were assigned by analogy to previously reported similar structures.[16]

R*f* = 0.14 (*n*-pentane:DCM 15:1).

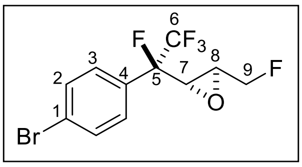

FT-IR ($\tilde{\nu}$ = cm^{-1})**:** 3457 (m), 3015 (s), 2970 (s), 2947 (m), 2574 (w), 2131 (w), 1739 (s), 1594 (m), 1493 m), 1437 (s), 1366 (s), 1366 (s), 1295 (m), 1228 (s), 1216 (s), 1157 (m), 1090 (m), 1077 (m), 1010 (m), 933 (m), 912 (m), 879 (m), 822 (m), 788 (m), 763 (m), 725 (m), 703 (w), 688 (w), 617 (m), 527 (s), 514 (s).

^{1}H NMR (500 MHz, $CDCl_3$): δ [ppm] = 7.68 – 7.57 (m, 2H, H-C2), 7.38 (d, $^3J_{HH}$ = 8.4 Hz, 2H, H-C3), 4.61 (ddd, $^2J_{HF}$ = 48.1 Hz, $^2J_{HH}$ = 11.3 Hz, $^3J_{HH}$ = 2.1 Hz, 1H, H^a-C9), 4.71 (ddd, $^2J_{HF}$ = 46.2 Hz, $^2J_{HH}$ = 11.3 Hz, $^3J_{HH}$ = 4.1 Hz, 1H, H^b-C9), 3.73 (dd, $^3J_{HF}$ = 18.1 Hz, $^3J_{HH}$ = 1.6 Hz, 1H, H-C7), 3.42 (dtd, $^3J_{HF}$ = 16.6 Hz, $^3J_{HH}$ = 4.3 Hz, $^3J_{HH}$ = 2.2 Hz, 1H, H-C8).

^{13}C NMR (126 MHz, $CDCl_3$): δ [ppm] = 132.3 (d, $^4J_{CF}$ = 1.6 Hz, C2), 130.4 (d, $^2J_{CF}$ = 22.4 Hz, C4), 127.8 (dq, $^3J_{CF}$ = 9.5 Hz, $^4J_{CF}$ = 1.5 Hz, C3), 125.0 (d, $^5J_{CF}$ = 1.5 Hz, C5), 122.4 (dq, $^1J_{CF}$ = 286.0 Hz, $^2J_{CF}$ = 31.1 Hz, C6), 91.8 (dq, $^1J_{CF}$ = 195.0 Hz, $^2J_{CF}$ = 31.1 Hz, C5), 80.5 (dd, $^1J_{CF}$ = 174.3 Hz, $^4J_{CF}$ = 0.9 Hz, C9), 127.8 (dd, $^2J_{CF}$ = 21.2 Hz, $^4J_{CF}$ = 8.8 Hz, C7), 127.8 (dd, $^2J_{CF}$ = 23.0 Hz, $^4J_{CF}$ = 6.8 Hz, C8).

^{19}F NMR (376 MHz, $CDCl_3$): δ [ppm] = -77.38 (d, $^3J_{FF}$ = 7.9 Hz, 3F, F-C6), -185.10 – -185.27 (m, 1F, F-C5), -230.89 – -231.68 (m, 1F, F-C9).

^{19}F{^{1}H} NMR (376 MHz, $CDCl_3$): δ [ppm] = -77.38 (d, $^{3}J_{FF}$ = 7.9 Hz, 3F, F-C6), -185.16 (q, $^{3}J_{FF}$ = 7.9 Hz, 1F, F-C5), -231.29 (s, 1F, F-C9).

GC-EI-MS: (*m/z*) requires: [$(C_{11}H_8BrOF_5)^+$] = 329.9673, (*m/z*) found: [$(C_{11}H_8BrOF_5)^+$] = 329.9672.

The diastereomers were assigned by analogy to previously reported similar structures.[16]

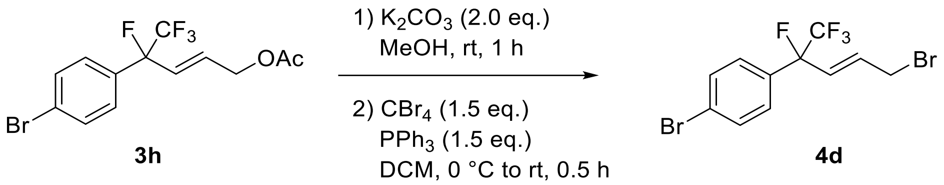

3h **4d**

(*E*)-1-Bromo-4-(5-bromo-1,1,1,2-tetrafluoropent-3-en-2-yl)benzene (4d)

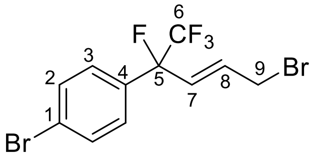

According to a modified literature procedure,[17] K_2CO_3 (56.2 mg, 0.4 mmol, 2.0 eq.) was added to a solution of (*E*)-4-(4-bromophenyl)-4,5,5,5-tetrafluoropent-2-en-1-yl (**3h)** (72.3 mg, 0.2 mmol, 1.0 eq.) in methanol . The reaction mixture was allowed to stir at room temperature for 1 h. H_2O was added and the mixture was extracted with EtOAc (3 × 30 mL). The combined organic layers were dried over Na_2SO_4, filtered, and concentrated under reduced pressure. The crude residue was used directly in the next step without further purification.

The crude residue obtained in the last step was dissolved in anhydrous DCM, followed by the addition of CBr_4 (99.7 mg, 0.3 mmol, 1.5 eq.).PPh_3 (81.3 mg, 0.3 mmol, 1.5 eq.) was then added portion-wise at 0 °C under argon. The reaction mixture was allowed to stir at room temperature for 0.5 h, then directly concentrated under reduced pressure. The crude residue was purified by column chromatography (*n*-pentane:DCM 200:1) to yield the title compound as a colorless liquid (75.2 mg, 0.20 mmol, 98% over two steps).

R*f* = 0.65 (*n*-pentane:DCM 10:1).

FT-IR ($\tilde{\nu}$ = cm^{-1})**:** 3457 (m), 3015 (m), 2970 (s), 2946 (m), 2574 (w), 2130 (w), 1739 (s), 1592 (w), 1489 (m), 1437 (s), 1366 (s), 1283 (m), 1272 (m), 1228 (s), 1216 (s), 1131 (m), 1092 (m), 1076 (m), 1035 (w), 1011 (m), 952 (m), 936 (m), 895 (m), 822 (s), 768 (m), 728 (m), 690 (m), 626 (m), 583 (m), 538 (m), 527 (s), 513 (s).

^{1}H NMR (500 MHz, $CDCl_3$): δ [ppm] = 7.74 – 7.49 (m, 2H, H-C2), 7.37 (d, $^3J_{HH}$ = 8.3 Hz, 2H, H-C3), 6.45 – 6.03 (m, 2H, H-C7, H-C8), 4.00 (dd, $^3J_{HH}$ = 7.1 Hz, $^5J_{HF}$ = 1.2 Hz, 2H, H-C9).

^{13}C NMR (126 MHz, $CDCl_3$): δ [ppm] = 132.9 (d, $^3J_{CF}$ = 11.6 Hz, C8), 132.8 (d, $^2J_{CF}$ = 22.4 Hz, C4), 132.0 (d, $^4J_{CF}$ = 1.4 Hz, C2), 127.8 (dq, $^3J_{CF}$ = 8.7 Hz, $^4J_{CF}$ = 1.3 Hz, C3), 126.6 (d, $^2J_{CF}$ = 18.1 Hz, C7), 124.4 (d, $^5J_{CF}$ = 1.7 Hz, C1), 122.5 (dq, $^1J_{CF}$ = 285.2 Hz, $^2J_{CF}$ = 30.7 Hz, C6), 93.3 (dq, $^1J_{CF}$ = 189.8 Hz, $^2J_{CF}$ = 32.4 Hz, C5), 29.9 (C9).

^{19}F NMR (470 MHz, $CDCl_3$): δ [ppm] = -79.21 (d, $^3J_{FF}$ = 8.8 Hz, 3F, F-C6), -167.00 (dq, $^3J_{FH}$ = 17.4 Hz, $^3J_{FF}$ = 8.8 Hz, 1F, F-C5).

^{19}F{^{1}H} NMR (470 MHz, $CDCl_3$): δ [ppm] = -79.21 (d, $^3J_{FF}$ = 8.8 Hz, 3F, F-C6), -167.00 (q, $^3J_{FF}$ = 8.8 Hz, 1F, F-C5).

GC-EI-MS: (*m/z*) requires: $[(C_{11}H_8Br_2F_4)^+]$ = 373.8923, (*m/z*) found: $[(C_{11}H_7Br_2F_5)^+]$ = 373.8926.

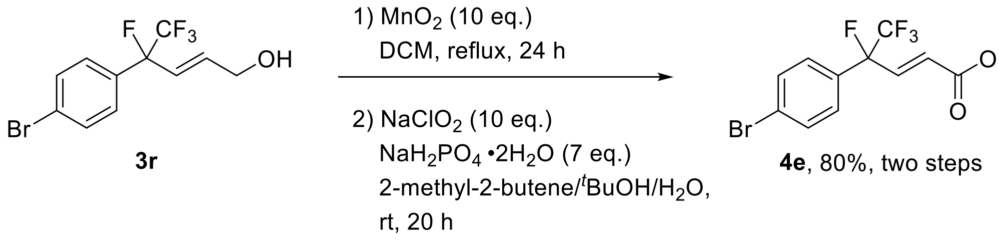

(*E*)-4-(4-Bromophenyl)-4,5,5,5-tetrafluoropent-2-enoic acid (5c)

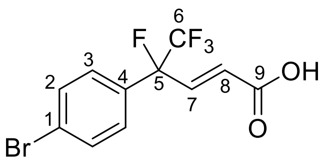

According to a modified literature procedure,[18] activated MnO_2 (10 eq.) was added to a solution of (*E*)-4-(4-bromophenyl)-4,5,5,5-tetrafluoropent-2-en-1-ol **(3r)** (62.6 mg, 0.2 mmol, 1.0 eq.) in anhydrous CH_2Cl_2 (3 mL) at room

temperature. The reaction mixture was then heated to reflux and stirred for at least 24 h, while being monitored by TLC. After complete consumption of the starting material, the reaction mixture was filtered through a pad of celite and rinsed by CH_2Cl_2. The resulting filtrate was concentrated under reduced pressure. The crude residue was directly used in the next step without further purification.

The crude residue obtained in the last step was dissolved in 2-methyl-2-butene (1 ml, 0.2 M), and ^{t}BuOH (4 ml, 0.05 M) was added. Then, a solution of $NaClO_2$ (90.4 mg, 2.0 mmol, 10.0 eq.) and NaH_2PO_4 dihydrate (156.0 mg, 1.4 mmol, 7.0 eq.) in H_2O (2.0 ml, 0.1 M) was added dropwise. The mixture was stirred at room temperature overnight. The reaction mixture was diluted with EtOAc and the organic layer was washed once with 1 M HCl. The aqueous layer was extracted with EtOAc (3 × 30 mL). The combined organic layers were dried over Na_2SO_4, filtered, and concentrated under reduced pressure. The crude residue was purified by column chromatography (*n*-pentane: EtOAc 3:1) to yield the title compound as a colorless liquid (51.9 mg, 0.16 mmol, 80%, two steps).

R*f* = 0.51 (*n*-pentane: EtOAc 1:1).

FT-IR ($\tilde{\nu}$ = cm^{-1})**:** 2927 (w), 1704 (s), 1662 (m), 1593 (w), 1494 (w), 1420 (m), 1401 (m), 1270 (m), 1180 (s), 1162 (m), 1116 (m), 1075 (m), 1030 (m), 1011 (m), 977 (m), 957 (m), 907 (m), 865 (m), 821 (m), 783 (m), 734 (m), 703 (m), 672 (w), 639 (m), 598 (m).

^{1}H NMR (500 MHz, $CDCl_3$): δ [ppm] = 7.65 – 7.56 (m, 2H, H-C2), 7.43 – 7.37 (m, 2H, H-C3), 7.33 (dd, $^3J_{HF}$ = 20.5 Hz, $^3J_{HH}$ = 15.6 Hz, 1H, H-C7), 6.39 (d, $^3J_{HH}$ = 15.6 Hz, 1H, H-C8).

^{13}C NMR (126 MHz, $CDCl_3$): δ [ppm] = 170.1 (C9), 140.0 (d, $^2J_{CF}$ = 18.1 Hz, C7), 132.4 (d, $^4J_{CF}$ = 1.4 Hz, C2), 131.5 (d, $^2J_{CF}$ = 22.1 Hz, C4), 127.6 (dq, $^3J_{CF}$ = 9.0 Hz, $^4J_{CF}$ = 1.4 Hz, C3), 125.0 (C8), 124.9 (d, $^5J_{CF}$ = 1.6 Hz, C1), 122.0 (dq, $^1J_{CF}$ = 285.4 Hz, $^2J_{CF}$ = 30.3 Hz, C6), 93.5 (dq, $^1J_{CF}$ = 193.6 Hz, $^2J_{CF}$ = 33.0 Hz, C5).

^{19}F NMR (470 MHz, $CDCl_3$): δ [ppm] = -78.79 (d, $^3J_{FF}$ = 8.2 Hz, 3F, F-C6), -172.04 (dq, $^3J_{FH}$ = 20.4 Hz, $^3J_{FF}$ = 8.2 Hz, 1F, F-C5).

^{19}F{^{1}H} NMR (470 MHz, $CDCl_3$): δ [ppm] = -78.79 (d, $^{3}J_{FF}$ = 8.2 Hz, 3F, F-C6), -172.04 (q, $^{3}J_{FF}$ = 8.2 Hz, 1F, F-C5).

ESI-MS: (*m/z*) requires: $[(C_{11}H_7BrF_4O_2\text{-}H)^-]$ = 324.9493, (*m/z*) found: $[(C_{11}H_7BrF_4O_2\text{-}H)^-]$ = 324.9497.

6. X-ray crystallographic data

X-Ray diffraction: Data sets for compound **2t** were collected with a Bruker D8 Venture Photon III Diffractometer. Programs used: data collection: *APEX4* Version 2021.4-0[19] (Bruker AXS Inc., **2021**); cell refinement: *SAINT* Version 8.40B (Bruker AXS Inc., **2021**); data reduction: *SAINT* Version 8.40B (Bruker AXS Inc., **2021**); absorption correction, *SADABS* Version 2016/2 (Bruker AXS Inc., **2021**); structure solution *SHELXT*-Version 2018-3[20] (Sheldrick, G. M. *Acta Cryst.,* **2015**, *A71*, 3-8); structure refinement *SHELXL*-Version 2018-3[21] (Sheldrick, G. M. *Acta Cryst.,* **2015**, *C71* (1), 3-8) and graphics, *XP*[22] (Version 5.1, Bruker AXS Inc., Madison, Wisconsin, USA, **1998**). *R*-values are given for observed reflections, and wR^2 values are given for all reflections.

Exceptions and special features: For compound **2t** the fluorine substituent and the CF_3 group on the benzylic carbon were found disordered over two positions in the asymmetric unit. Several restraints (SADI, SAME, ISOR and SIMU) were used in order to improve refinement stability.

X-ray crystal structure analysis of 2t (gil10343): A colorless, prism-like specimen of $C_{19}H_{12}F_5NO_2$, approximate dimensions 0.058 mm x 0.100 mm x 0.240 mm, was used for the X-ray crystallographic analysis. The X-ray intensity data were measured on a single crystal diffractometer Bruker D8 Venture Photon III system equipped with a micro focus tube Cu ImS (CuKα, λ = 1.54178 Å) and a MX mirror monochromator. A total of 1951 frames were collected. The total exposure time was 23.80 hours. The frames were integrated with the Bruker SAINT software package using a wide-frame algorithm. The integration of the data using a monoclinic unit cell yielded a total of 44279 reflections to a maximum θ angle of 66.62° (0.84 Å resolution), of which 2894 were independent (average redundancy 15.300, completeness = 99.7%, R_{int} = 7.11%, R_{sig} = 2.43%) and 2393 (82.69%) were greater than $2\sigma(F^2)$. The final cell constants of a = 17.4415(4) Å, b = 6.57700(10) Å, c = 15.9589(3) Å, β = 116.4470(10)°, volume = 1639.10(6) $Å^3$, are based upon the refinement of the XYZ-centroids of 9956 reflections above 20 σ(I) with 5.659° < 2θ < 133.0°. Data were corrected for absorption effects using the Multi-Scan method (SADABS). The ratio of minimum to maximum apparent transmission was 0.823. The calculated minimum and

maximum transmission coefficients (based on crystal size) are 0.7590 and 0.9330. The structure was solved and refined using the Bruker SHELXTL Software Package, using the space group $P2_1/c$, with Z = 4 for the formula unit, $C_{19}H_{12}F_5NO_2$. The final anisotropic full-matrix least-squares refinement on F^2 with 284 variables converged at R1 = 3.93%, for the observed data and wR2 = 10.06% for all data. The goodness-of-fit was 1.030. The largest peak in the final difference electron density synthesis was 0.427 $e^-/Å^3$ and the largest hole was -0.287 $e^-/Å^3$ with an RMS deviation of 0.049 $e^-/Å^3$. On the basis of the final model, the calculated density was 1.545 g/cm^3 and F(000), 776 e^-. CCDC Nr.: 2194476.

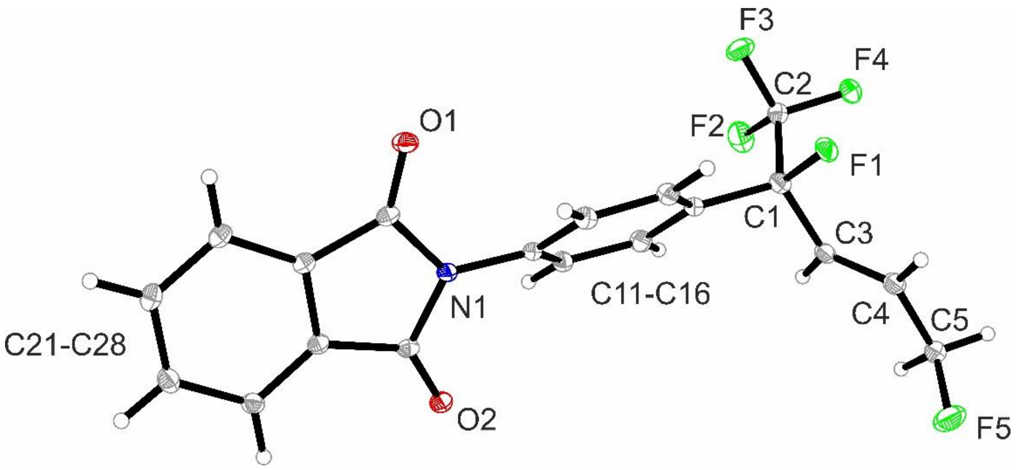

Figure S1: Crystal structure of compound **2t**.

Thermal ellipsoids are shown at 30% probability.

7. NMR-Spectra of key compounds

Ethyl (*Z*)-3-(4-bromophenyl)-4,4,4-trifluorobut-2-enoate

^{1}H NMR (599 MHz, $CDCl_3$)

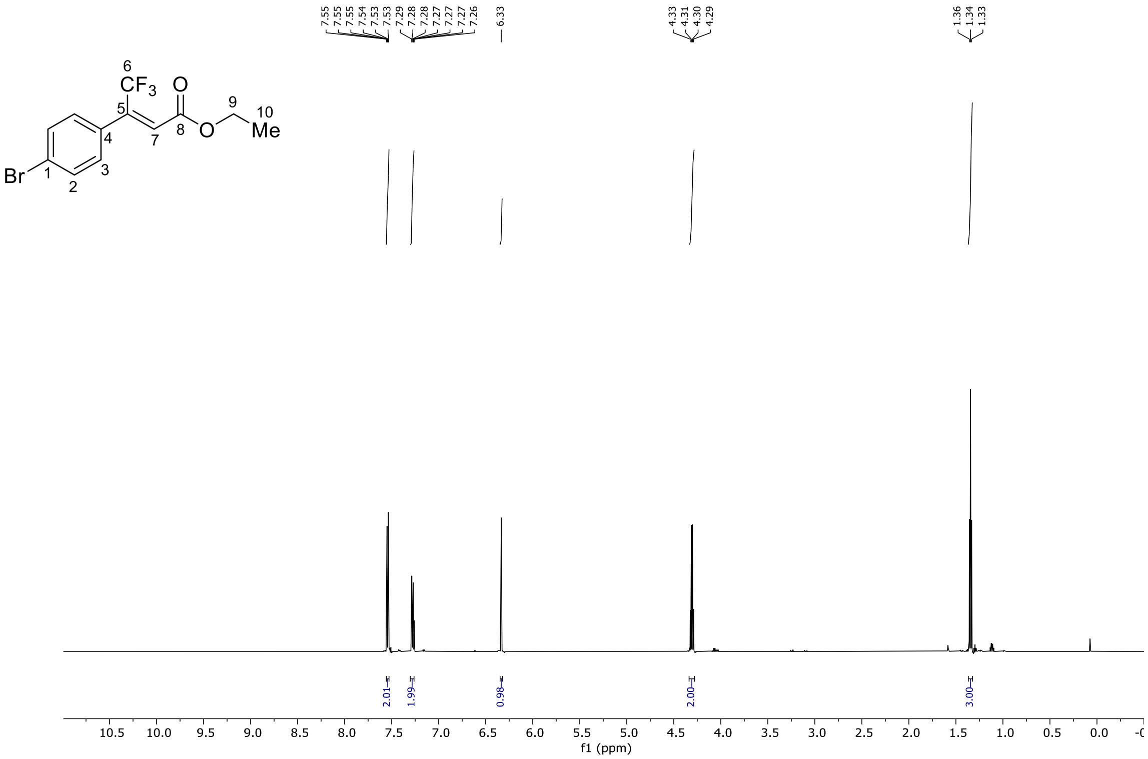

^{13}C NMR (151 MHz, $CDCl_3$)

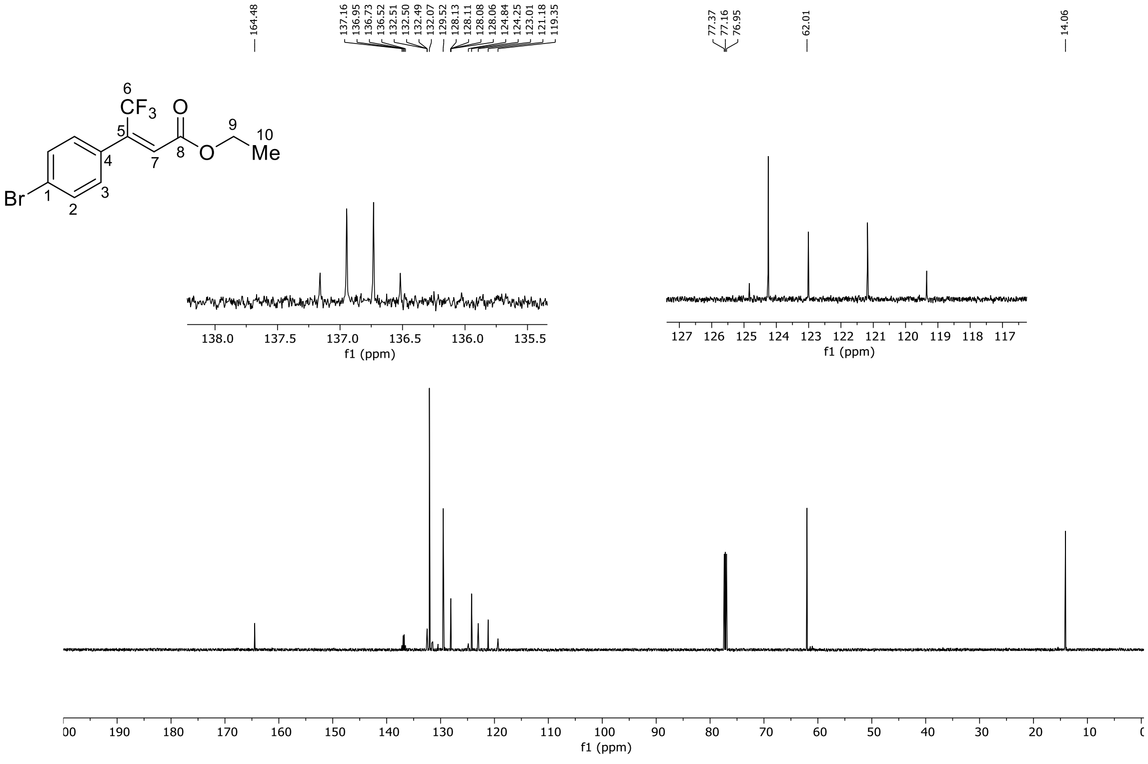

^{19}F NMR (564 MHz, $CDCl_3$)

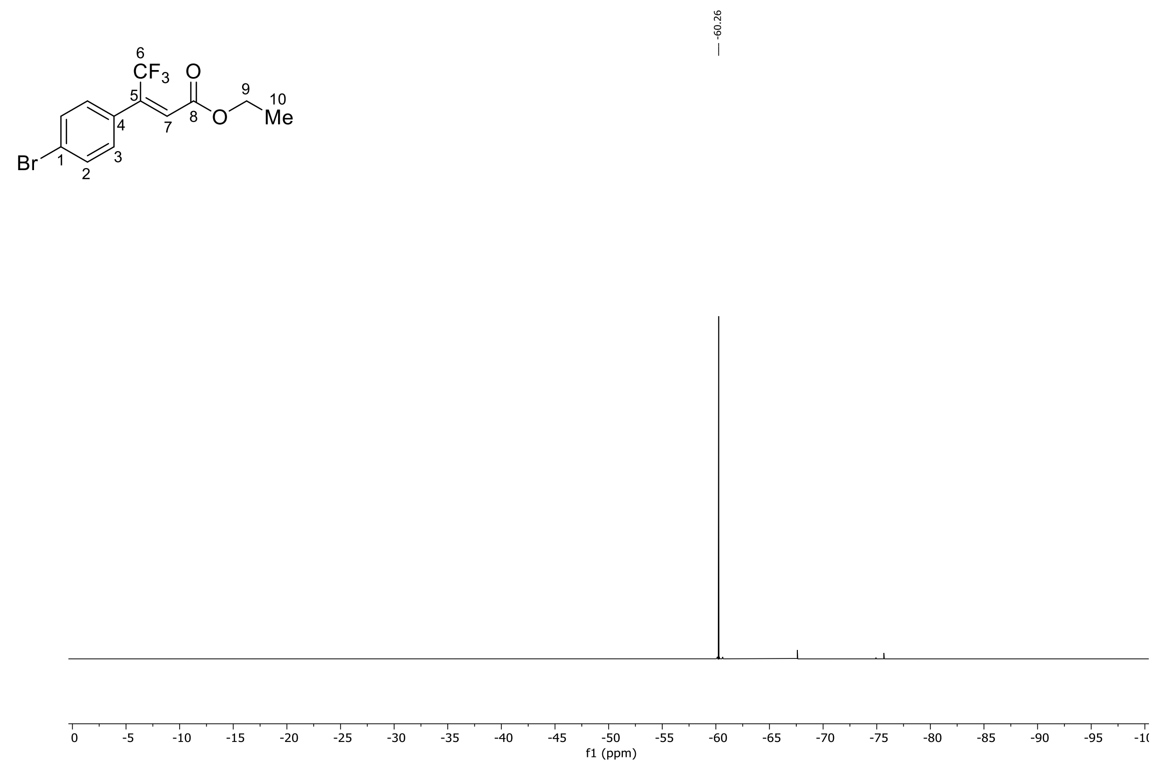

^{19}F{^{1}H} NMR (564 MHz, $CDCl_3$)

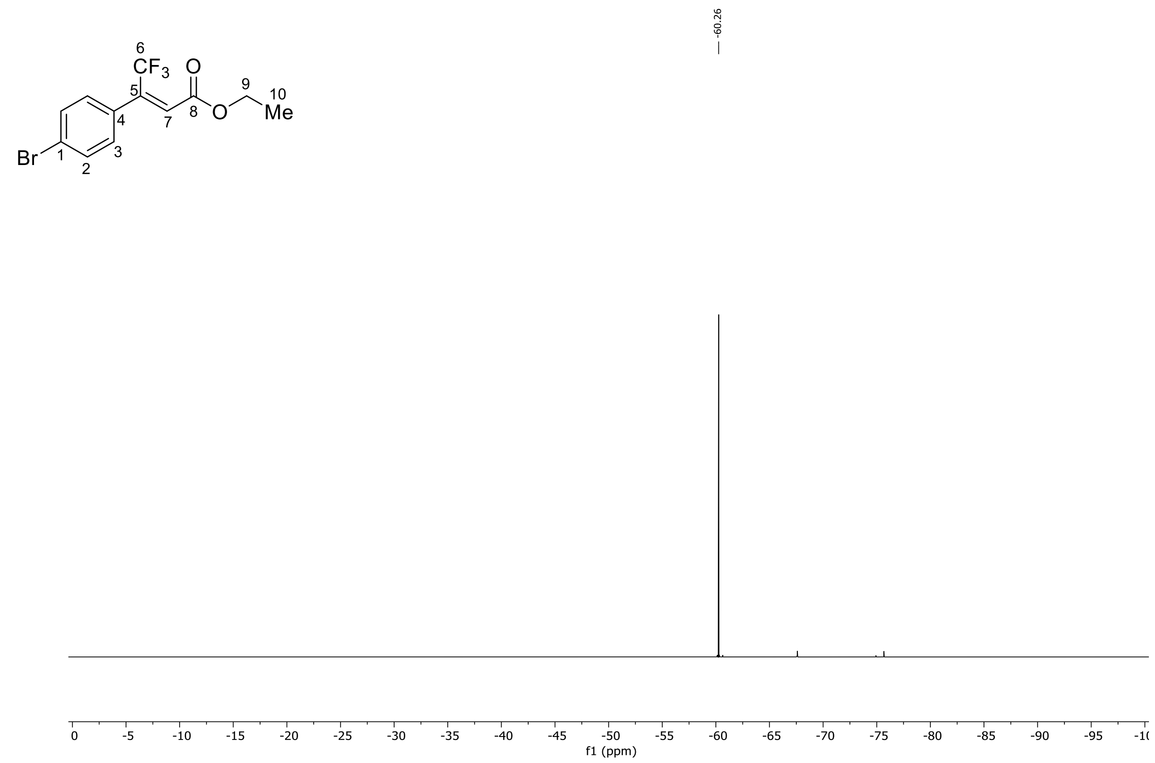

(*Z*)-1-Bromo-4-(1,1,1-trifluoropenta-2,4-dien-2-yl)benzene (*Z*-1a)

^{1}H NMR (599 MHz, $CDCl_3$)

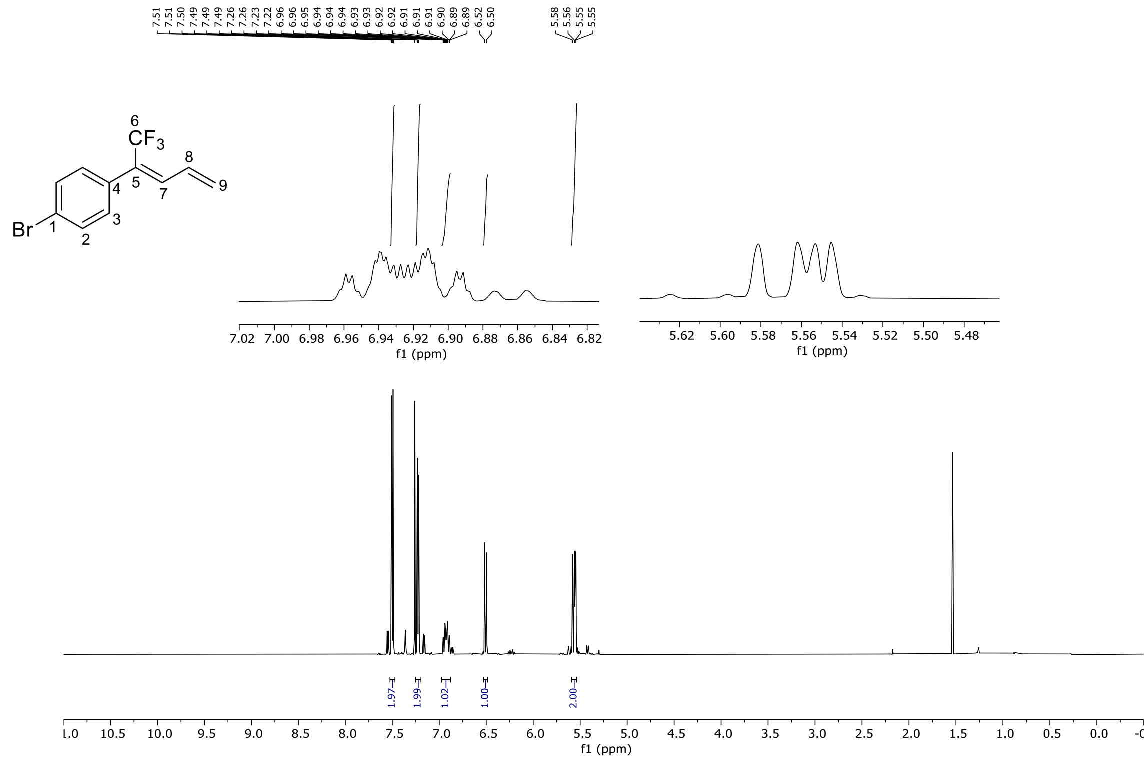

^{13}C NMR (151 MHz, $CDCl_3$)

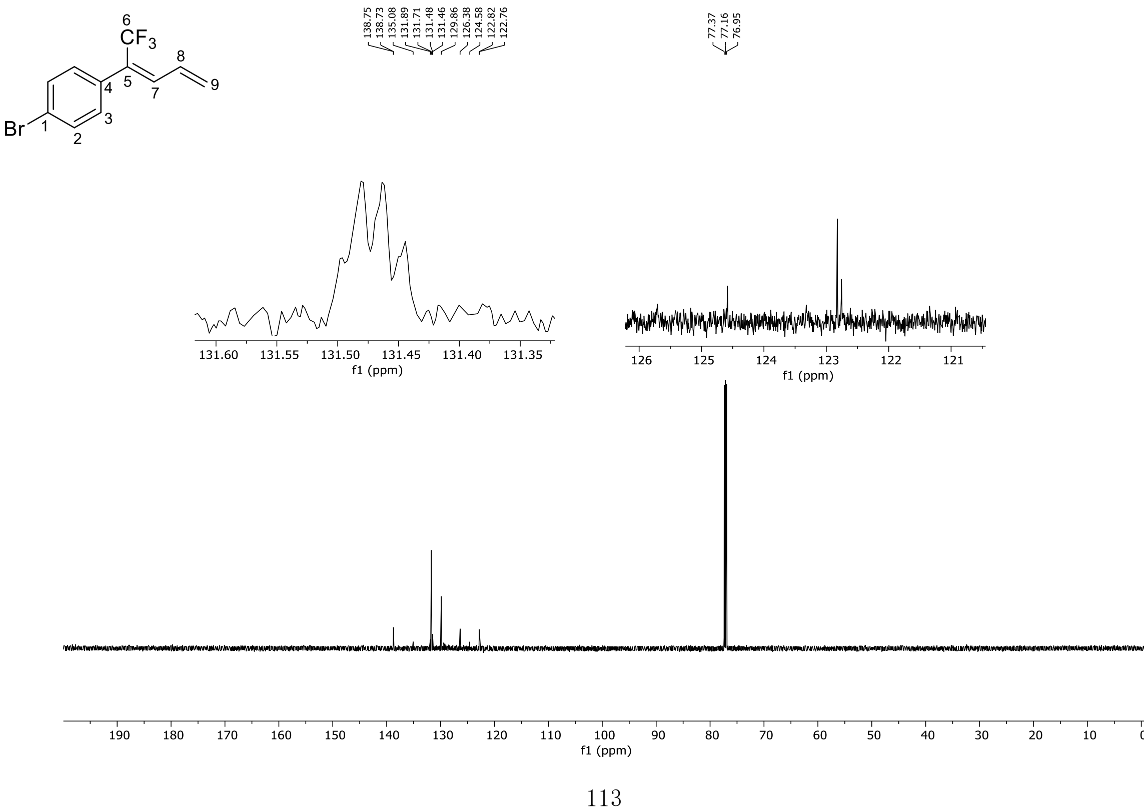

^{19}F NMR (564 MHz, $CDCl_3$)

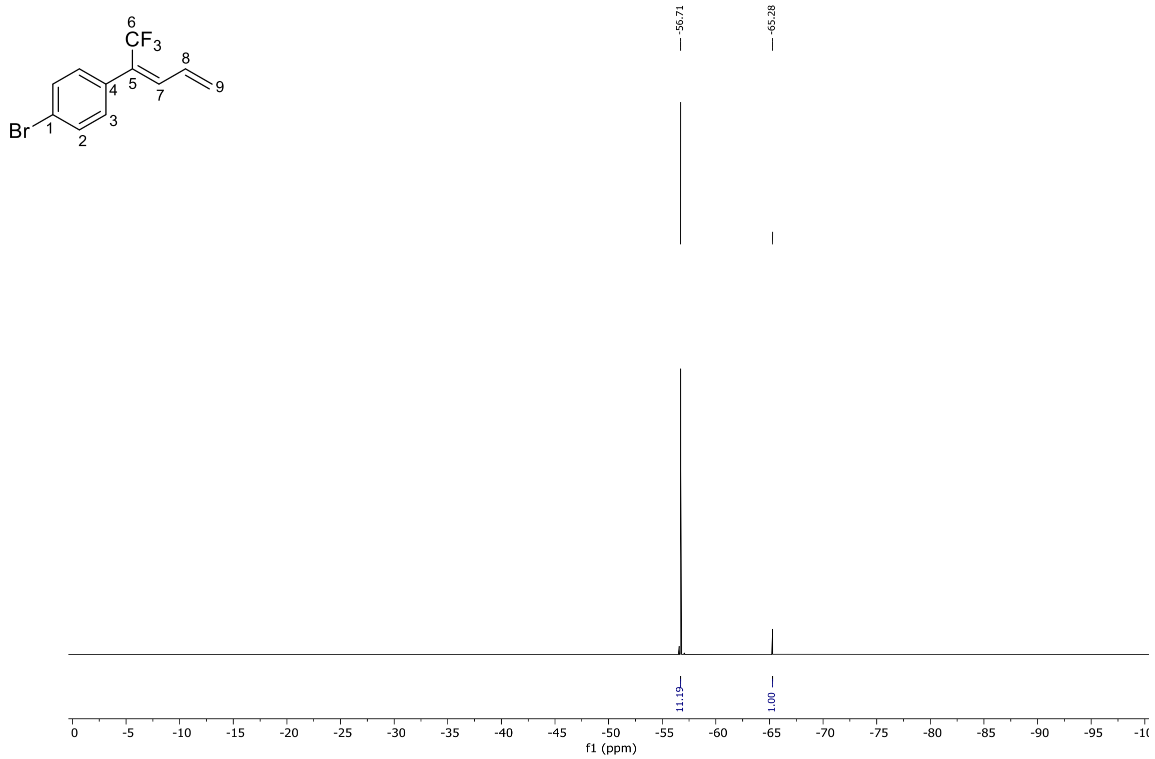

^{19}F{^{1}H} NMR (564 MHz, $CDCl_3$)

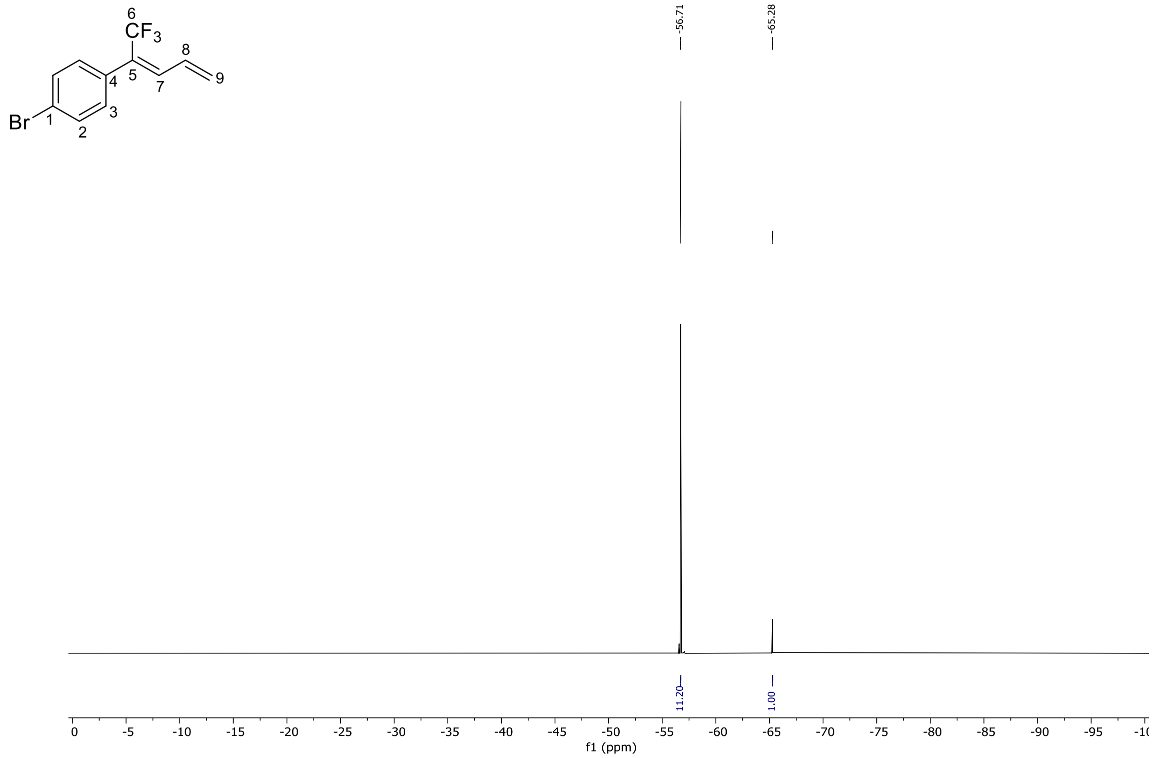

1-Bromo-4-(1,1,1-trifluoropenta-2,4-dien-2-yl)benzene (1a)

^{1}H NMR (500 MHz, $CDCl_3$)

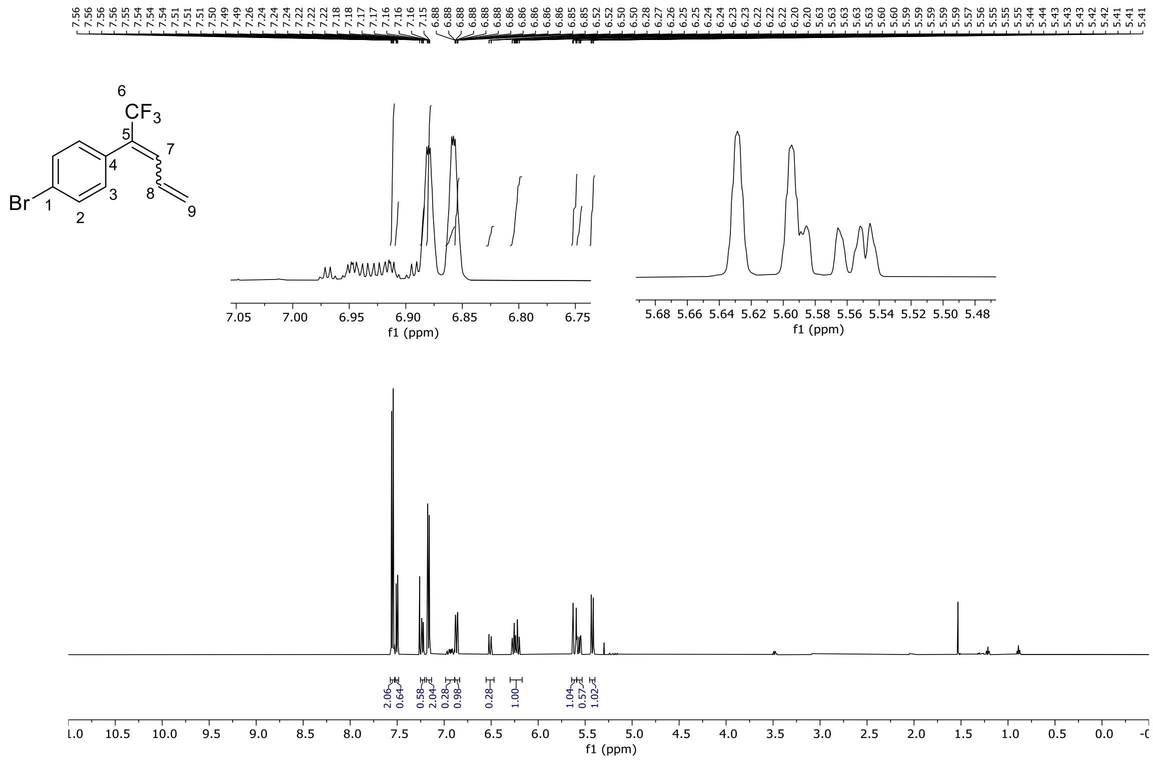

^{13}C NMR (126 MHz, $CDCl_3$)

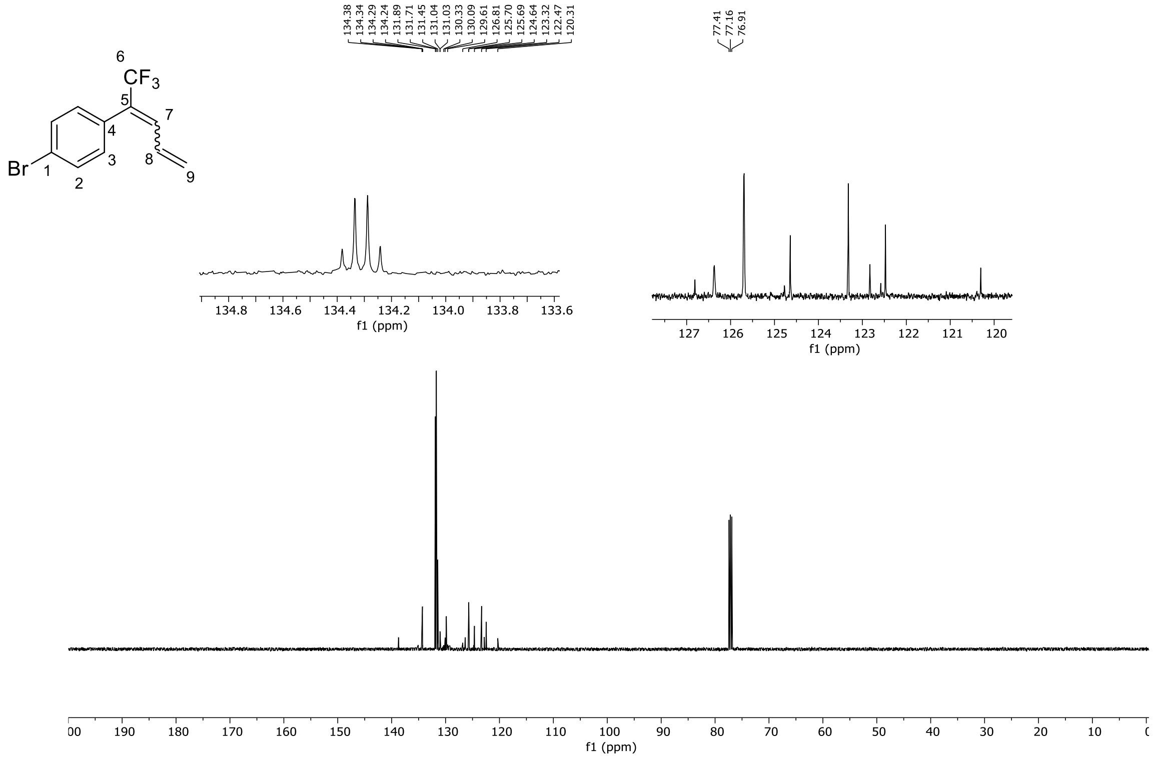

^{19}**F NMR** (470 MHz, $CDCl_3$)

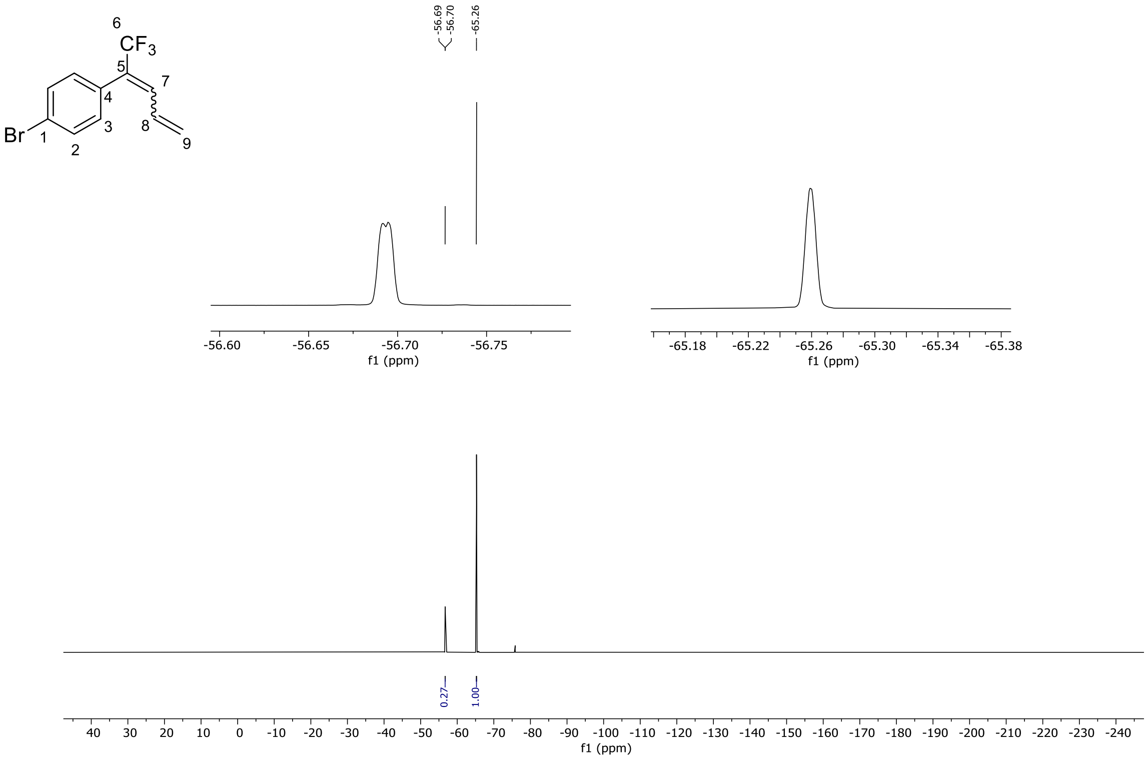

^{19}**F{^{1}H} NMR** (470 MHz, $CDCl_3$)

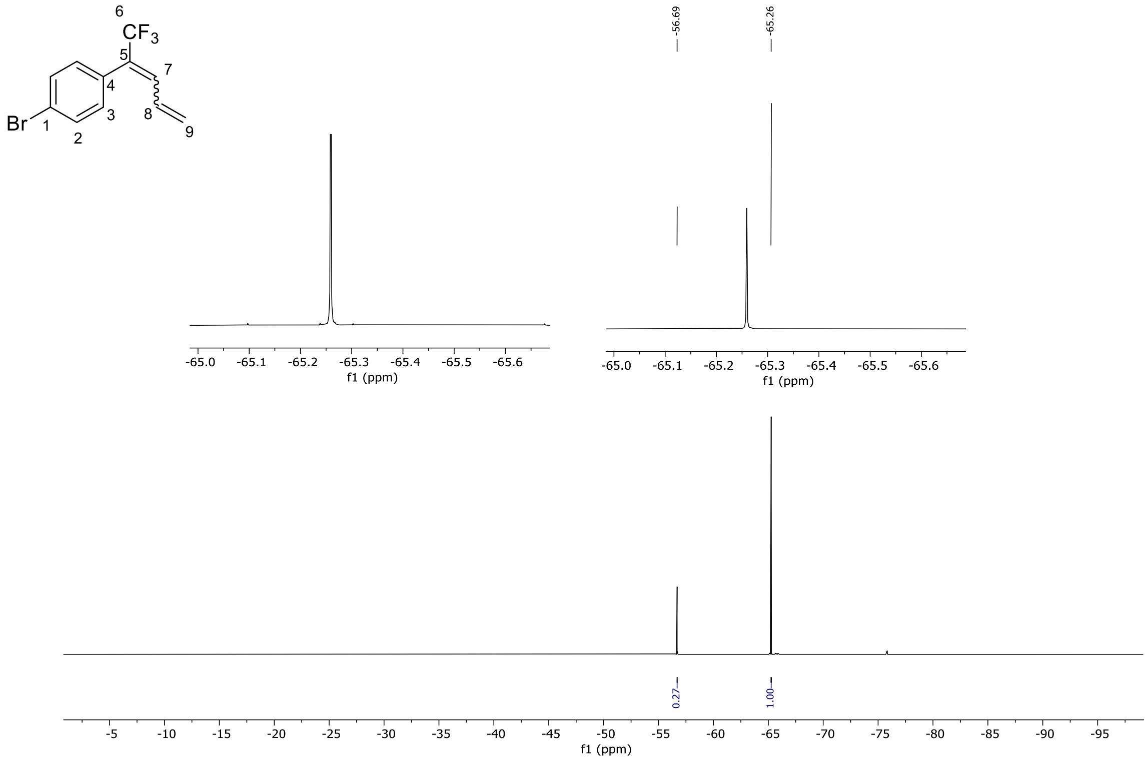

2-(3-Bromophenyl)-1,1,1-trifluoropent-4-en-2-ol (1b-1)

^{1}H NMR (599 MHz, $CDCl_3$)

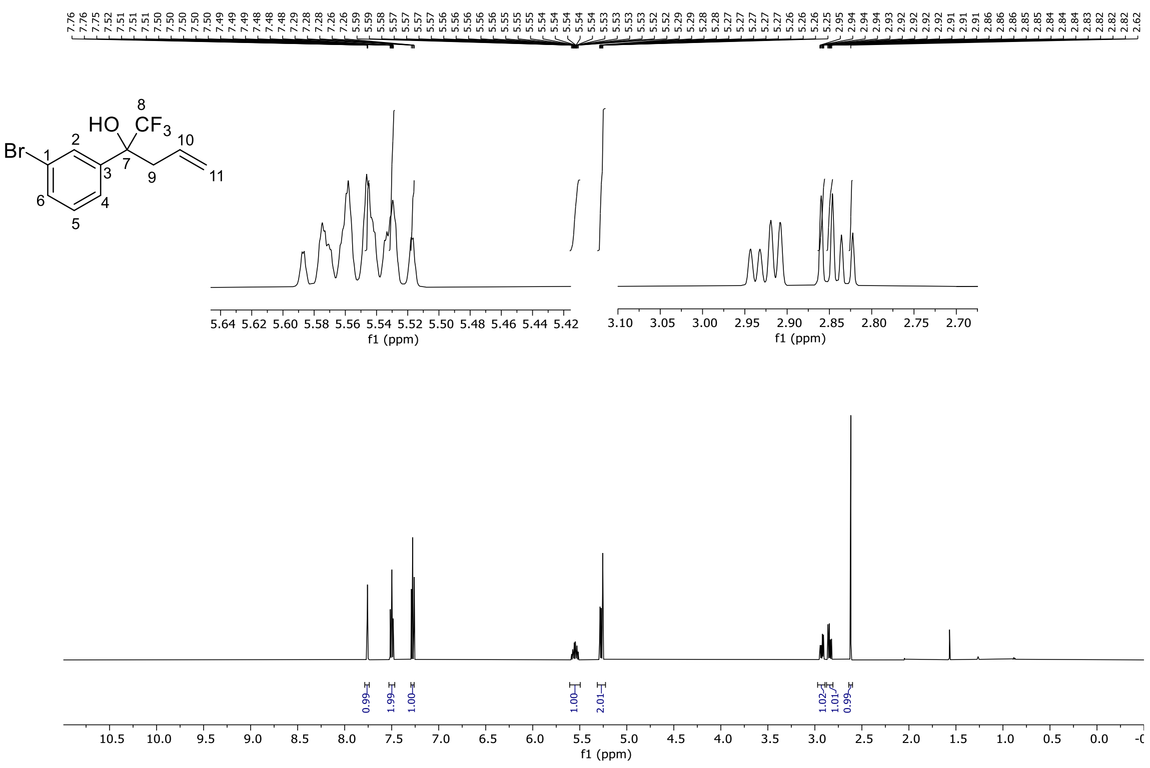

^{13}C NMR (151 MHz, $CDCl_3$)

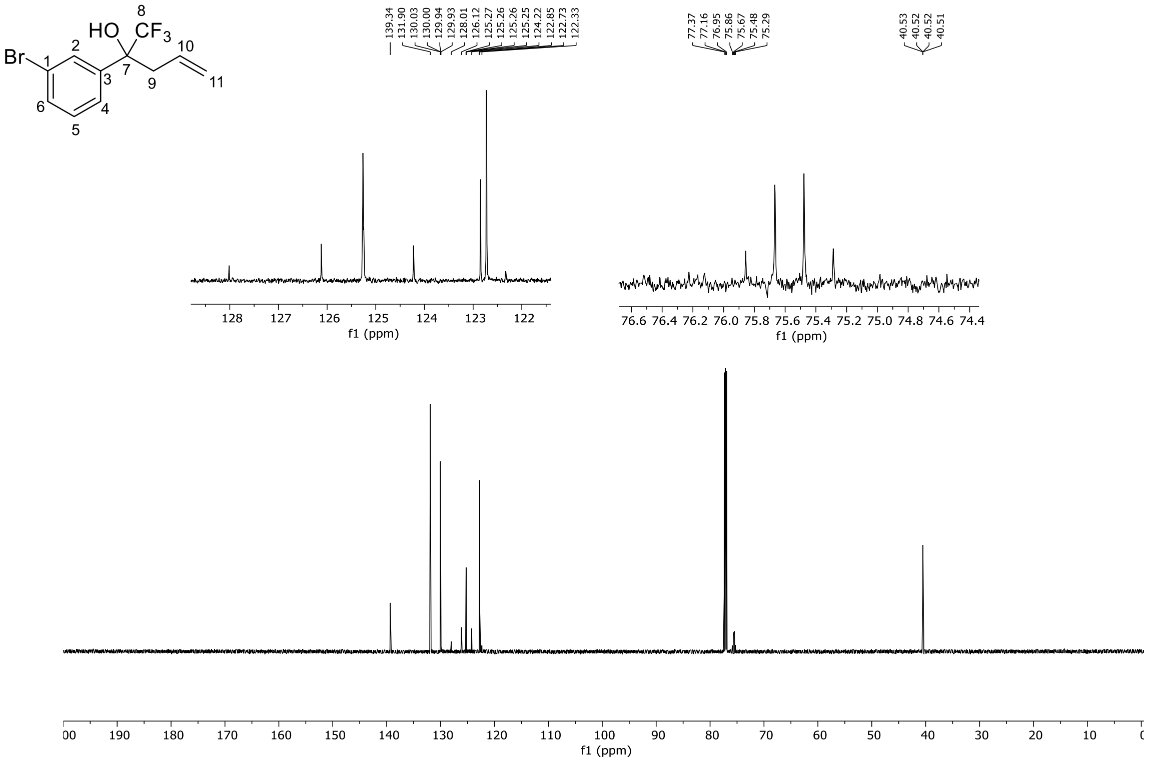

^{19}**F NMR** (564 MHz, $CDCl_3$)

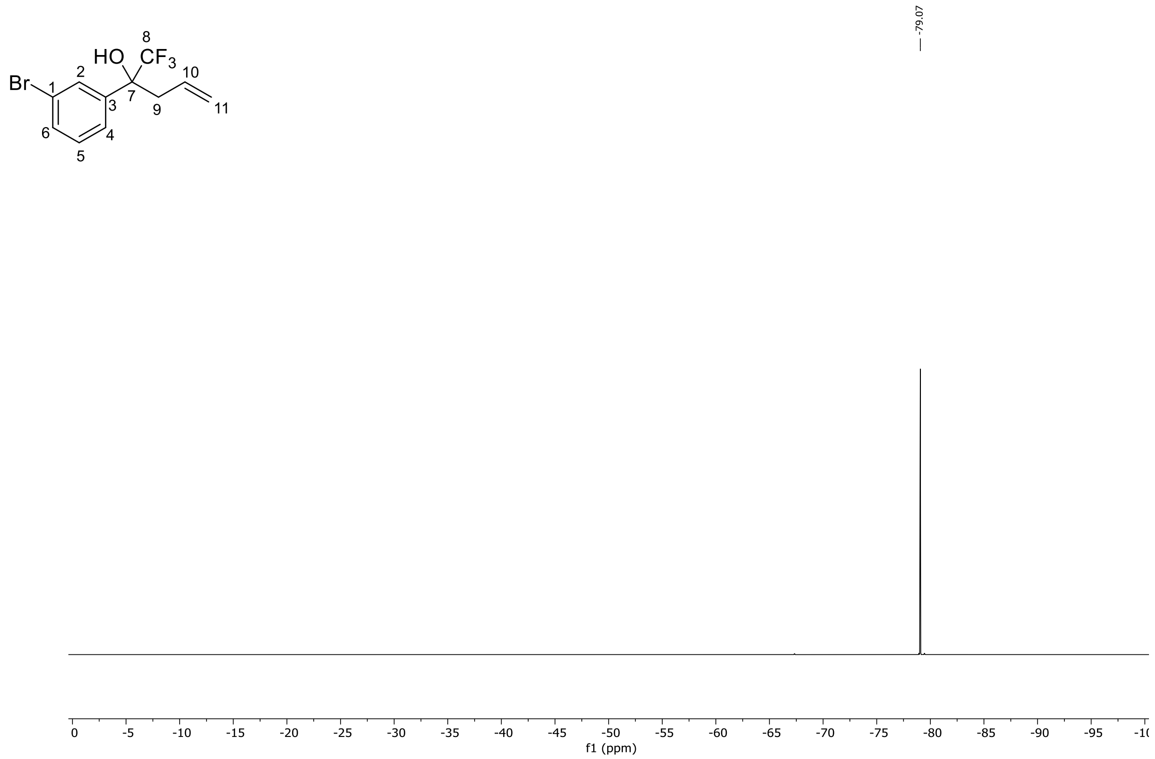

^{19}**F{^{1}H} NMR** (564 MHz, $CDCl_3$)

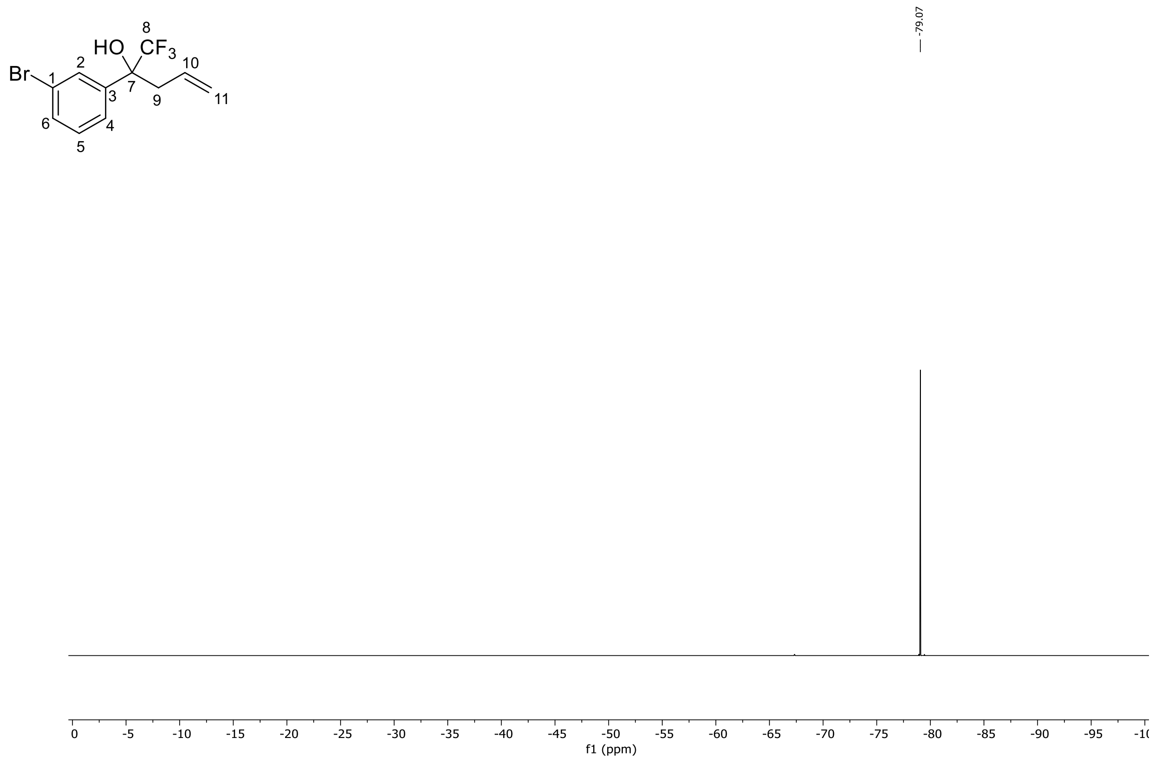

1-Bromo-3-(1,1,1-trifluoropenta-2,4-dien-2-yl)benzene (1b)

^{1}H NMR (599 MHz, $CDCl_3$)

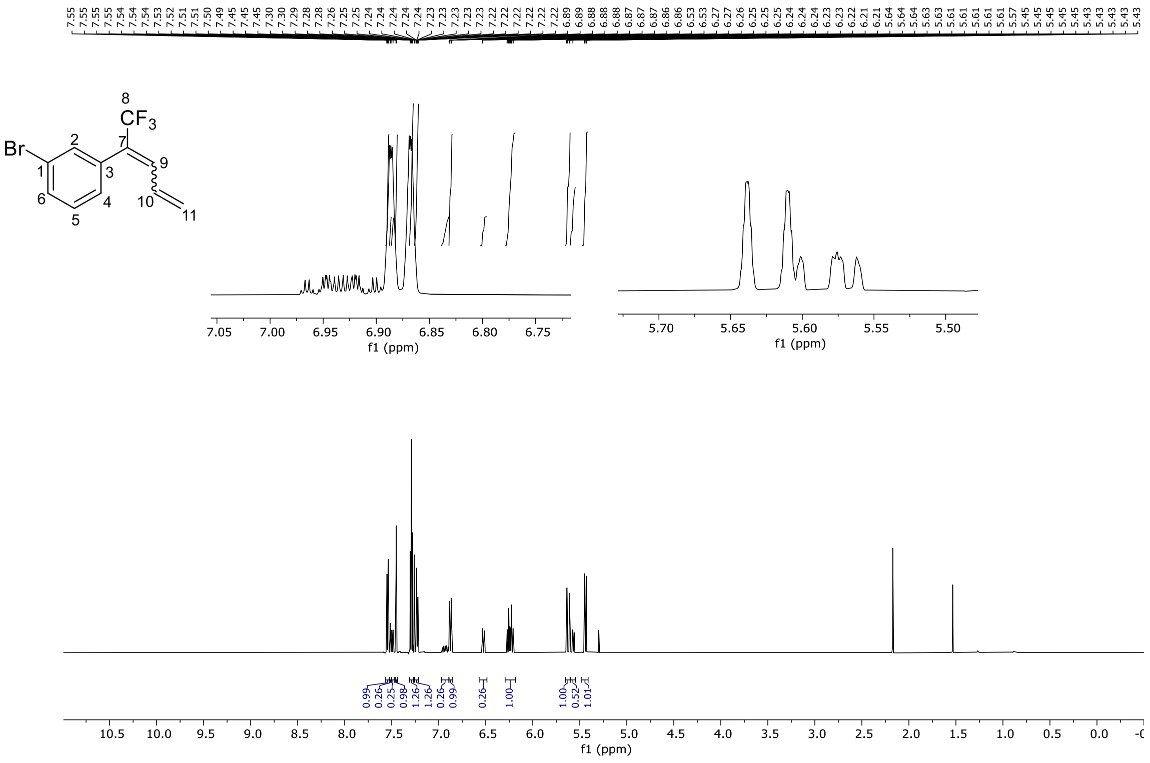

^{13}C NMR (151 MHz, $CDCl_3$)

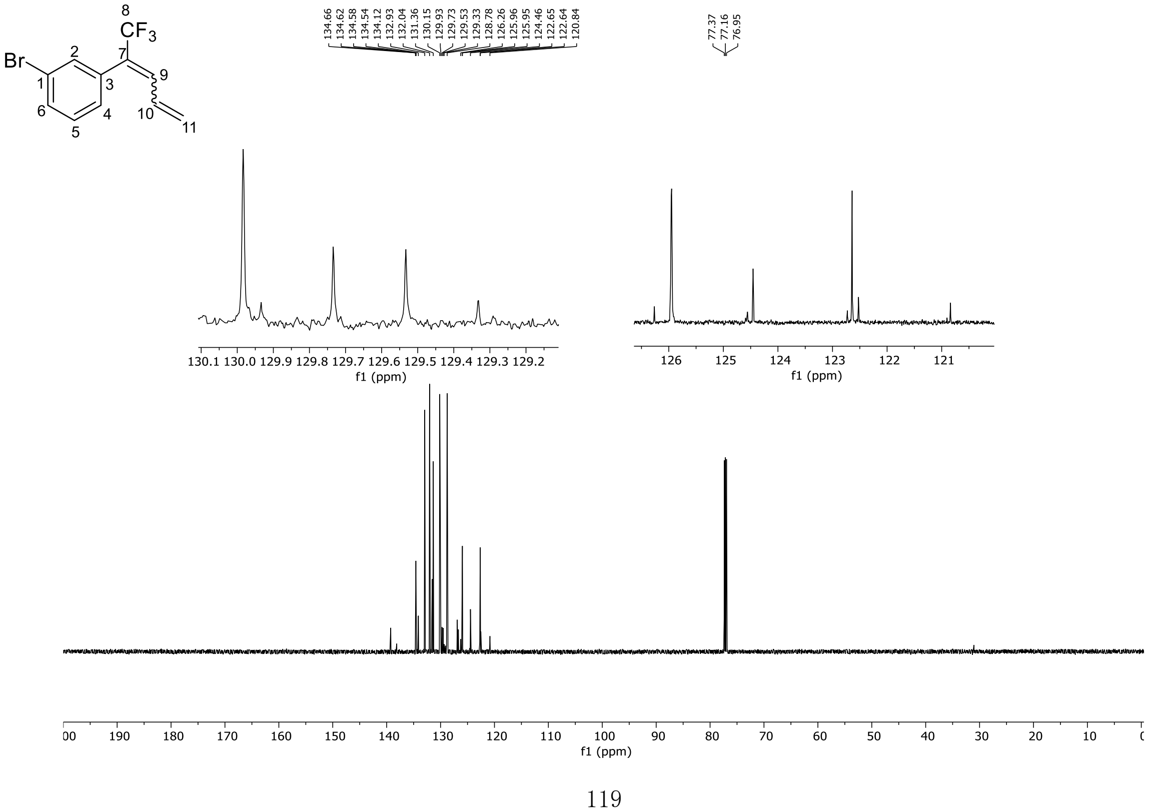

^{19}F NMR (564 MHz, $CDCl_3$)

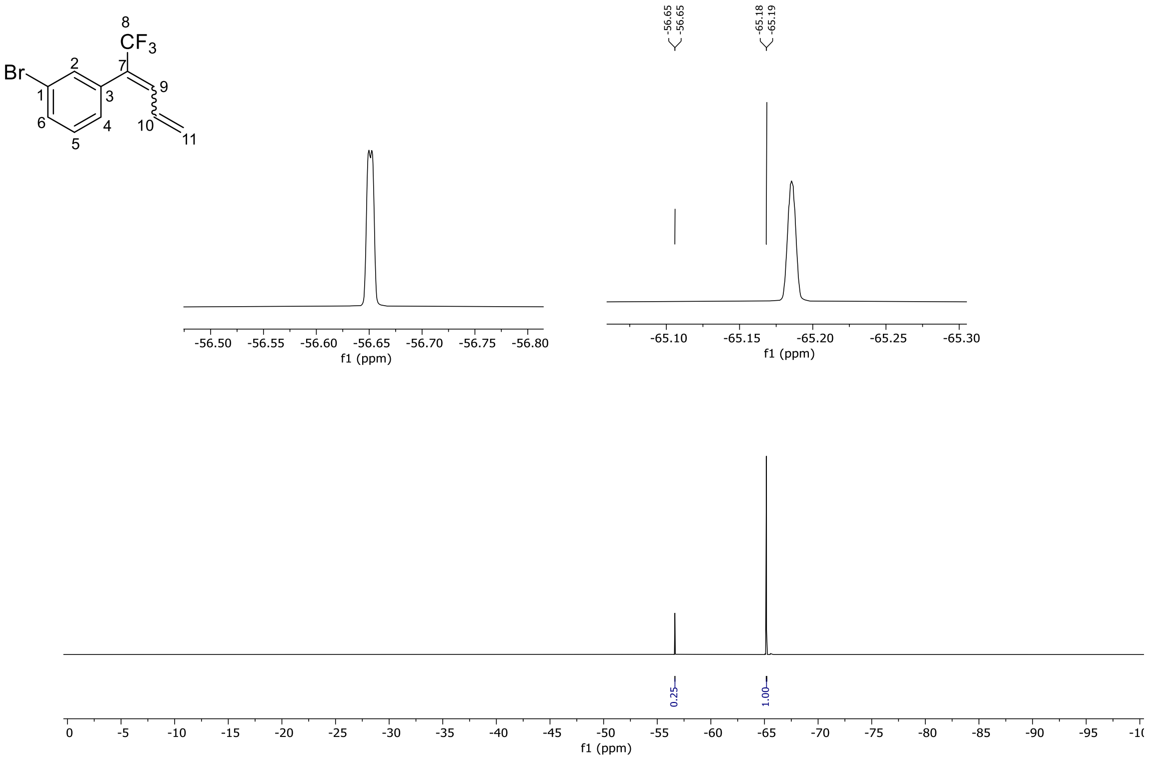

^{19}F{^{1}H} NMR (564 MHz, $CDCl_3$)

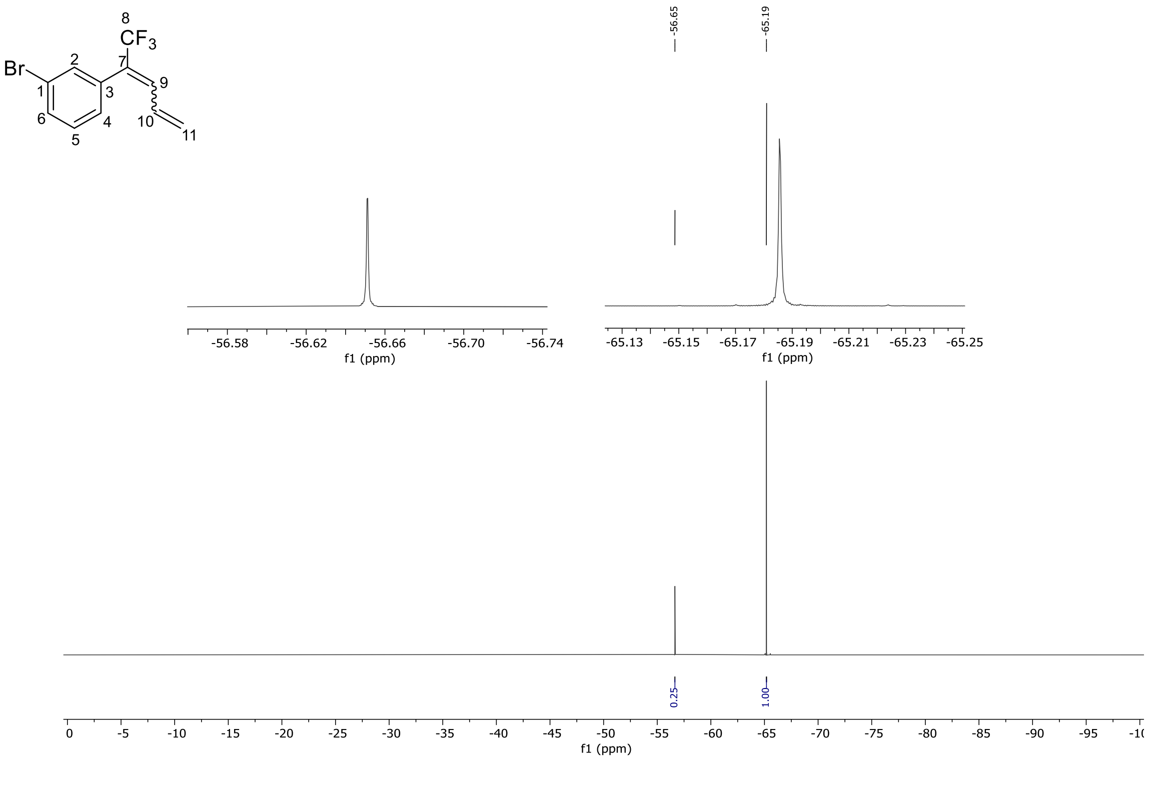

1-Chloro-4-(1,1,1-trifluoropenta-2,4-dien-2-yl)benzene (1c)

^{1}H NMR (599 MHz, $CDCl_3$)

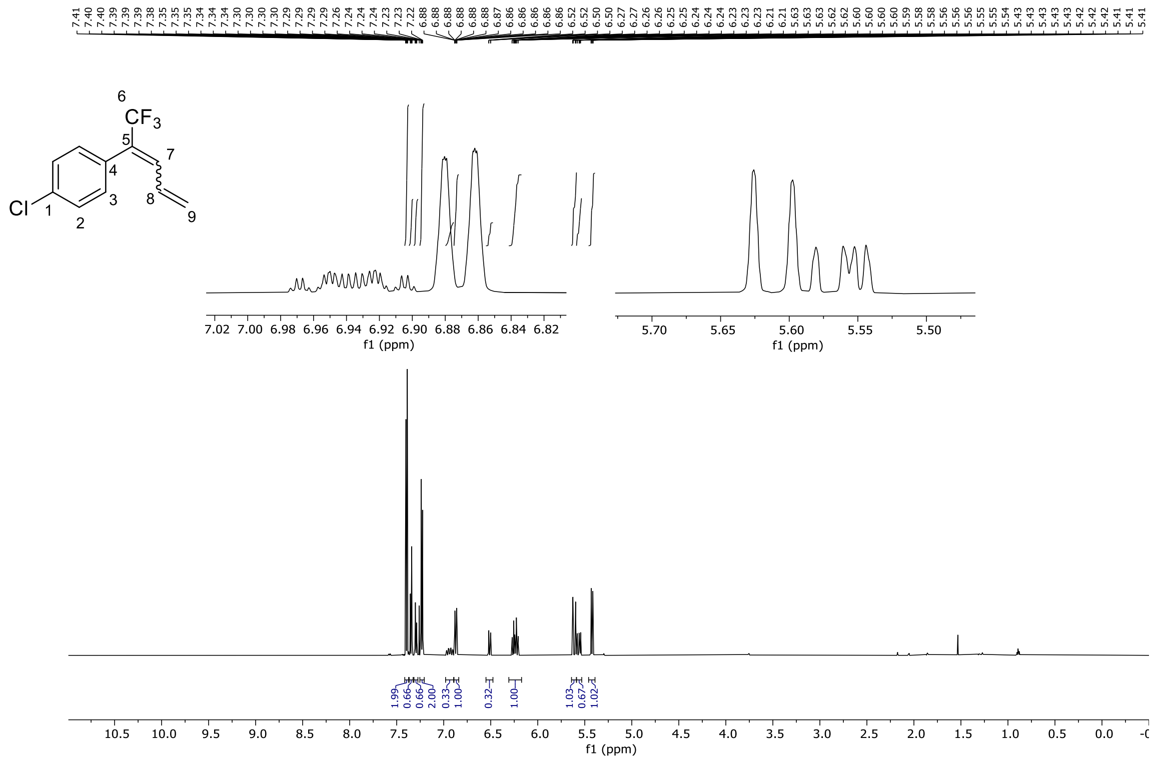

^{13}C NMR (151 MHz, $CDCl_3$)

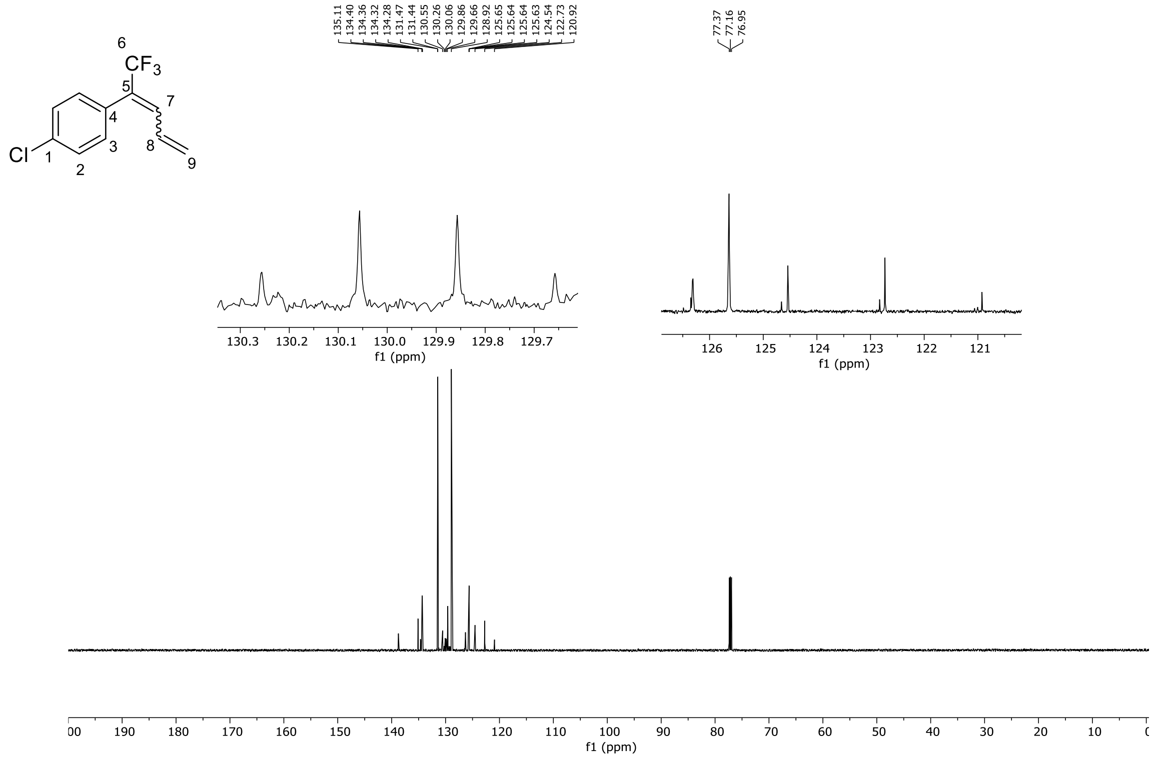

^{19}F NMR (564 MHz, $CDCl_3$)

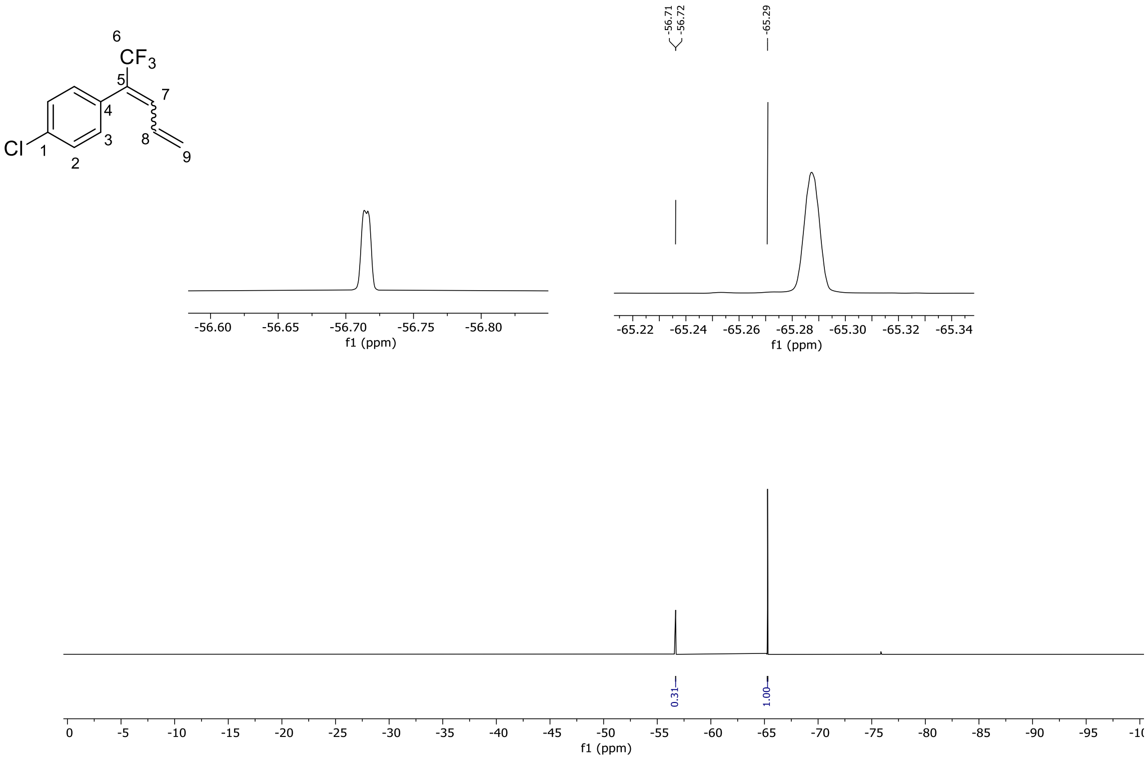

^{19}F{^{1}H} NMR (564 MHz, $CDCl_3$)

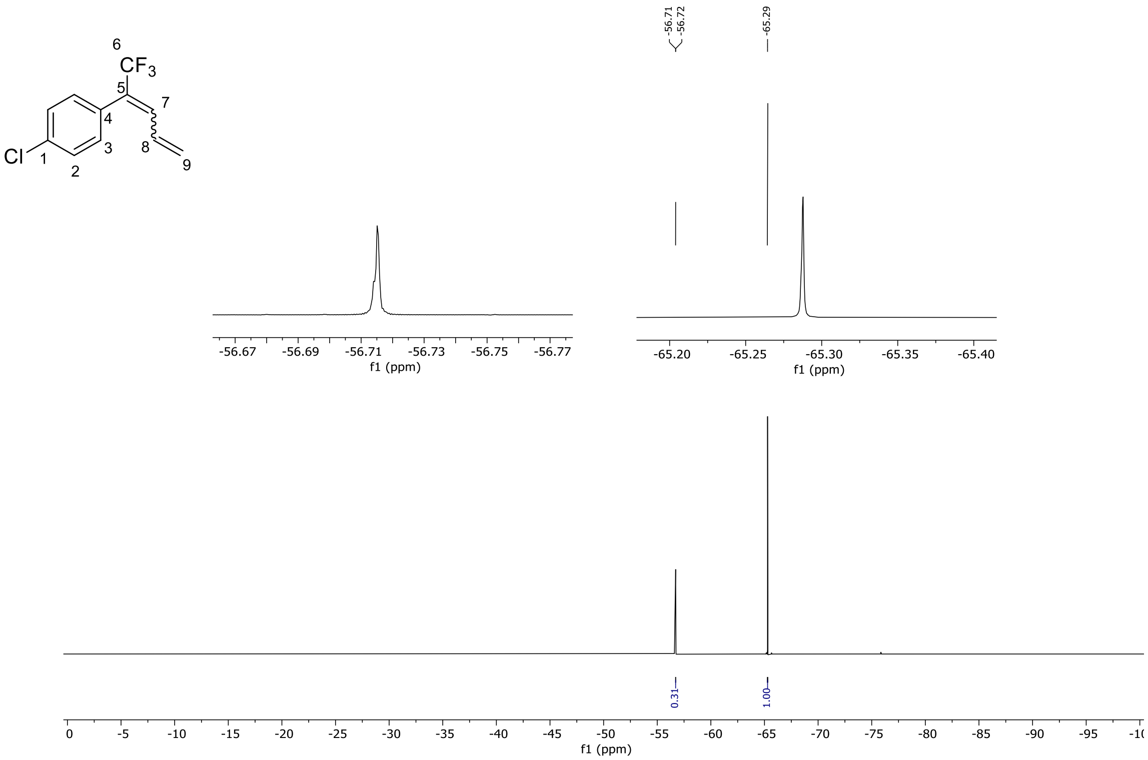

2-(3-Chlorophenyl)-1,1,1-trifluoropent-4-en-2-ol (1d-1)

^{1}H NMR (599 MHz, $CDCl_3$)

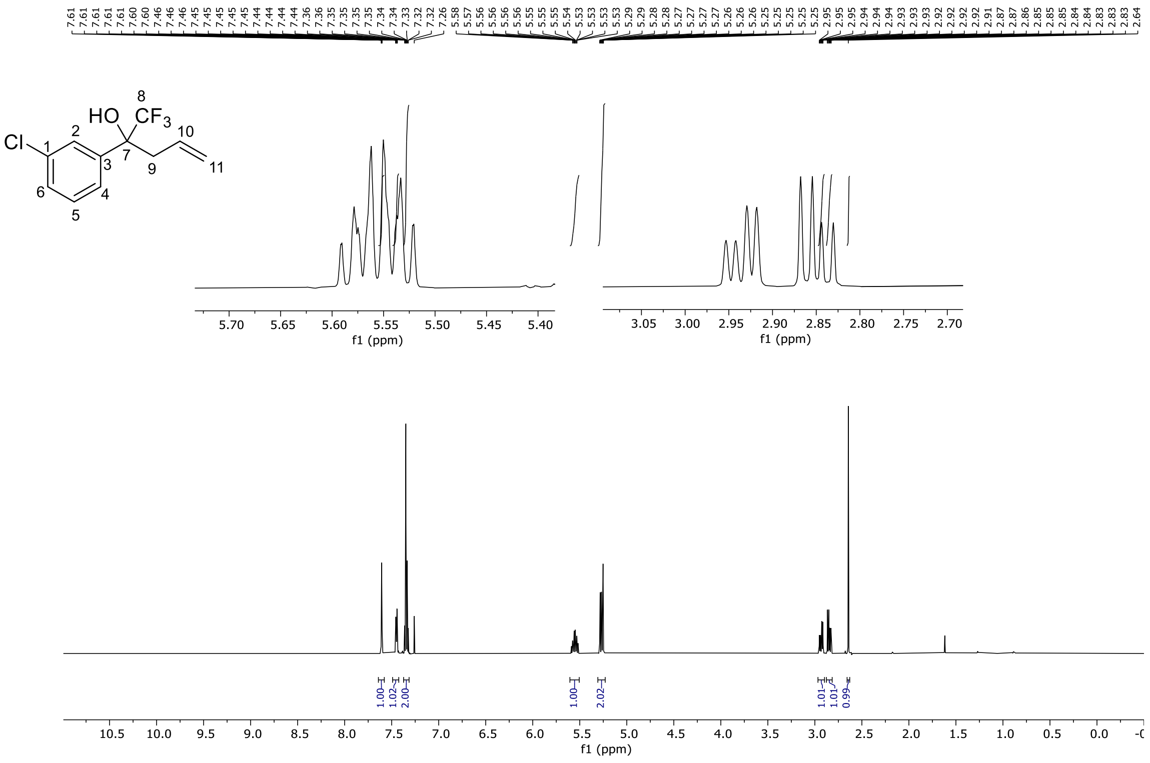

^{13}C NMR (151 MHz, $CDCl_3$)

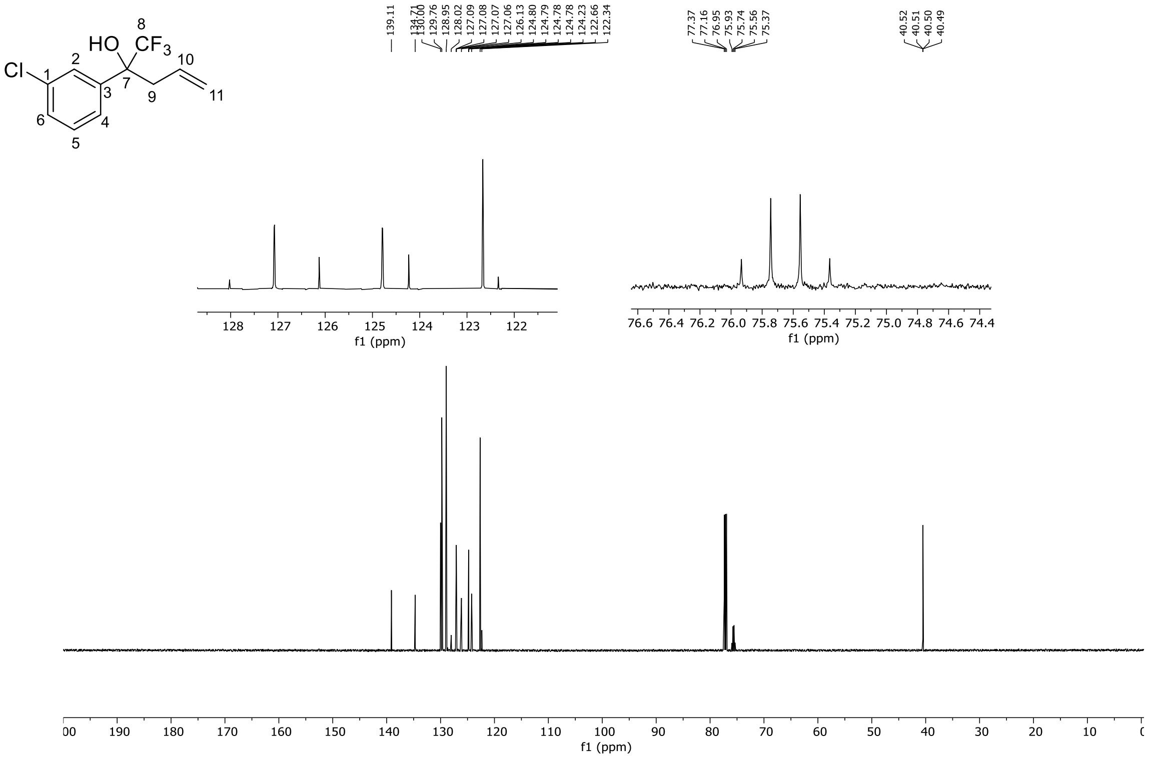

^{19}**F NMR** (564 MHz, $CDCl_3$)

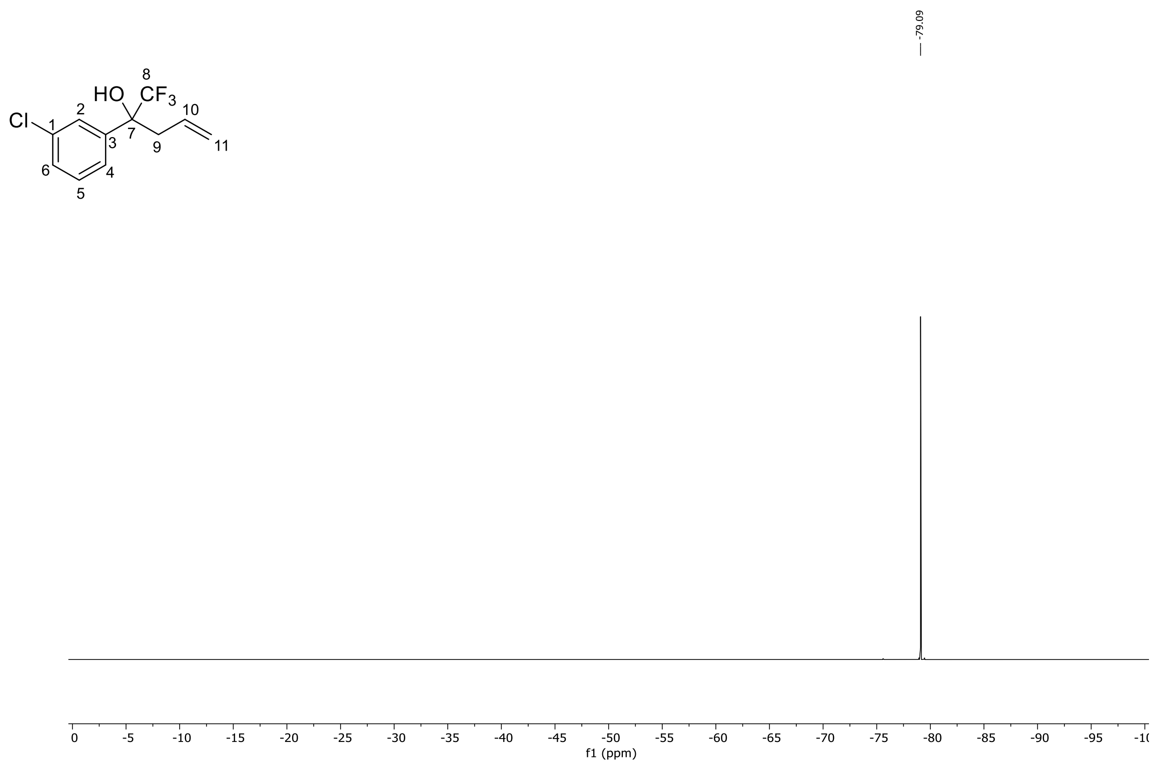

^{19}**F{^{1}H} NMR** (564 MHz, $CDCl_3$)

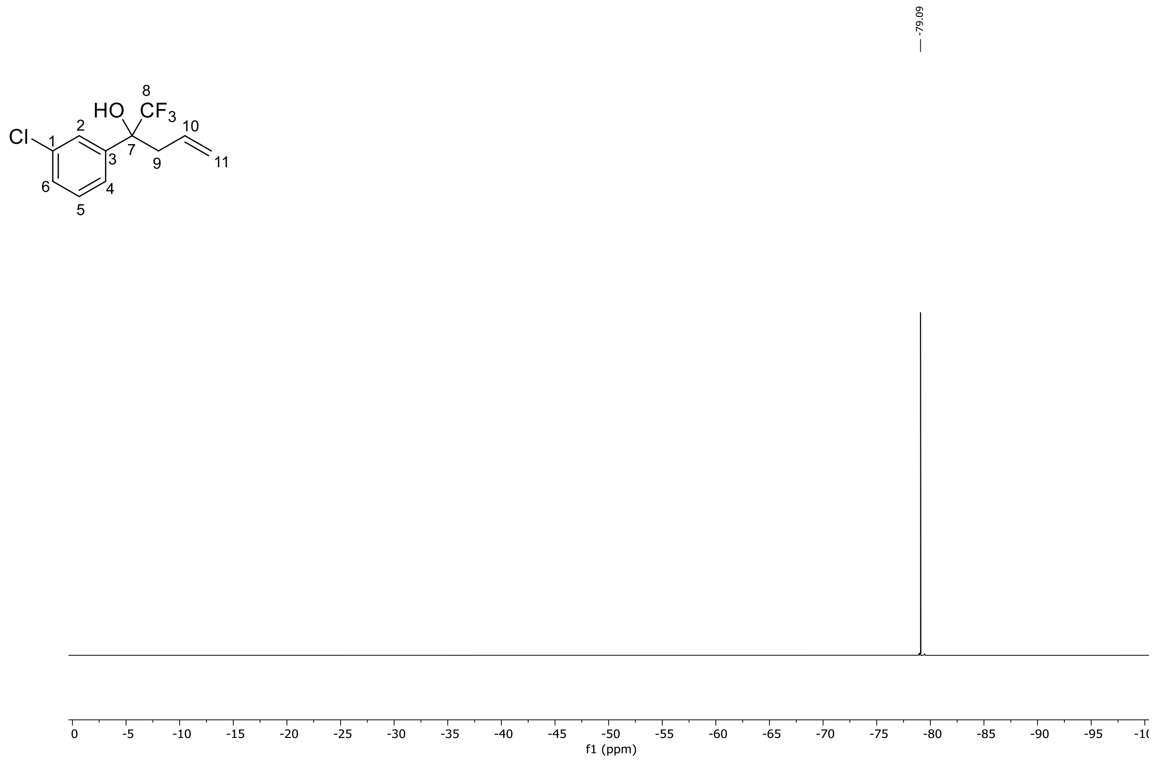

1-Chloro-3-(1,1,1-trifluoropenta-2,4-dien-2-yl)benzene (1d)

^{1}H NMR (500 MHz, $CDCl_3$)

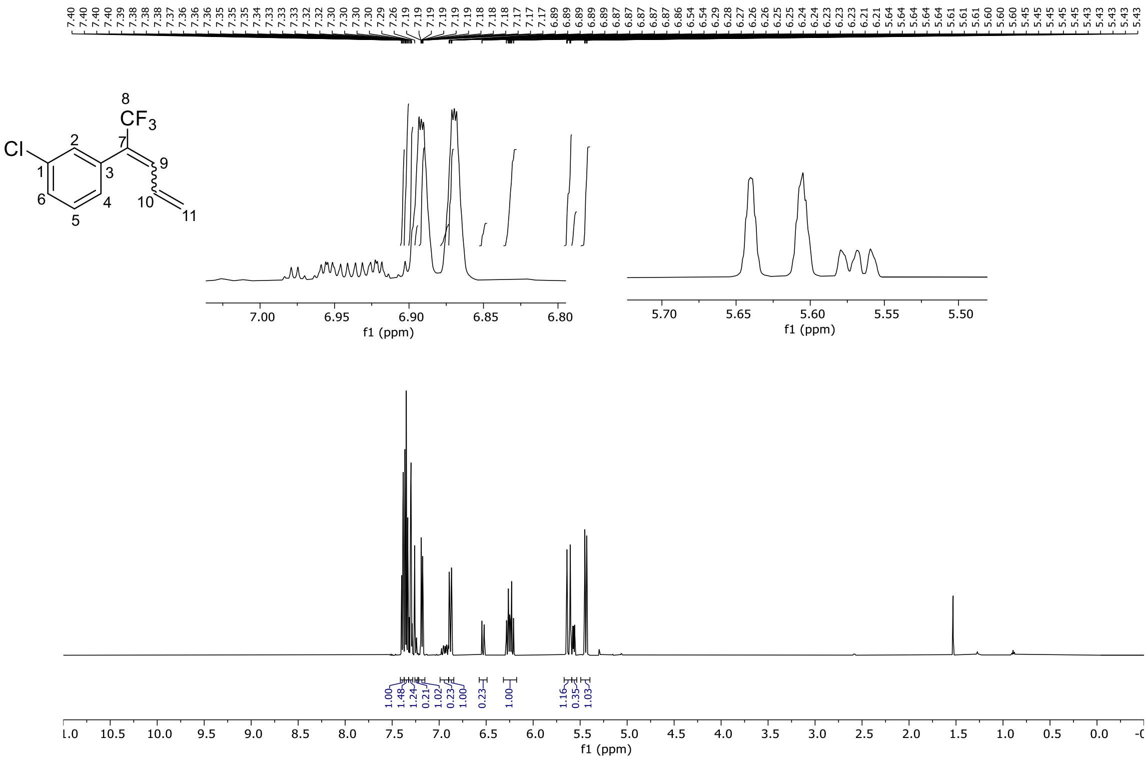

^{13}C NMR (126 MHz, $CDCl_3$)

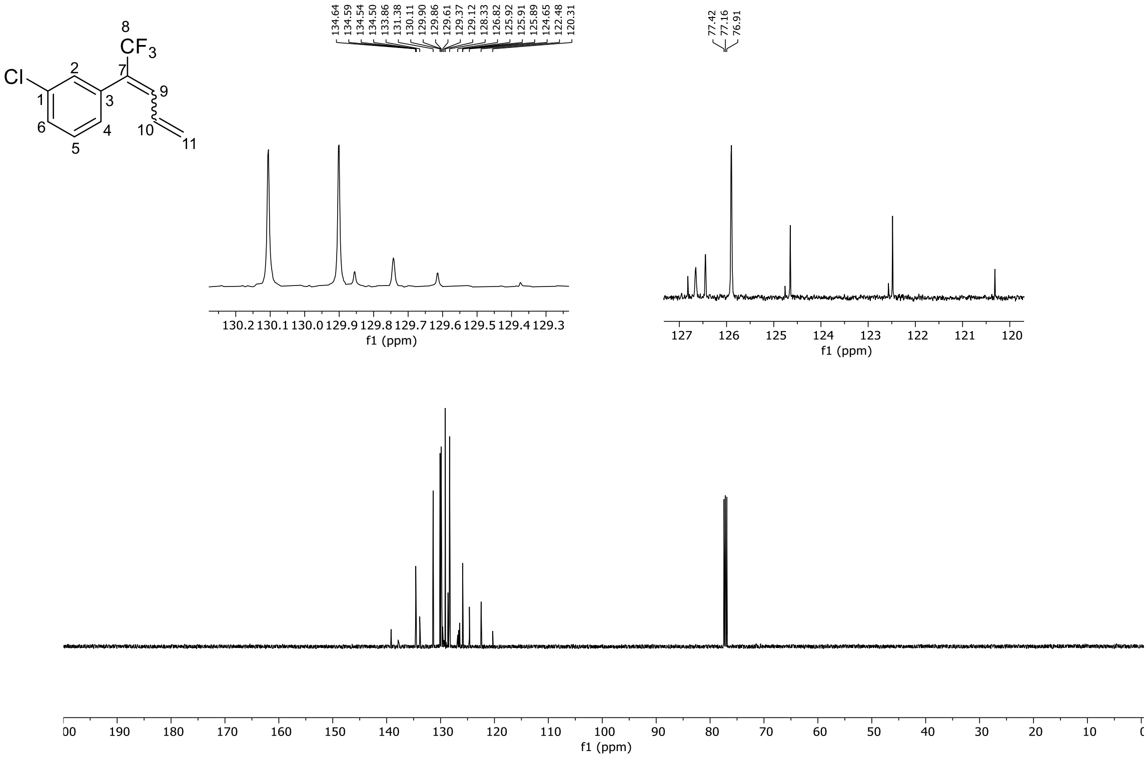

^{19}F NMR (470 MHz, $CDCl_3$)

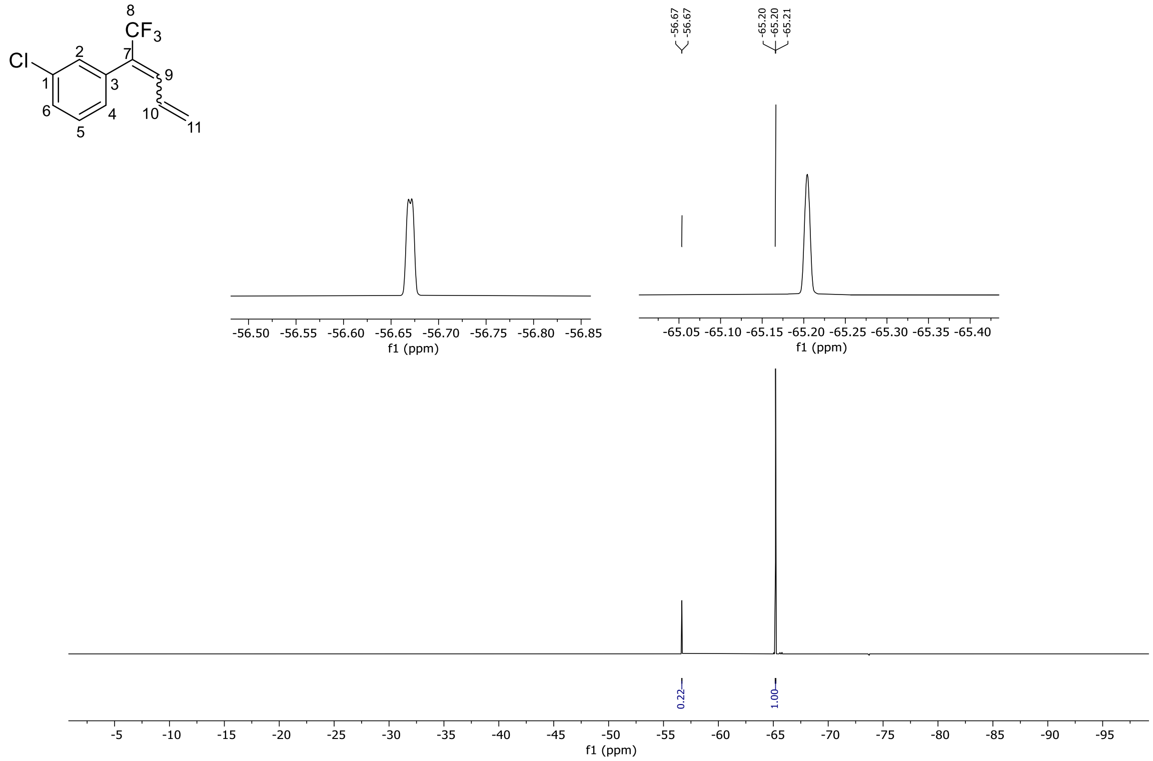

^{19}F{^{1}H} NMR (470 MHz, $CDCl_3$)

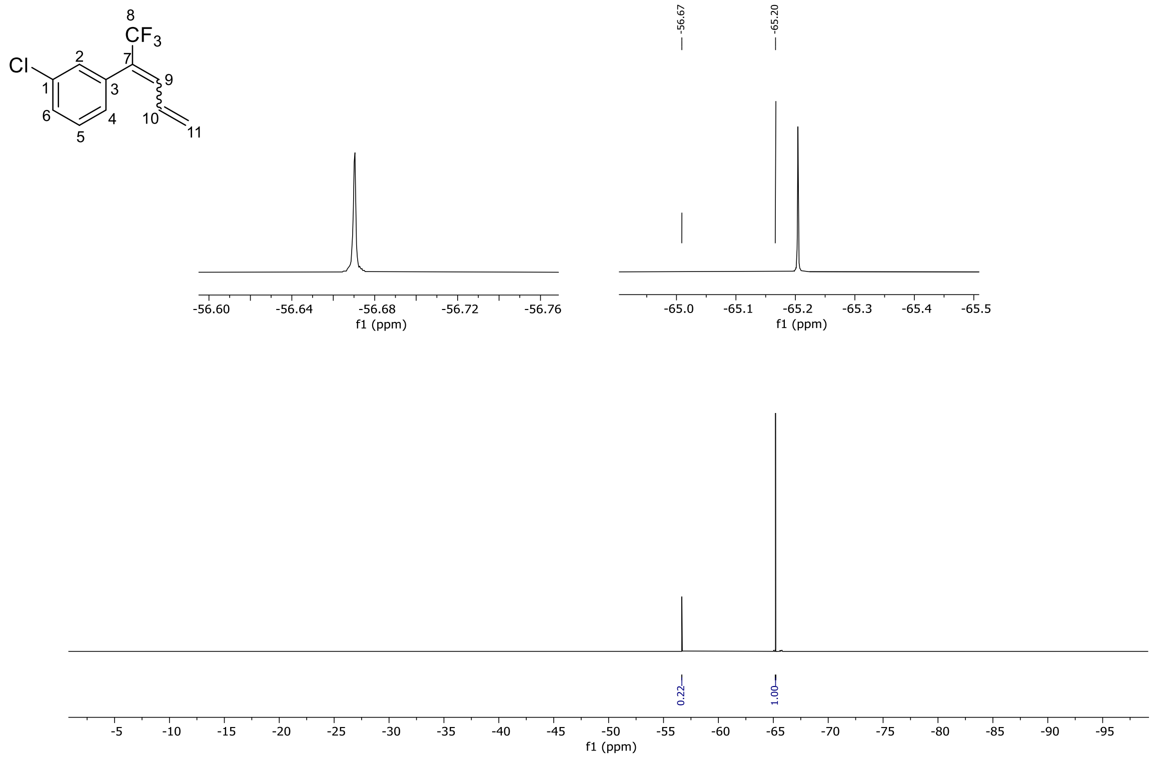

2-(3,5-Dichlorophenyl)-1,1,1-trifluoropent-4-en-2-ol (1e-1)

^{1}H NMR (500 MHz, $CDCl_3$)

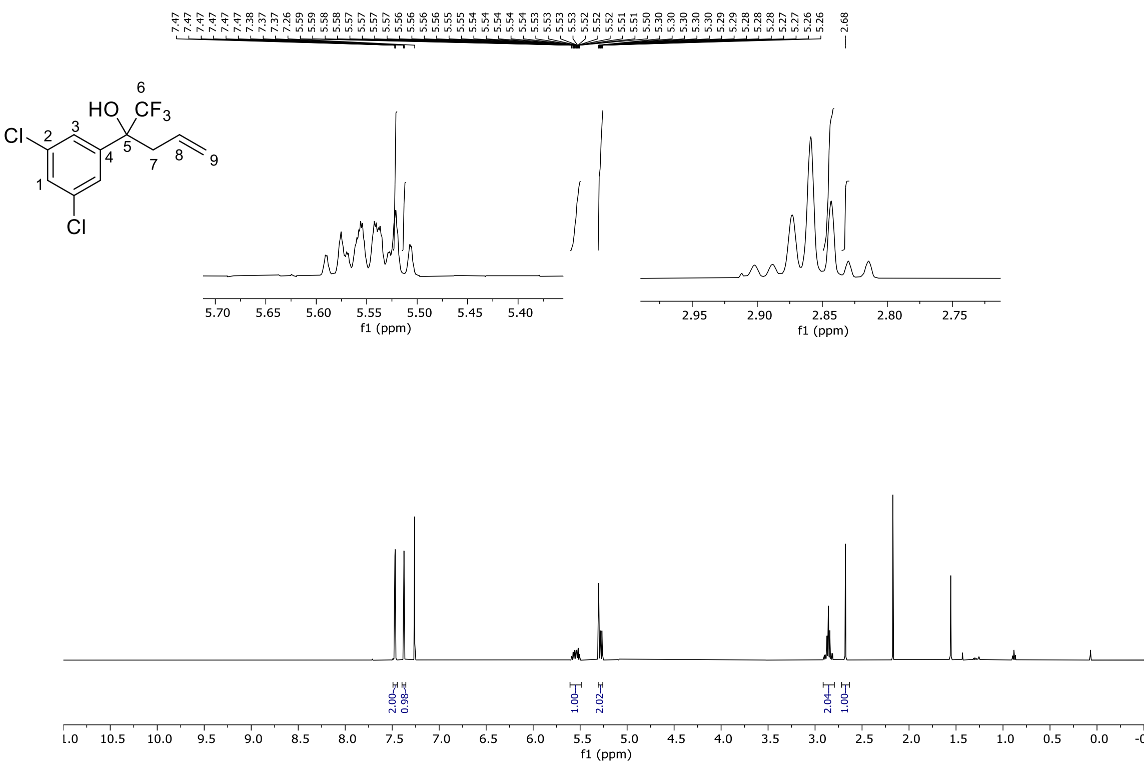

^{13}C NMR (126 MHz, $CDCl_3$)

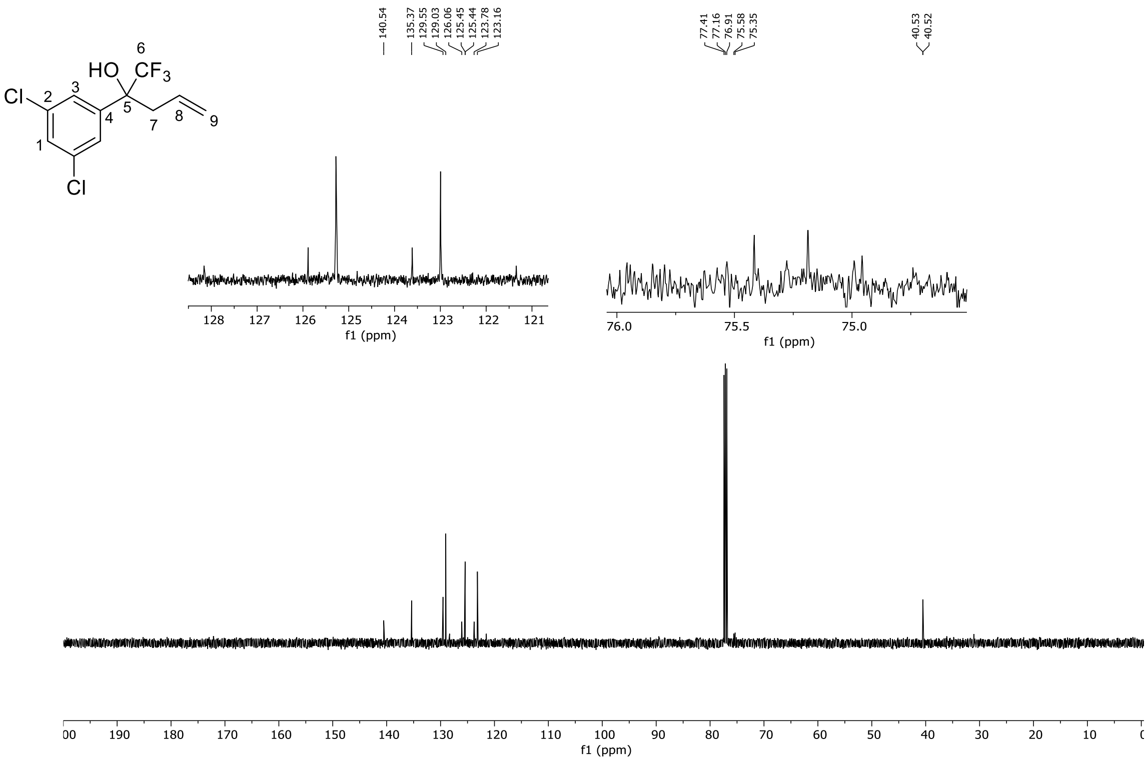

^{19}F NMR (470 MHz, $CDCl_3$)

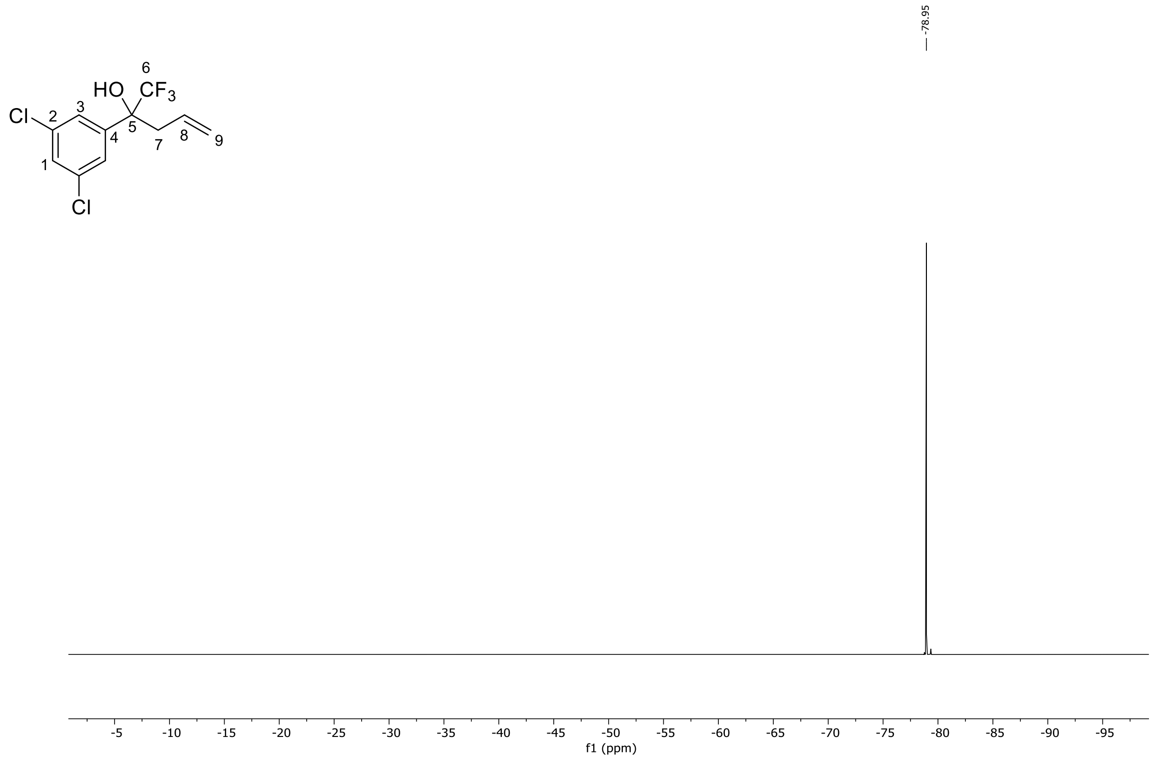

^{19}F{^{1}H} NMR (470 MHz, $CDCl_3$)

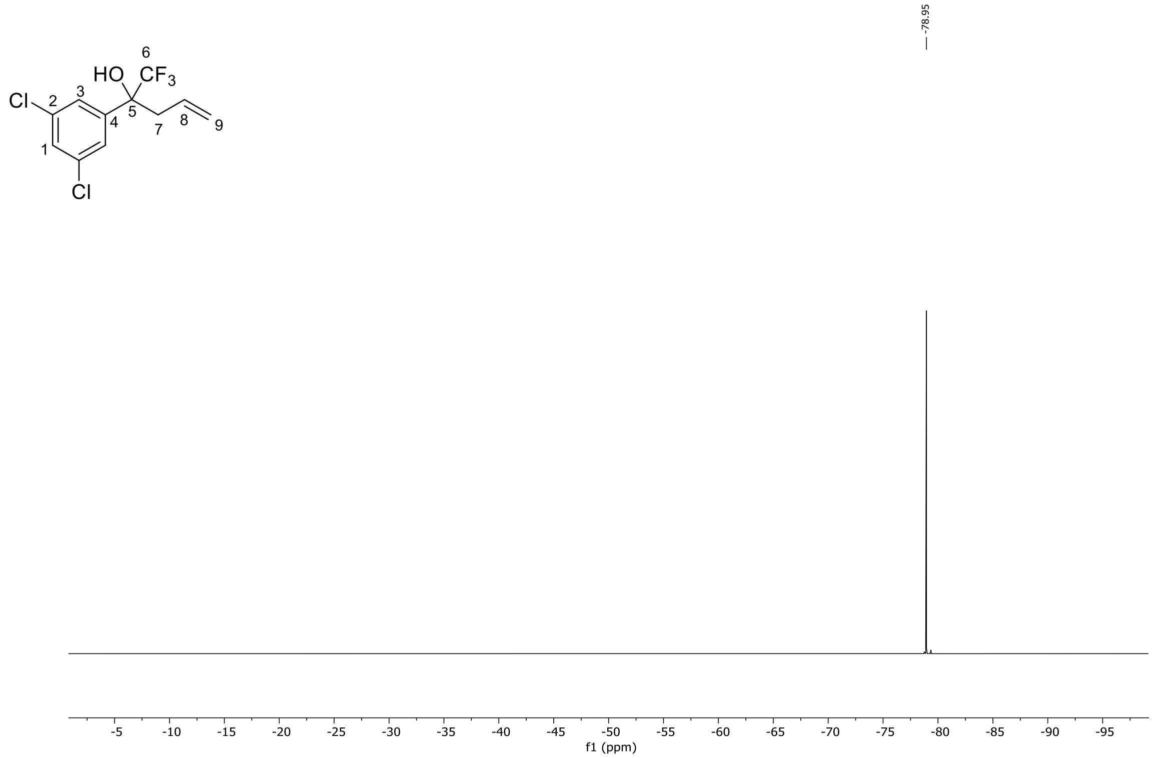

1-Chloro-3-(1,1,1-trifluoropenta-2,4-dien-2-yl)benzene (1e)

^{1}H NMR (599 MHz, $CDCl_3$)

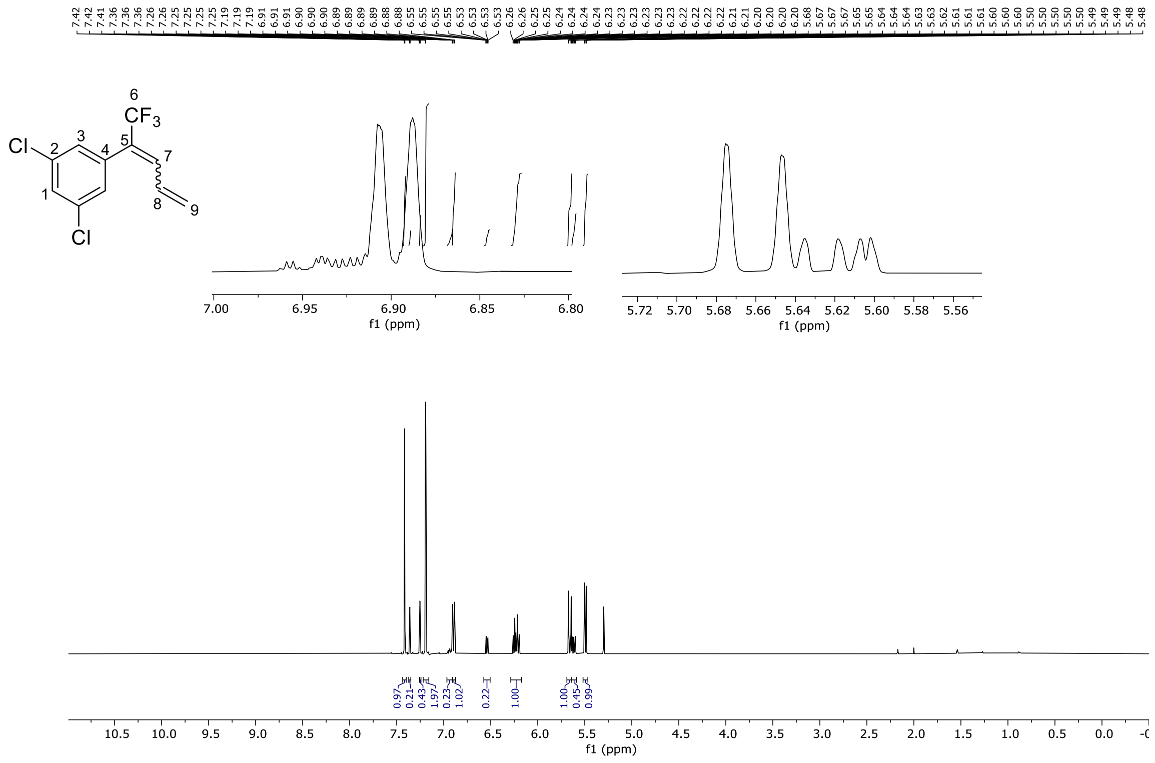

^{13}C NMR (151 MHz, $CDCl_3$)

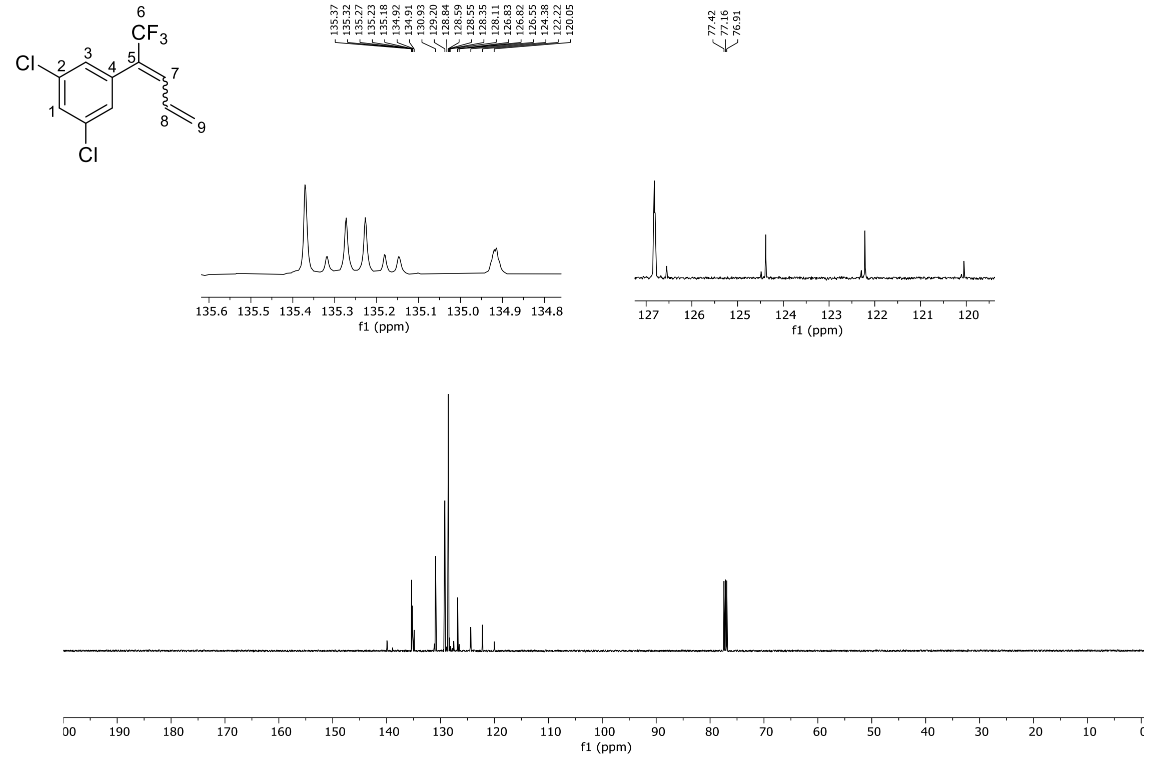

^{19}F NMR (564 MHz, $CDCl_3$)

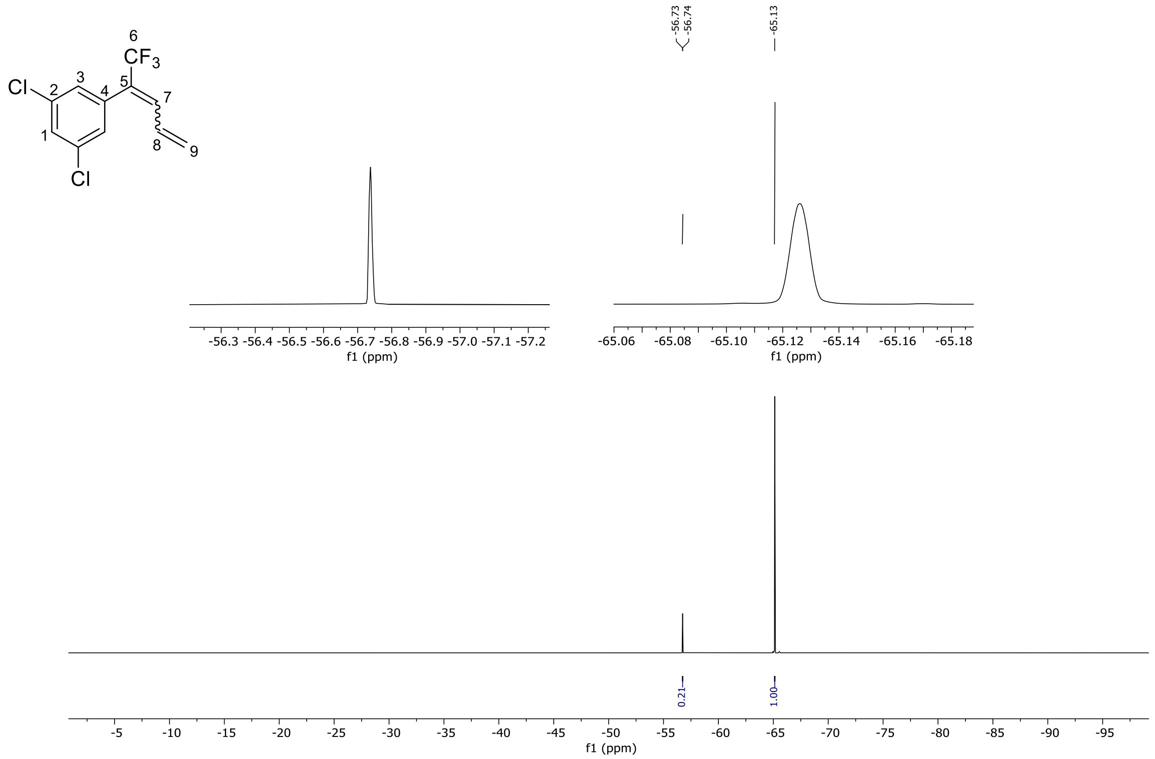

^{19}F{^{1}H} NMR (564 MHz, $CDCl_3$)

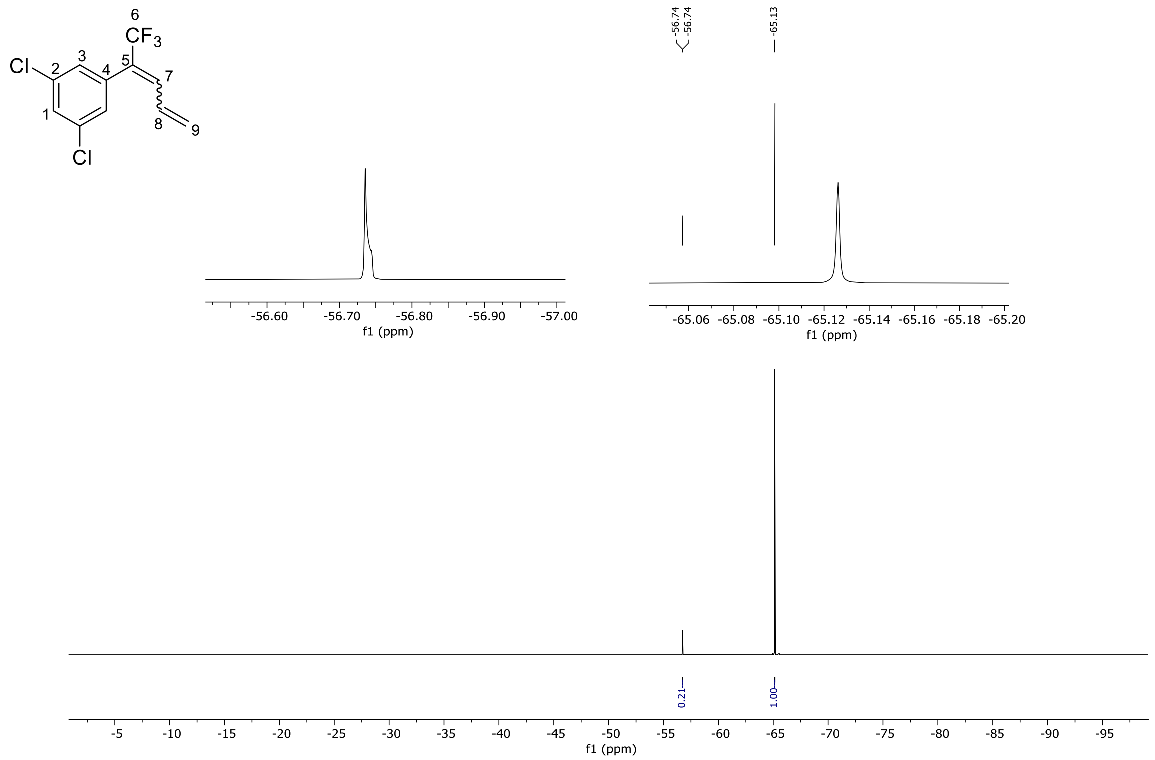

1-Fluoro-4-(1,1,1-trifluoropenta-2,4-dien-2-yl)benzene (1f)

^{1}H NMR (599 MHz, $CDCl_3$)

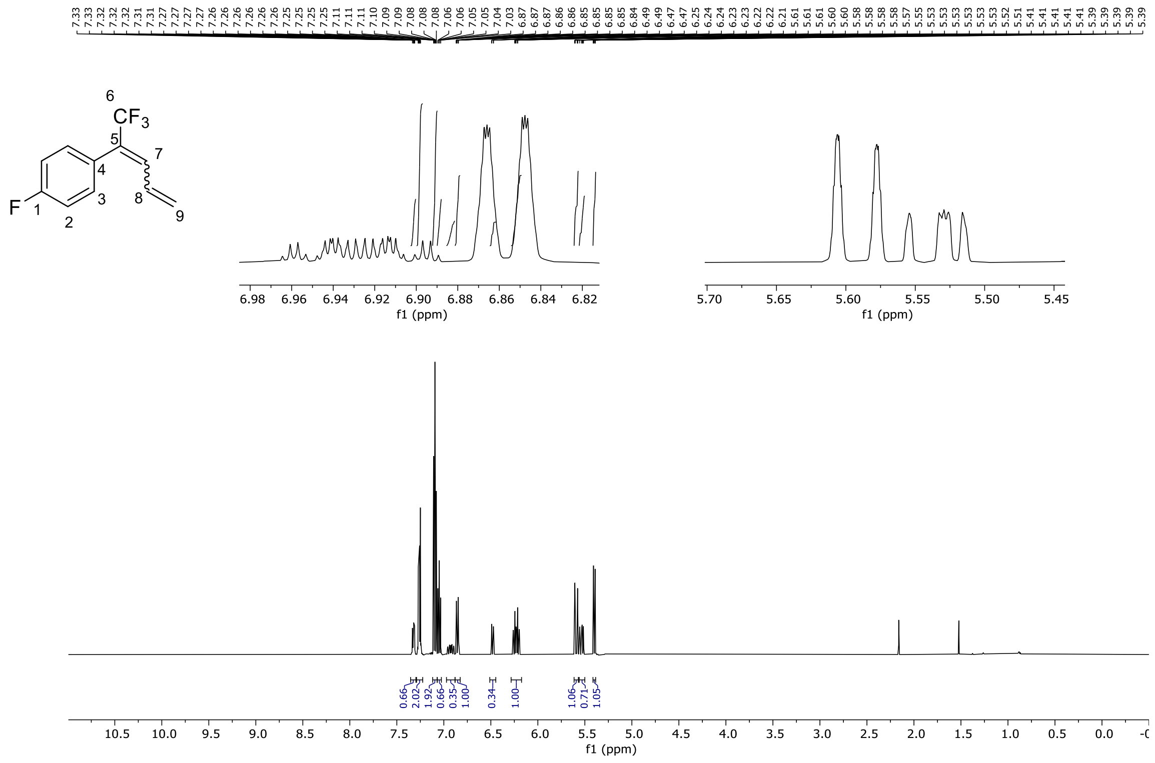

^{13}C NMR (151 MHz, $CDCl_3$)

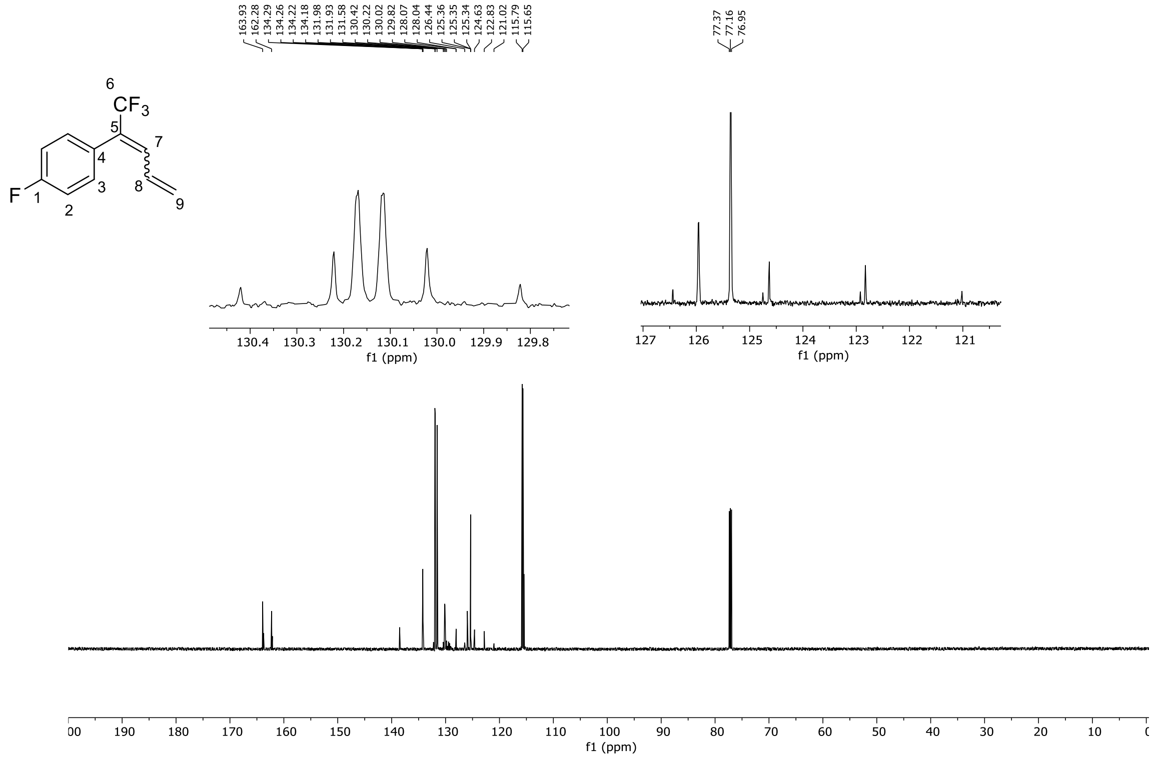

^{19}F NMR (564 MHz, $CDCl_3$)

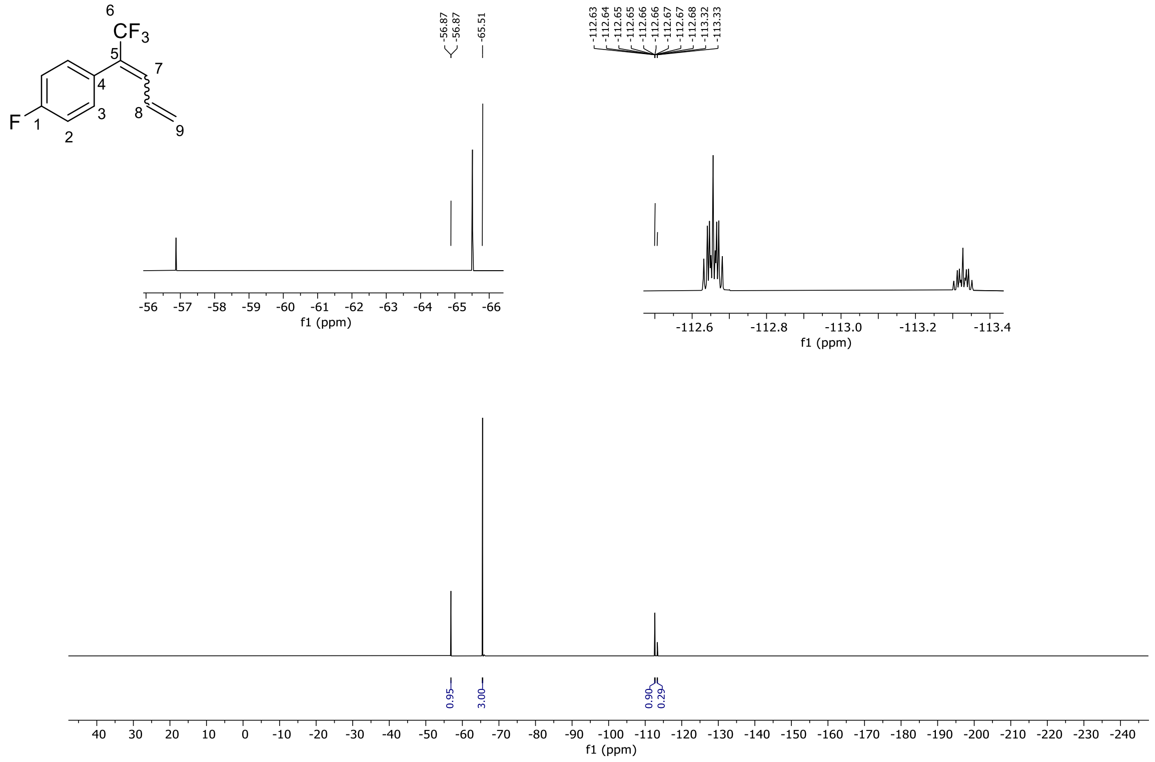

^{19}F{^{1}H} NMR (376 MHz, $CDCl_3$)

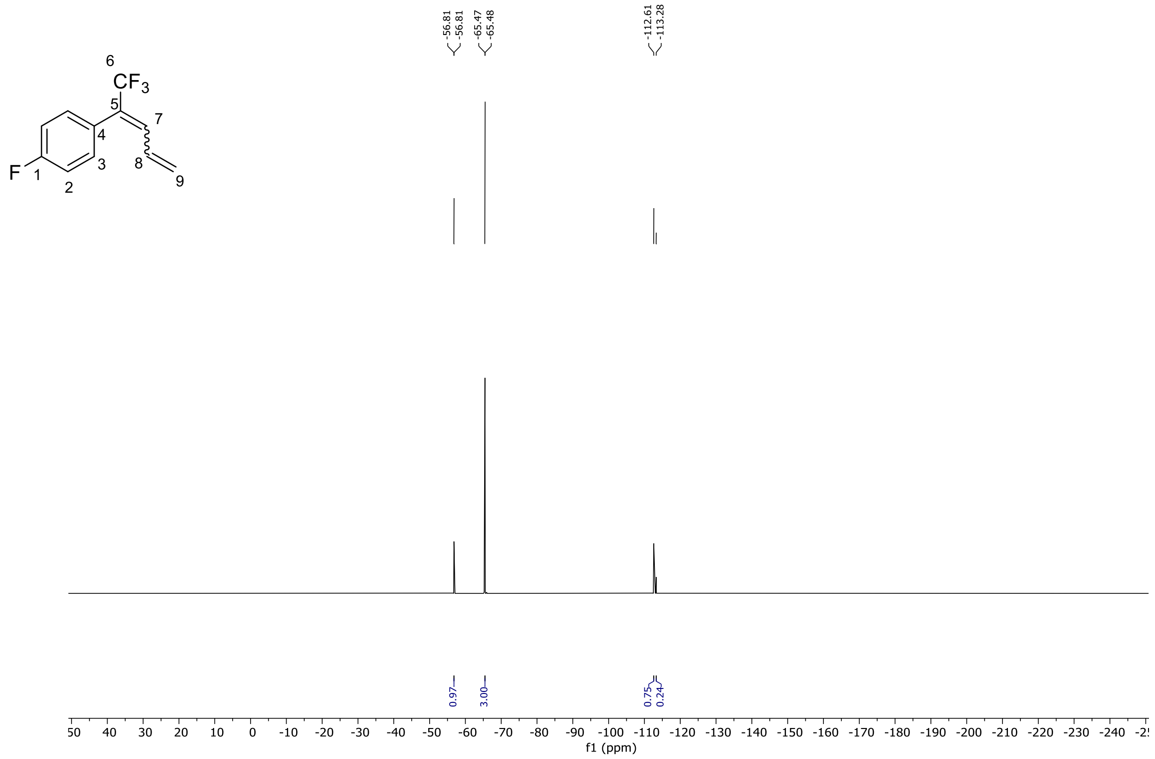

1,1,1-Trifluoro-2-(4-fluoro-2-methylphenyl)pent-4-en-2-ol (1g-1)

¹H NMR (500 MHz, $CDCl_3$)

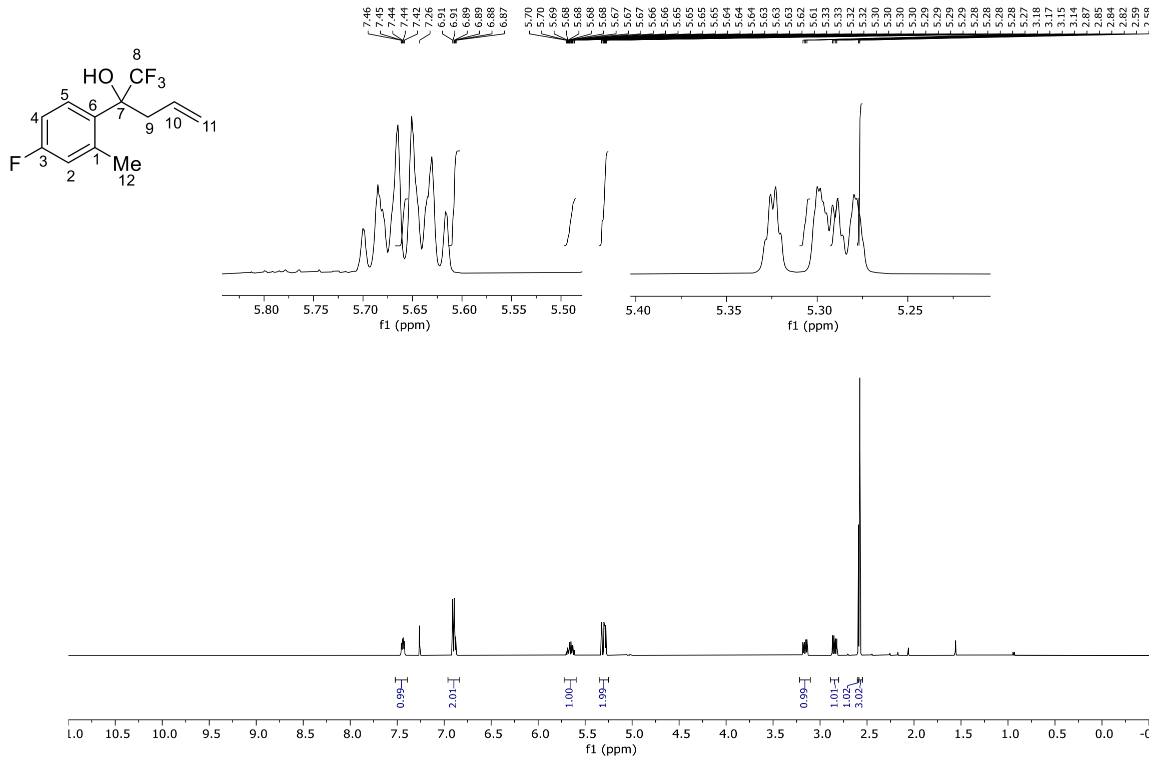

¹³C NMR (126 MHz, $CDCl_3$)

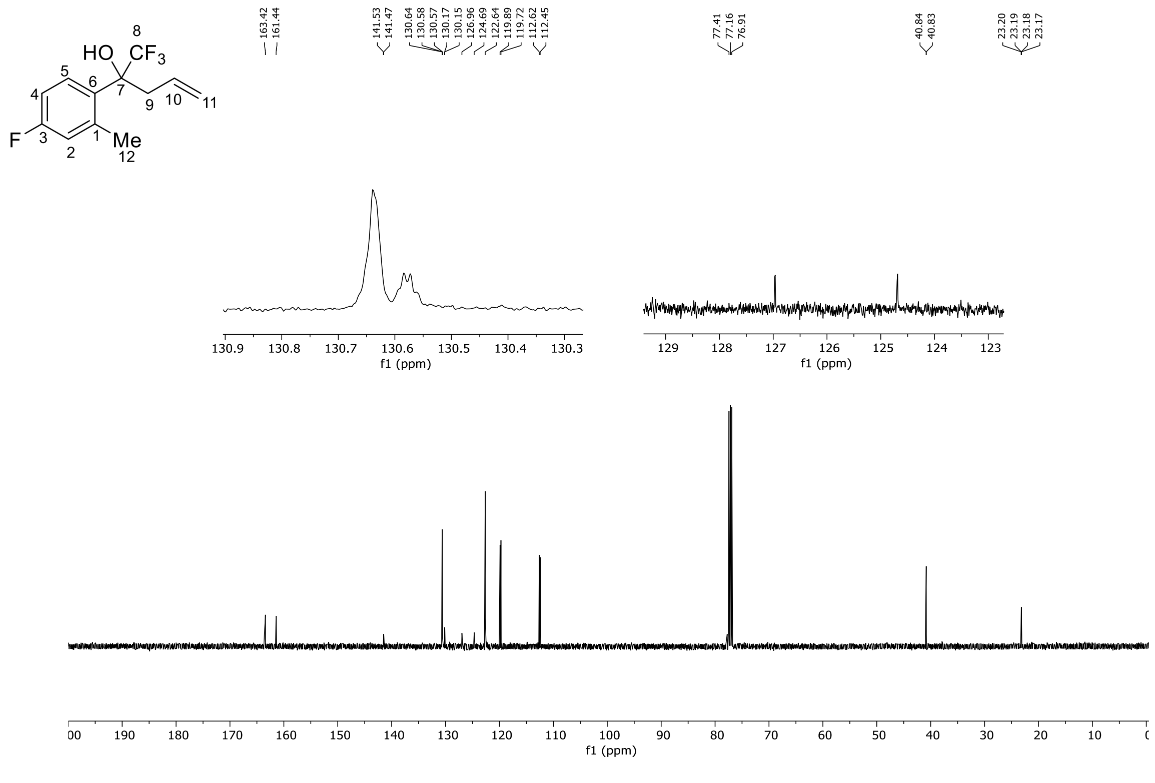

^{19}F NMR (470 MHz, $CDCl_3$)

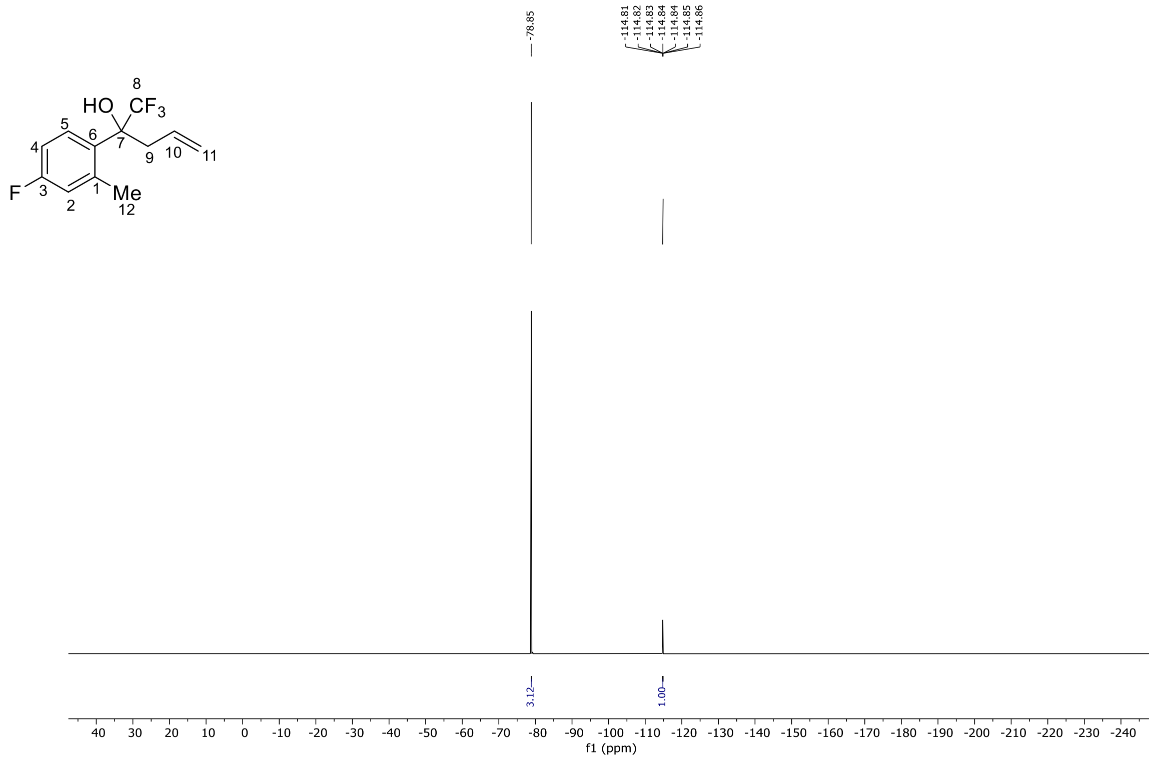

^{19}F{^{1}H} NMR (470 MHz, $CDCl_3$)

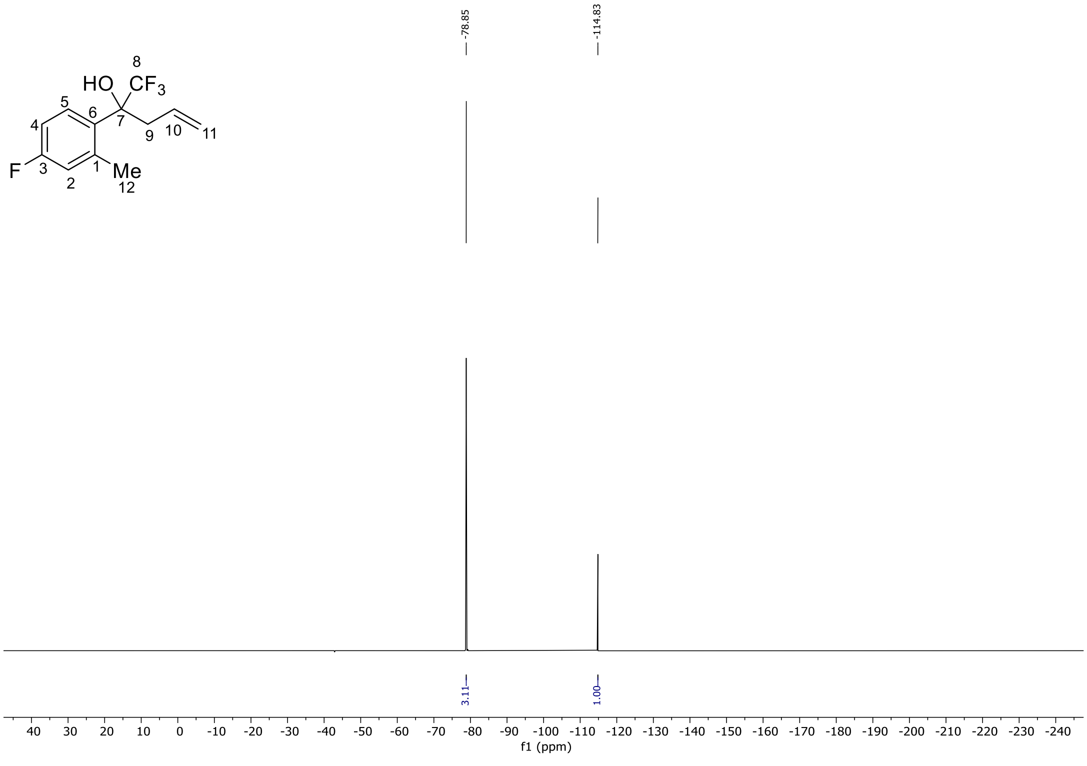

4-Fluoro-2-methyl-1-(1,1,1-trifluoropenta-2,4-dien-2-yl)benzene (1g)

^{1}H NMR (500 MHz, $CDCl_3$)

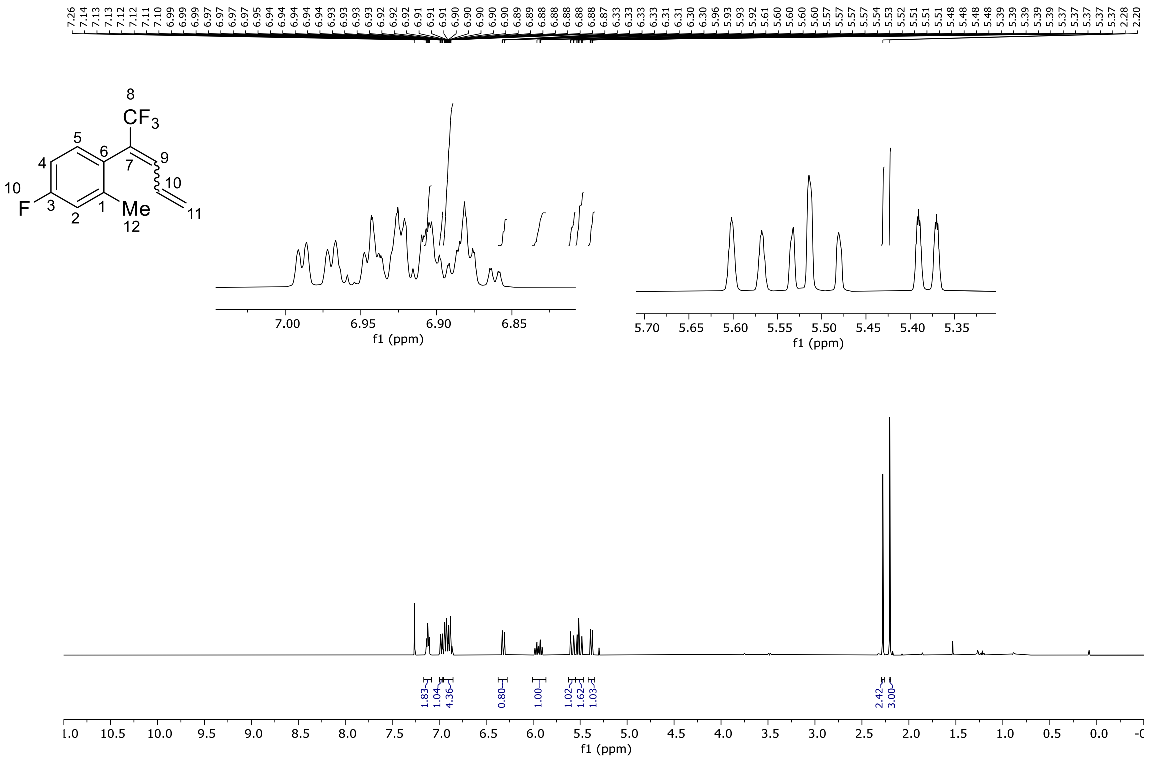

^{13}C NMR (126 MHz, $CDCl_3$)

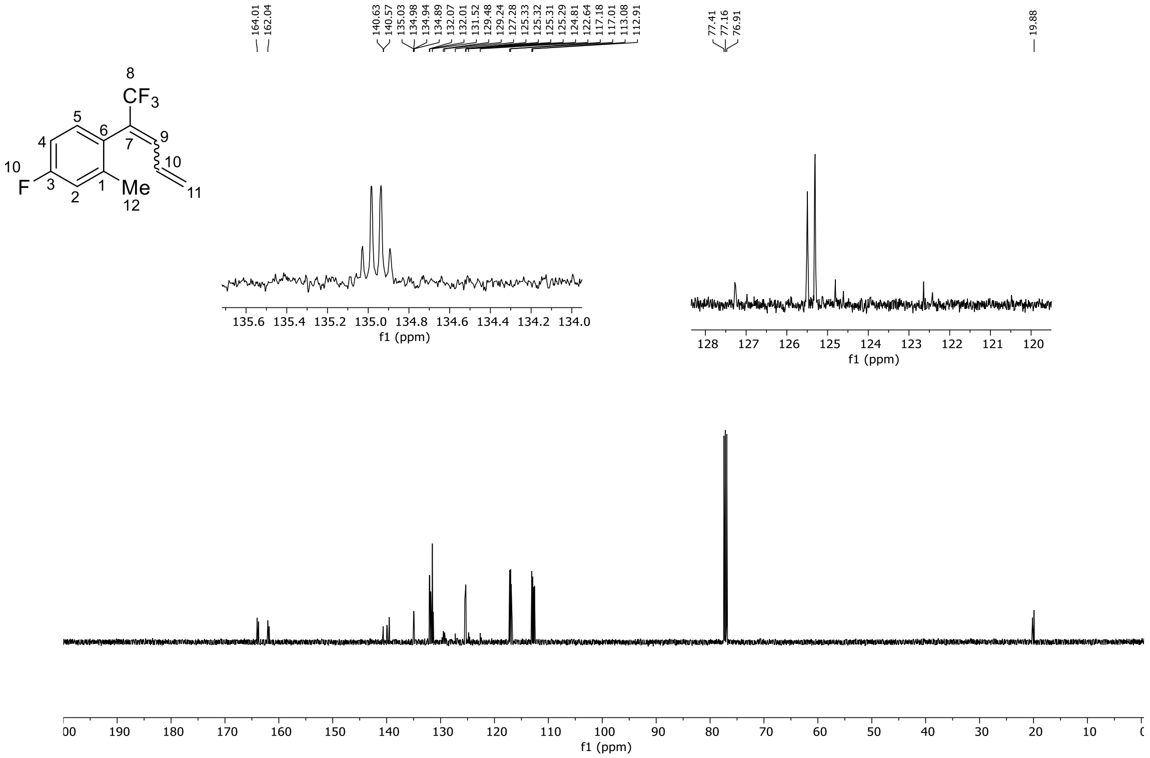

^{19}F NMR (470 MHz, CDCl$_3$)

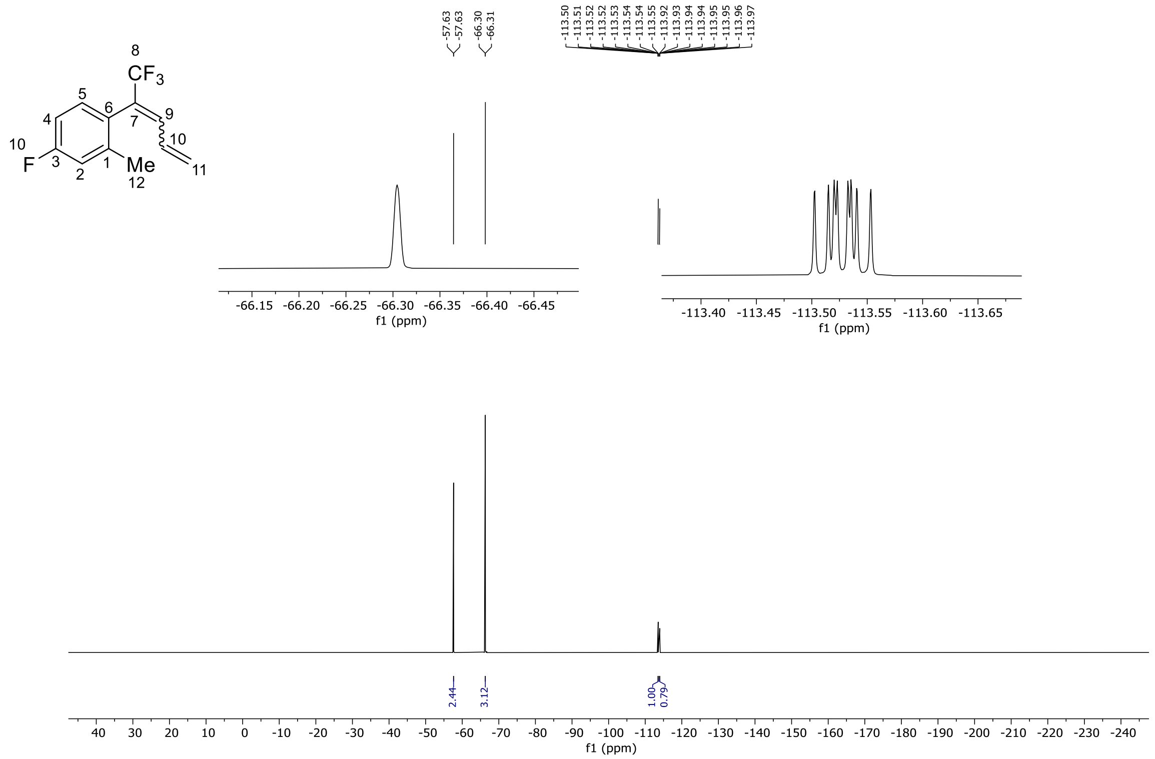

^{19}F{^{1}H} NMR (376 MHz, CDCl$_3$)

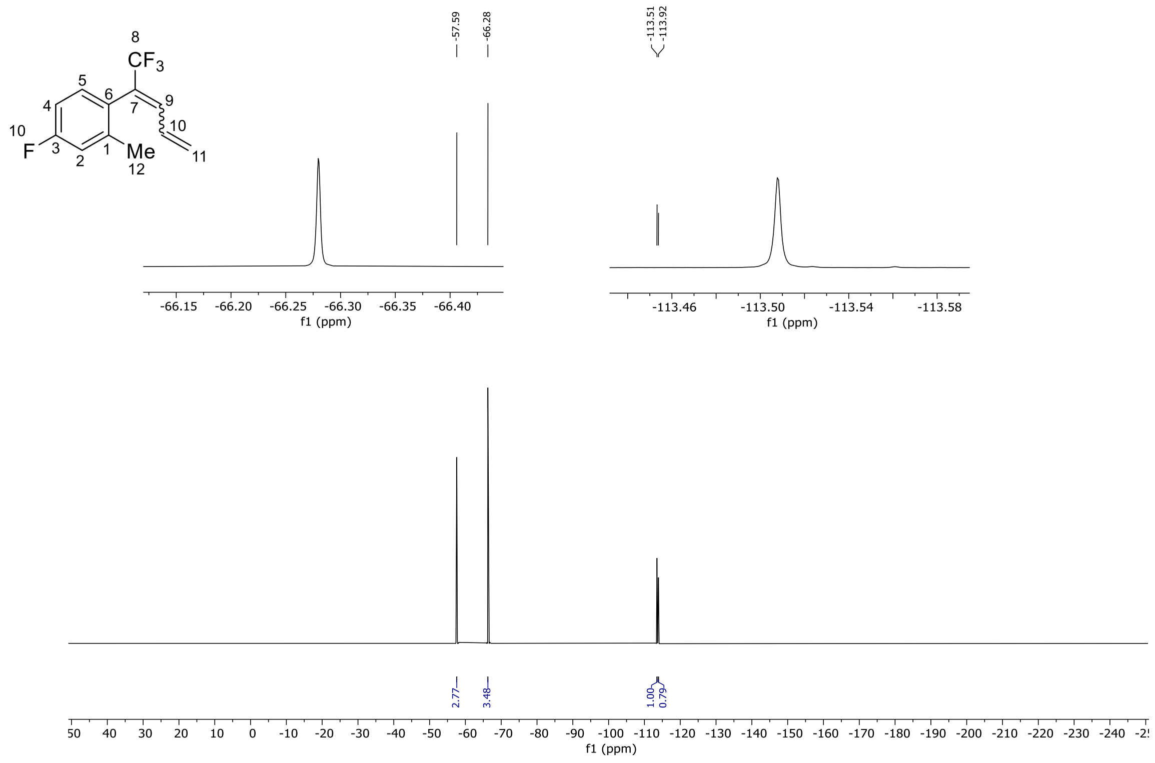

1-(Trifluoromethyl)-4-(1,1,1-trifluoropenta-2,4-dien-2-yl)benzene (1h)

^{1}H NMR (500 MHz, $CDCl_3$)

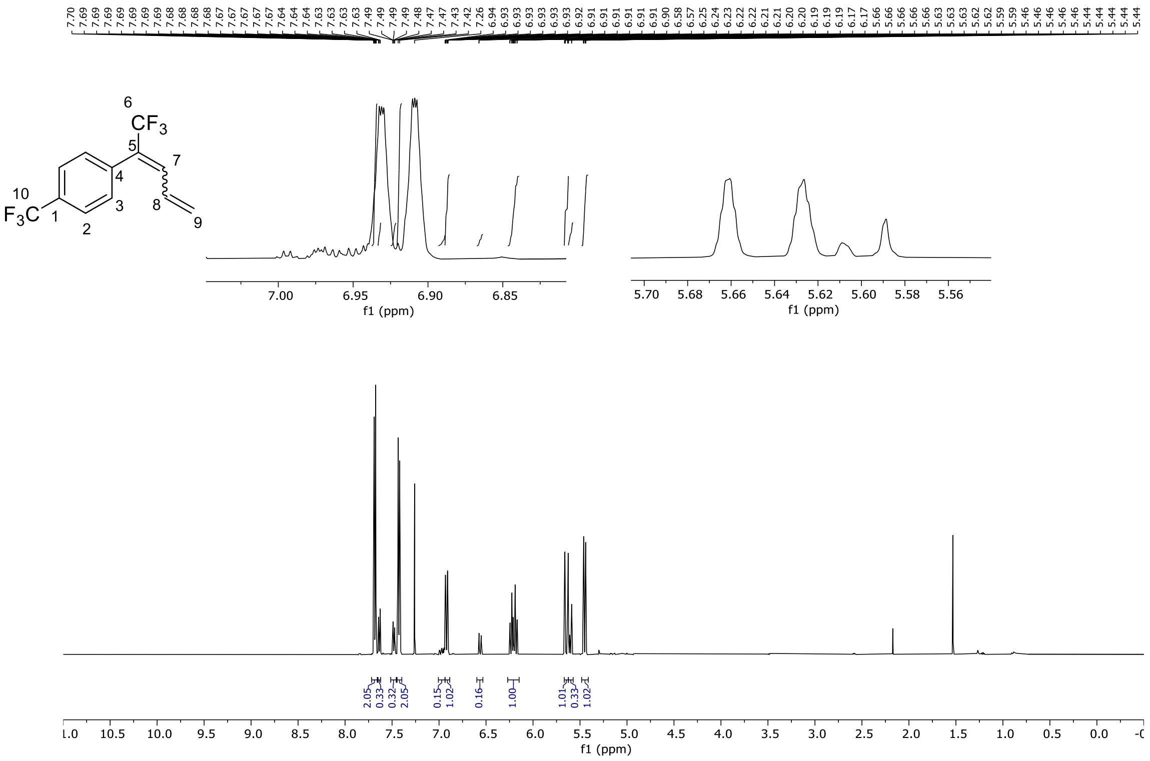

^{13}C NMR (126 MHz, $CDCl_3$)

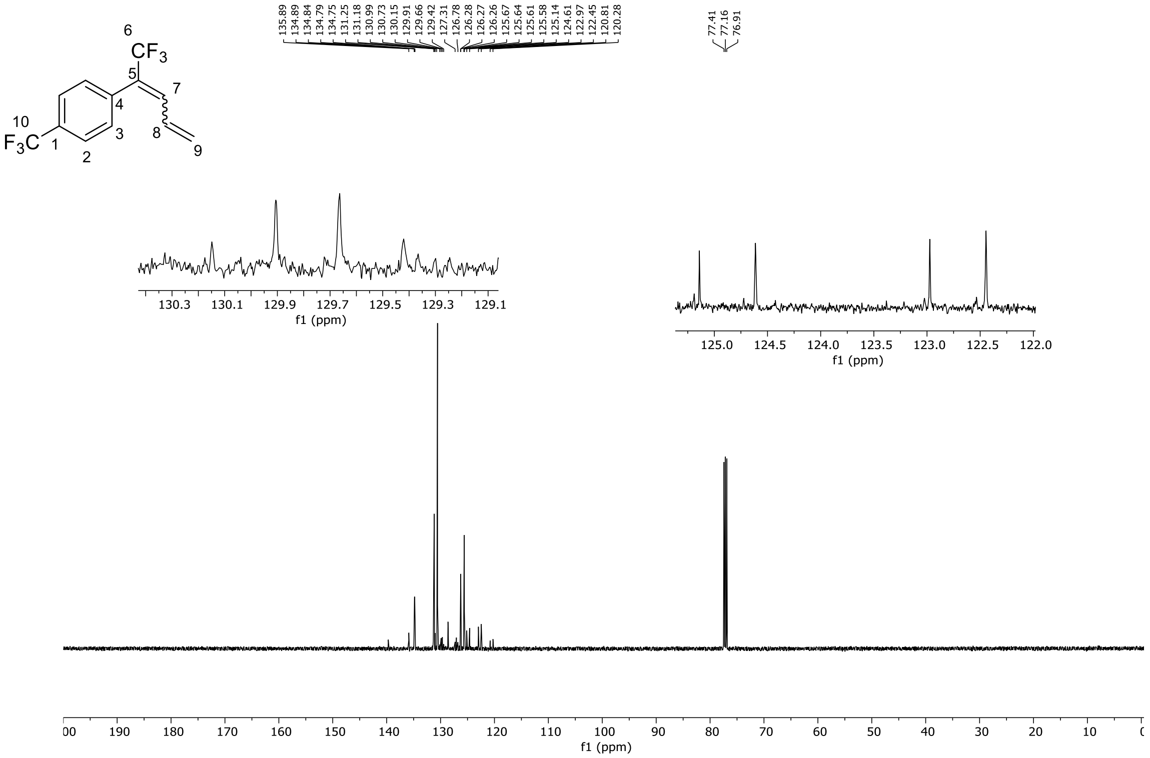

^{19}F NMR (470 MHz, $CDCl_3$)

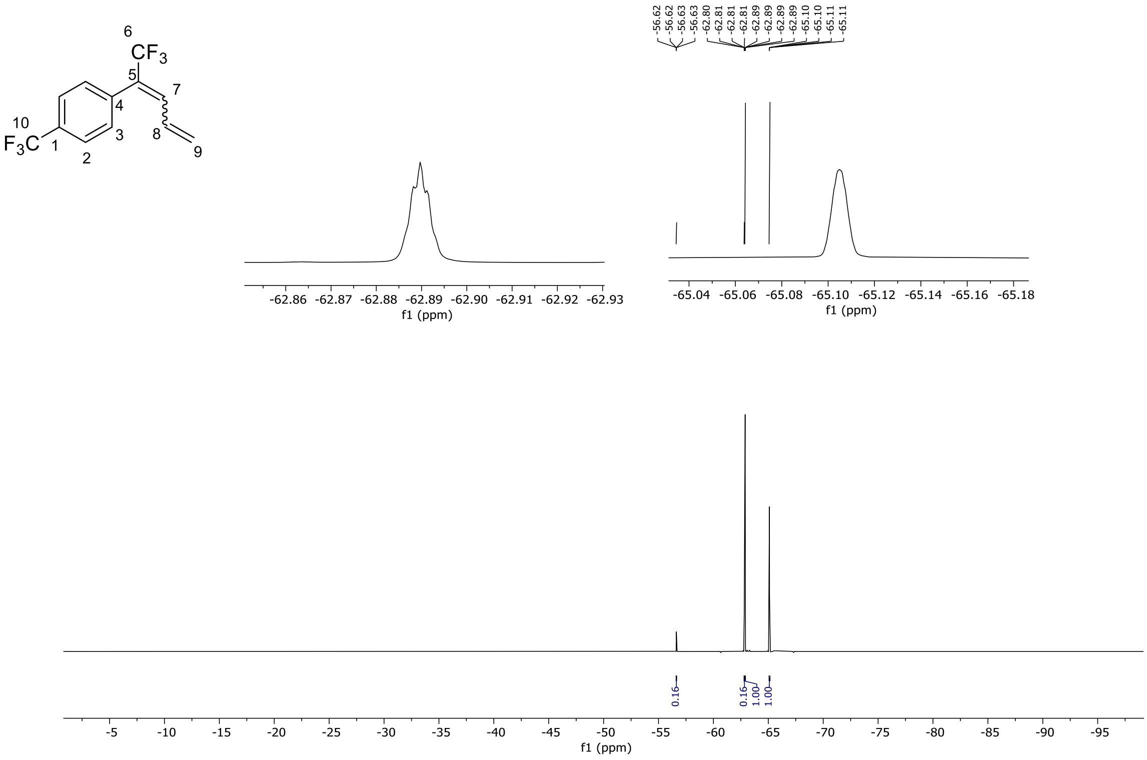

^{19}F{^{1}H} NMR (470 MHz, $CDCl_3$)

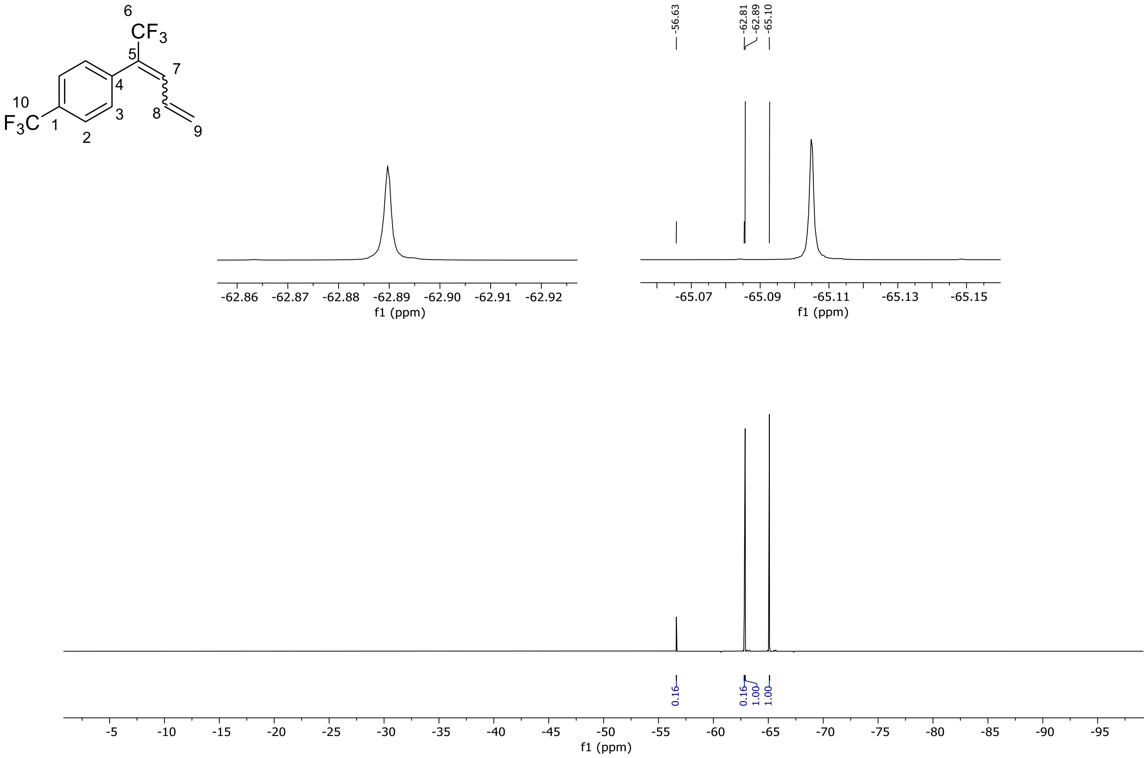

1-Methyl-4-(1,1,1-trifluoropenta-2,4-dien-2-yl)benzene (1i)

^{1}H NMR (599 MHz, $CDCl_3$)

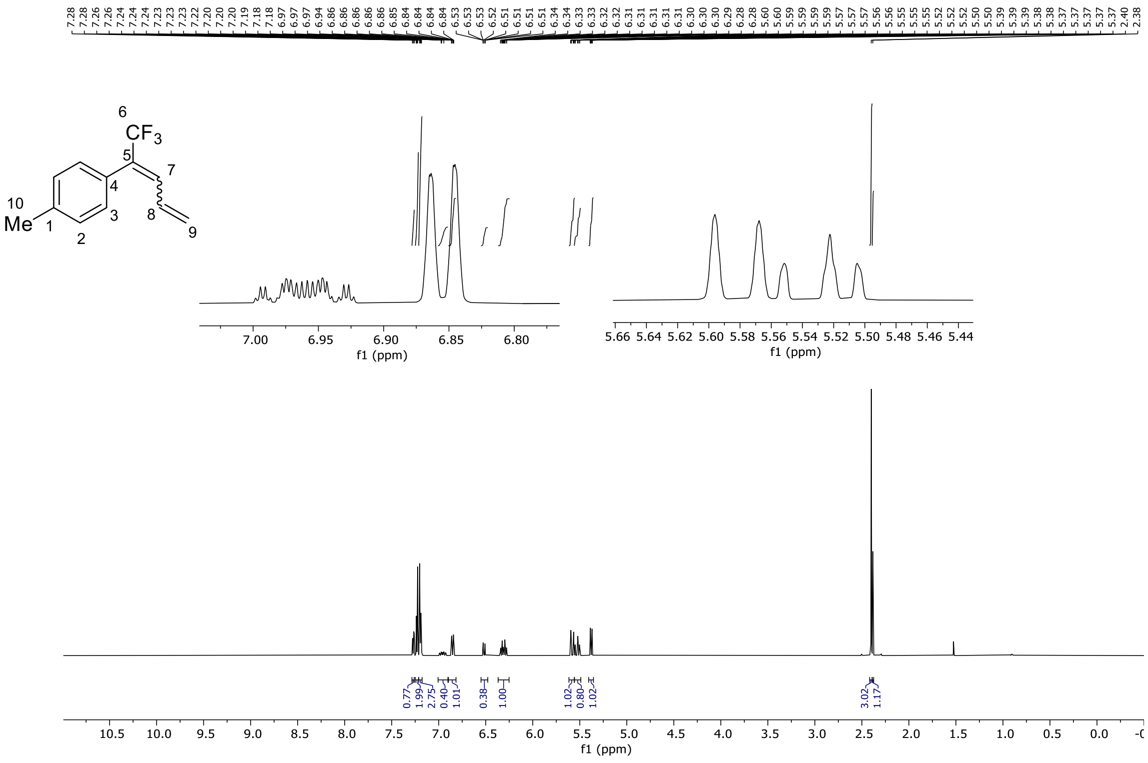

^{13}C NMR (151 MHz, $CDCl_3$)

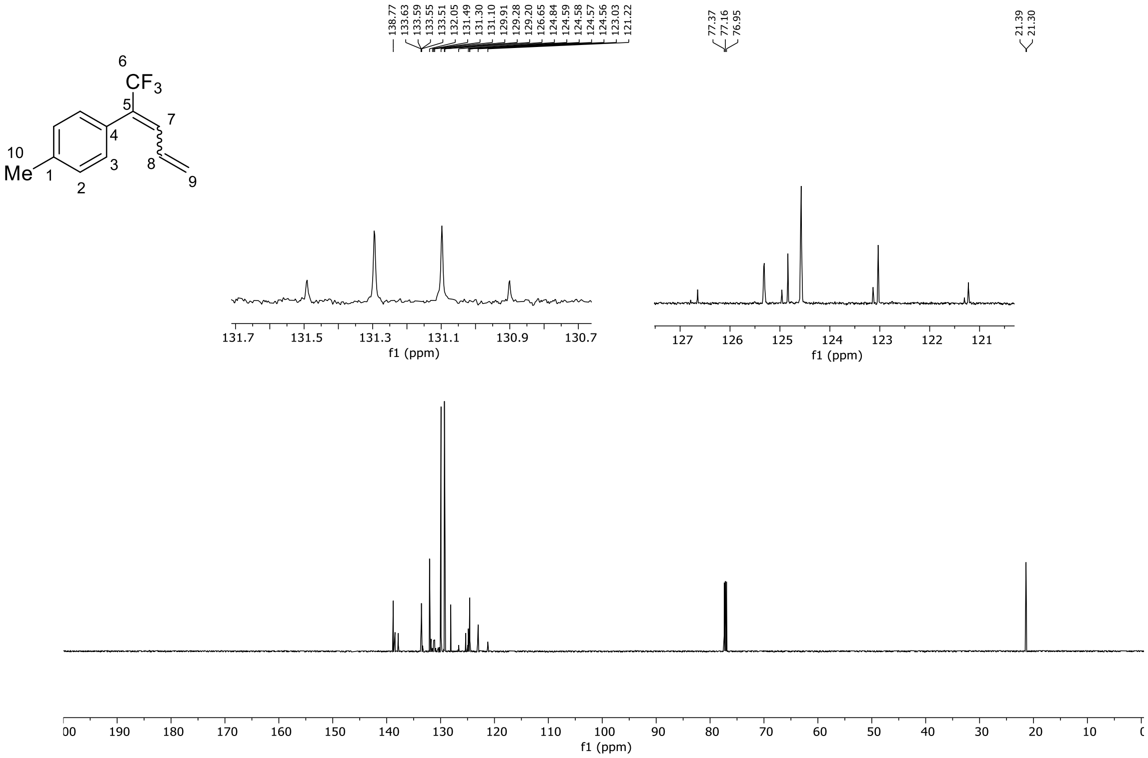

^{19}**F NMR** (564 MHz, $CDCl_3$)

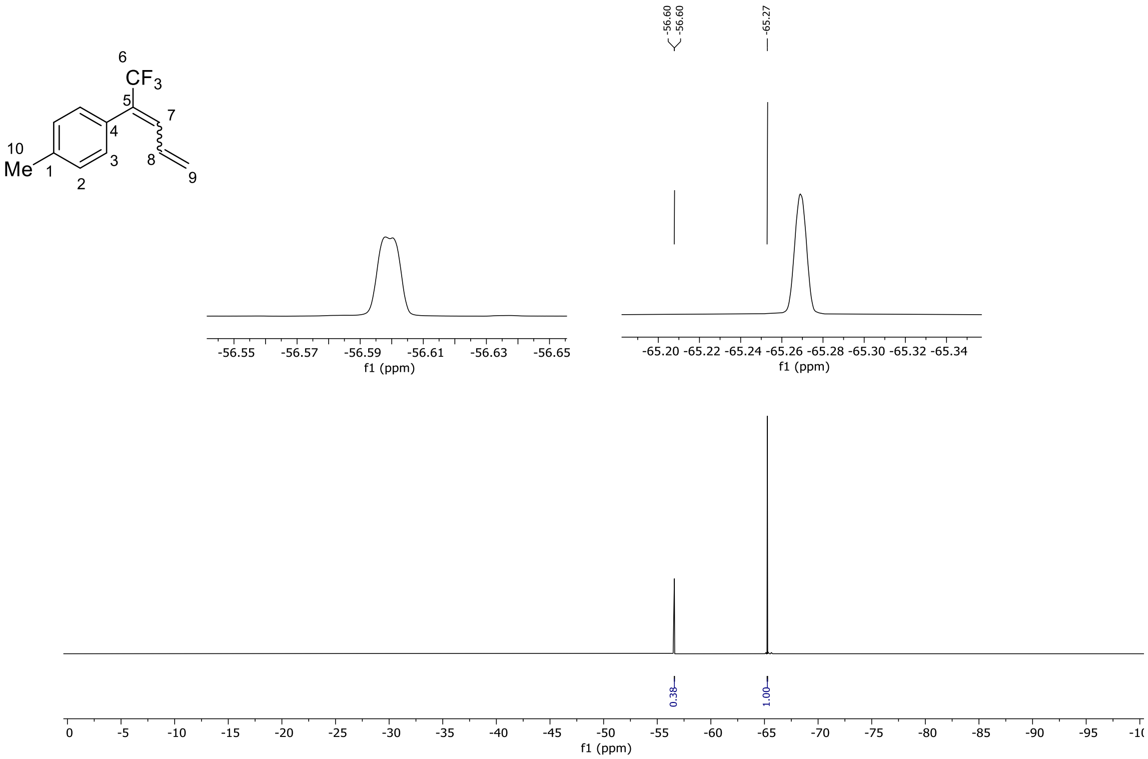

^{19}**F{^{1}H} NMR** (564 MHz, $CDCl_3$)

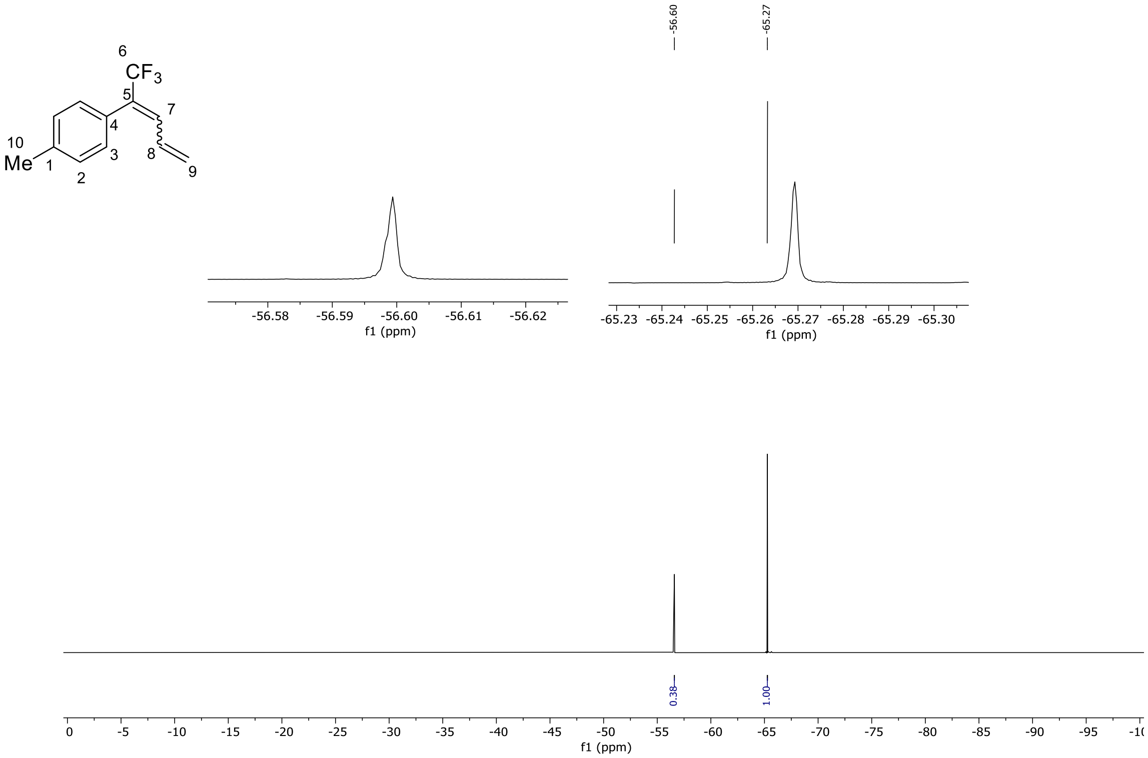

1,1,1-Trifluoro-2-(4-nitrophenyl)pent-4-en-2-ol (1k-1)

^{1}H NMR (500 MHz, $CDCl_3$)

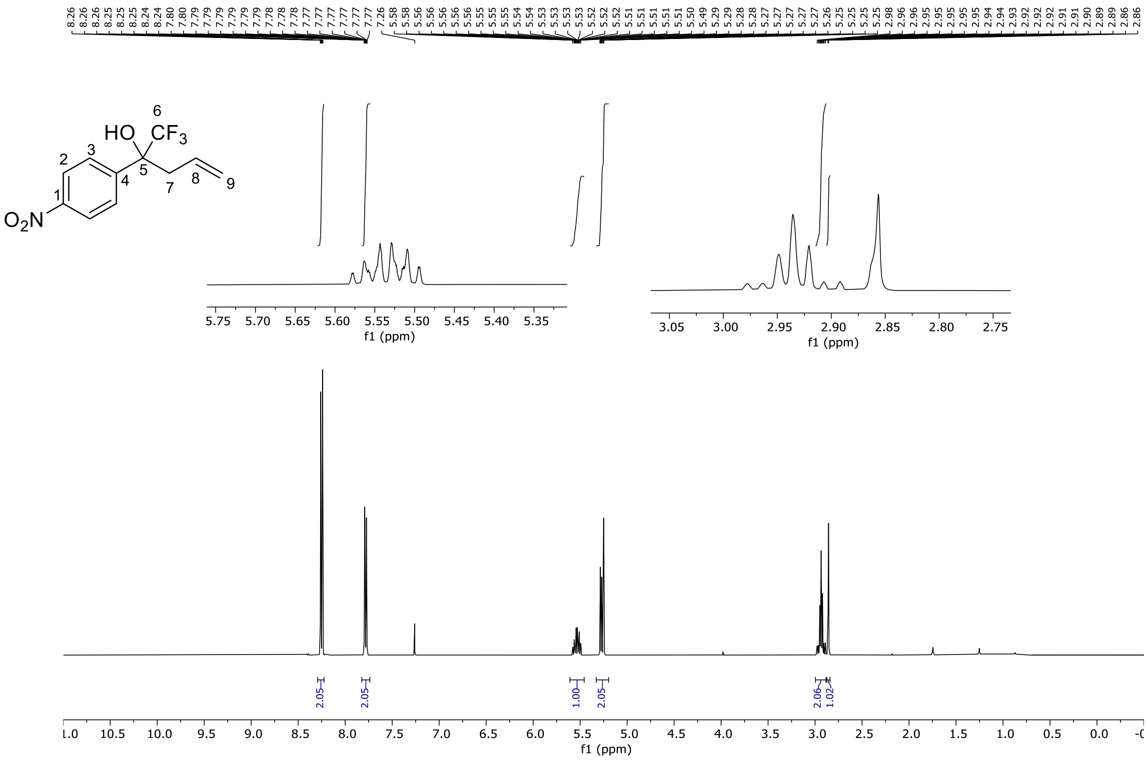

^{13}C NMR (126 MHz, $CDCl_3$)

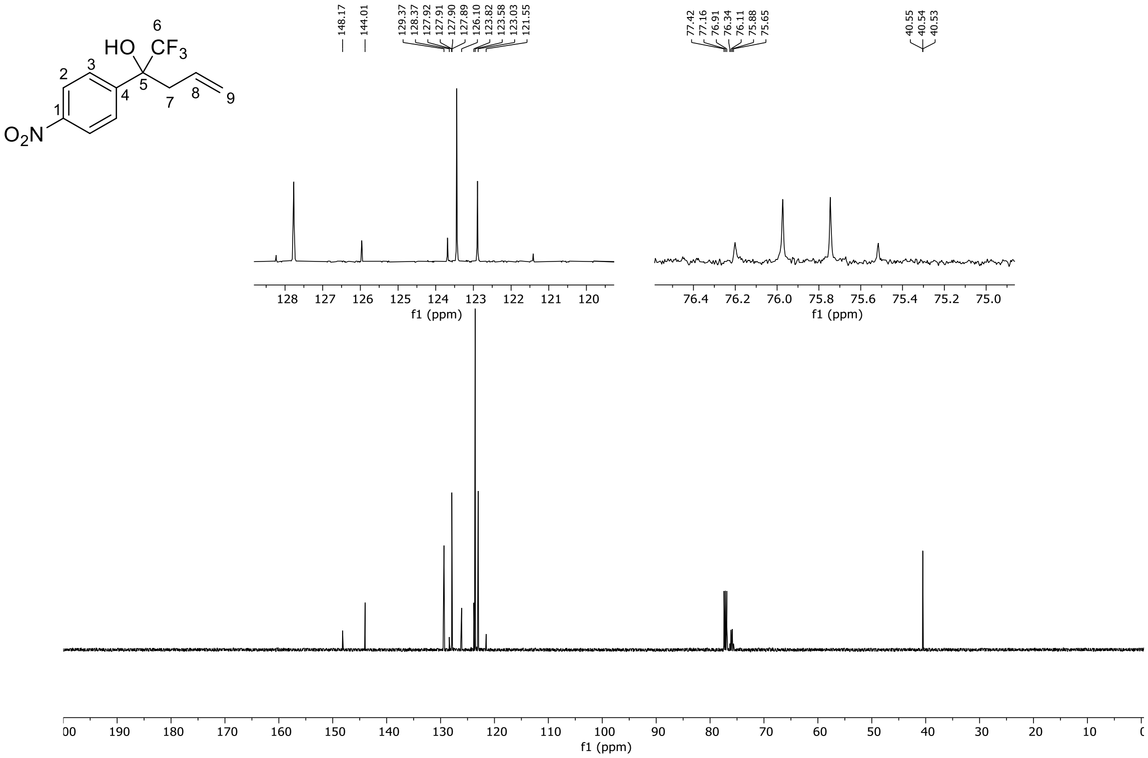

^{19}**F NMR** (470 MHz, $CDCl_3$)

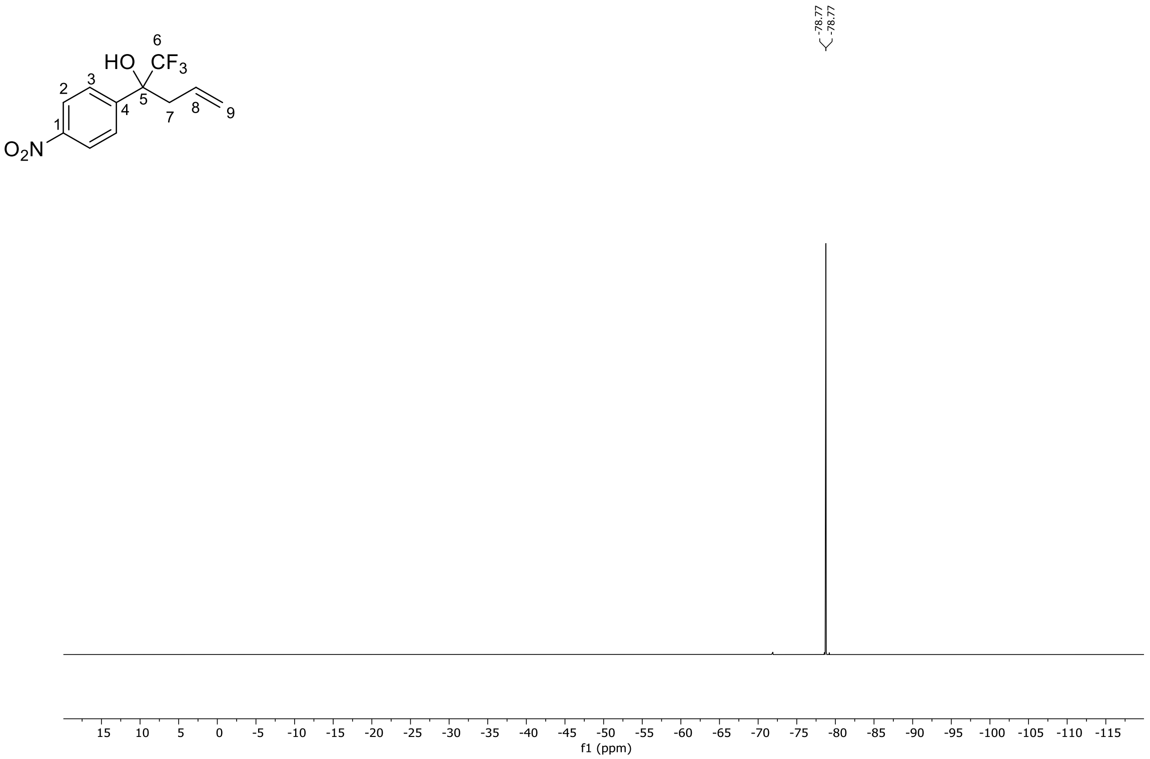

^{19}**F{^{1}H} NMR** (470 MHz, $CDCl_3$)

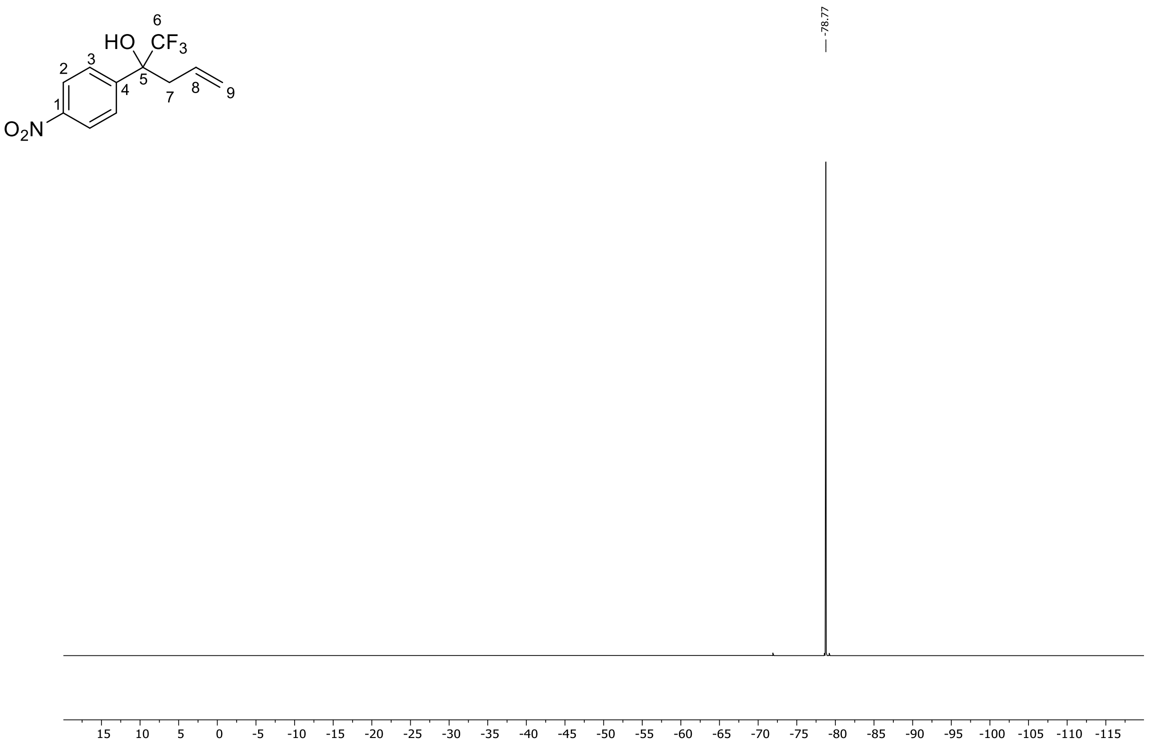

1-Nitro-4-(1,1,1-trifluoropenta-2,4-dien-2-yl)benzene (1k)

^{1}H NMR (500 MHz, $CDCl_3$)

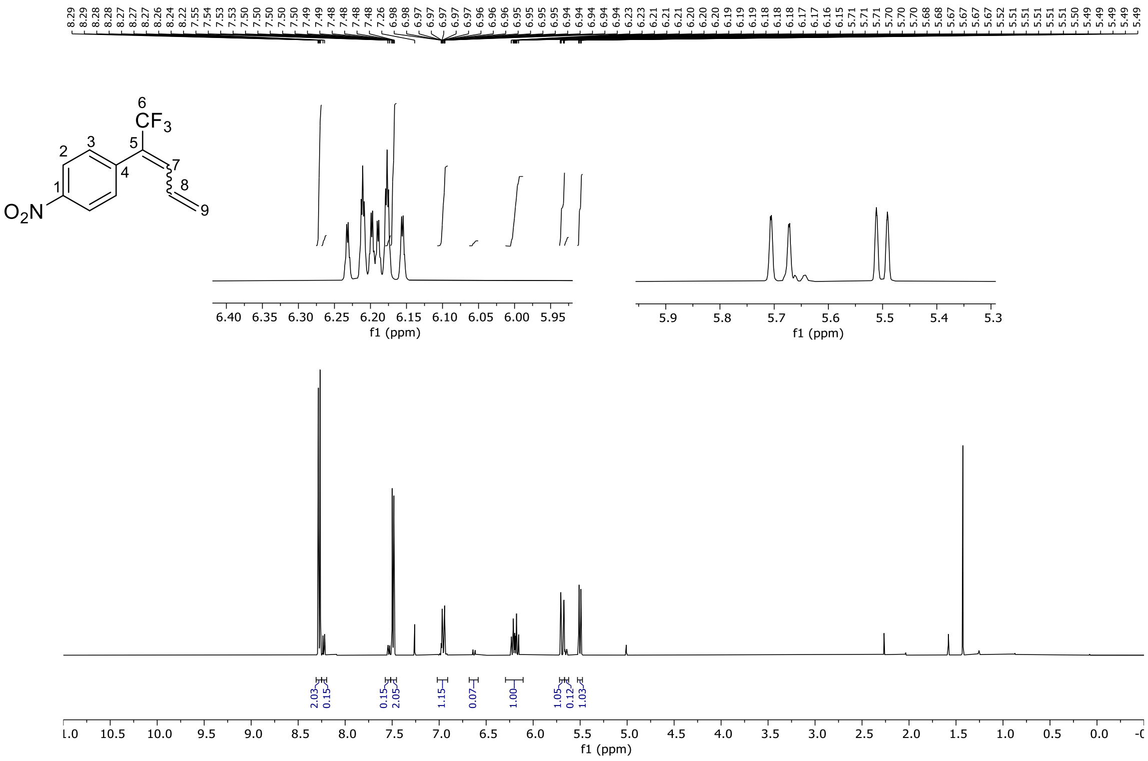

^{13}C NMR (126 MHz, $CDCl_3$)

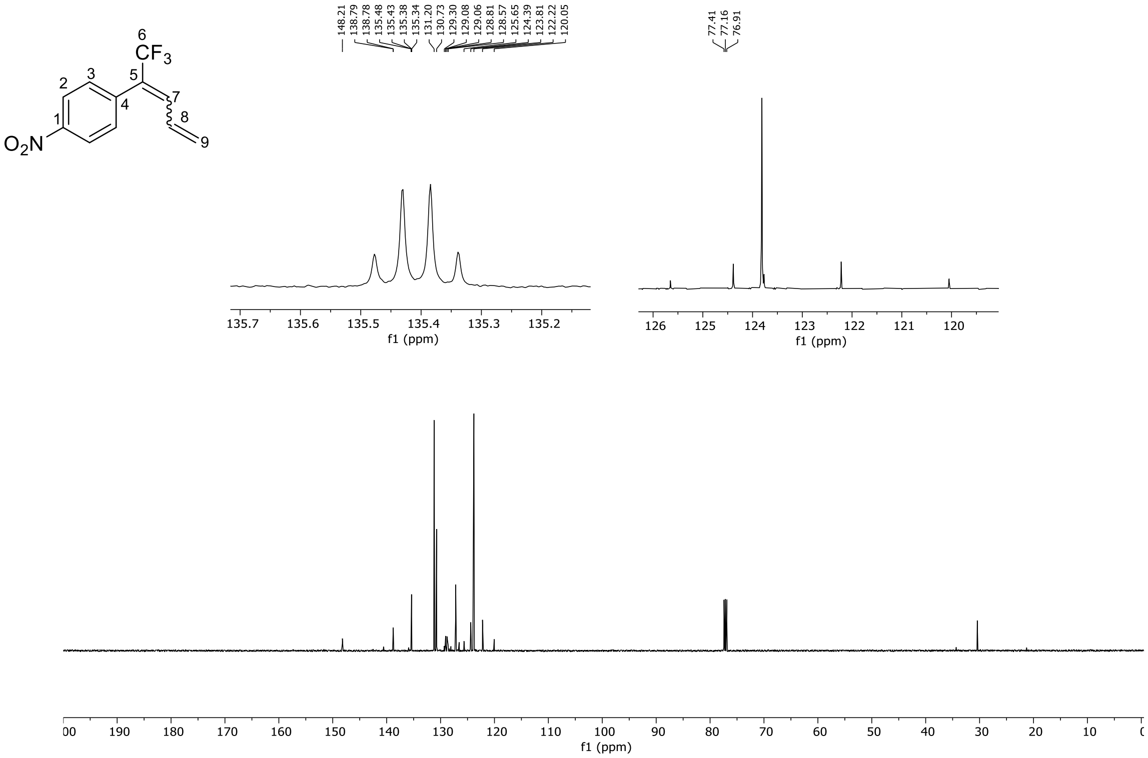

^{19}F NMR (470 MHz, $CDCl_3$)

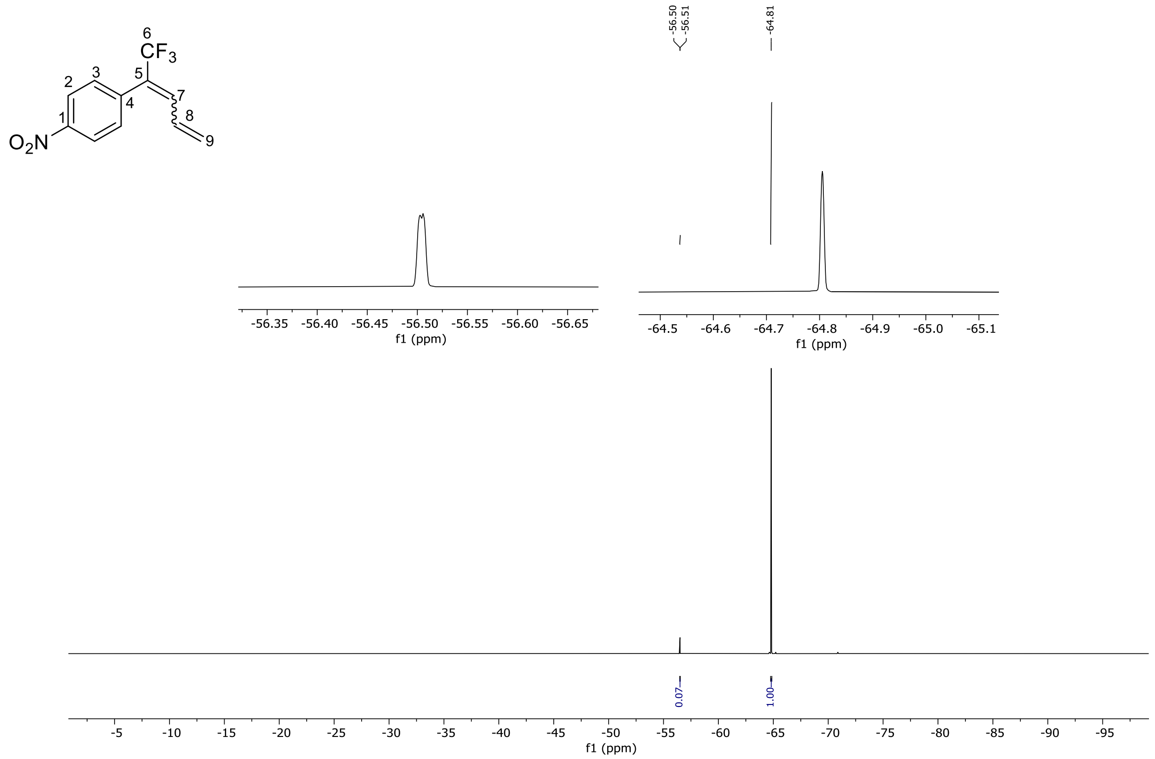

^{19}F{^{1}H} NMR (470 MHz, $CDCl_3$)

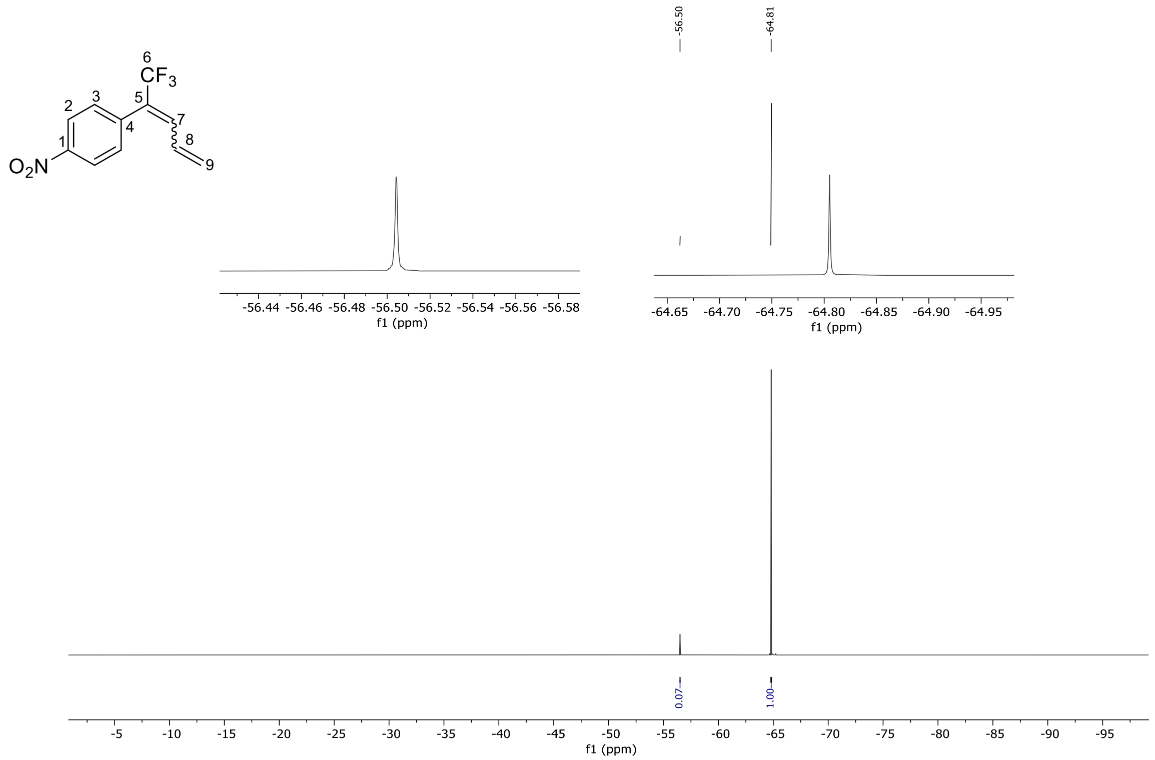

4-(1,1,1-Trifluoro-2-hydroxypent-4-en-2-yl)benzonitrile (1l-1)

^{1}H NMR (599 MHz, $CDCl_3$)

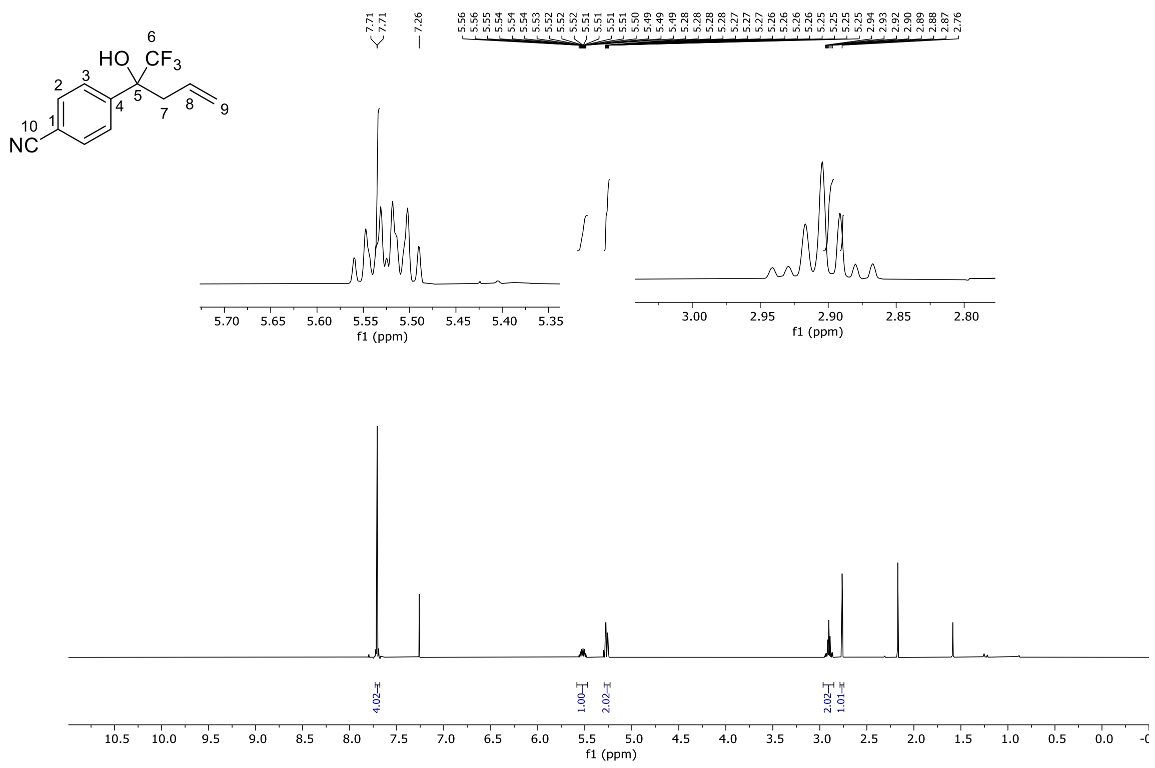

^{13}C NMR (151 MHz, $CDCl_3$)

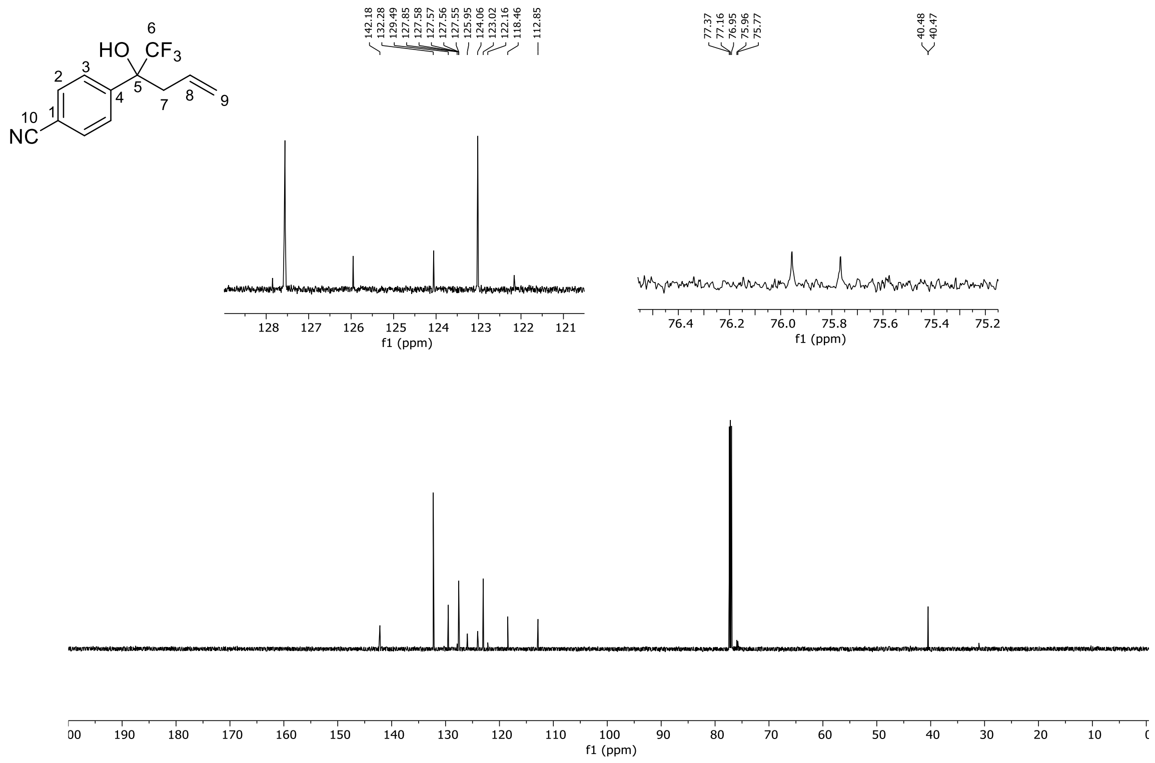

^{19}**F NMR** (564 MHz, $CDCl_3$)

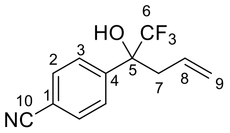

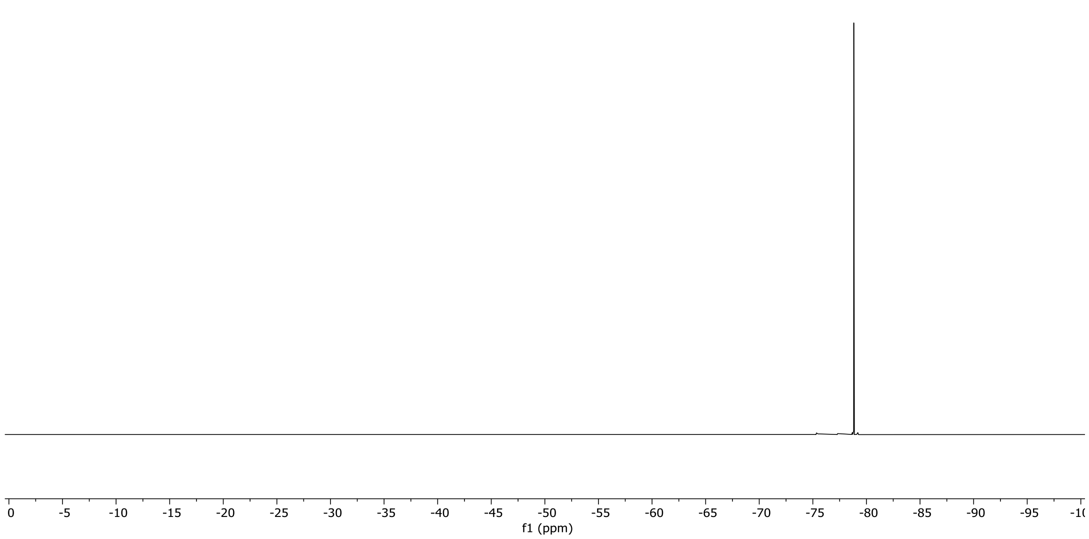

^{19}**F{^{1}H} NMR** (564 MHz, $CDCl_3$)

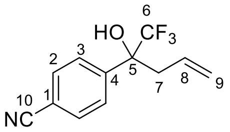

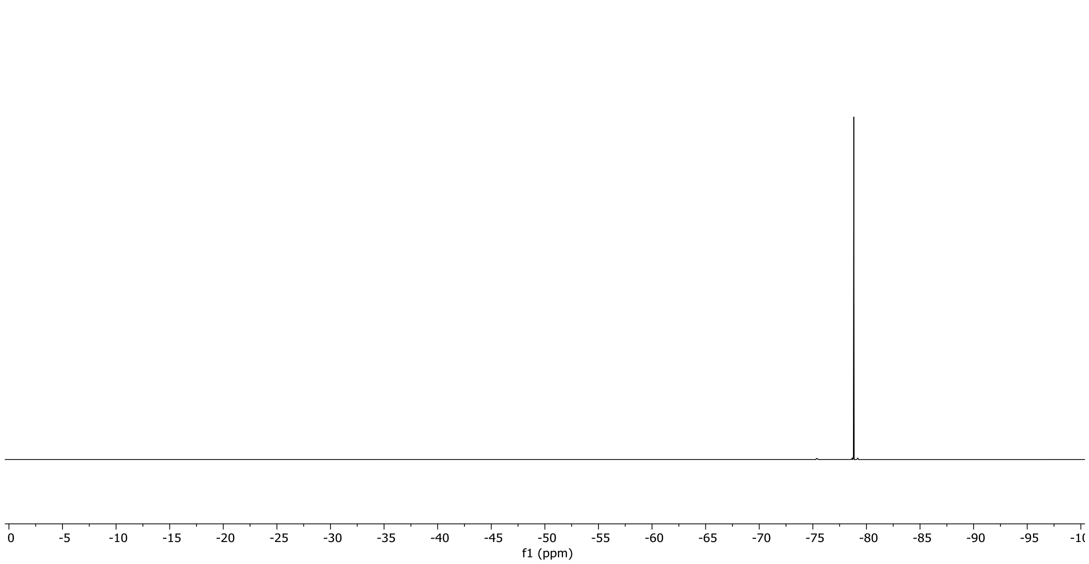

4-(1,1,1-Trifluoropenta-2,4-dien-2-yl)benzonitrile (1l)

^{1}H NMR (500 MHz, $CDCl_3$)

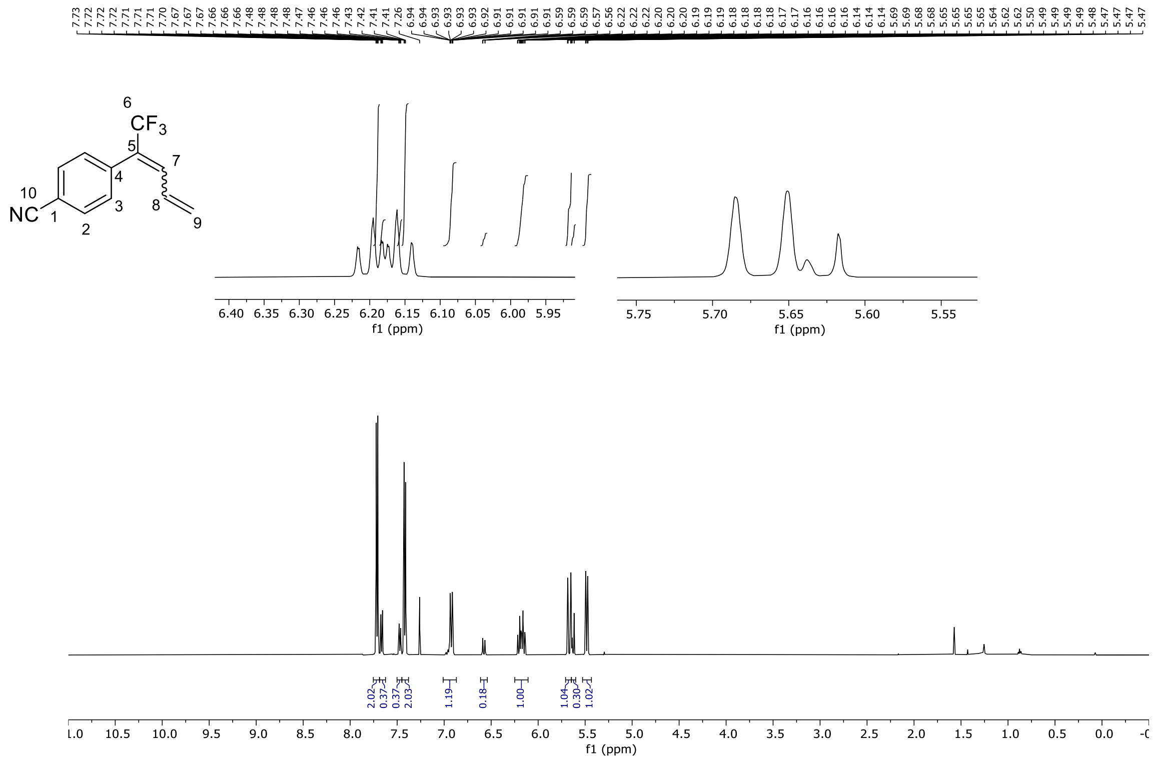

^{13}C NMR (126 MHz, $CDCl_3$)

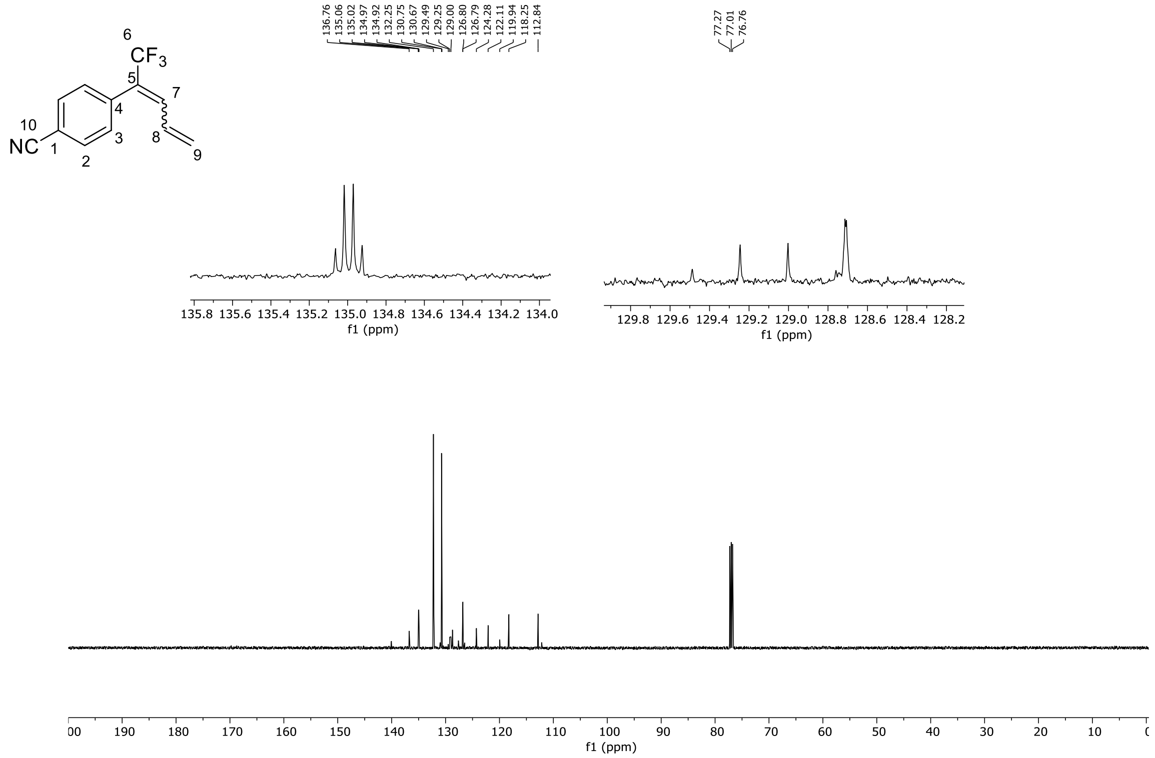

^{19}F NMR (470 MHz, $CDCl_3$)

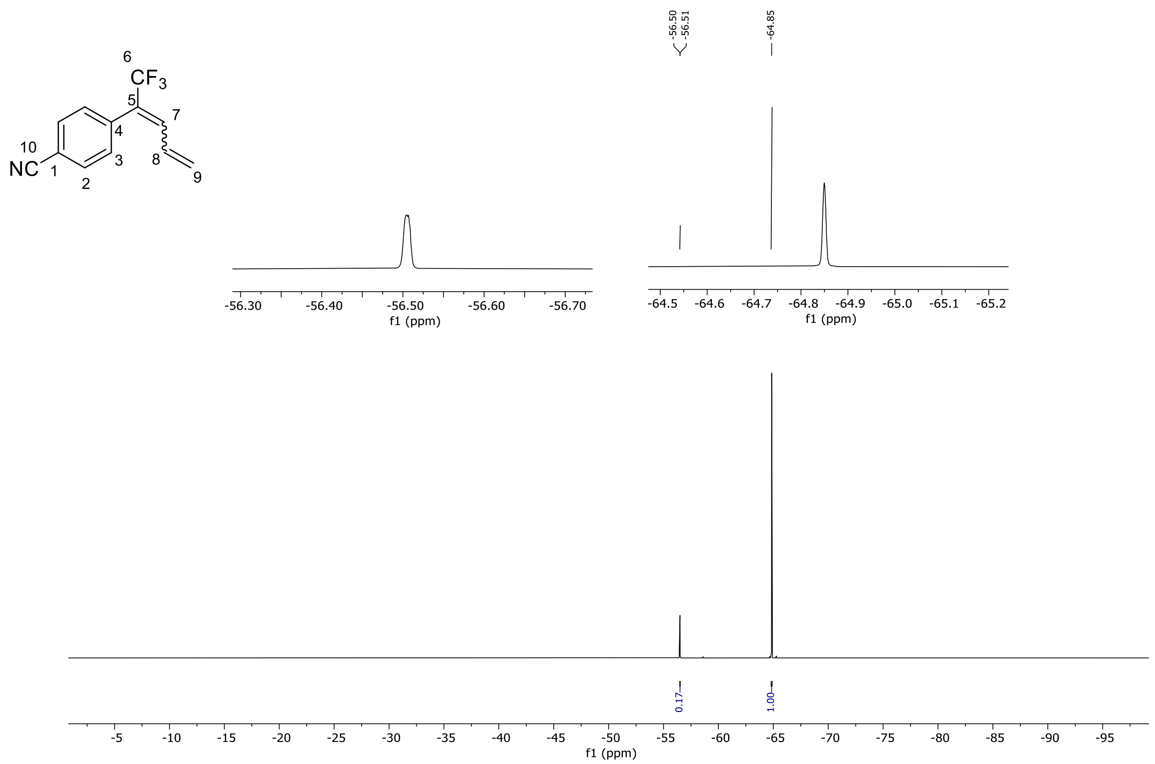

^{19}F{^{1}H} NMR (470 MHz, $CDCl_3$)

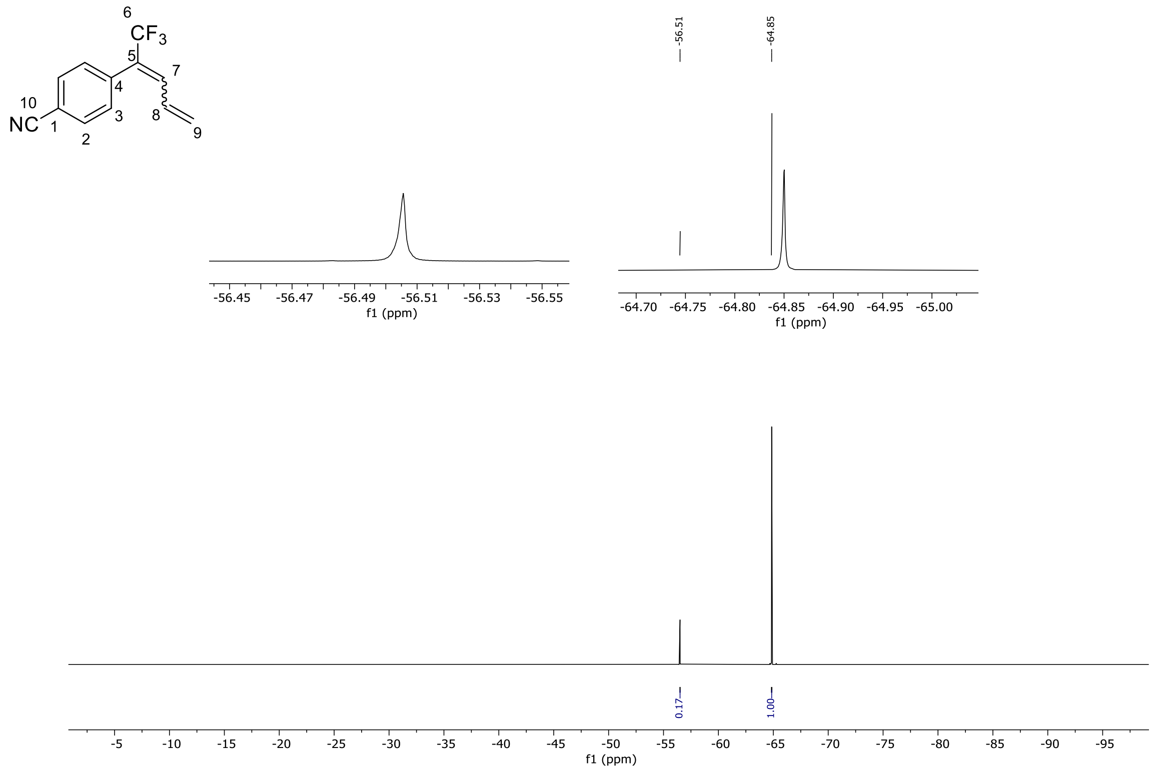

Methyl 4-(1,1,1-trifluoro-2-hydroxypent-4-en-2-yl)benzoate (1m-1)

^{1}H NMR (500 MHz, $CDCl_3$)

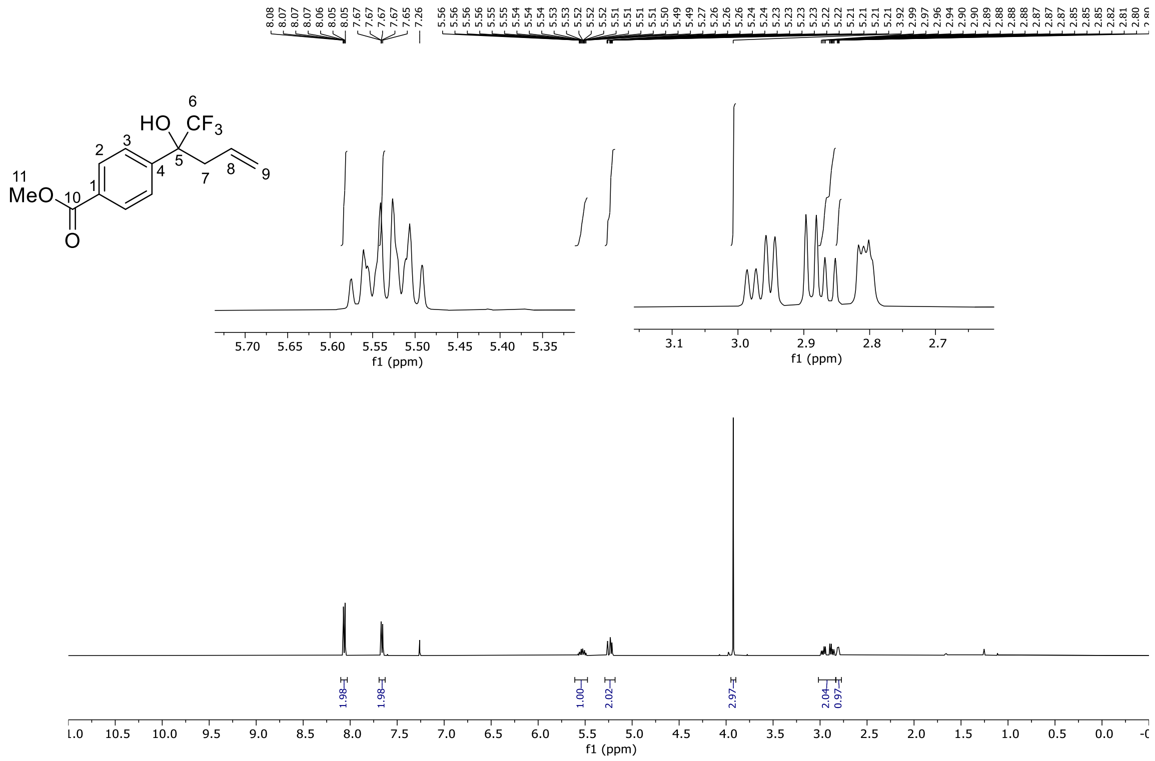

^{13}C NMR (126 MHz, $CDCl_3$)

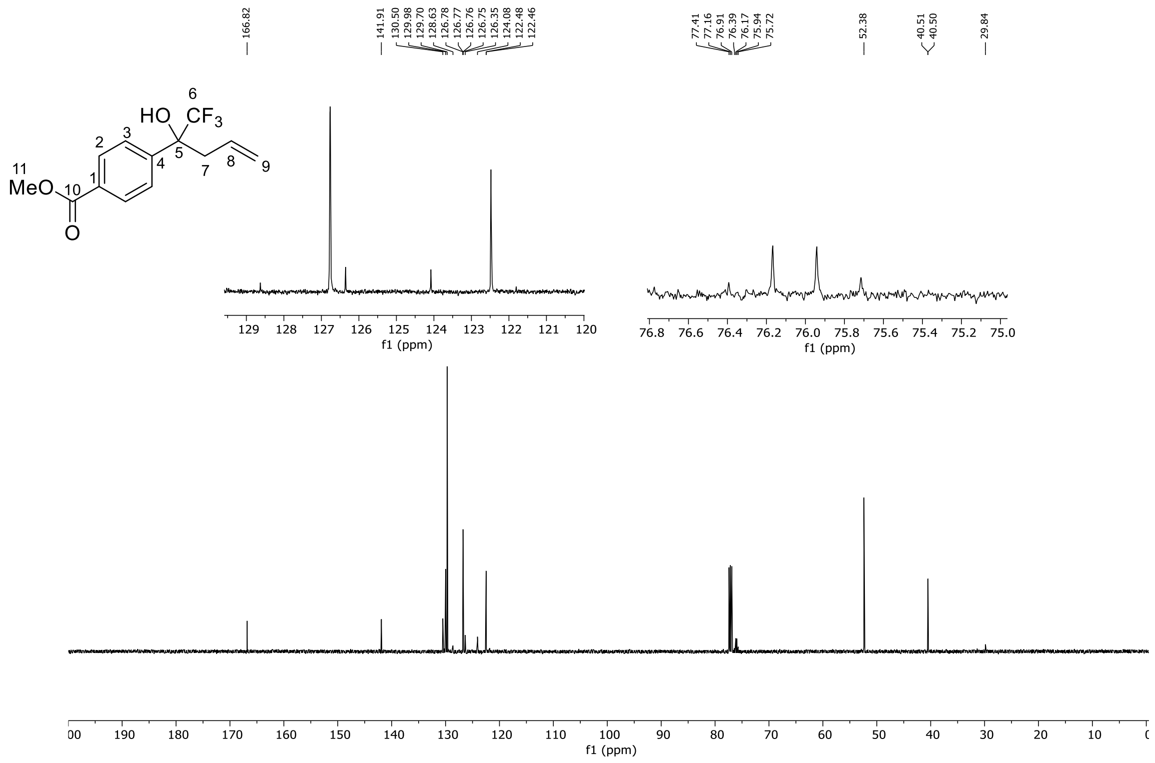

^{19}**F NMR** (470 MHz, $CDCl_3$)

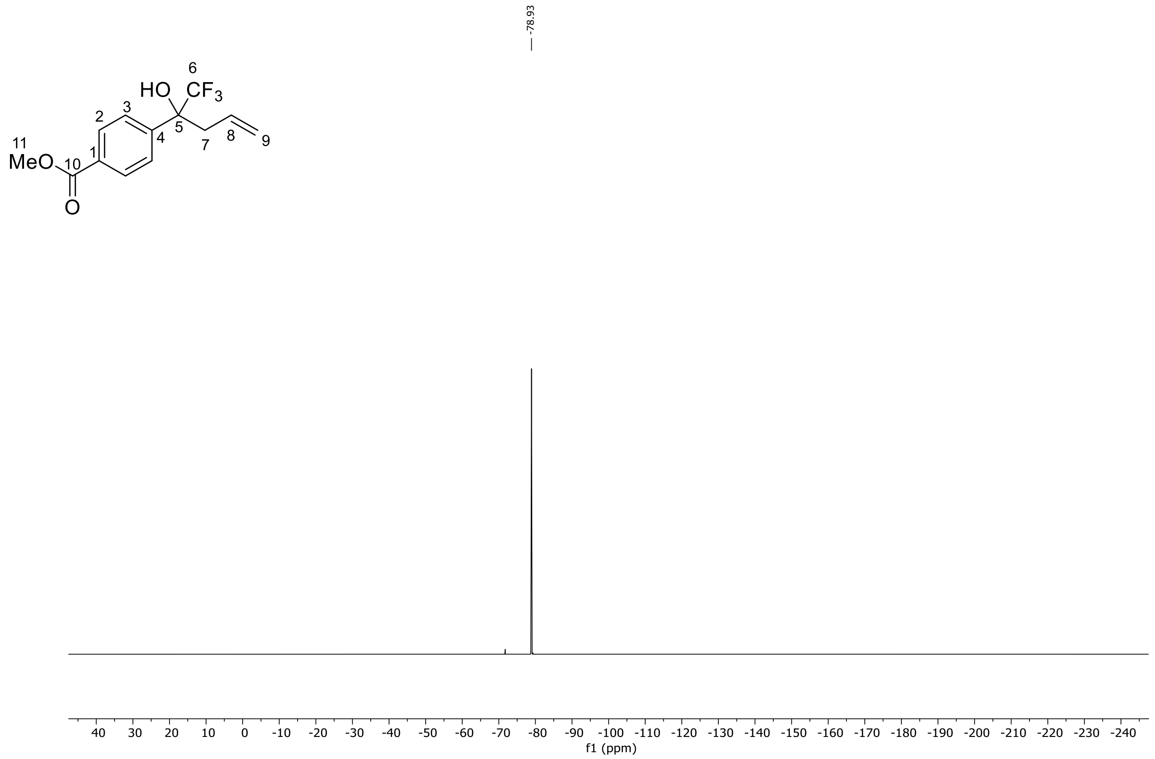

^{19}**F{^{1}H} NMR** (470 MHz, $CDCl_3$)

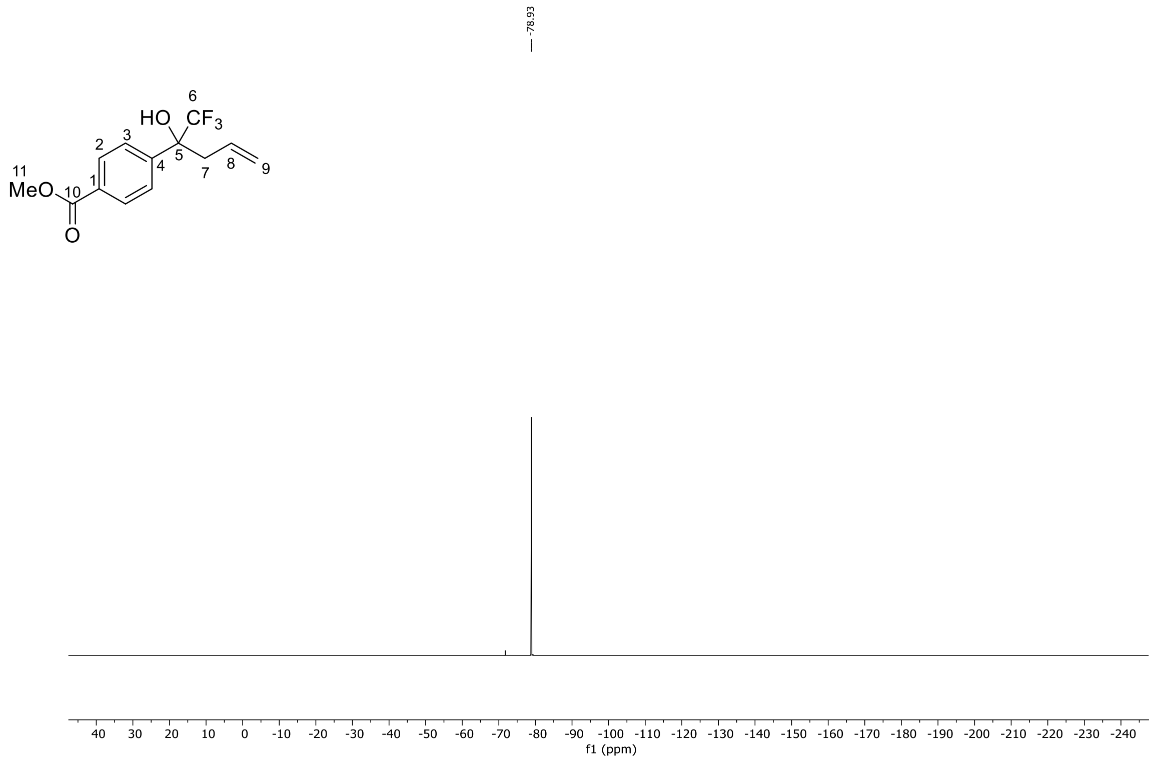

Methyl 4-(1,1,1-trifluoropenta-2,4-dien-2-yl)benzoate (1m)

^{1}H NMR (599 MHz, $CDCl_3$)

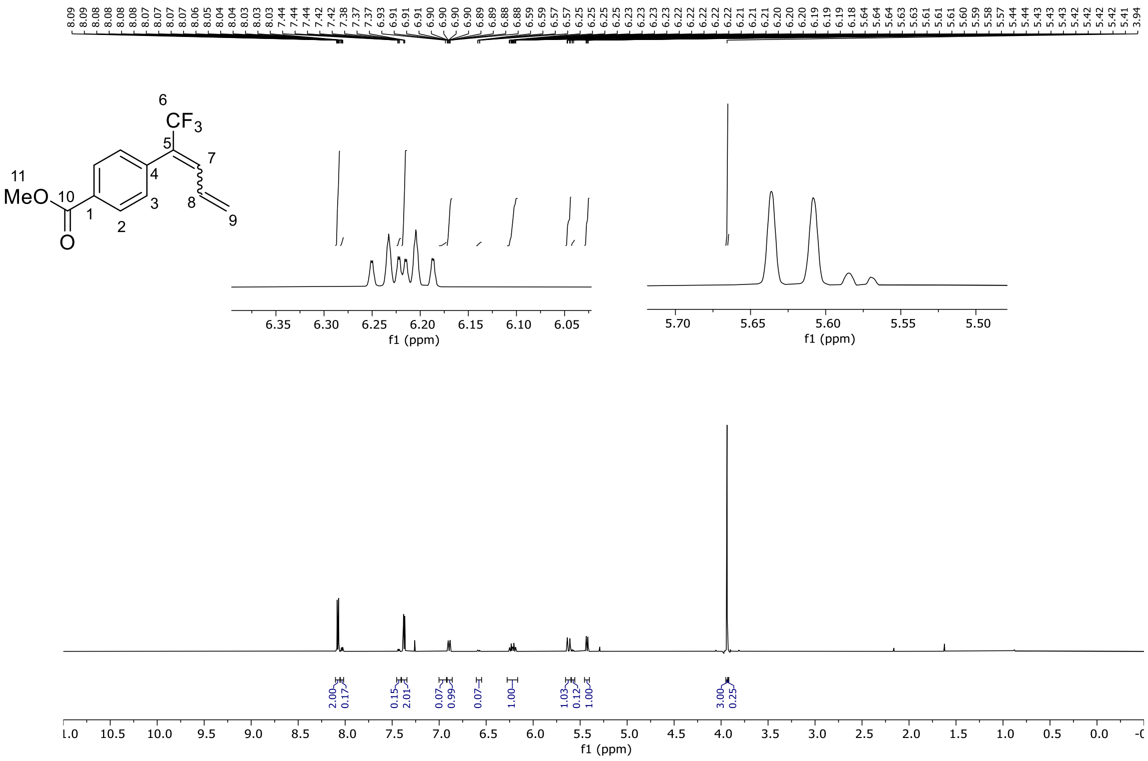

^{13}C NMR (151 MHz, $CDCl_3$)

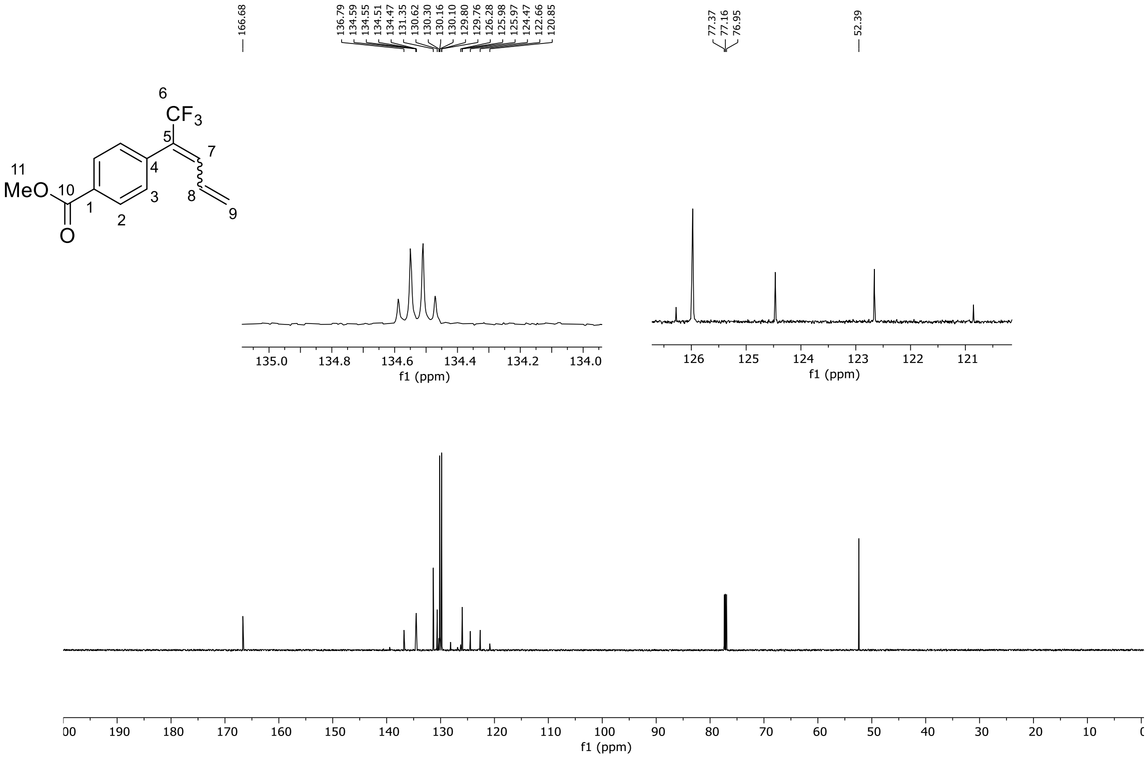

^{19}F NMR (564 MHz, $CDCl_3$)

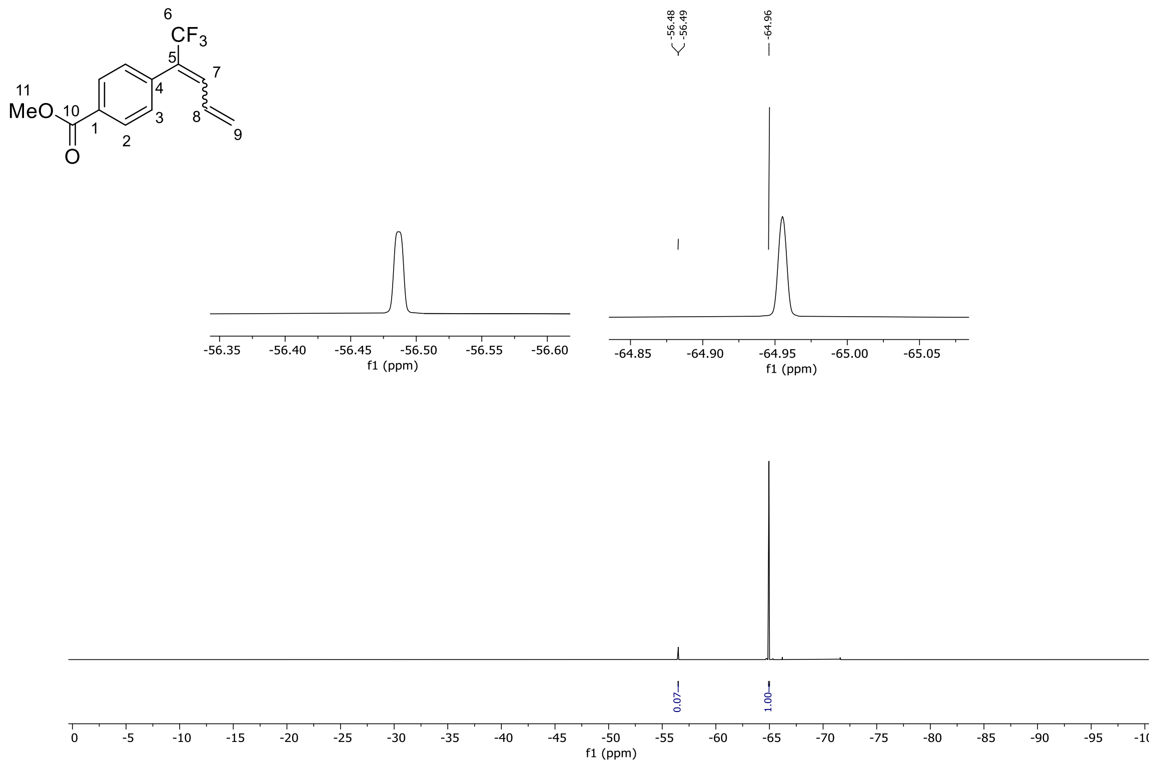

^{19}F{^{1}H} NMR (564 MHz, $CDCl_3$)

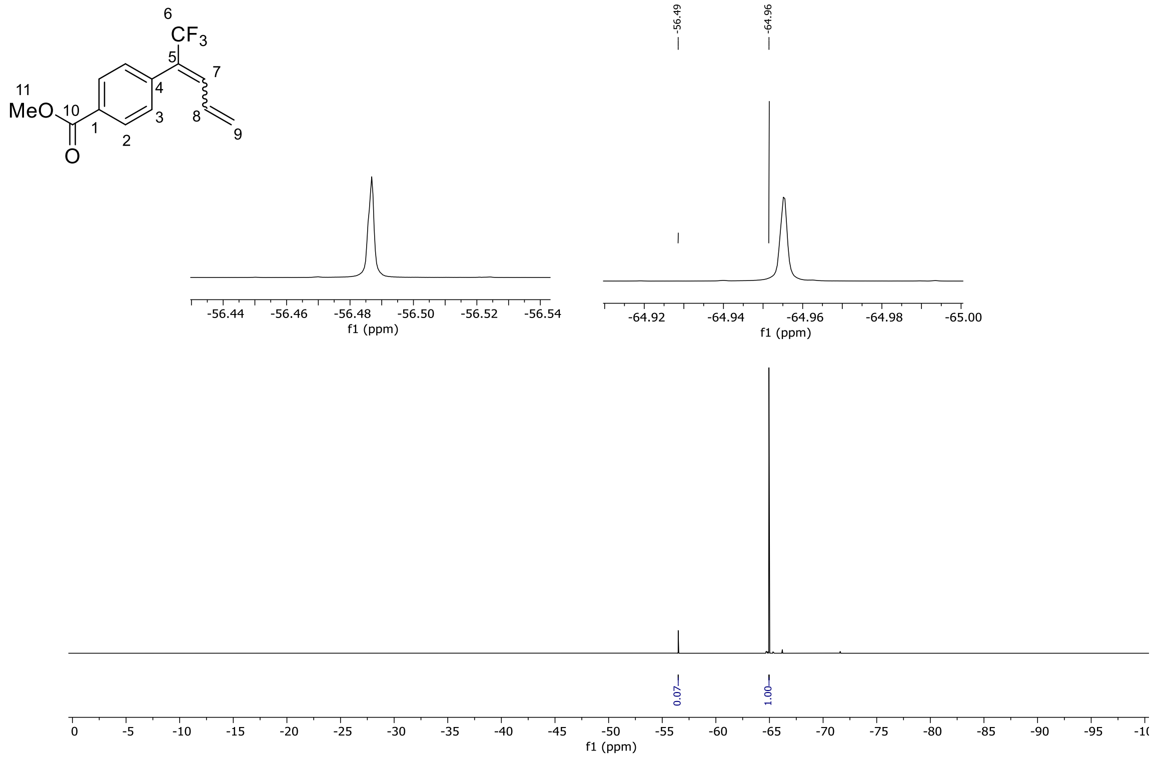

1,1,1-Trifluoro-2-(4-(methylsulfonyl)phenyl)pent-4-en-2-ol (1n-1)

^{1}H NMR (500 MHz, $CDCl_3$)

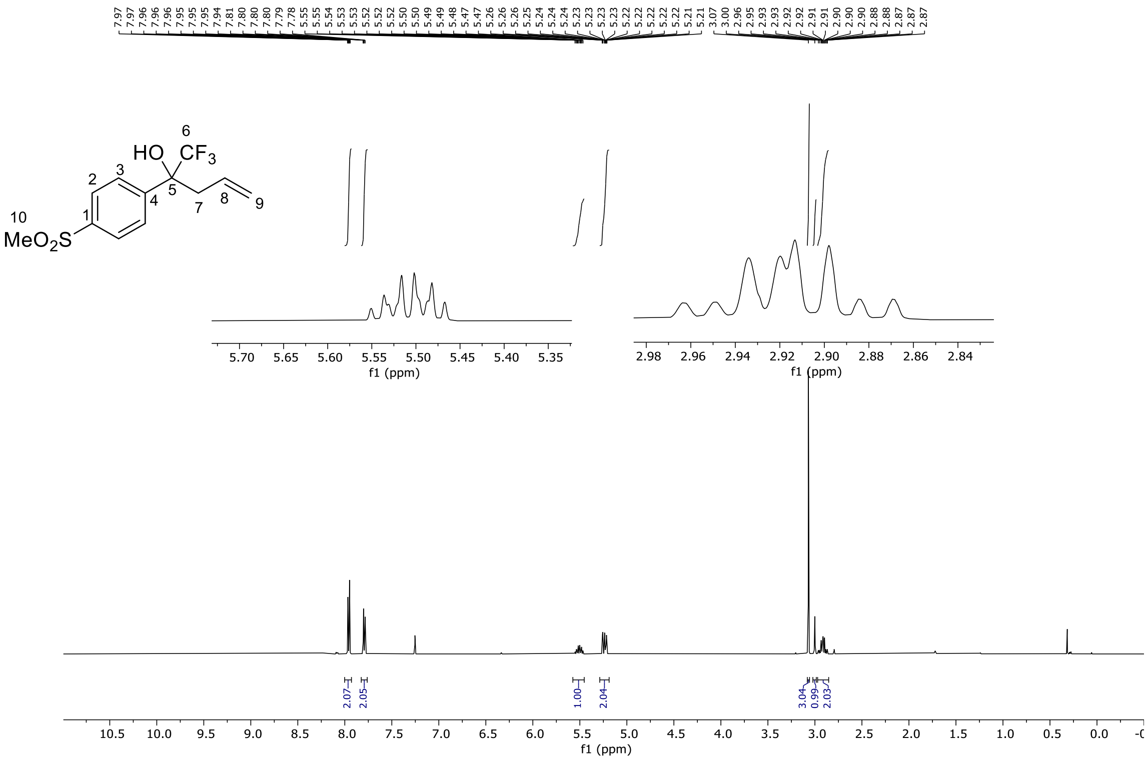

^{13}C NMR (126 MHz, $CDCl_3$)

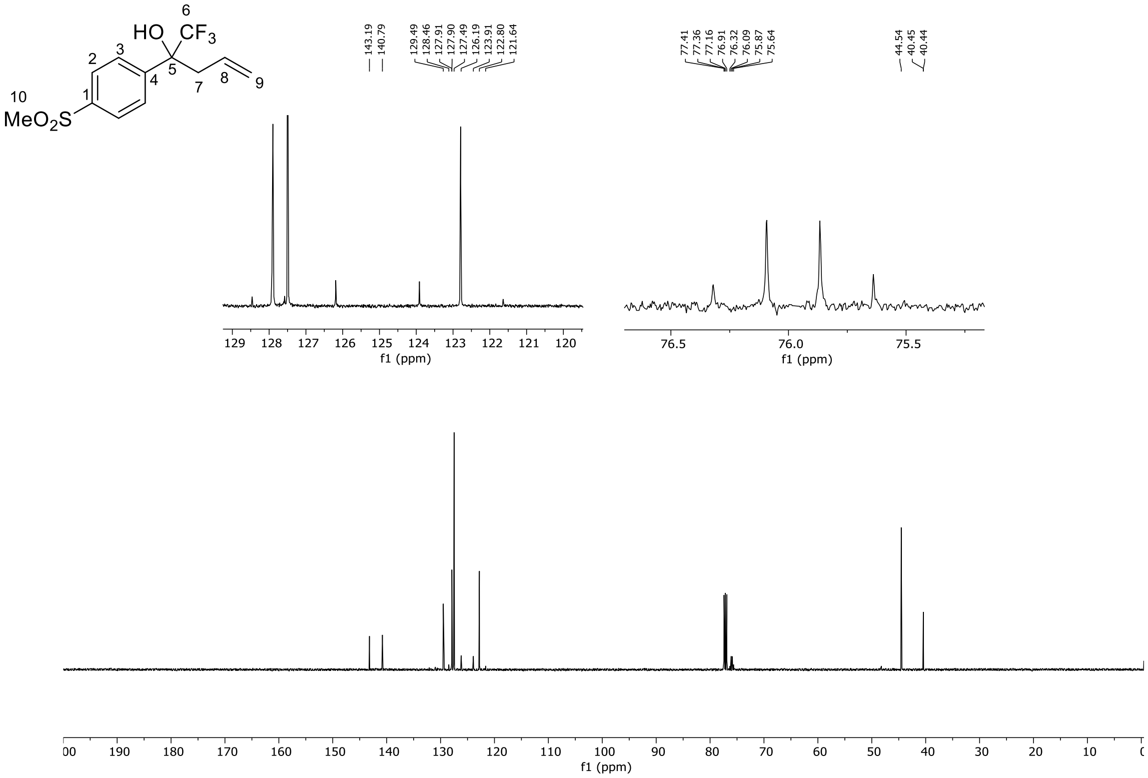

^{19}F NMR (470 MHz, $CDCl_3$

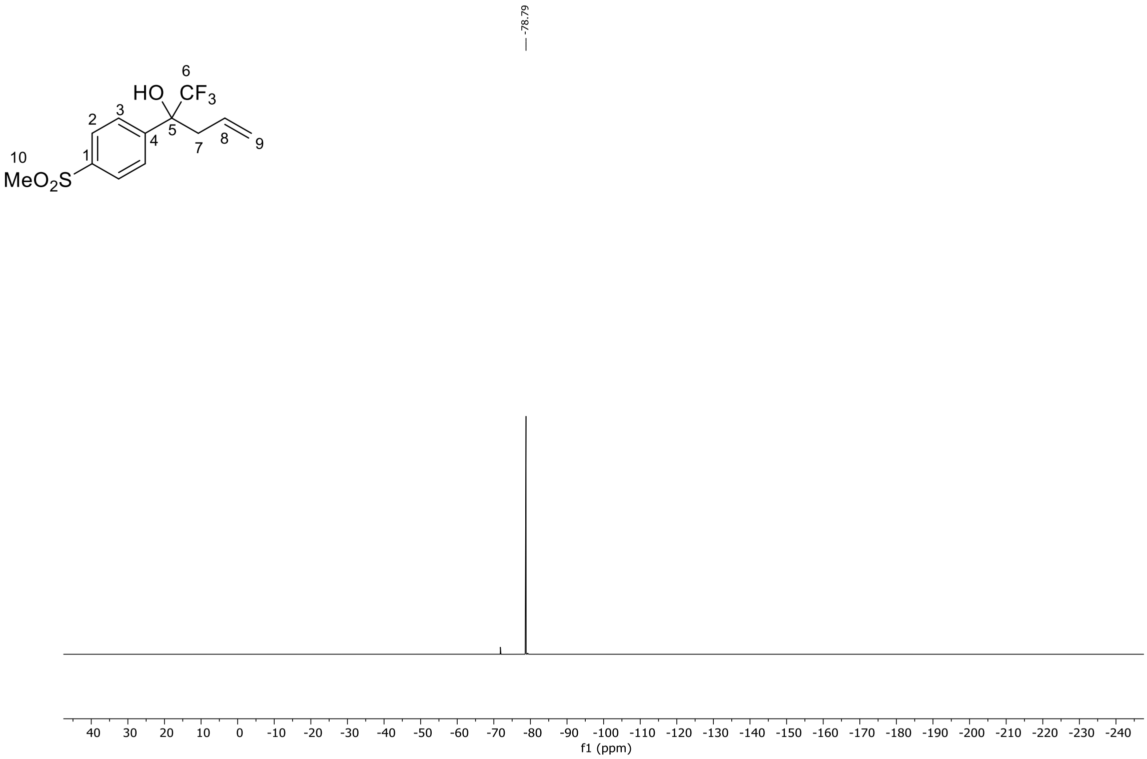

^{19}F{^{1}H} NMR (470 MHz, $CDCl_3$)

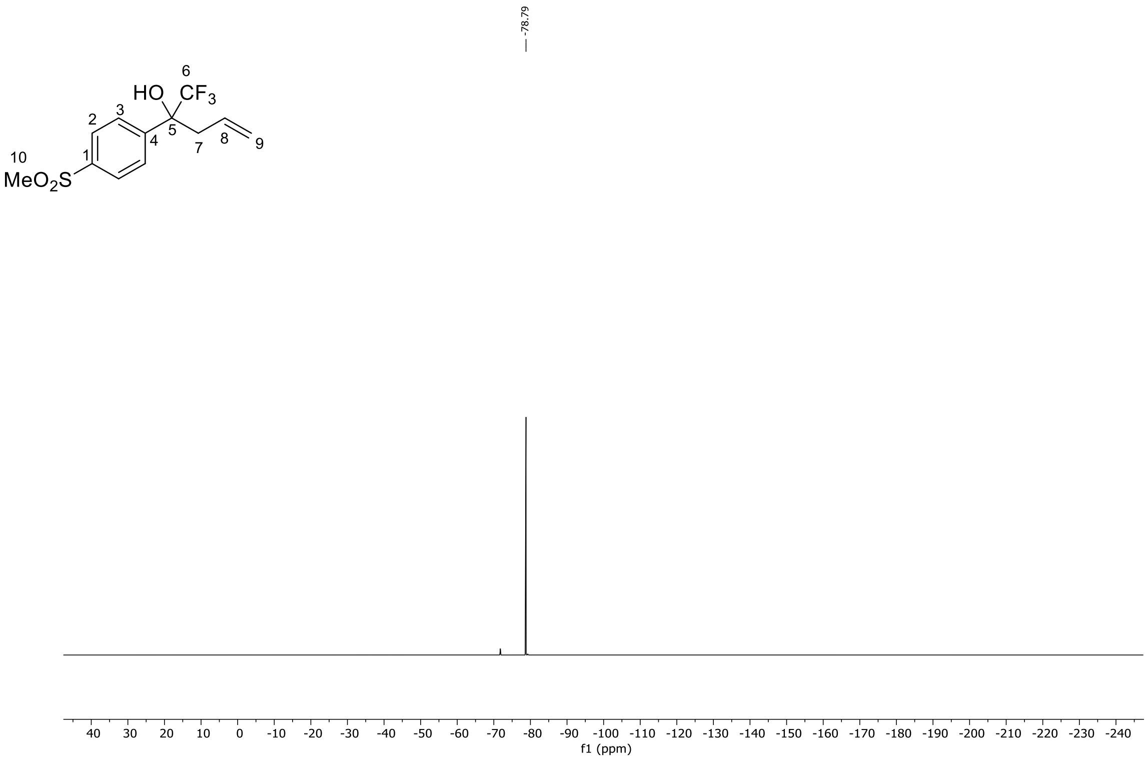

(*E*)-1-(Methylsulfonyl)-4-(1,1,1-trifluoropenta-2,4-dien-2-yl)benzene (1n)

^{1}H NMR (599 MHz, $CDCl_3$)

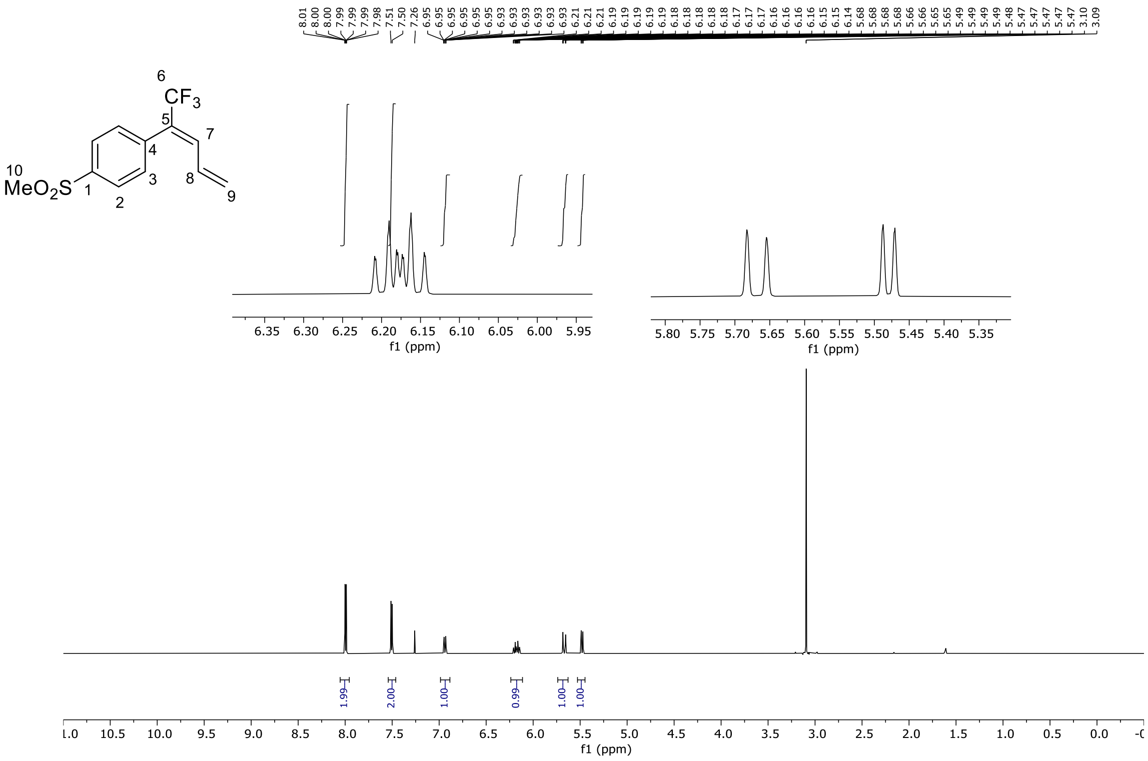

^{13}C NMR (151 MHz, $CDCl_3$)

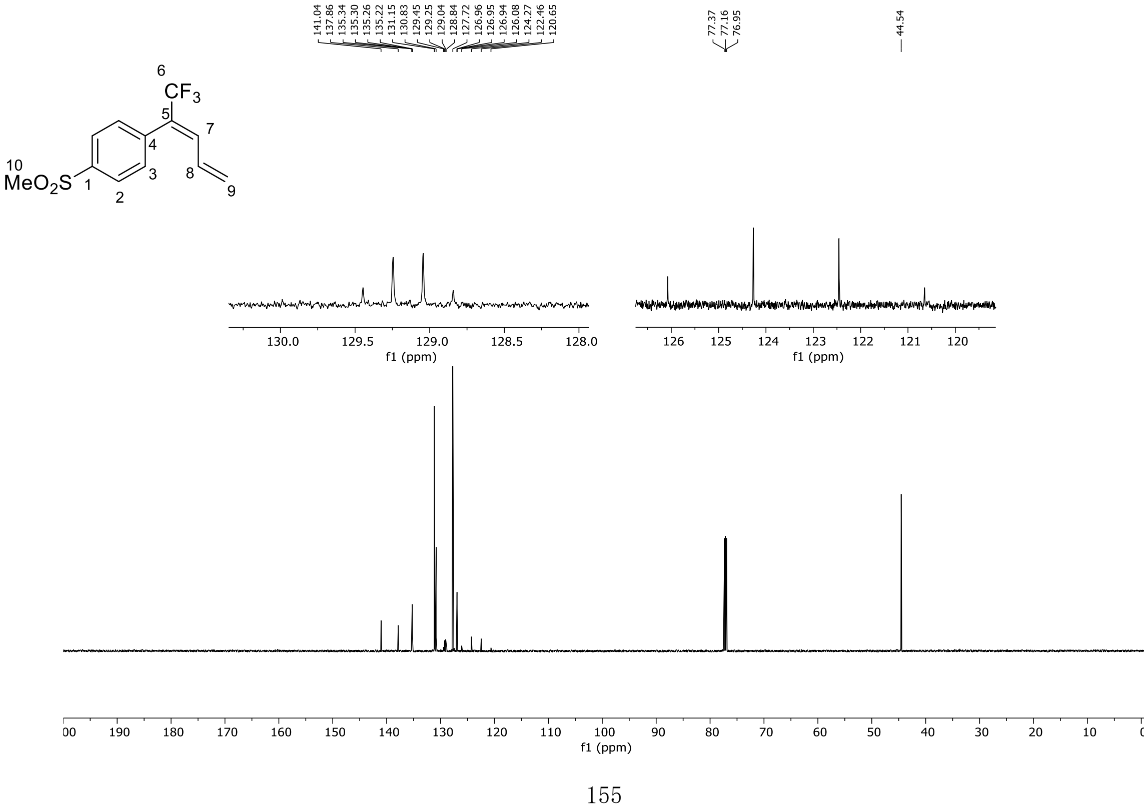

^{19}**F NMR** (564 MHz, $CDCl_3$)

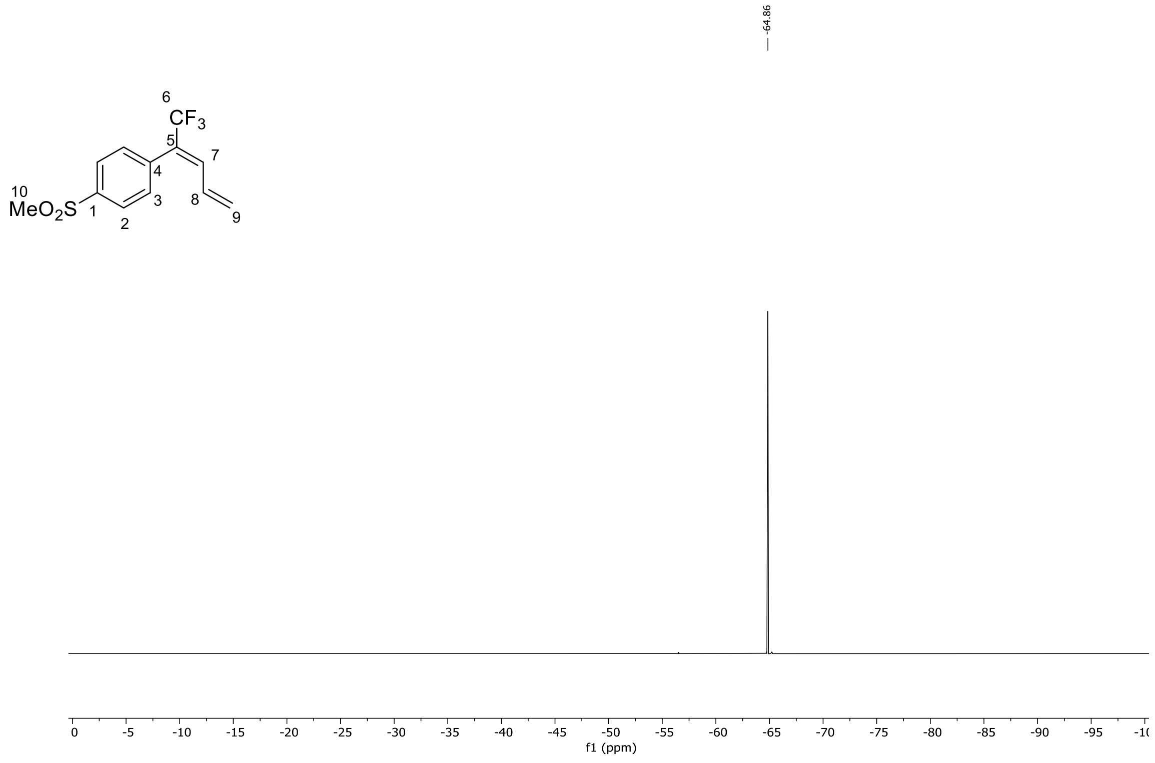

^{19}**F{^{1}H} NMR** (564 MHz, $CDCl_3$)

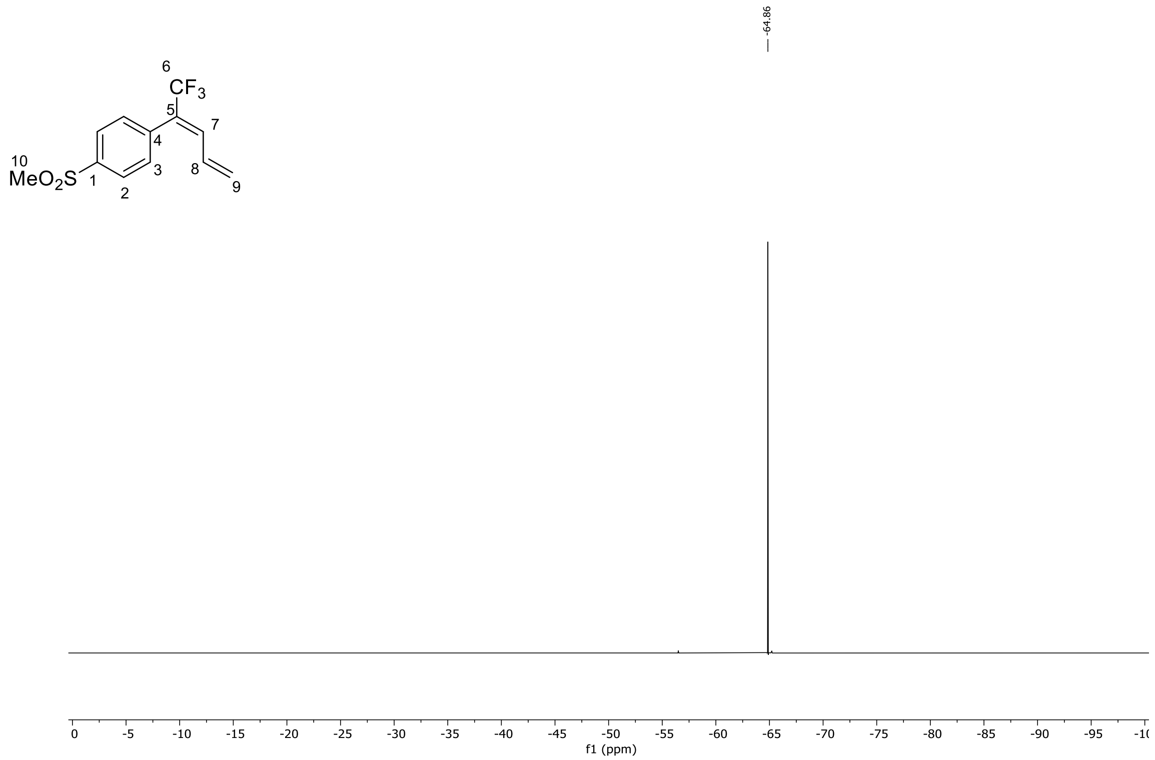

3-(1,1,1-Trifluoro-2-hydroxypent-4-en-2-yl)phenyl 4-methylbenzenesulfonate (1o-1)

^{1}H NMR (500 MHz, $CDCl_3$)

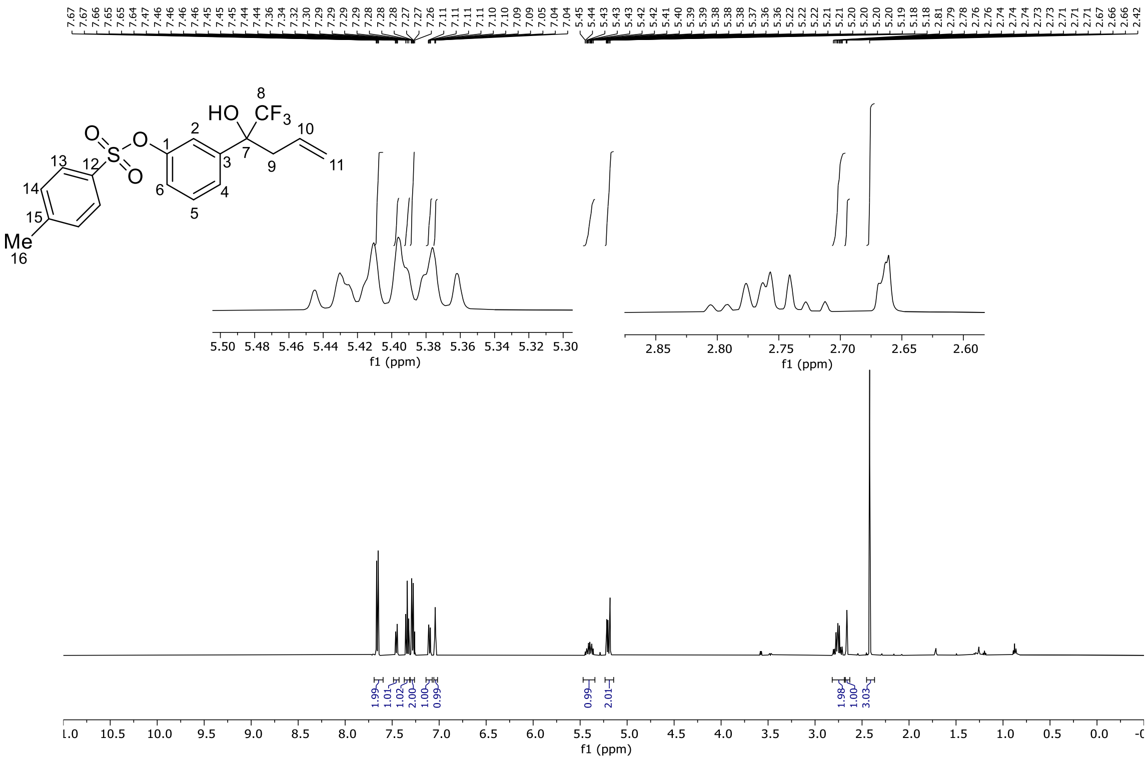

^{13}C NMR (126 MHz, $CDCl_3$)

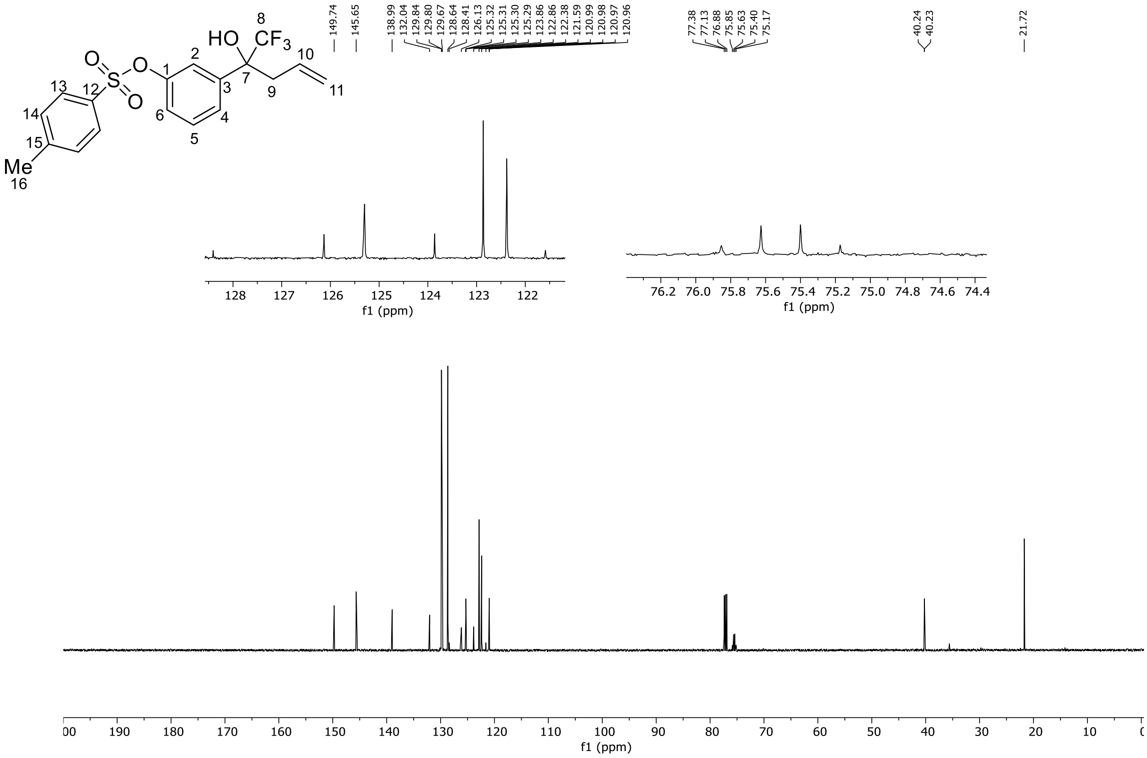

^{19}**F NMR** (470 MHz, $CDCl_3$)

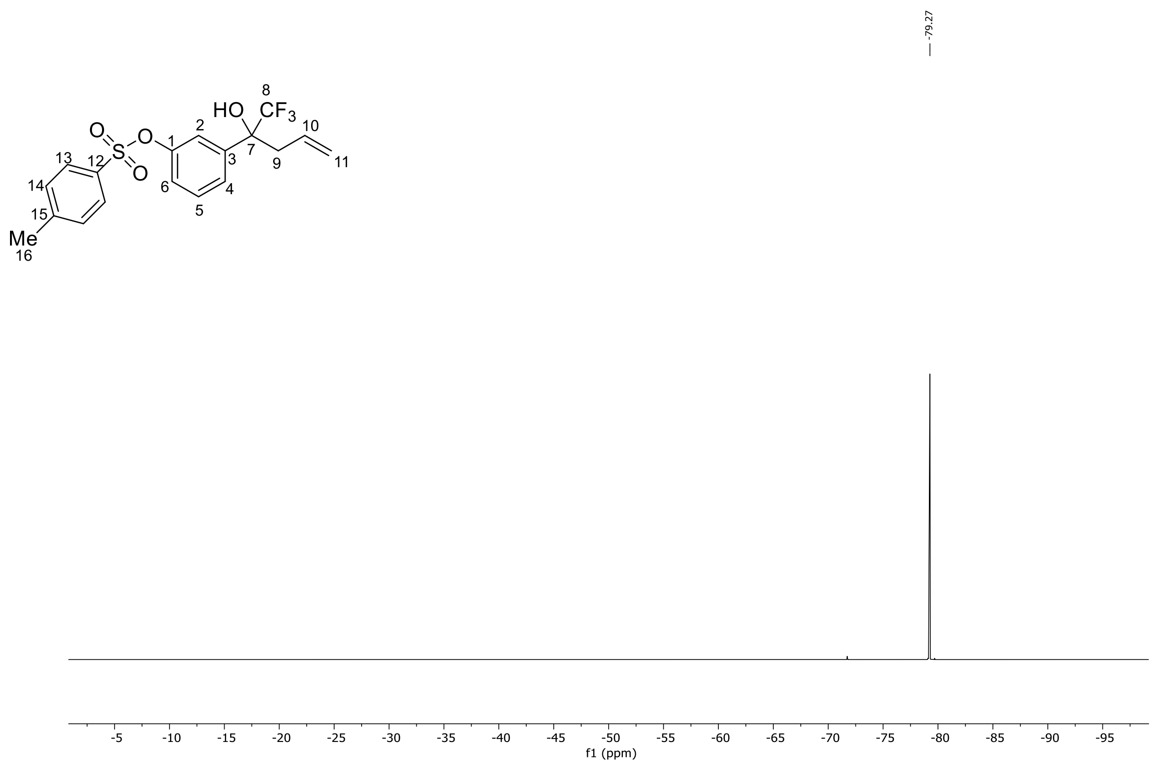

^{19}**F{^{1}H} NMR** (470 MHz, $CDCl_3$)

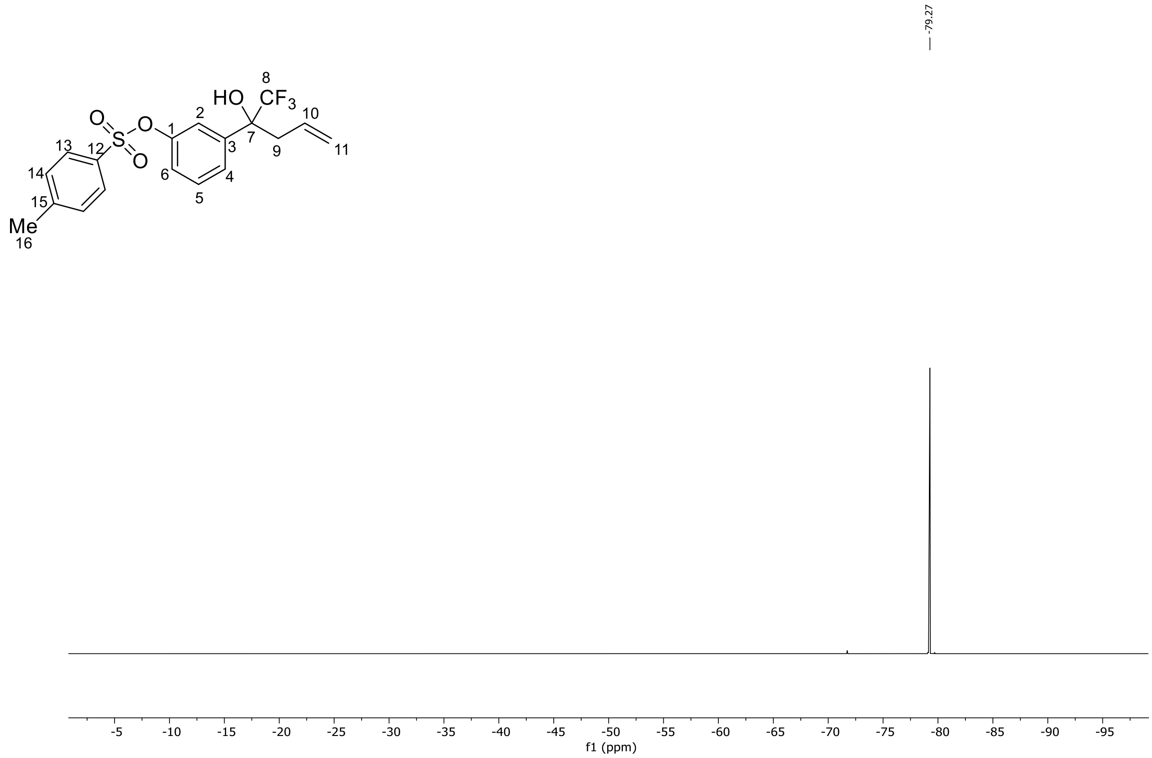

3-(1,1,1-Trifluoropenta-2,4-dien-2-yl)phenyl 4-methylbenzenesulfonate (1o)

^{1}H NMR (500 MHz, $CDCl_3$)

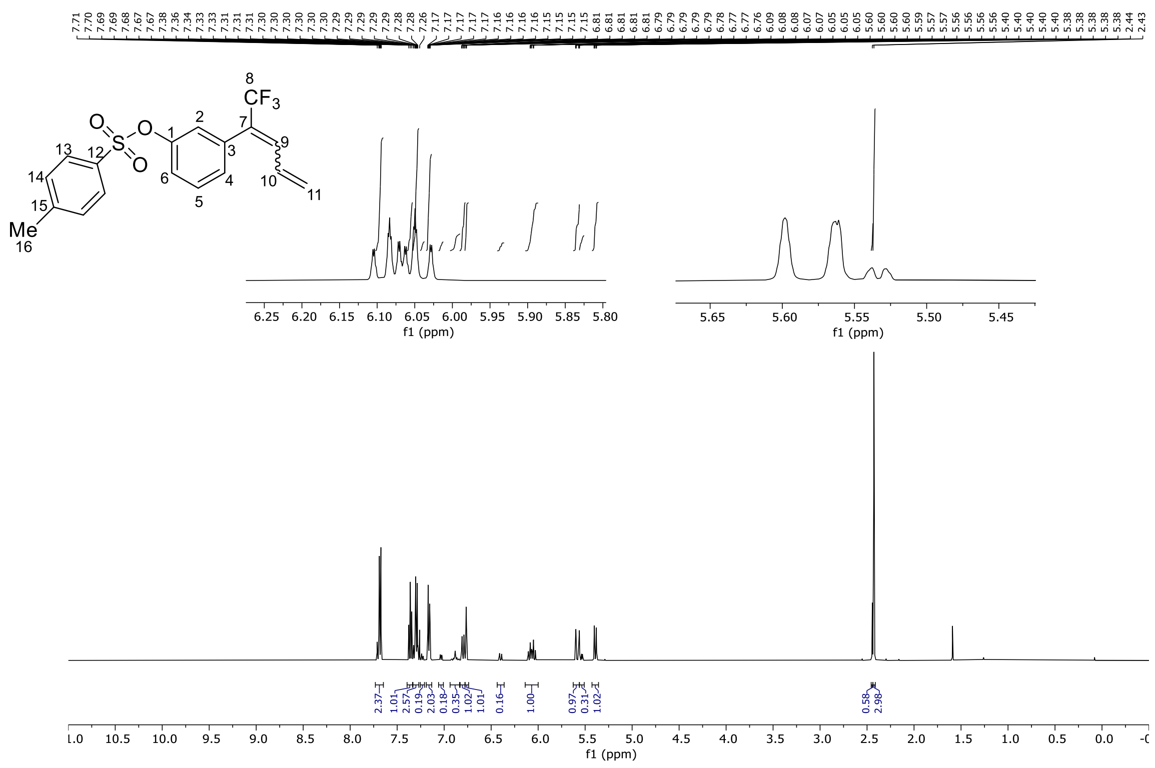

^{13}C NMR (126 MHz, $CDCl_3$)

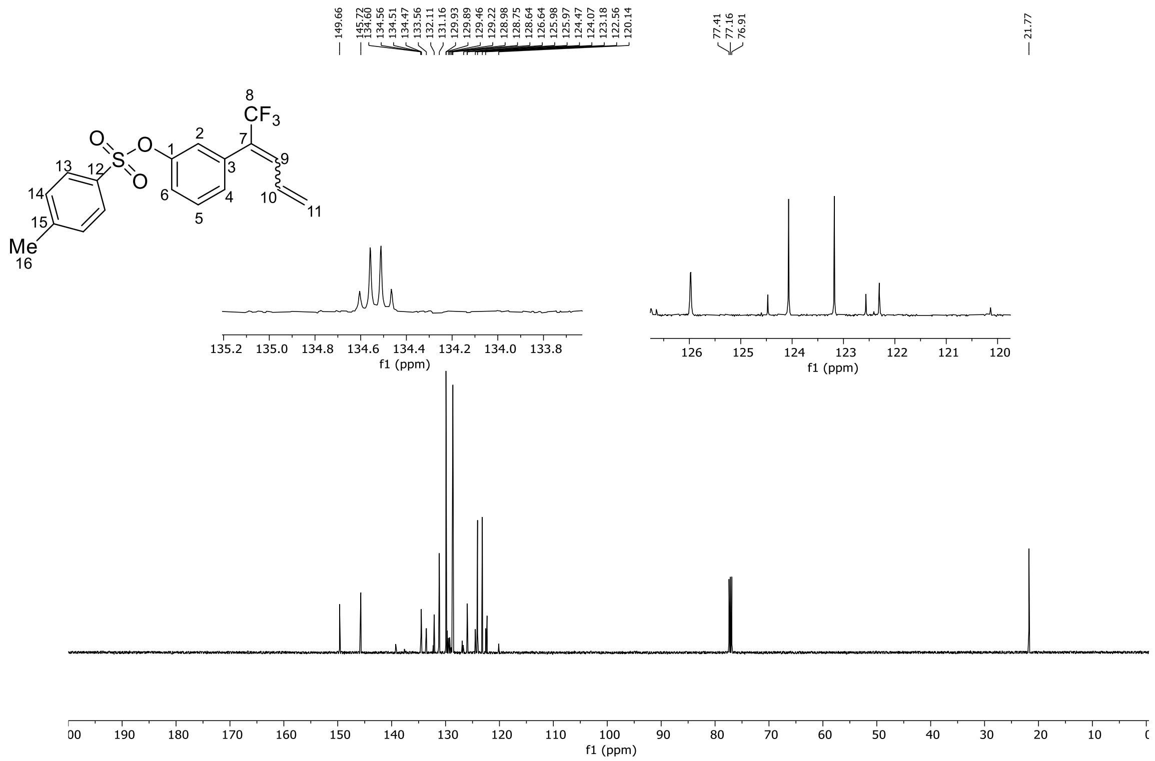

^{19}F NMR (470 MHz, $CDCl_3$)

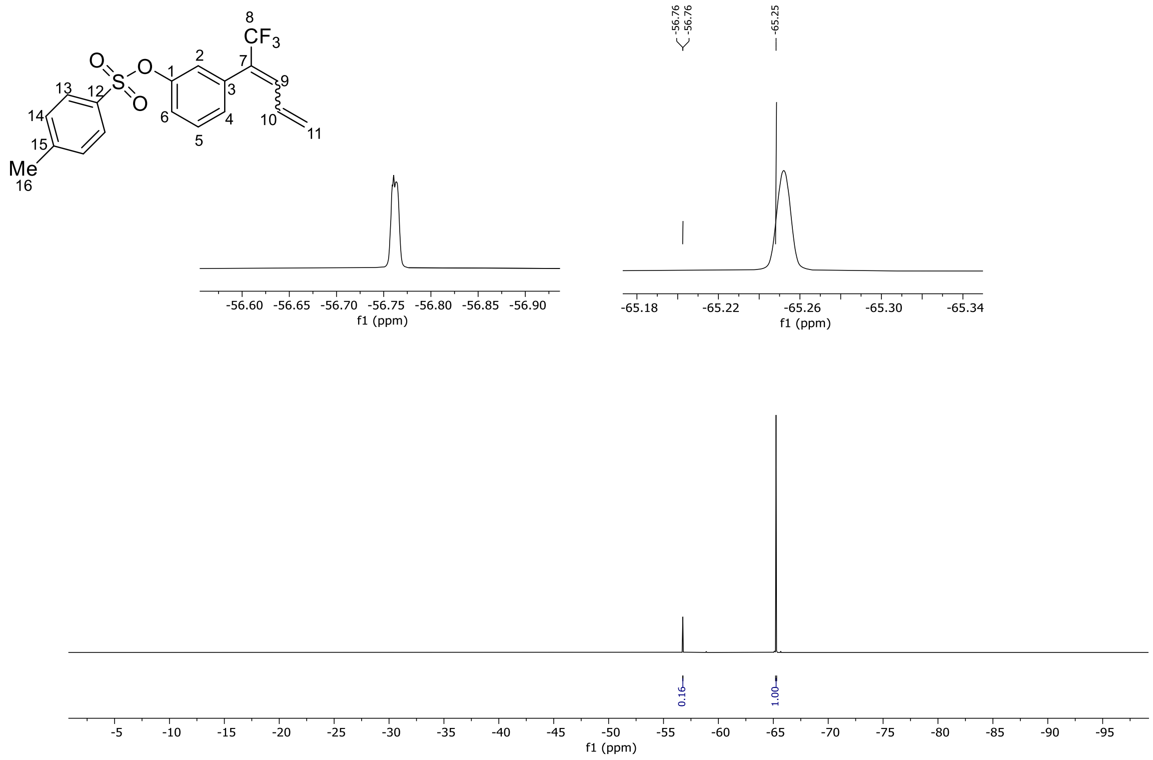

^{19}F{^{1}H} NMR (470 MHz, $CDCl_3$)

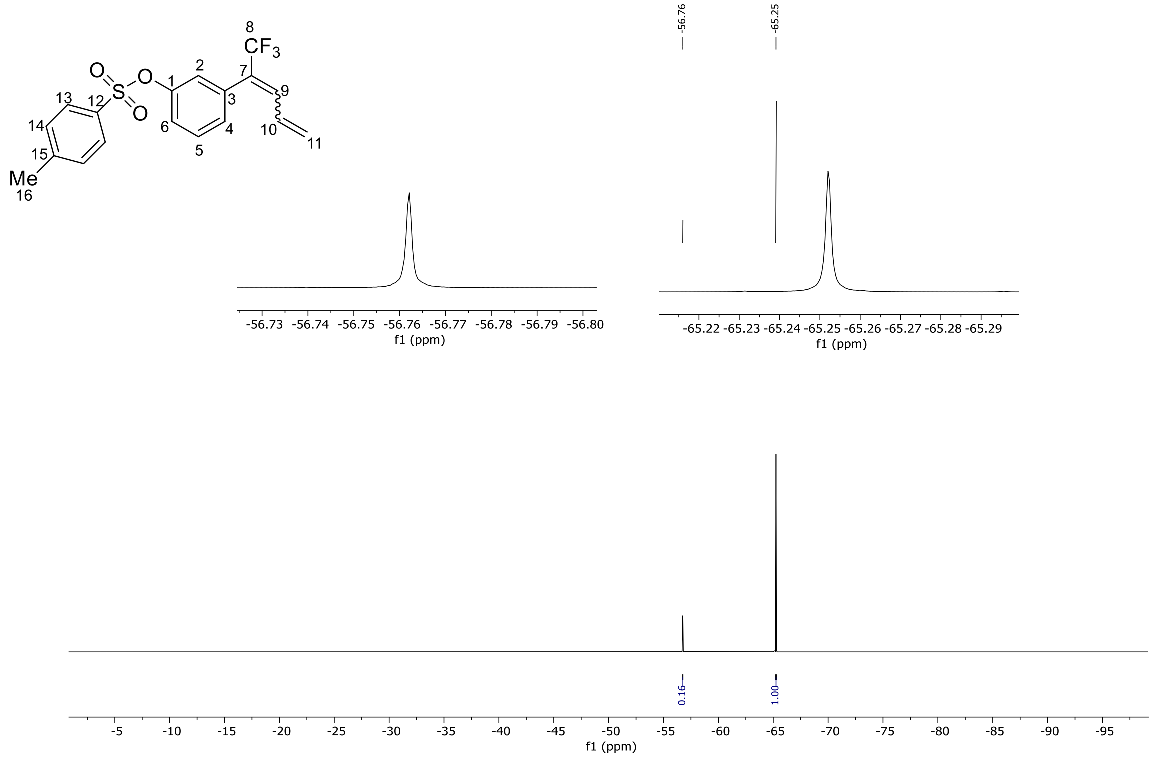

1,1,1-Trifluoro-2-(3-(trifluoromethoxy)phenyl)pent-4-en-2-ol (1p-1)

^{1}H NMR (500 MHz, $CDCl_3$)

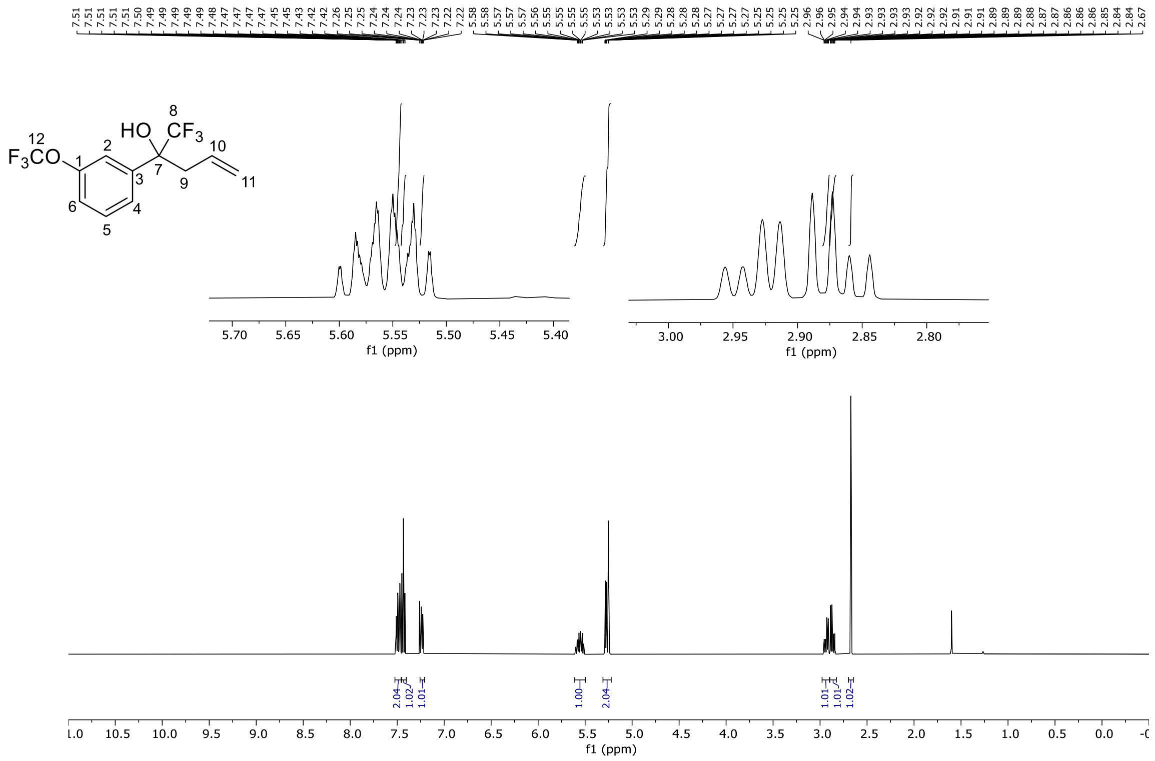

^{13}C NMR (126 MHz, $CDCl_3$)

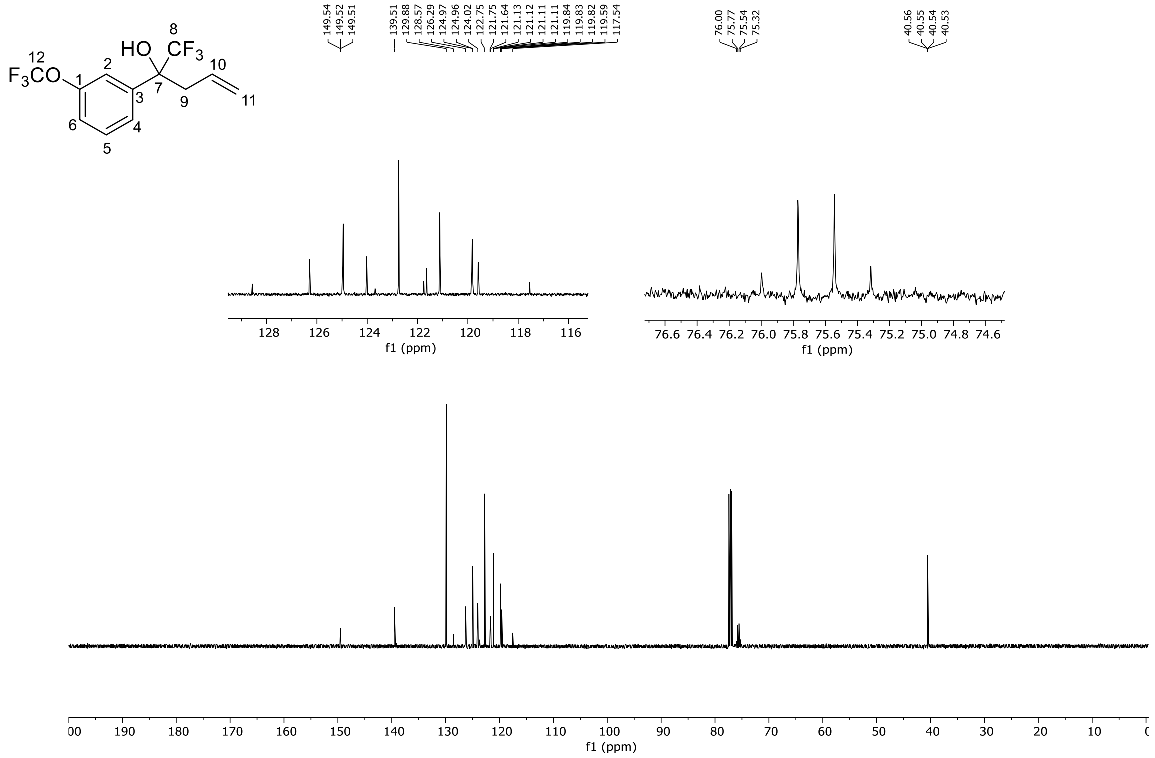

^{19}F NMR (470 MHz, $CDCl_3$)

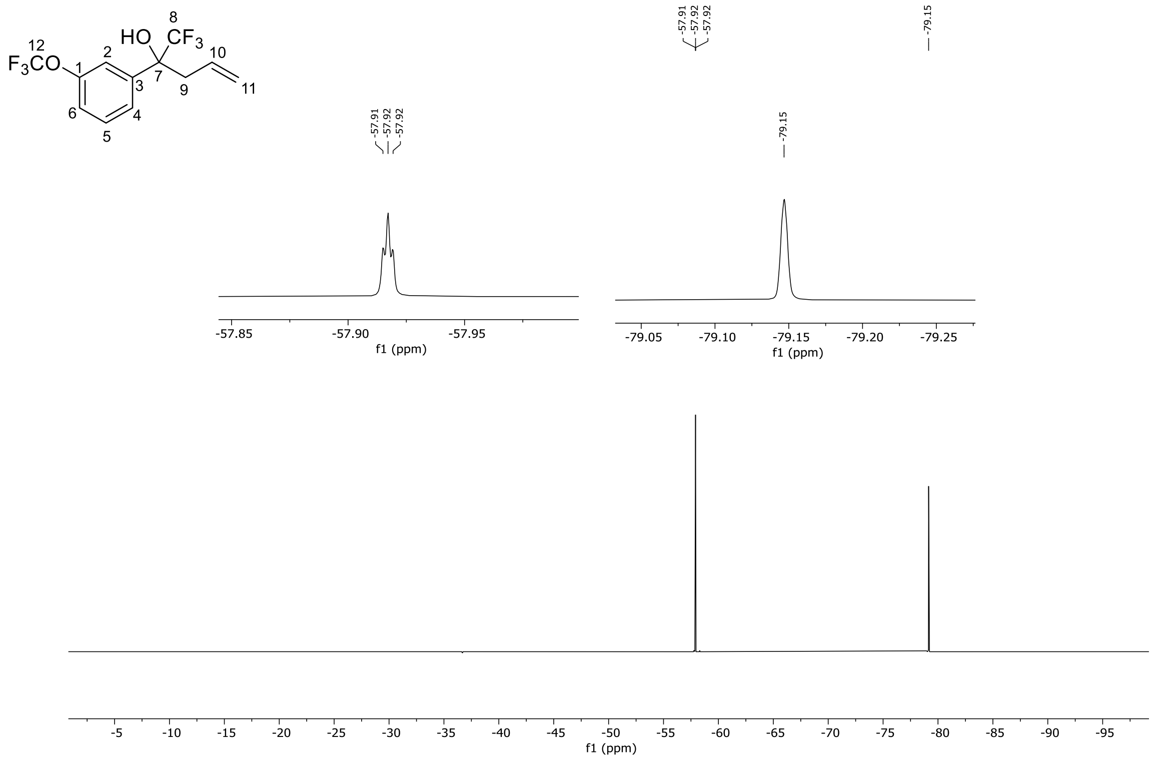

^{19}F{^{1}H} NMR (470 MHz, $CDCl_3$)

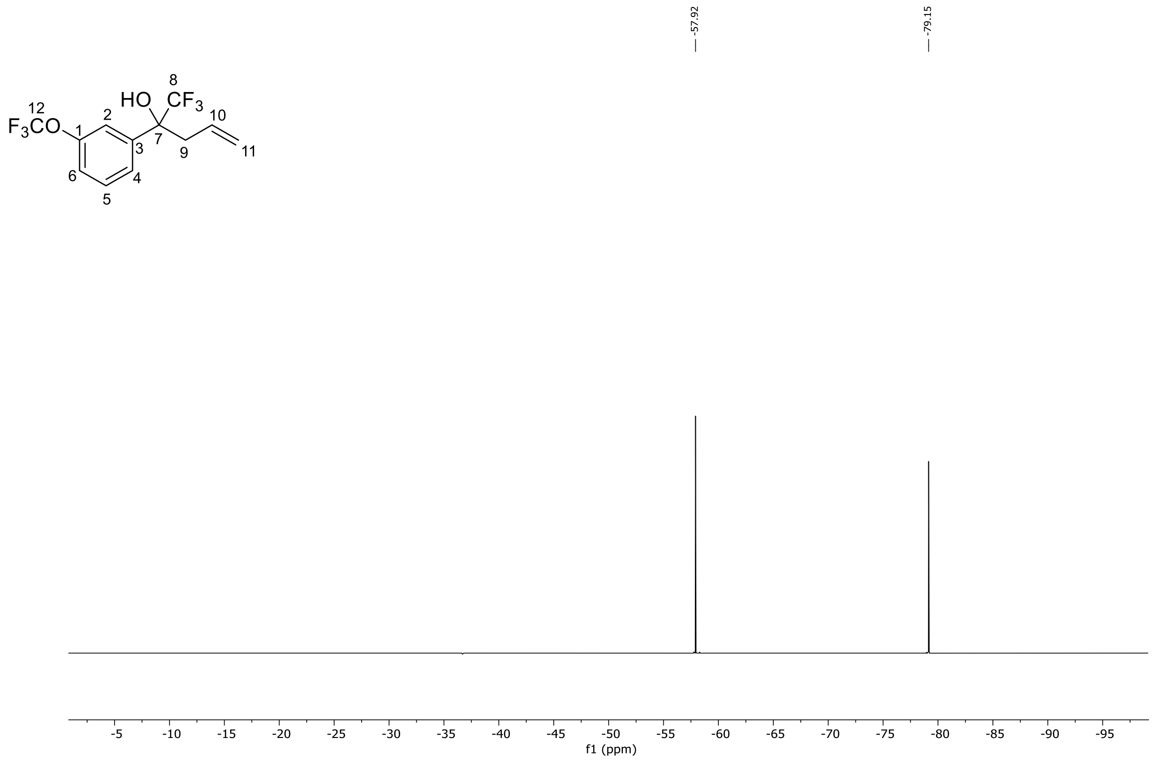

1-(Trifluoromethoxy)-3-(1,1,1-trifluoropenta-2,4-dien-2-yl)benzene (1p)

^{1}H NMR (500 MHz, $CDCl_3$)

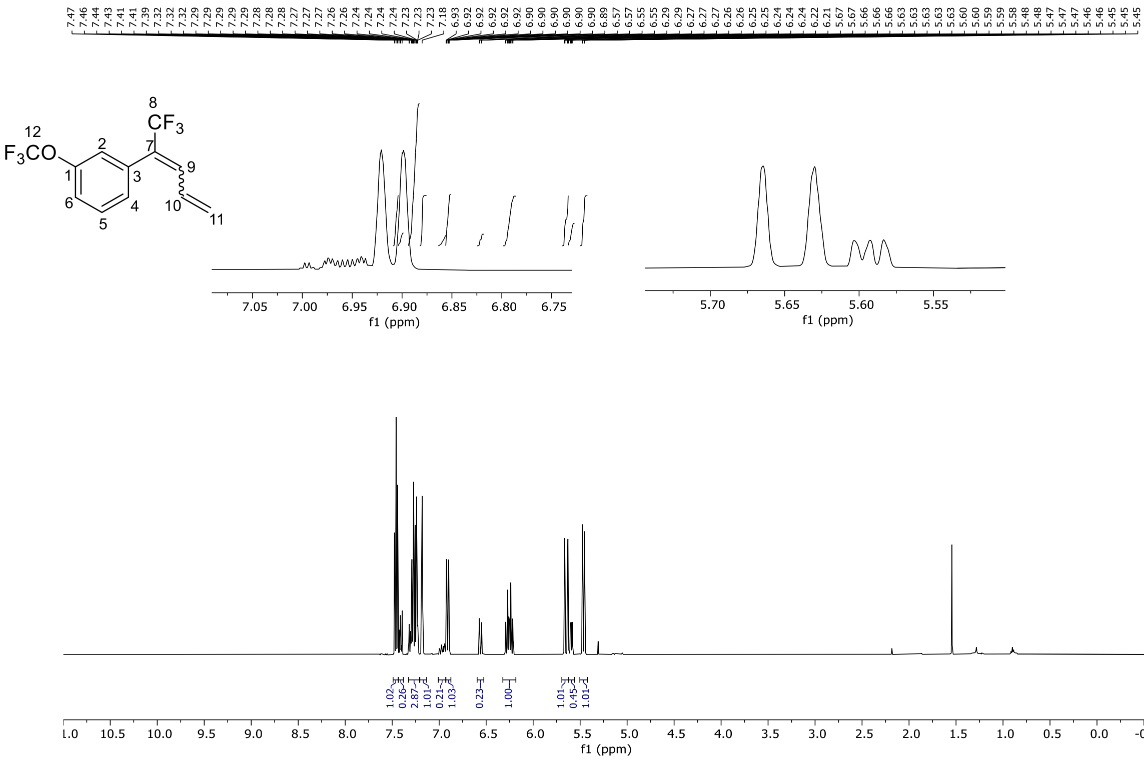

^{13}C NMR (126 MHz, $CDCl_3$)

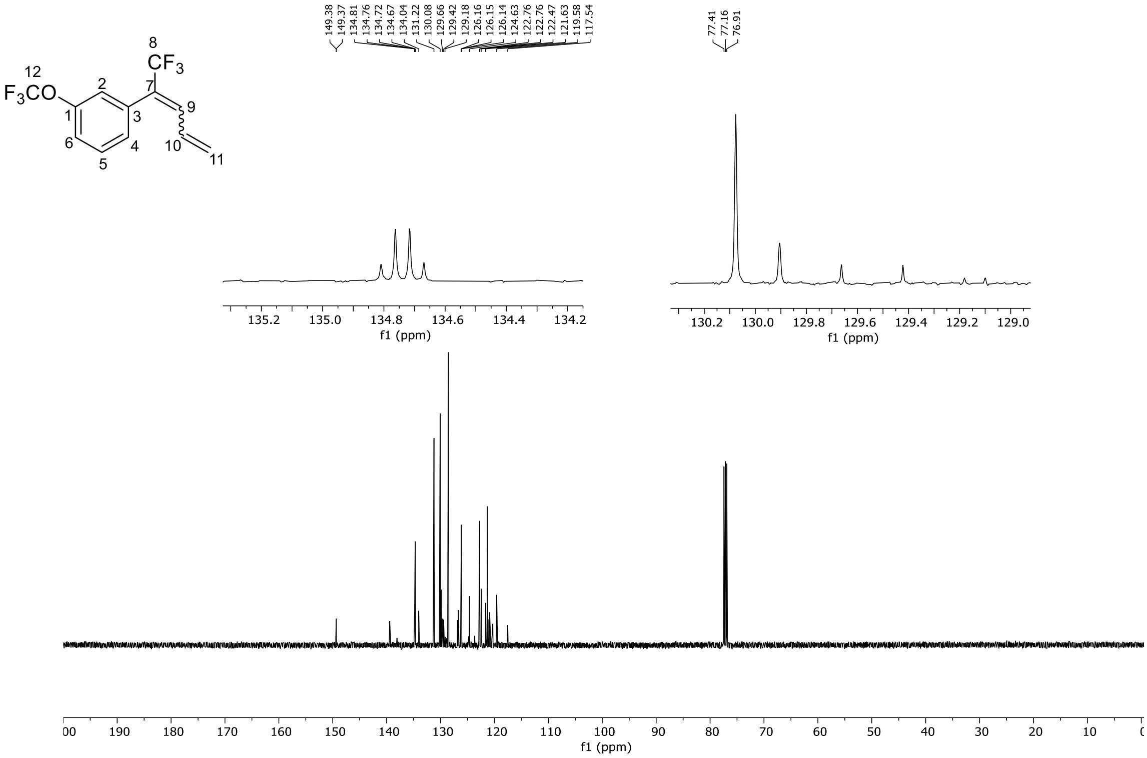

^{19}**F NMR** (470 MHz, $CDCl_3$)

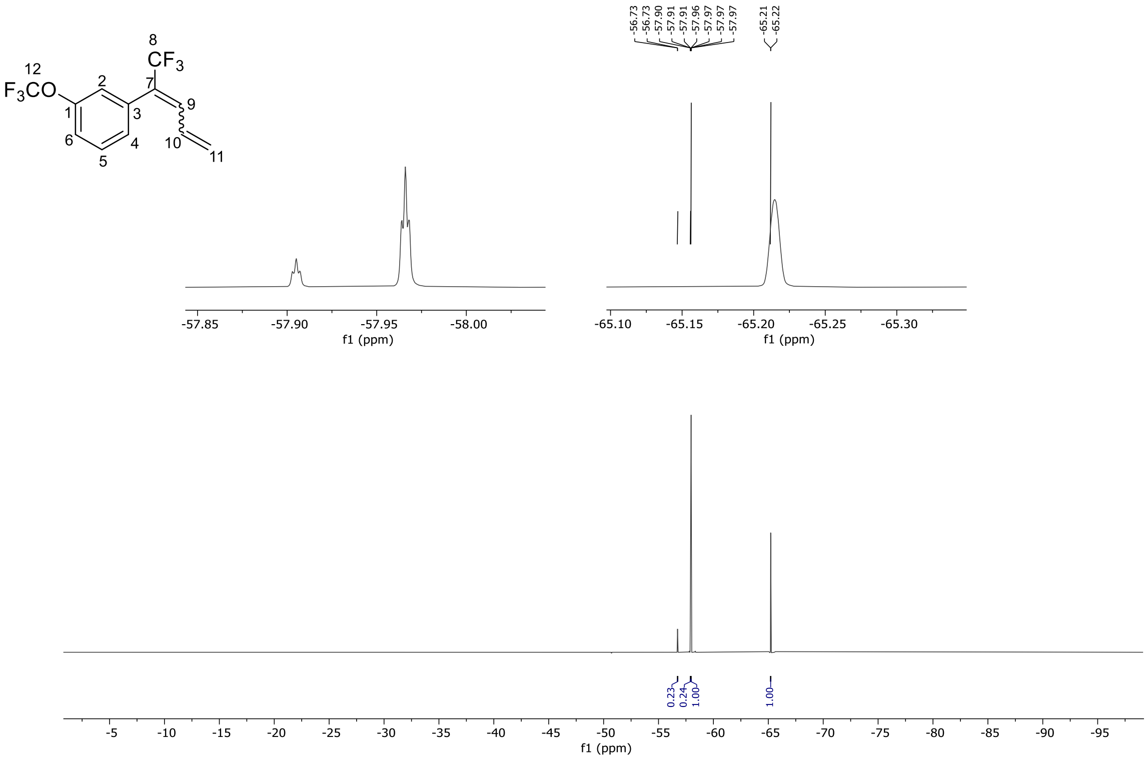

^{19}**F{^{1}H} NMR** (470 MHz, $CDCl_3$)

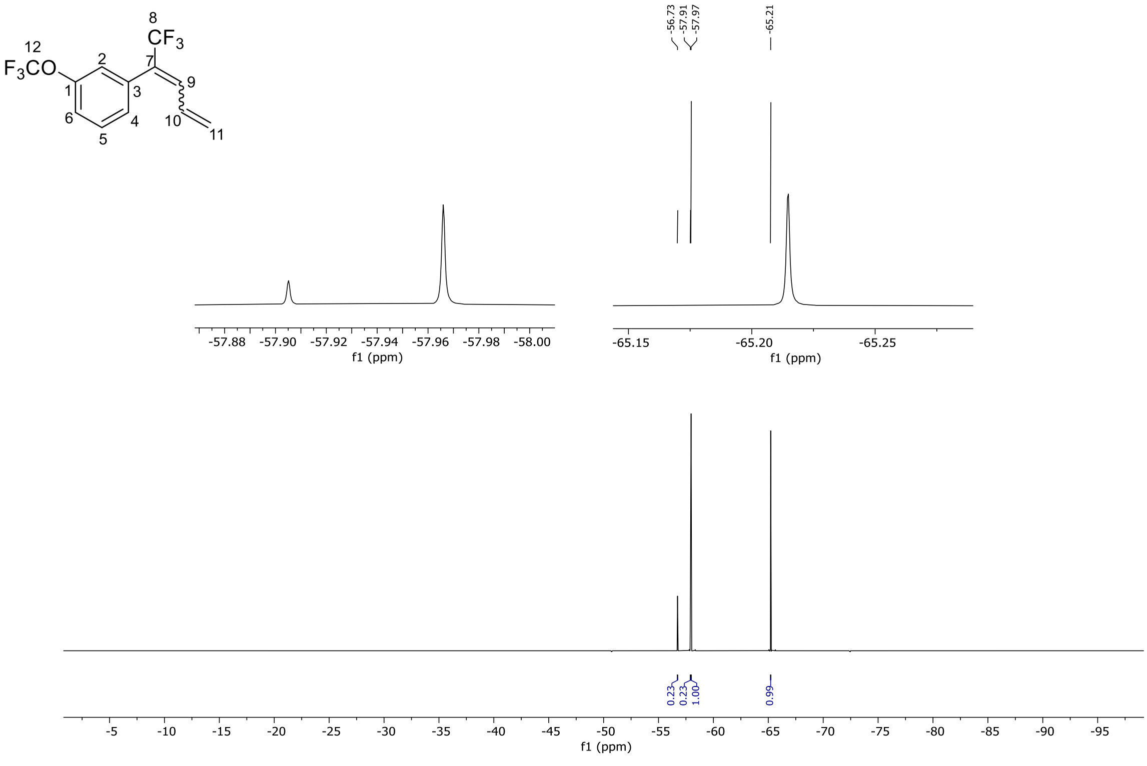

4-(1,1,1-Trifluoro-2-hydroxypent-4-en-2-yl)phenyl trifluoromethanesulfonate (1q-1)

^{1}H NMR (500 MHz, $CDCl_3$)

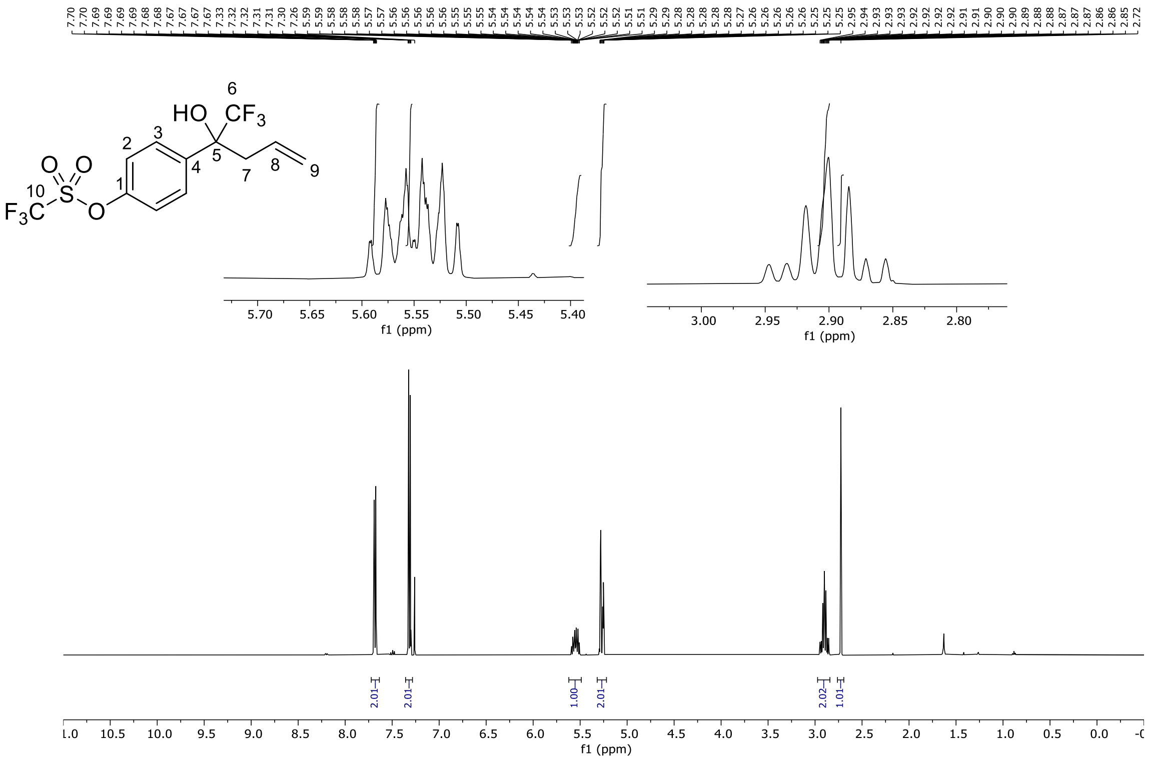

^{13}C NMR (126 MHz, $CDCl_3$)

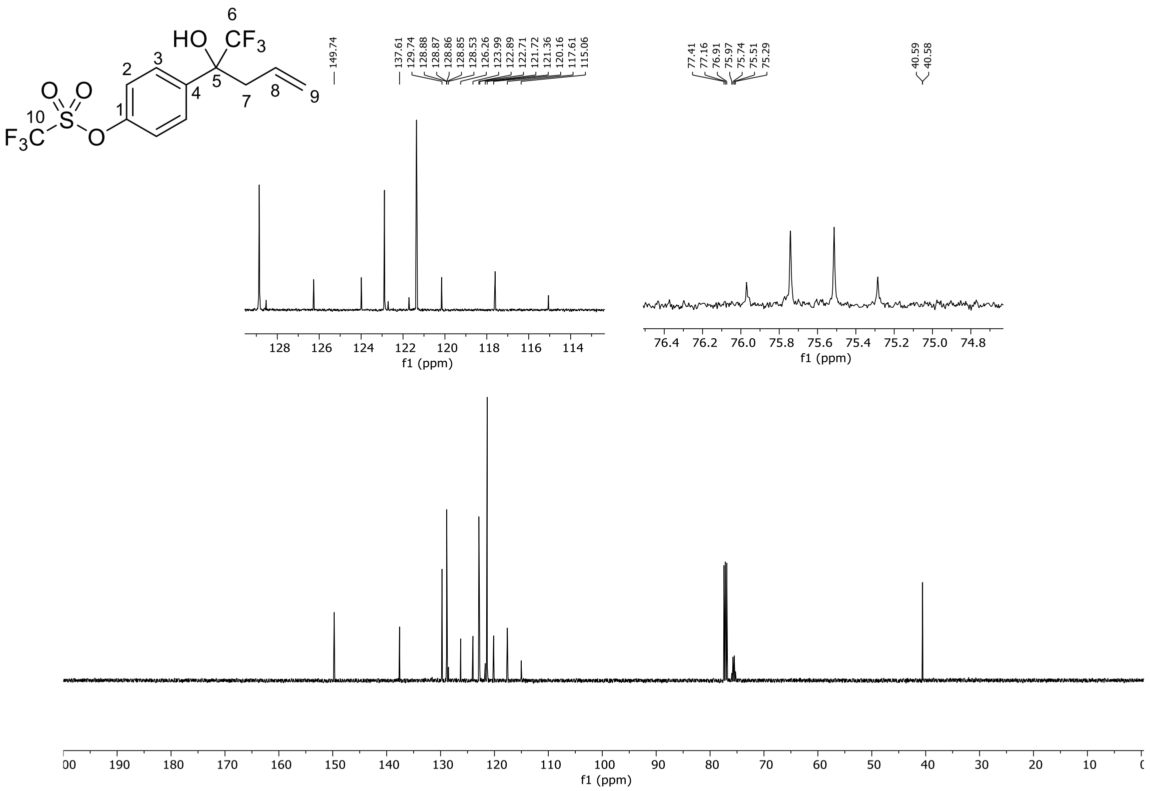

^{19}F **NMR** (470 MHz, CDCl$_3$)

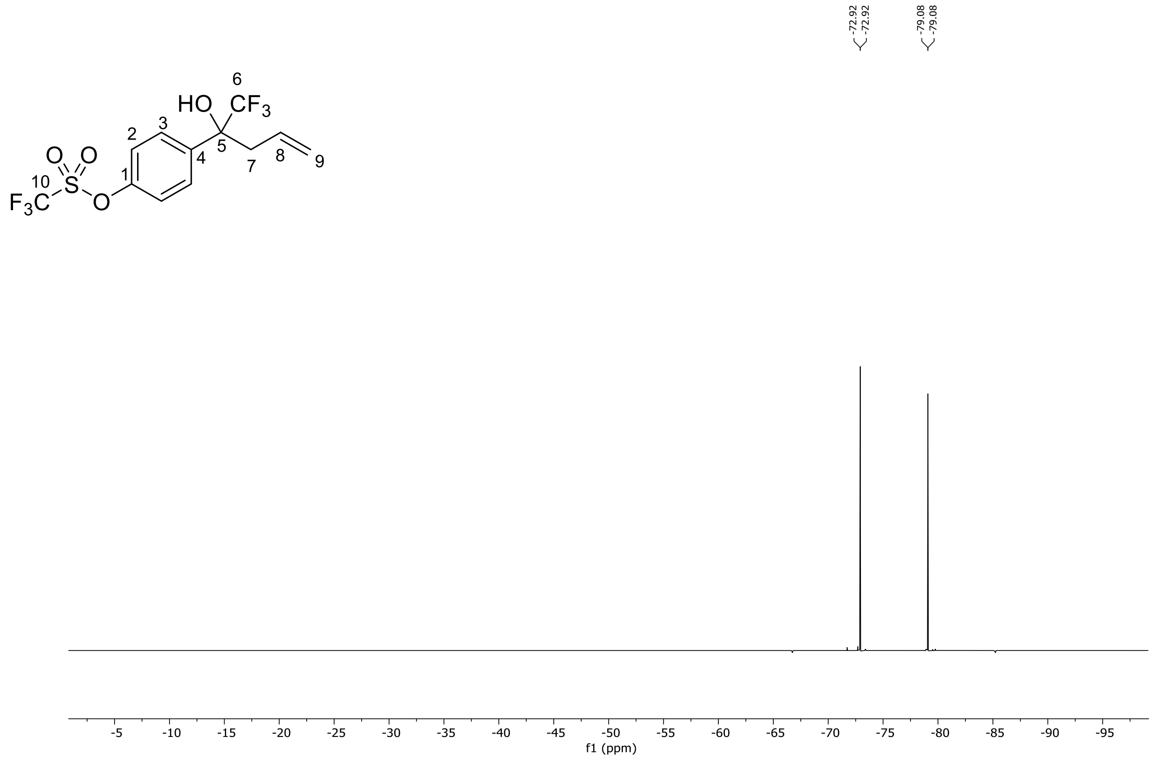

^{19}F{^{1}H} **NMR** (470 MHz, CDCl$_3$)

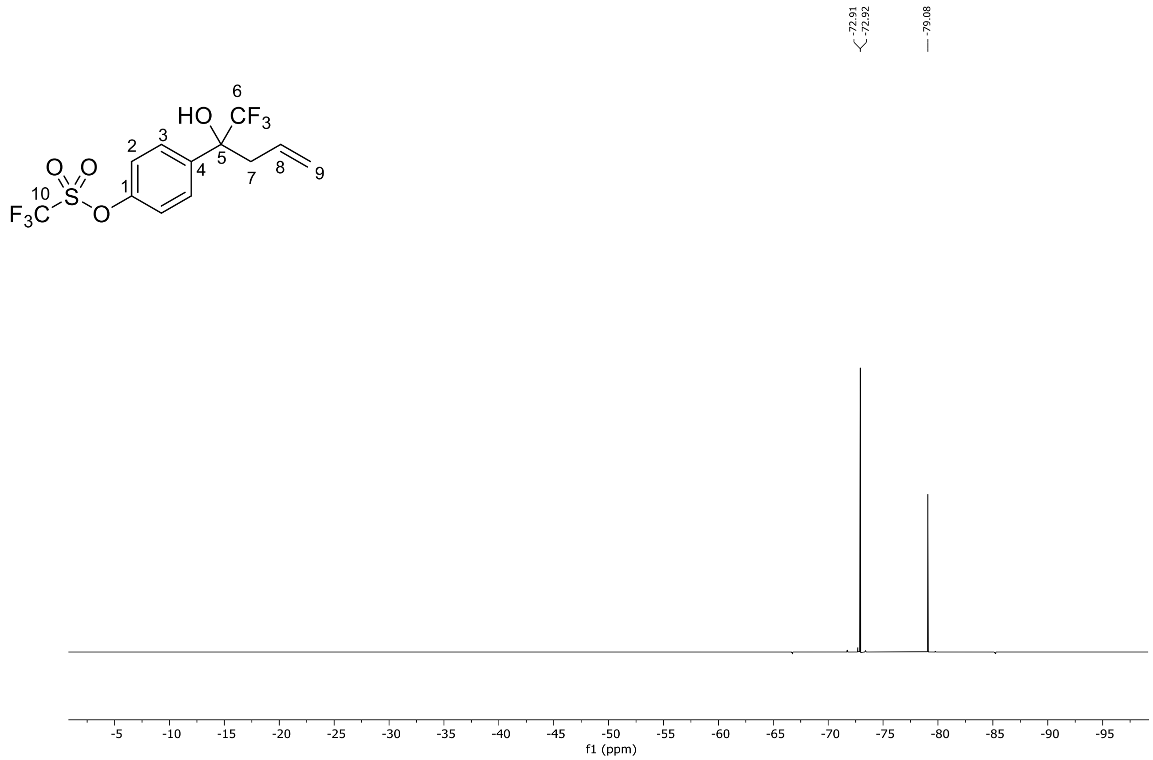

(*E*)-4-(1,1,1-Trifluoropenta-2,4-dien-2-yl)phenyl trifluoromethanesulfonate (1q)

^{1}H NMR (500 MHz, $CDCl_3$)

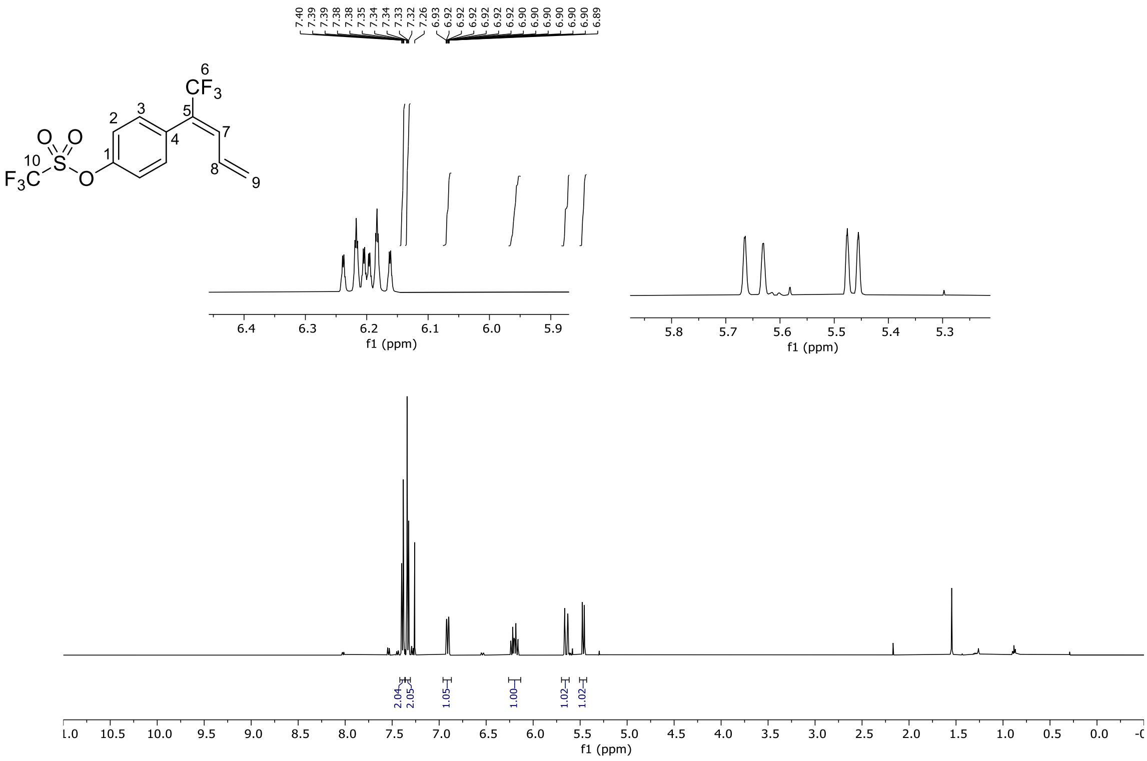

^{13}C NMR (126 MHz, $CDCl_3$)

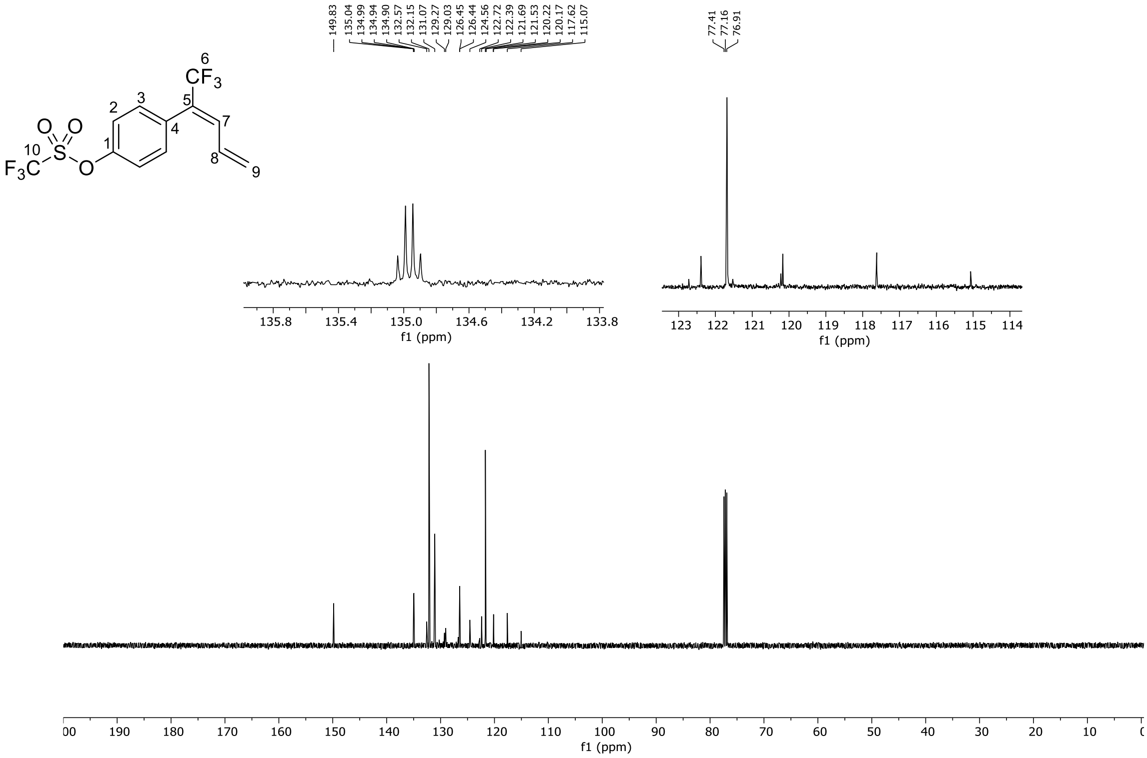

^{19}F NMR (470 MHz, $CDCl_3$)

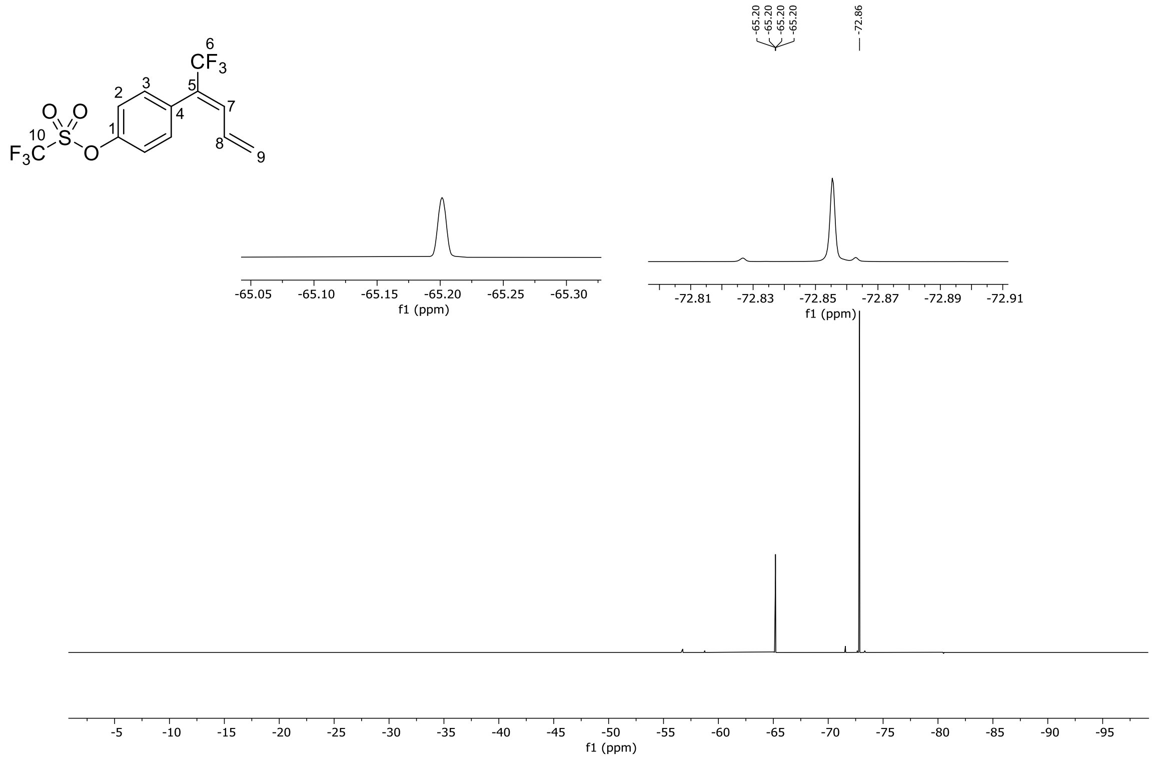

^{19}F{^{1}H} NMR (470 MHz, $CDCl_3$)

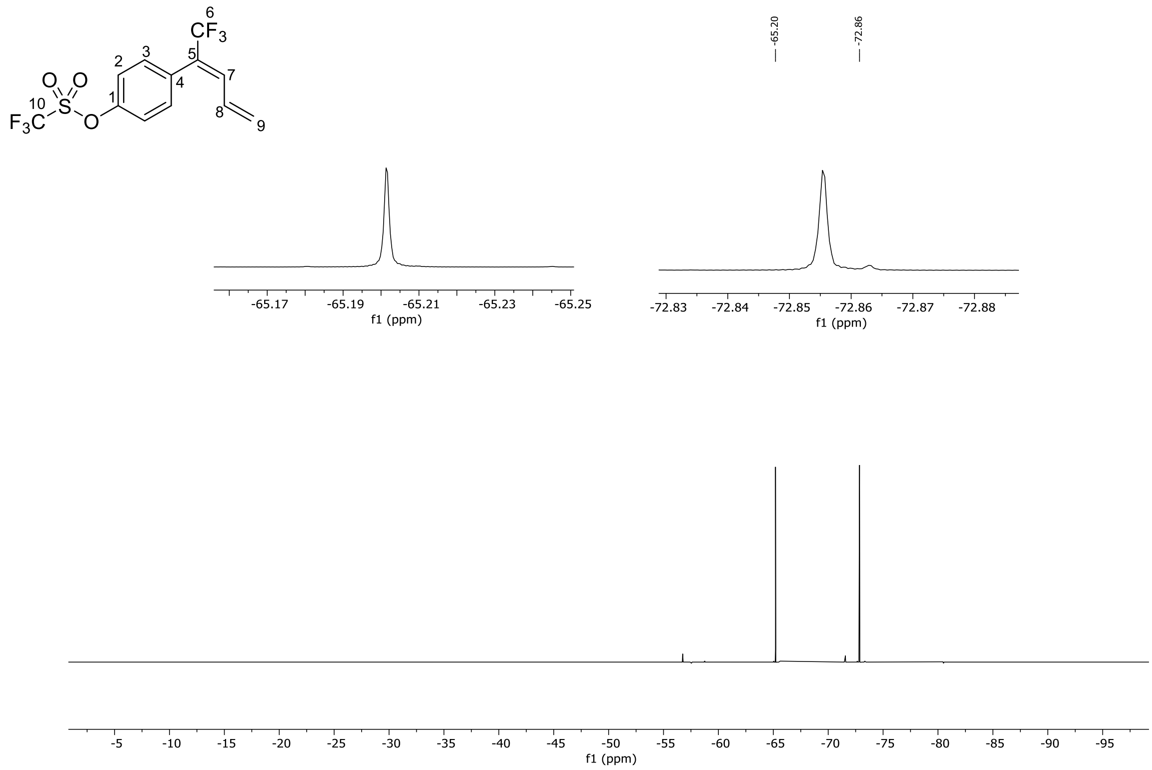

4-(1,1,1-Trifluoro-2-hydroxy-4-methylpent-4-en-2-yl)phenyl 4-methylbenzenesulfonate (1r-1)

^{1}H NMR (500 MHz, $CDCl_3$)

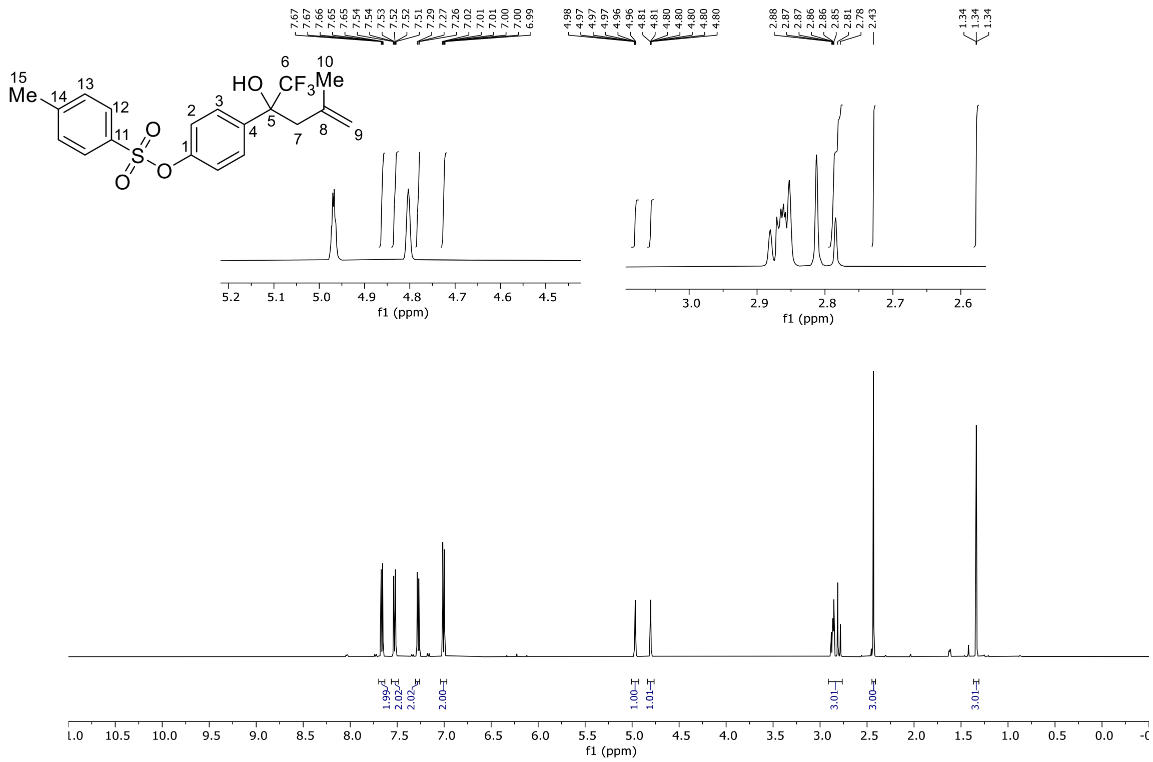

^{13}C NMR (126 MHz, $CDCl_3$)

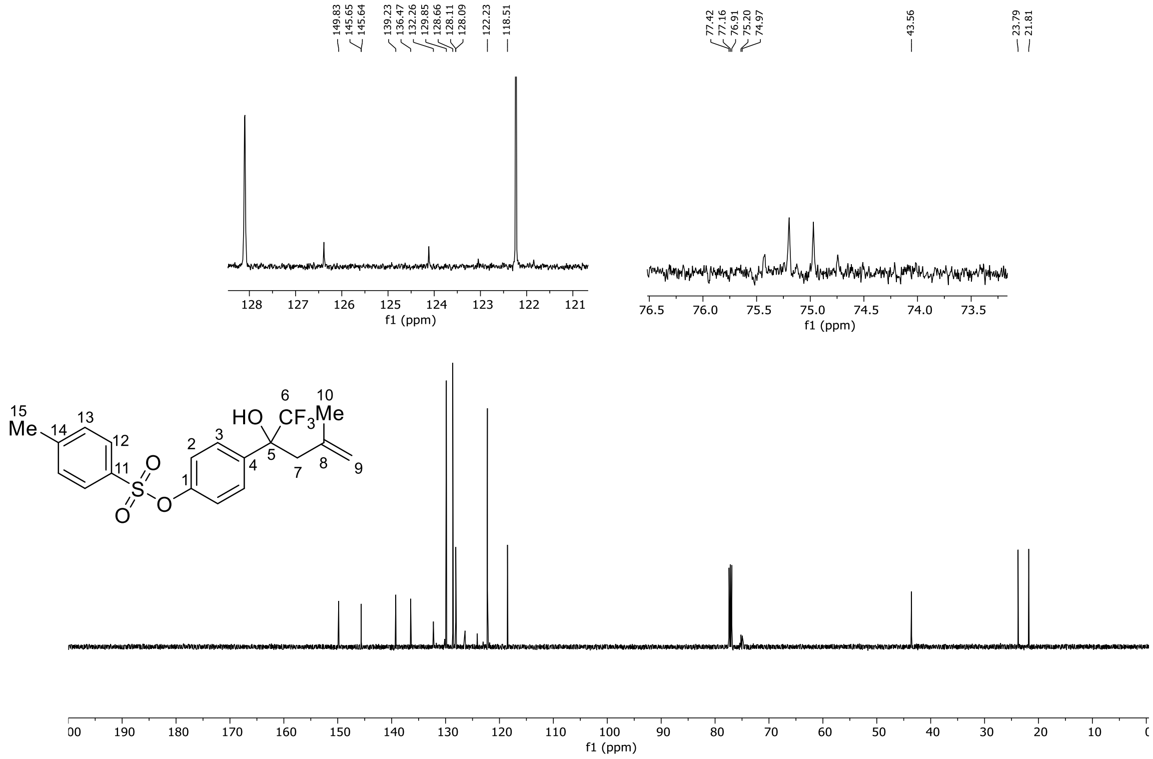

^{19}**F NMR** (470 MHz, $CDCl_3$)

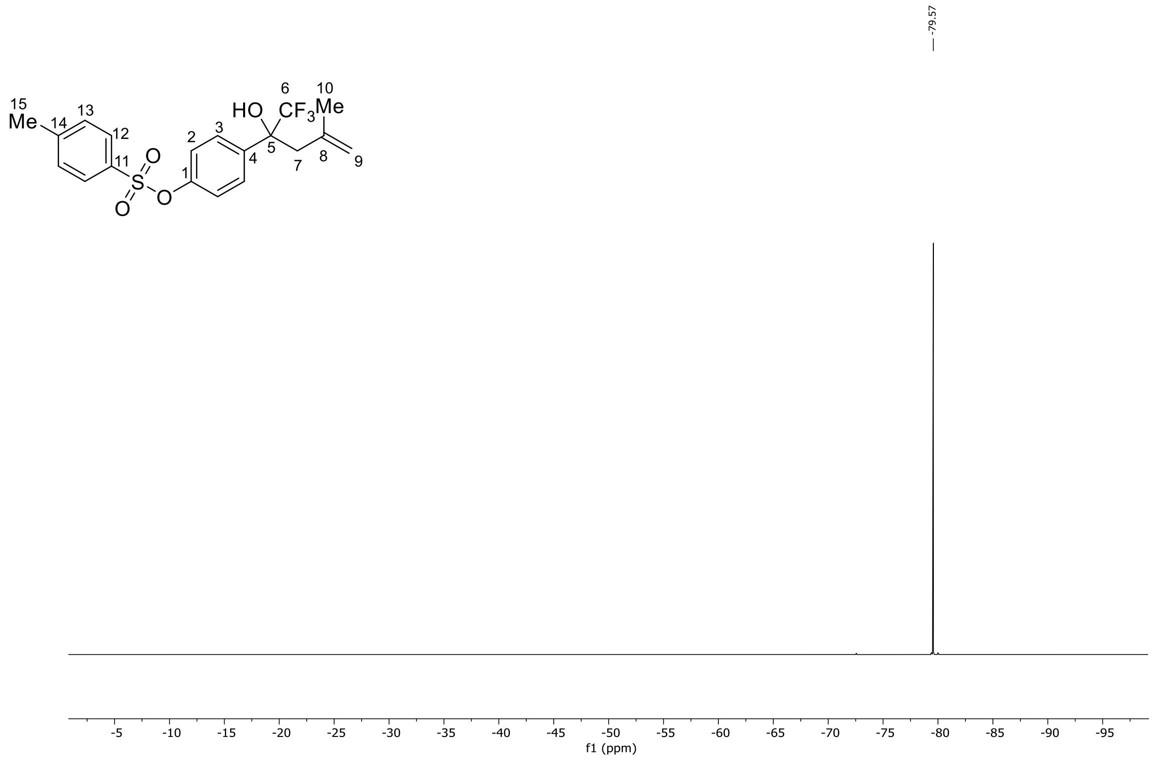

^{19}**F{^{1}H} NMR** (470 MHz, $CDCl_3$)

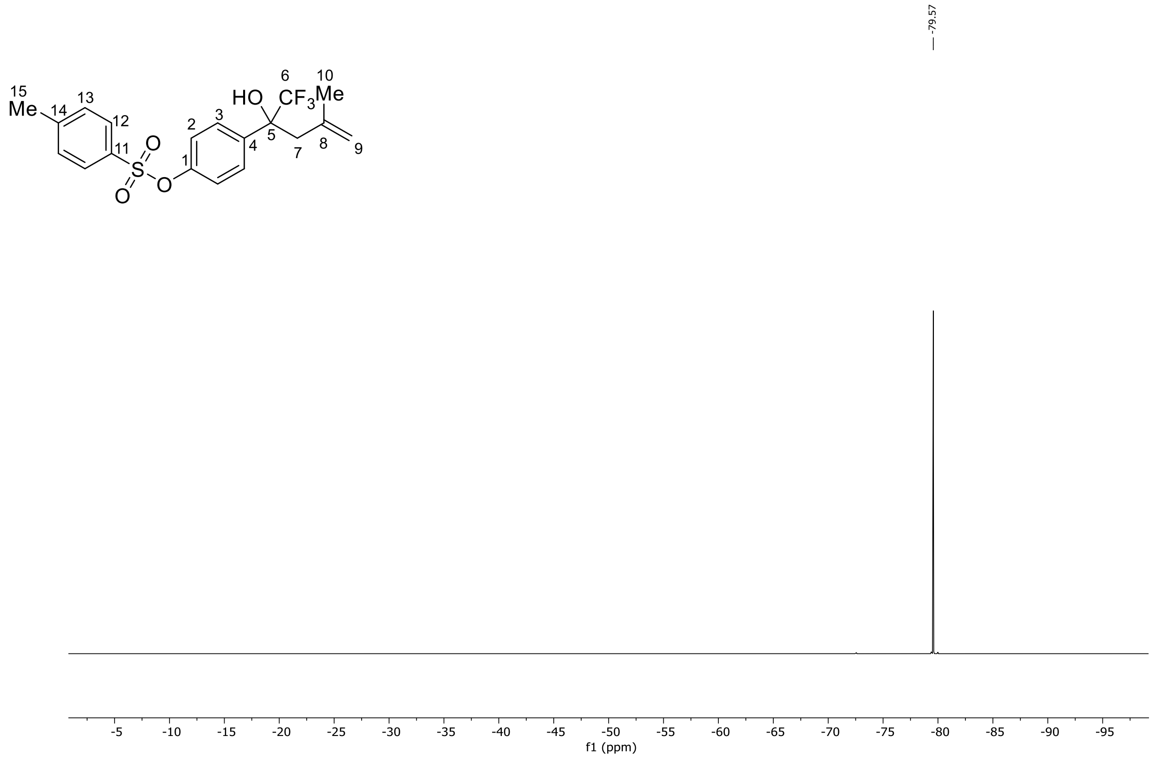

(E)-4-(1,1,1-Trifluoro-4-methylpenta-2,4-dien-2-yl)phenyl 4-methylbenzenesulfonate (1r)

^{1}H NMR (500 MHz, $CDCl_3$)

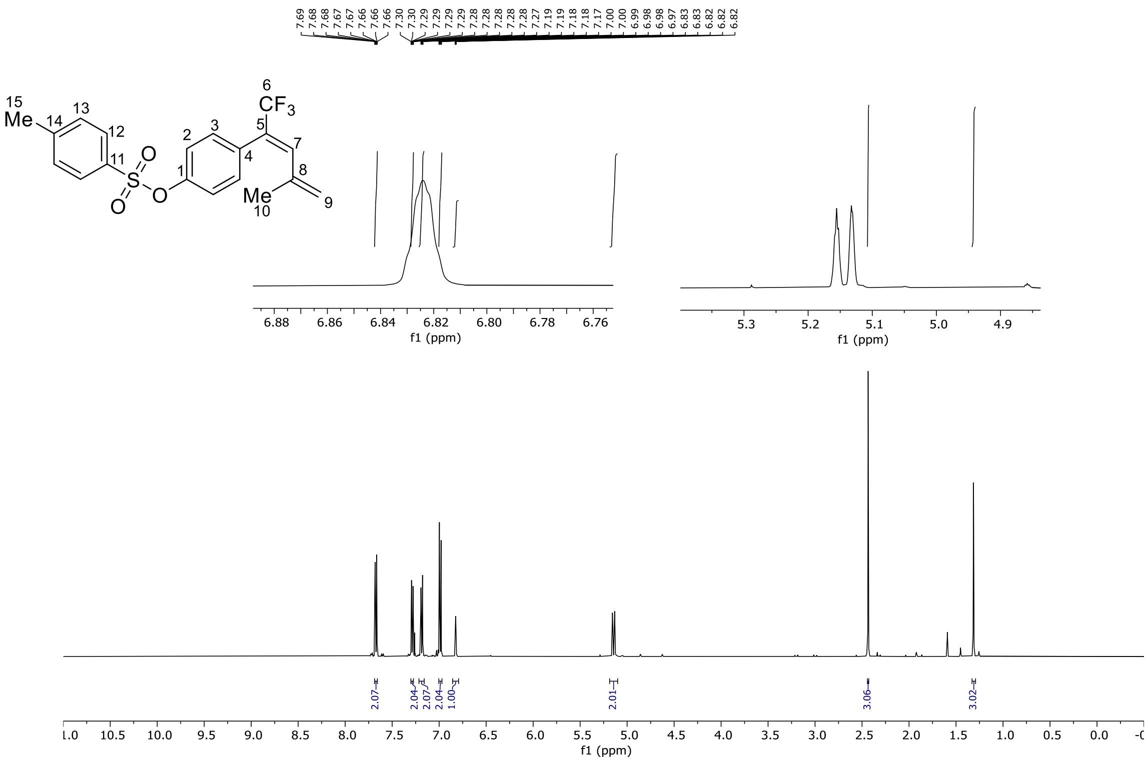

^{13}C NMR (126 MHz, $CDCl_3$)

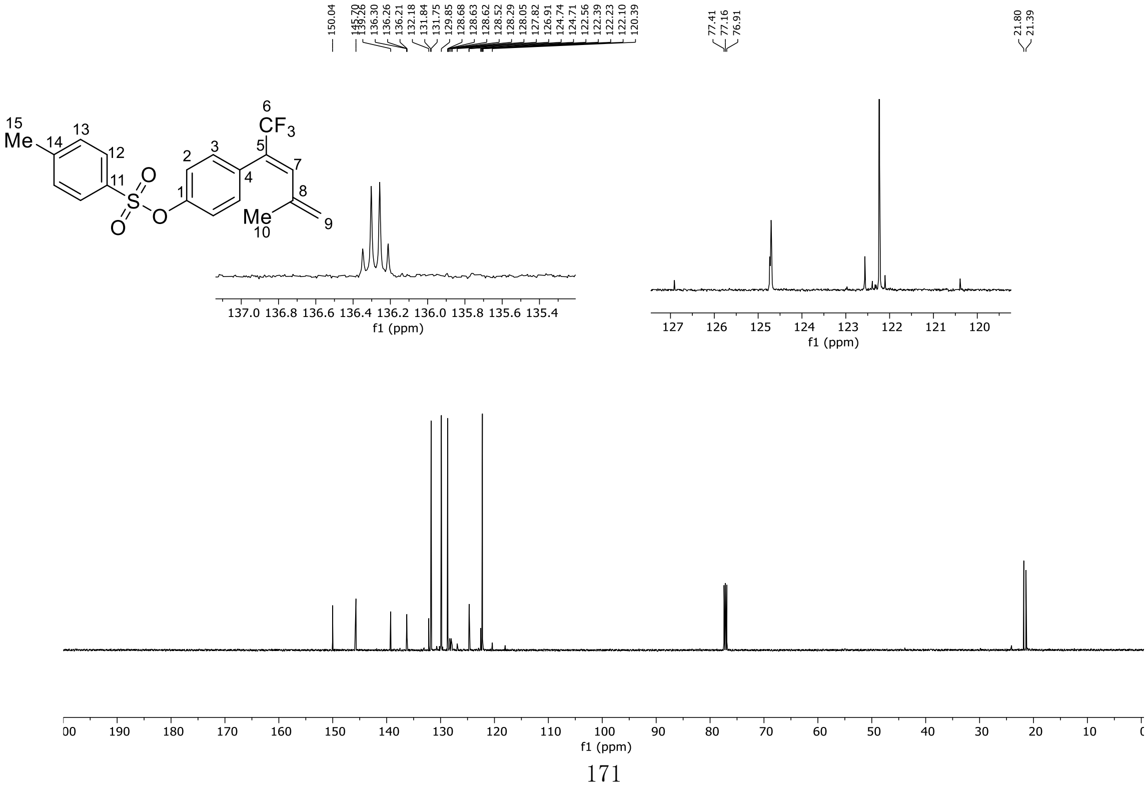

^{19}F NMR (470 MHz, $CDCl_3$)

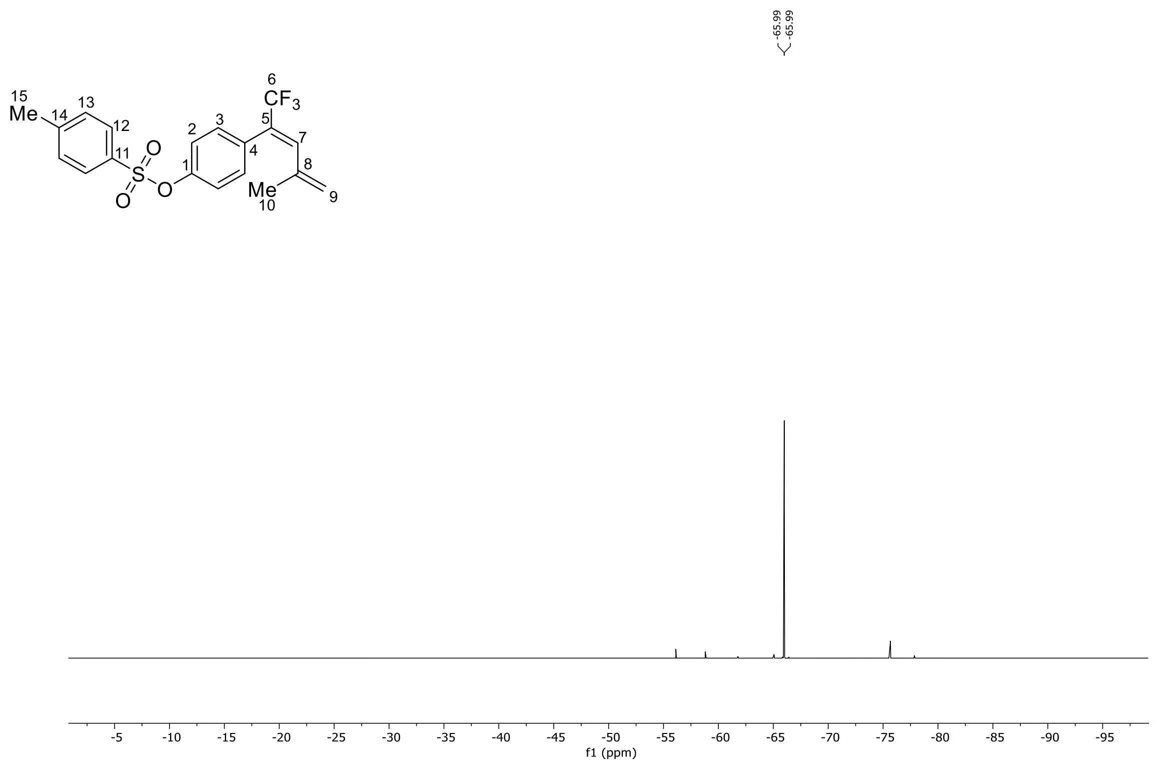

^{19}F{^{1}H} NMR (470 MHz, $CDCl_3$)

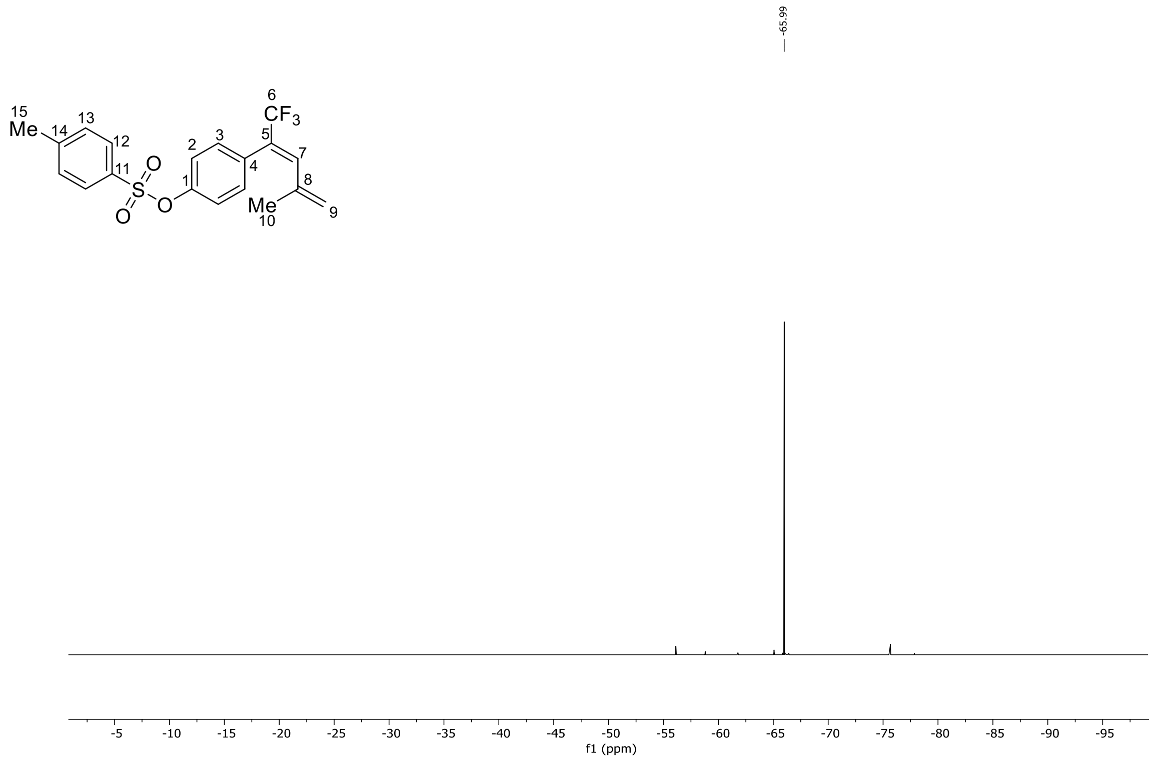

2-Cyclohexyl-1,1,1-trifluoropent-4-en-2-ol (1s-1)

^{1}H NMR (500 MHz, $CDCl_3$)

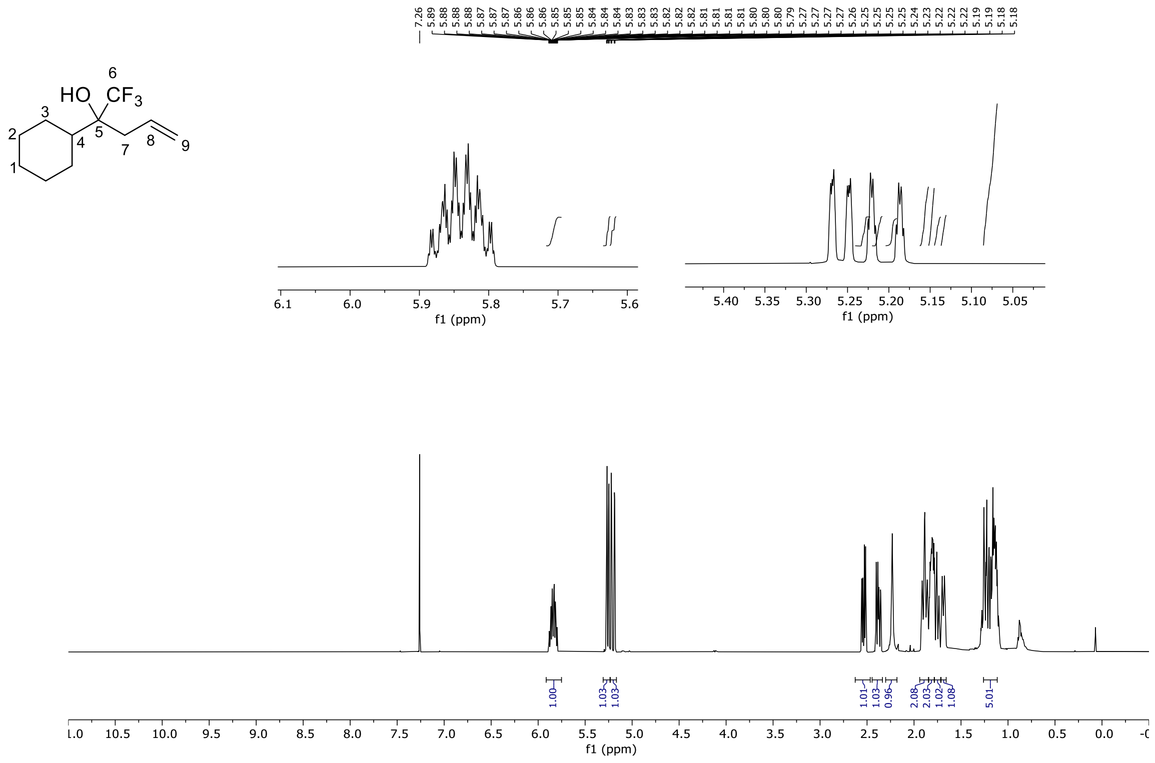

^{13}C NMR (126 MHz, $CDCl_3$)

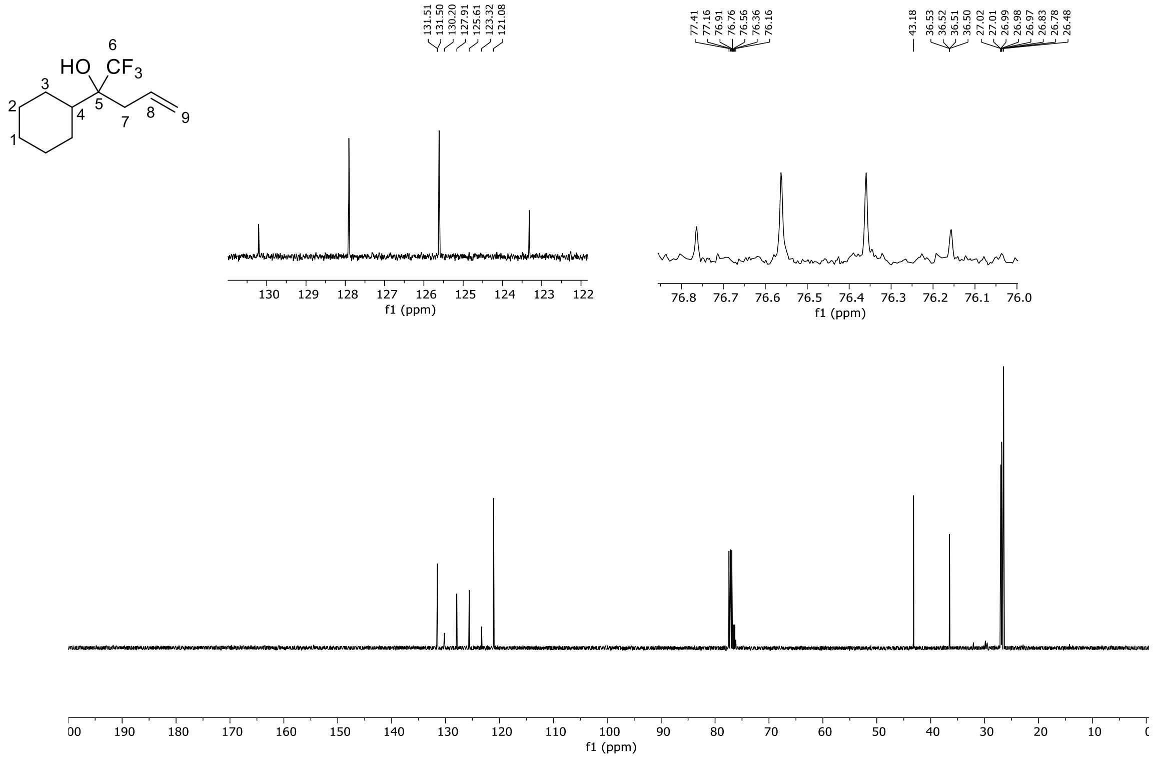

^{19}F NMR (470 MHz, $CDCl_3$)

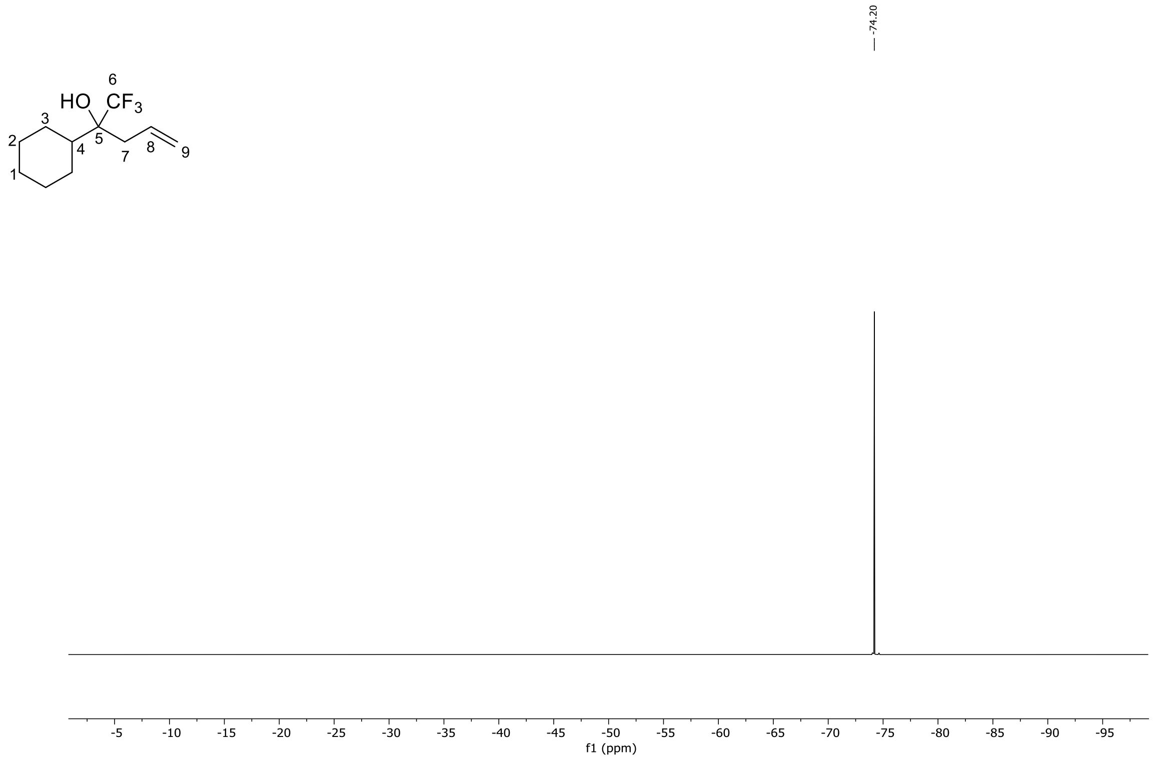

^{19}F{^{1}H} NMR (470 MHz, $CDCl_3$)

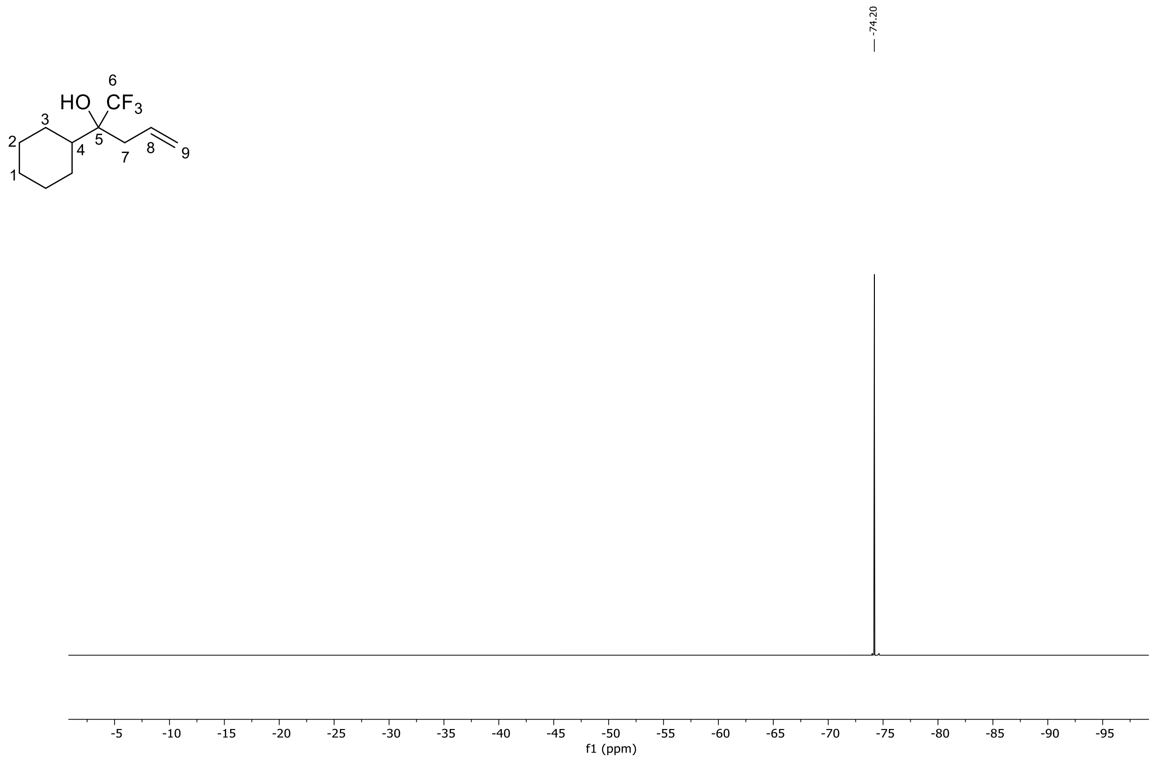

(1,1,1-Trifluoropenta-2,4-dien-2-yl)cyclohexane (1s)

^{1}H NMR (500 MHz, CDCl$_3$)

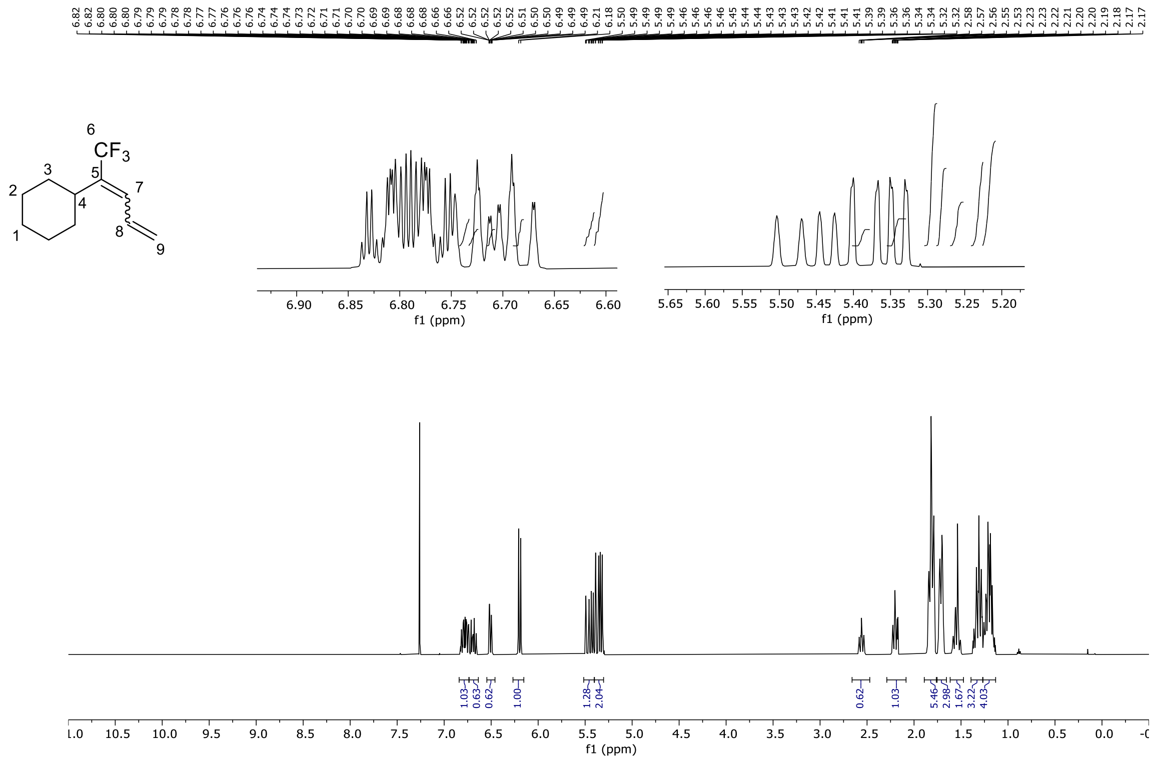

^{13}C NMR (126 MHz, CDCl$_3$)

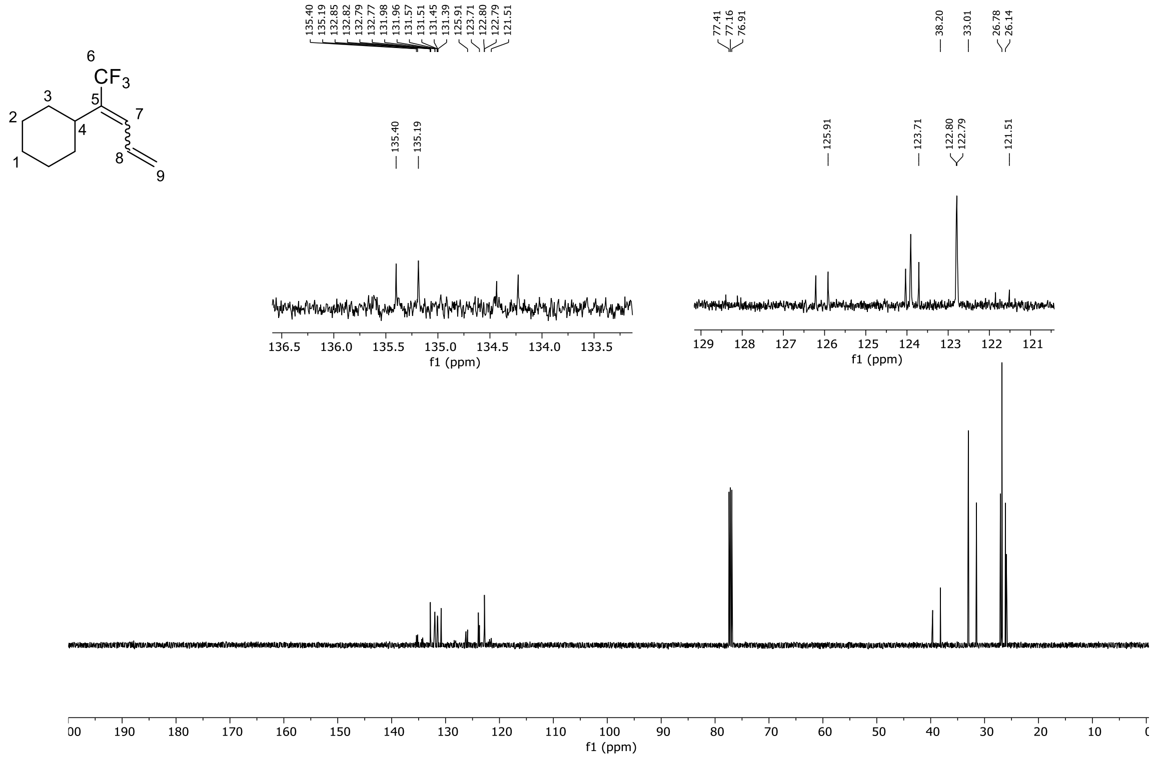

^{19}F NMR (470 MHz, $CDCl_3$)

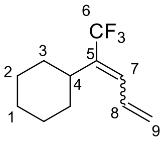

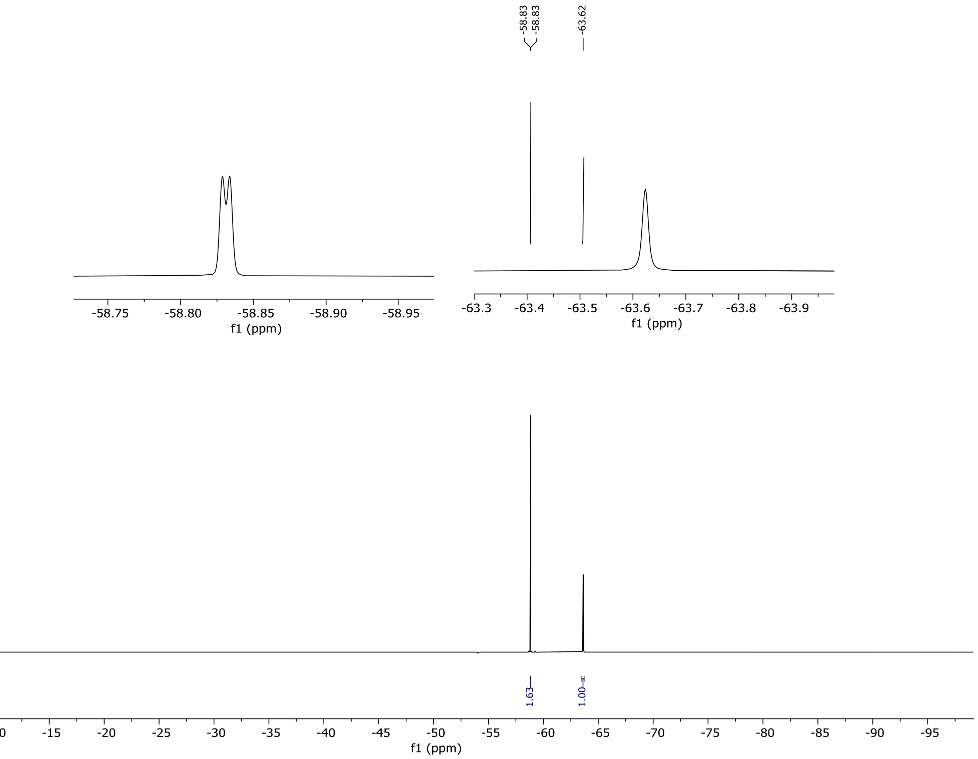

^{19}F{^{1}H} NMR (470 MHz, $CDCl_3$)

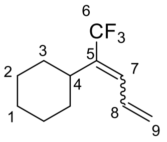

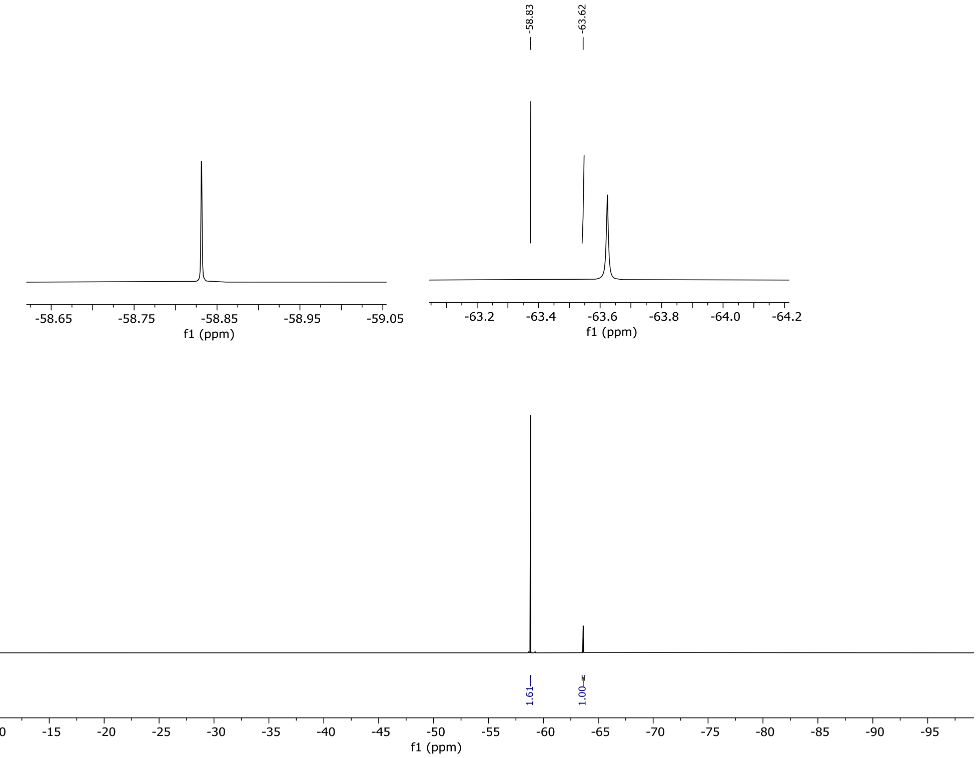

2-(4-(1,1,1-Trifluoro-2-hydroxypent-4-en-2-yl)phenyl)isoindoline-1,3-dione (1t-1)

^{1}H NMR (500 MHz, DMSO-d_6)

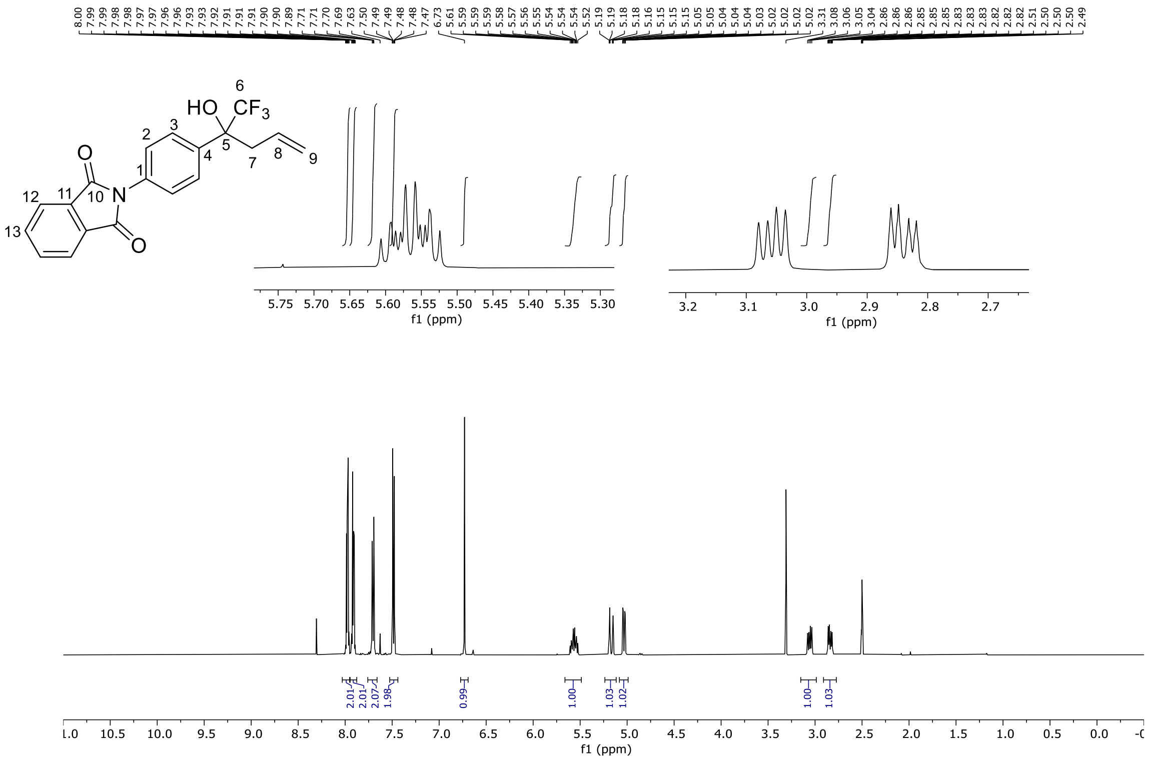

^{13}C NMR (126 MHz, DMSO-d_6)

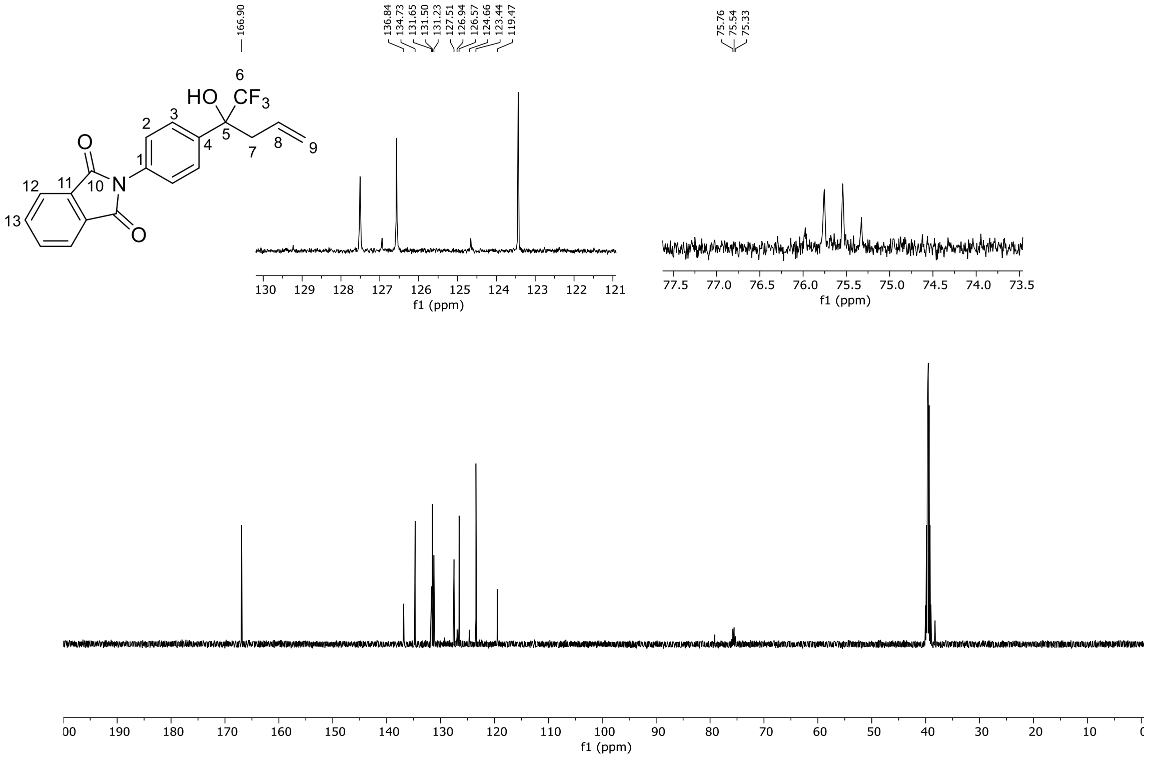

^{19}F NMR (470 MHz, DMSO-d_6)

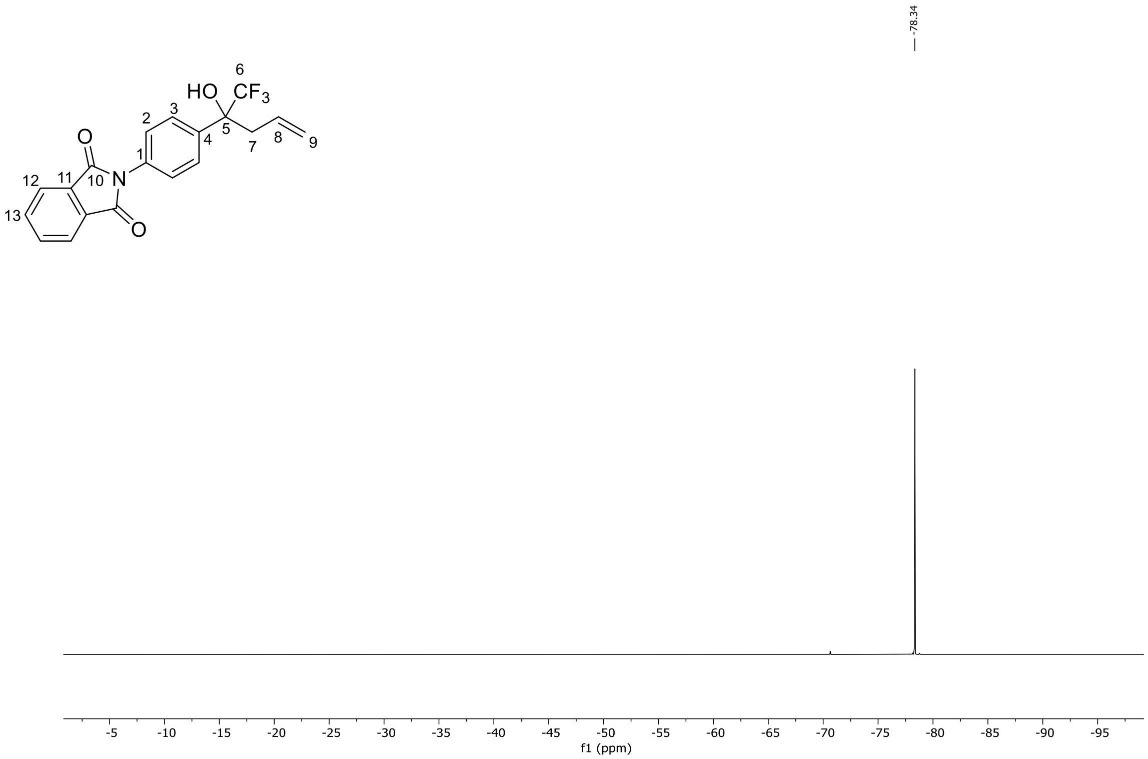

^{19}F{^{1}H} NMR (470 MHz, DMSO-d_6)

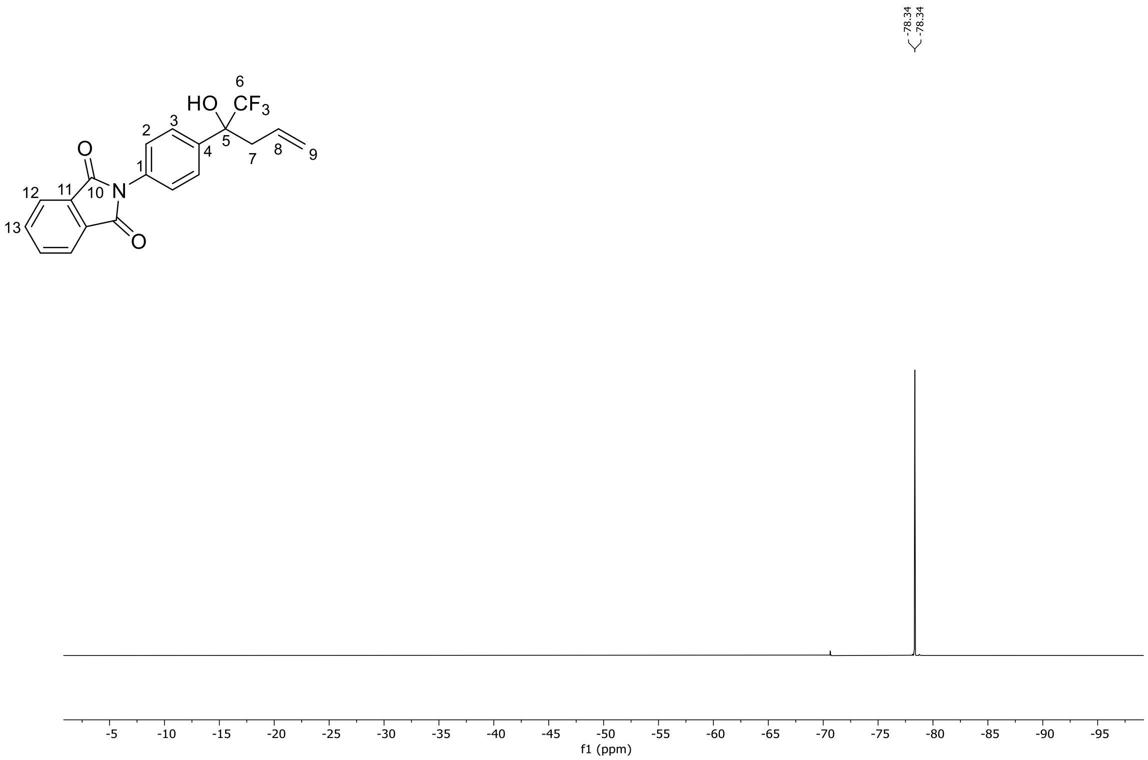

2-(4-(1,1,1-Trifluoropenta-2,4-dien-2-yl)phenyl)isoindoline-1,3-dione (1t)

^{1}H NMR (500 MHz, $CDCl_3$)

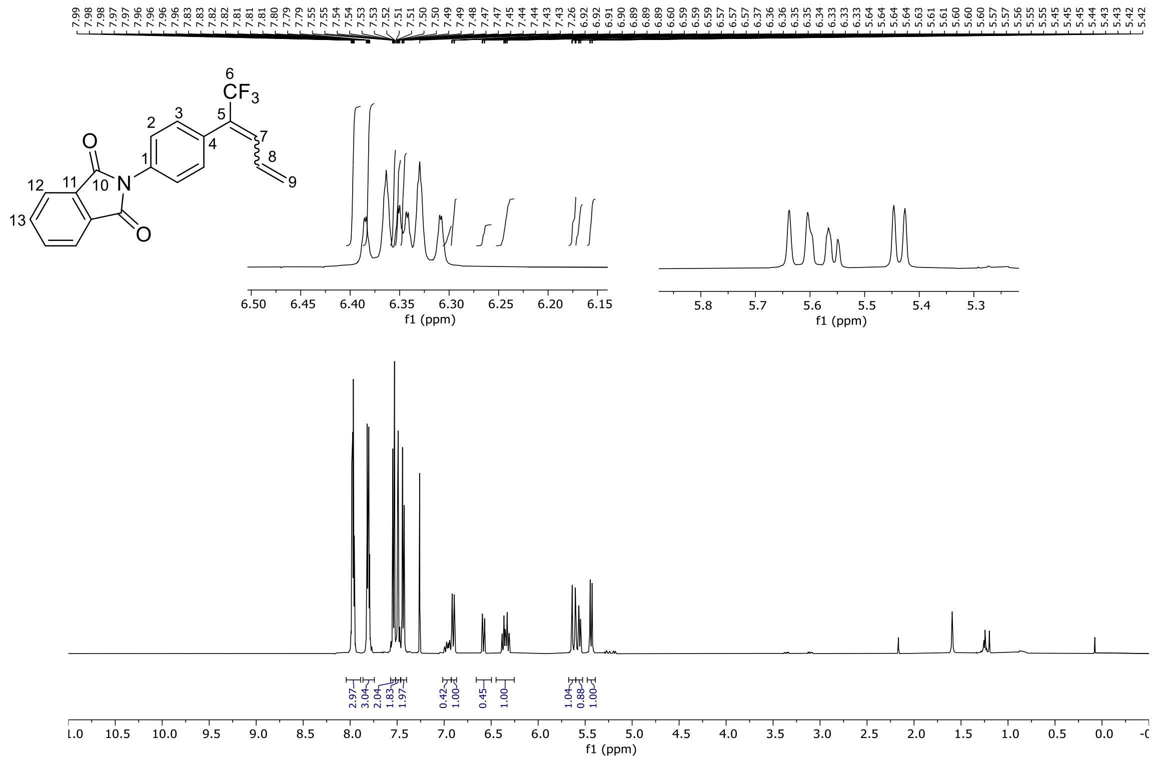

^{13}C NMR (126 MHz, $CDCl_3$)

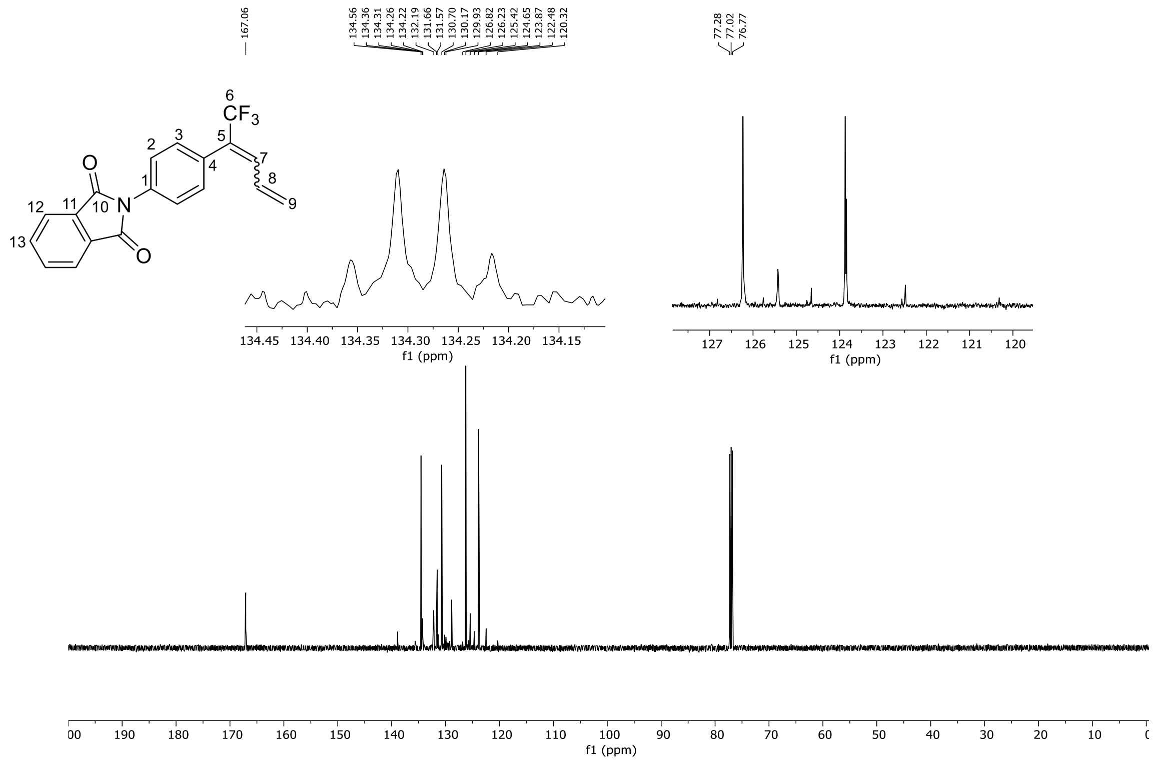

^{19}**F NMR** (470 MHz, $CDCl_3$)

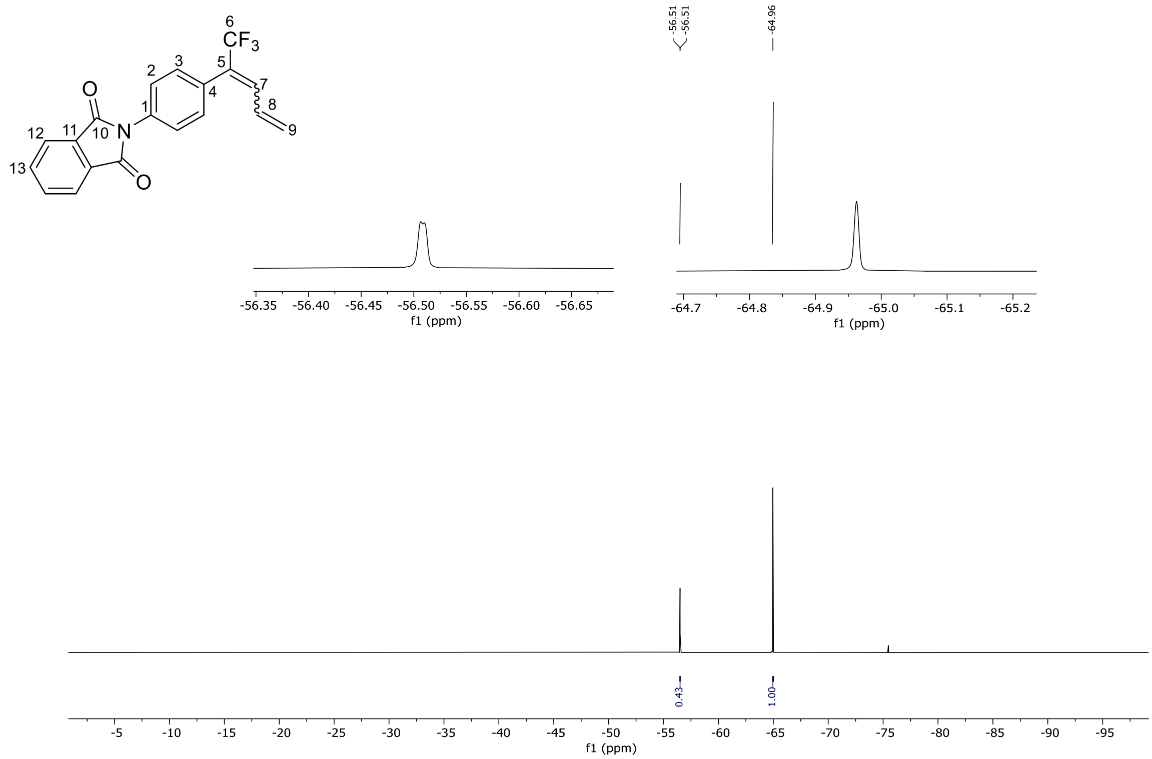

^{19}**F{^{1}H} NMR** (470 MHz, $CDCl_3$)

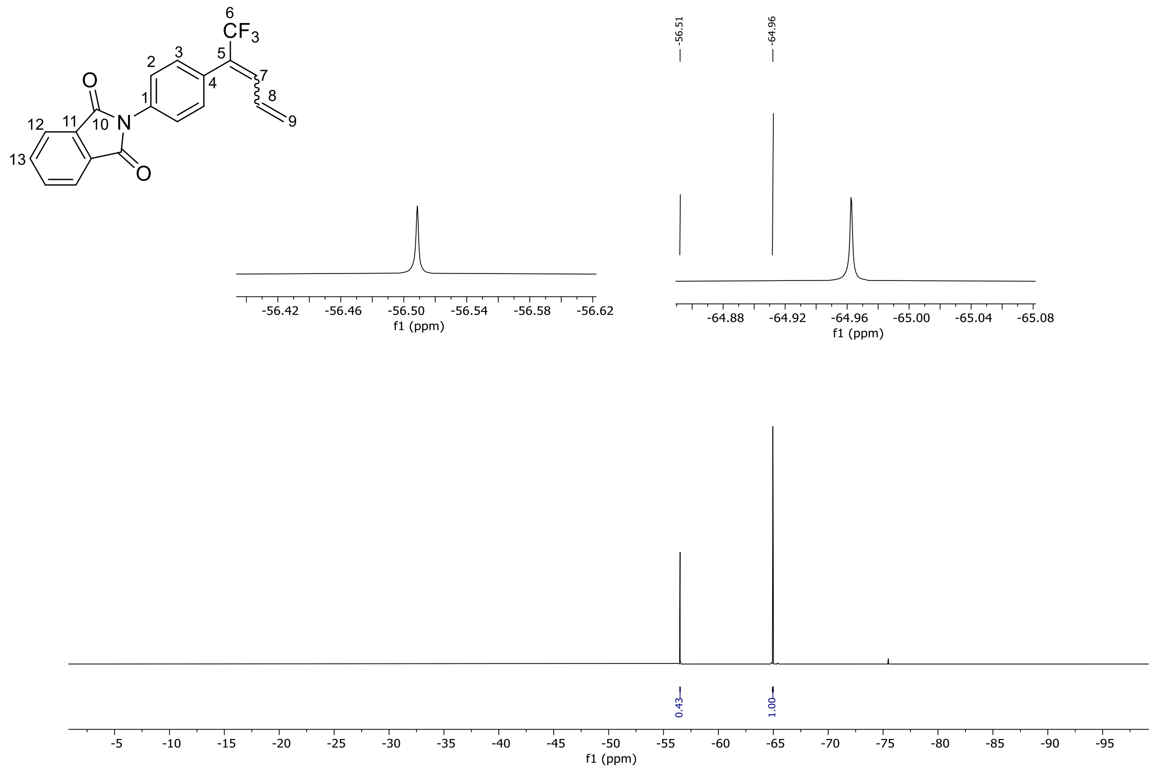

(*E*)-1-Bromo-4-(1,1,1,2,5-pentafluoropent-3-en-2-yl)benzene (2a)

^{1}H NMR (599 MHz, $CDCl_3$)

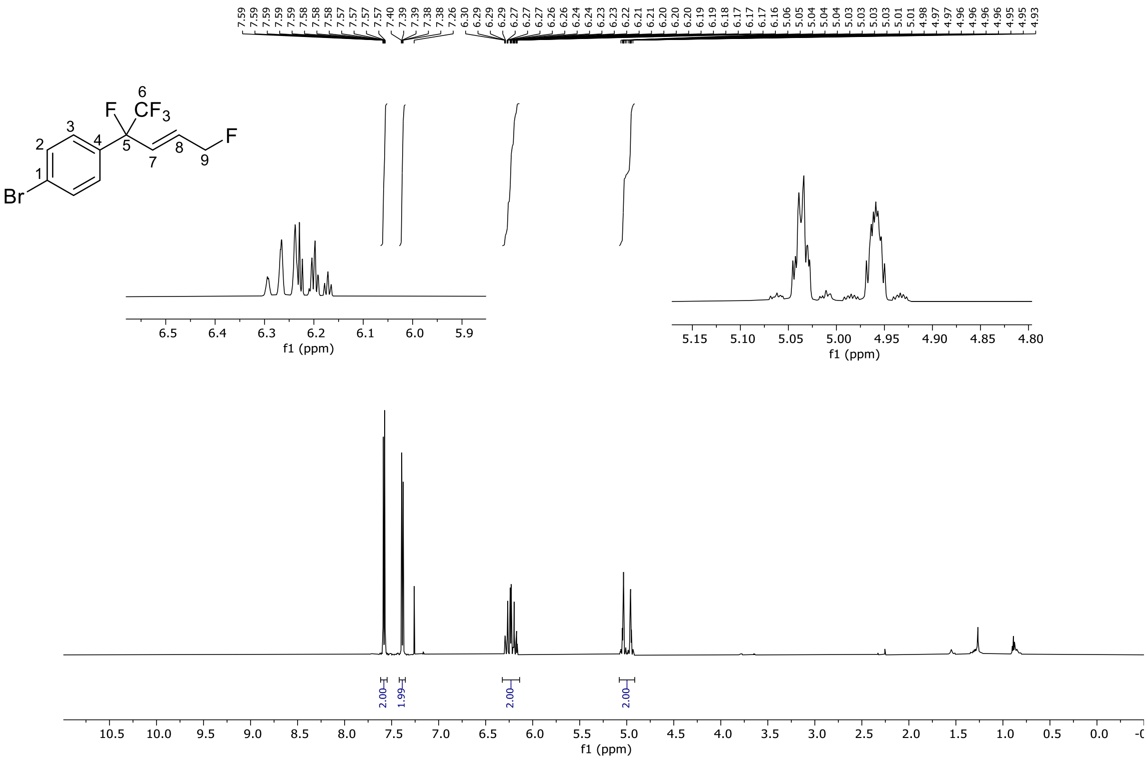

^{13}C NMR (151 MHz, $CDCl_3$)

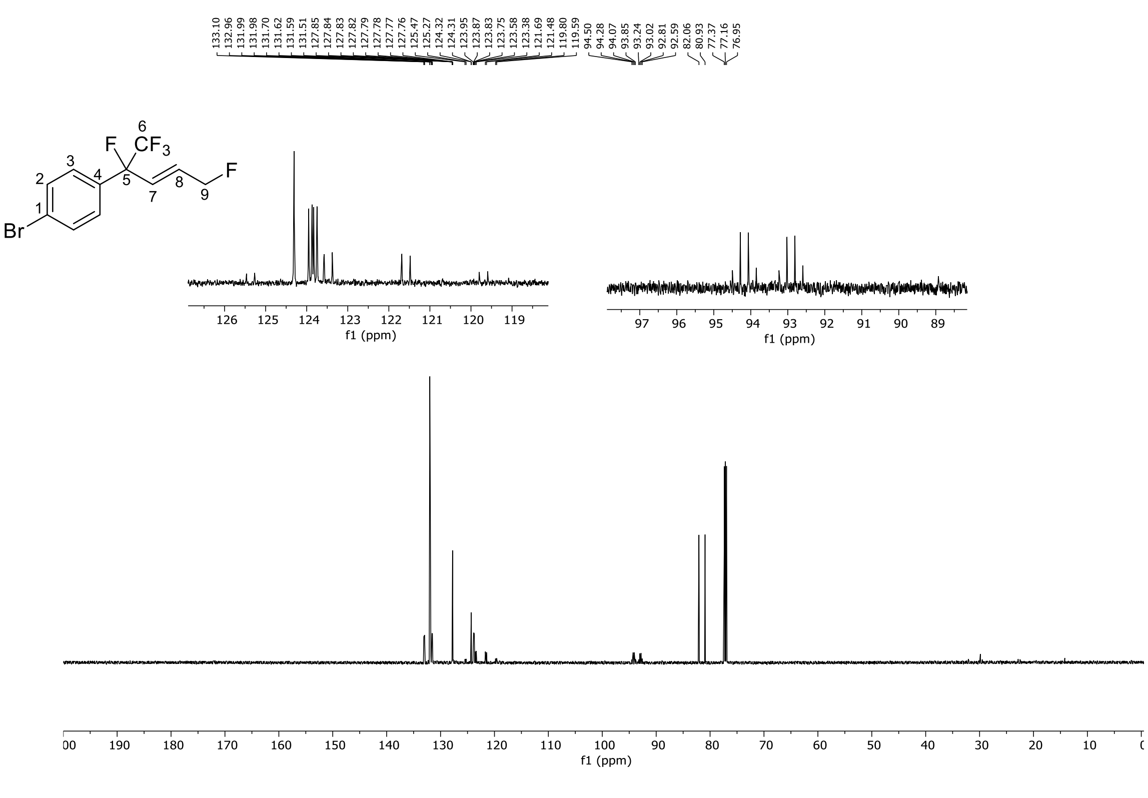

^{19}F NMR (564 MHz, $CDCl_3$)

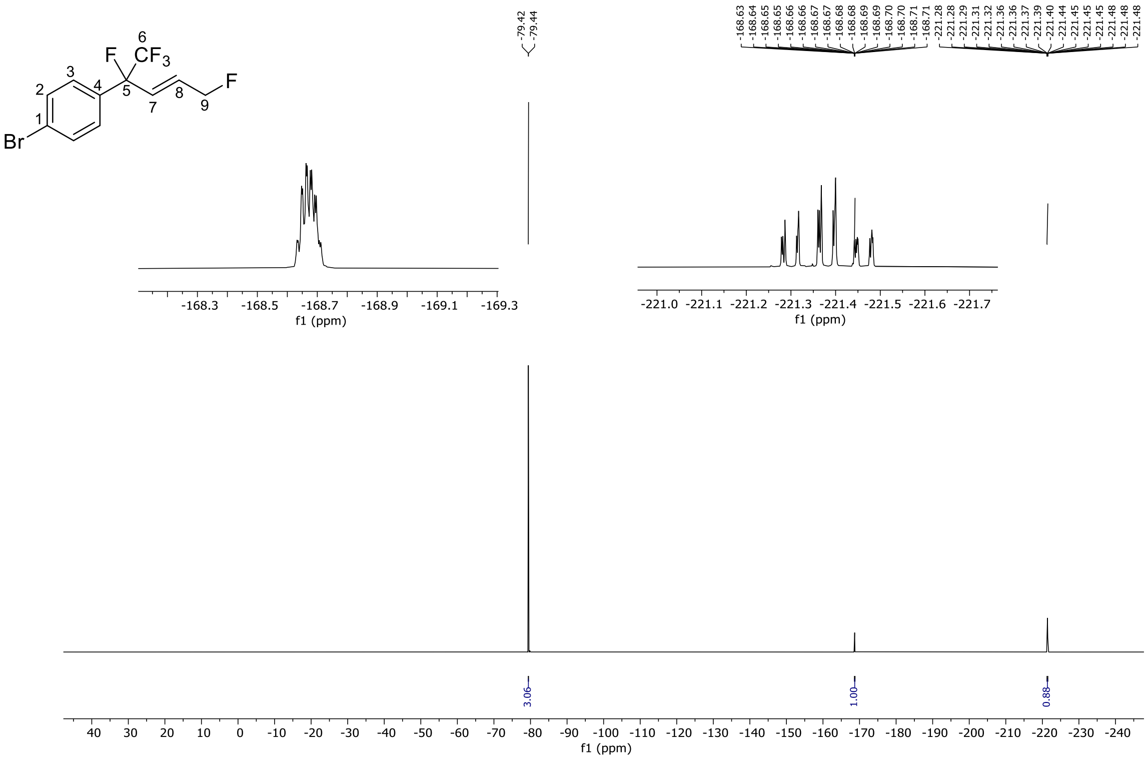

^{19}F{^{1}H} NMR (564 MHz, $CDCl_3$)

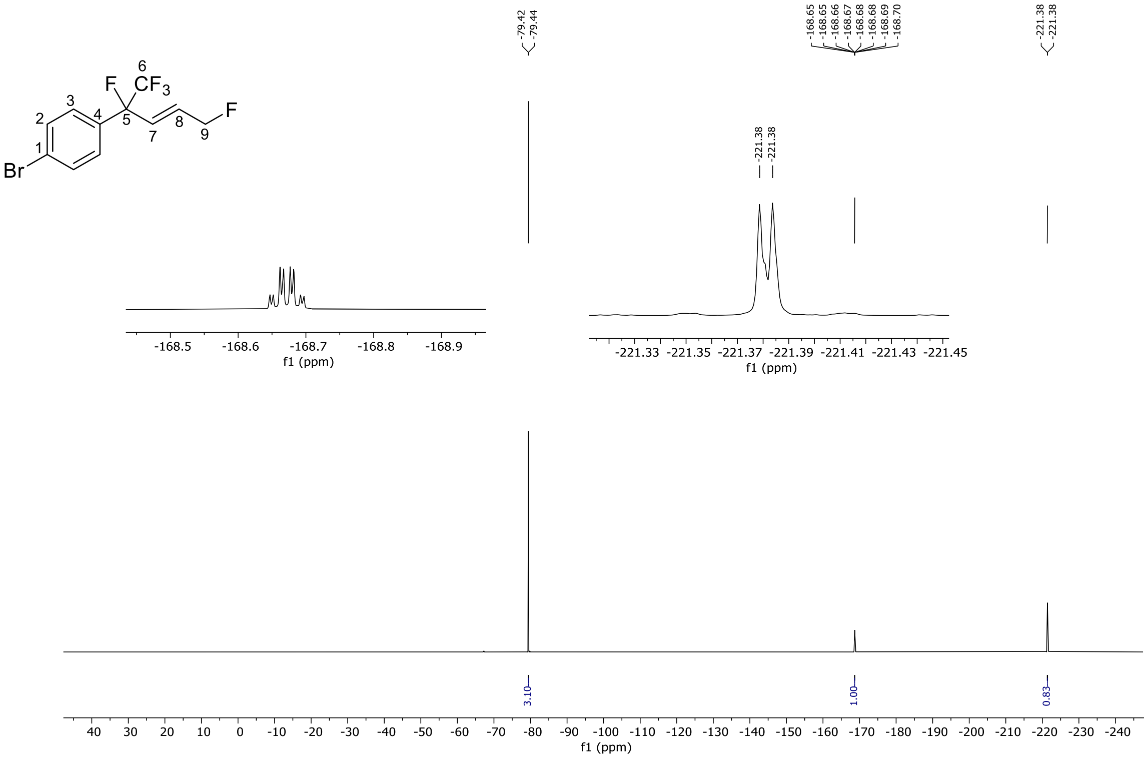

(*E*)-1-Bromo-3-(1,1,1,2,5-pentafluoropent-3-en-2-yl)benzene (2b)

¹H NMR (500 MHz, $CDCl_3$)

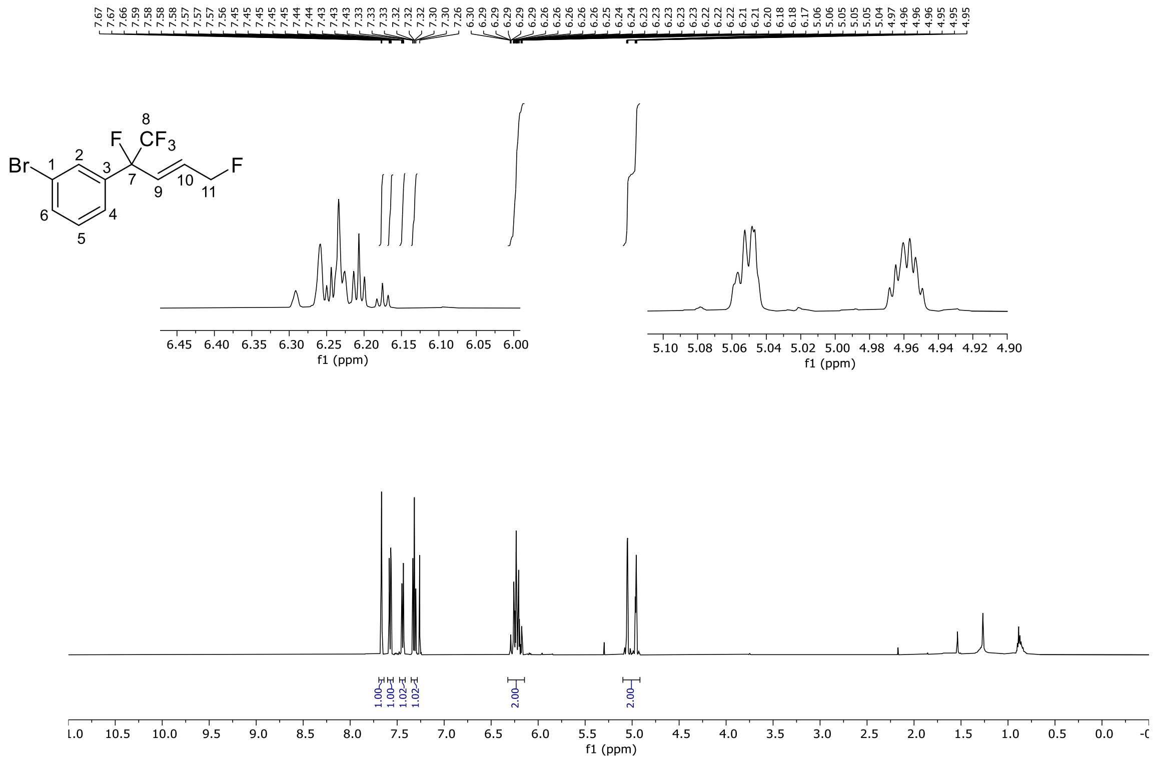

¹³C NMR (126 MHz, $CDCl_3$)

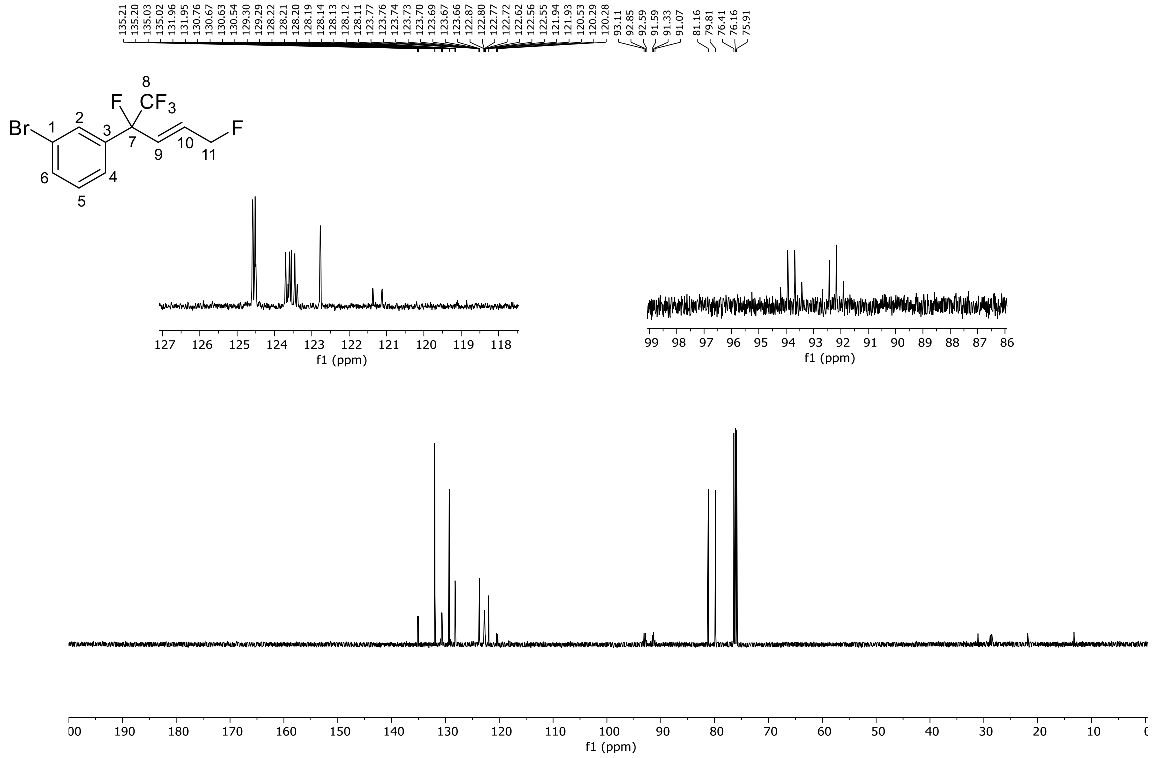

^{19}F NMR (470 MHz, $CDCl_3$)

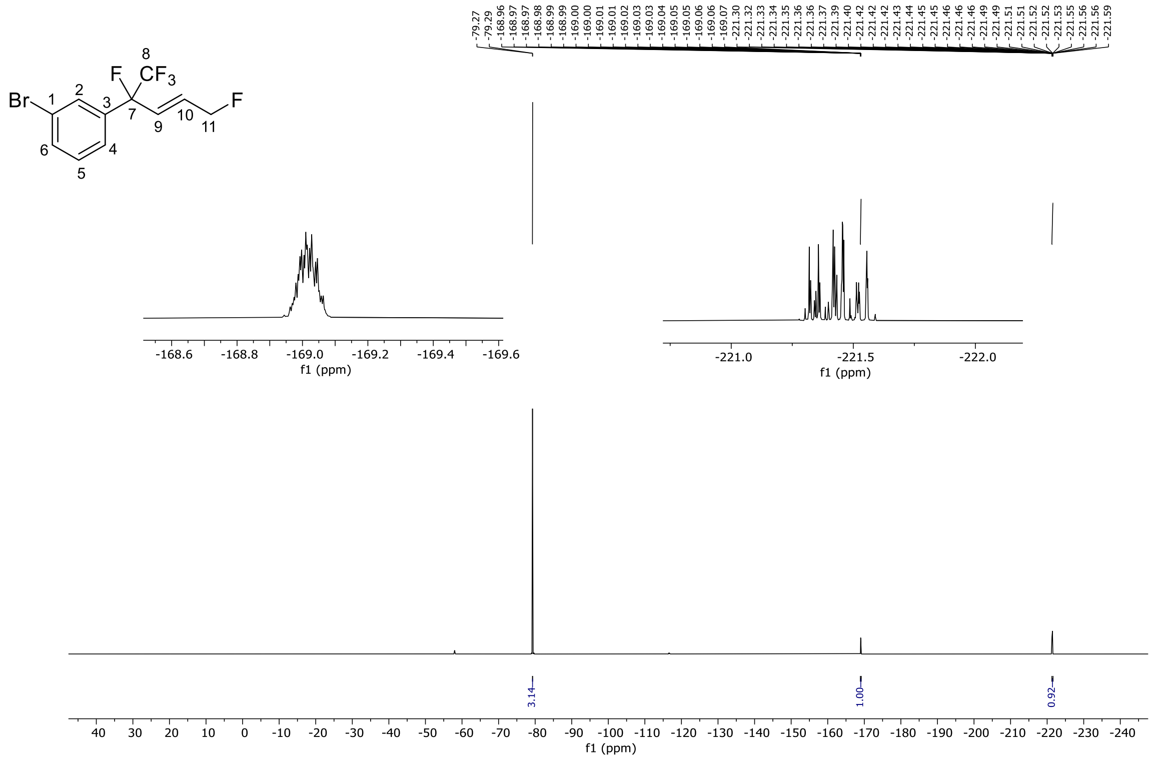

^{19}F{^{1}H} NMR (470 MHz, $CDCl_3$)

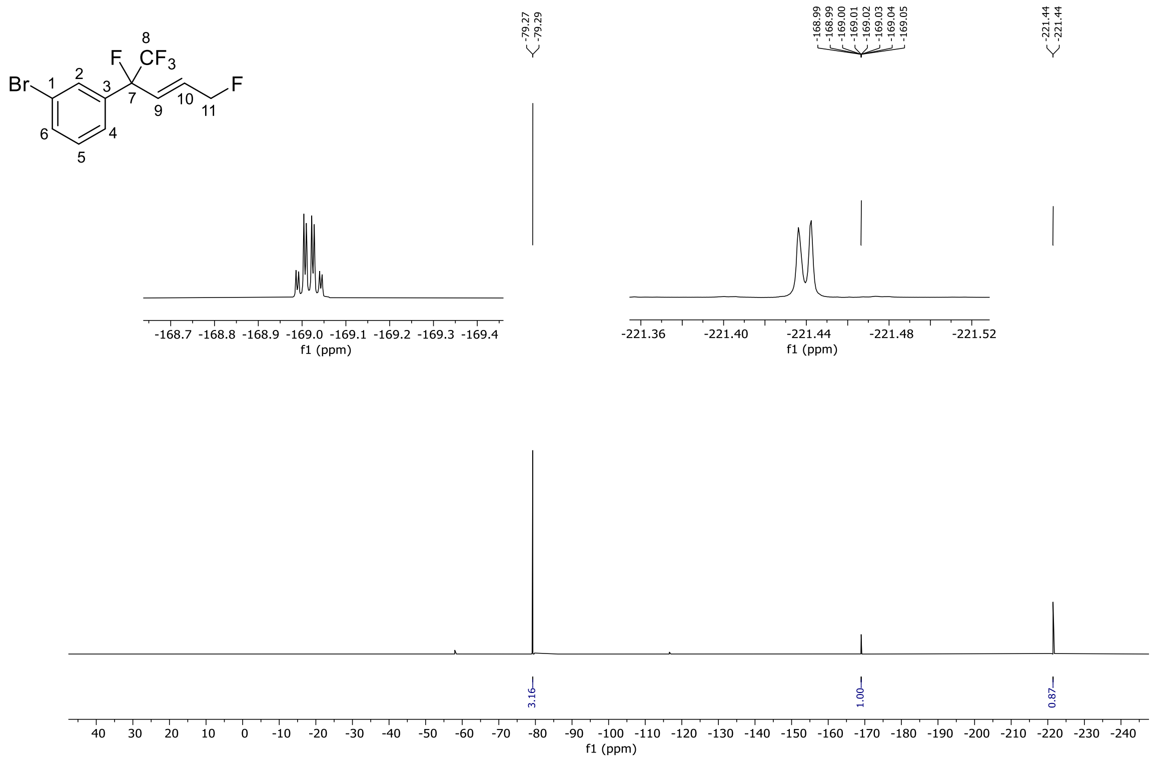

(*E*)-1-Chloro-4-(1,1,1,2,5-pentafluoropent-3-en-2-yl)benzene (2c)

^{1}H NMR (500 MHz, $CDCl_3$)

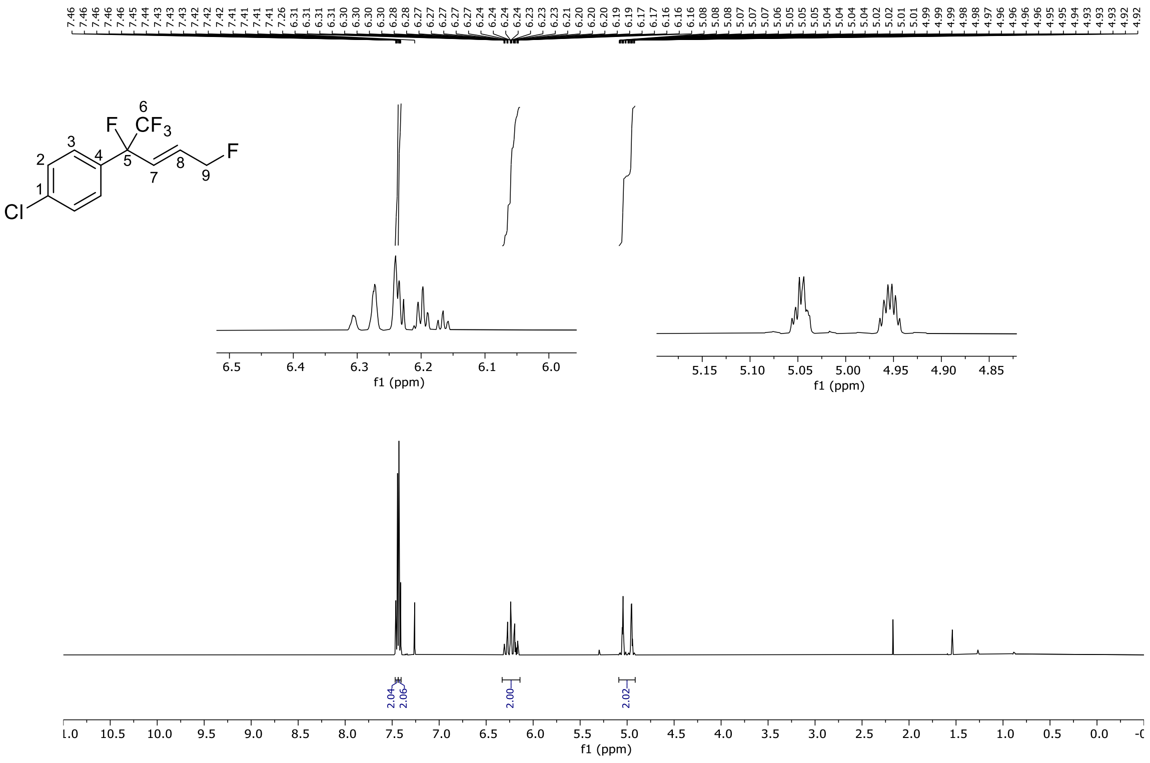

^{13}C NMR (126 MHz, $CDCl_3$)

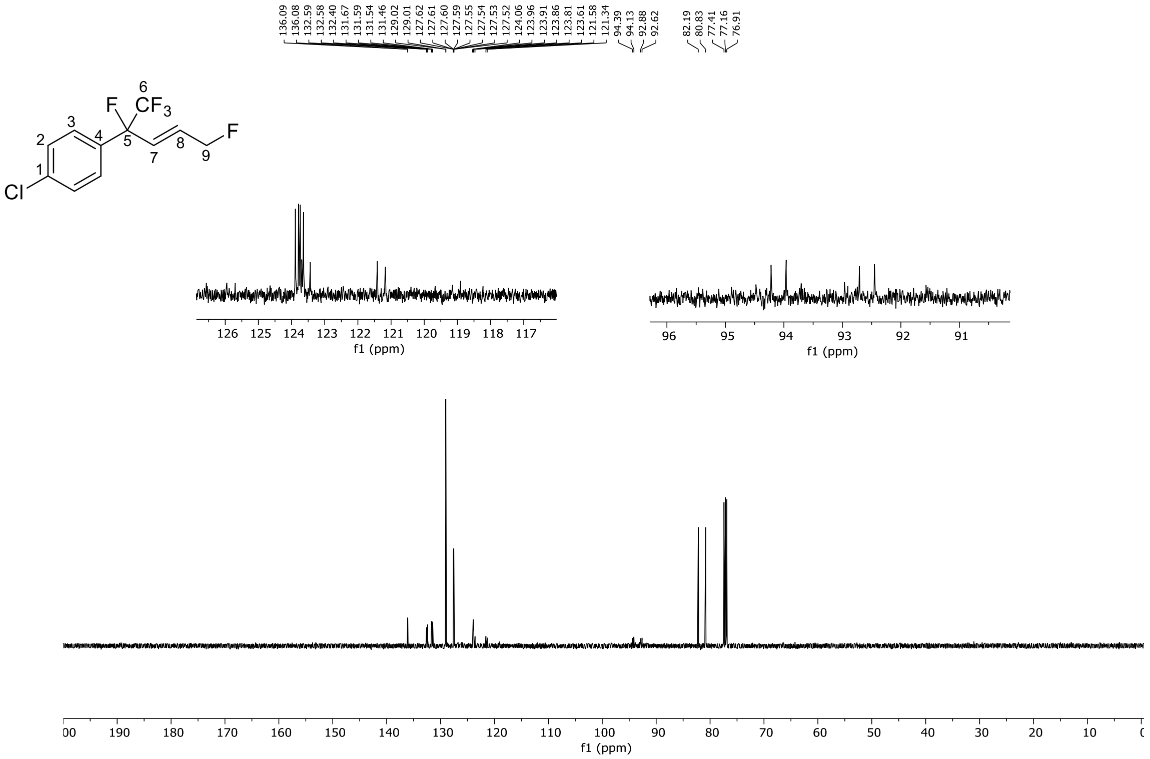

^{19}F NMR (470 MHz, $CDCl_3$)\

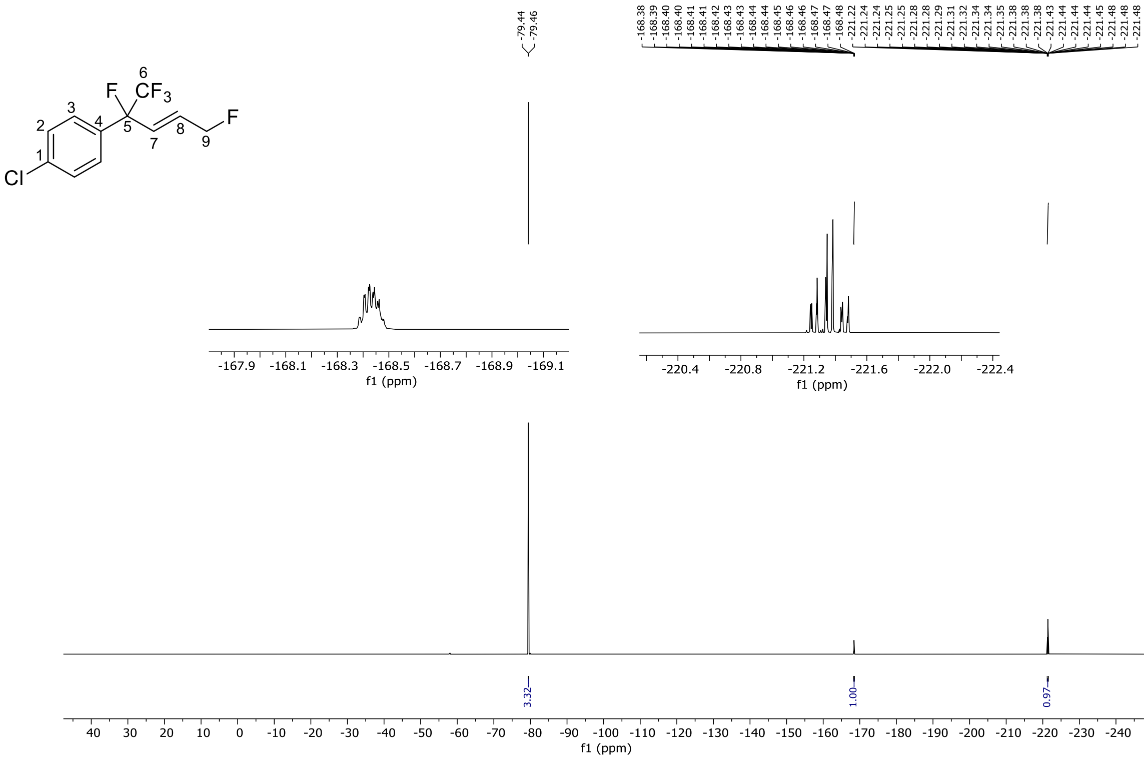

^{19}F{^{1}H} NMR (376 MHz, $CDCl_3$)

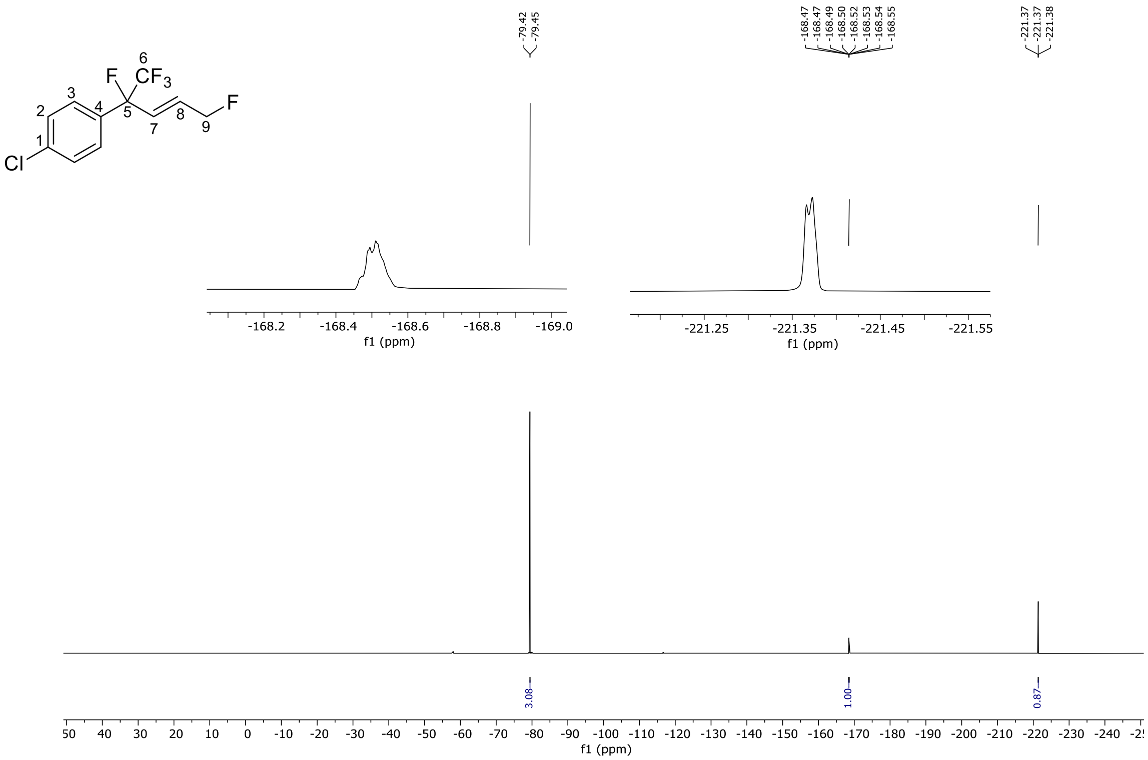

(*E*)-1-Chloro-3-(1,1,1,2,5-pentafluoropent-3-en-2-yl)benzene (2d)

^{1}H NMR (500 MHz, $CDCl_3$)

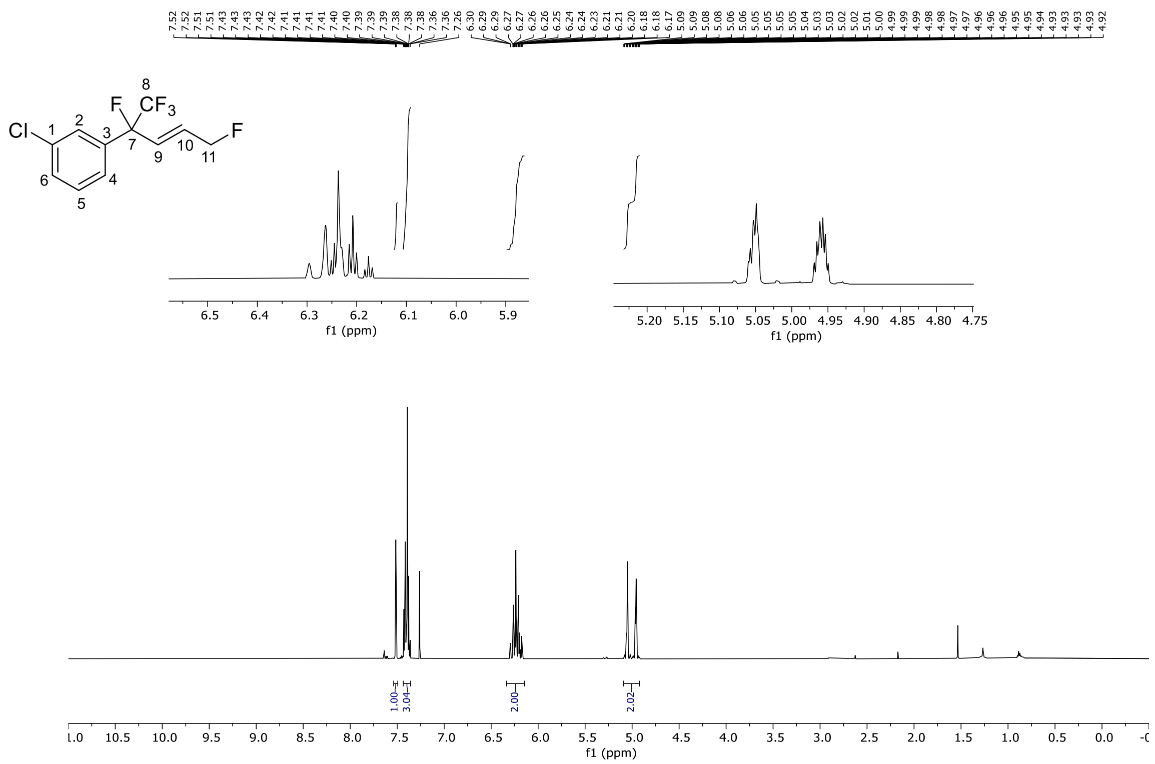

^{13}C NMR (126 MHz, $CDCl_3$)\

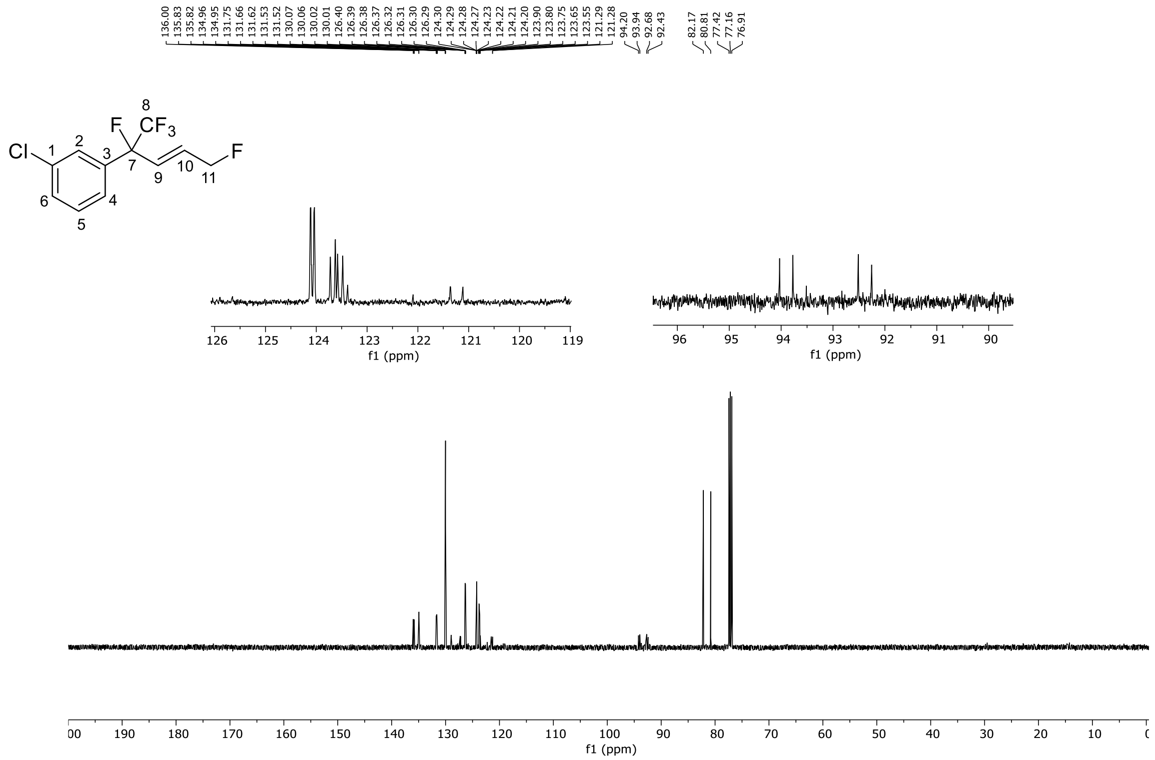

^{19}F NMR (470 MHz, CDCl$_3$)

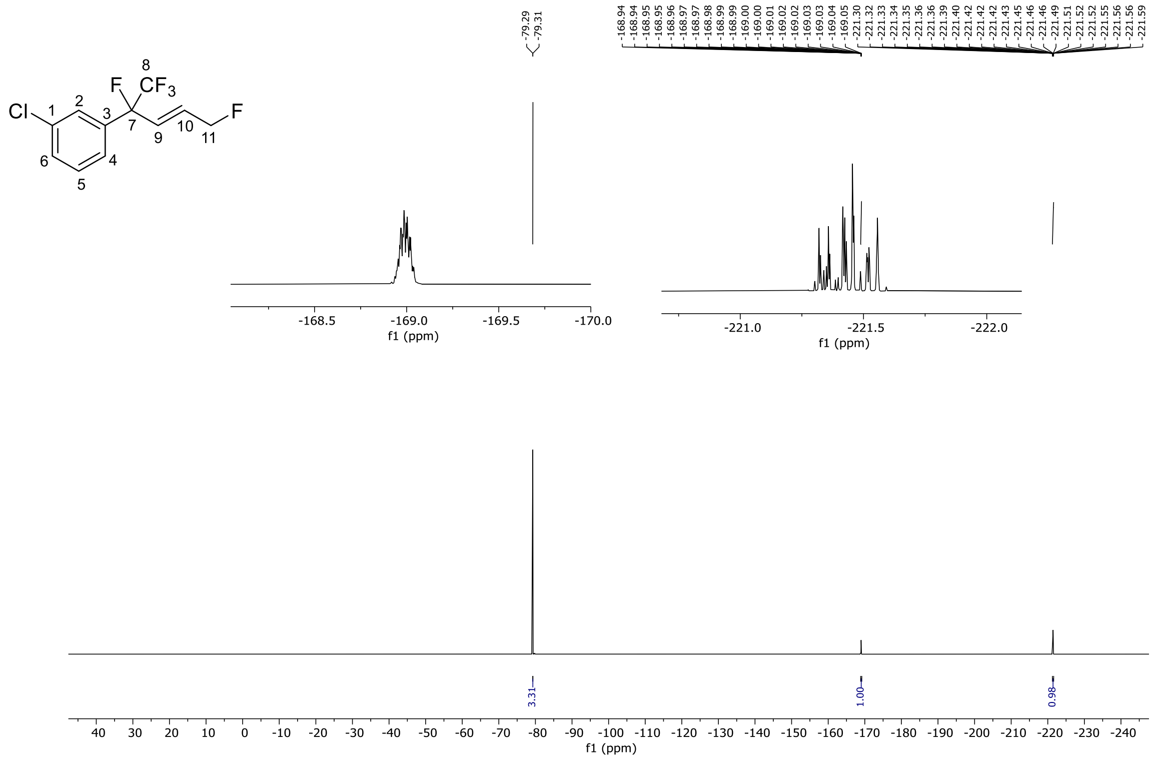

^{19}F{^{1}H} NMR (470 MHz, CDCl$_3$)

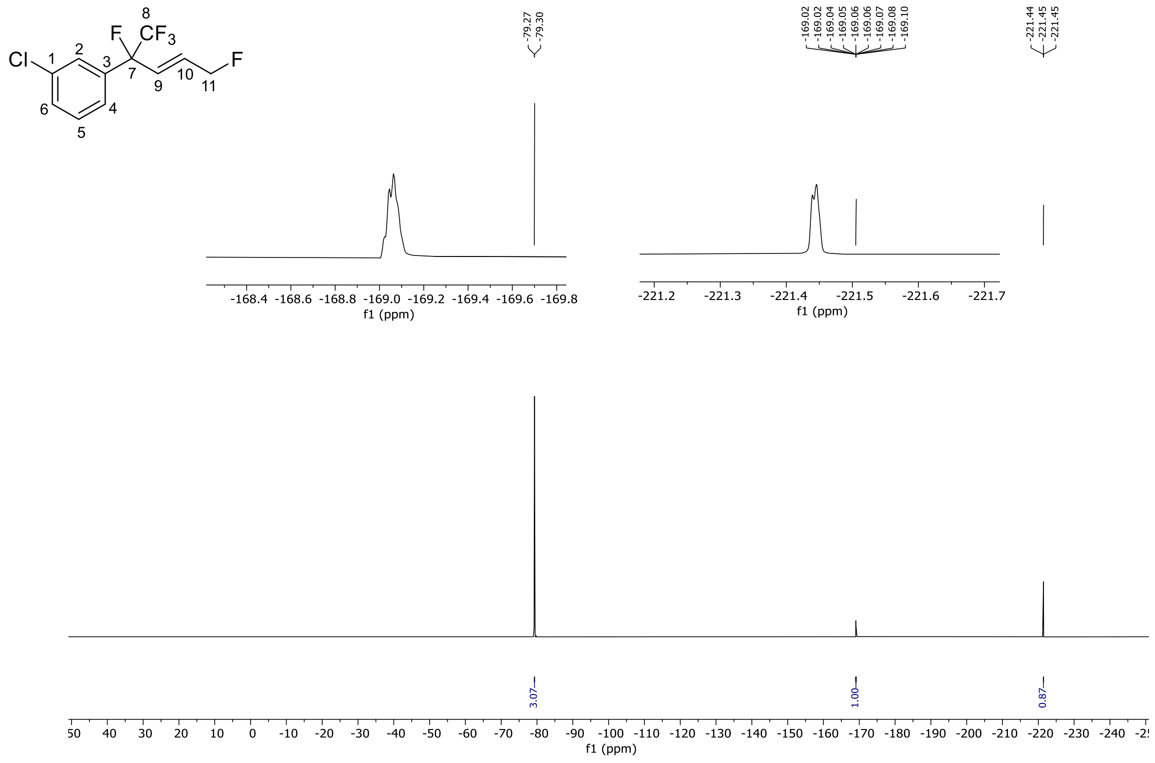

(*E*)-1,3-Dichloro-5-(1,1,1,2,5-pentafluoropent-3-en-2-yl)benzene (2e)

^{1}H NMR (500 MHz, $CDCl_3$)

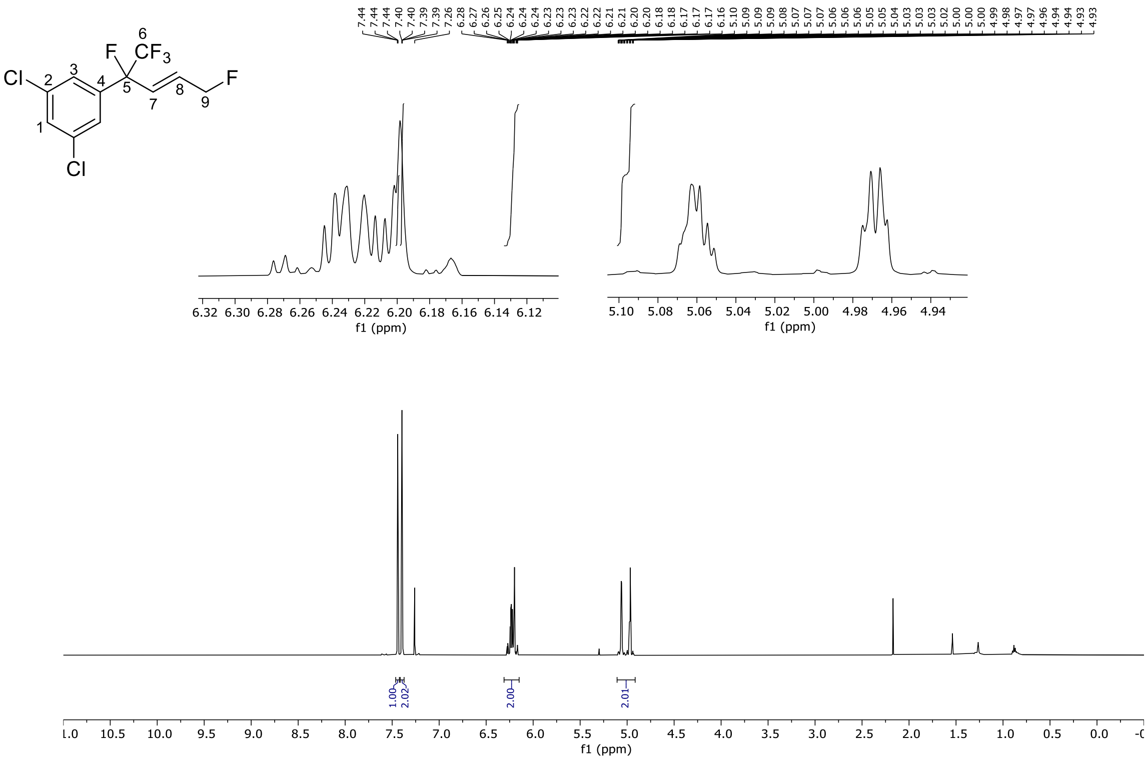

^{13}C NMR (126 MHz, $CDCl_3$)

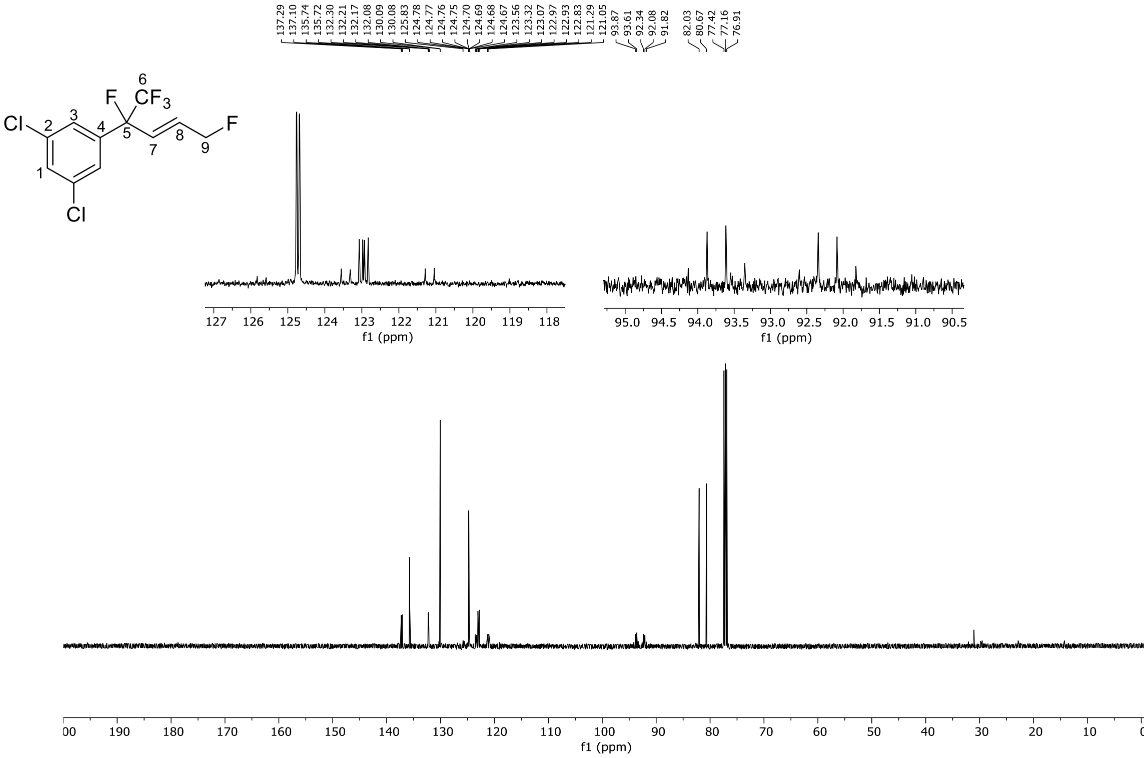

^{19}F NMR (470 MHz, $CDCl_3$)

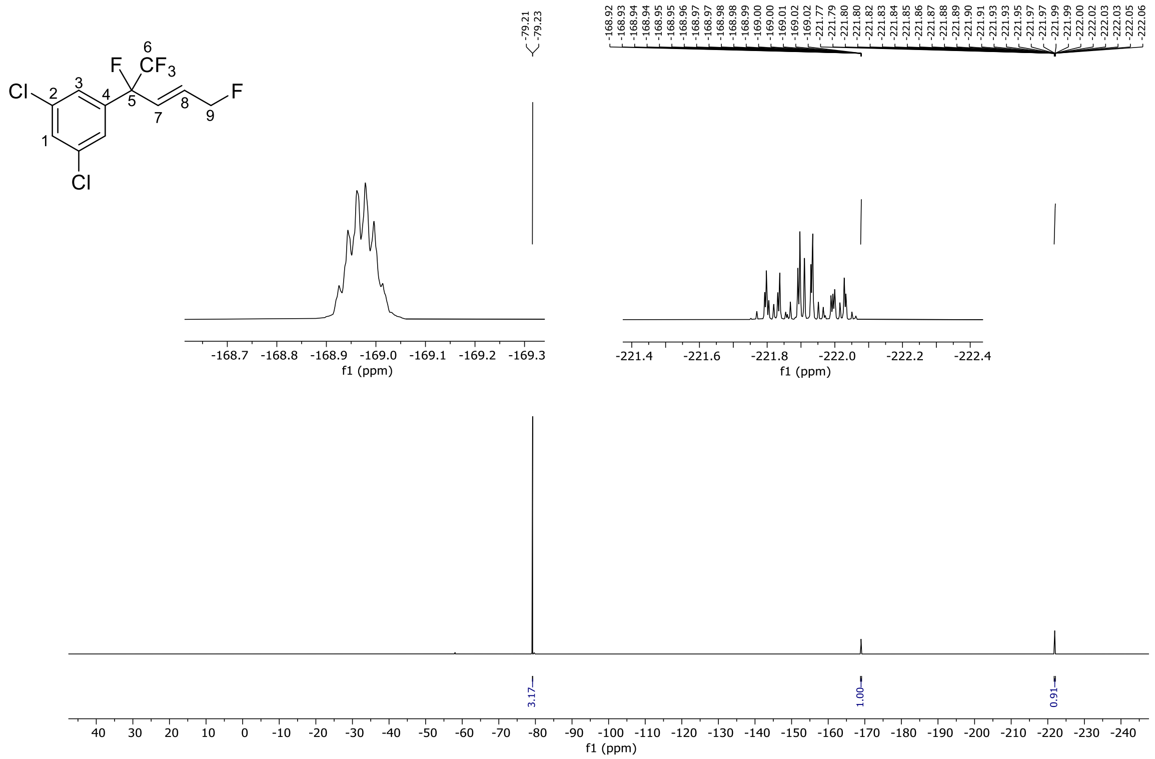

^{19}F{^{1}H} NMR (376 MHz, $CDCl_3$)

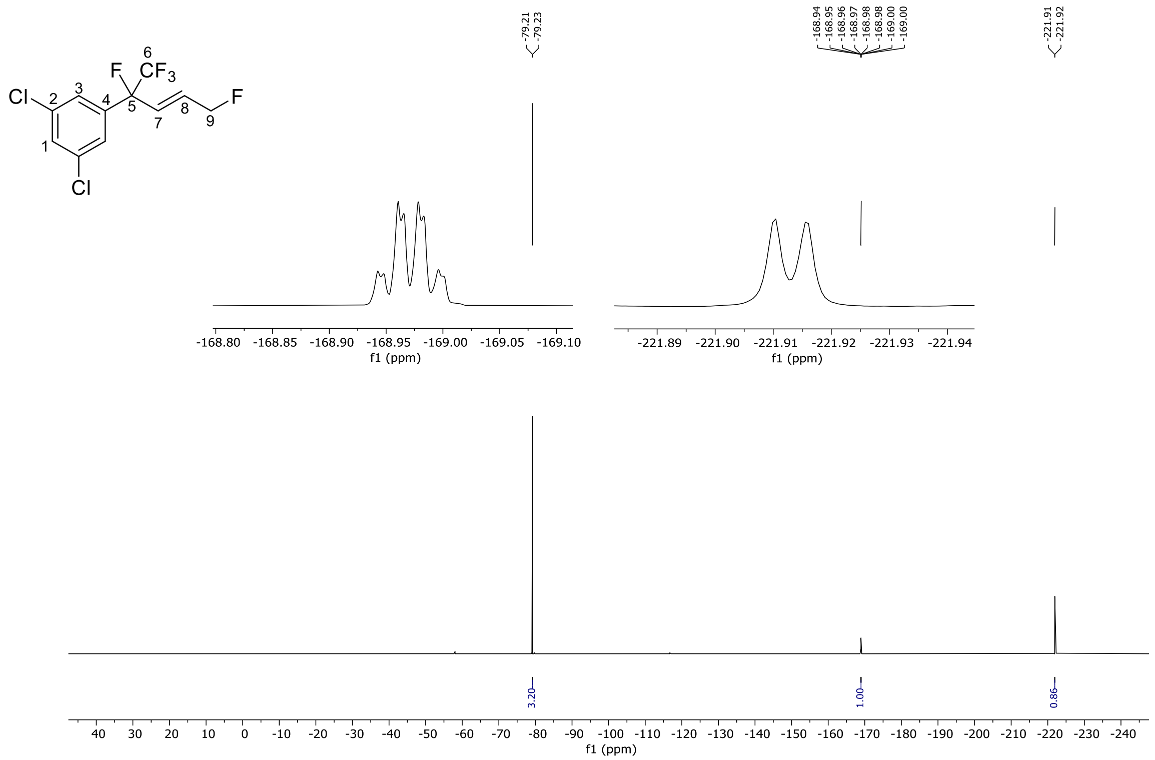

(*E*)-1-Fluoro-4-(1,1,1,2,5-pentafluoropent-3-en-2-yl)benzene (2f)

^{1}H NMR (500 MHz, $CDCl_3$)

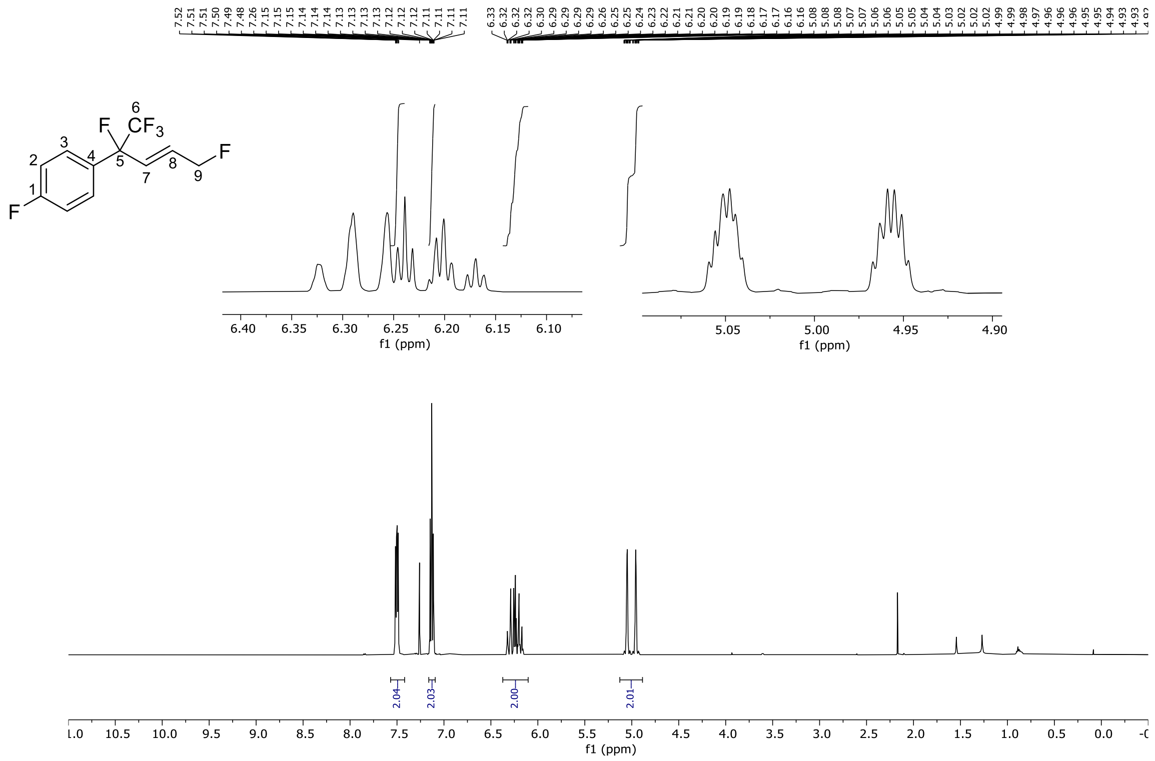

^{13}C NMR (126 MHz, $CDCl_3$)

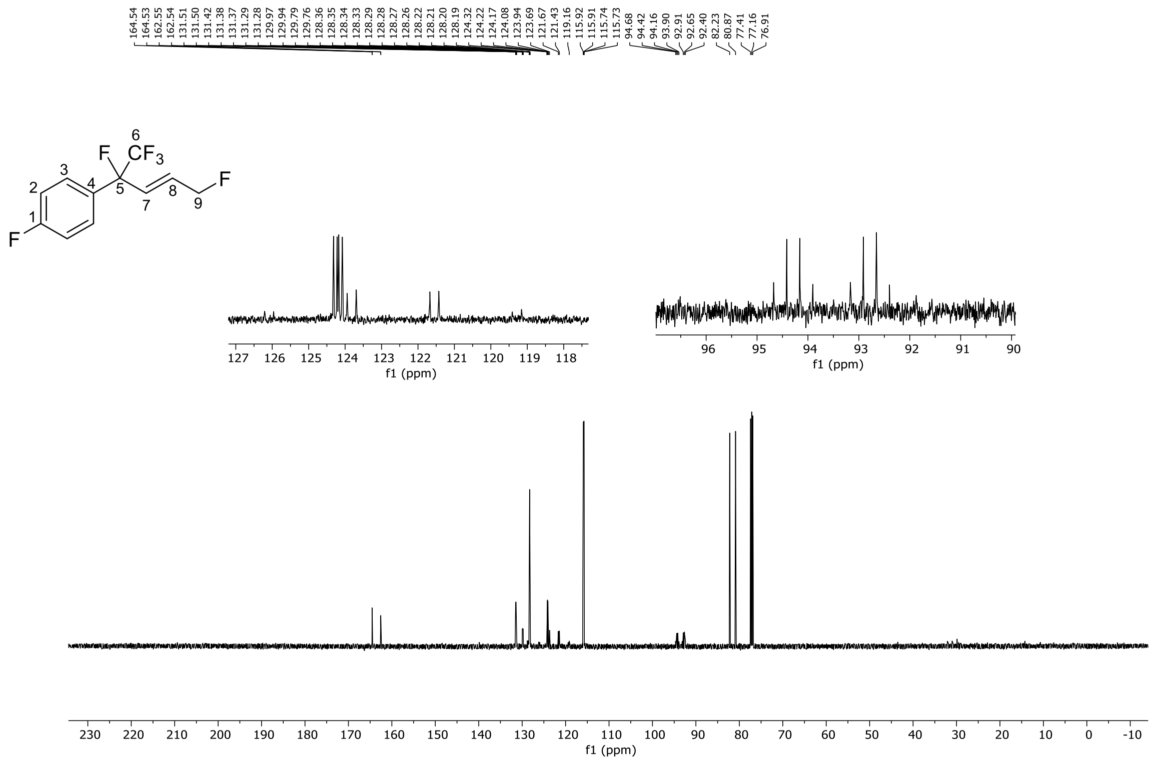

^{19}**F NMR** (470 MHz, $CDCl_3$)

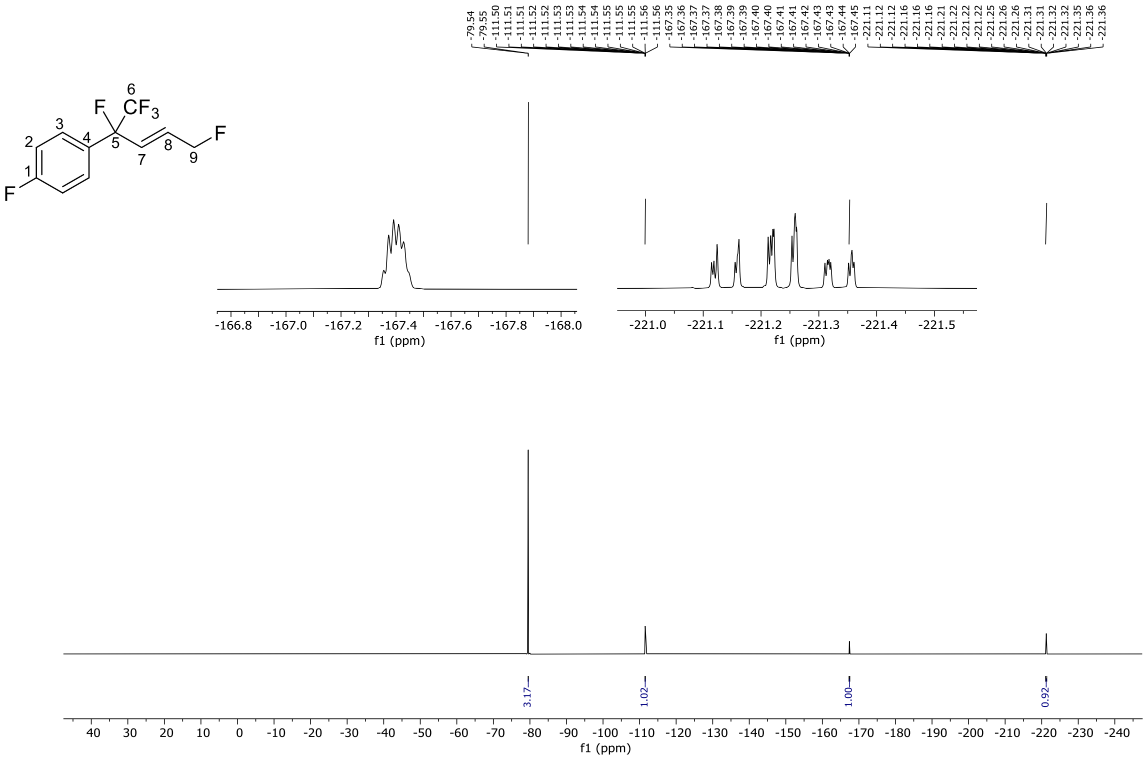

^{19}**F{^{1}H} NMR** (376 MHz, $CDCl_3$)

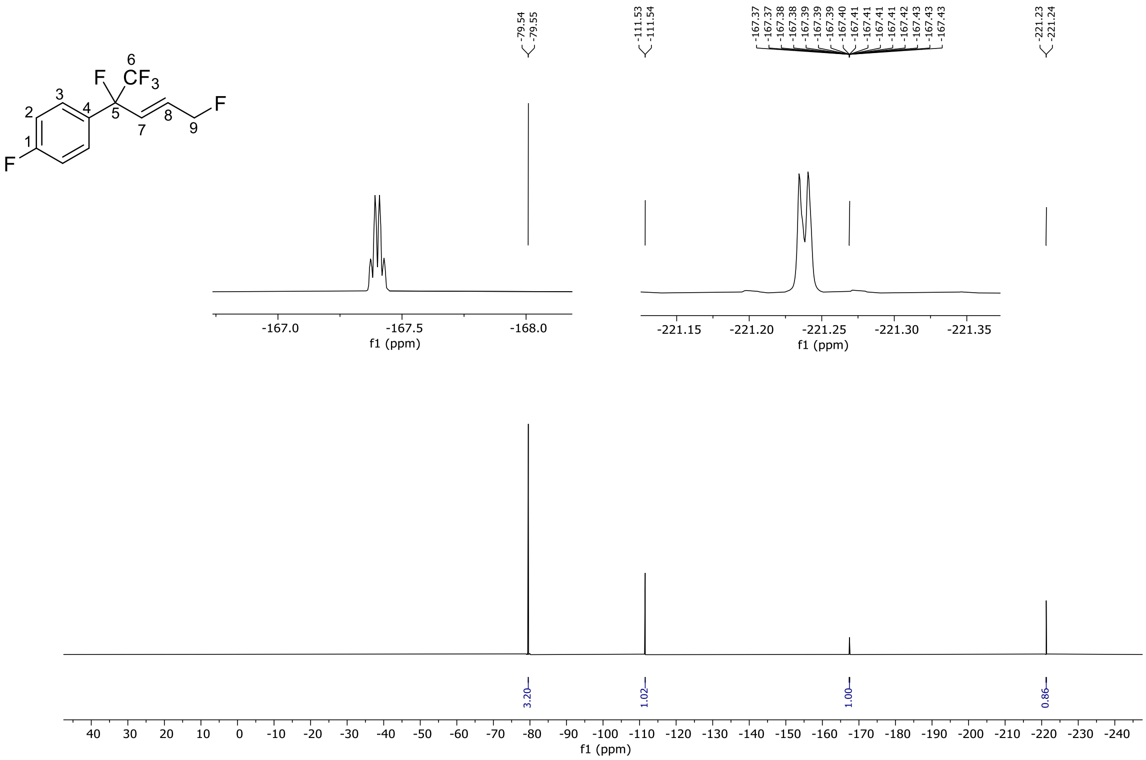

(*E*)-4-Fluoro-2-methyl-1-(1,1,1,2,5-pentafluoropent-3-en-2-yl)benzene (2g)

^{1}H NMR (500 MHz, $CDCl_3$)

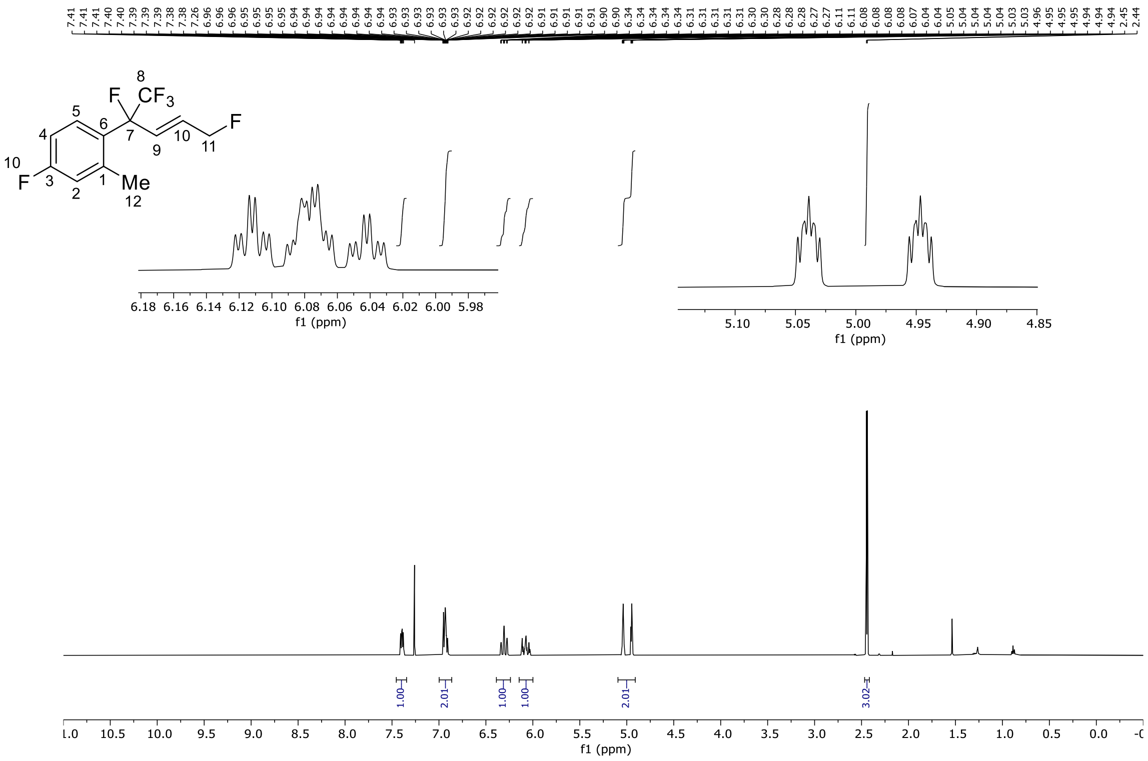

^{13}C NMR (126 MHz, $CDCl_3$)

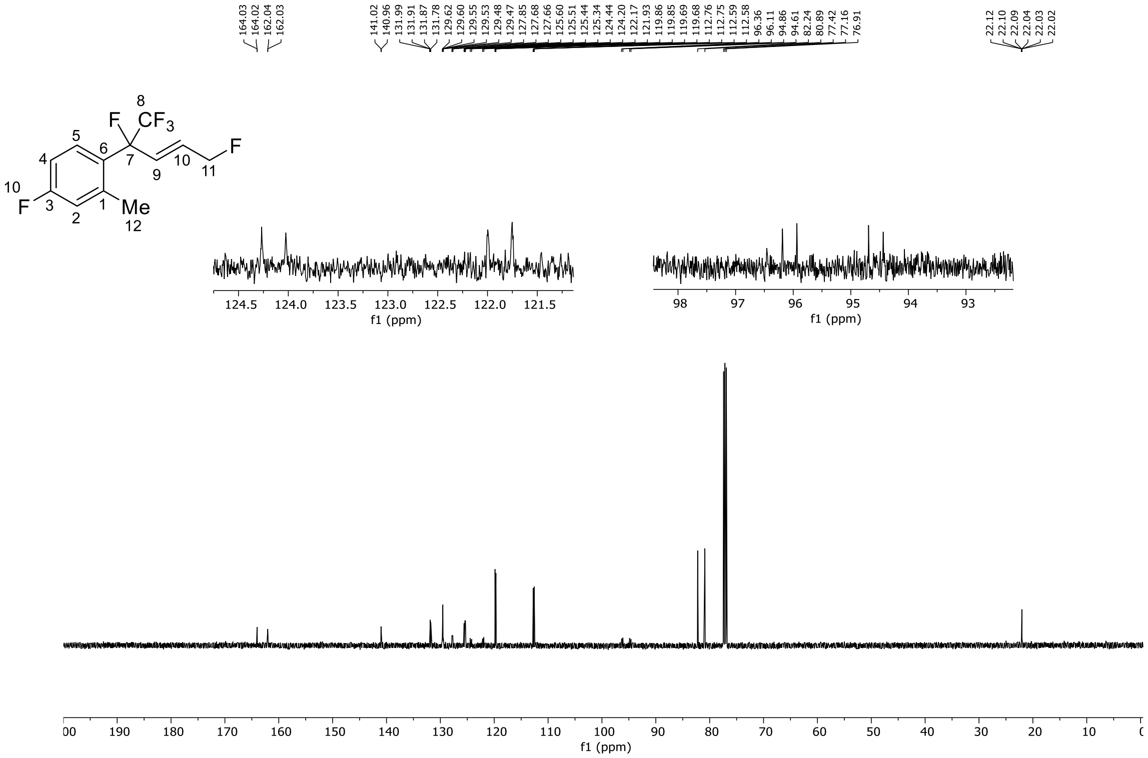

^{19}F NMR (470 MHz, $CDCl_3$)

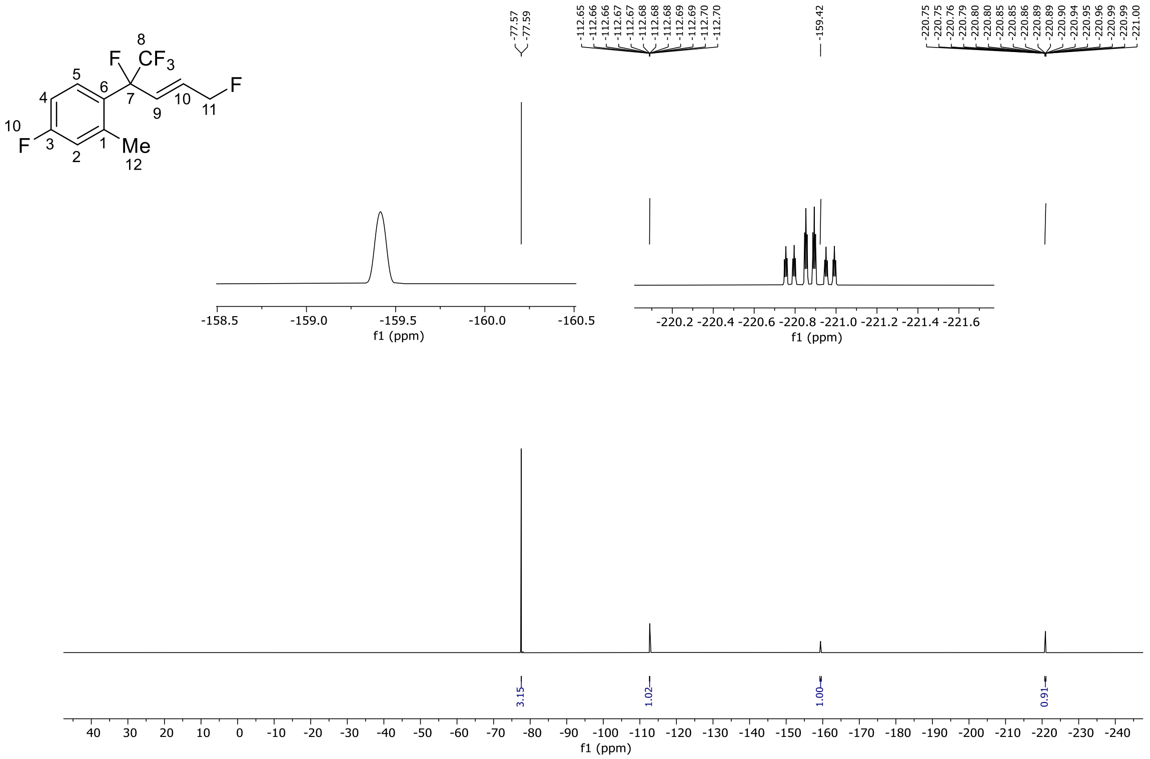

^{19}F{^{1}H} NMR (470 MHz, $CDCl_3$)

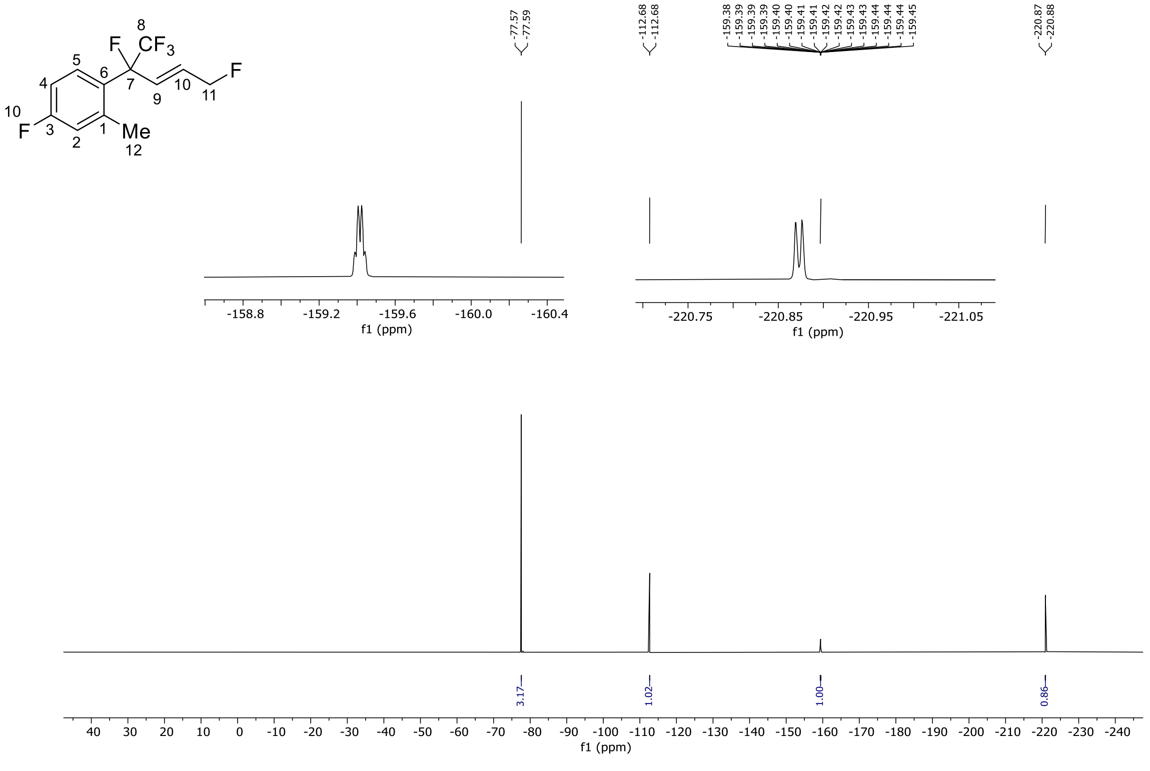

(*E*)-1-(1,1,1,2,5-Pentafluoropent-3-en-2-yl)-4-(trifluoromethyl)benzene (2h)

^{1}H NMR (500 MHz, $CDCl_3$)

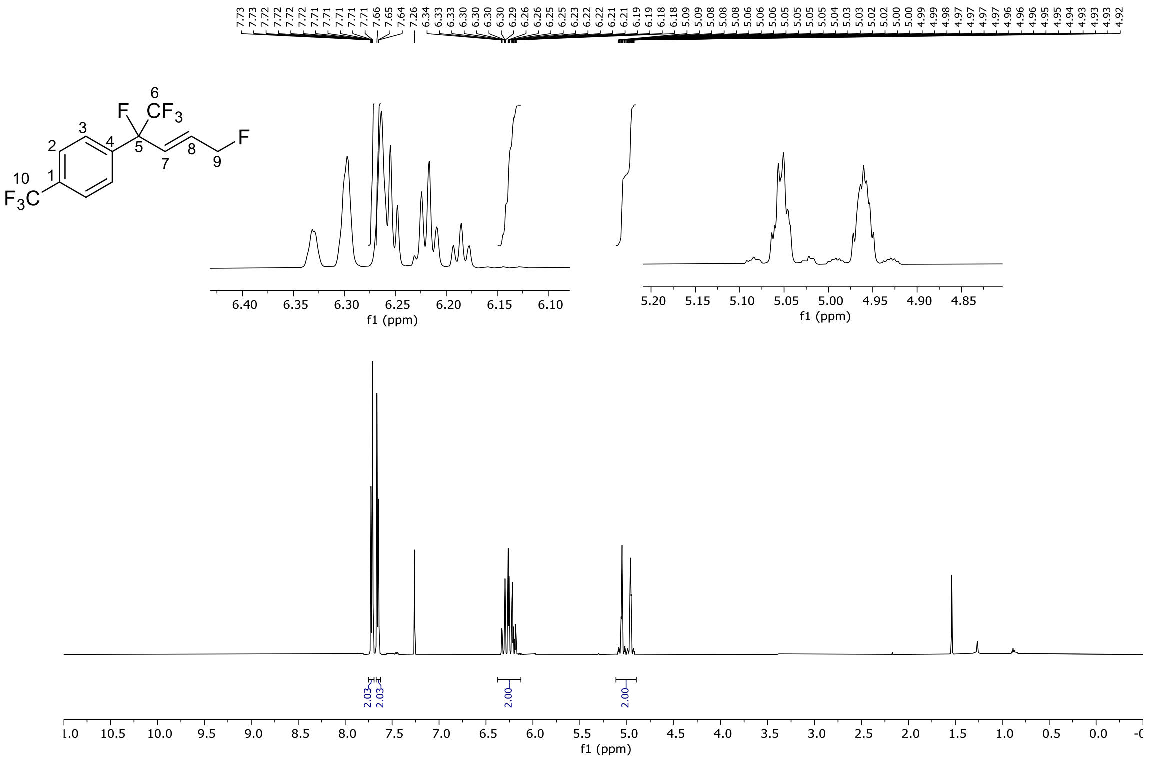

^{13}C NMR (126 MHz, $CDCl_3$)

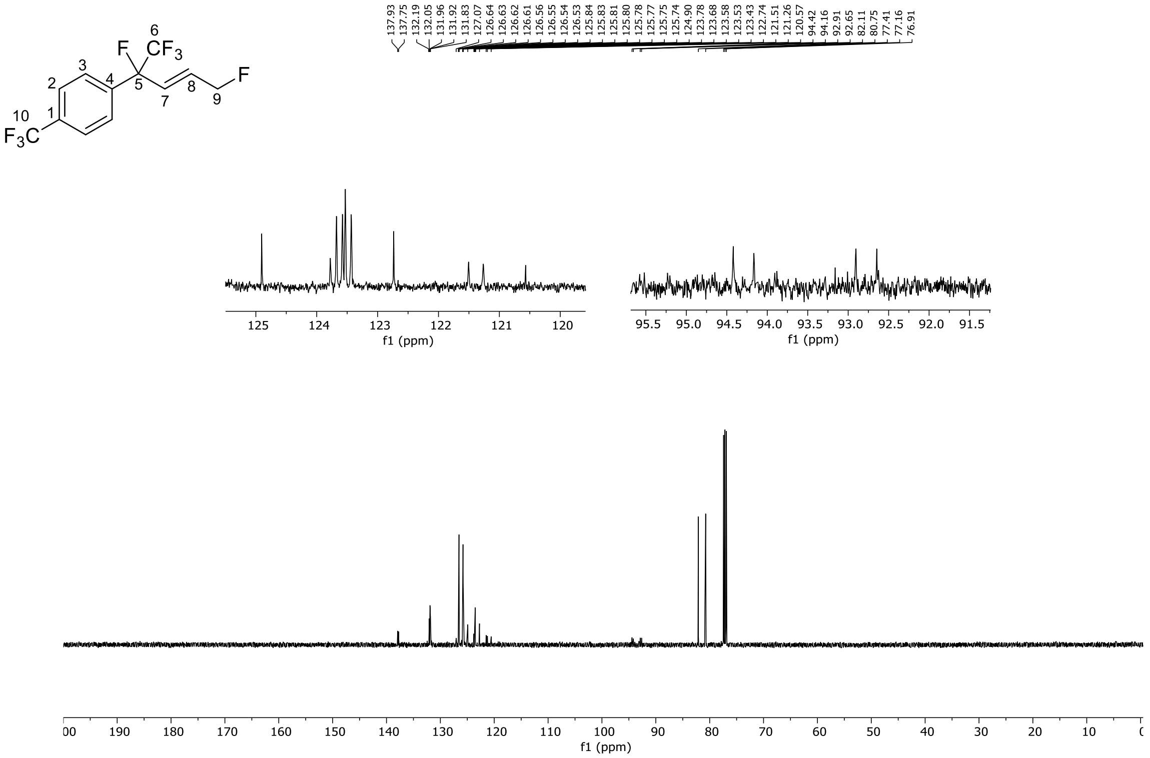

^{19}F NMR (470 MHz, $CDCl_3$)

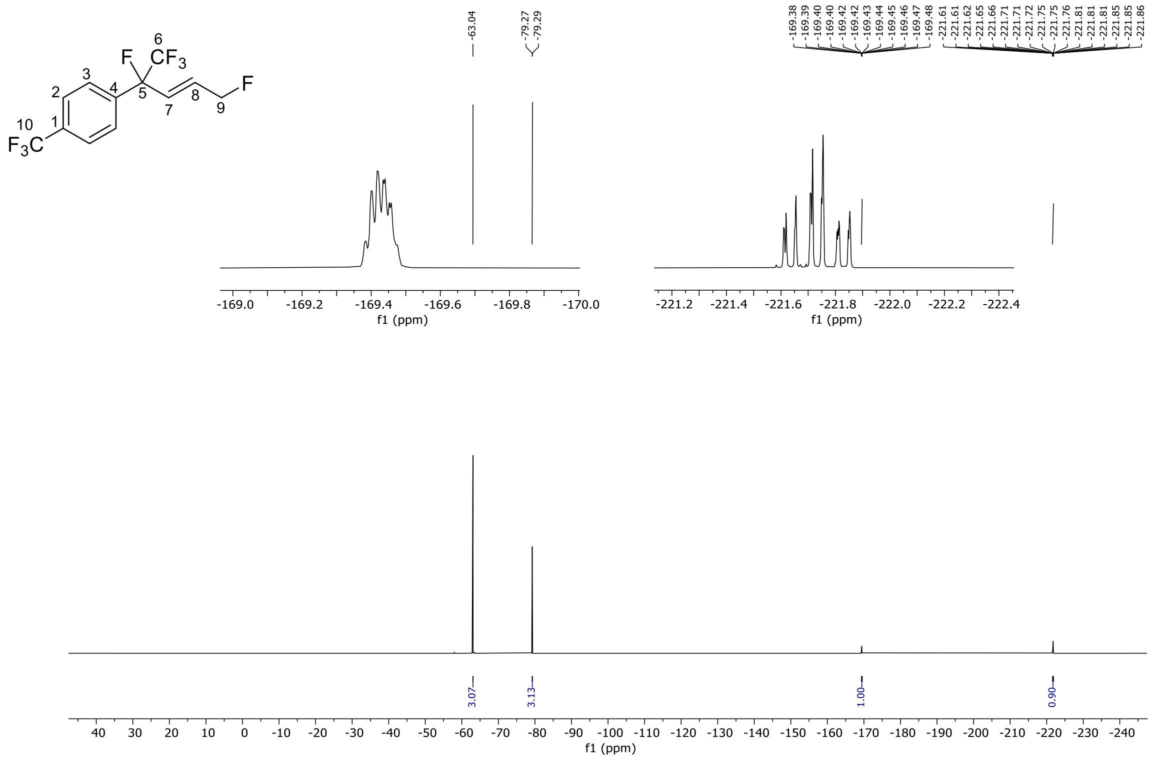

^{19}F{^{1}H} NMR (470 MHz, $CDCl_3$)

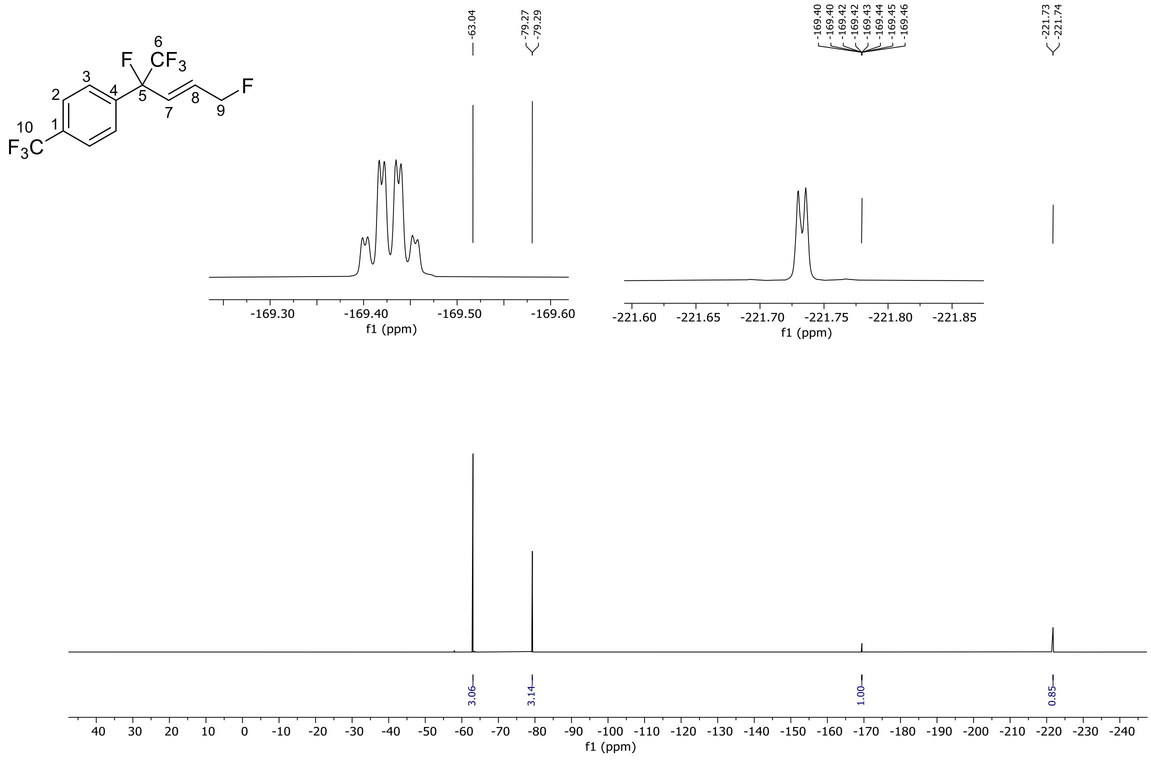

(*E*)-1-Methyl-4-(1,1,1,2,5-pentafluoropent-3-en-2-yl)benzene (2i)

^{1}H NMR (500 MHz, $CDCl_3$)

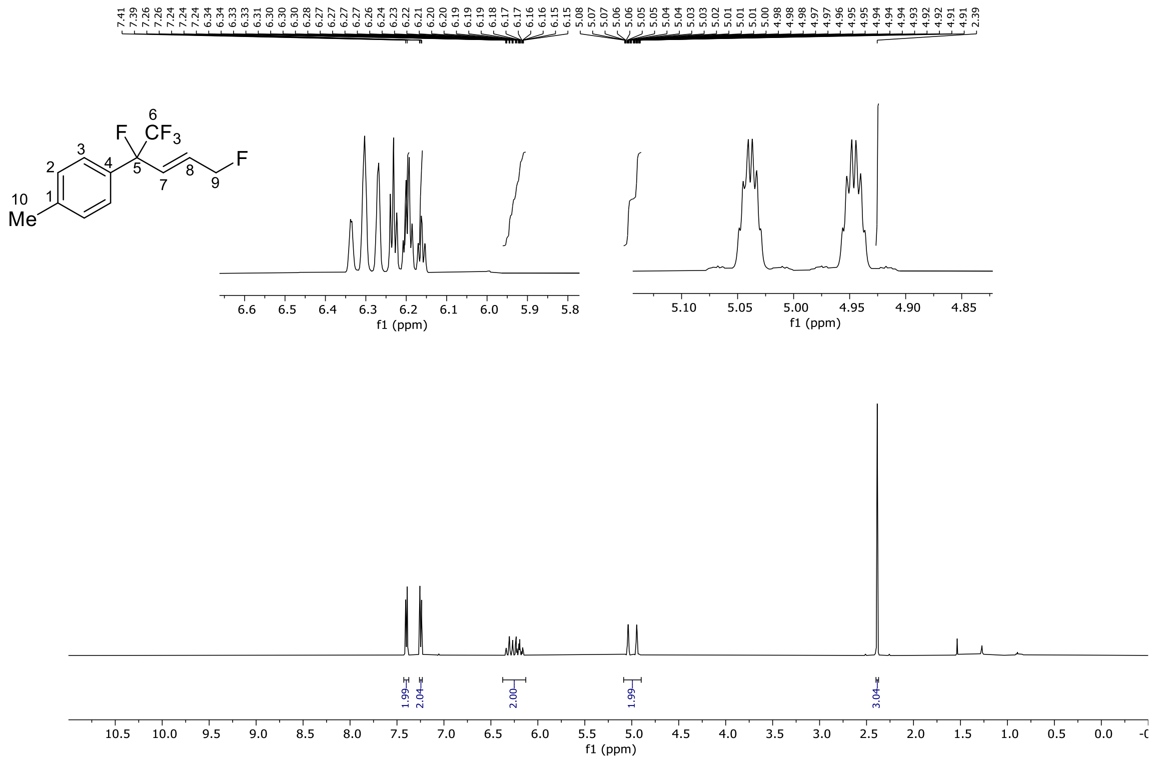

^{13}C NMR (126 MHz, $CDCl_3$)

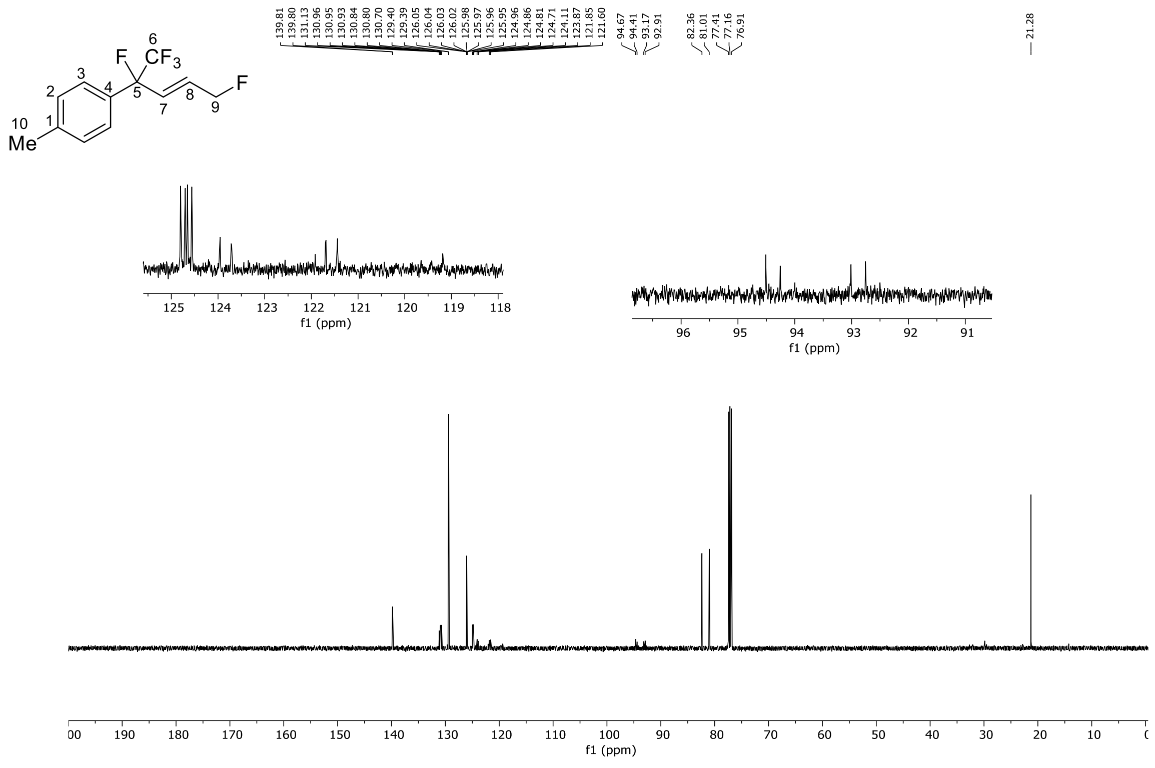

^{19}F NMR (470 MHz, $CDCl_3$)

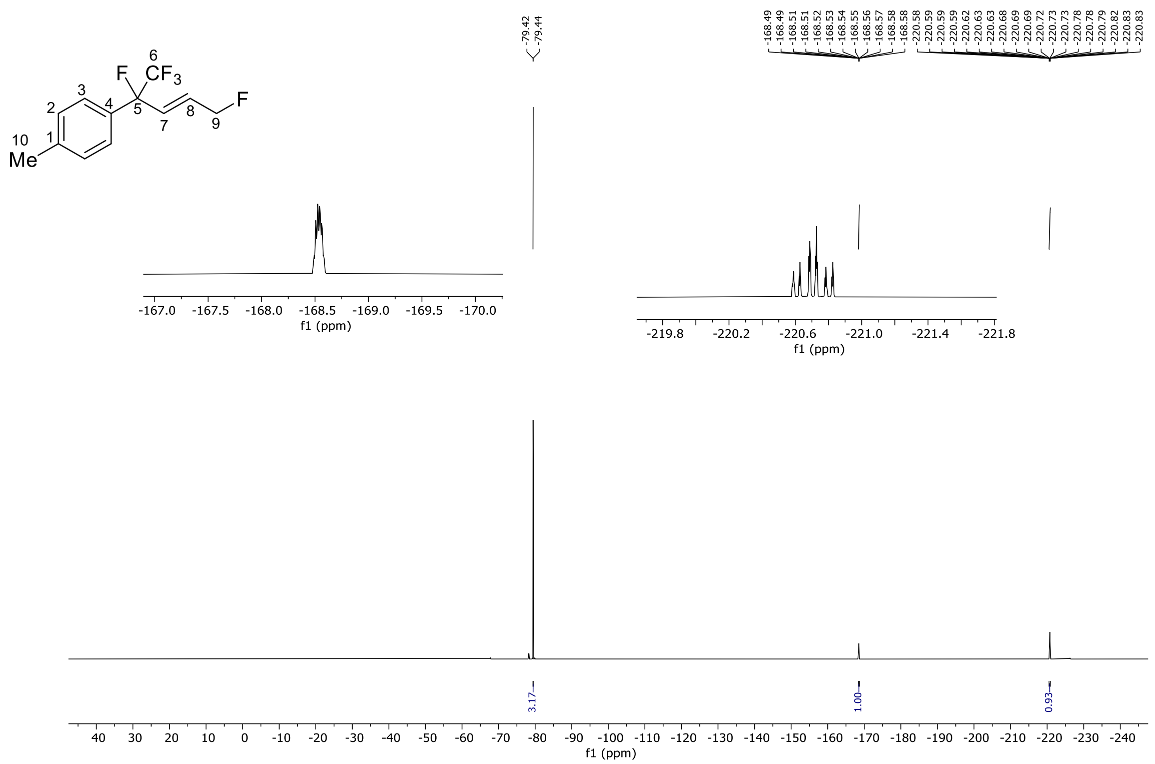

^{19}F{^{1}H} NMR (376 MHz, $CDCl_3$)

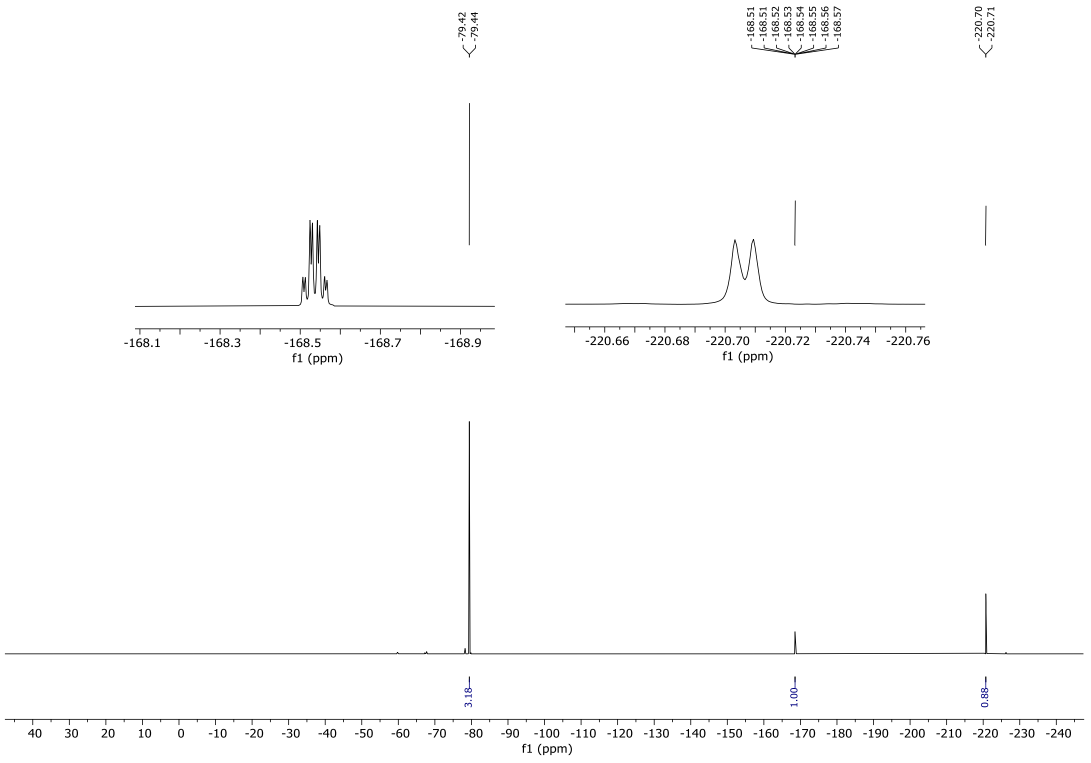

(*E*)-(1,1,1,2,5-Pentafluoropent-3-en-2-yl)benzene (2j)

^{1}H NMR (599 MHz, $CDCl_3$)

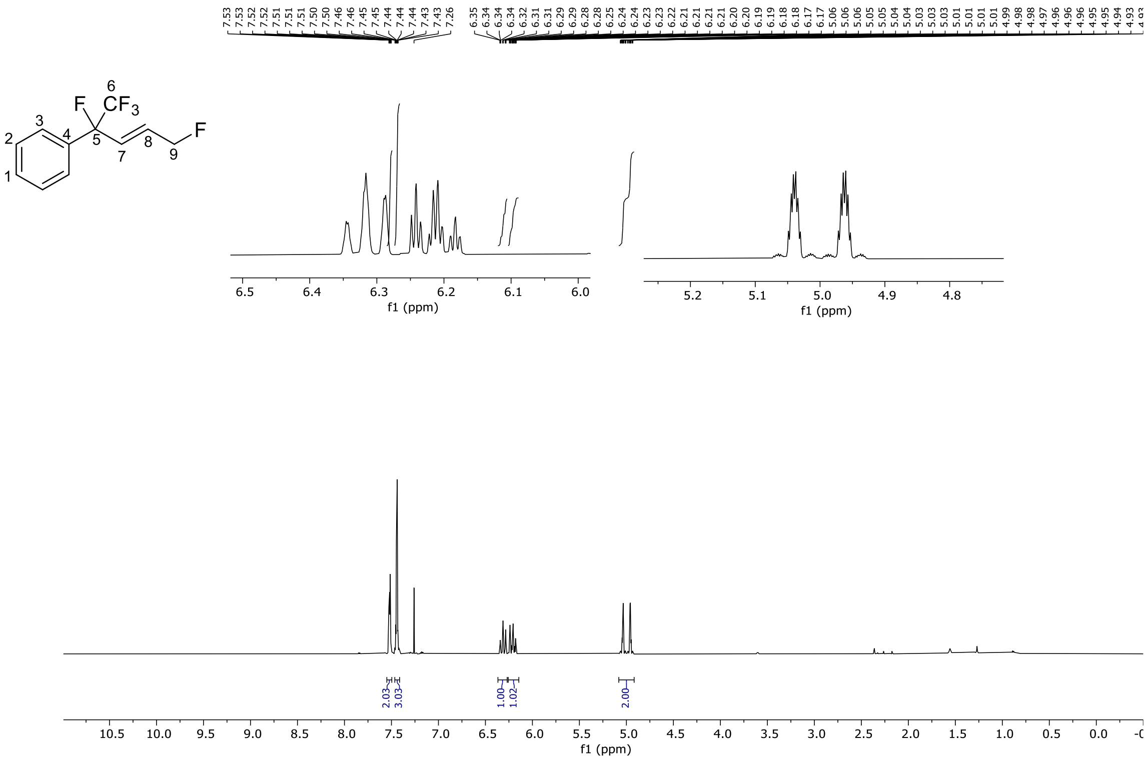

^{13}C NMR (151 MHz, $CDCl_3$)

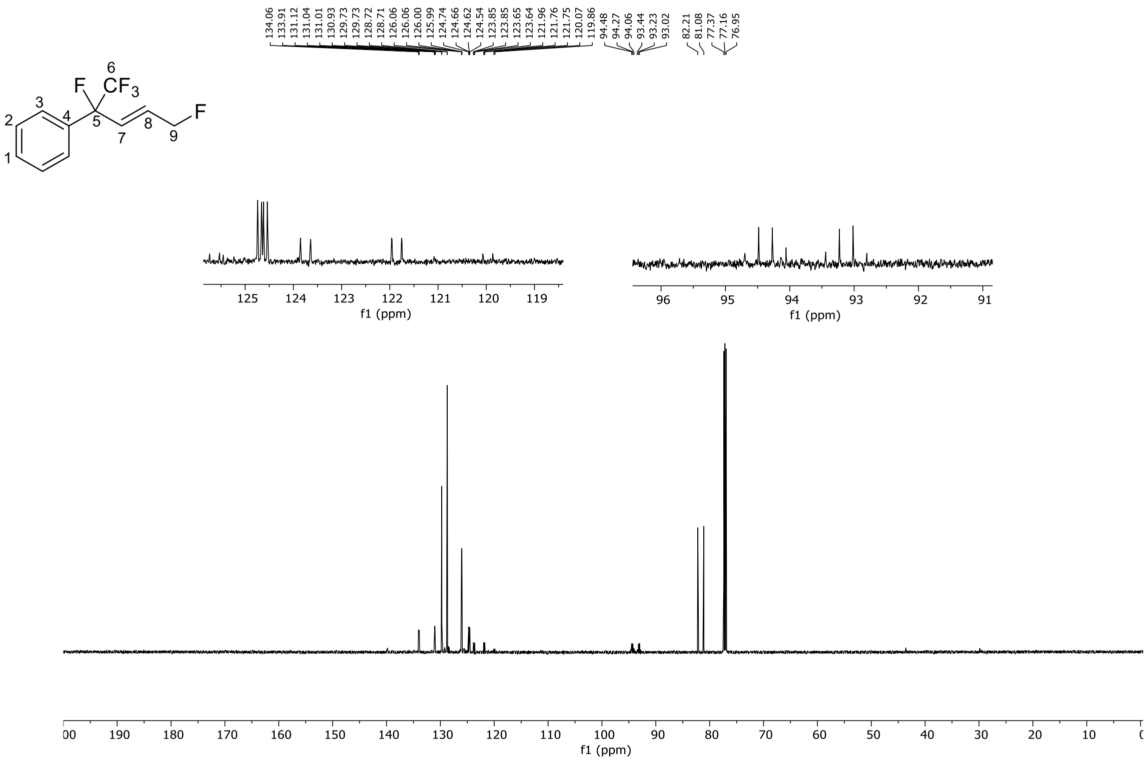

^{19}F NMR (564 MHz, $CDCl_3$)

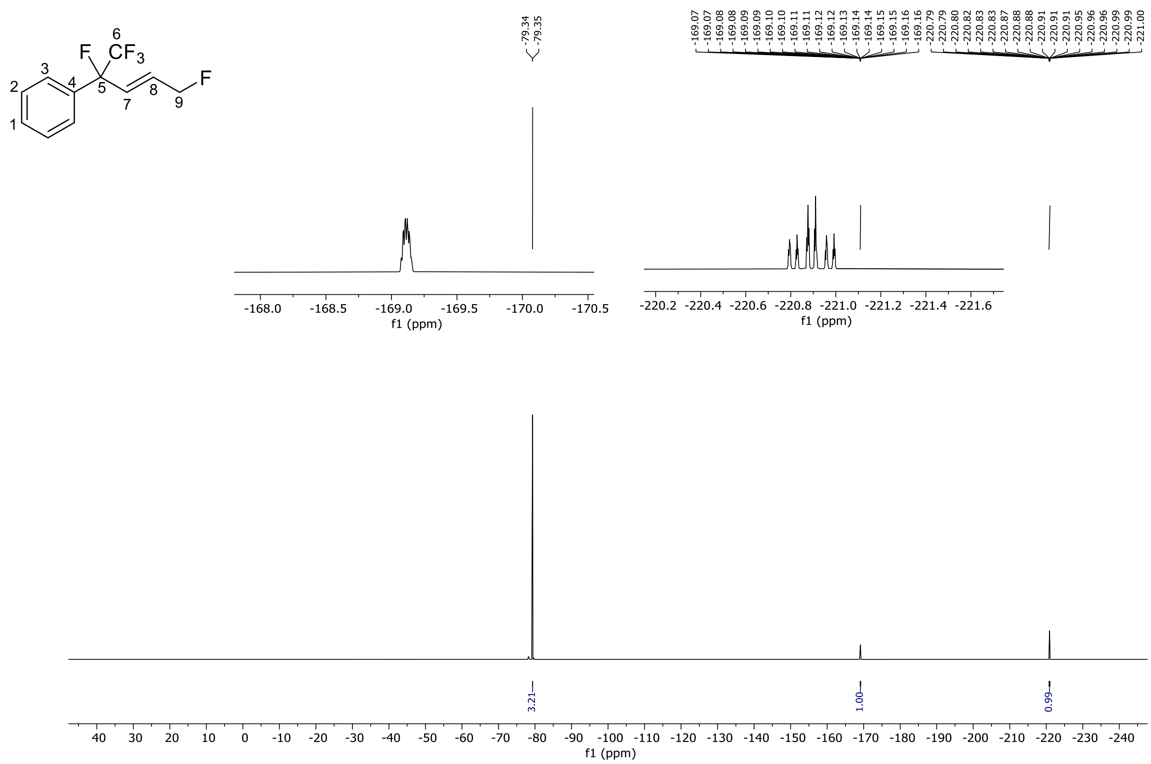

^{19}F{^{1}H} NMR (376 MHz, $CDCl_3$)

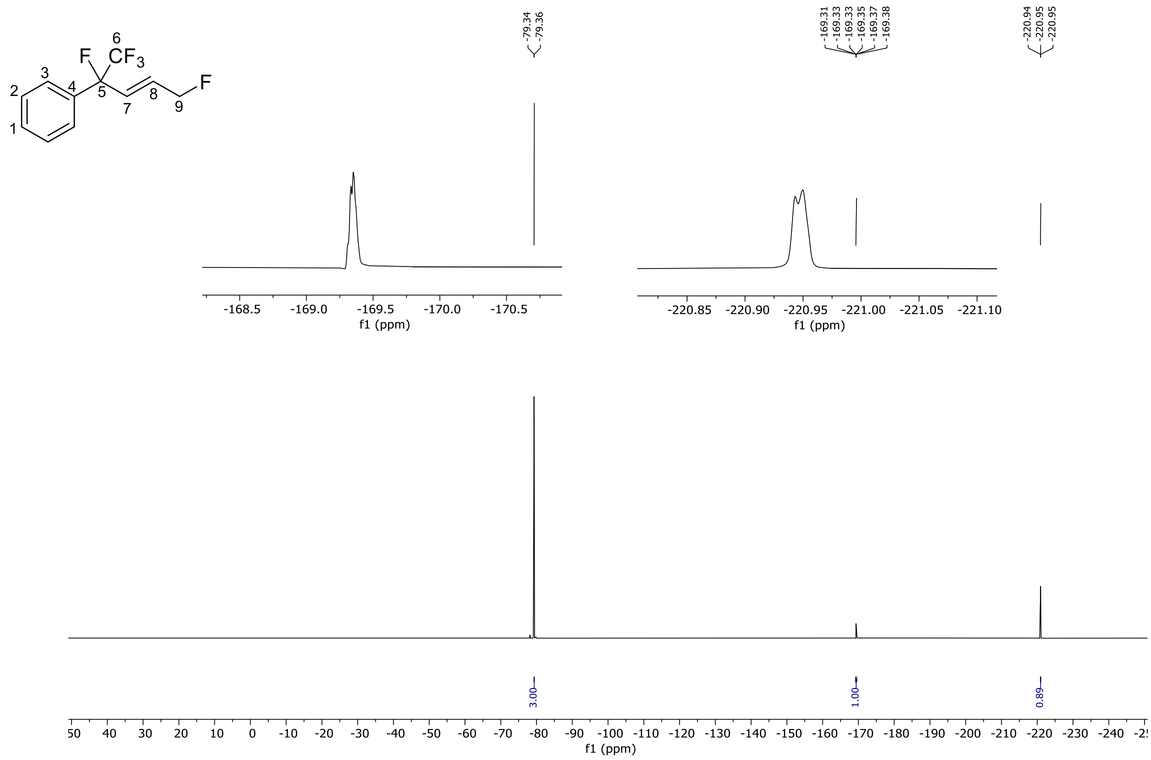

(*E*)-1-Nitro-4-(1,1,1,2,5-pentafluoropent-3-en-2-yl)benzene (2k)

^{1}H NMR (500 MHz, $CDCl_3$)

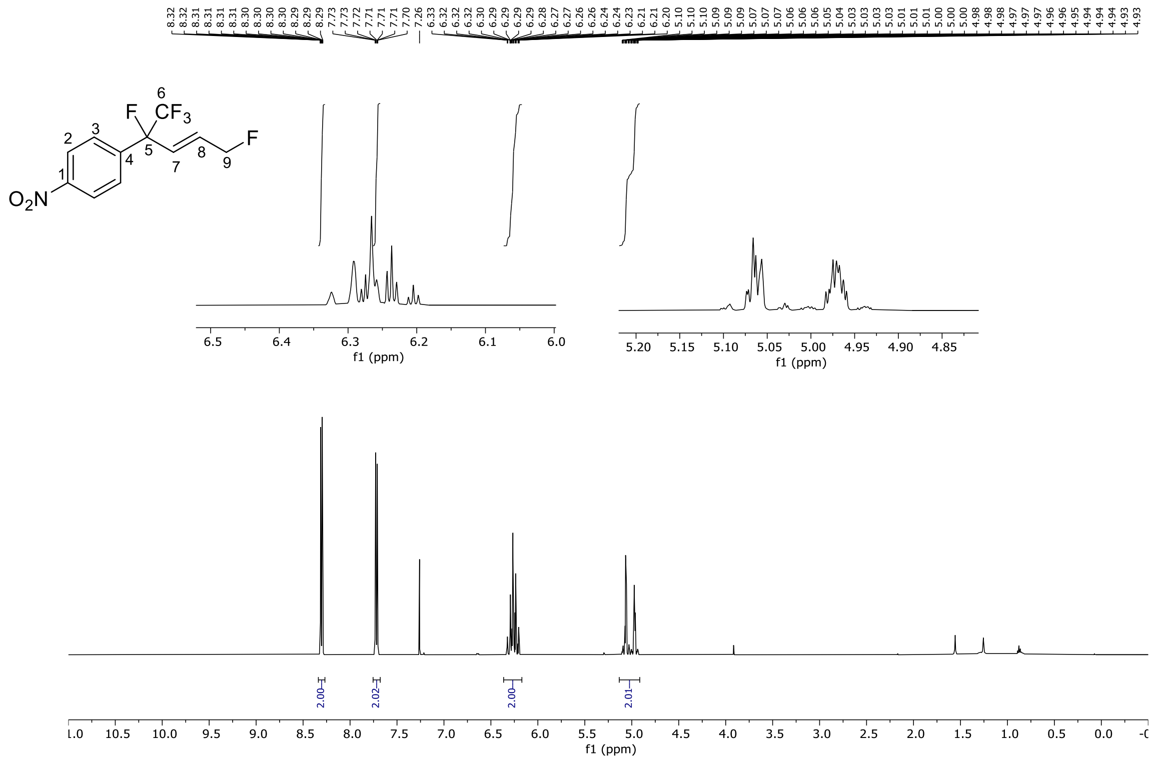

^{13}C NMR (126 MHz, $CDCl_3$)

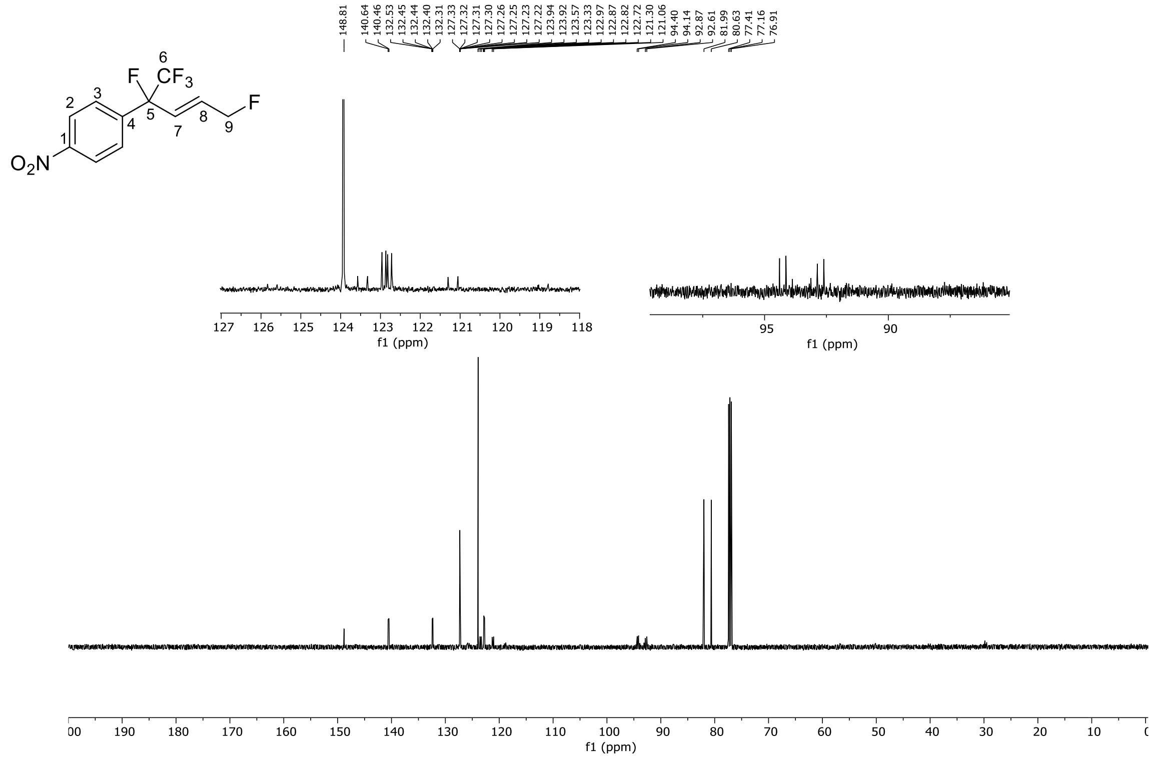

^{19}F NMR (470 MHz, $CDCl_3$)

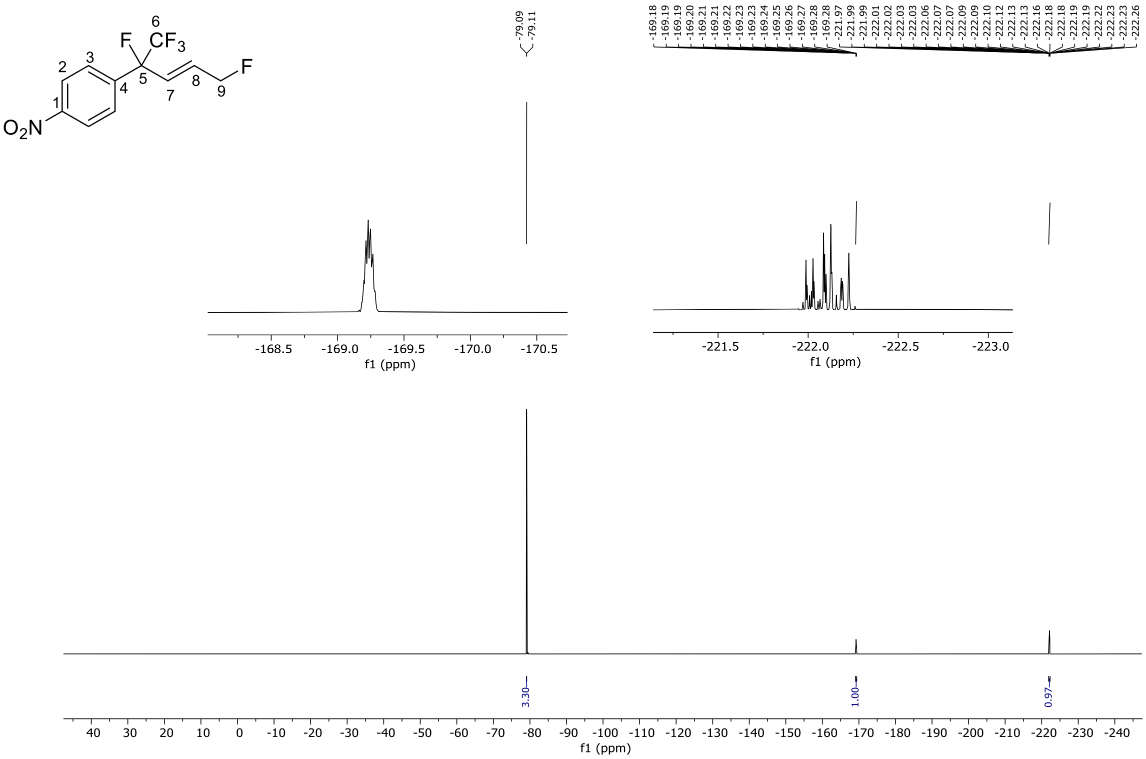

^{19}F{^{1}H} NMR (470 MHz, $CDCl_3$)

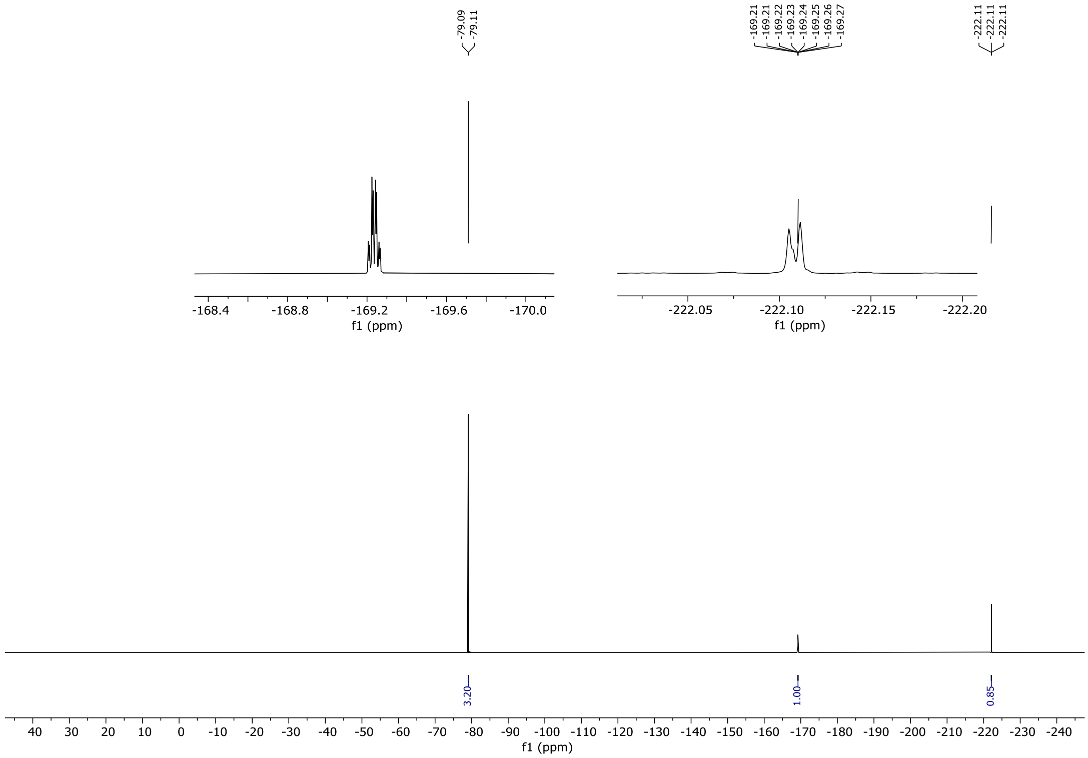

(*E*)-4-(1,1,1,2,5-Pentafluoropent-3-en-2-yl)benzonitrile (2l)

^{1}H NMR (500 MHz, $CDCl_3$)

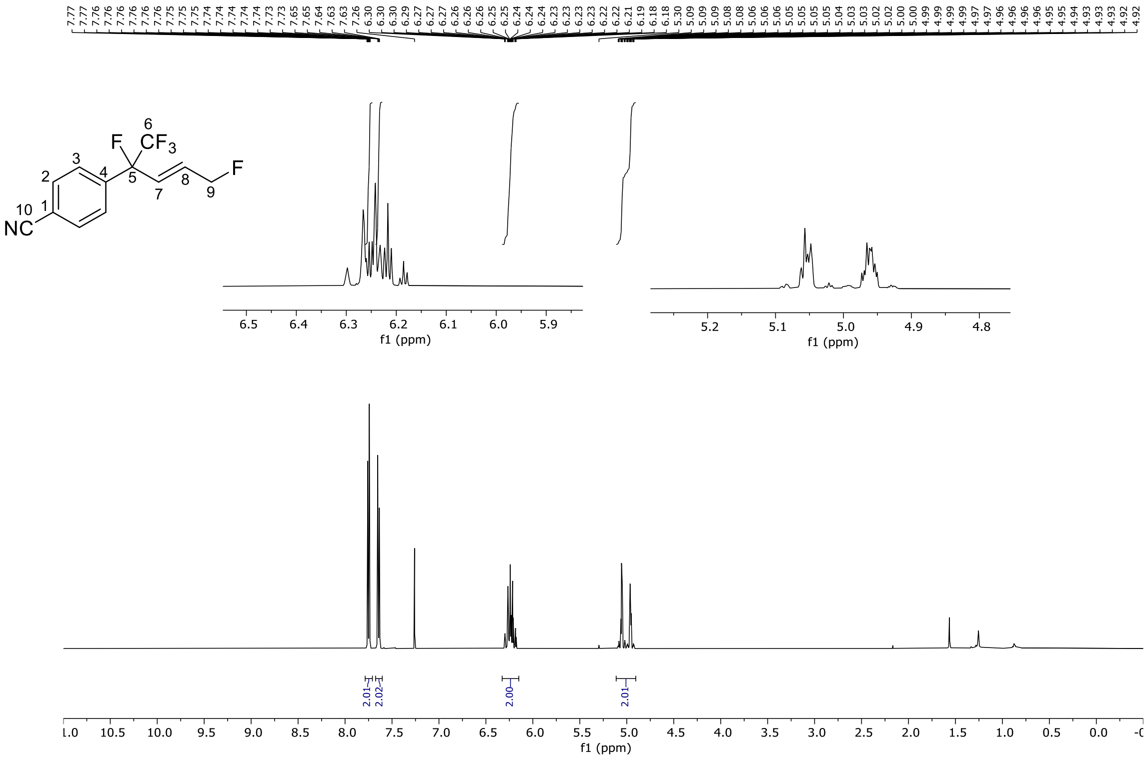

^{13}C NMR (126 MHz, $CDCl_3$)

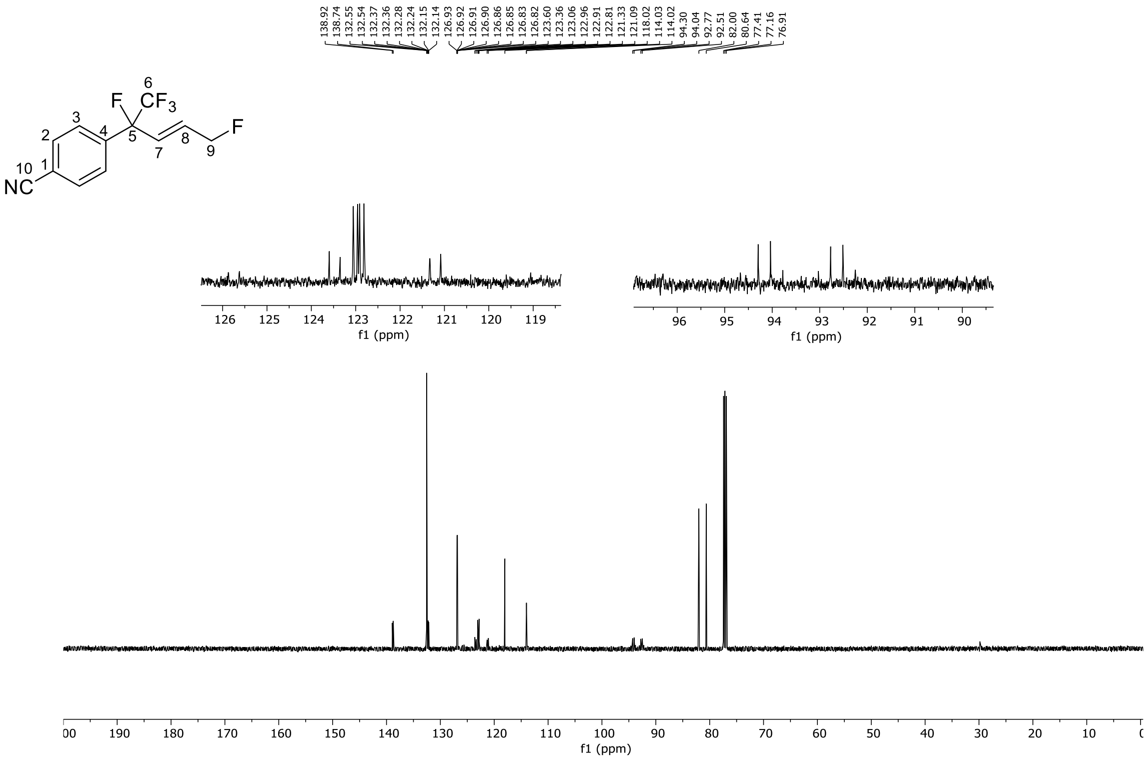

^{19}F NMR (470 MHz, $CDCl_3$)

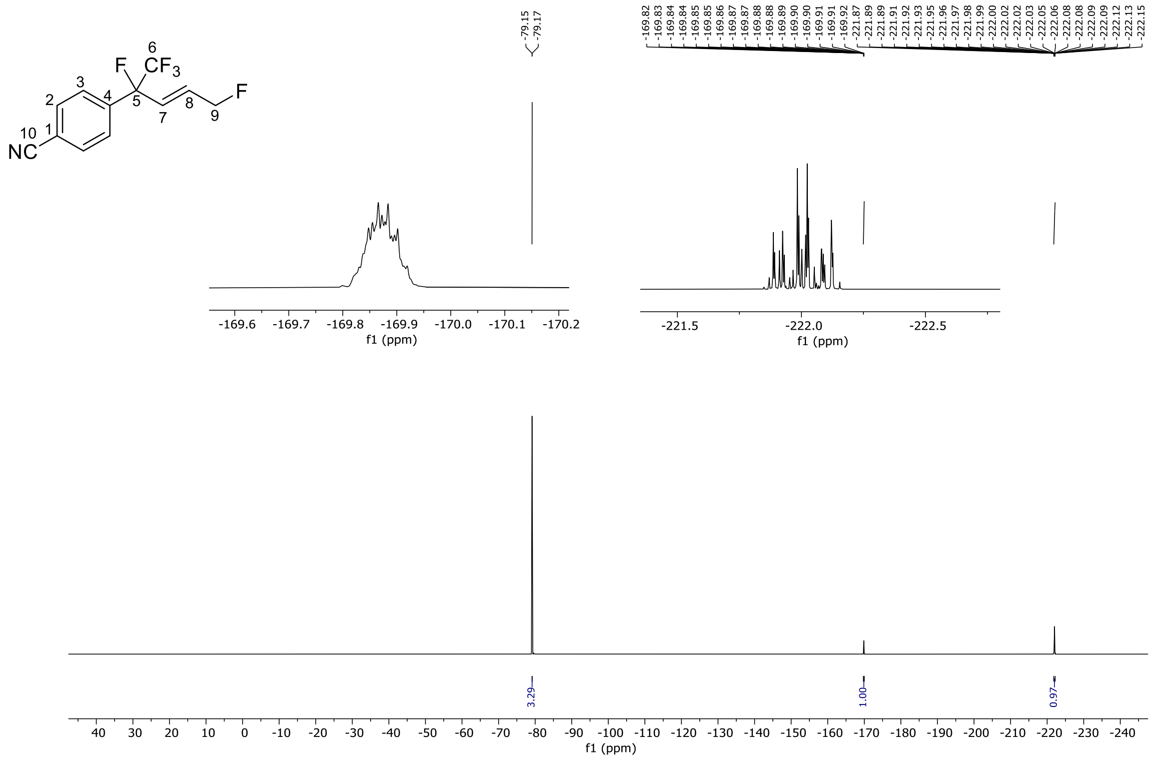

^{19}F{^{1}H} NMR (376 MHz, $CDCl_3$)

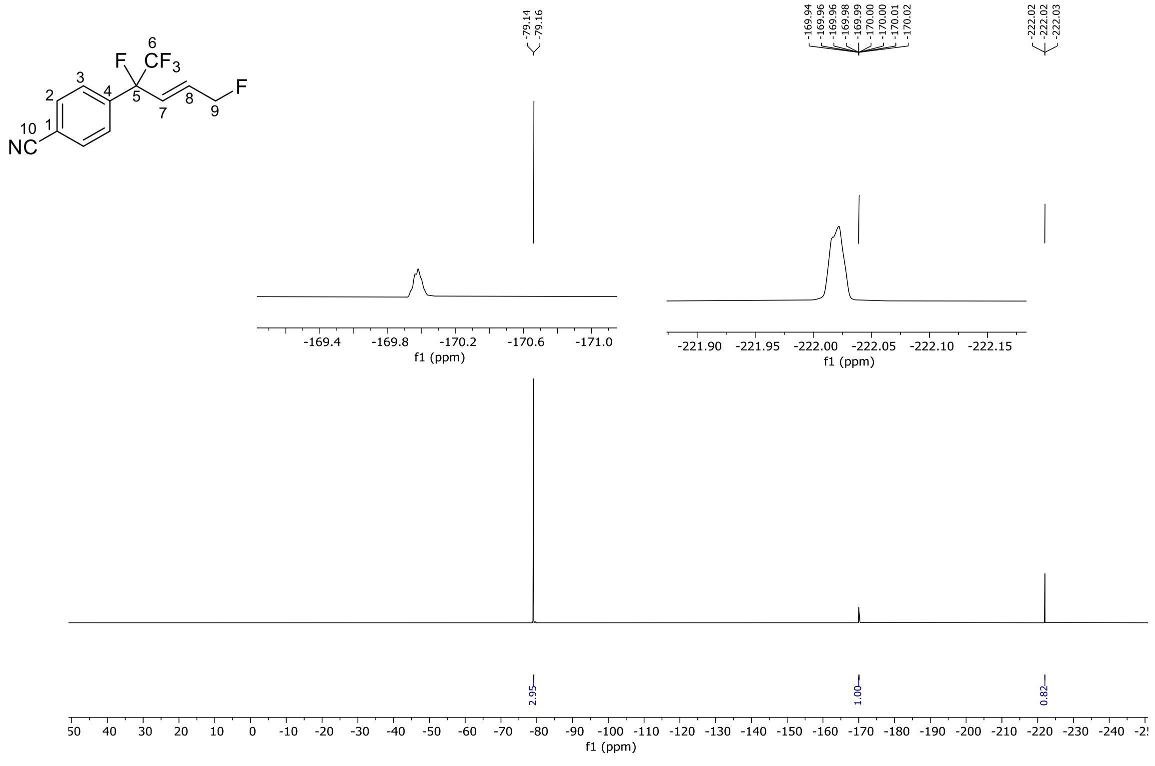

Methyl (*E*)-4-(1,1,1,2,5-pentafluoropent-3-en-2-yl)benzoate (2m)

^{1}H NMR (500 MHz, $CDCl_3$)

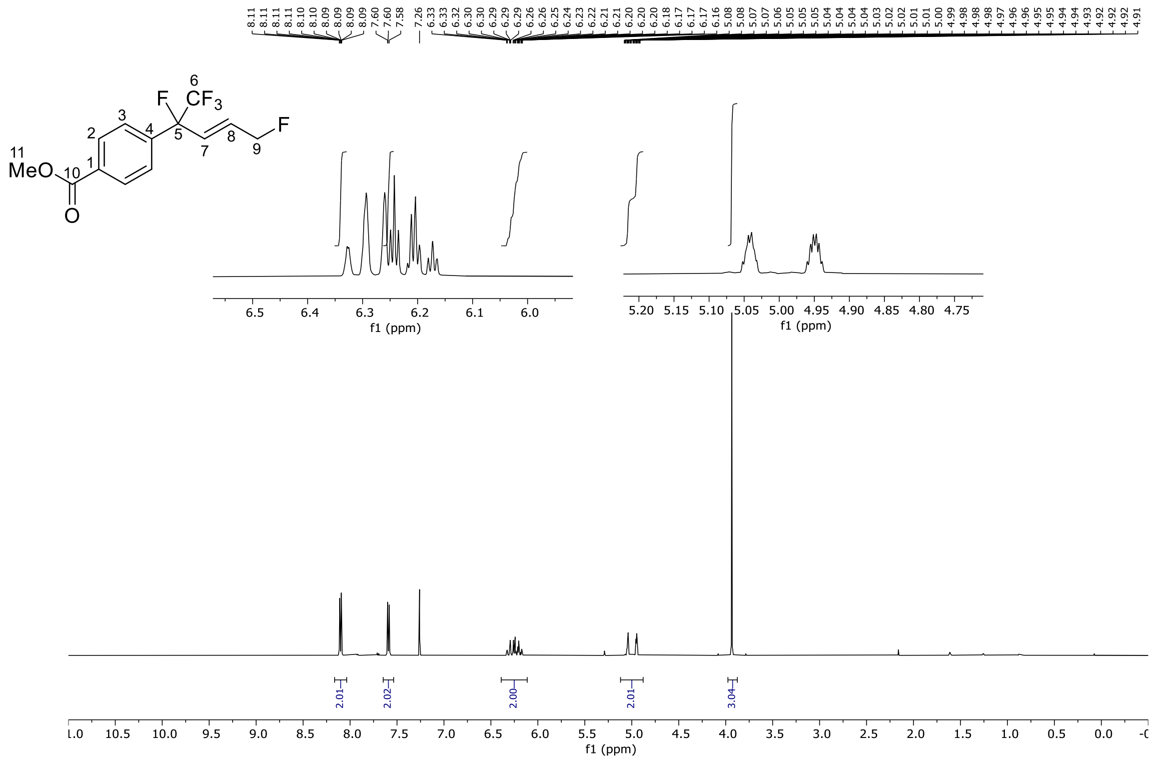

^{13}C NMR (126 MHz, $CDCl_3$)

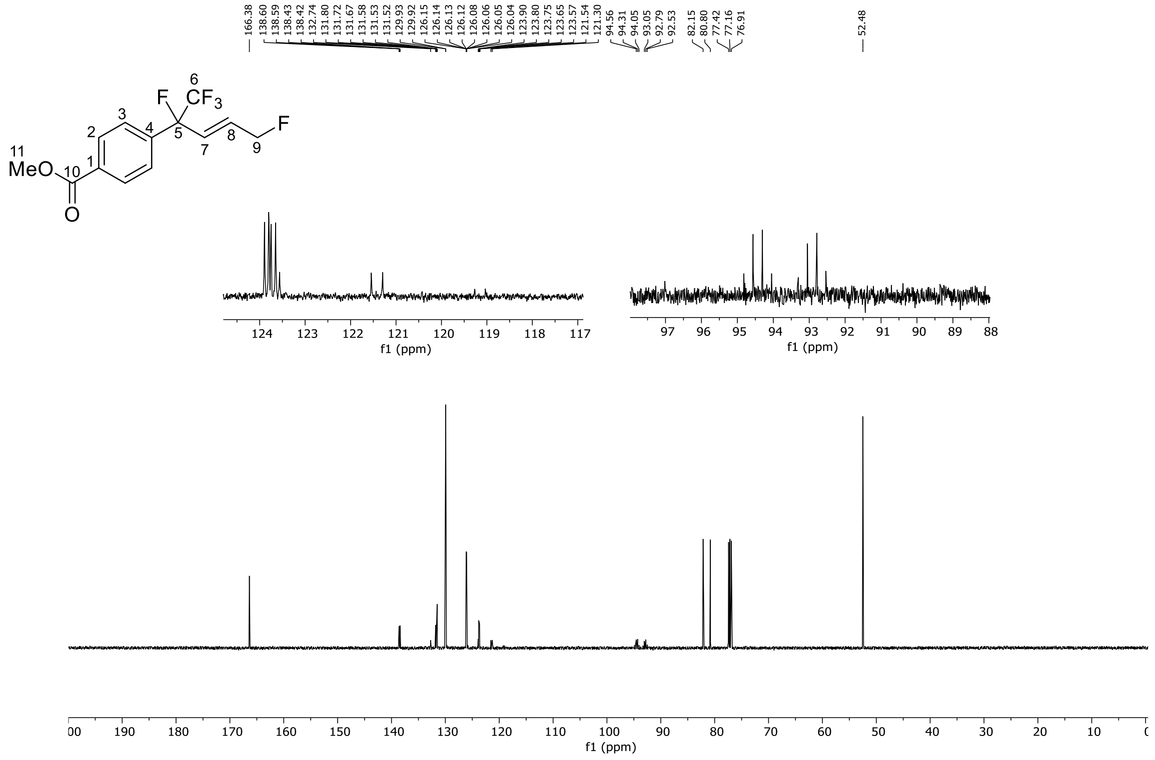

^{19}**F NMR** (470 MHz, $CDCl_3$)

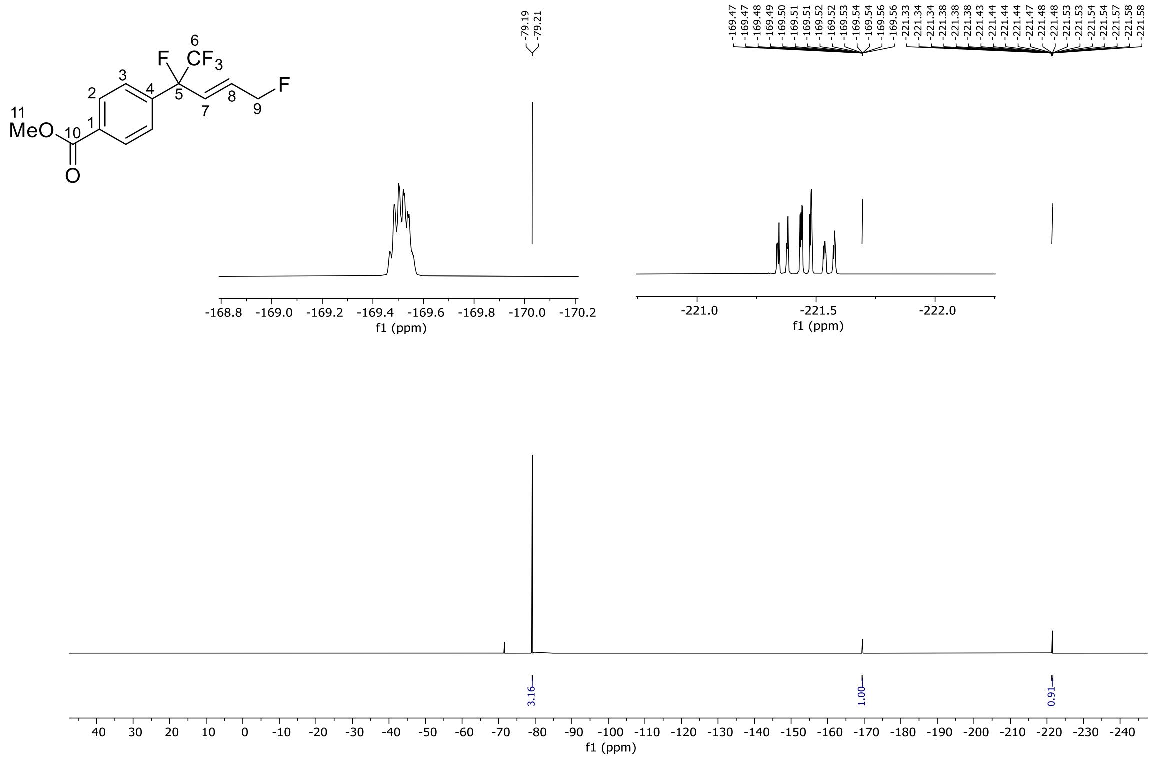

^{19}**F{^{1}H} NMR** (376 MHz, $CDCl_3$)

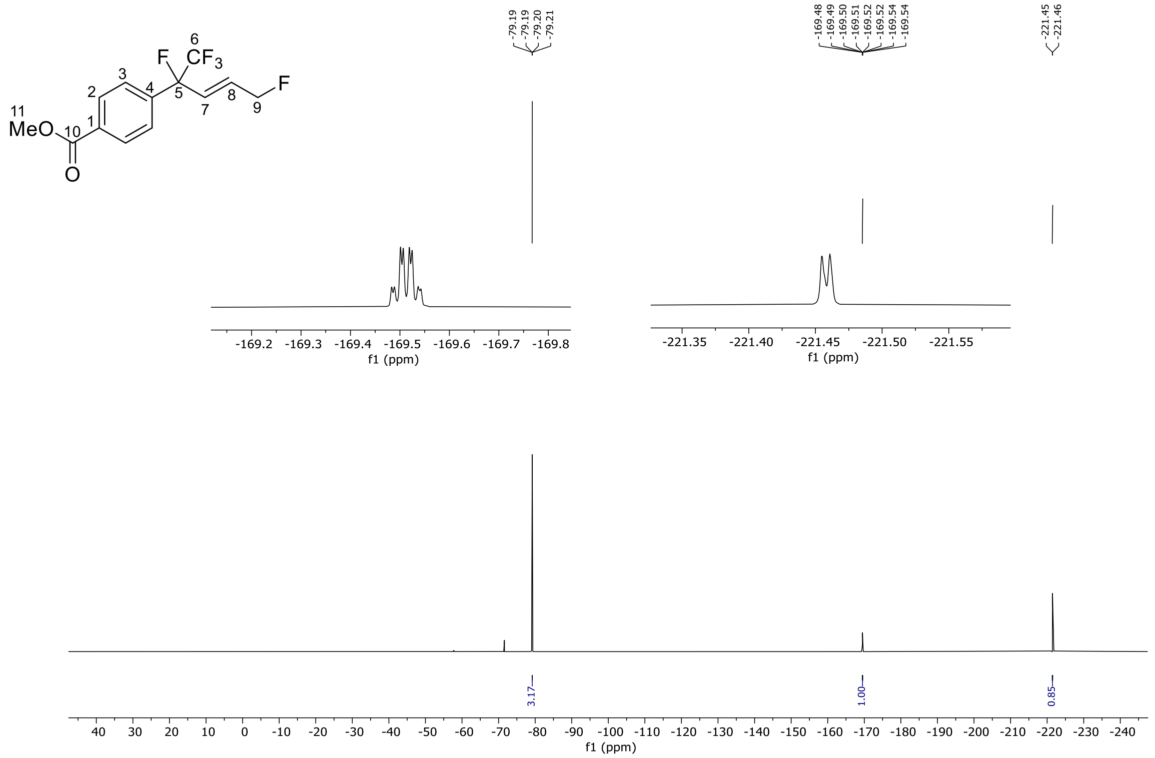

(*E*)-1-(Methylsulfonyl)-4-(1,1,1,2,5-pentafluoropent-3-en-2-yl)benzene (2n)

^{1}H NMR (500 MHz, $CDCl_3$)

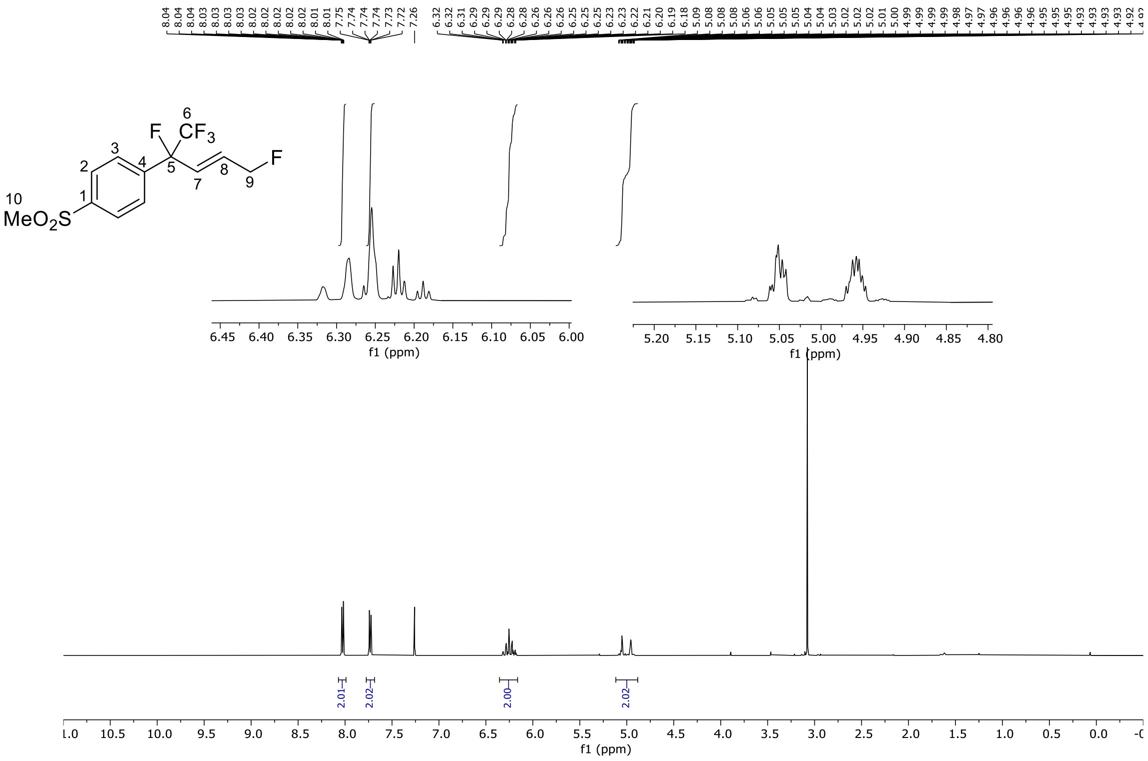

^{13}C NMR (126 MHz, $CDCl_3$)

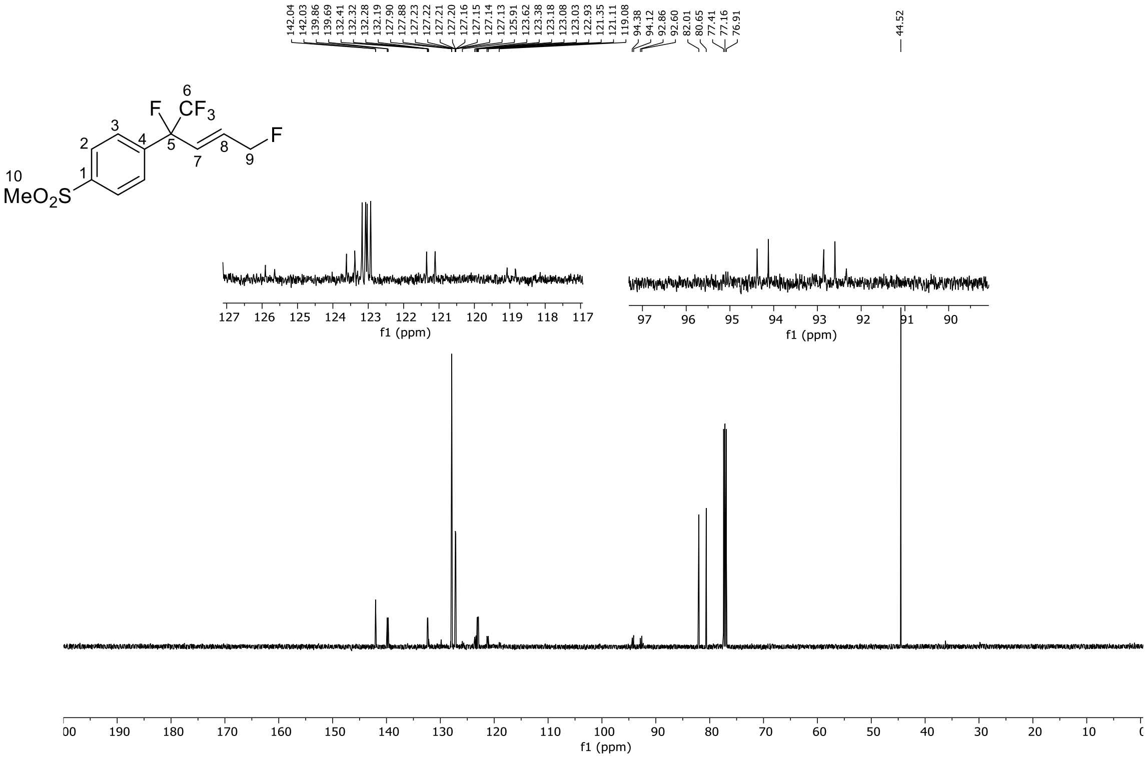

^{19}F NMR (470 MHz, $CDCl_3$)

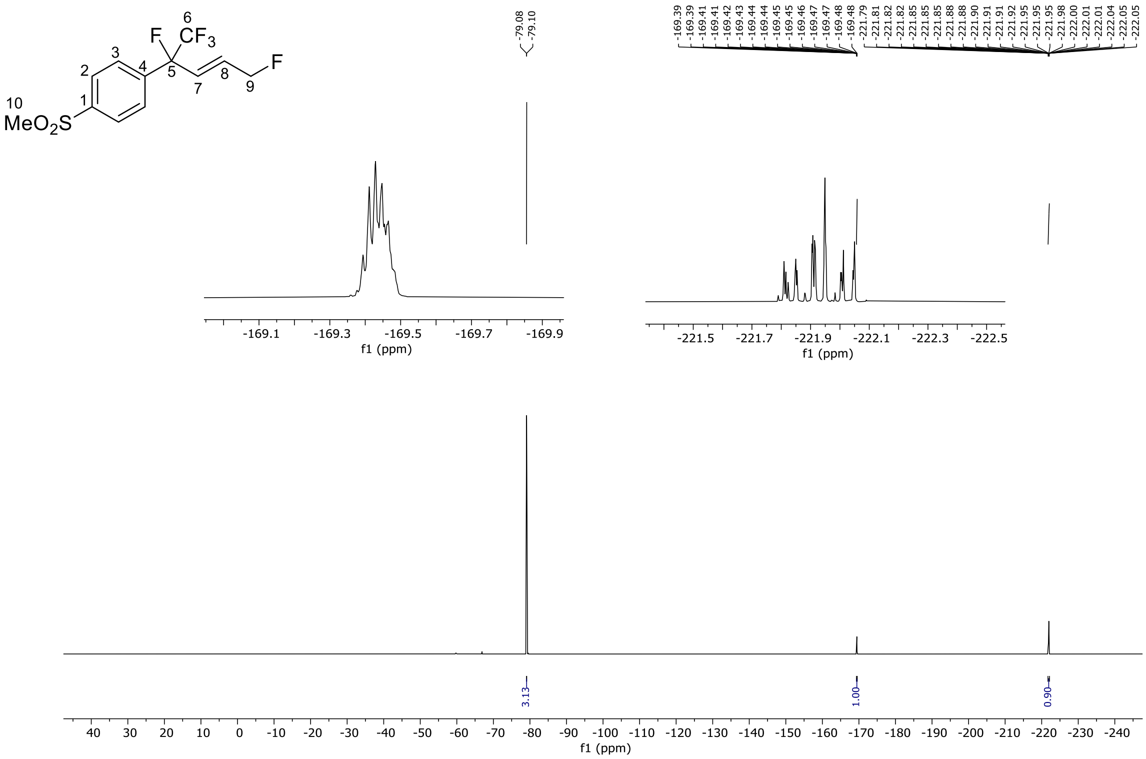

^{19}F{^{1}H} NMR (376 MHz, $CDCl_3$)

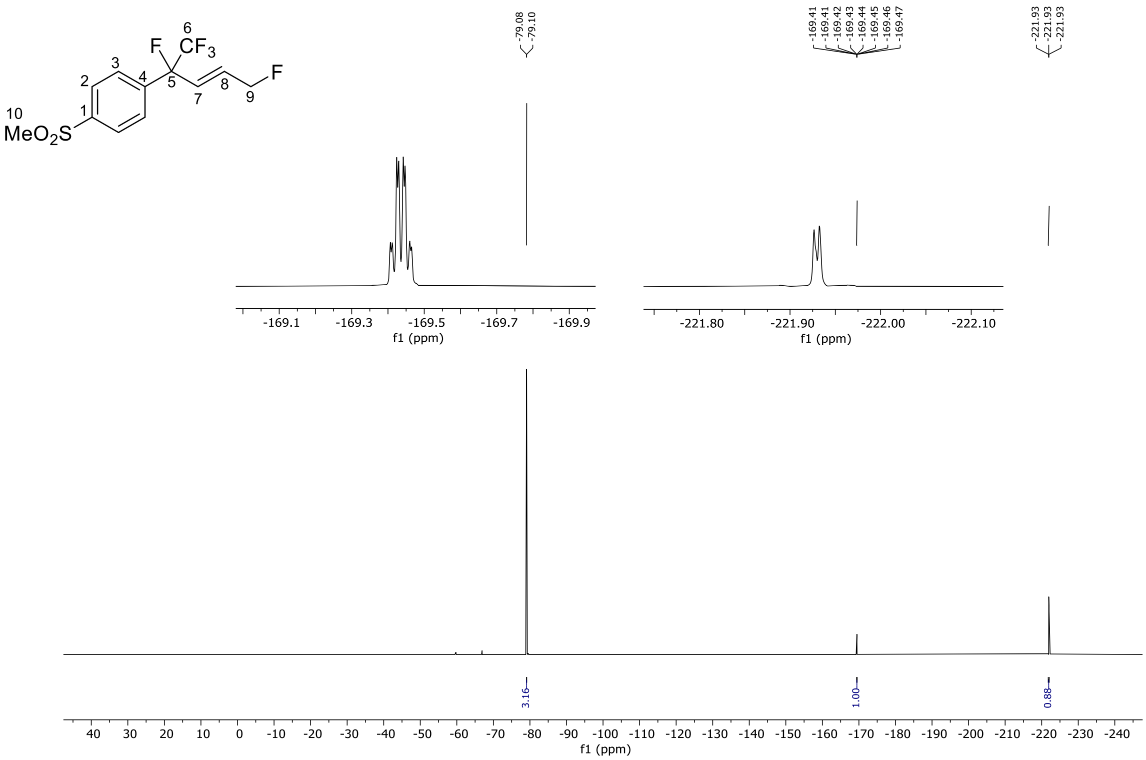

(*E*)-3-(1,1,1,2,5-Pentafluoropent-3-en-2-yl)phenyl 4-methylbenzenesulfonate (2o)

^{1}H NMR (500 MHz, $CDCl_3$)

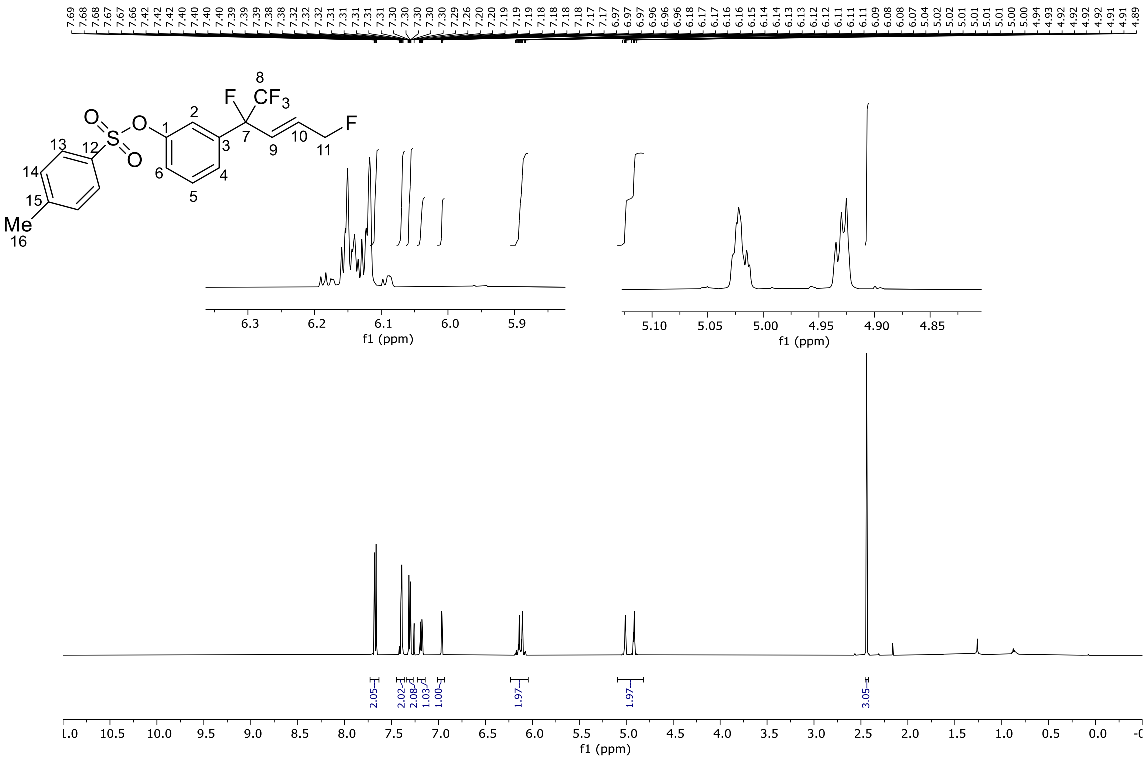

^{13}C NMR (126 MHz, $CDCl_3$)

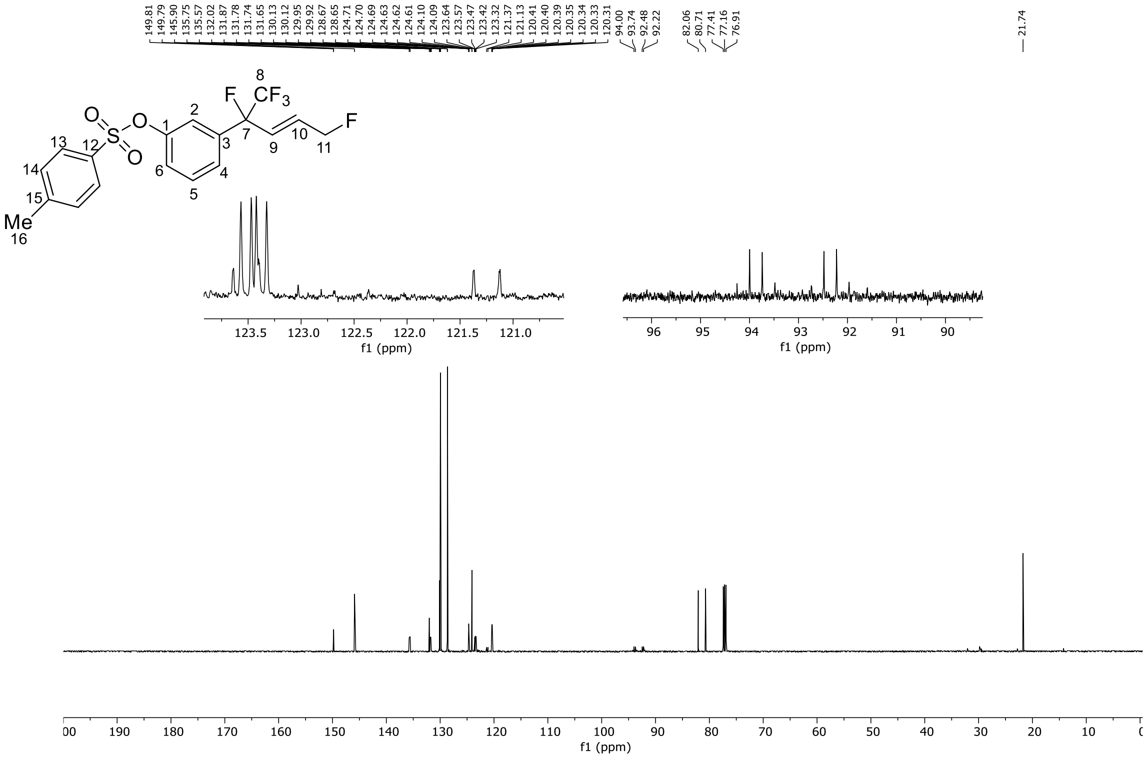

^{19}F NMR (470 MHz, $CDCl_3$)

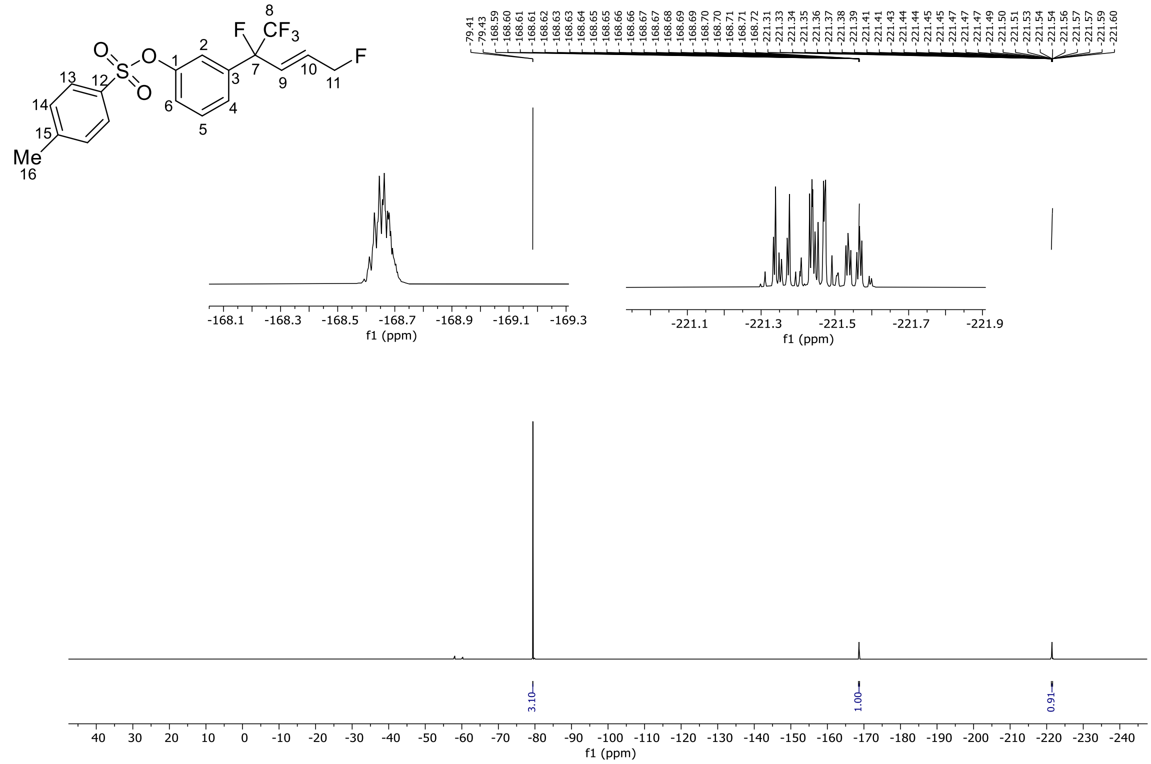

^{19}F{^{1}H} NMR (470 MHz, $CDCl_3$)

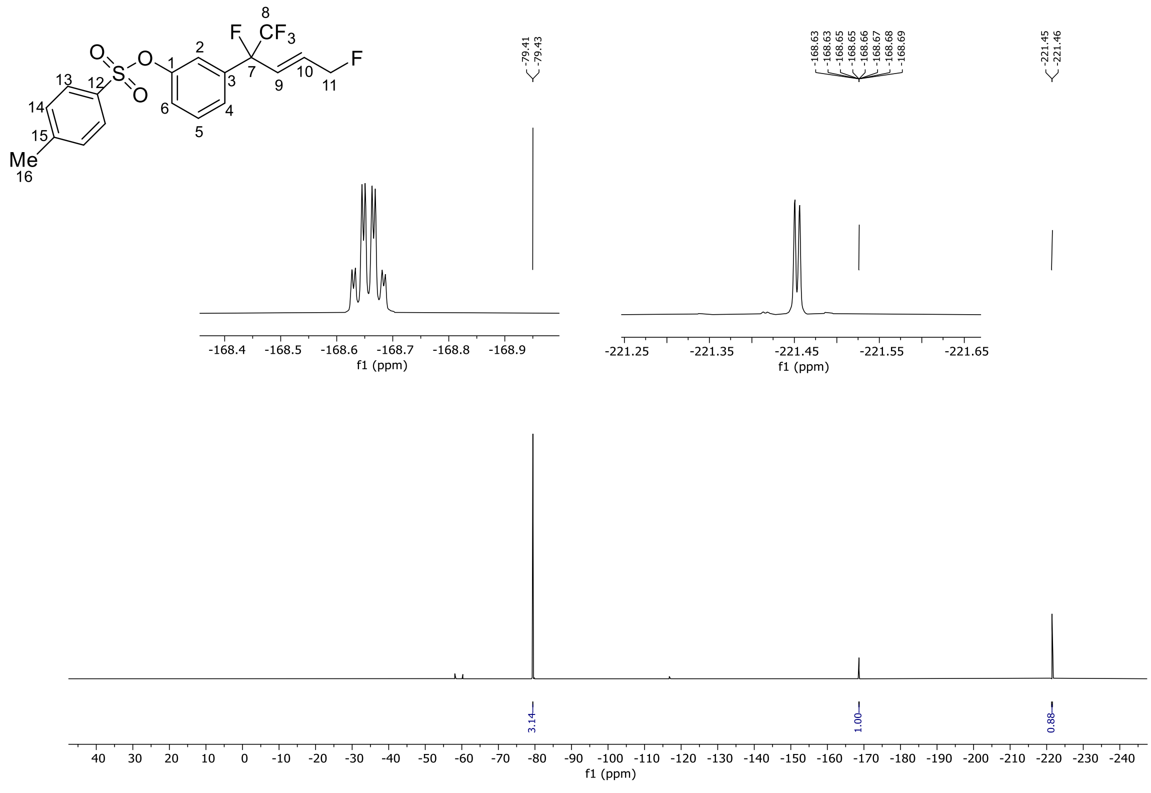

(*E*)-1-(1,1,1,2,5-Pentafluoropent-3-en-2-yl)-3-(trifluoromethoxy)benzene (2p)

^{1}H NMR (500 MHz, $CDCl_3$)

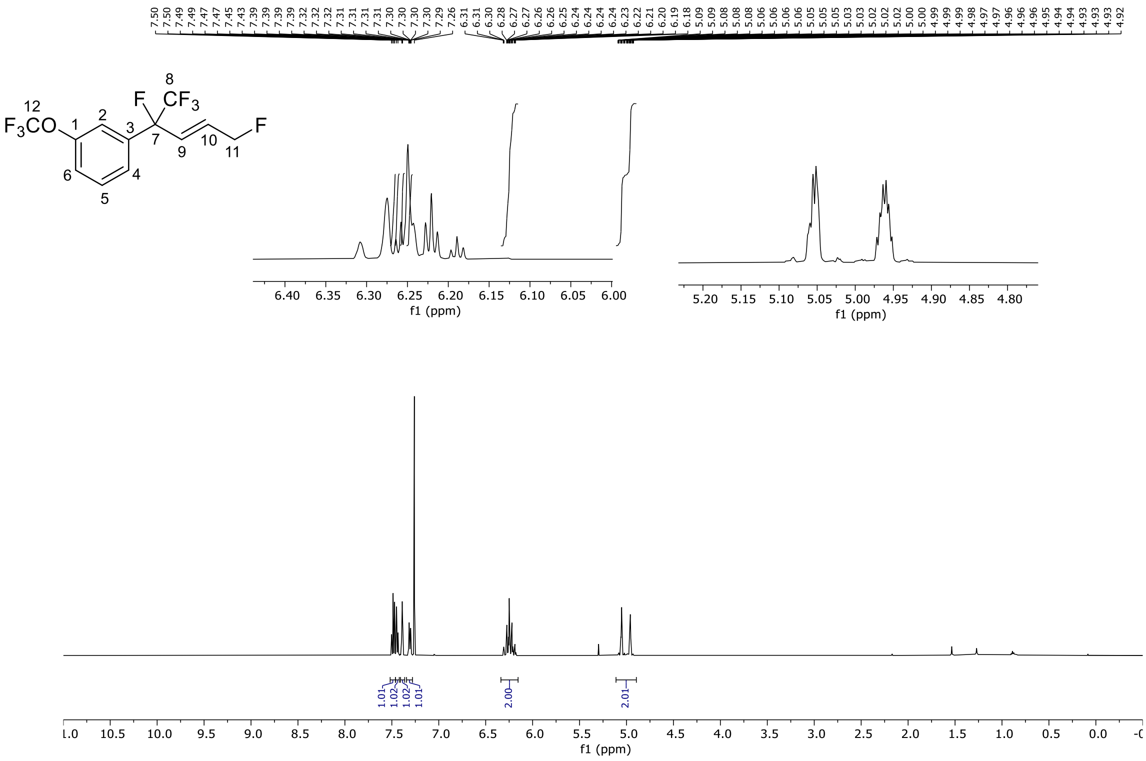

^{13}C NMR (126 MHz, $CDCl_3$)

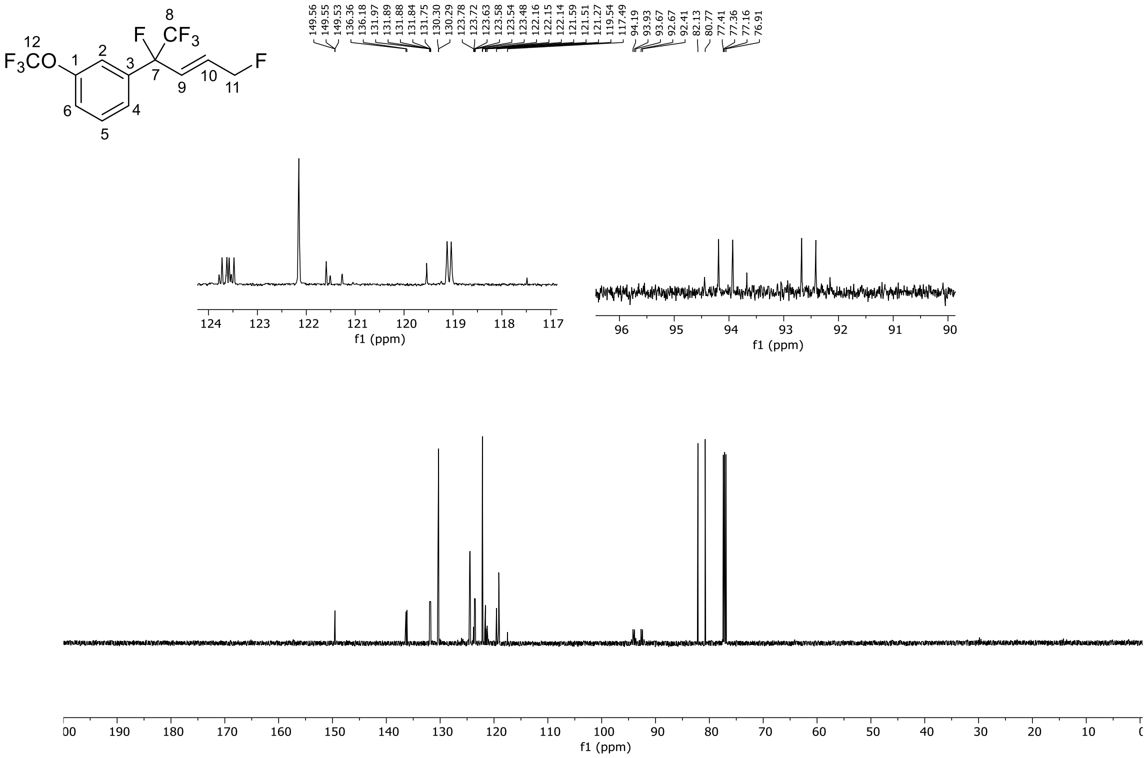

^{19}F NMR (470 MHz, $CDCl_3$)

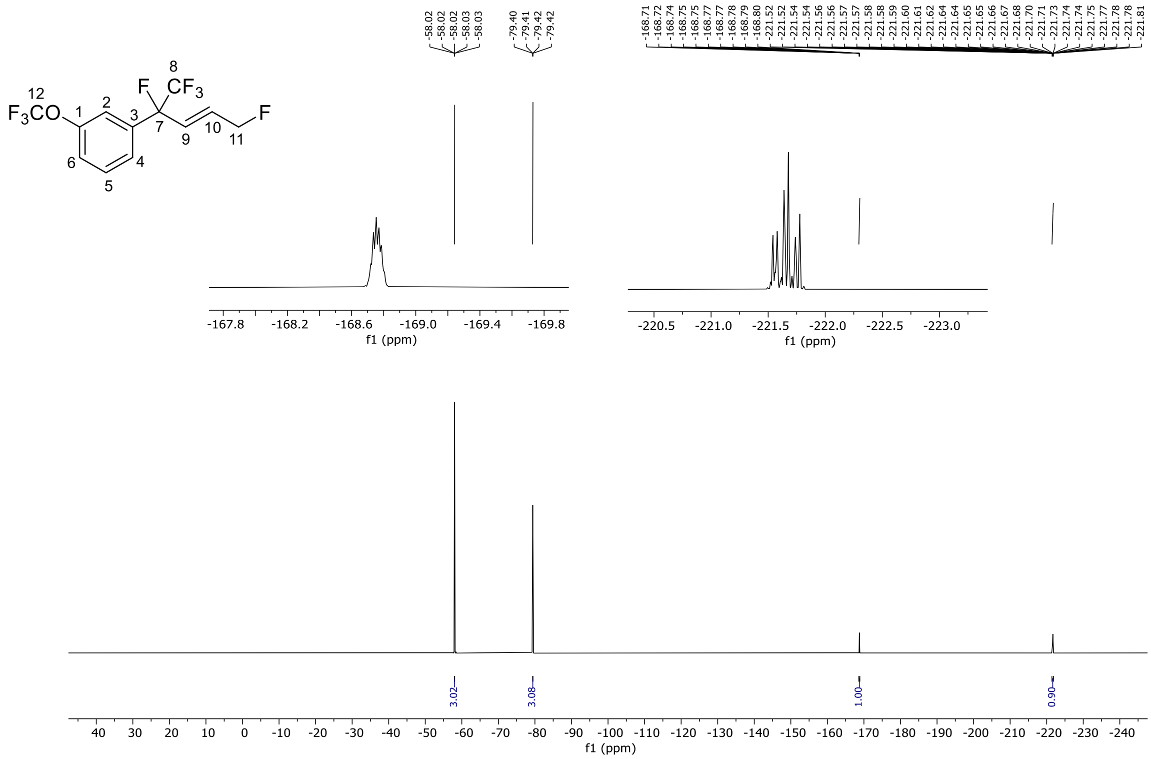

^{19}F{^{1}H} NMR (470 MHz, $CDCl_3$)

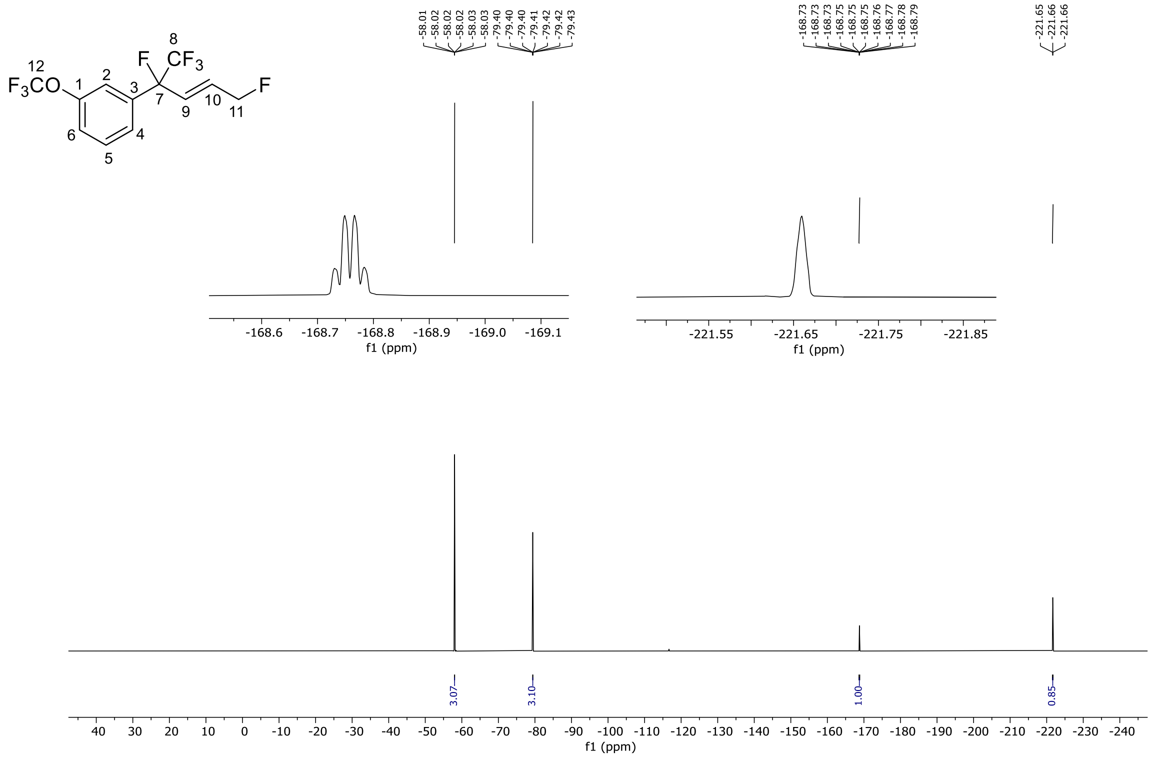

(*E*)-4-(1,1,1,2,5-Pentafluoropent-3-en-2-yl)phenyl trifluoromethanesulfonate (2q)

^{1}H NMR (500 MHz, $CDCl_3$)

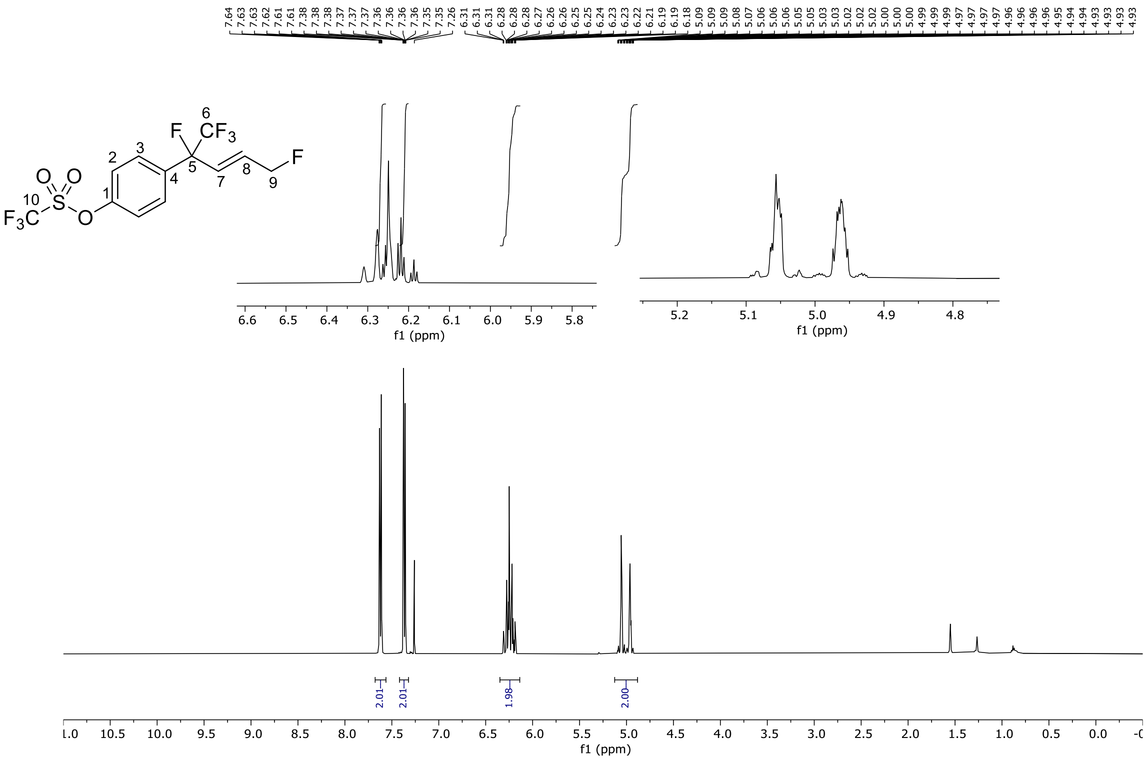

^{13}C NMR (126 MHz, $CDCl_3$)

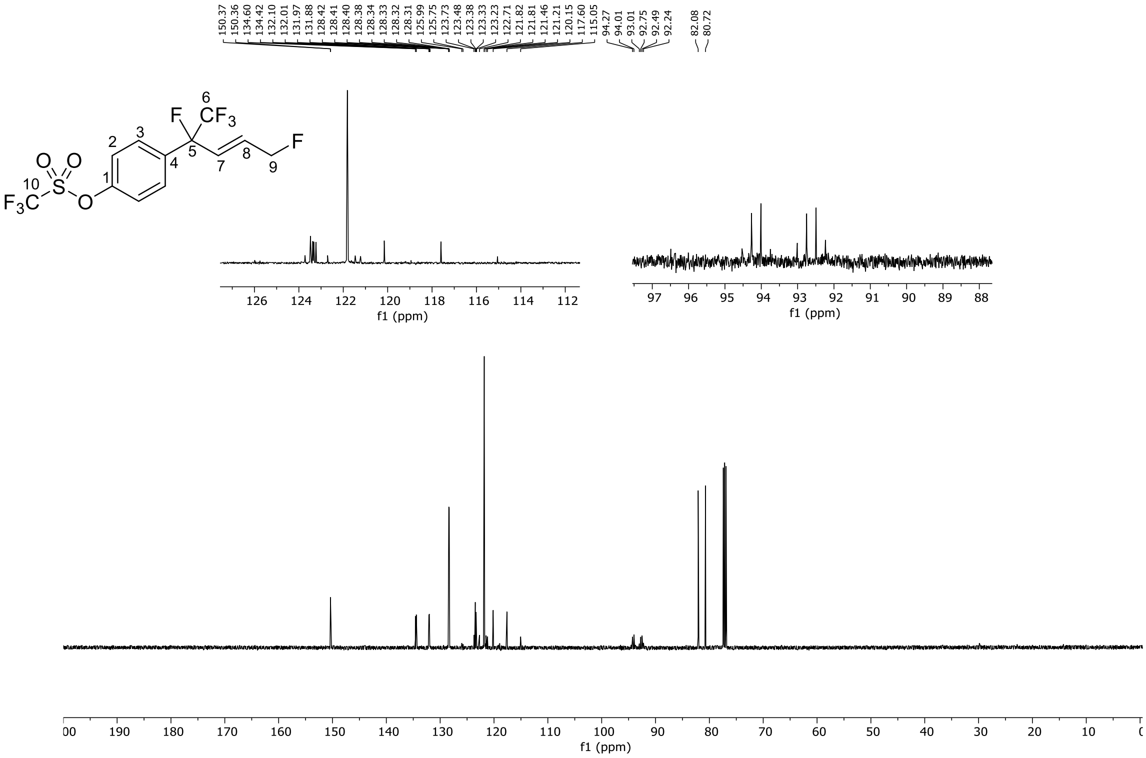

^{19}F NMR (470 MHz, $CDCl_3$)

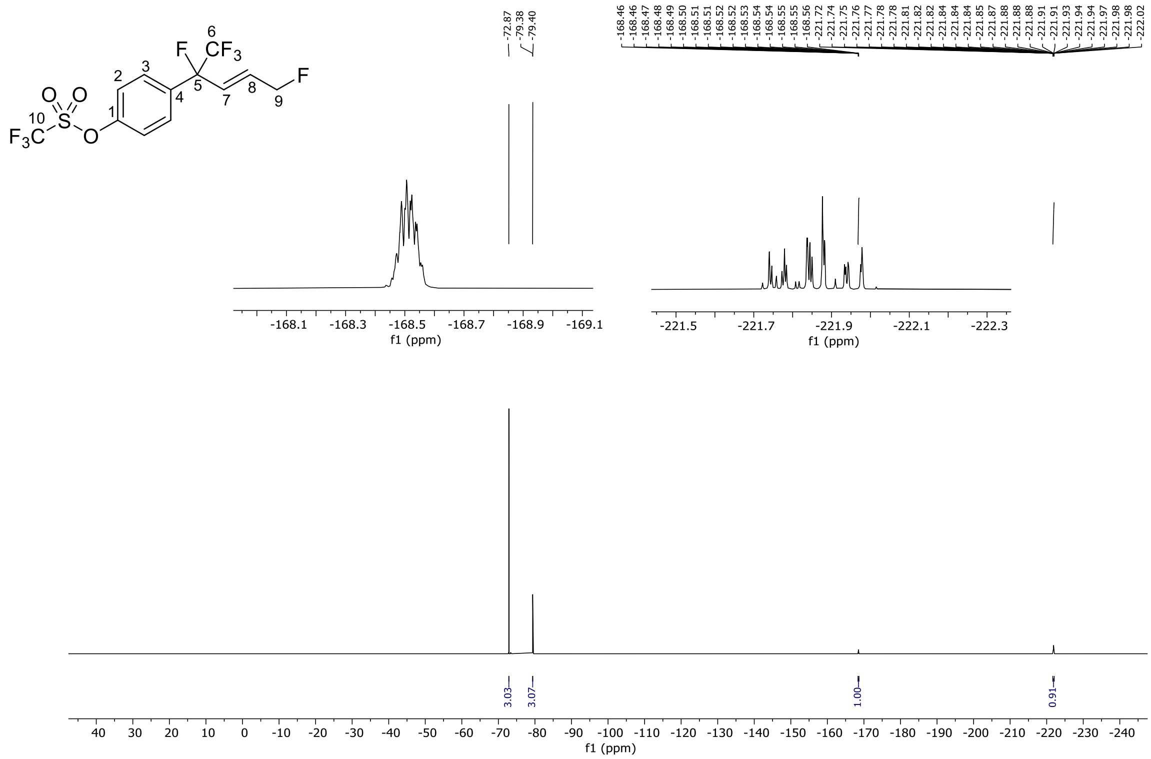

^{19}F{^{1}H} NMR (470 MHz, $CDCl_3$)

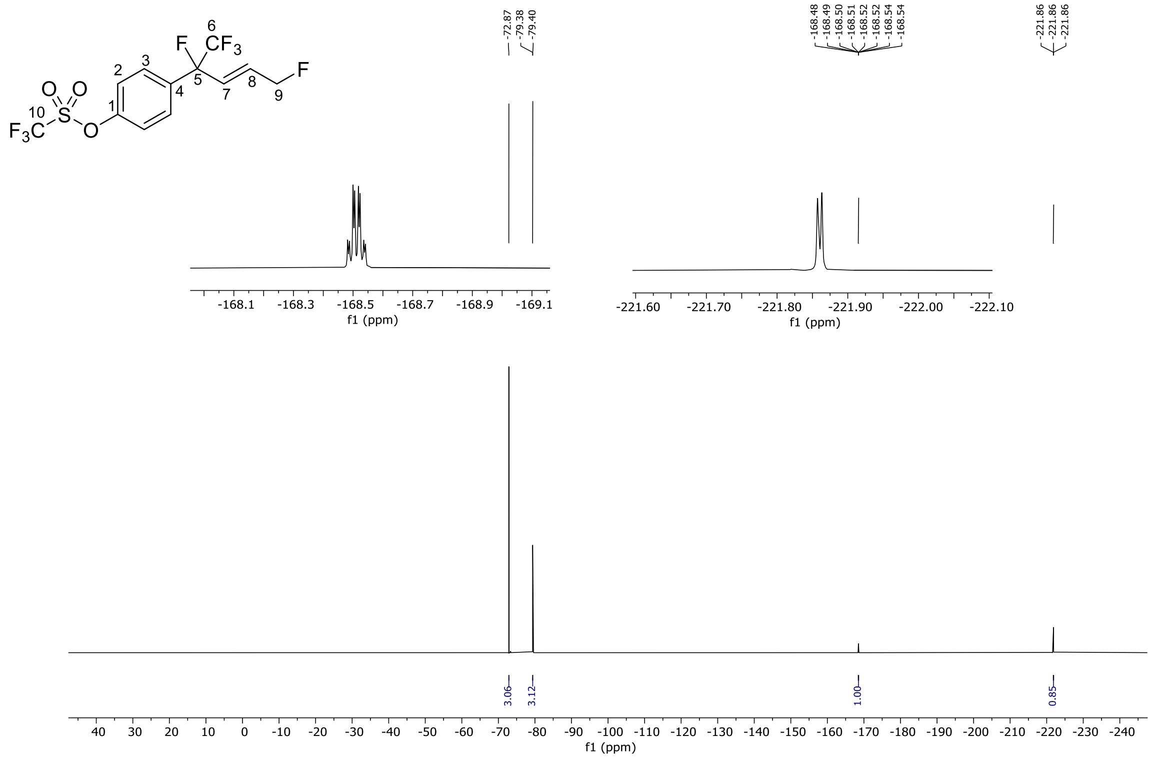

(*E*)-4-(1,1,1,2,5-Pentafluoro-4-methylpent-3-en-2-yl)phenyl 4-methylbenzenesulfonate (2r)

^{1}H NMR (500 MHz, $CDCl_3$)

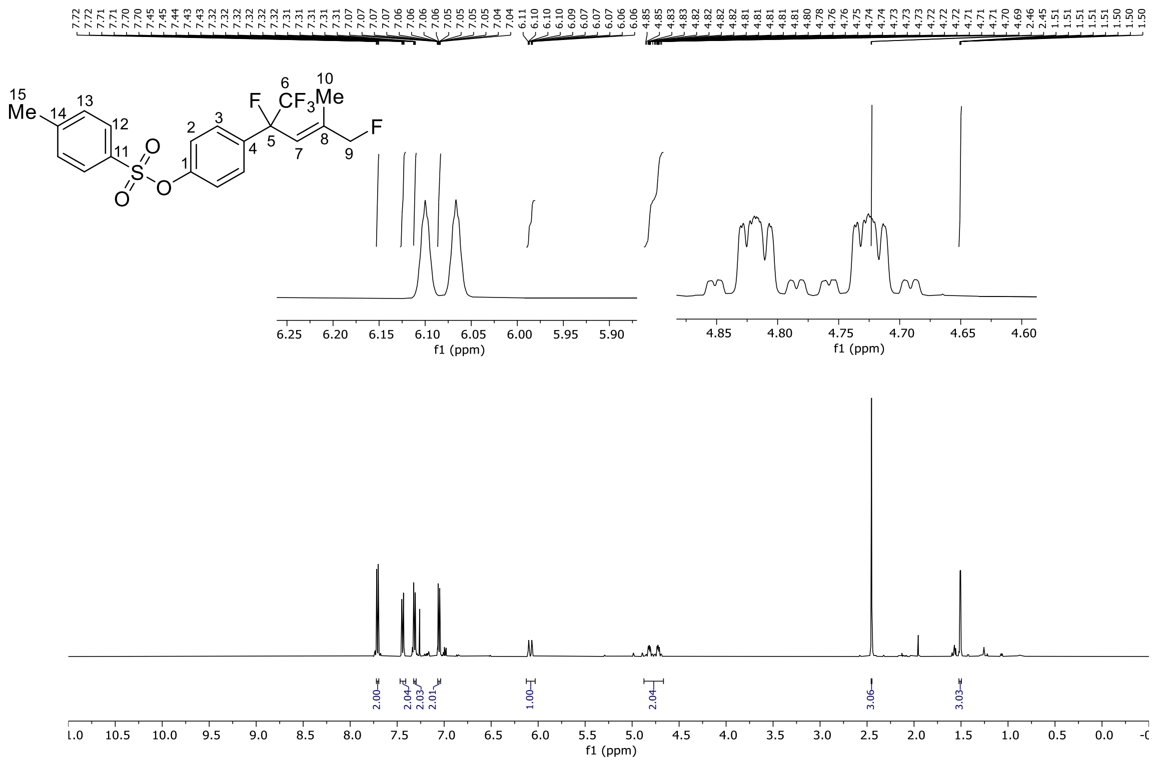

^{13}C NMR (126 MHz, $CDCl_3$)

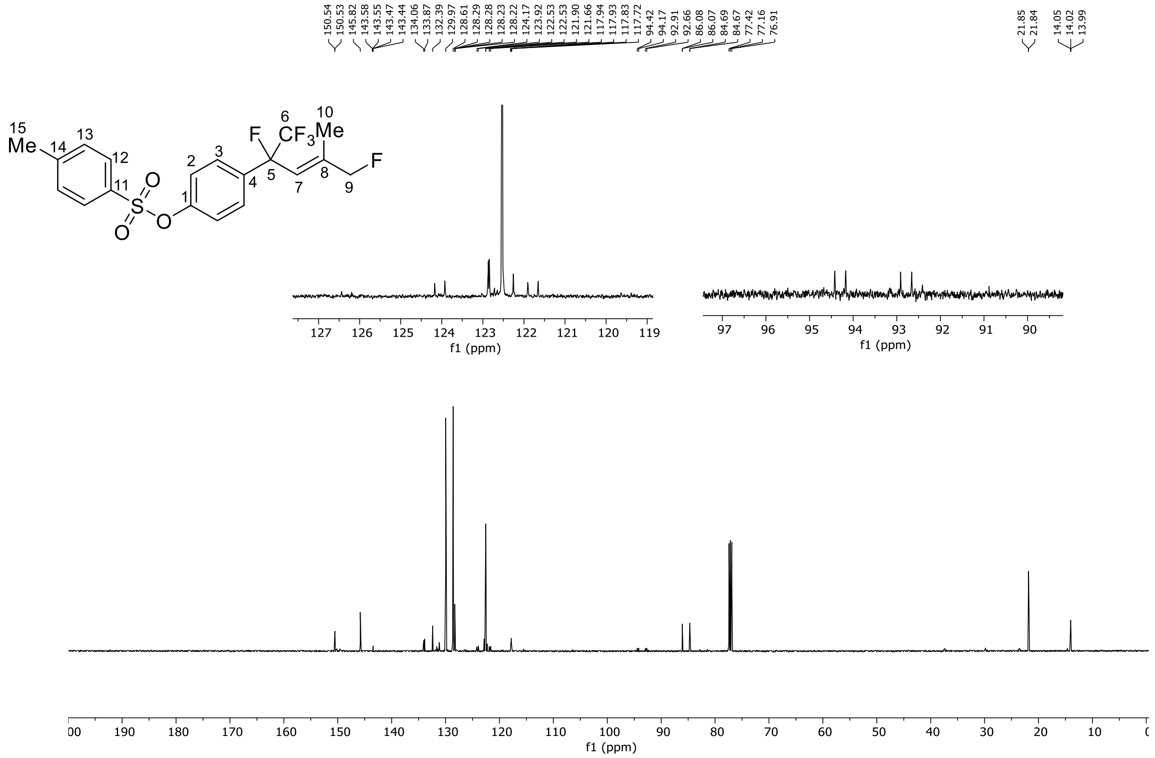

^{19}F NMR (470 MHz, $CDCl_3$)

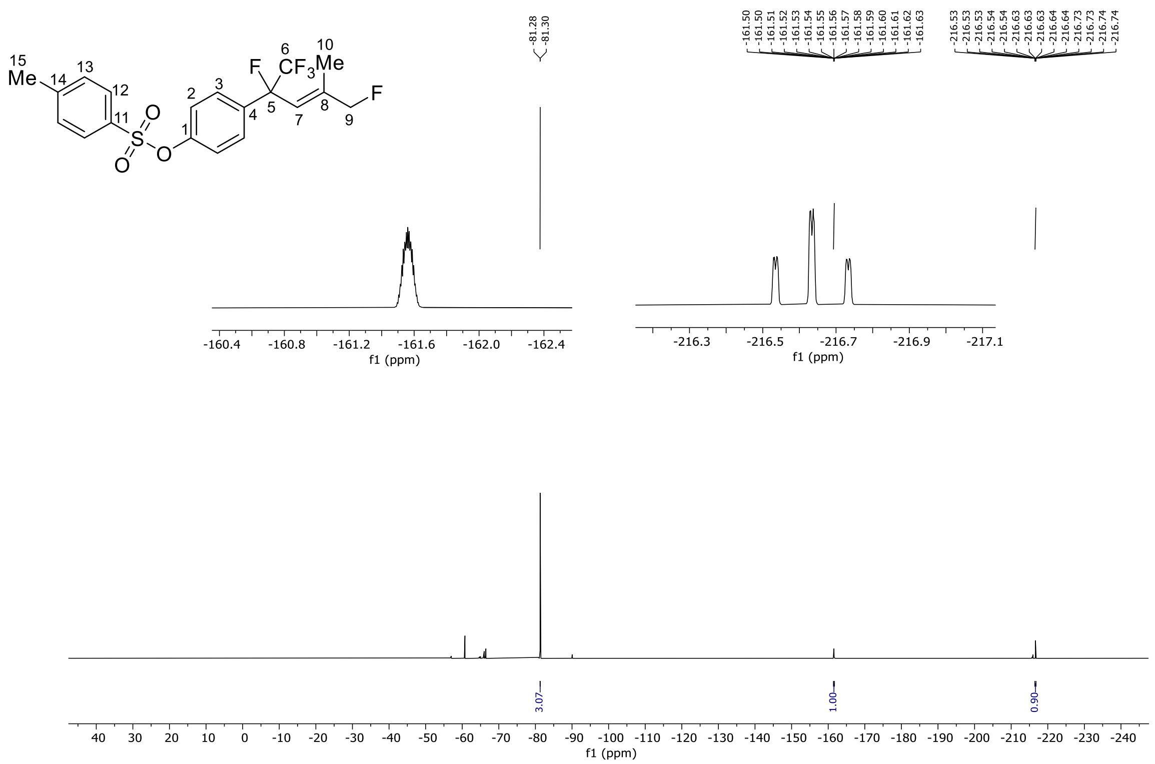

^{19}F{^{1}H} NMR (470 MHz, $CDCl_3$)

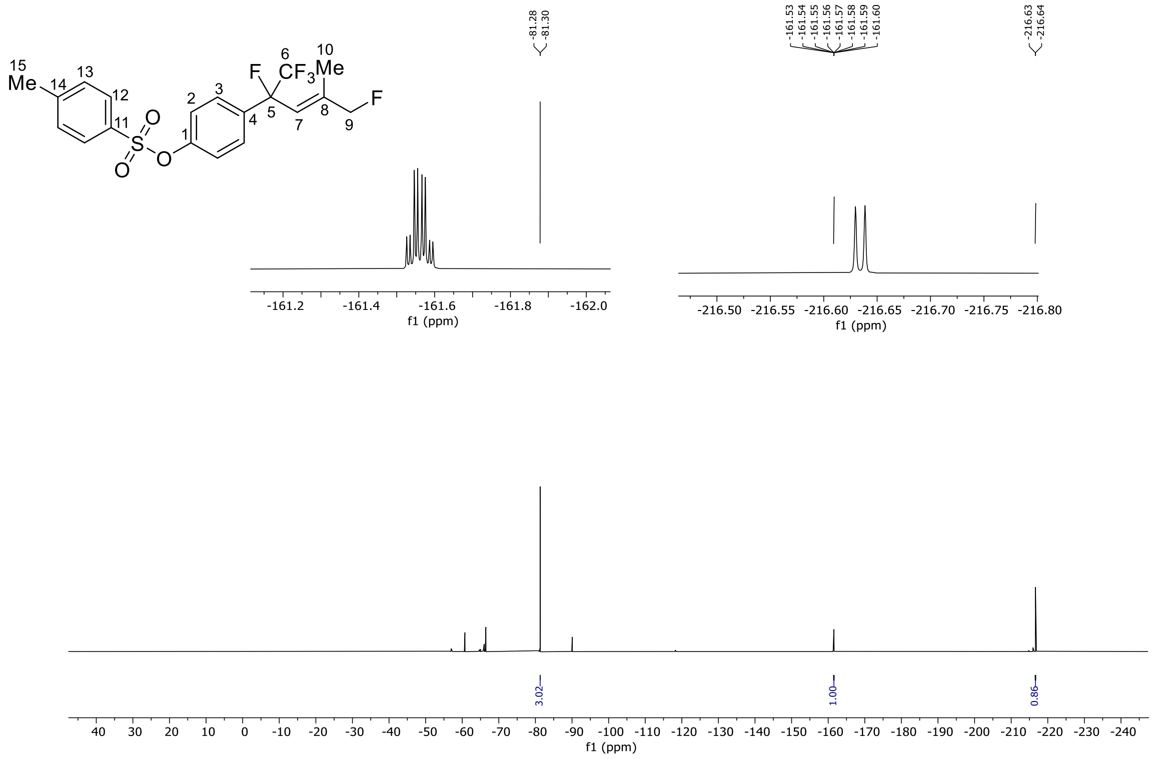

(*E*)-(1,1,1,2,5-Pentafluoropent-3-en-2-yl)cyclohexane (2s)

^{1}H NMR (500 MHz, $CDCl_3$)

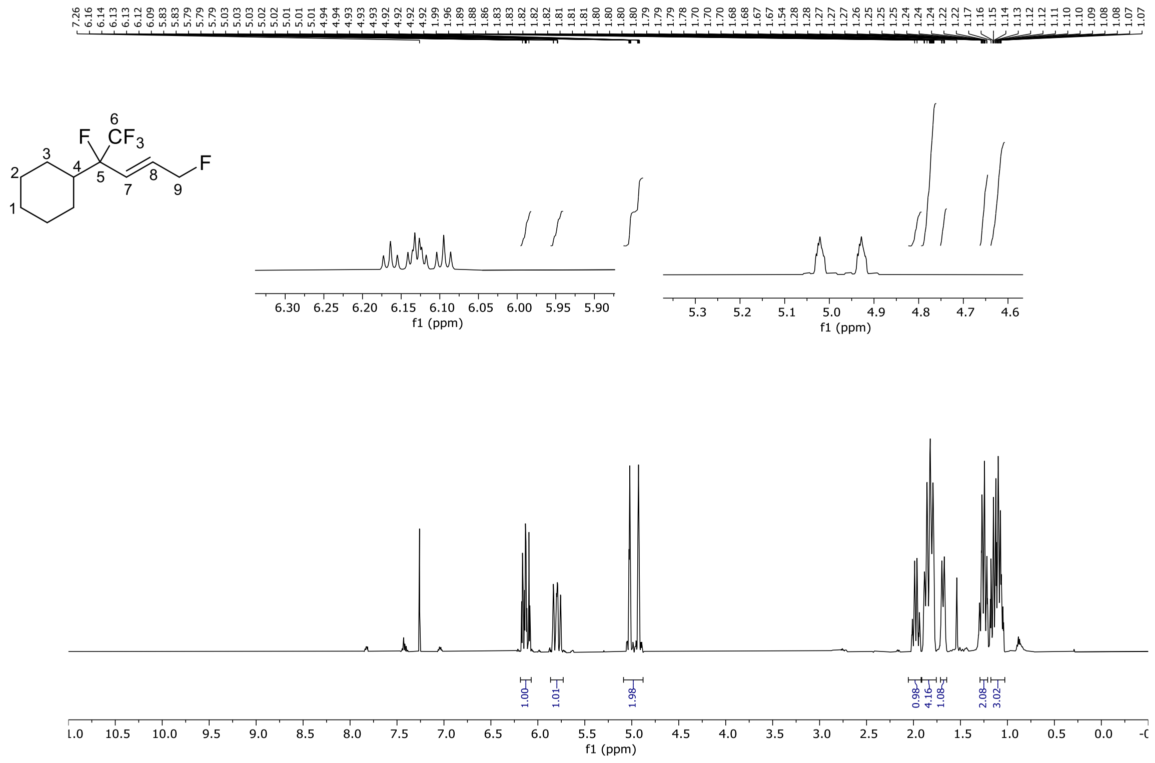

^{13}C NMR (126 MHz, $CDCl_3$)

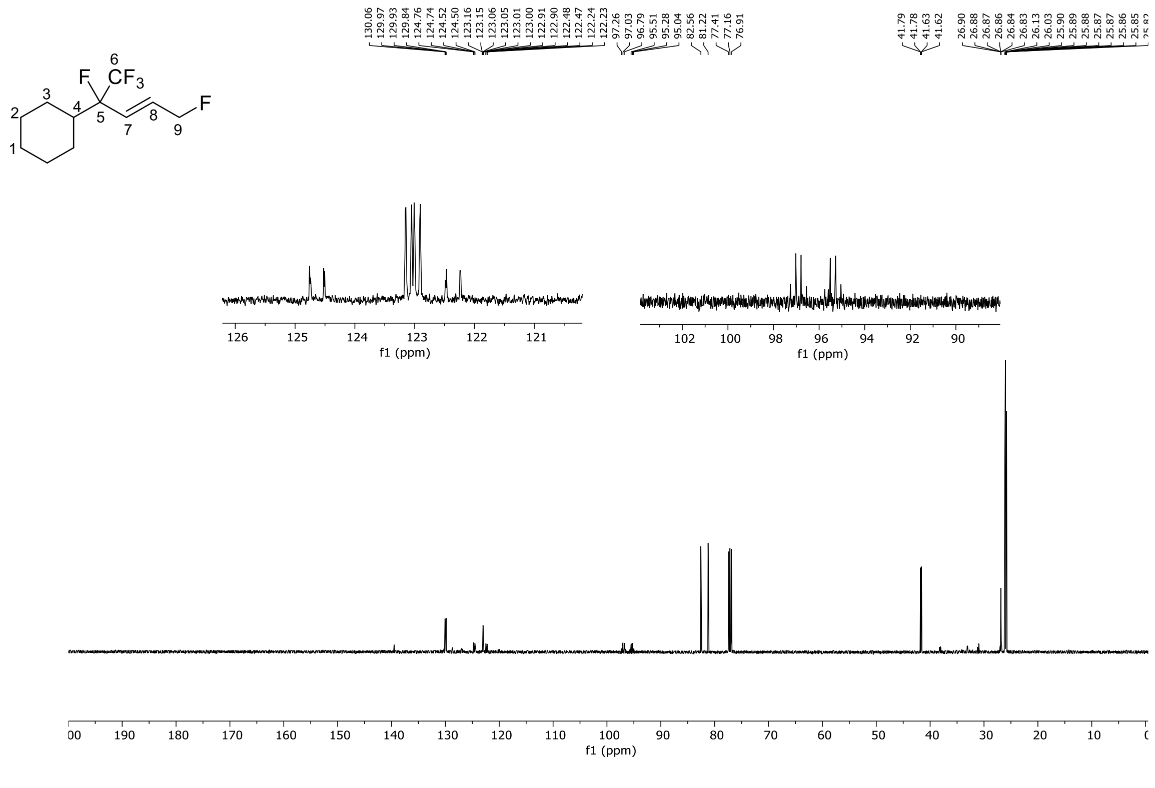

^{19}F NMR (470 MHz, $CDCl_3$)

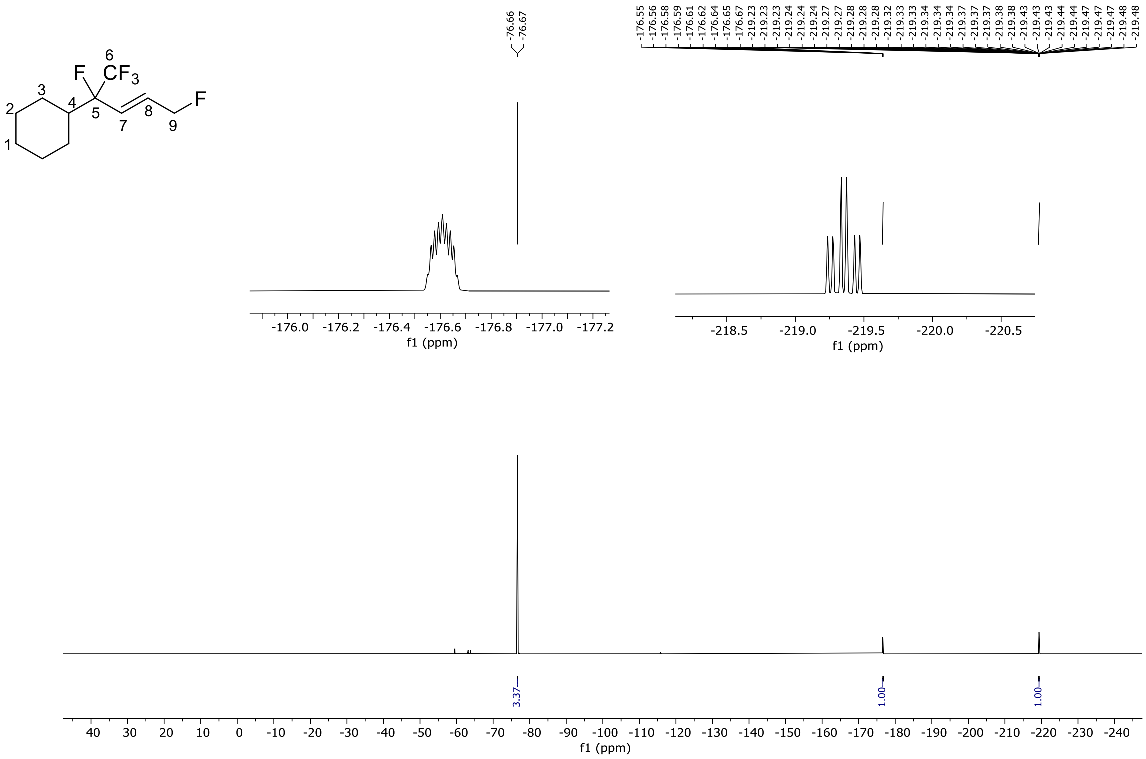

^{19}F{^{1}H} NMR (377 MHz, $CDCl_3$)

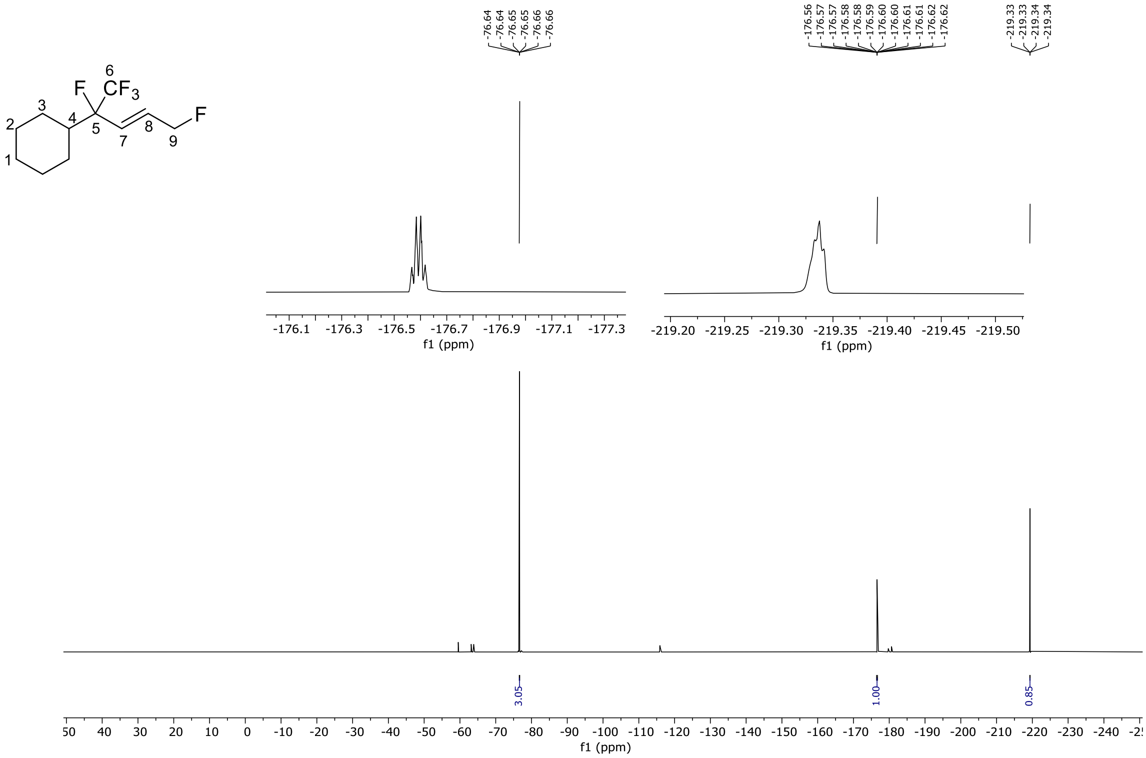

(*E*)-2-(4-(1,1,1,2,5-Pentafluoropent-3-en-2-yl)phenyl)isoindoline-1,3-dione (2t)

^{1}H NMR (500 MHz, $CDCl_3$)

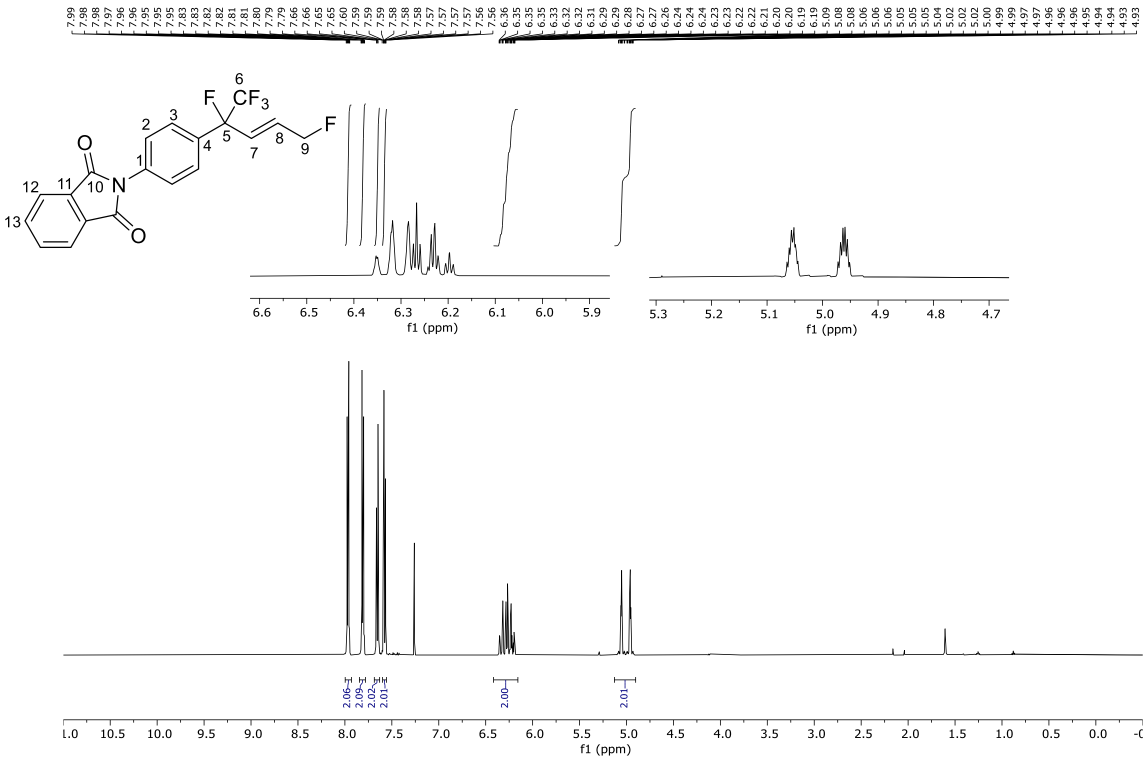

^{13}C NMR (126 MHz, $CDCl_3$)

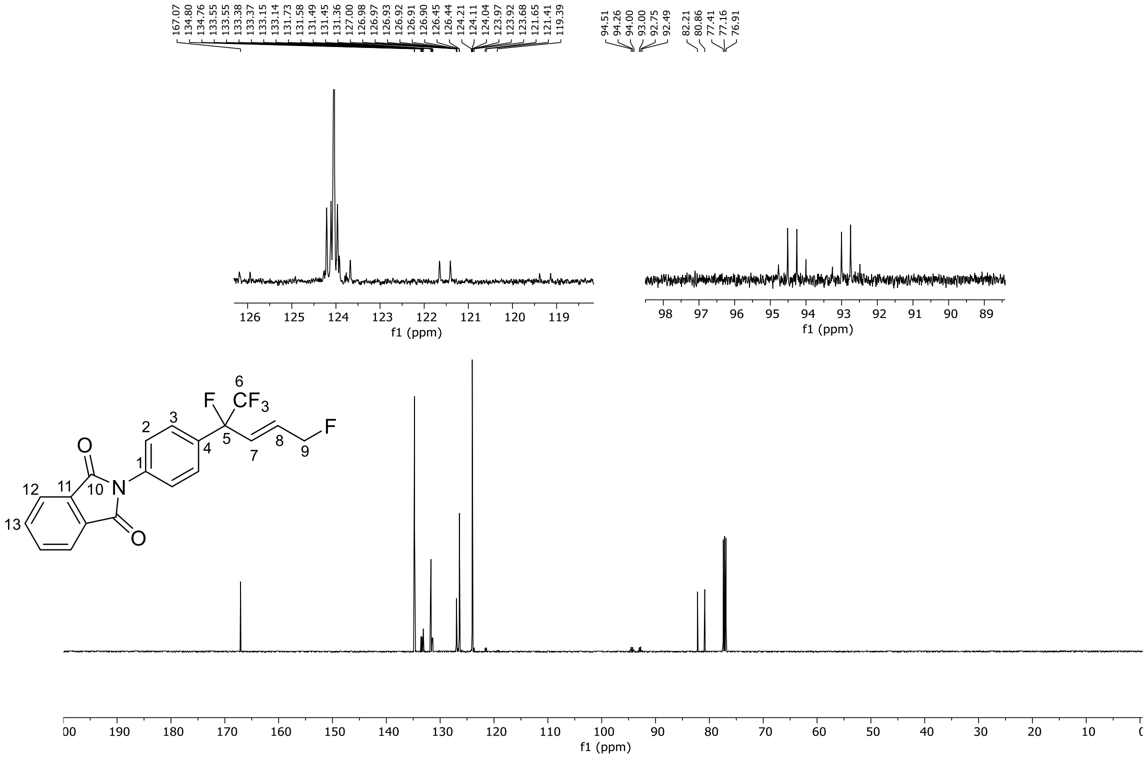

^{19}F NMR (470 MHz, $CDCl_3$)

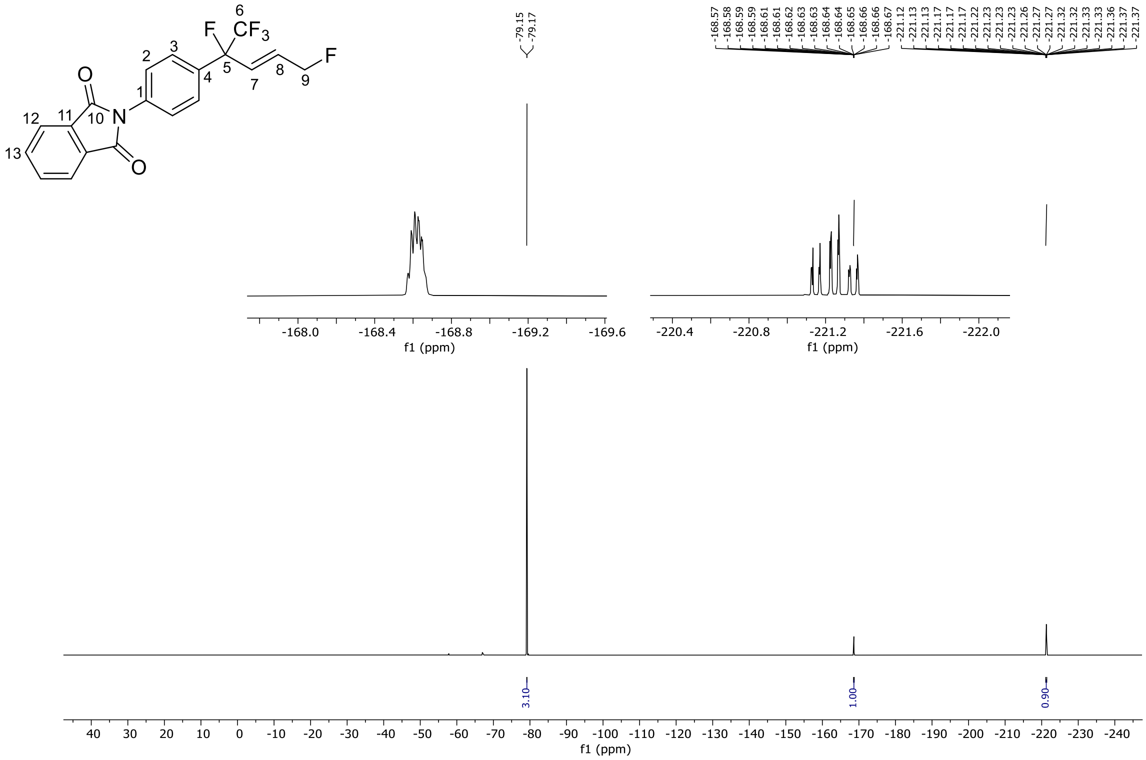

^{19}F{^{1}H} NMR (470 MHz, $CDCl_3$)

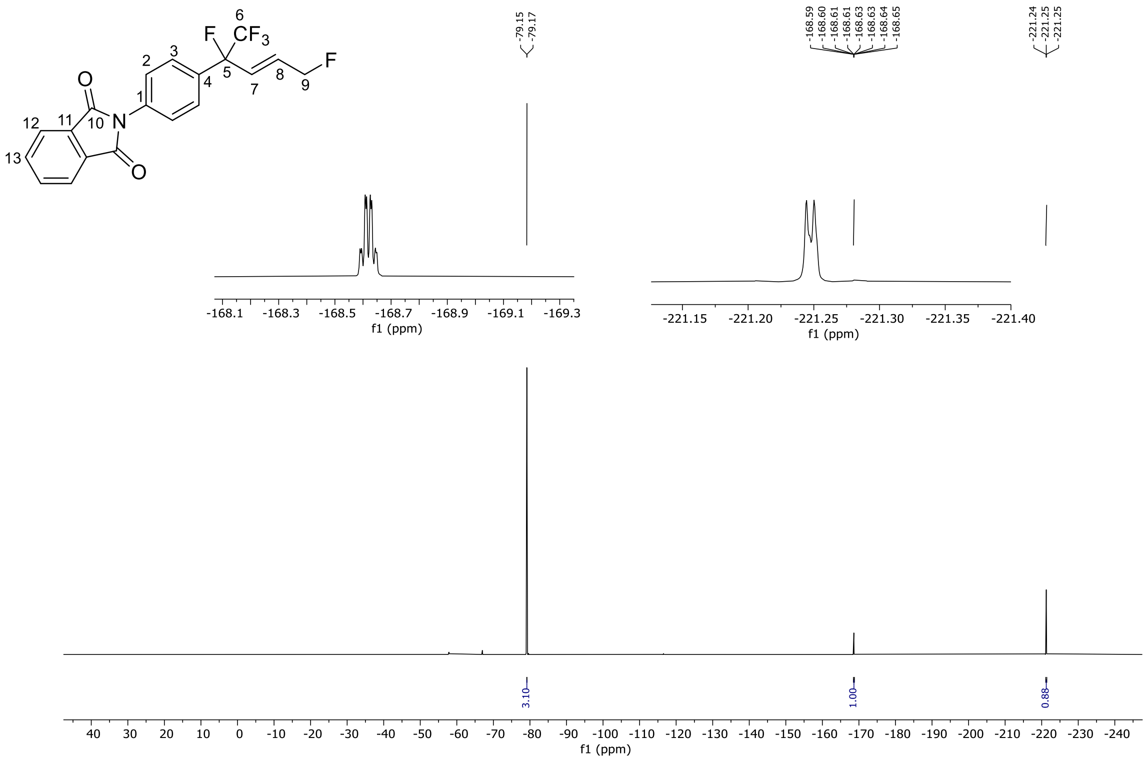

(*E*)-*N*-(4-(4-Bromophenyl)-4,5,5,5-tetrafluoropent-2-en-1-yl)acetamide (3a)

^{1}H NMR (599 MHz, $CDCl_3$)

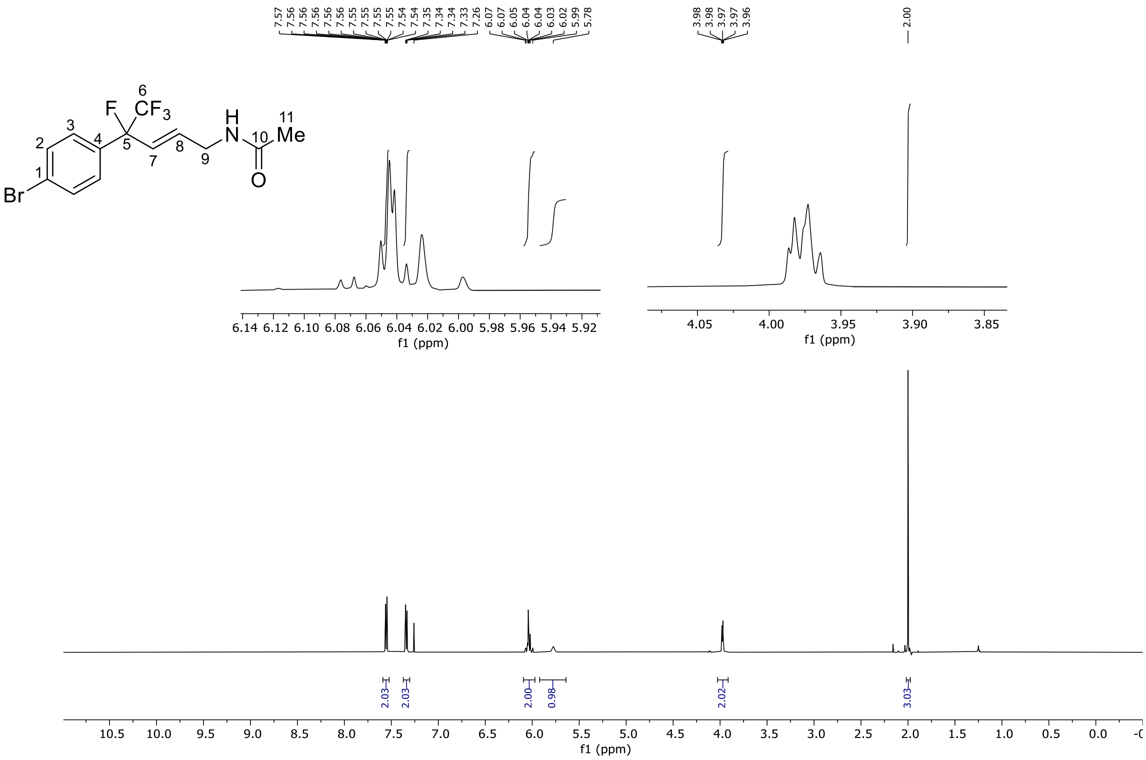

^{13}C NMR (151 MHz, $CDCl_3$)

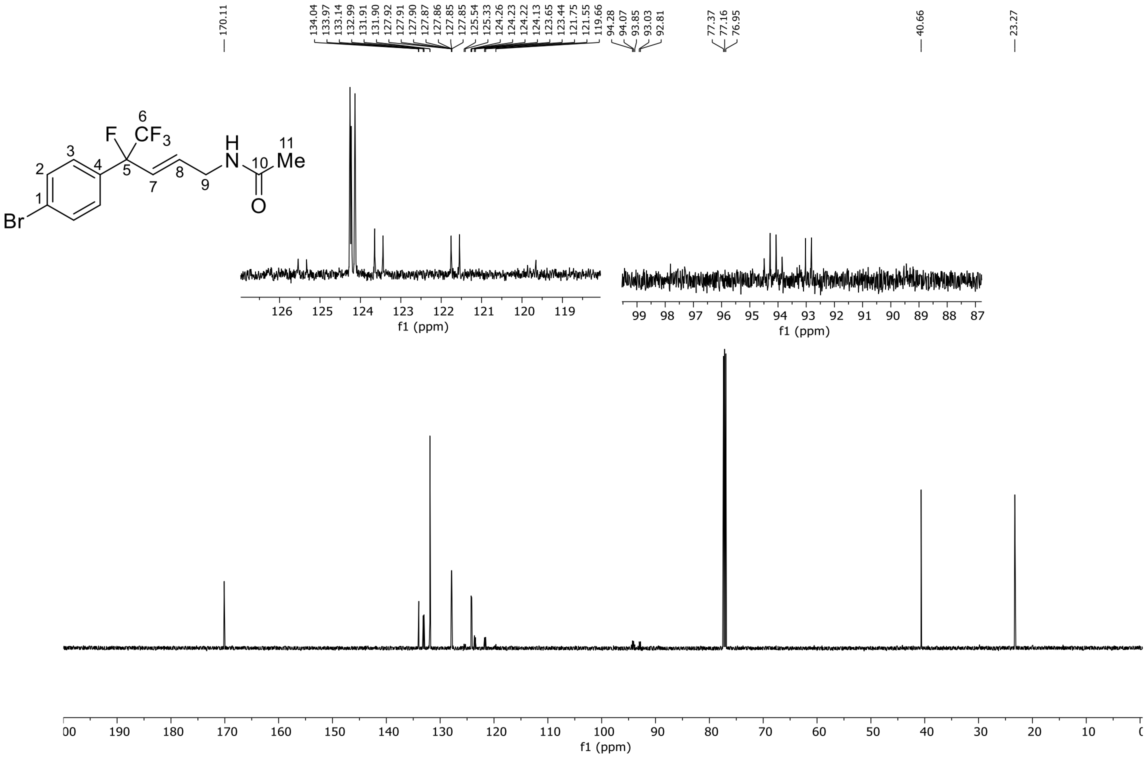

^{19}F NMR (564 MHz, $CDCl_3$)

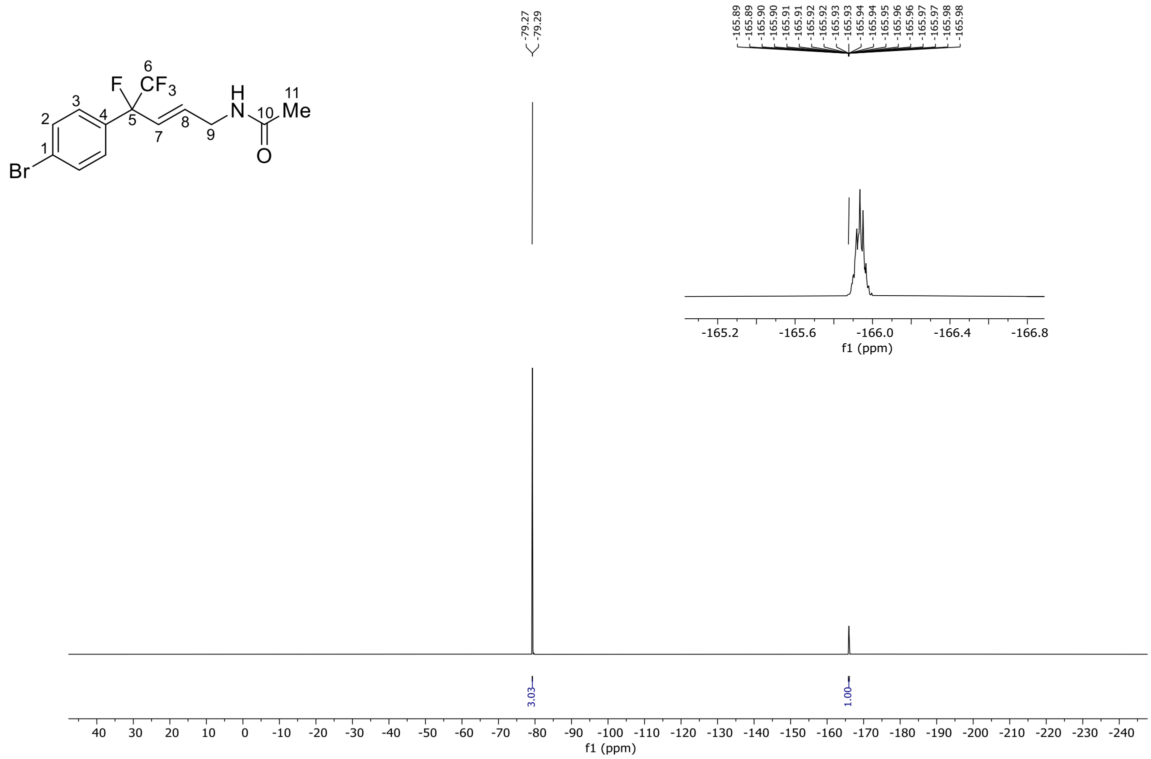

^{19}F{^{1}H} NMR (564 MHz, $CDCl_3$)

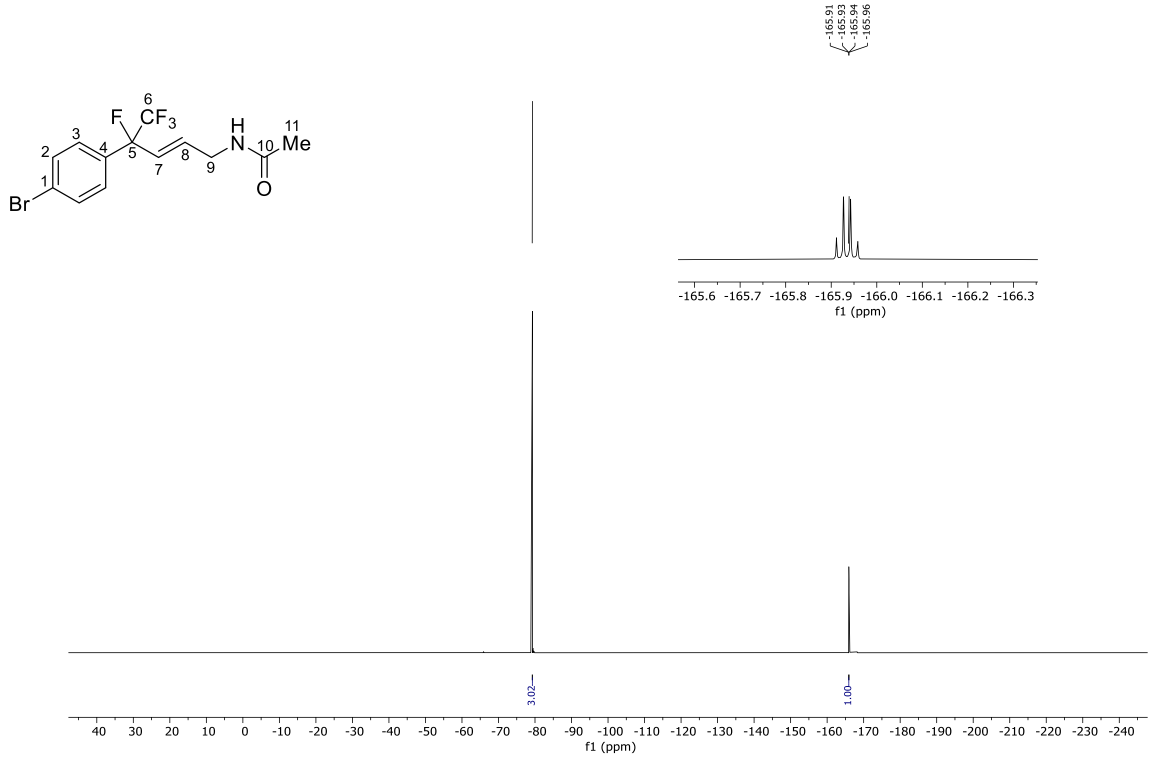

(*E*)-*N*-(4-(4-Bromophenyl)-4,5,5,5-tetrafluoropent-2-en-1-yl)propionamide (3b)

^{1}H NMR (500 MHz, $CDCl_3$)

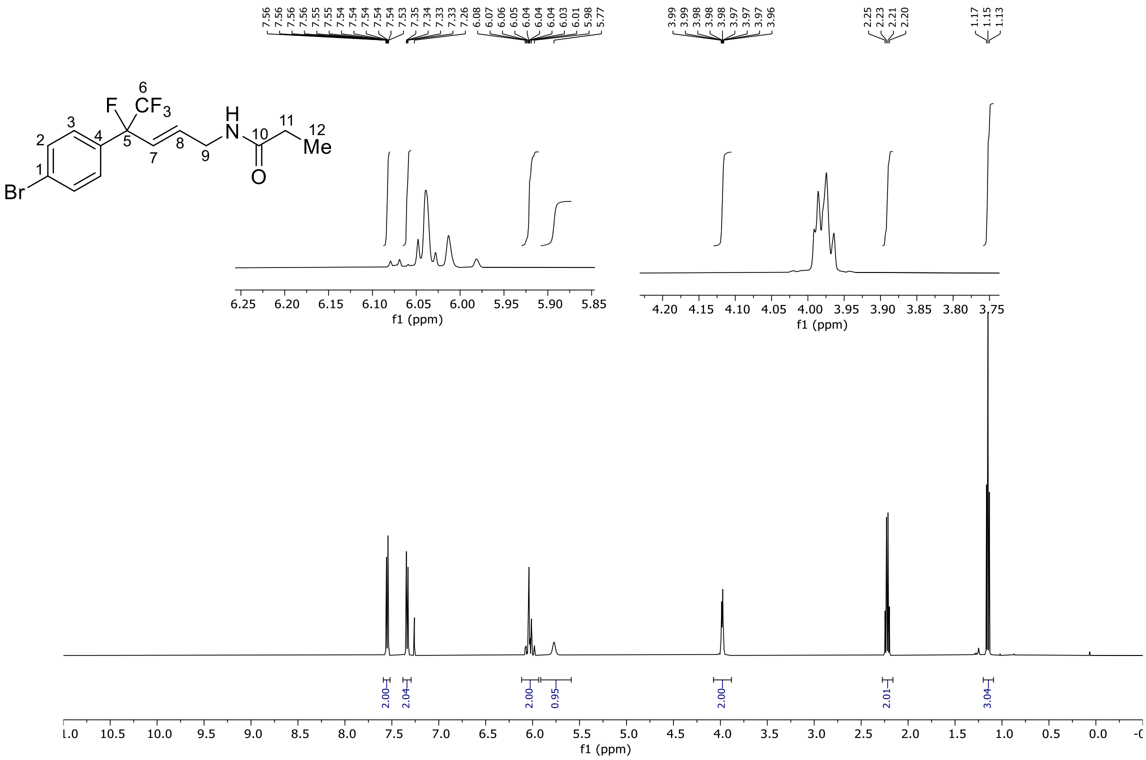

^{13}C NMR (126 MHz, $CDCl_3$)

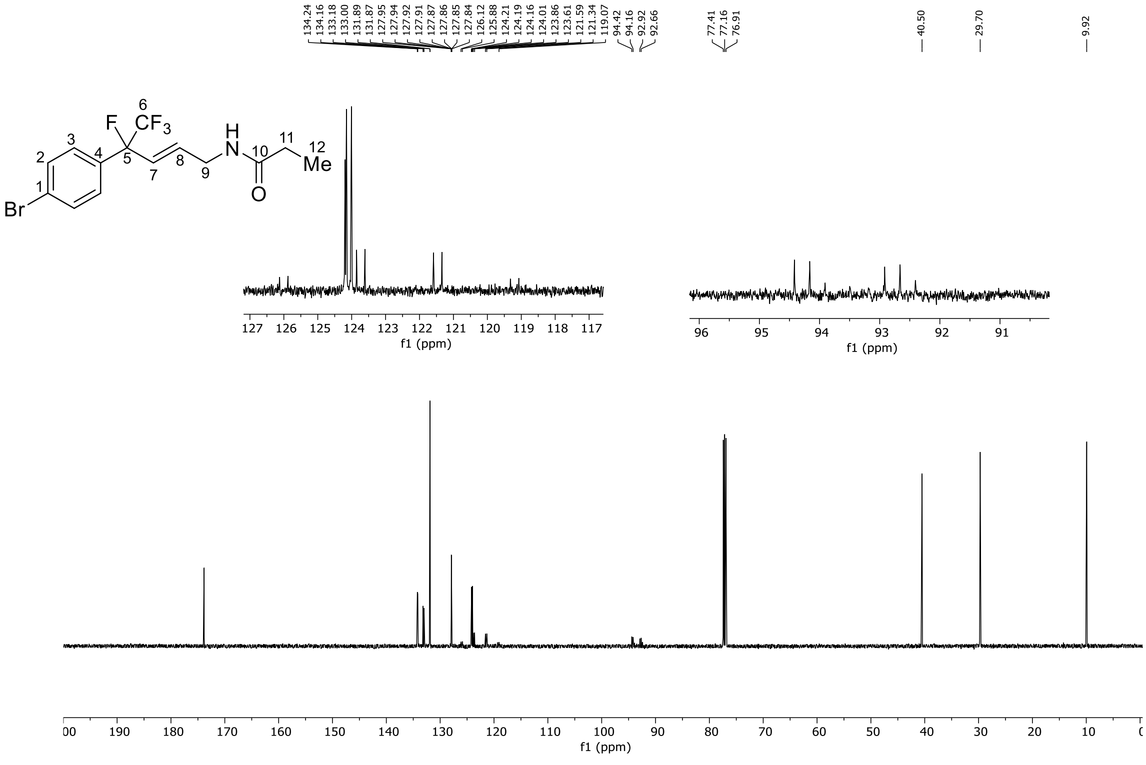

^{19}F NMR (470 MHz, $CDCl_3$)

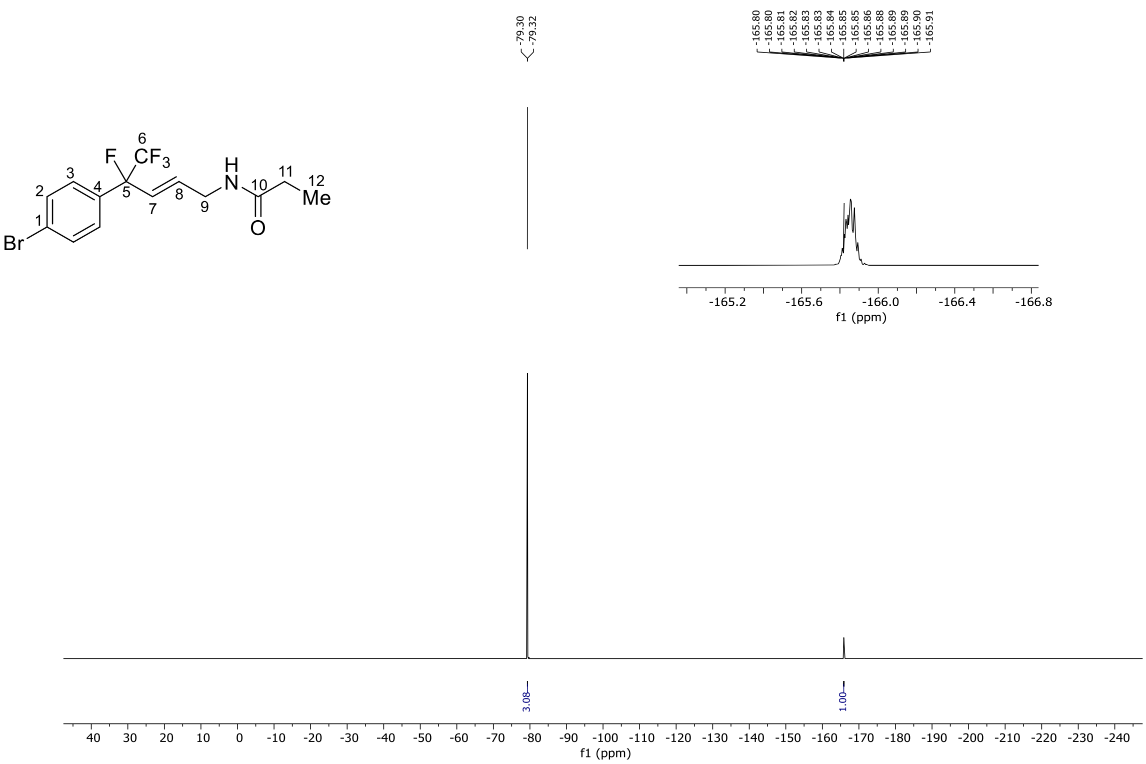

^{19}F{^{1}H} NMR (470 MHz, $CDCl_3$)

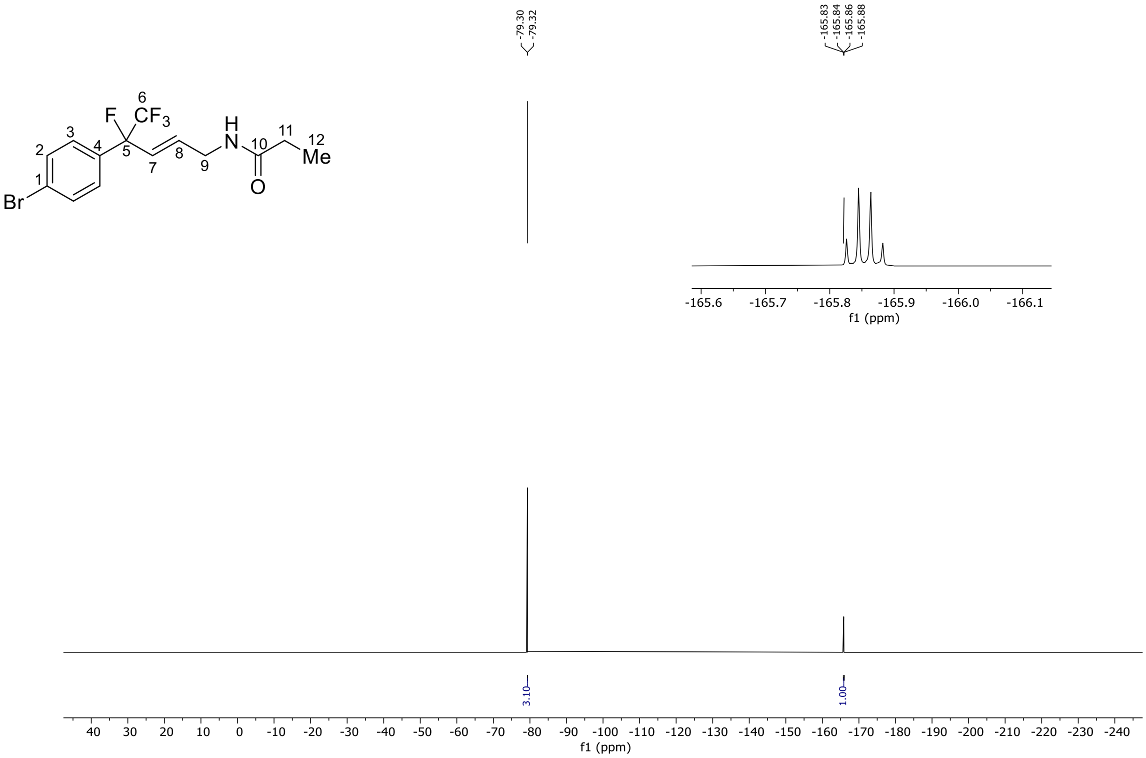

(*E*)-*N*-(4-(4-Bromophenyl)-4,5,5,5-tetrafluoropent-2-en-1-yl)isobutyramide (3c)

^{1}H NMR (500 MHz, $CDCl_3$)

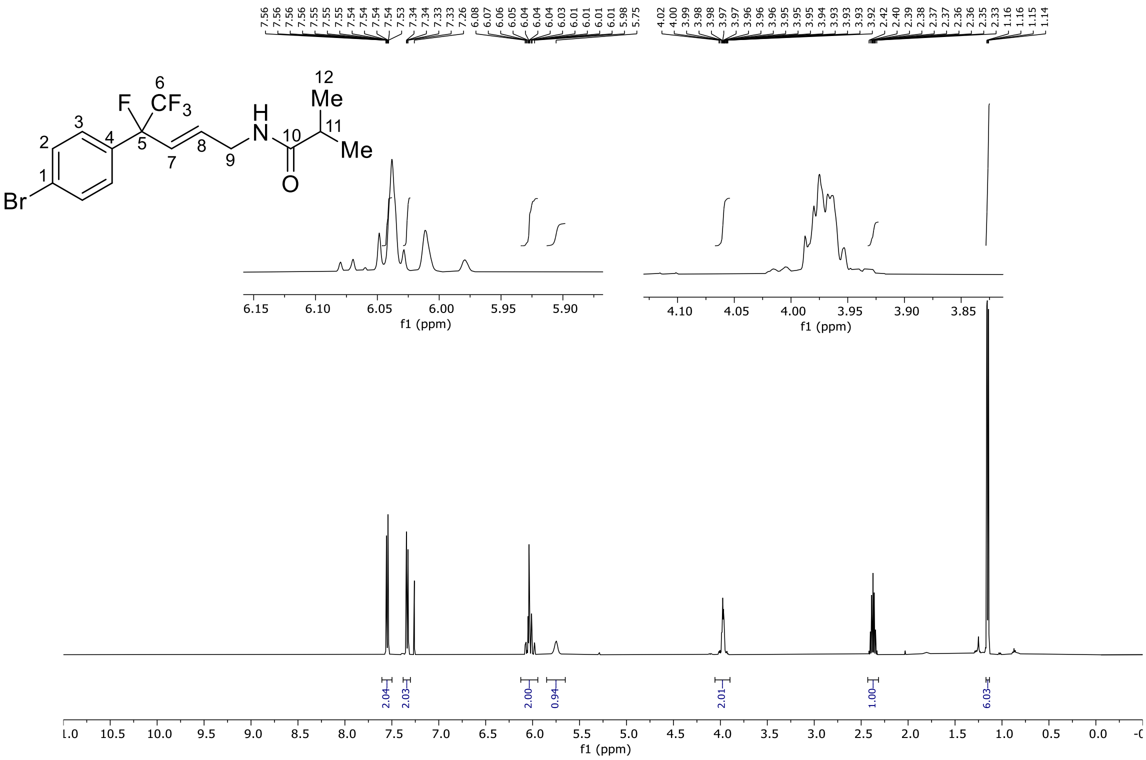

^{13}C NMR (126 MHz, $CDCl_3$)

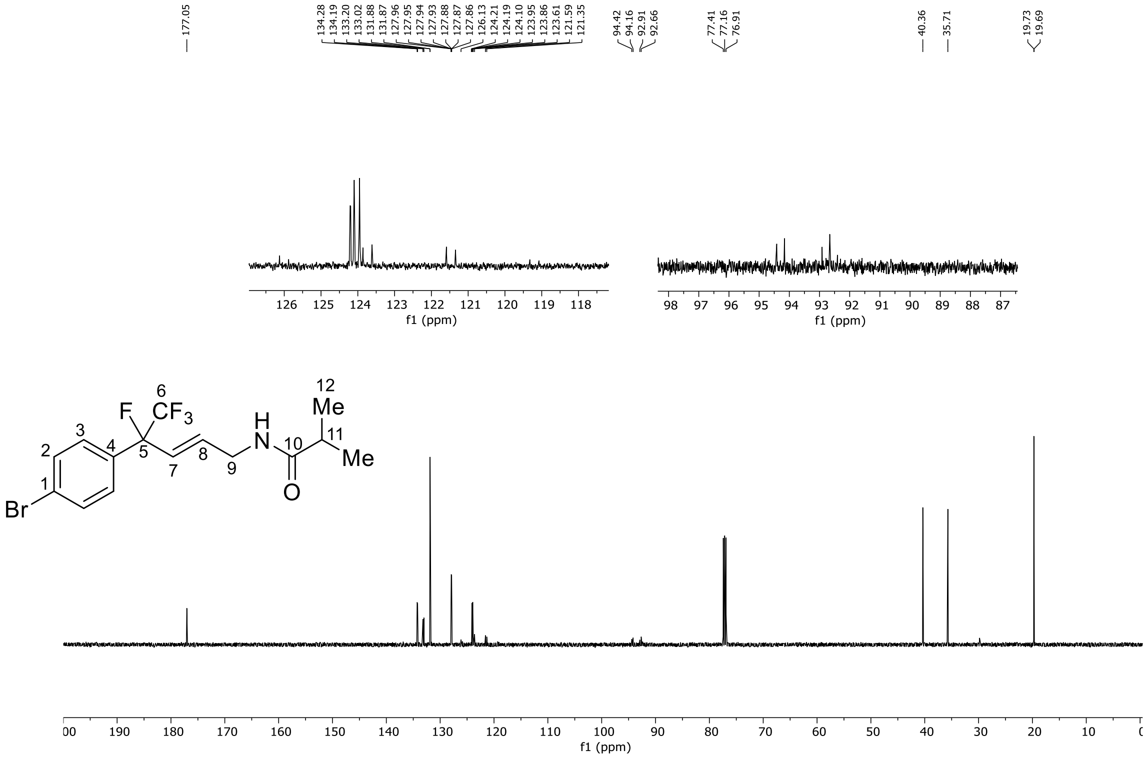

^{19}**F NMR** (470 MHz, $CDCl_3$)

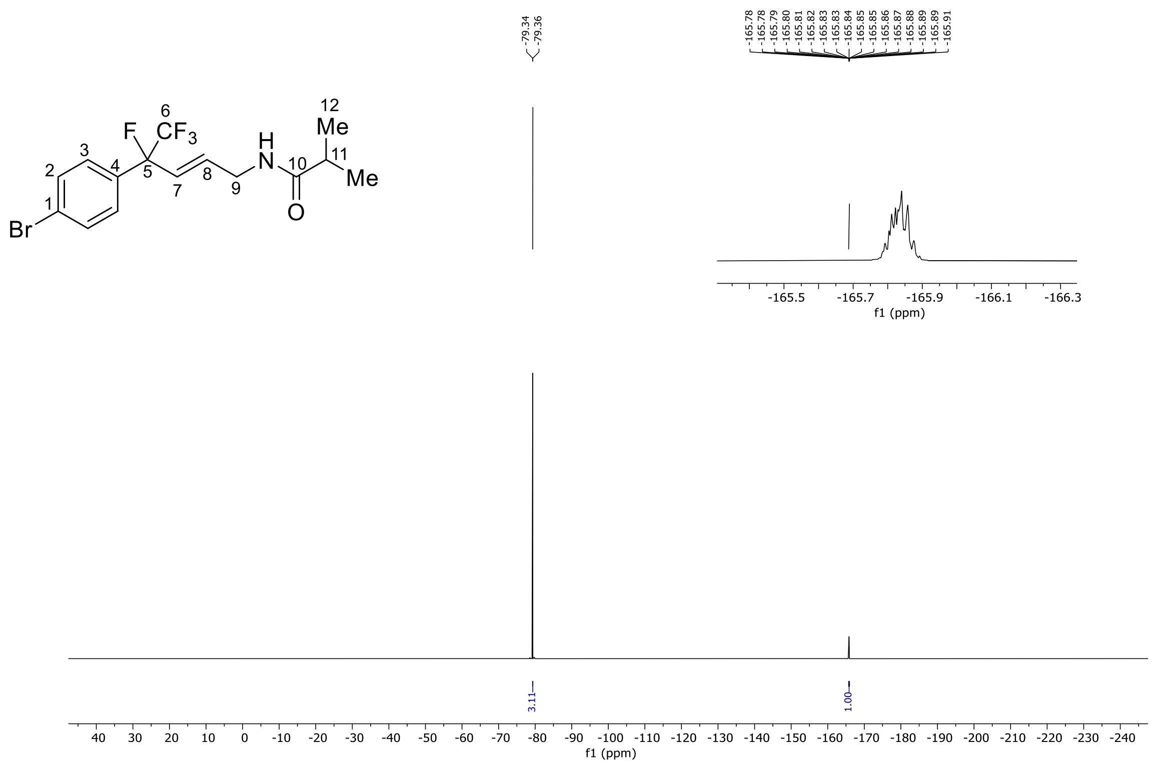

^{19}**F{^{1}H} NMR** (470 MHz, $CDCl_3$)

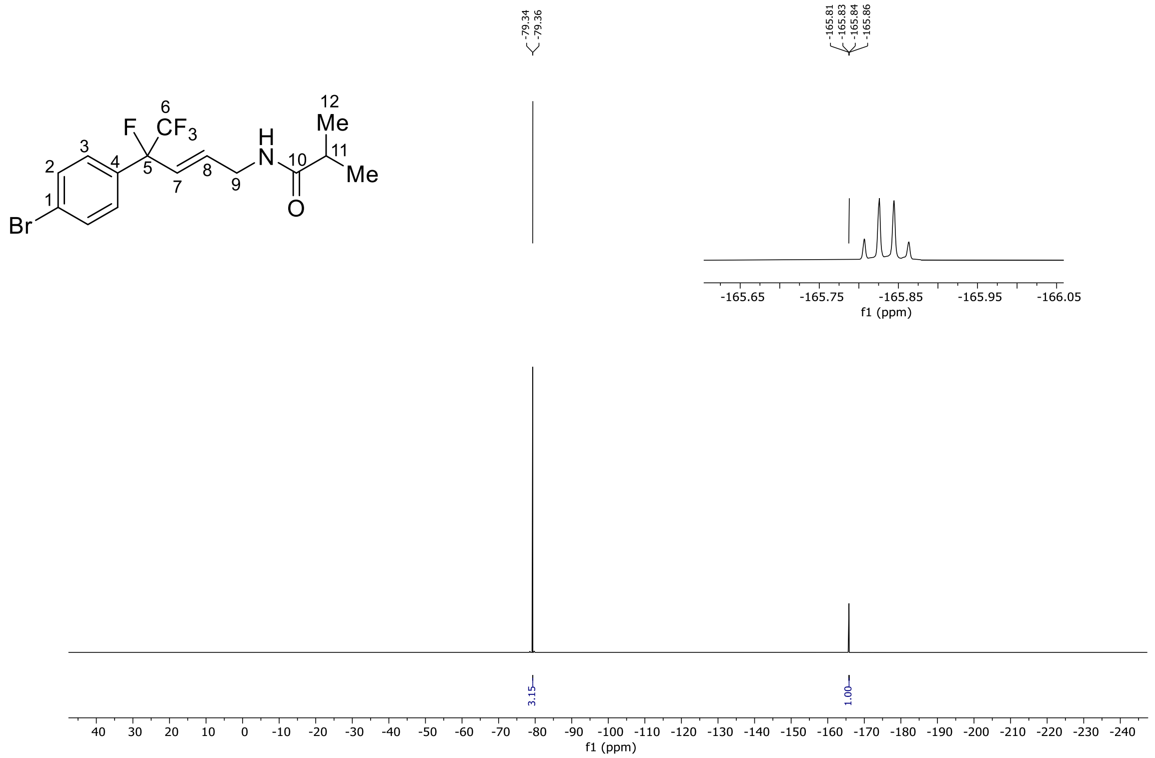

(*E*)-*N*-(4-(4-Bromophenyl)-4,5,5,5-tetrafluoropent-2-en-1-yl)pivalamide (3d)

^{1}H NMR (500 MHz, $CDCl_3$)

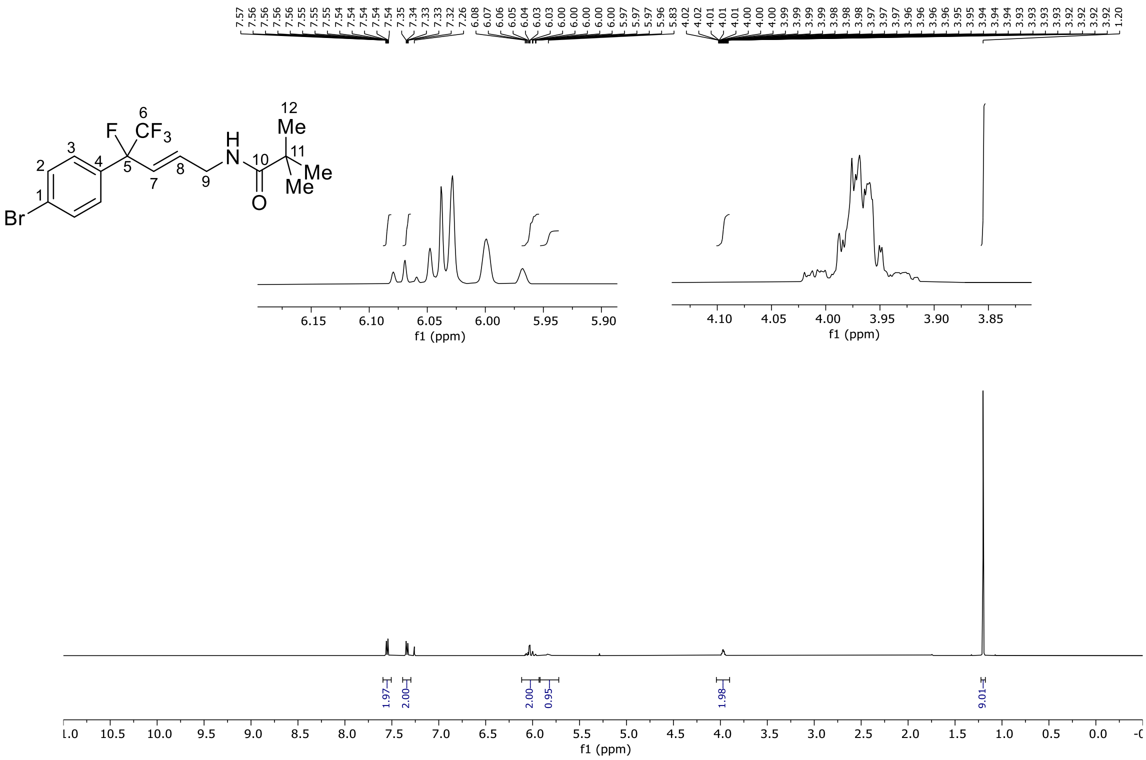

^{13}C NMR (126 MHz, $CDCl_3$)

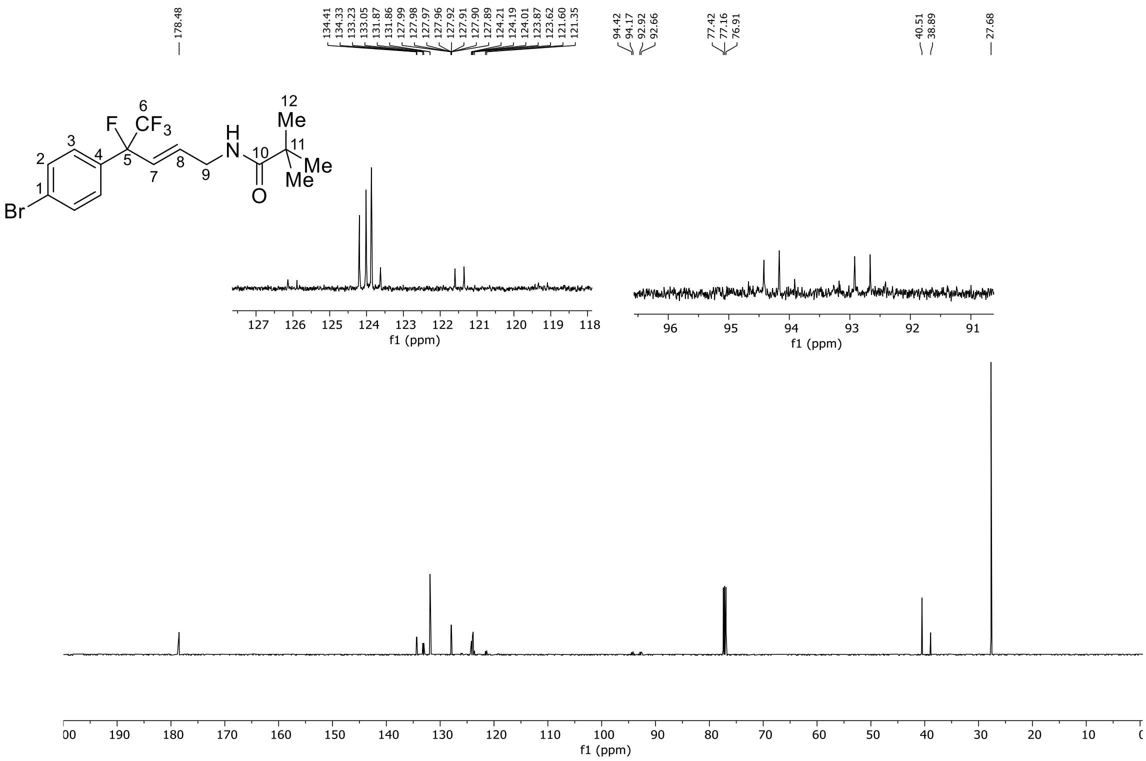

^{19}F NMR (470 MHz, $CDCl_3$)

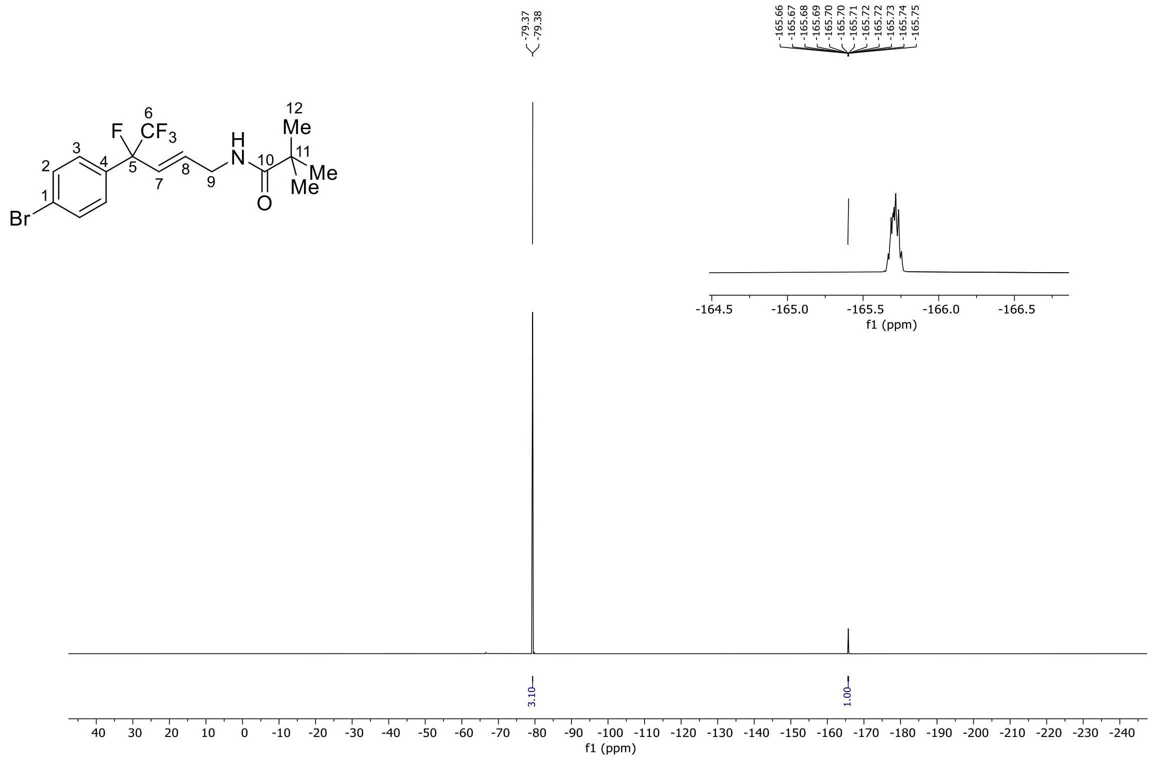

^{19}F{^{1}H} NMR (470 MHz, $CDCl_3$)

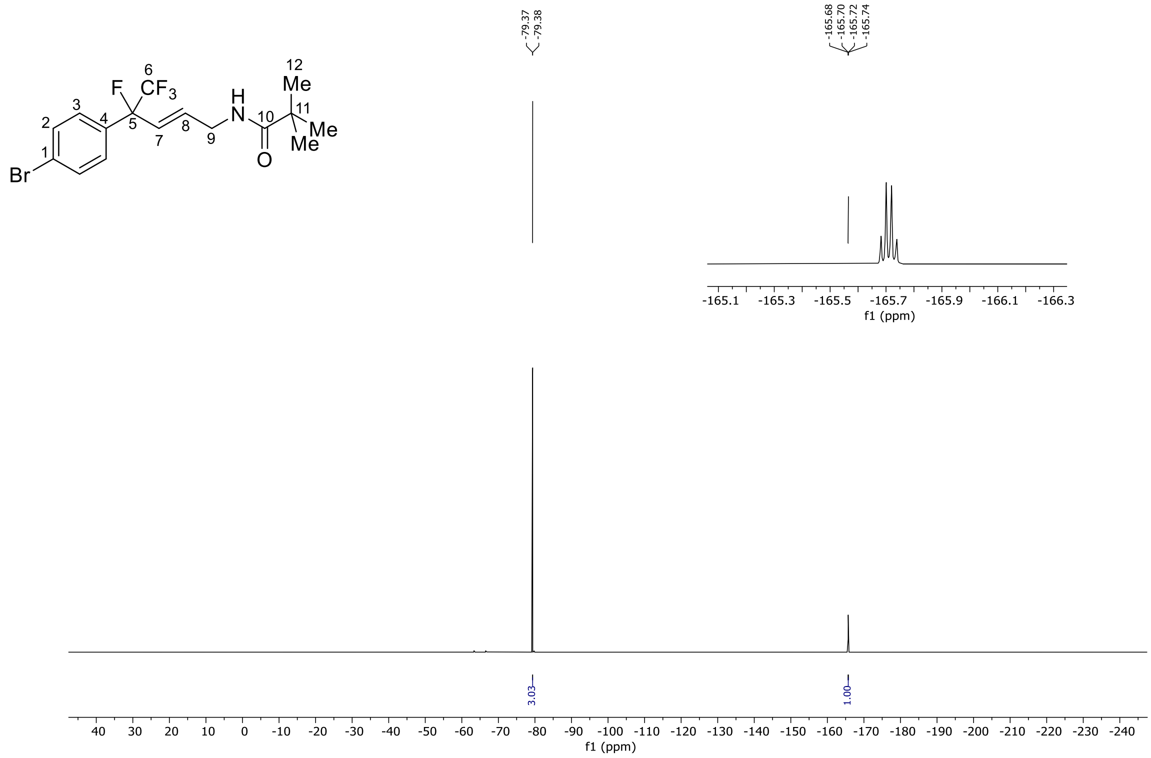

(*E*)-*N*-(4-(4-Bromophenyl)-4,5,5,5-tetrafluoropent-2-en-1-yl)benzamide (3e)

^{1}H NMR (500 MHz, $CDCl_3$)

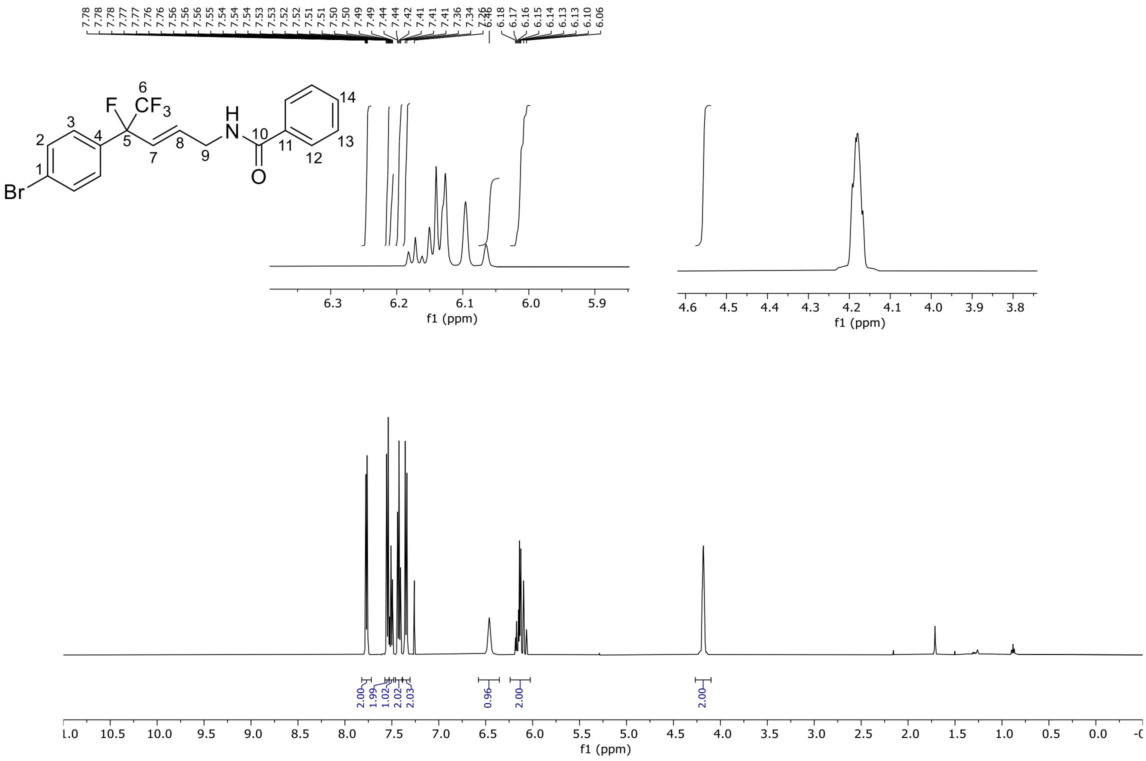

^{13}C NMR (126 MHz, $CDCl_3$)

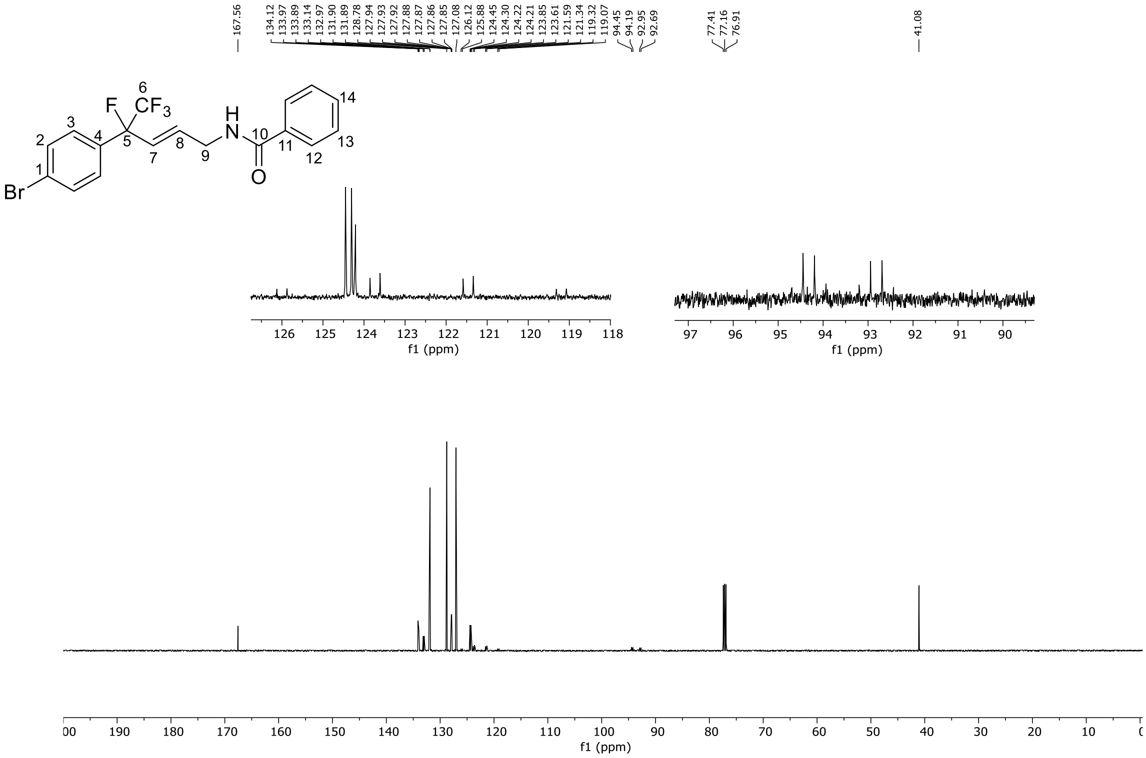

^{19}**F NMR** (470 MHz, $CDCl_3$)

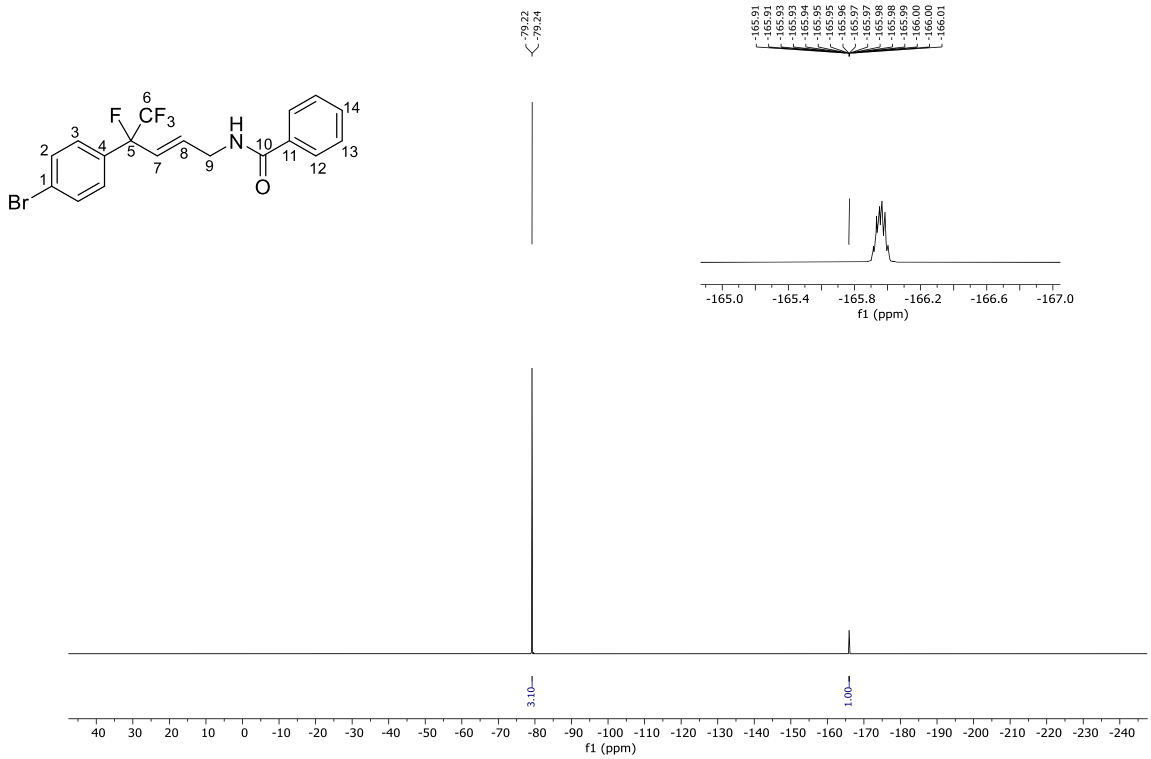

^{19}**F{^{1}H} NMR** (470 MHz, $CDCl_3$)

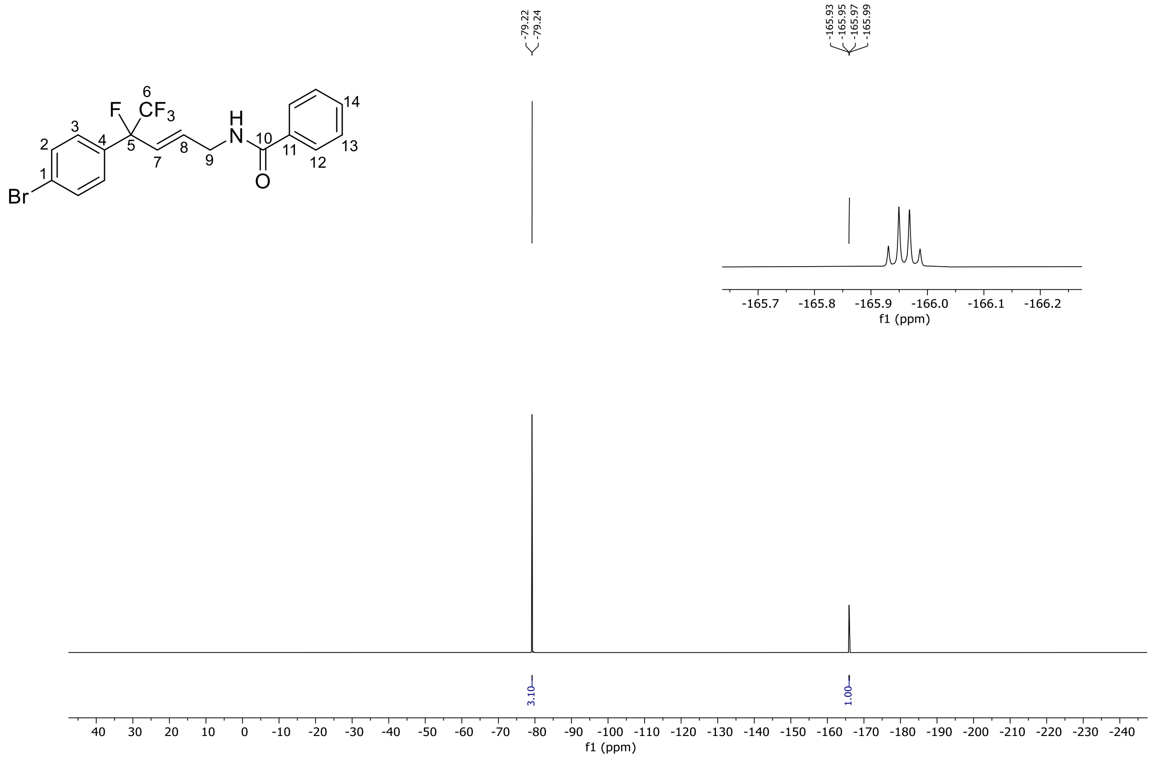

(*E*)-*N*-(4-(4-Bromophenyl)-4,5,5,5-tetrafluoropent-2-en-1-yl)acetamide-2,2,2-*d*$_3$ (3f)

^{1}H NMR (500 MHz, $CDCl_3$)

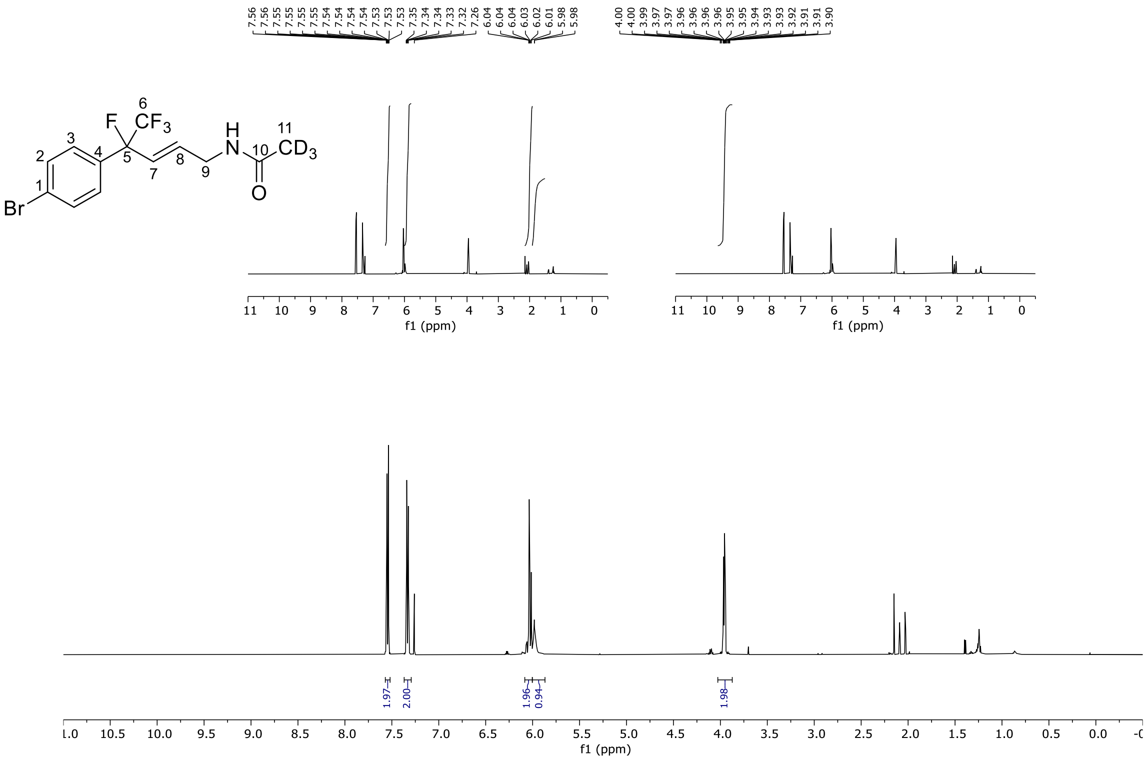

^{13}C NMR (126 MHz, $CDCl_3$)

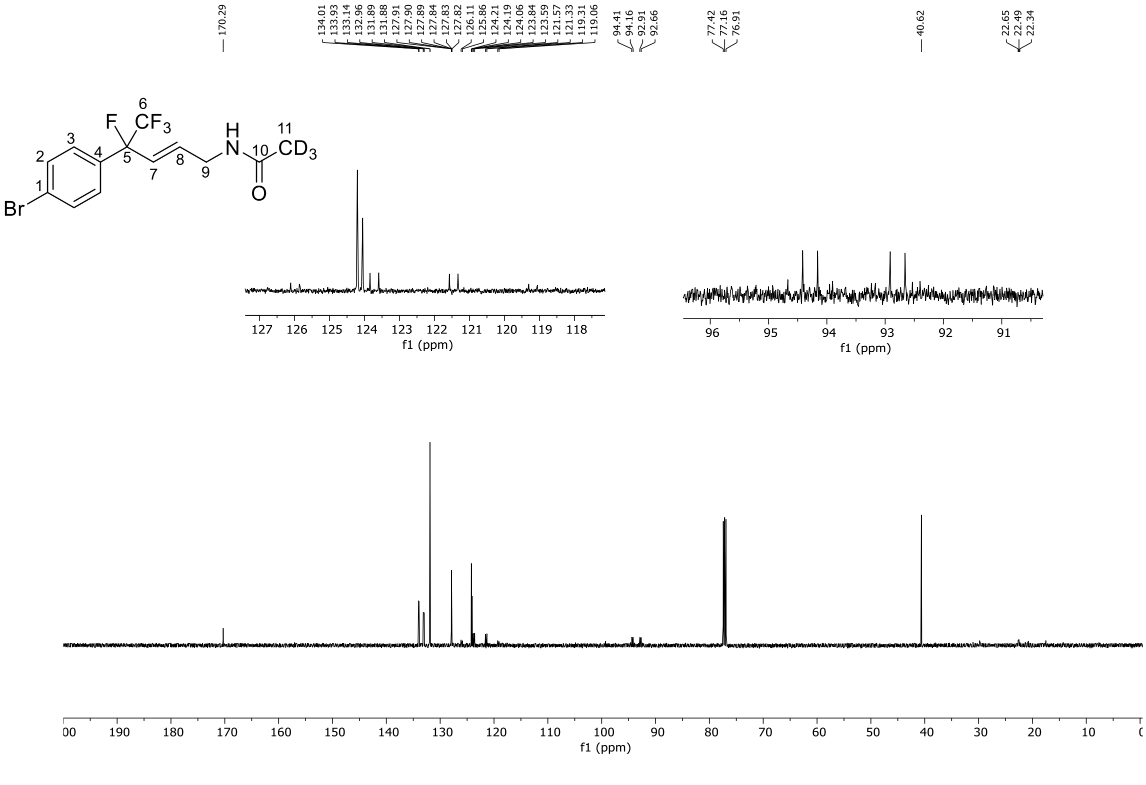

^{19}F NMR (564 MHz, $CDCl_3$)

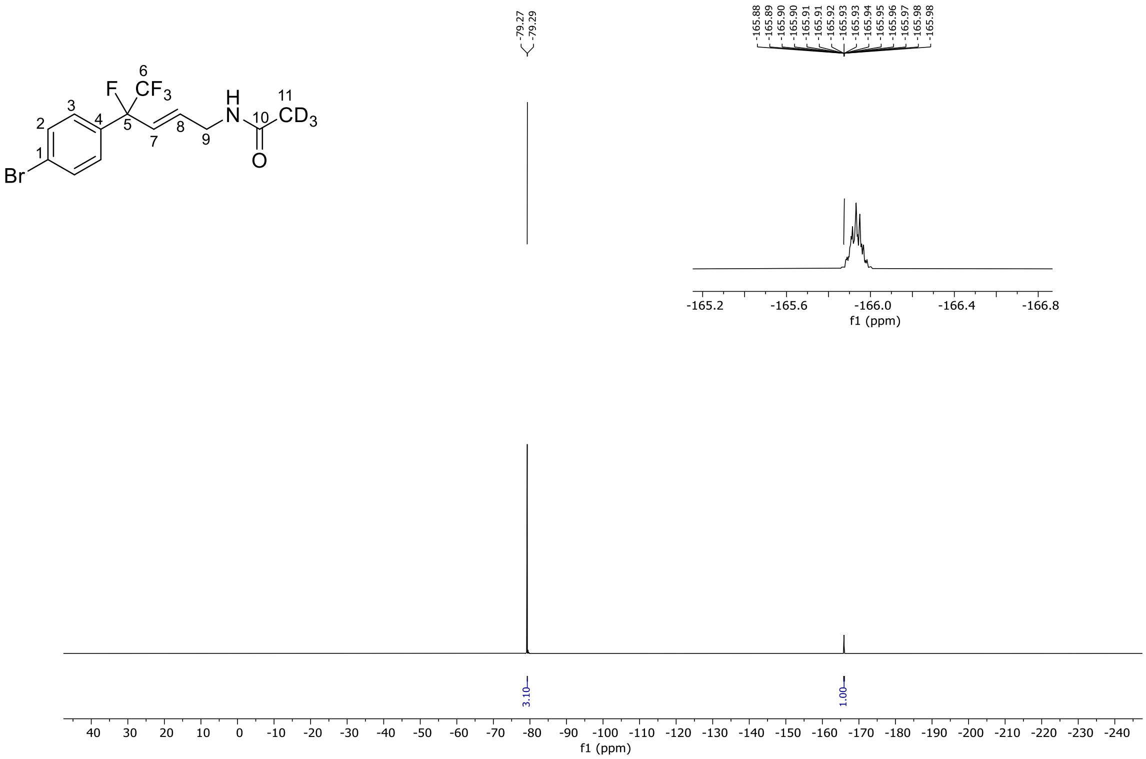

^{19}F{^{1}H} NMR (564 MHz, $CDCl_3$)

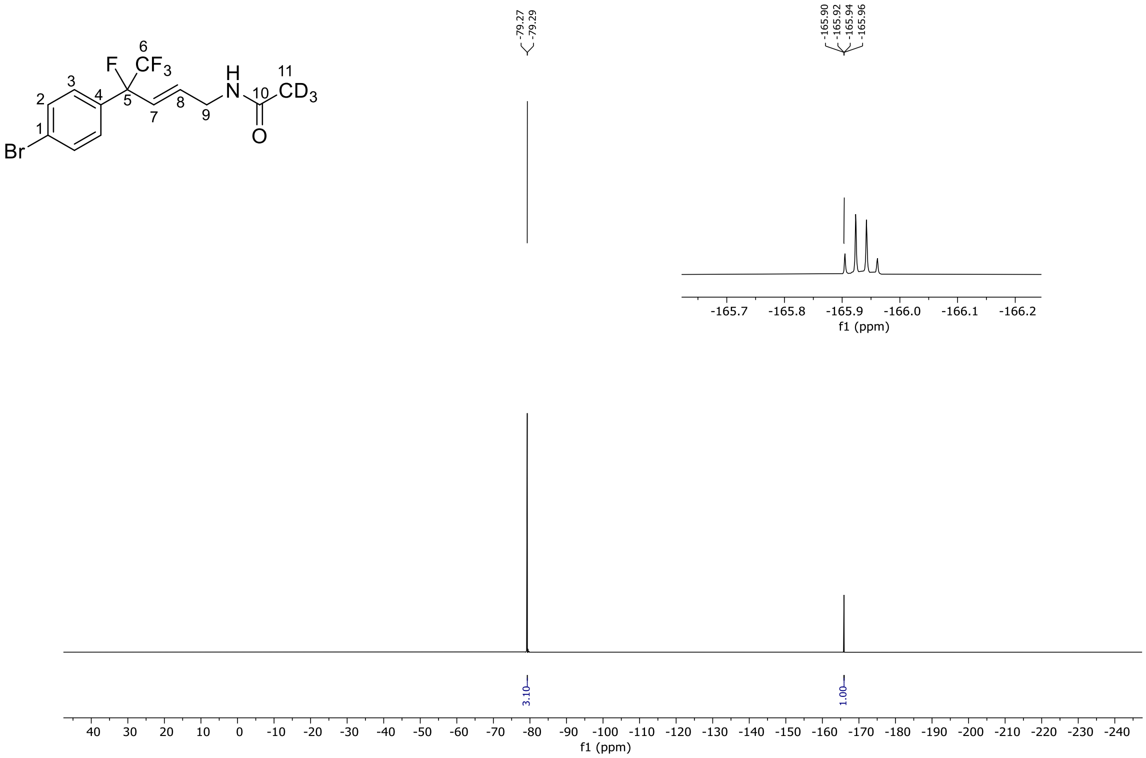

(*E*)-4-(4-Bromophenyl)-4,5,5,5-tetrafluoropent-2-en-1-yl formate (3g)

^{1}H NMR (500 MHz, $CDCl_3$)

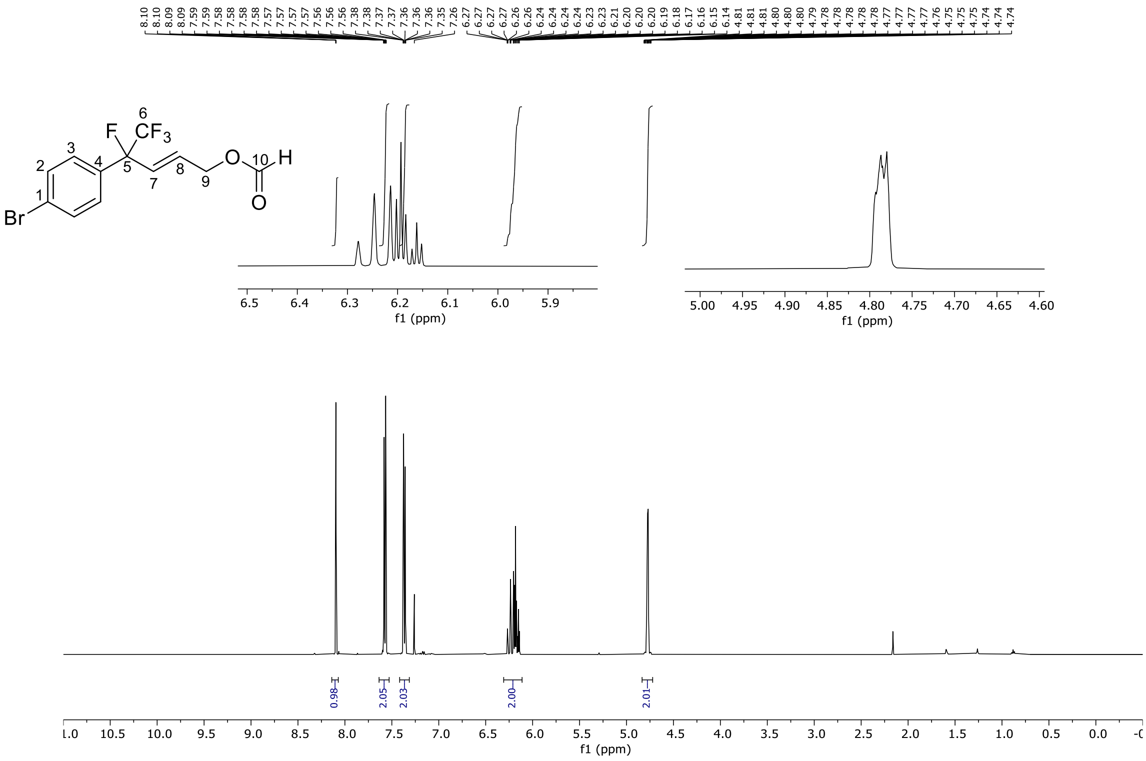

^{13}C NMR (126 MHz, $CDCl_3$)

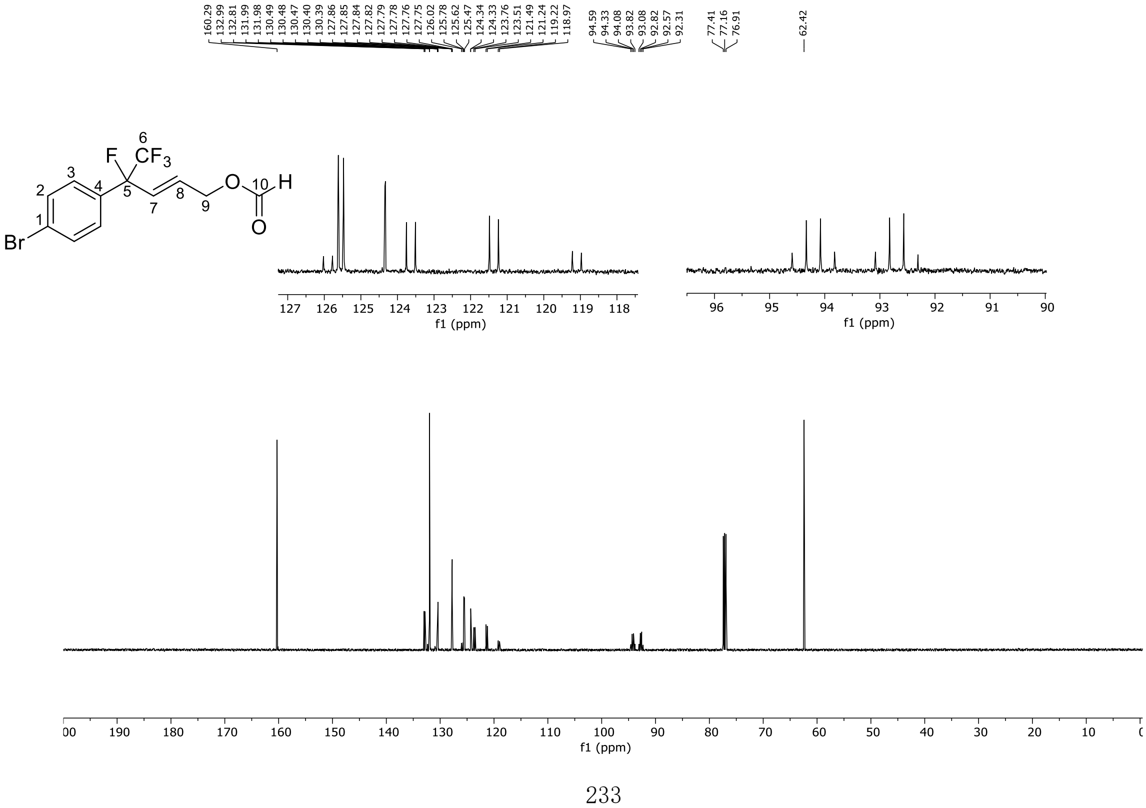

^{19}F NMR (470 MHz, $CDCl_3$)

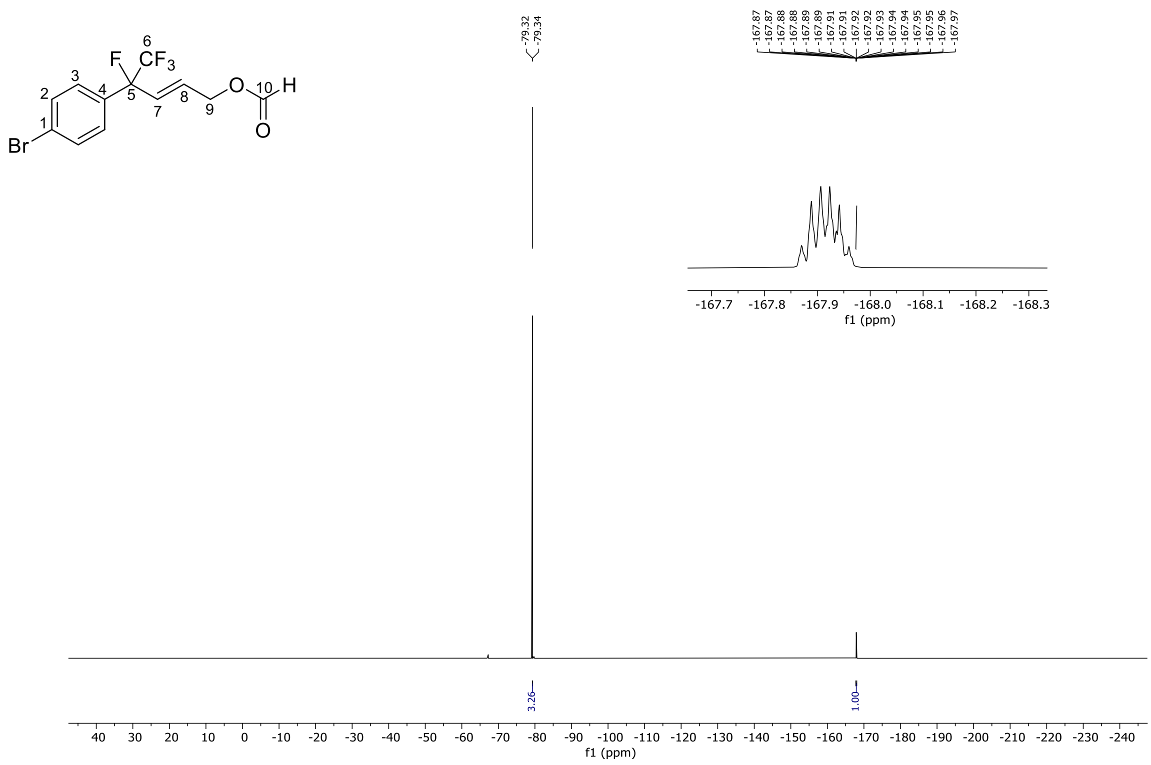

^{19}F{^{1}H} NMR (376 MHz, $CDCl_3$)

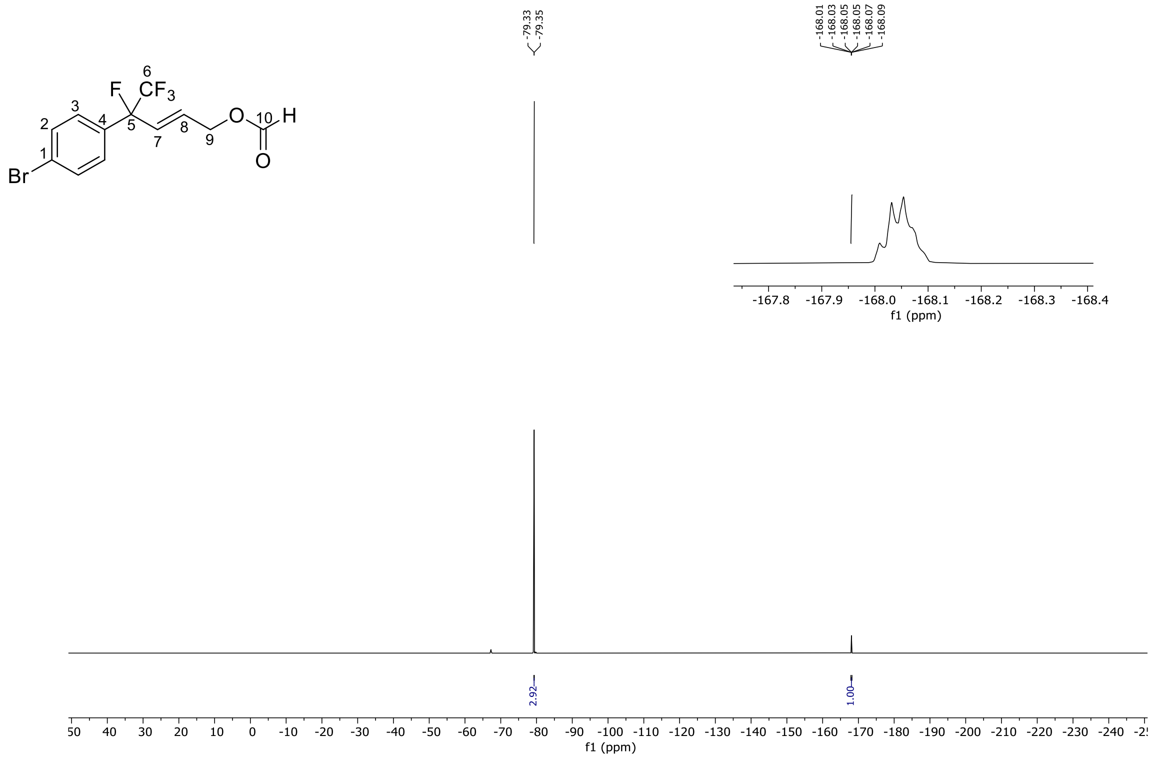

(*E*)-4-(4-Bromophenyl)-4,5,5,5-tetrafluoropent-2-en-1-yl acetate (3h)

^{1}H NMR (500 MHz, $CDCl_3$)

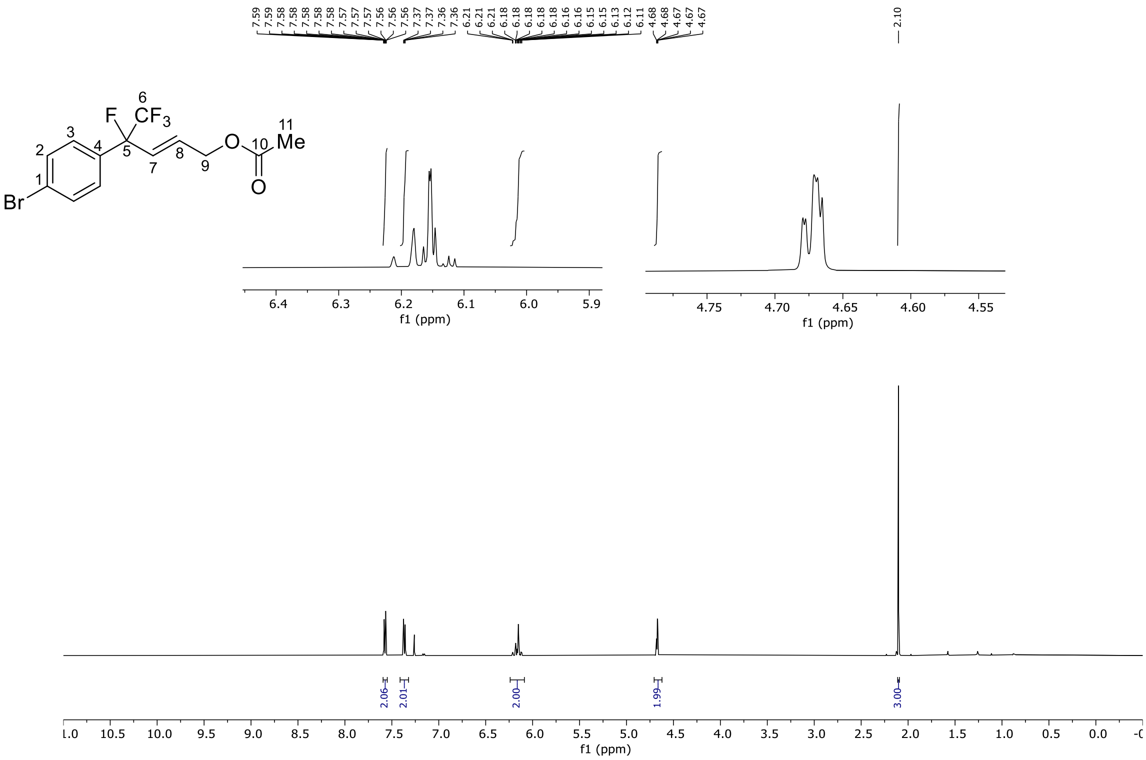

^{13}C NMR (126 MHz, $CDCl_3$)

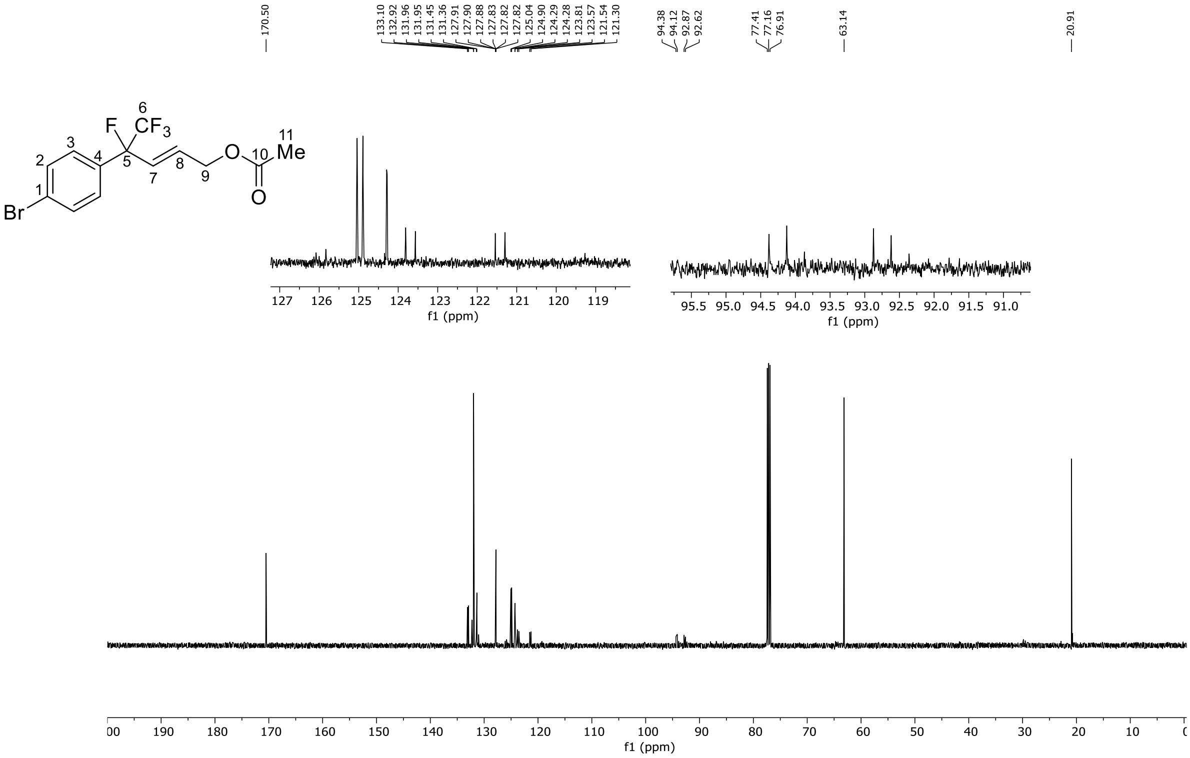

^{19}**F NMR** (470 MHz, $CDCl_3$)

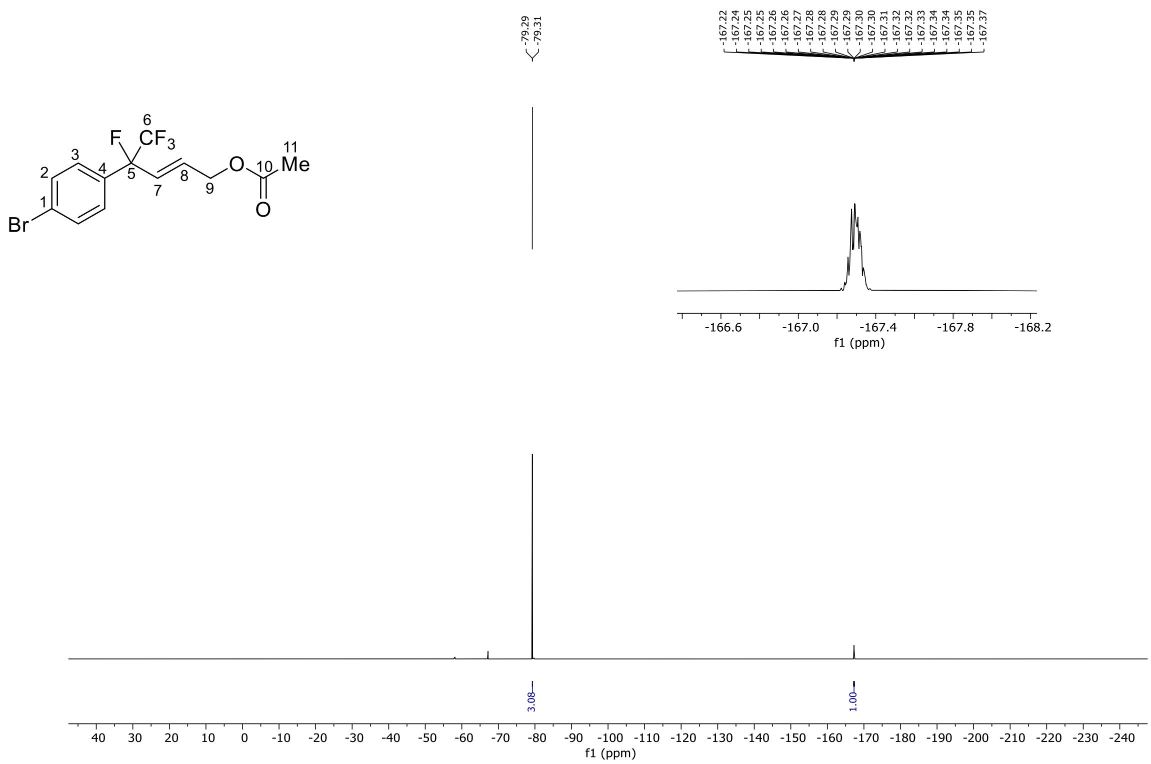

^{19}**F{**^{1}**H} NMR** (470 MHz, $CDCl_3$)

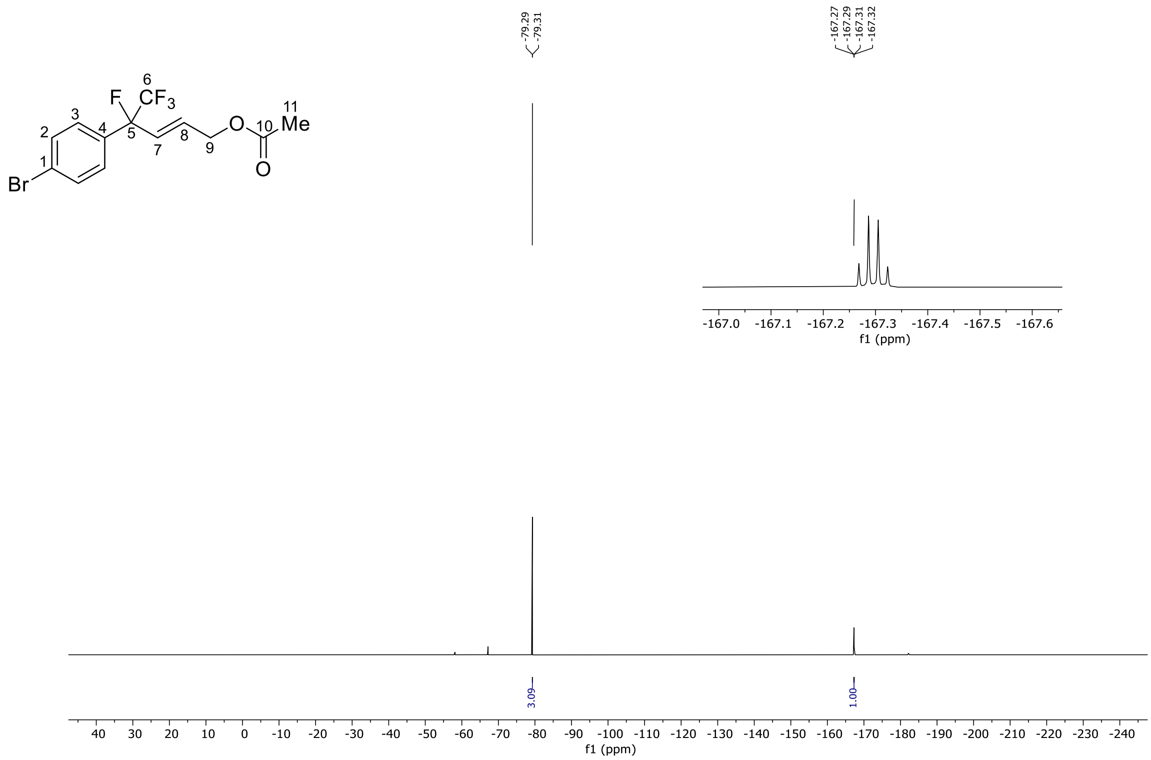

(*E*)-4-(4-Bromophenyl)-4,5,5,5-tetrafluoropent-2-en-1-yl propionate (3i)

^{1}H NMR (500 MHz, $CDCl_3$)

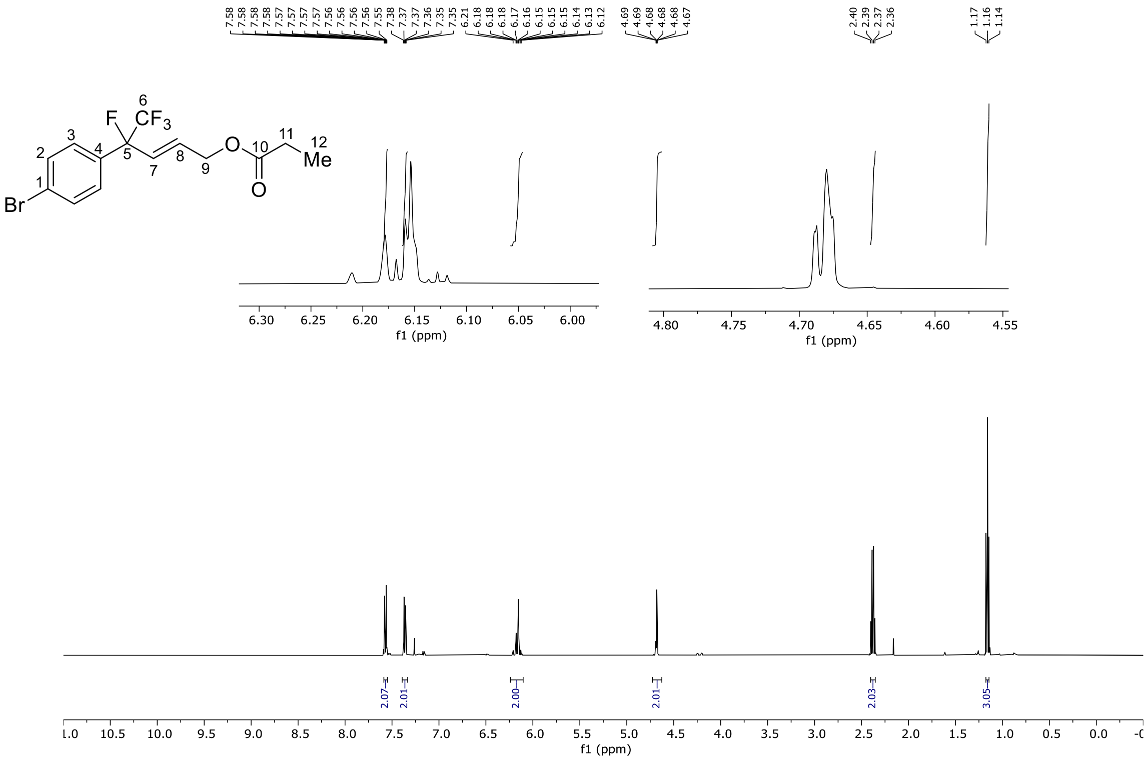

^{13}C NMR (126 MHz, $CDCl_3$)

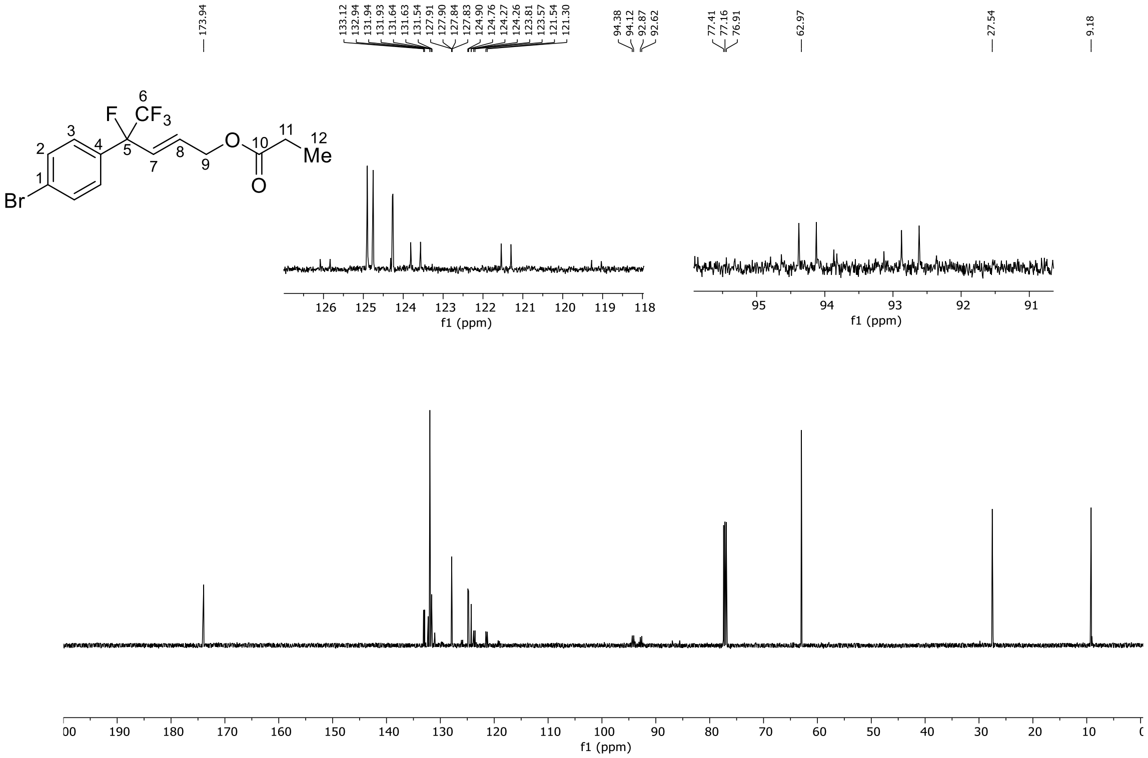

^{19}F NMR (470 MHz, $CDCl_3$)

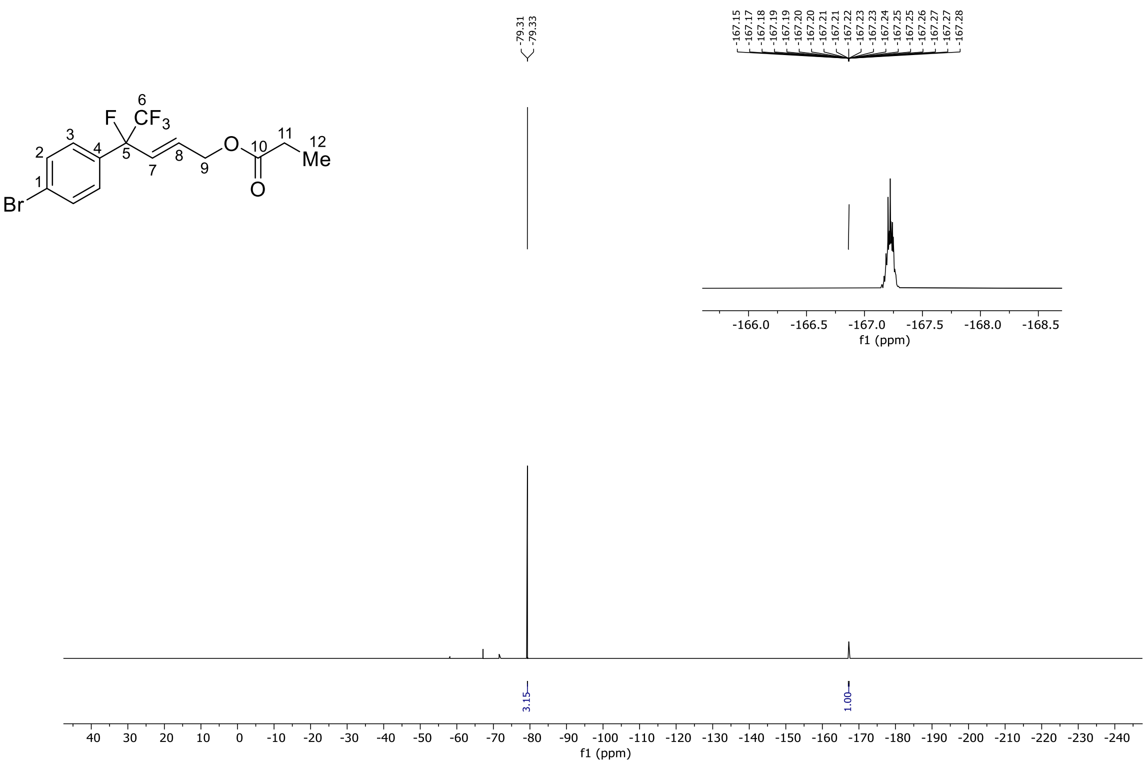

^{19}F{^{1}H} NMR (470 MHz, $CDCl_3$)

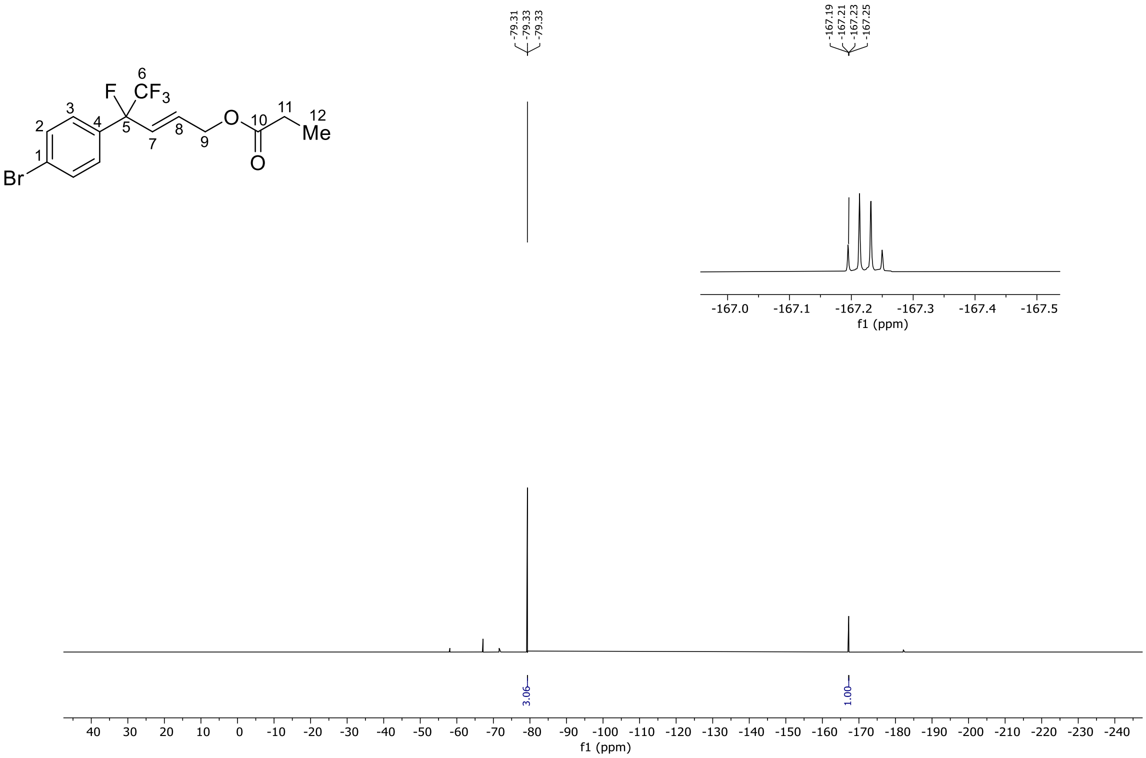

(*E*)-4-(4-Bromophenyl)-4,5,5,5-tetrafluoropent-2-en-1-yl hexanoate (3j)

^{1}H NMR (500 MHz, $CDCl_3$)

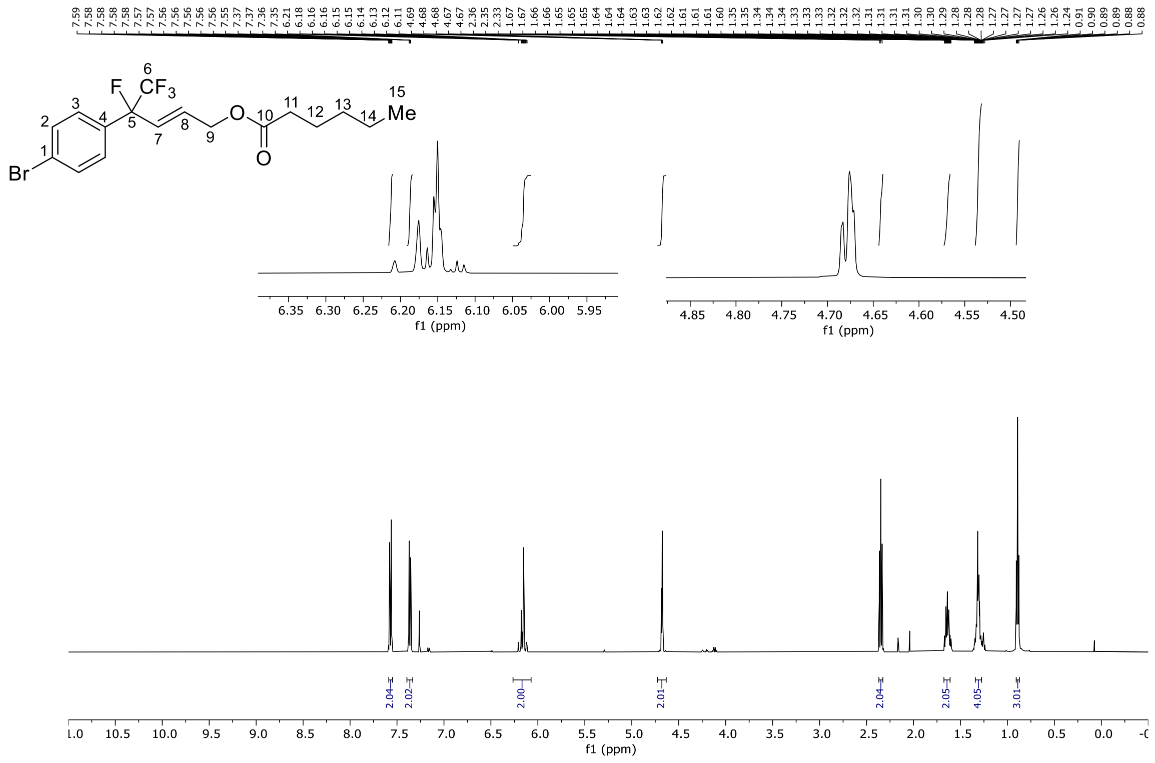

^{13}C NMR (126 MHz, $CDCl_3$)

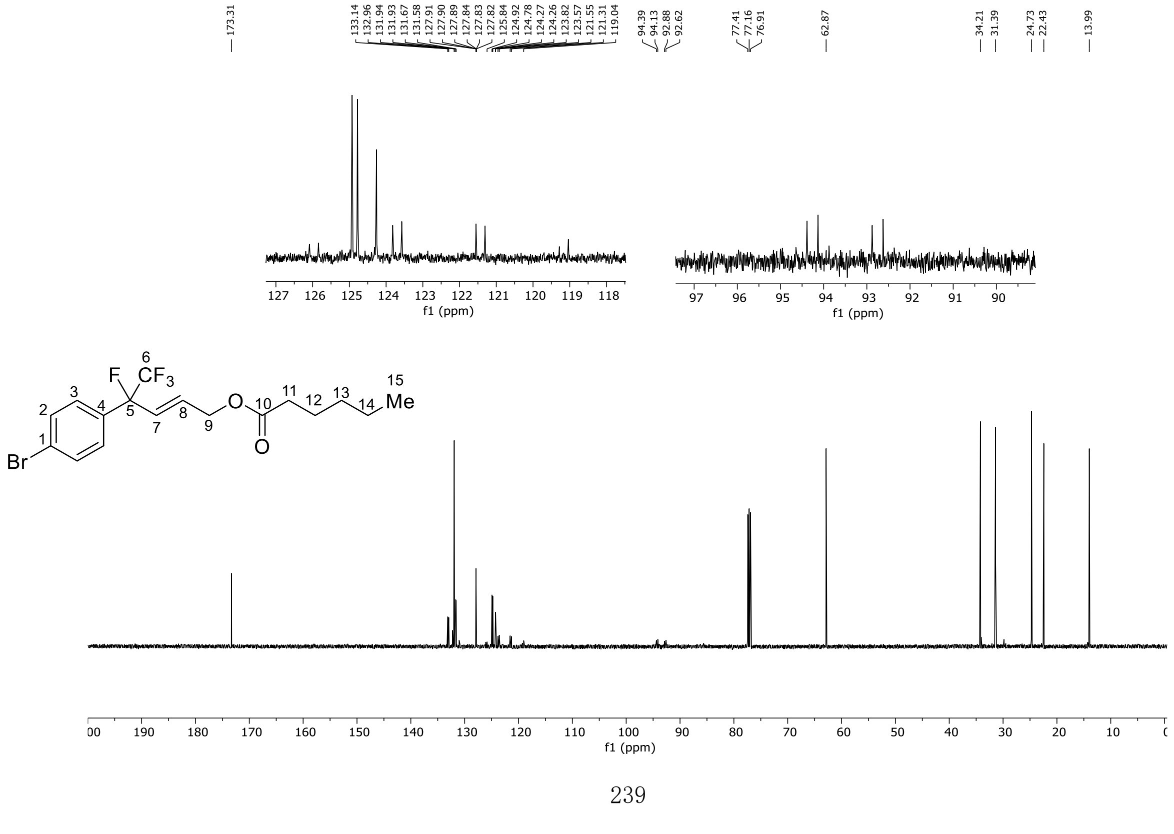

^{19}F NMR (470 MHz, $CDCl_3$)

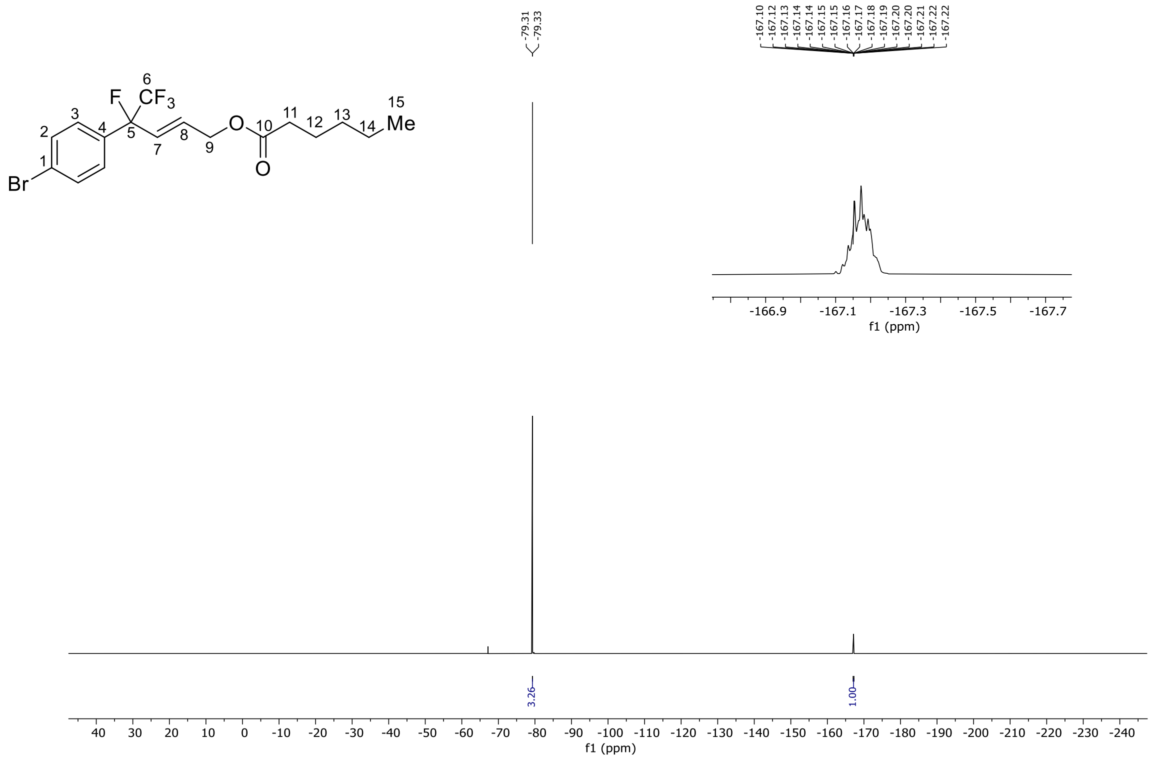

^{19}F{^{1}H} NMR (377 MHz, $CDCl_3$)

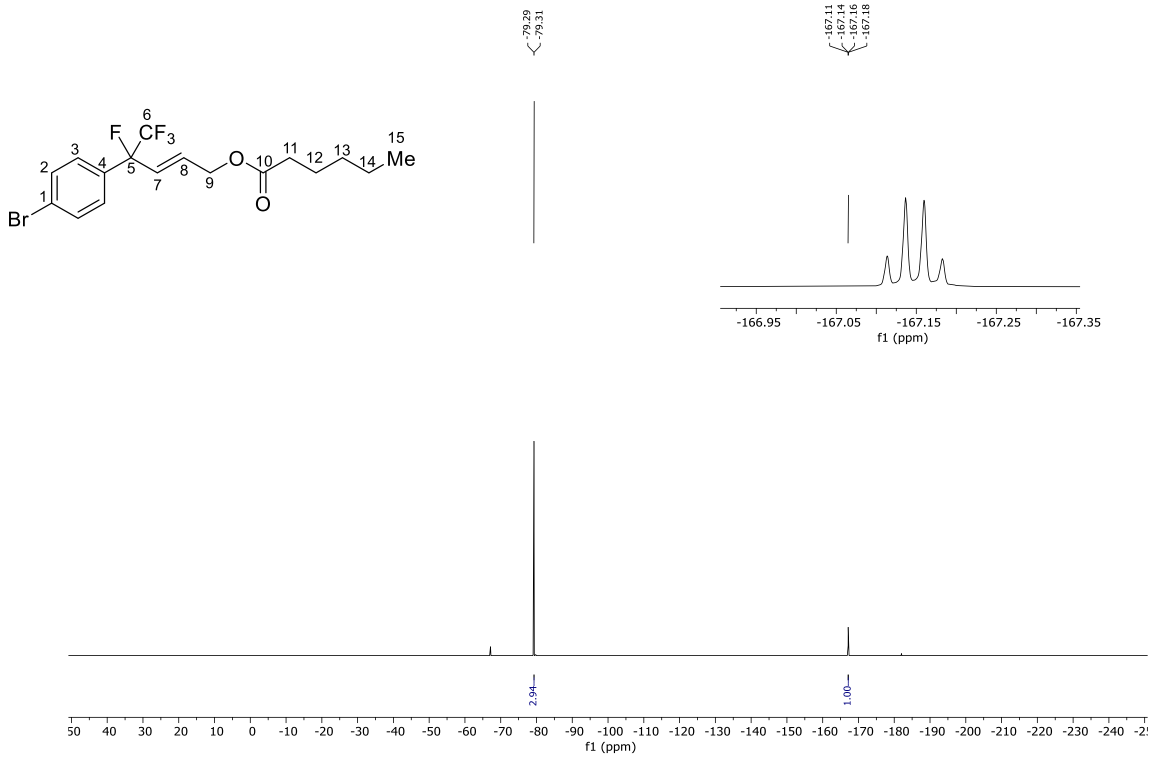

(*E*)-4-(4-Bromophenyl)-4,5,5,5-tetrafluoropent-2-en-1-yl cyclopropanecarboxylate (3k)

^{1}H NMR (500 MHz, $CDCl_3$)

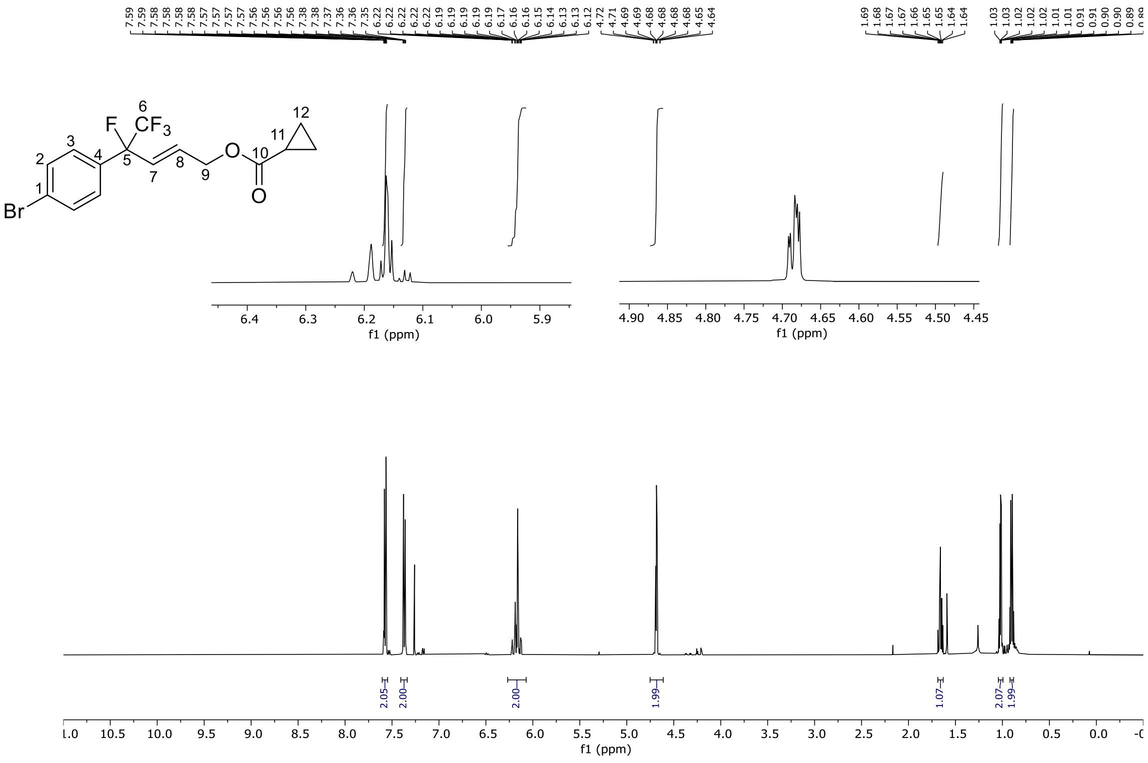

^{13}C NMR (126 MHz, $CDCl_3$)

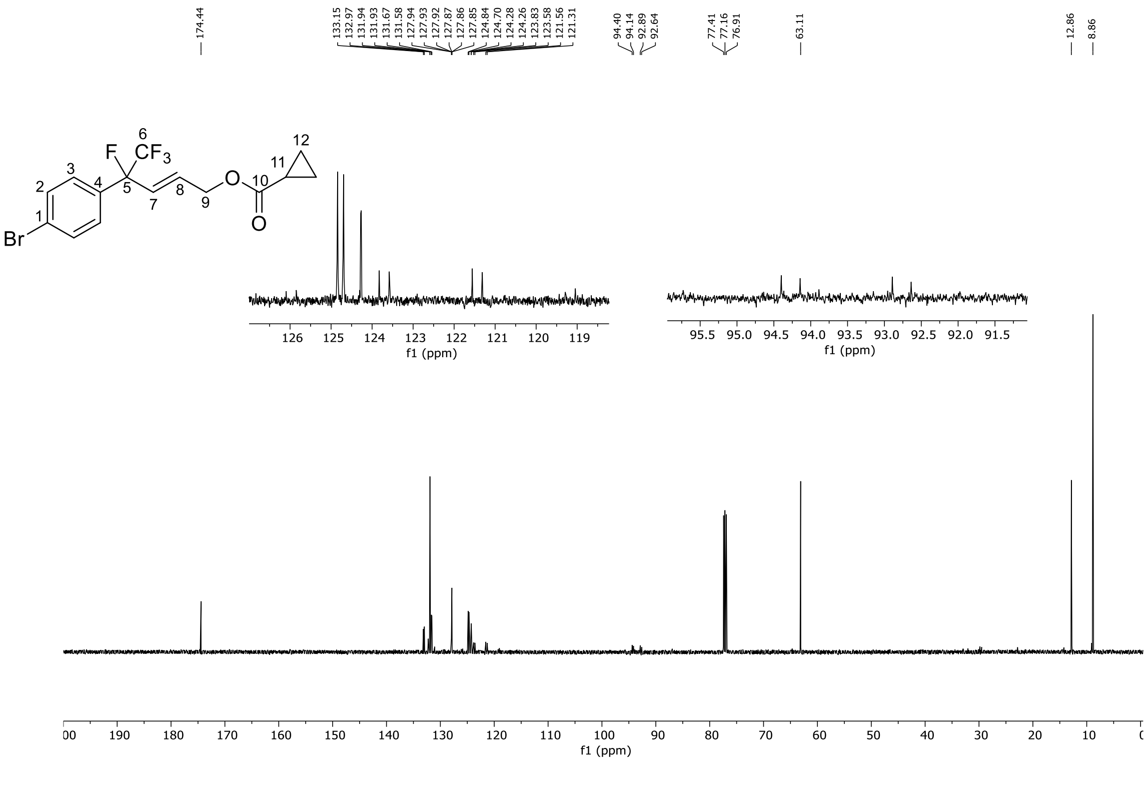

^{19}F NMR (470 MHz, $CDCl_3$)

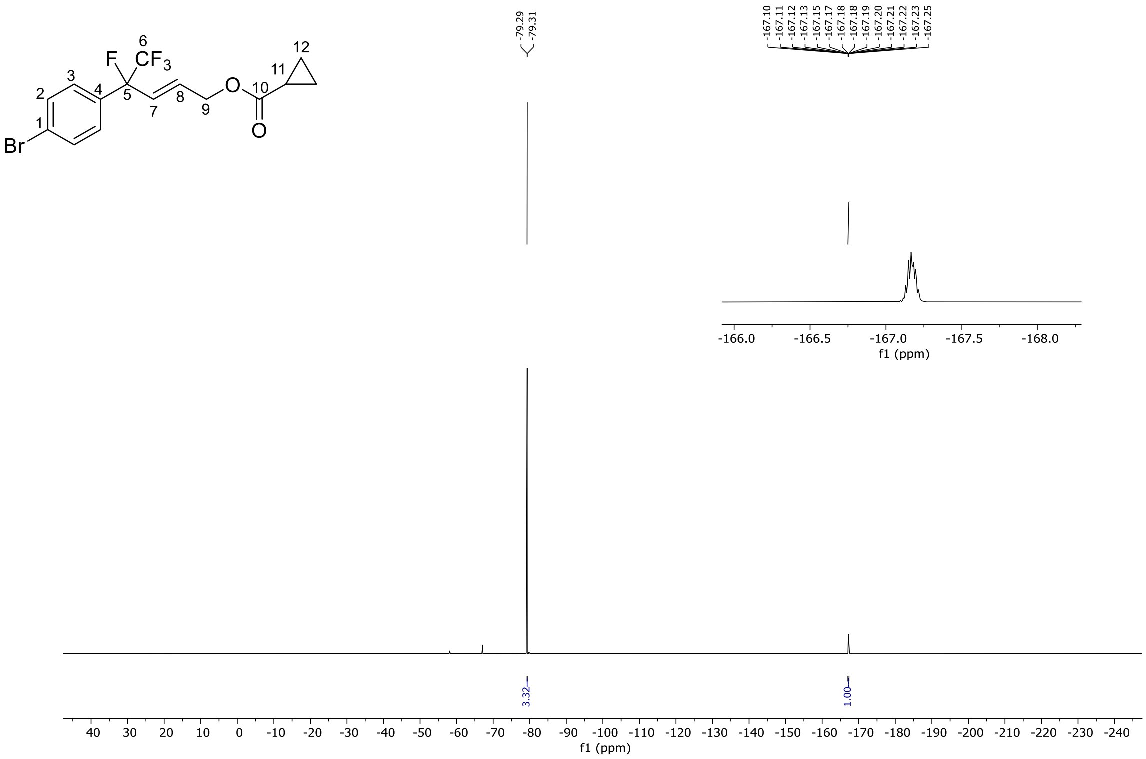

^{19}F{^{1}H} NMR (377 MHz, $CDCl_3$)

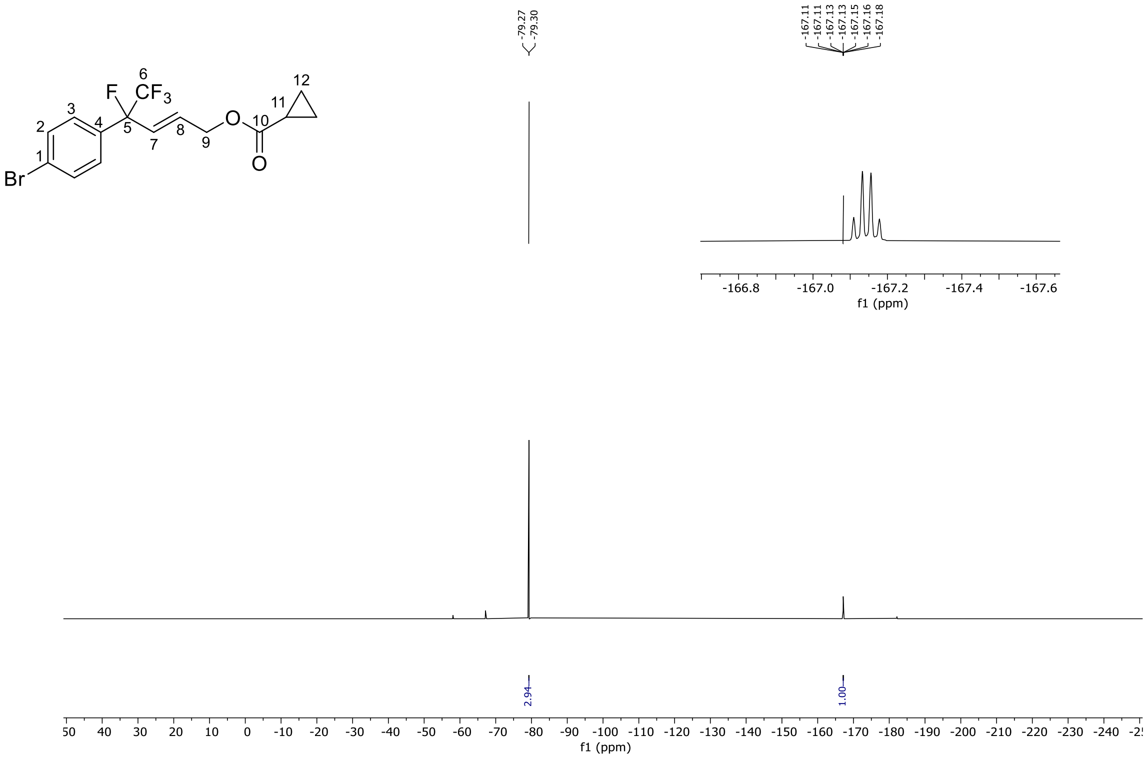

(*E*)-4-(4-Bromophenyl)-4,5,5,5-tetrafluoropent-2-en-1-yl 3-oxocyclobutane-1-carboxylate (3l)

^{1}H NMR (500 MHz, $CDCl_3$)

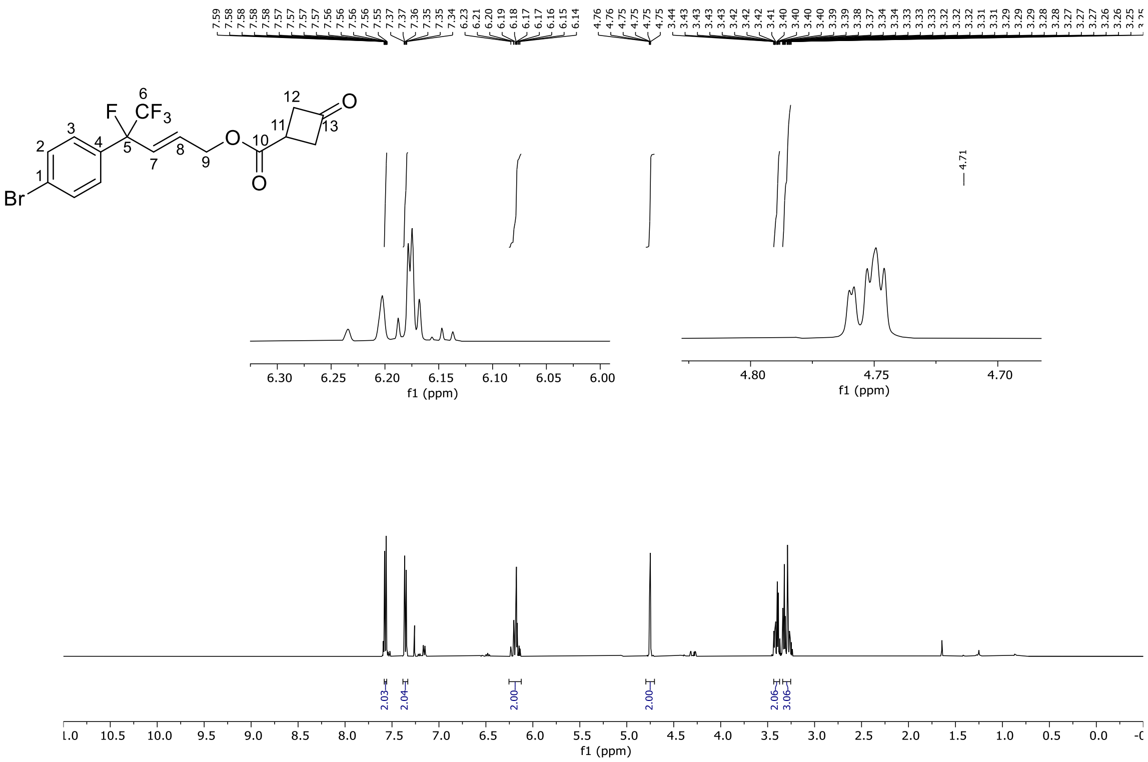

^{13}C NMR (126 MHz, $CDCl_3$)

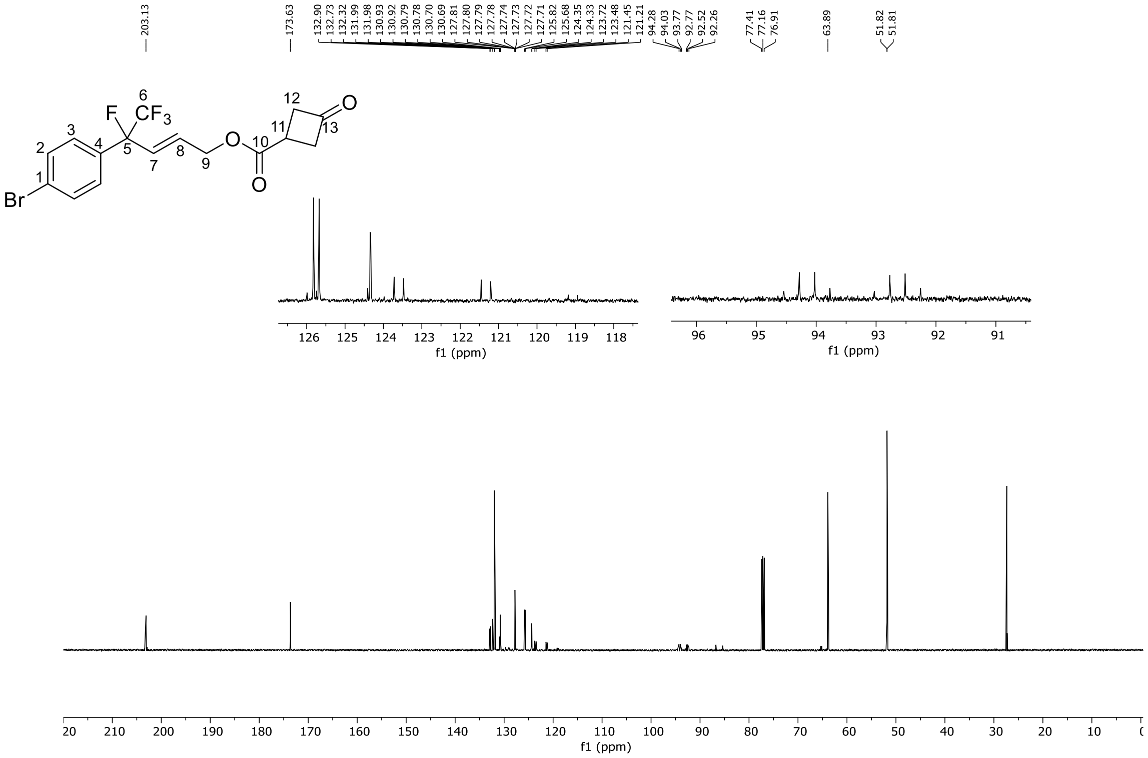

^{19}F NMR (470 MHz, $CDCl_3$)

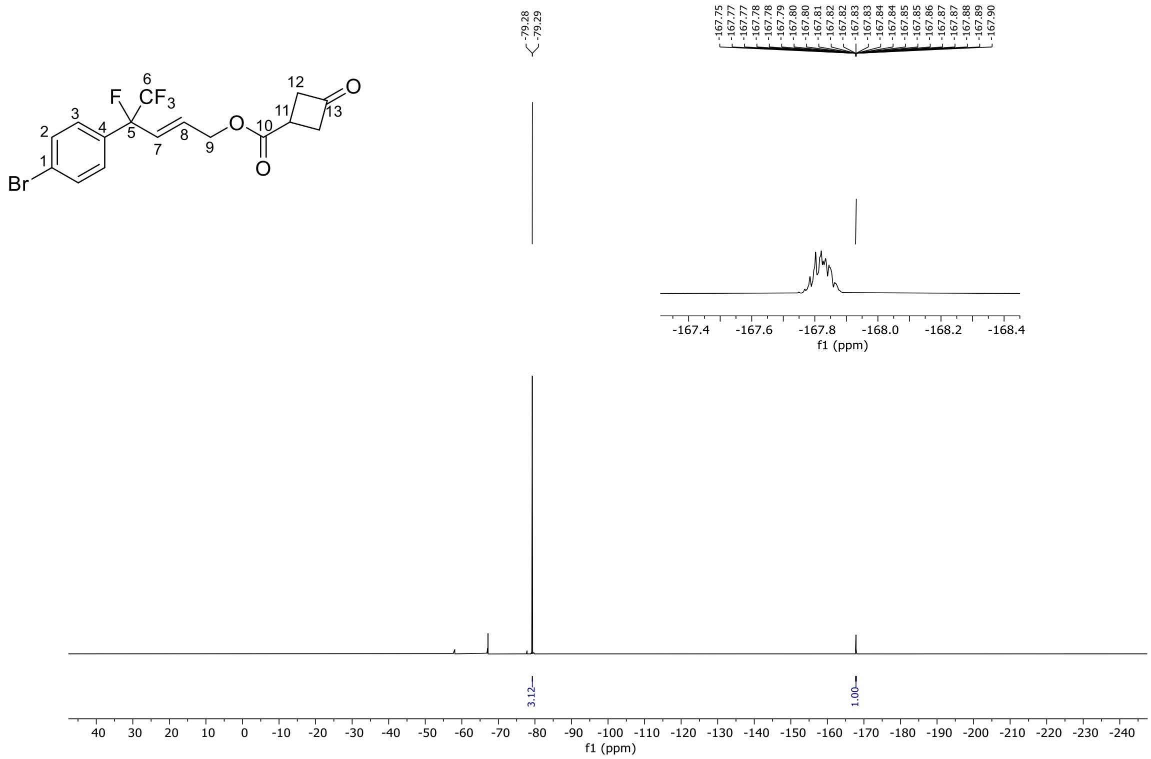

^{19}F{^{1}H} NMR (470 MHz, $CDCl_3$)

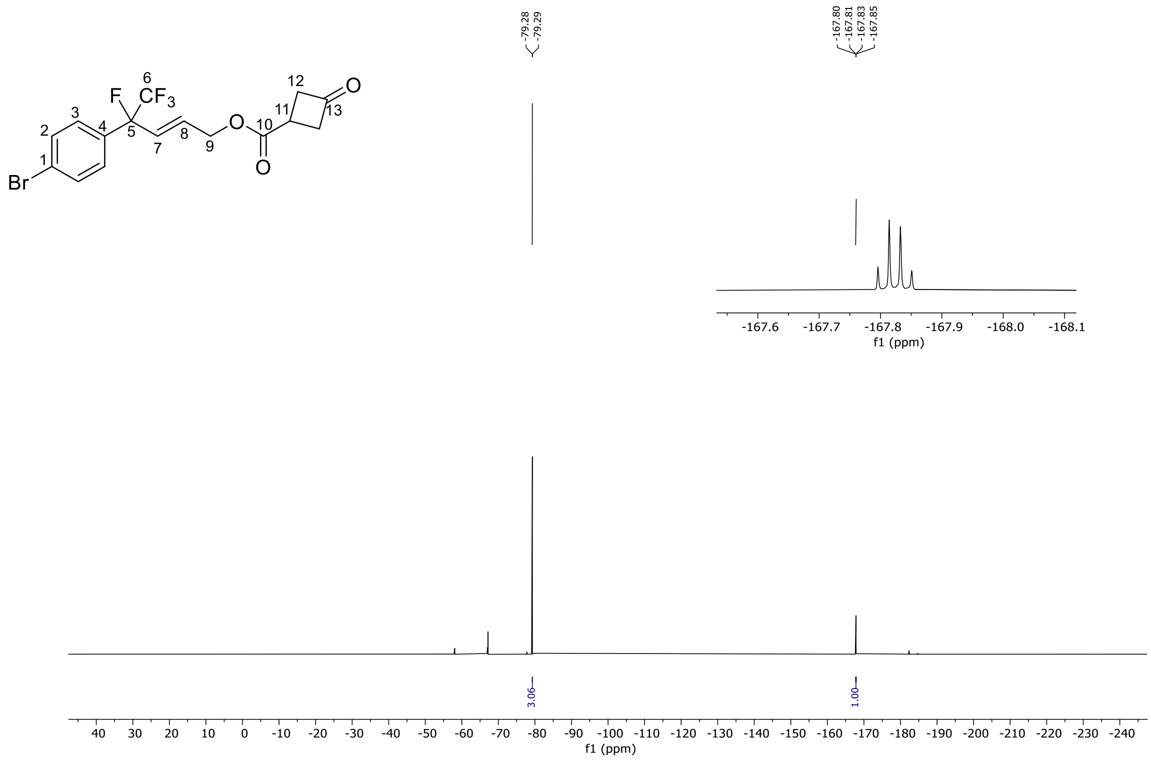

(*E*)-1-Bromo-4-(1,1,1,2-tetrafluoro-5-methoxypent-3-en-2-yl)benzene (3m)

^{1}H NMR (400 MHz, $CDCl_3$)

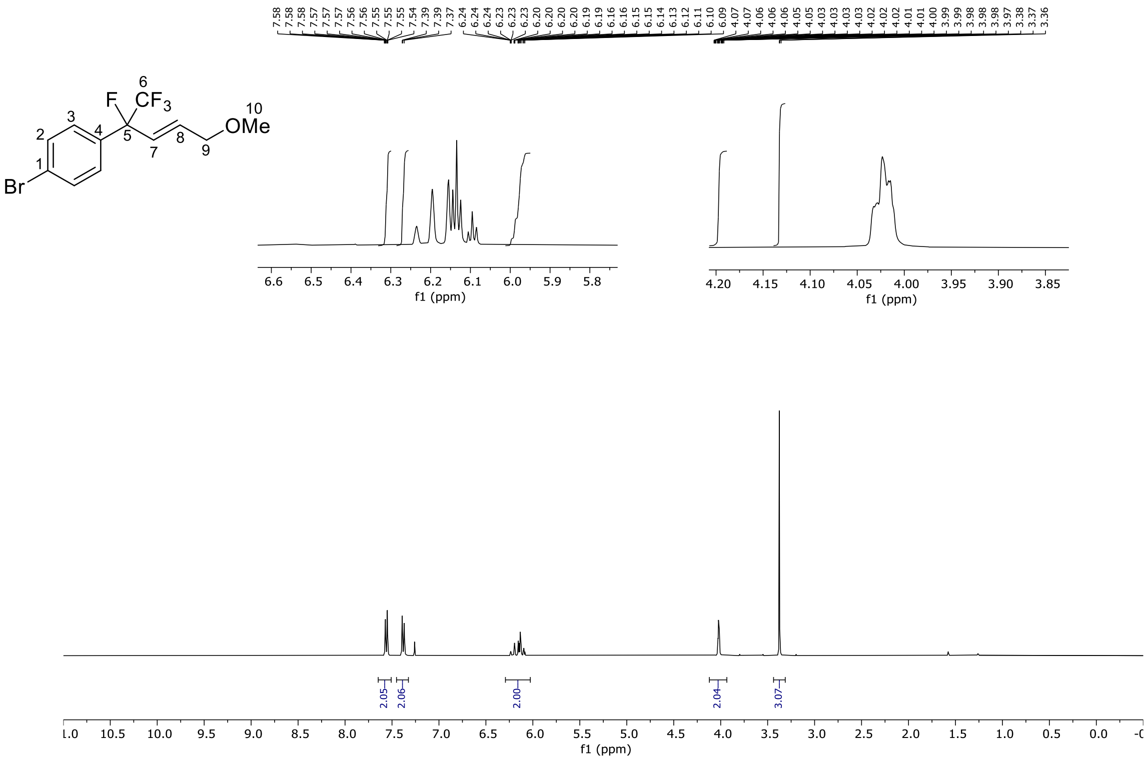

^{13}C NMR (101MHz, $CDCl_3$)

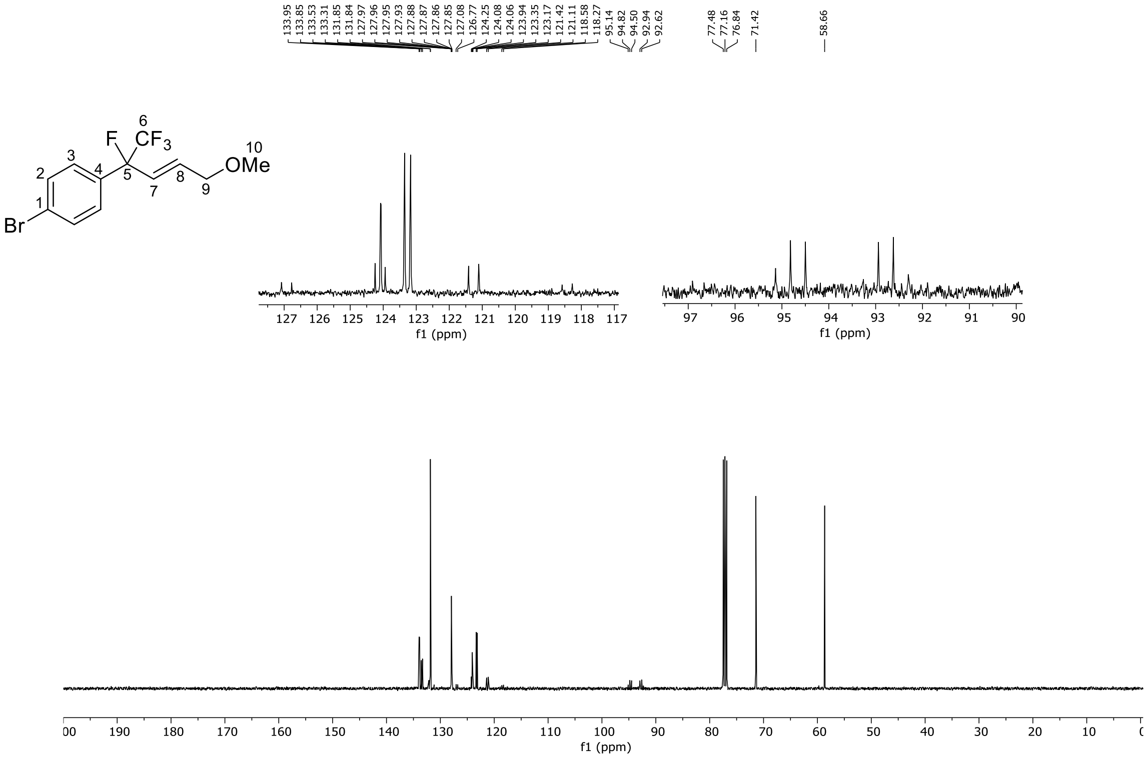

^{19}F NMR (470 MHz, $CDCl_3$)

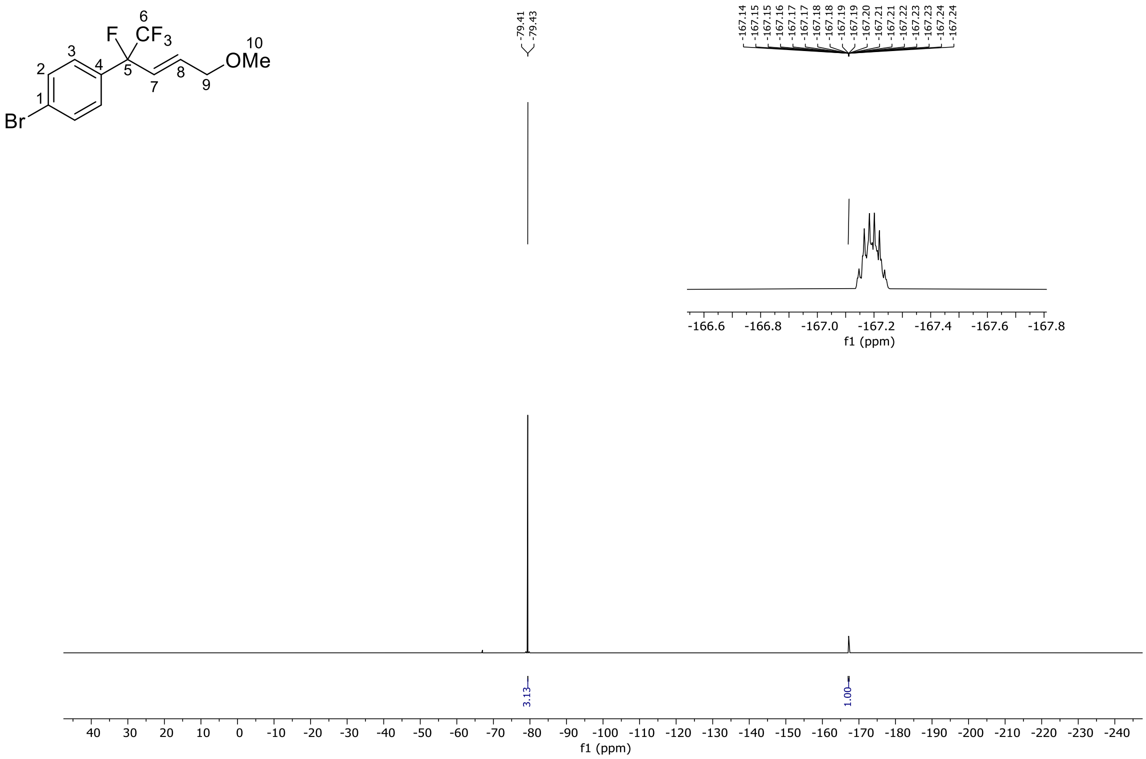

^{19}F{^{1}H} NMR (470 MHz, $CDCl_3$)

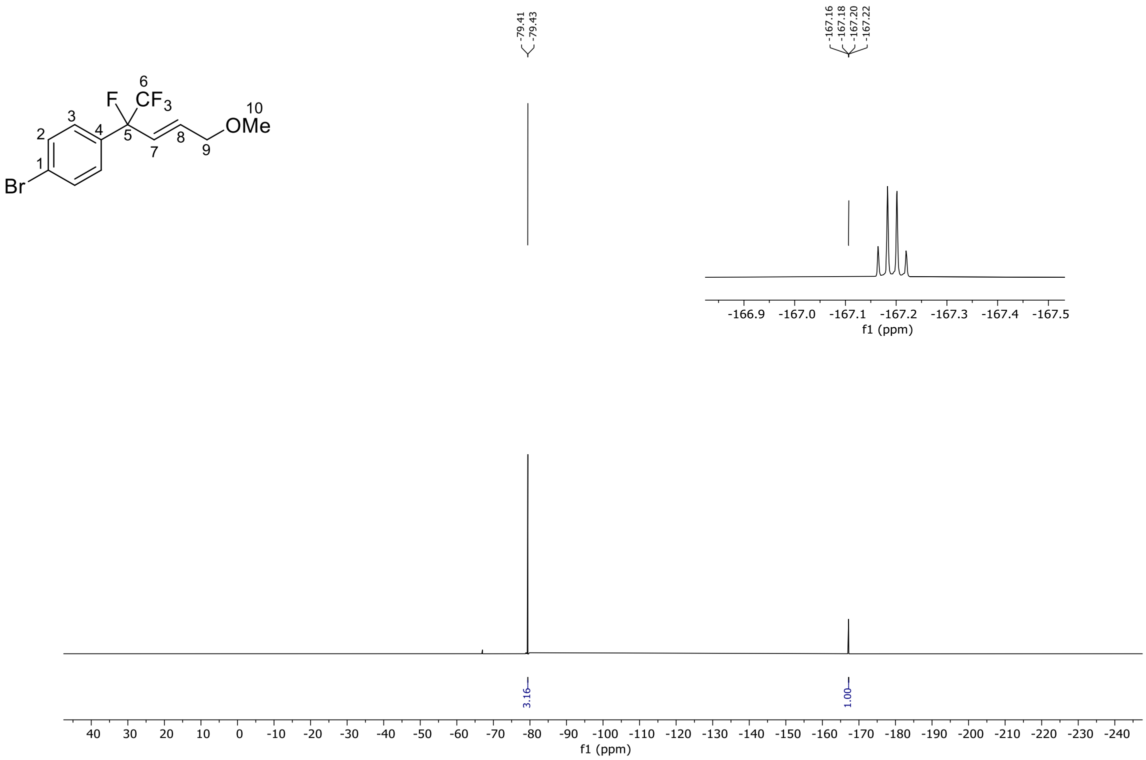

(*E*)-1-Bromo-4-(5-ethoxy-1,1,1,2-tetrafluoropent-3-en-2-yl)benzene (3n)

^{1}H NMR (500 MHz, $CDCl_3$)

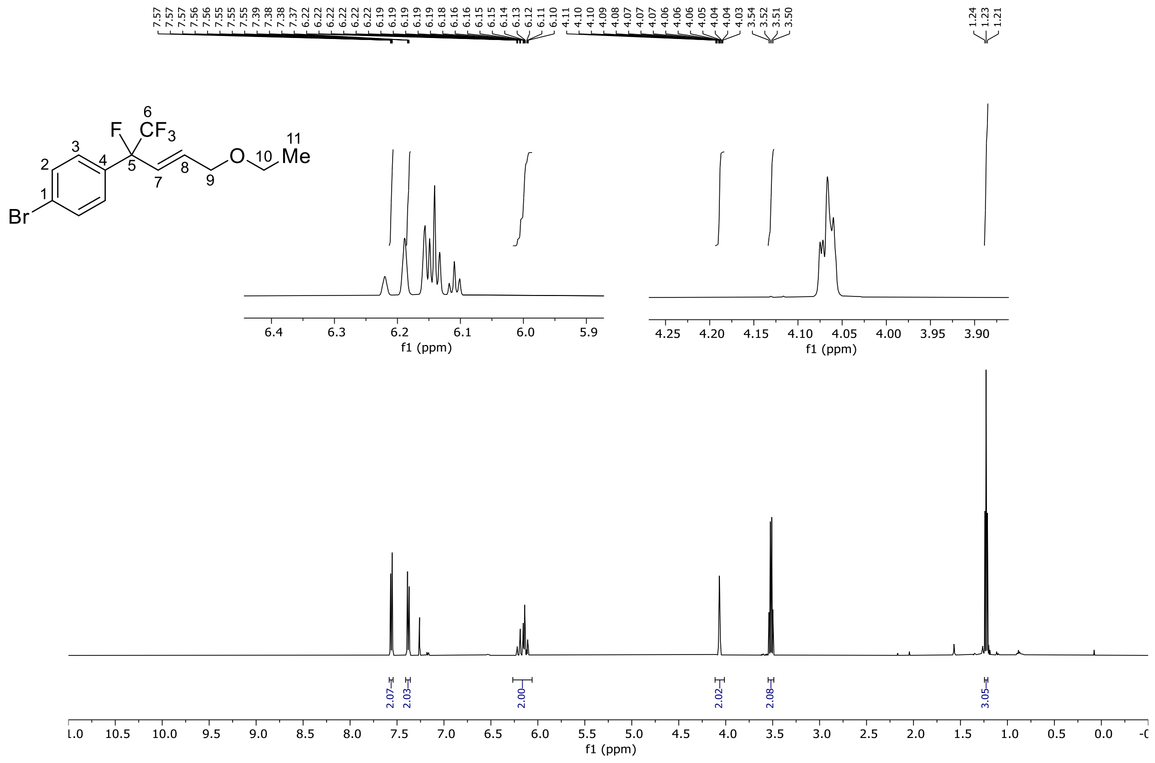

^{13}C NMR (126MHz, $CDCl_3$)

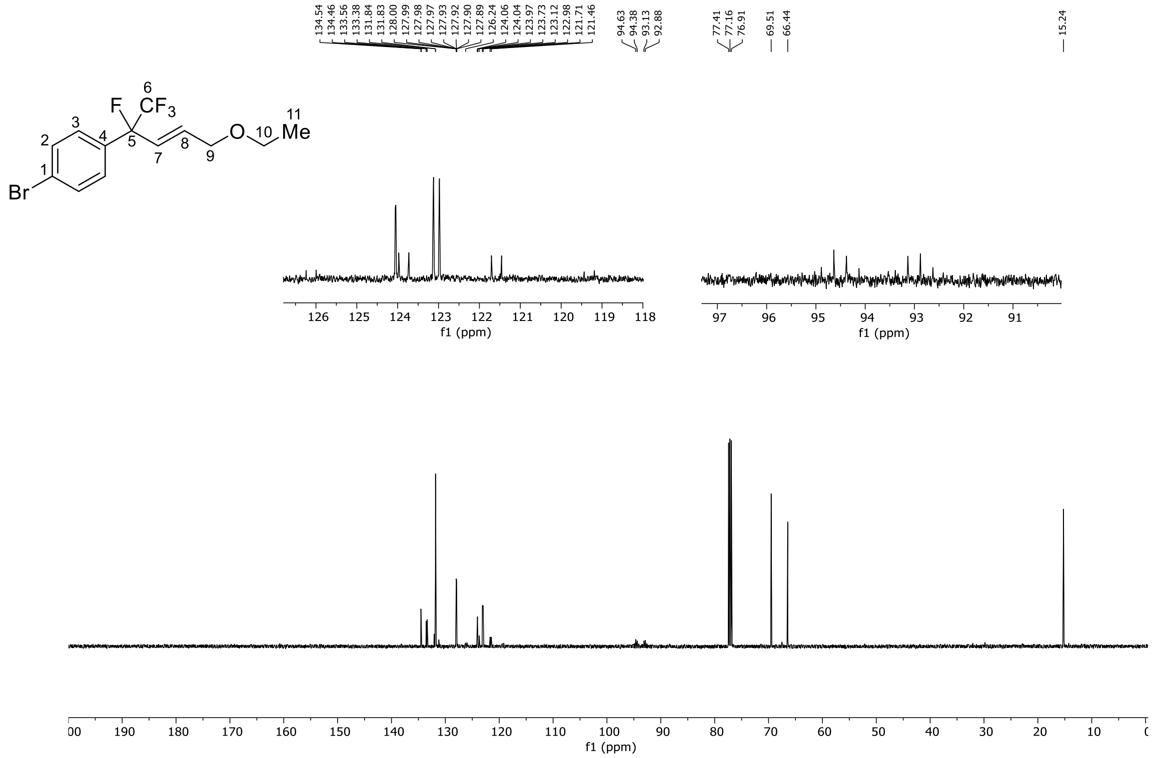

^{19}**F NMR** (470 MHz, $CDCl_3$)

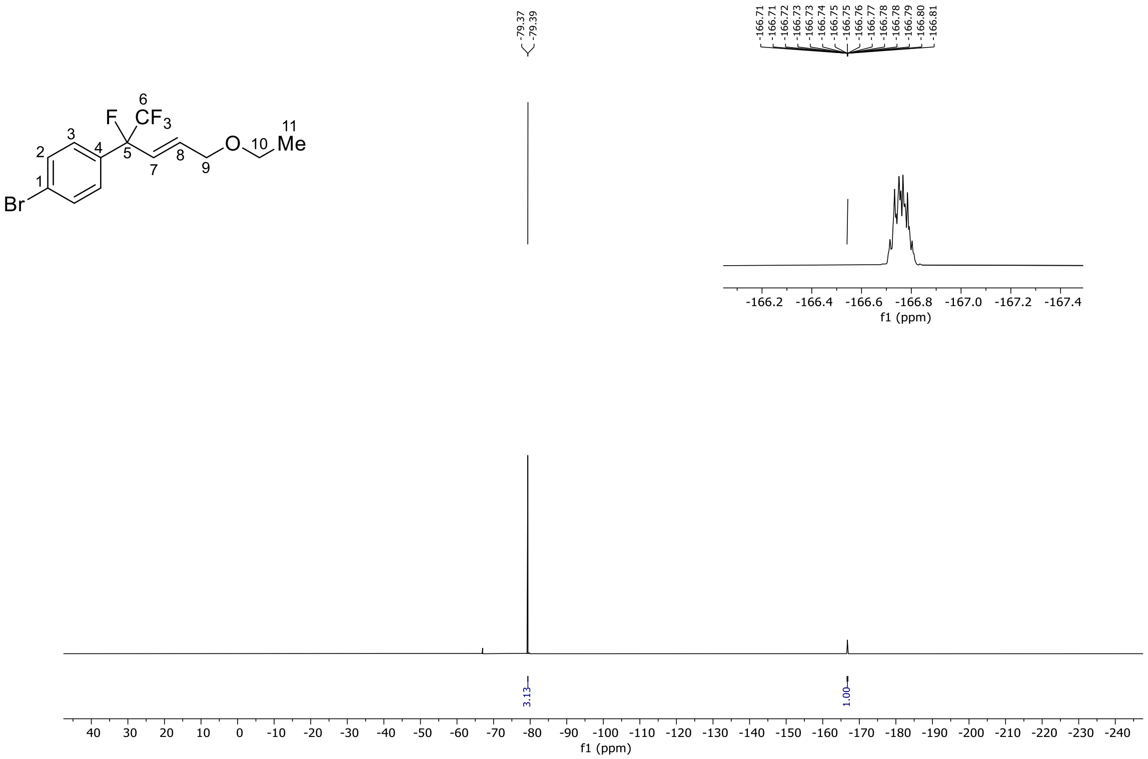

^{19}**F{^{1}H} NMR** (470 MHz, $CDCl_3$)

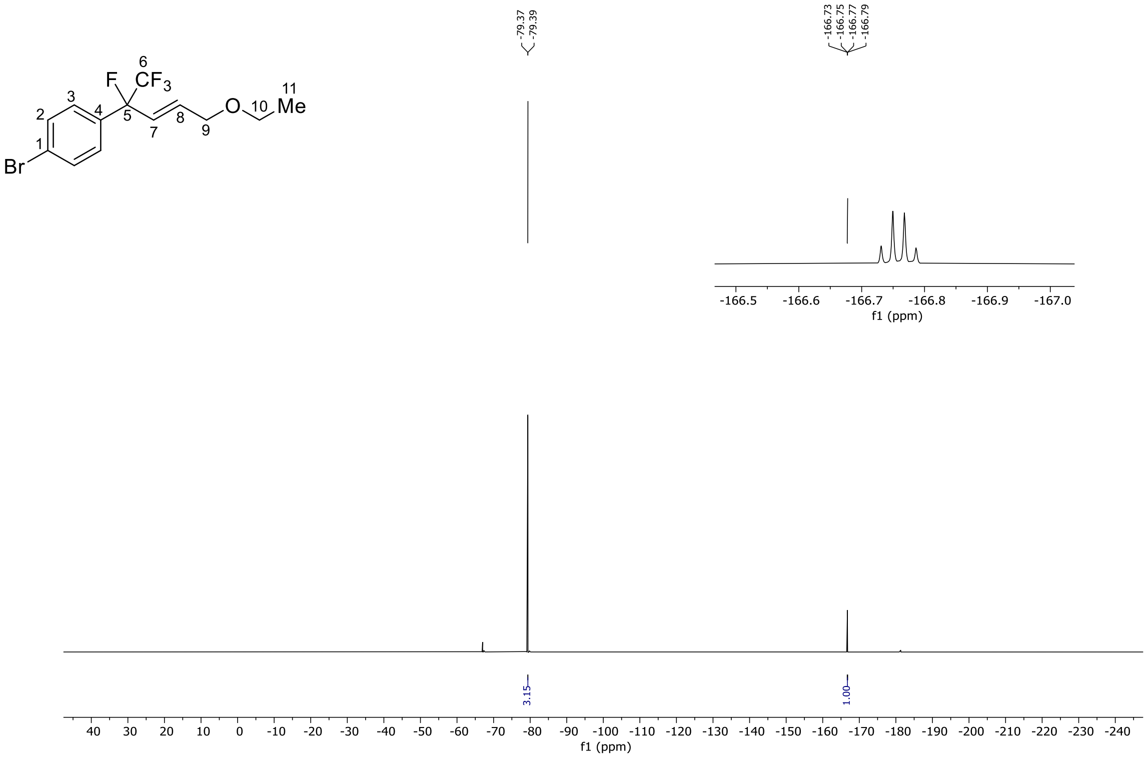

(*E*)-1-Bromo-4-(1,1,1,2-tetrafluoro-5-isopropoxypent-3-en-2-yl)benzene (3o)

^{1}H NMR (500 MHz, $CDCl_3$)

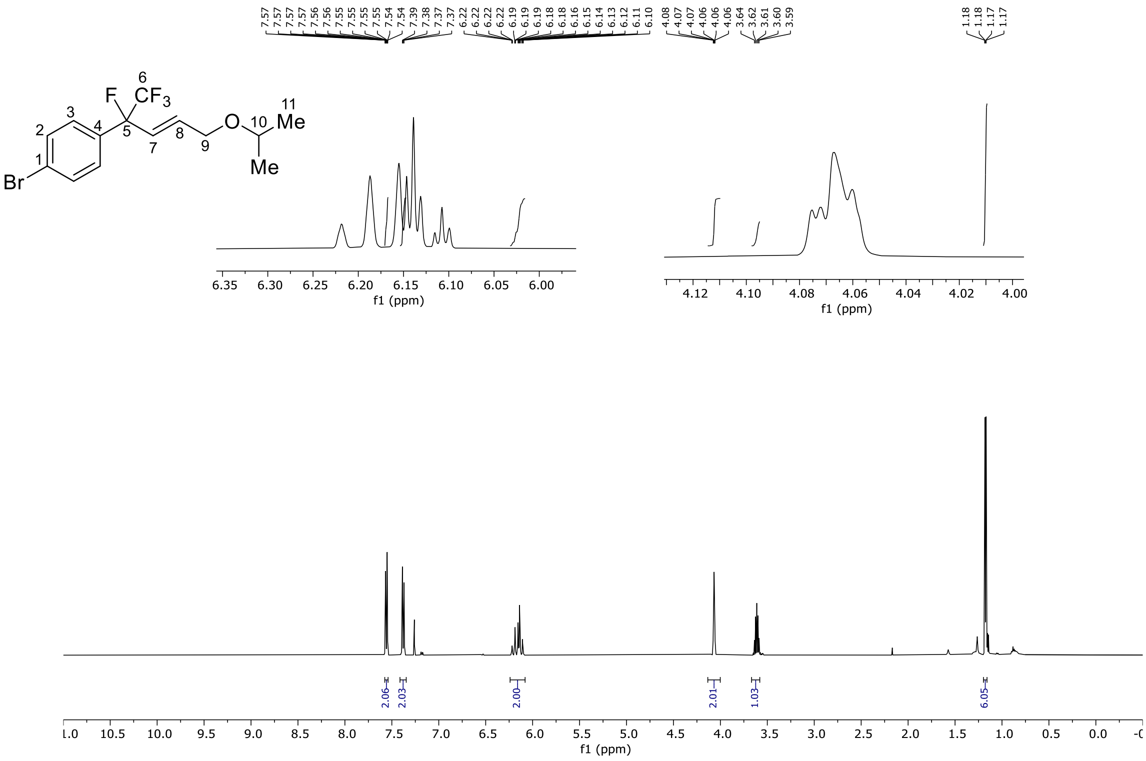

^{13}C NMR (126MHz, $CDCl_3$)

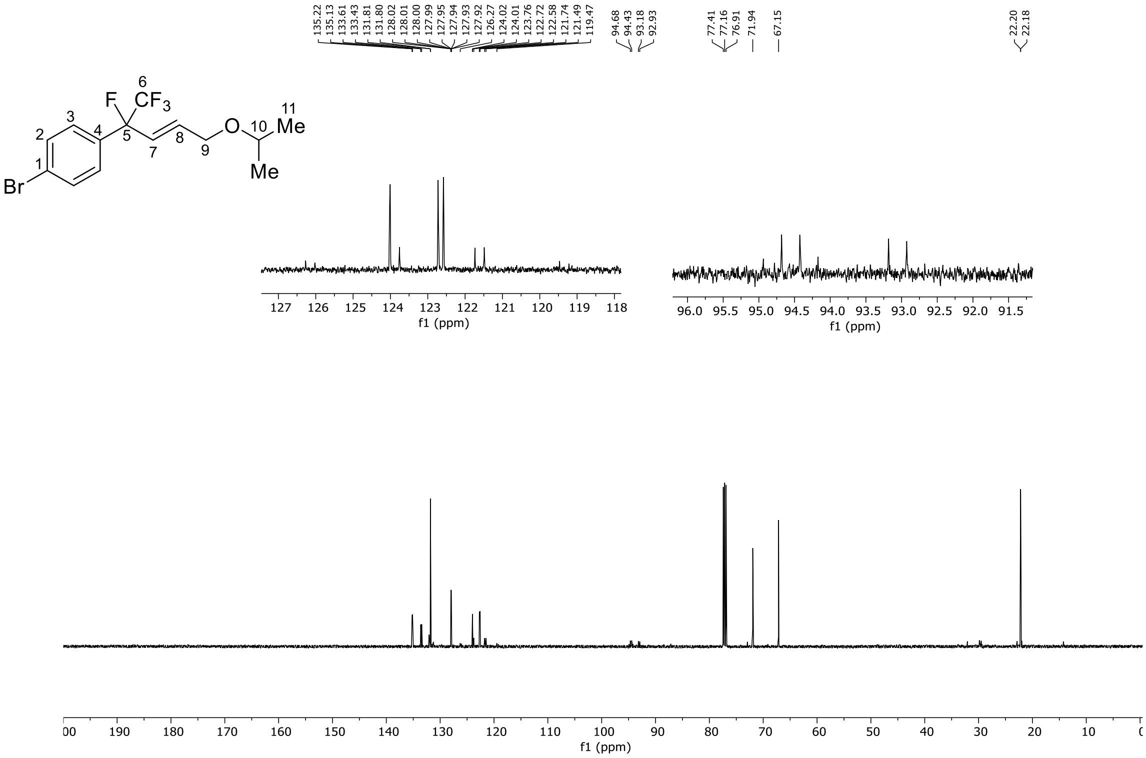

^{19}F NMR (470 MHz, $CDCl_3$)

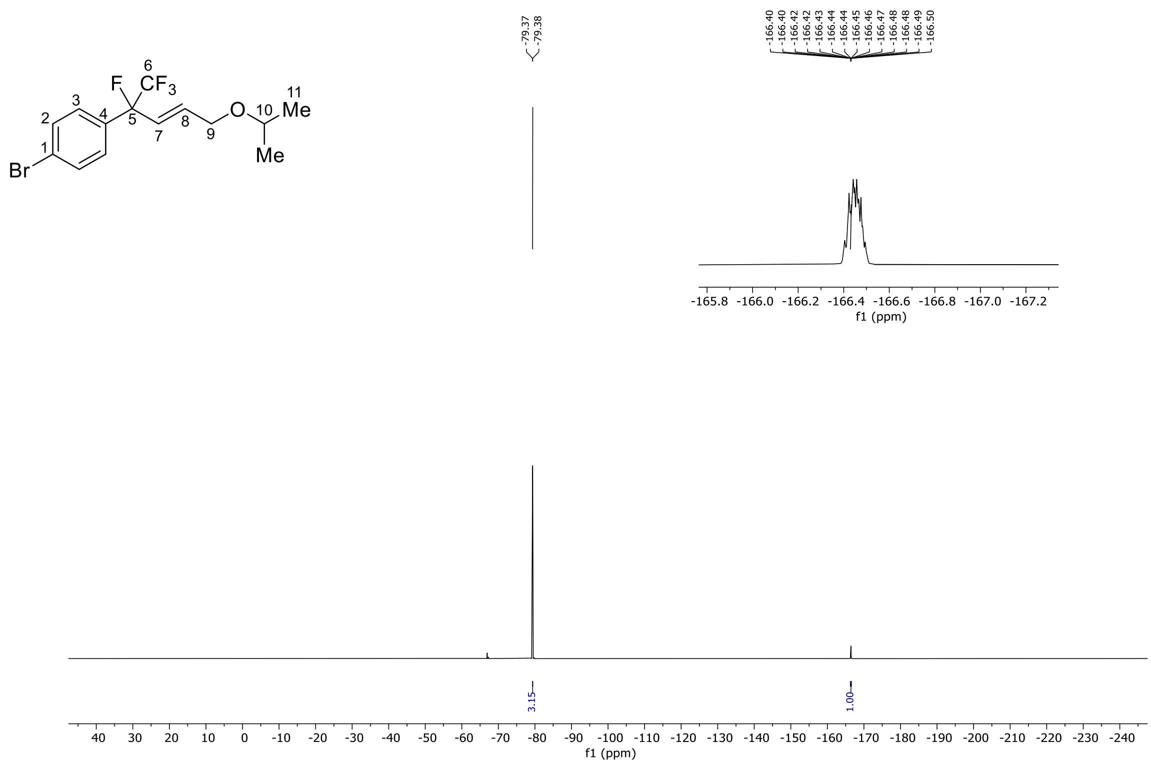

^{19}F{^{1}H} NMR (470 MHz, $CDCl_3$)

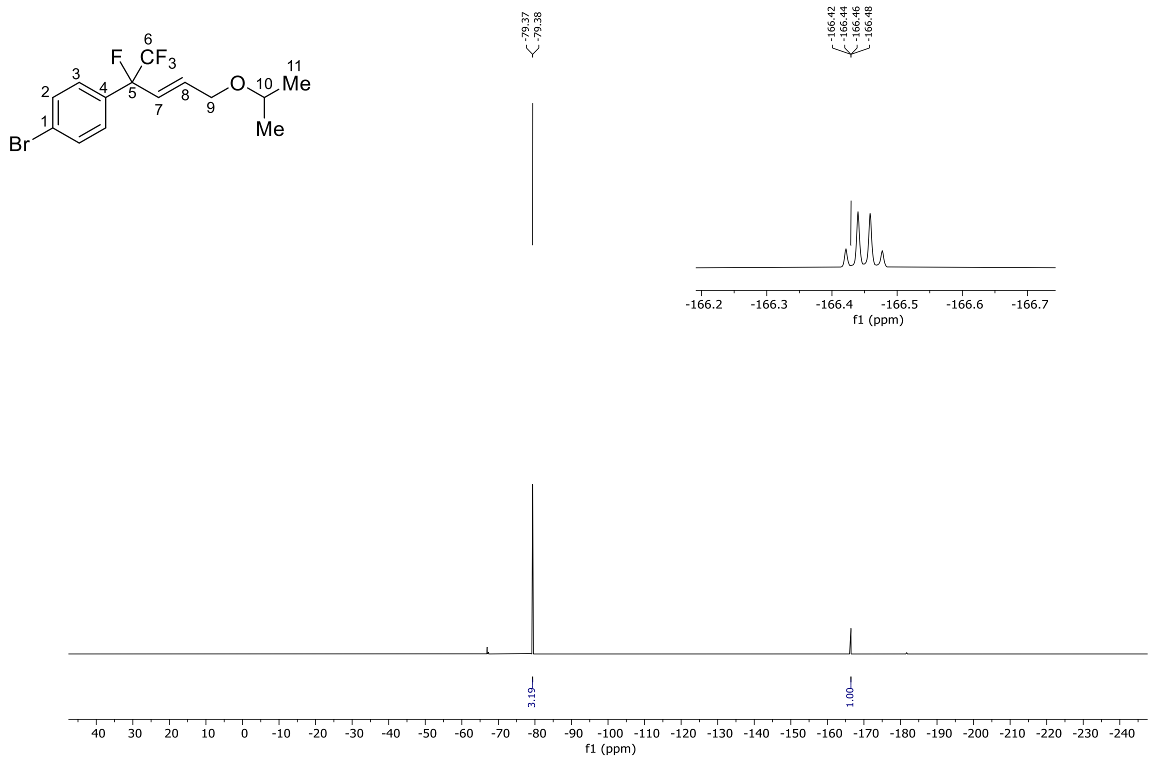

(*E*)-1-Bromo-4-(1,1,1,2-tetrafluoro-5-(2-(2-methoxyethoxy)ethoxy)pent-3-en-2-yl)benzene (3p)

^{1}H NMR (500 MHz, $CDCl_3$)

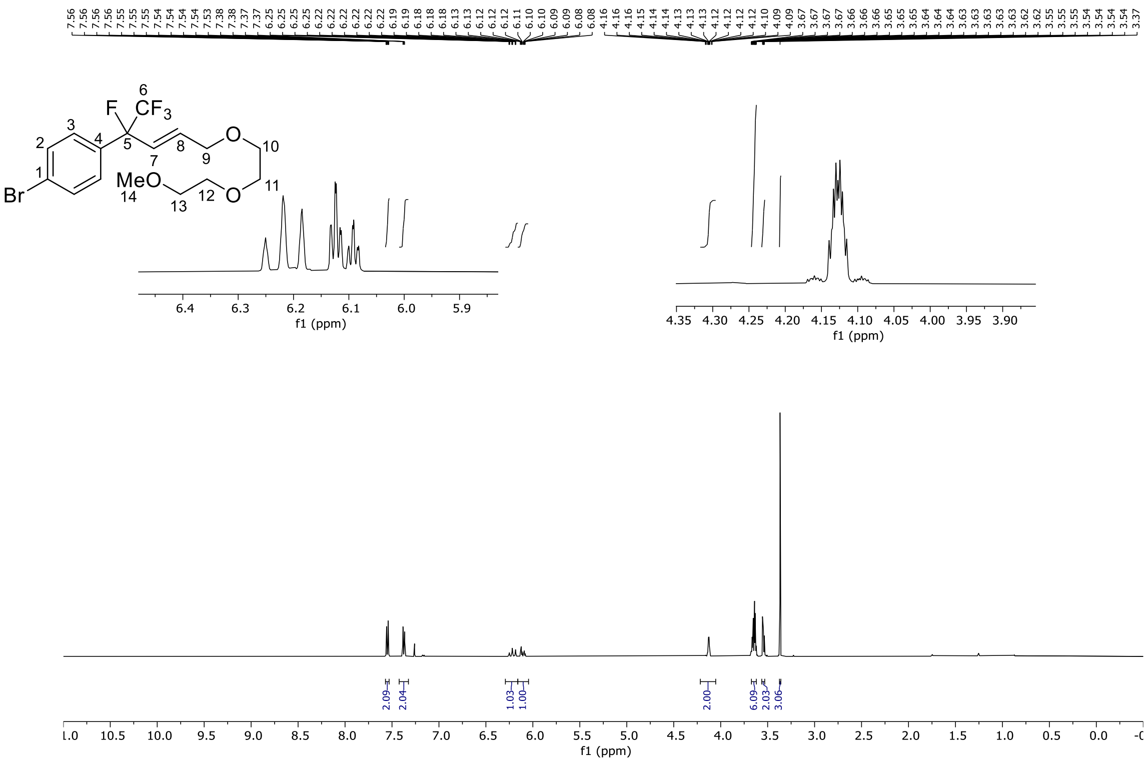

^{13}C NMR (126MHz, $CDCl_3$)

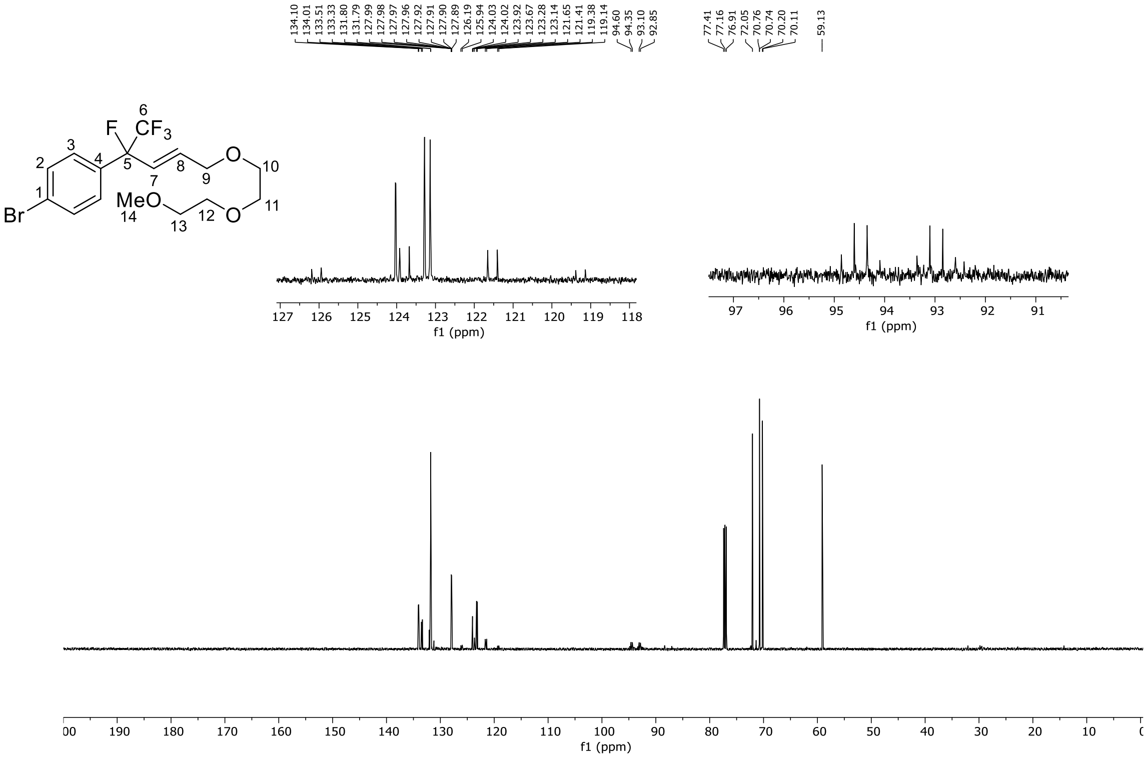

^{19}**F NMR** (470 MHz, $CDCl_3$)

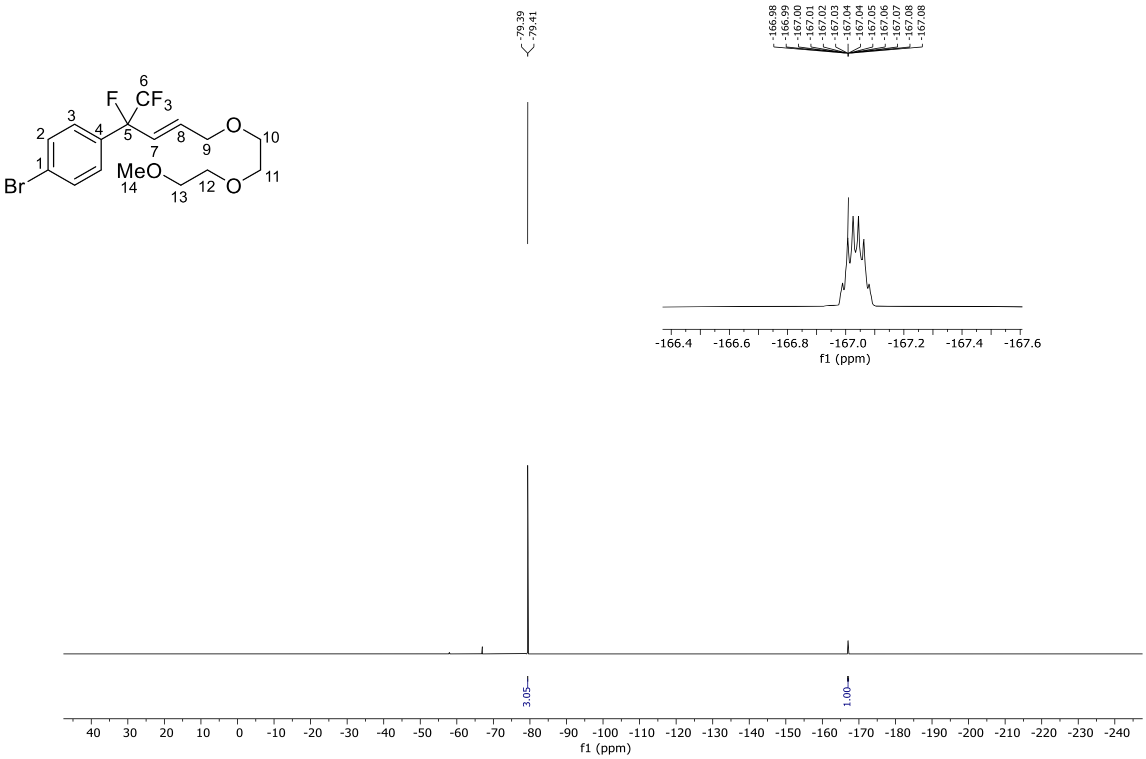

^{19}**F{^{1}H} NMR** (470 MHz, $CDCl_3$)

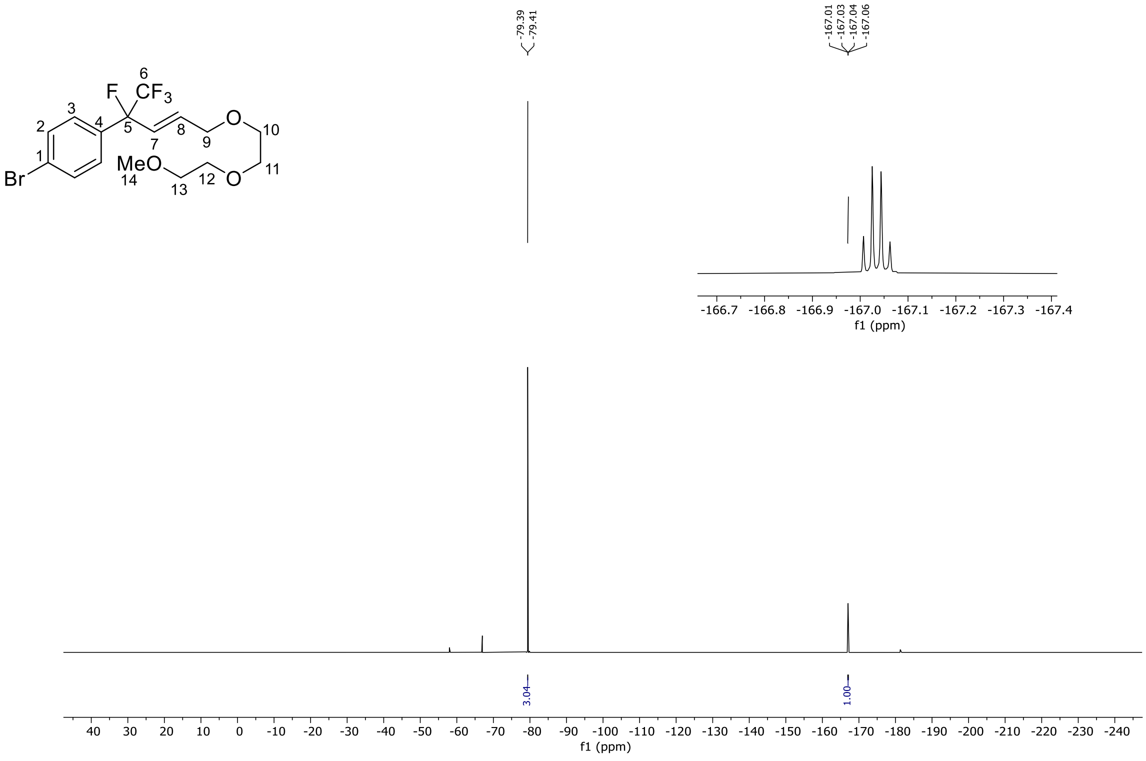

Methyl (2*S*)-2-(((*E*)-4-(4-bromophenyl)-4,5,5,5-tetrafluoropent-2-en-1-yl)oxy)propanoate (3q)

^{1}H NMR (500 MHz, $CDCl_3$)

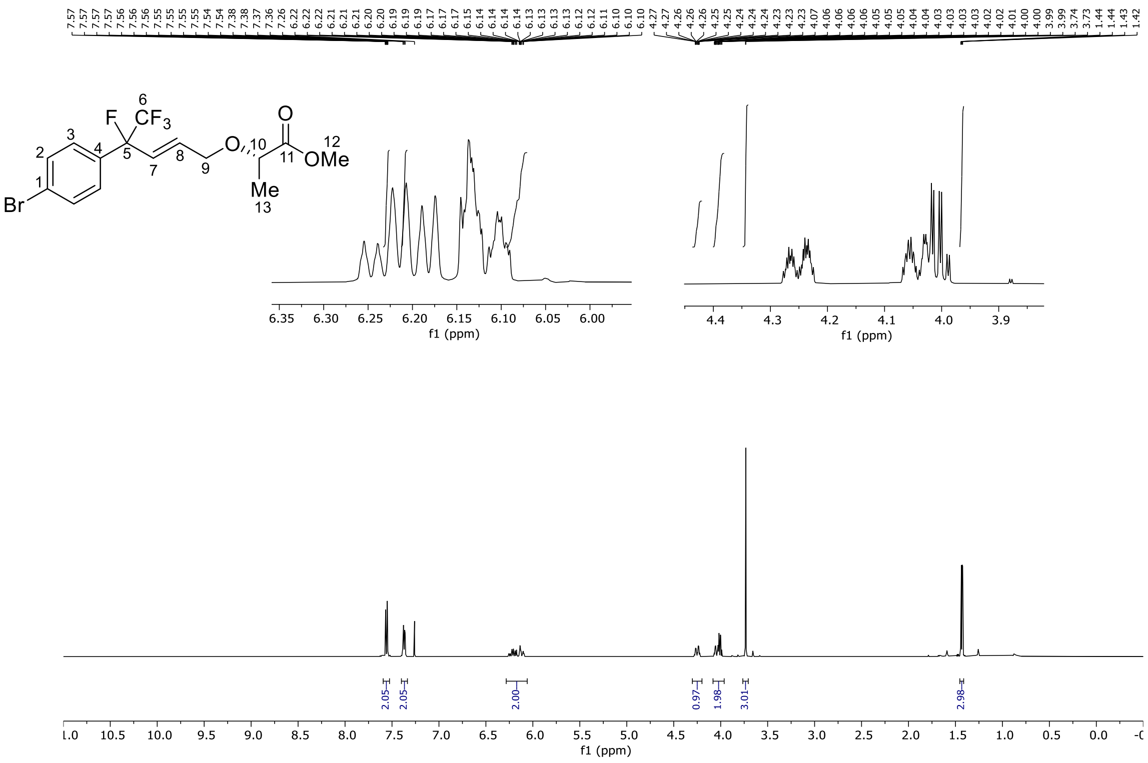

^{13}C NMR (126MHz, $CDCl_3$)

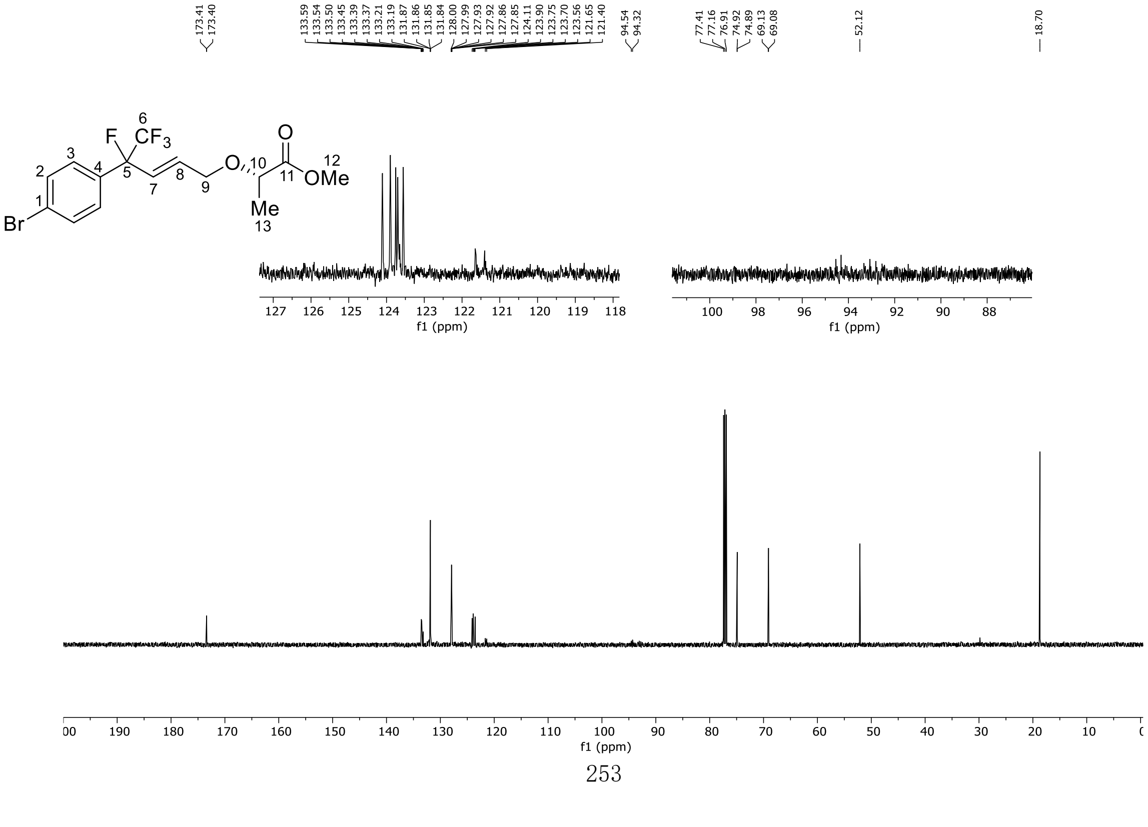

^{19}F NMR (470 MHz, $CDCl_3$)

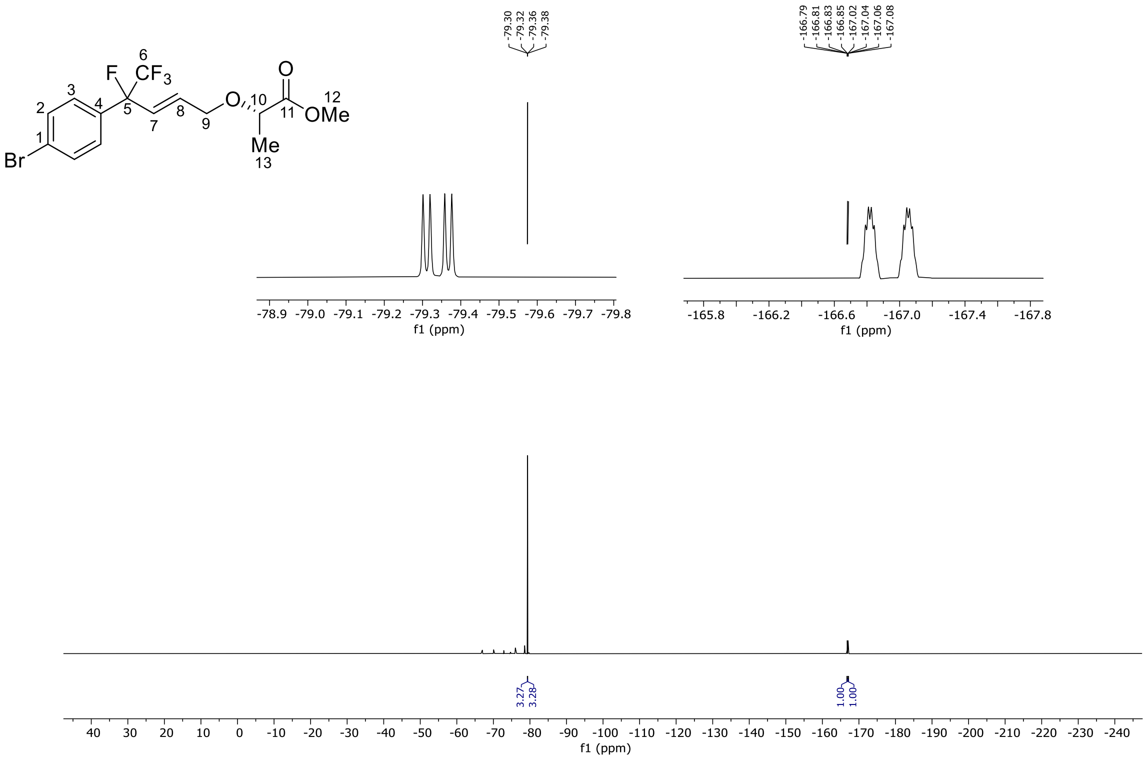

^{19}F{^{1}H} NMR (470 MHz, $CDCl_3$)

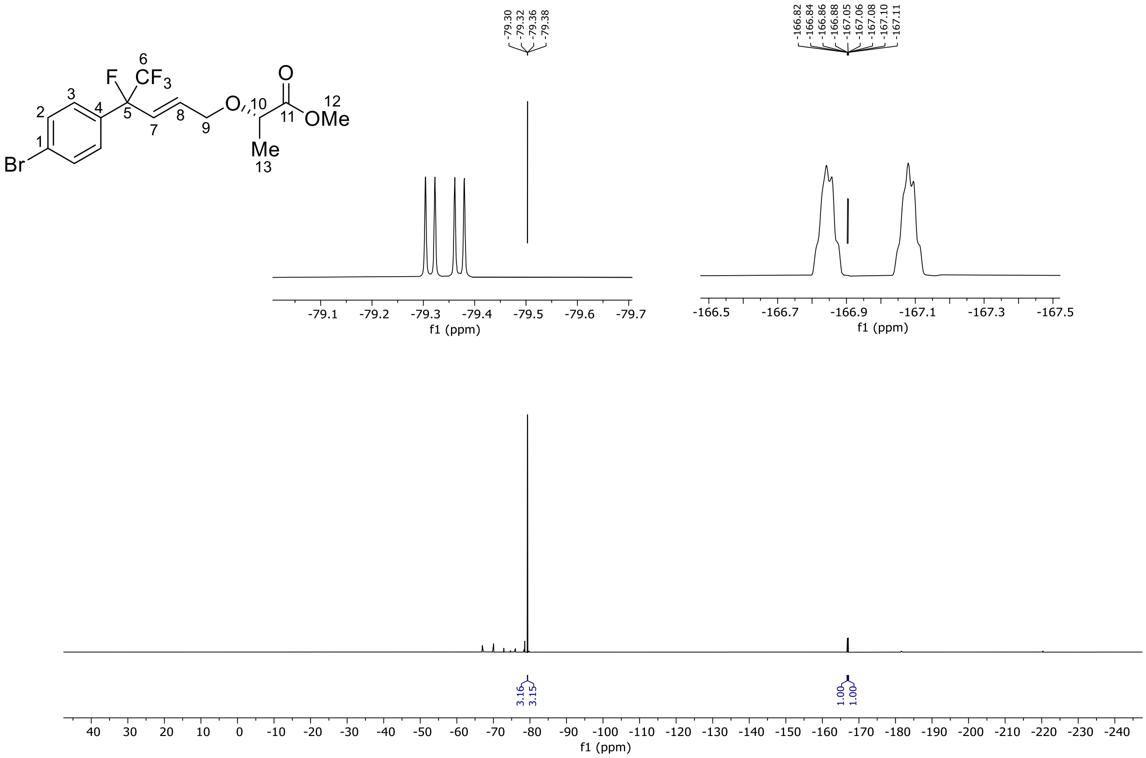

(*E*)-4-(4-Bromophenyl)-4,5,5,5-tetrafluoropent-2-en-1-ol (3r)

^{1}H NMR (500 MHz, $CDCl_3$)

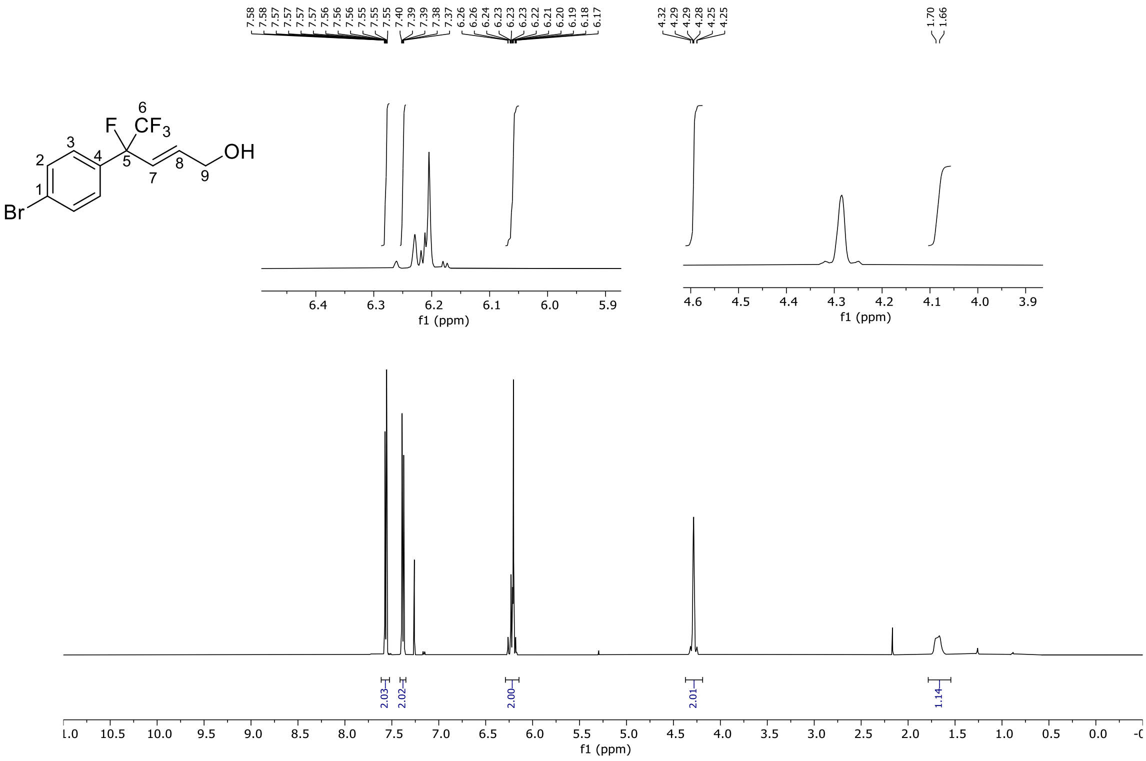

^{13}C NMR (126 MHz, $CDCl_3$)

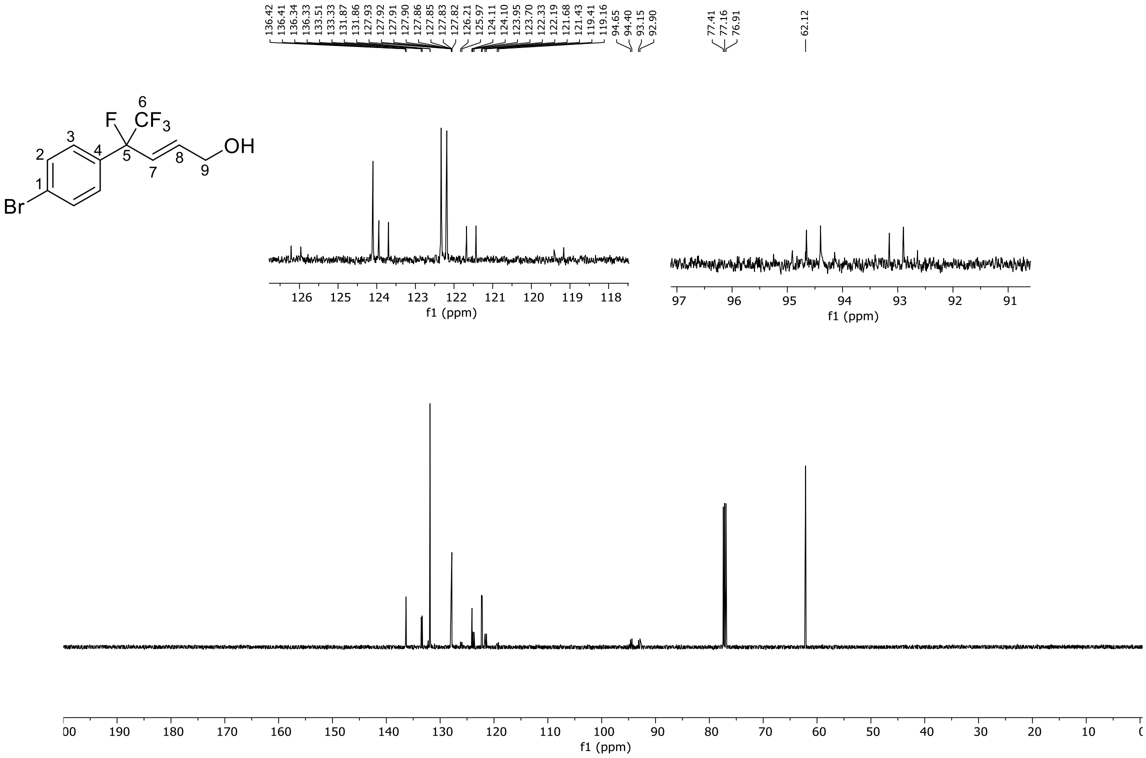

^{19}**F NMR** (470 MHz, $CDCl_3$)

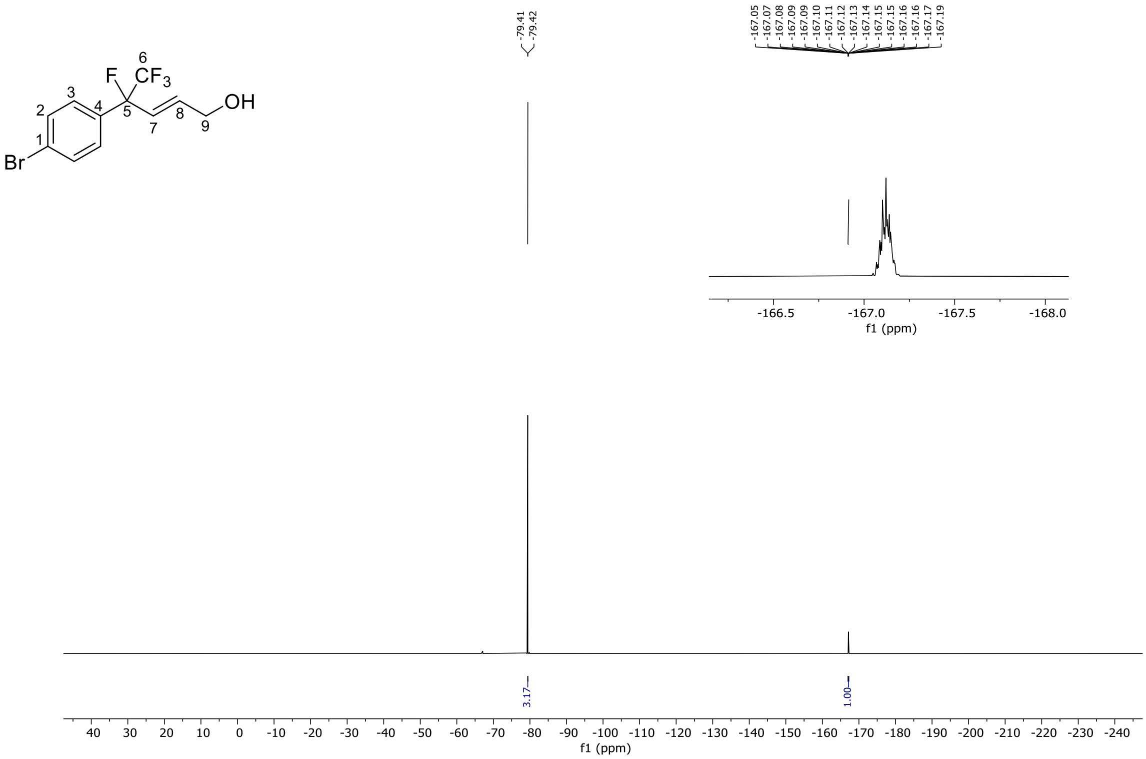

^{19}**F{^{1}H}NMR** (470 MHz, $CDCl_3$)

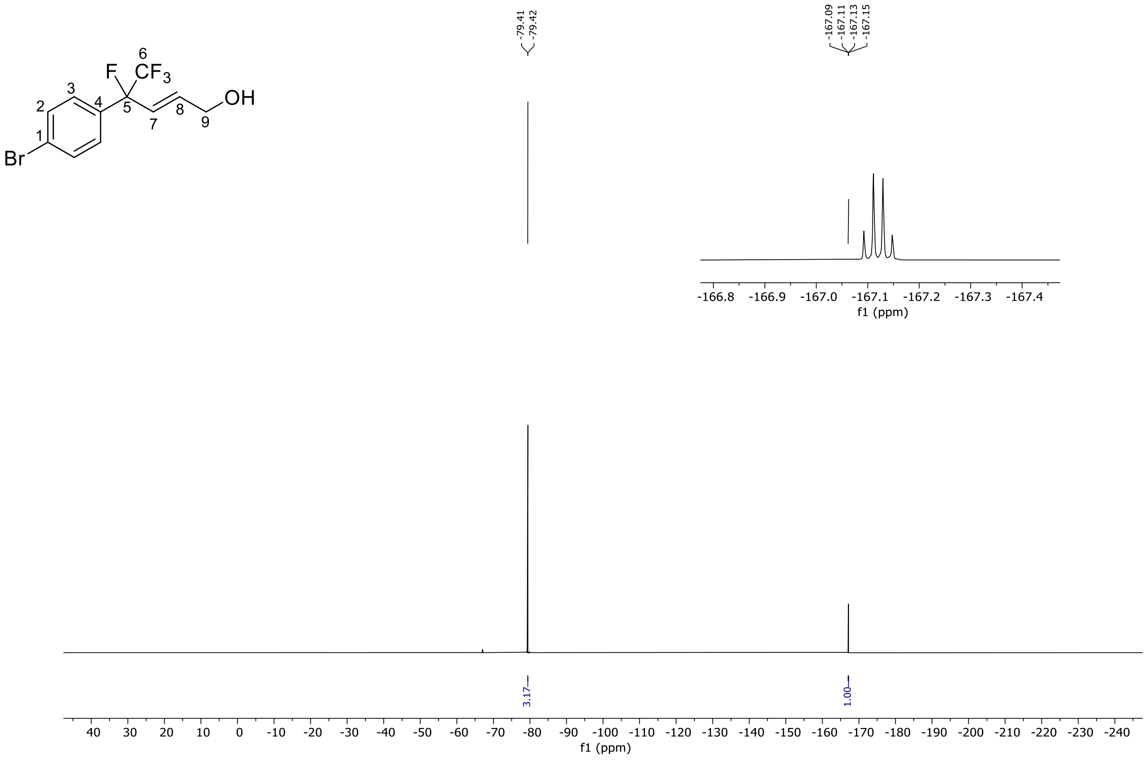

(*E*)-1-Bromo-4-(5-bromo-1,1,1,2,5-pentafluoropent-3-en-2-yl)benzene (4a)

^{1}H NMR (500 MHz, $CDCl_3$)

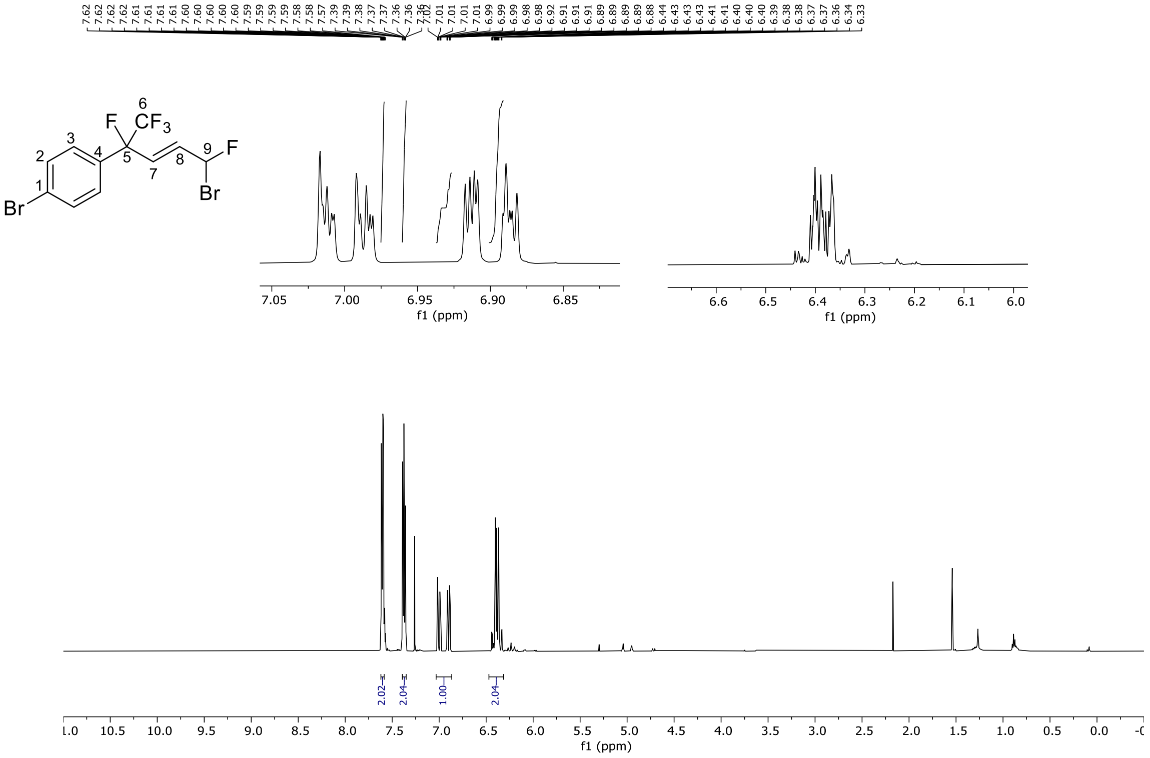

^{13}C NMR (126 MHz, $CDCl_3$)

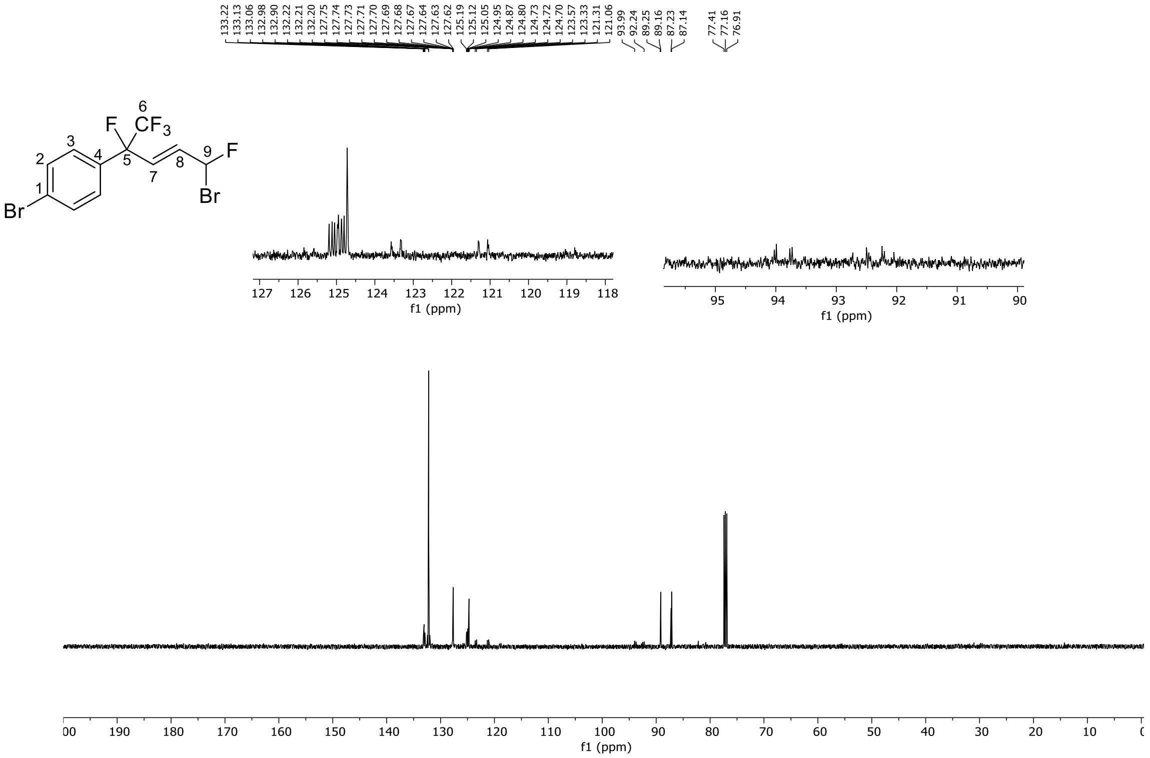

^{19}F NMR (470 MHz, $CDCl_3$)

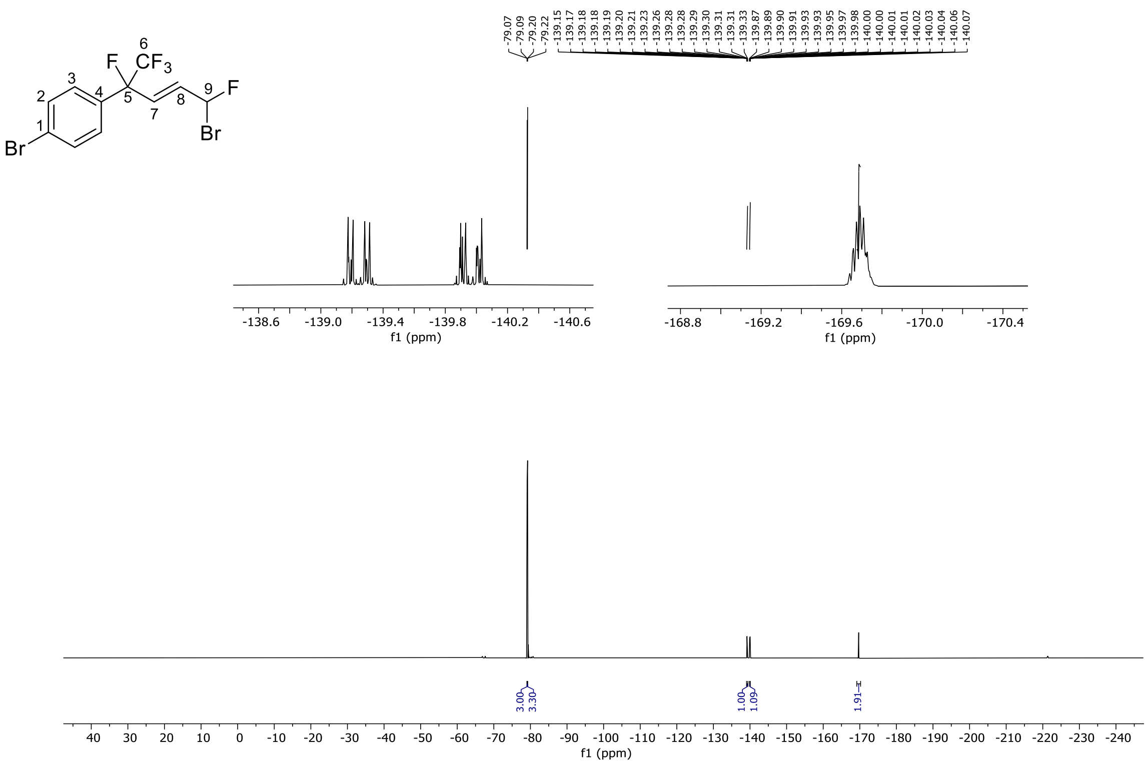

^{19}F{^{1}H} NMR (470 MHz, $CDCl_3$)

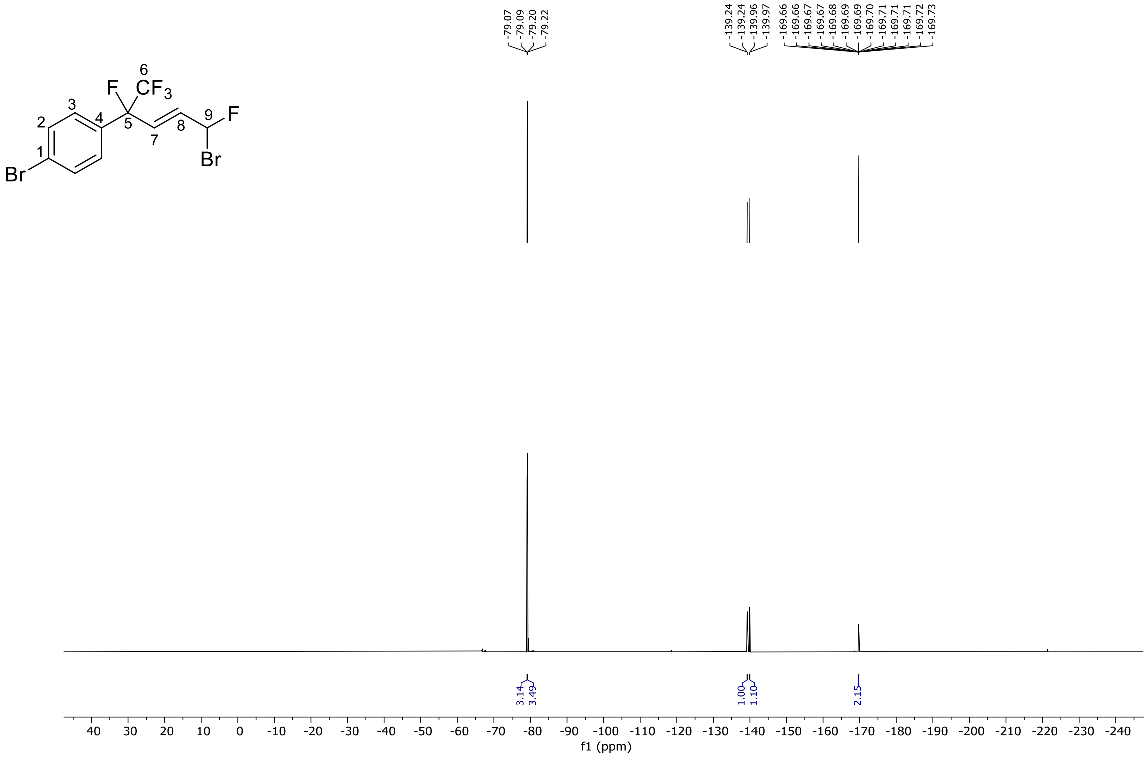

2-(4-Bromophenyl)-2,3,3,3-tetrafluoropropan-1-ol (4b)

^{1}H NMR (500 MHz, $CDCl_3$)

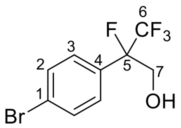

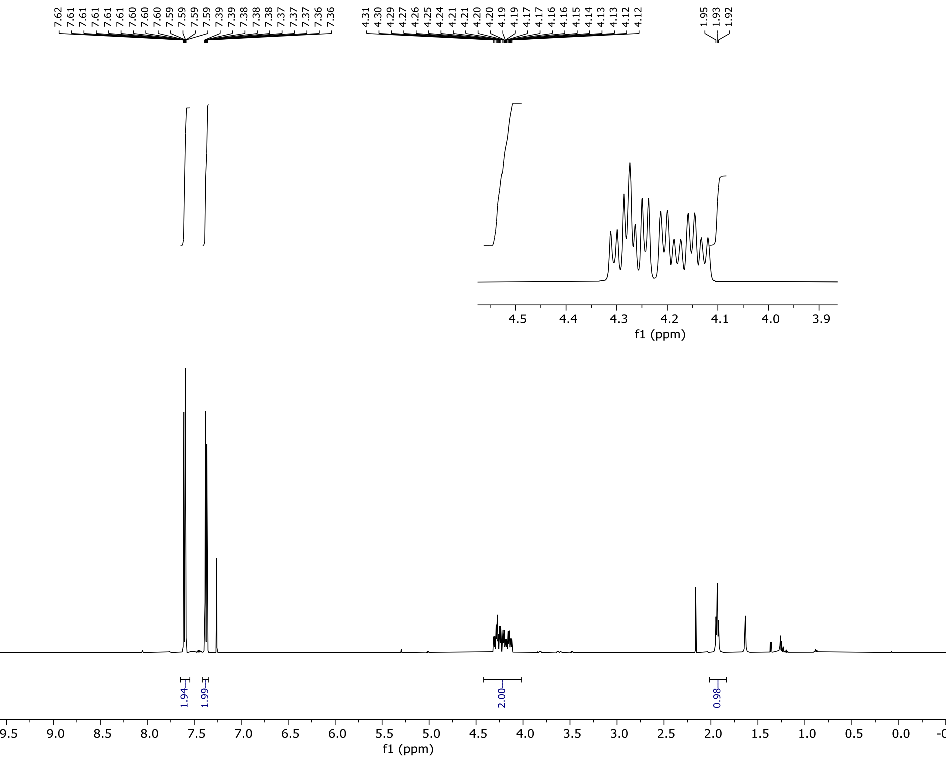

^{13}C NMR (126 MHz, $CDCl_3$)

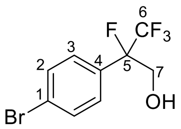

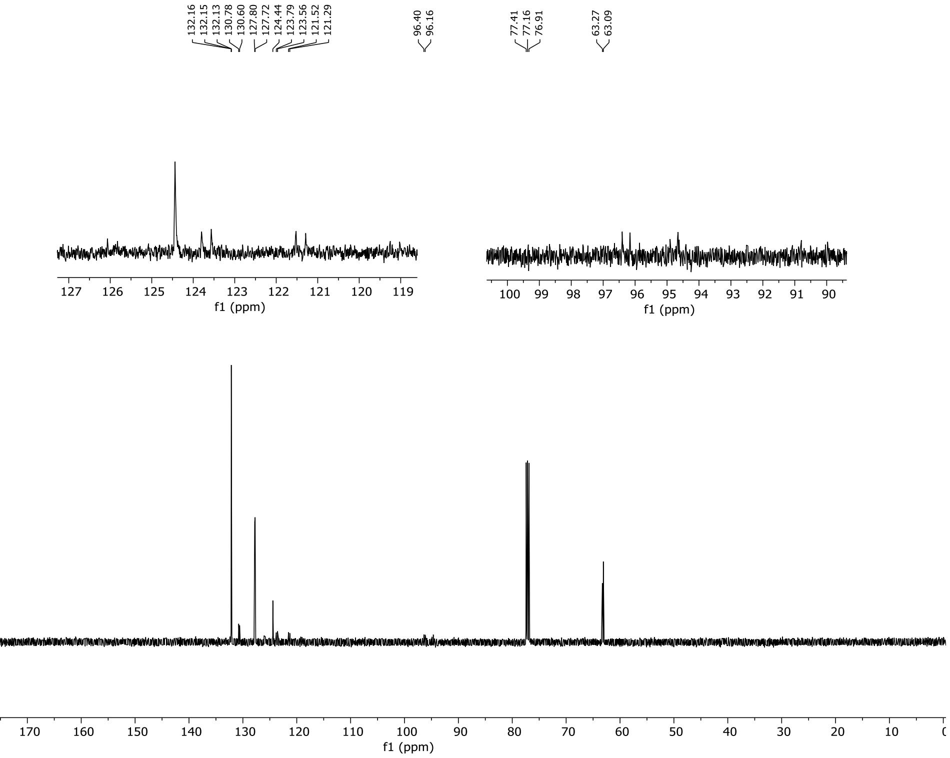

^{19}F NMR (470 MHz, CDCl$_3$)

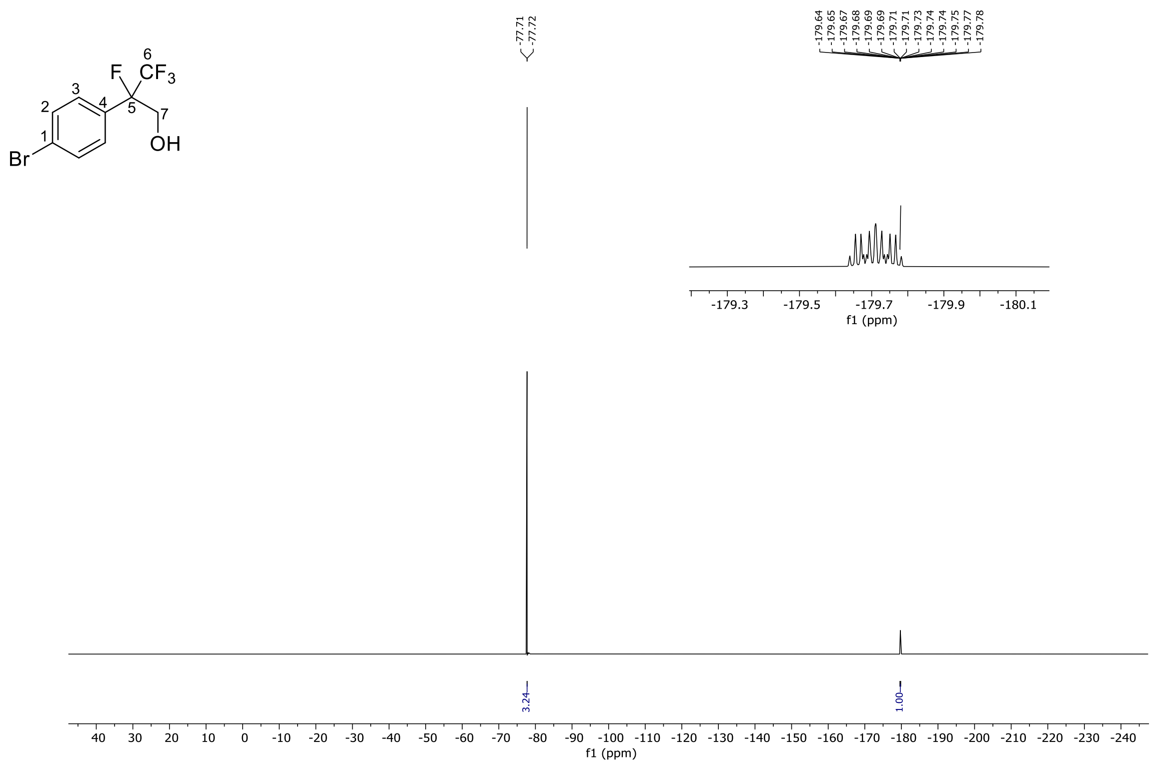

^{19}F{^{1}H} NMR (470 MHz, CDCl$_3$)

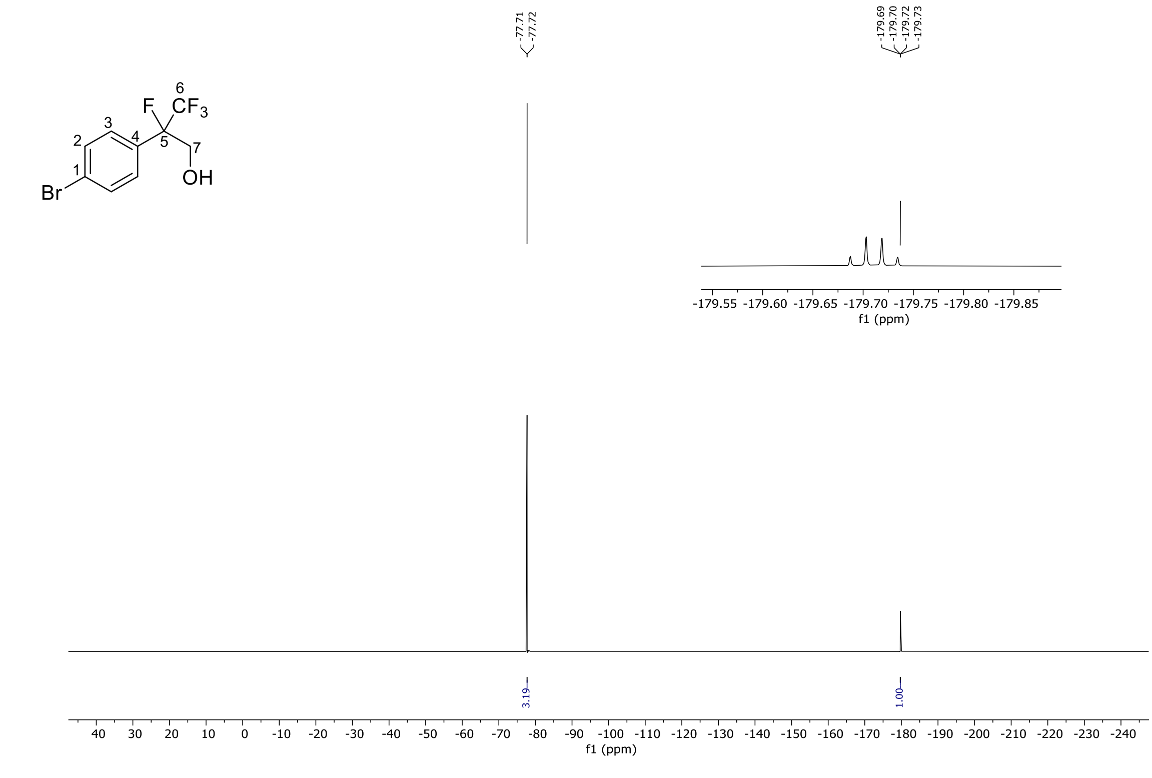

(2*S*,3*S*)-2-((*R*)-1-(4-Bromophenyl)-1,2,2,2-tetrafluoroethyl)-3-(fluoromethyl)oxirane (4c)

^{1}H NMR (500 MHz, $CDCl_3$)

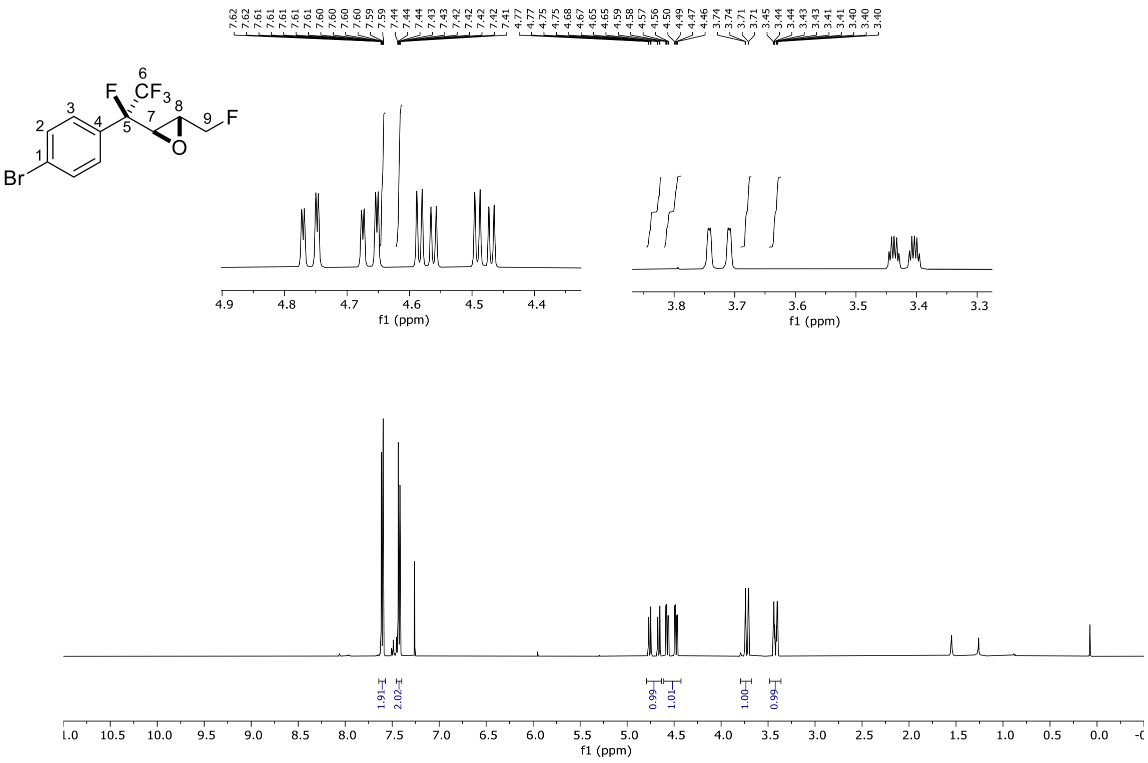

^{13}C NMR (126 MHz, $CDCl_3$)

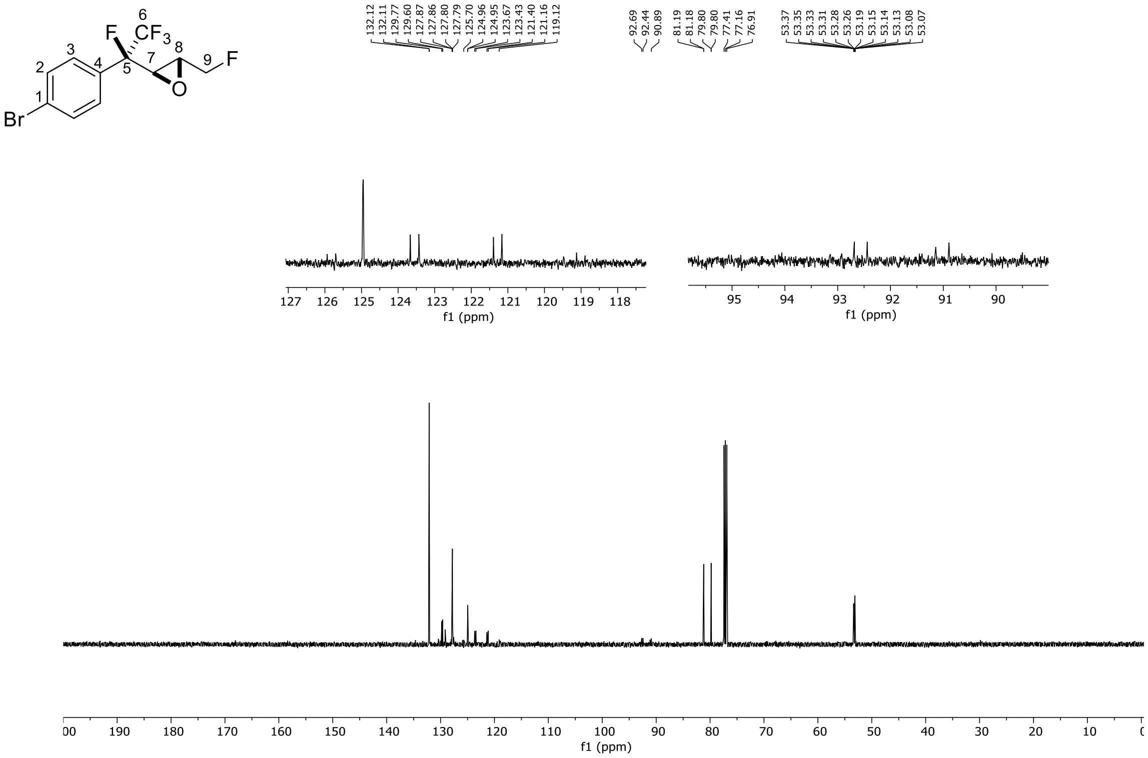

^{19}F NMR (470 MHz, CDCl$_3$)

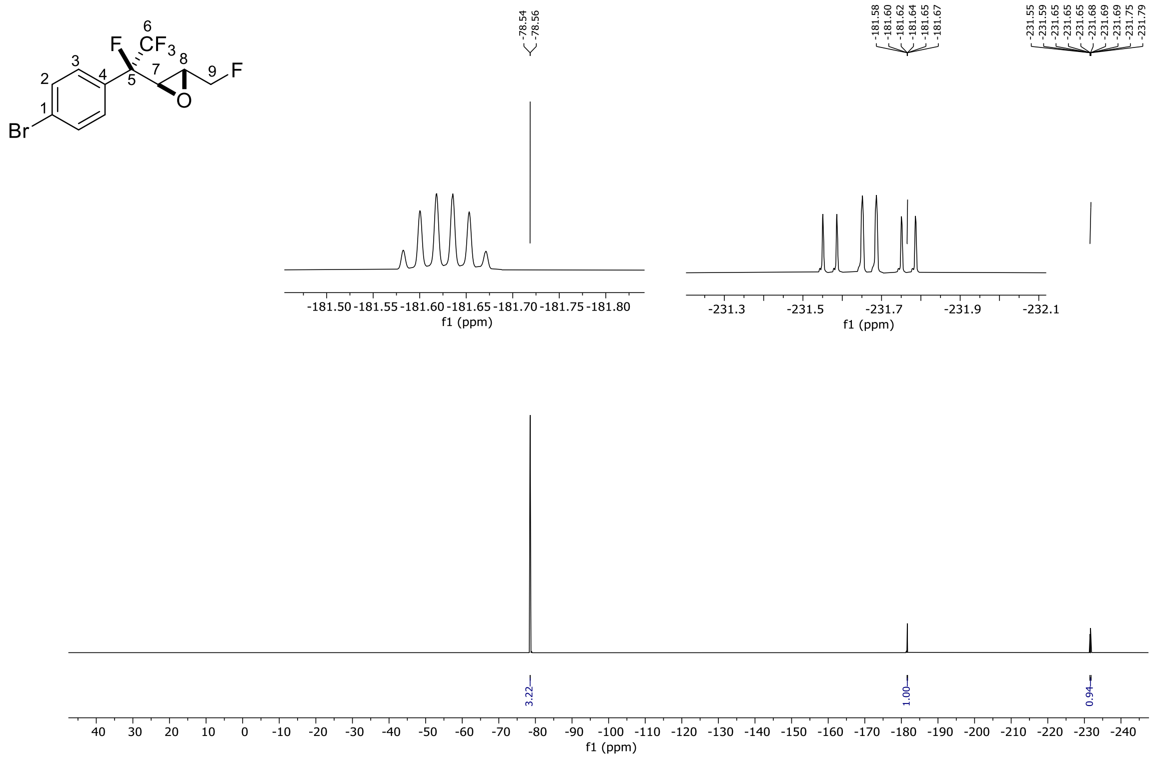

^{19}F{^{1}H} NMR (470 MHz, CDCl$_3$)

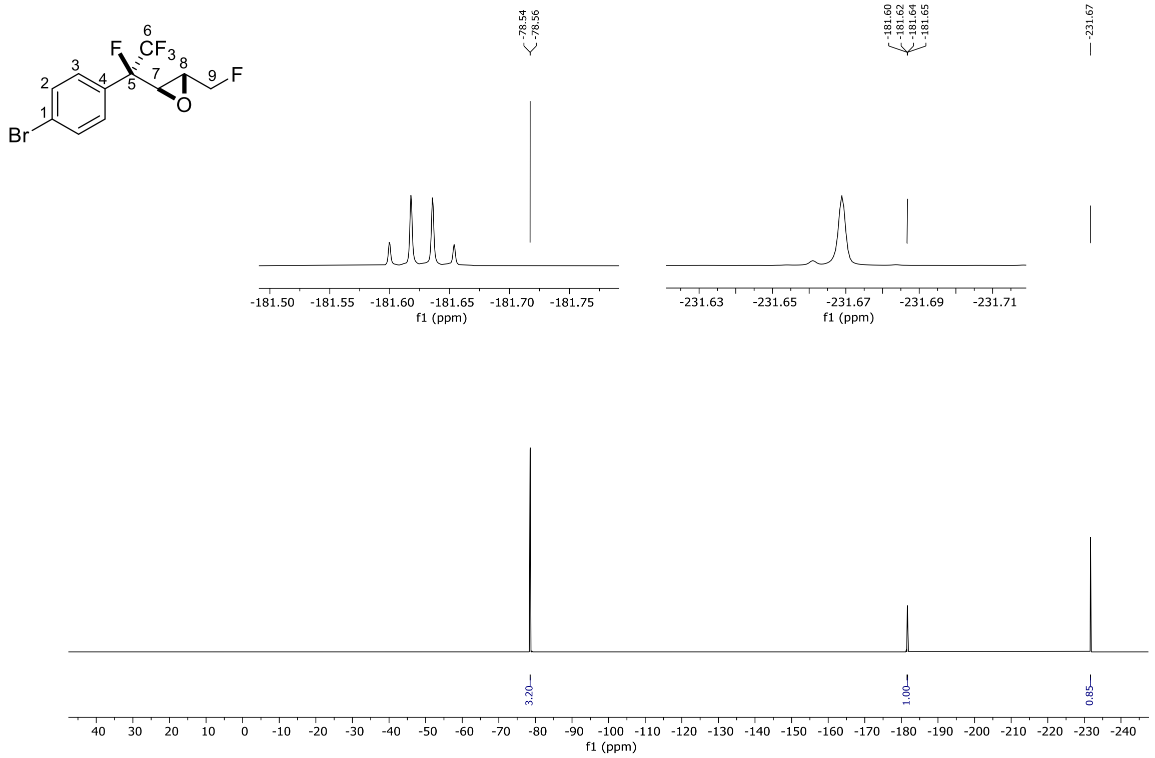

(2*R*,3*R*)-2-((*R*)-1-(4-Bromophenyl)-1,2,2,2-tetrafluoroethyl)-3-(fluoromethyl)oxirane (4c)

^{1}H NMR (500 MHz, $CDCl_3$)

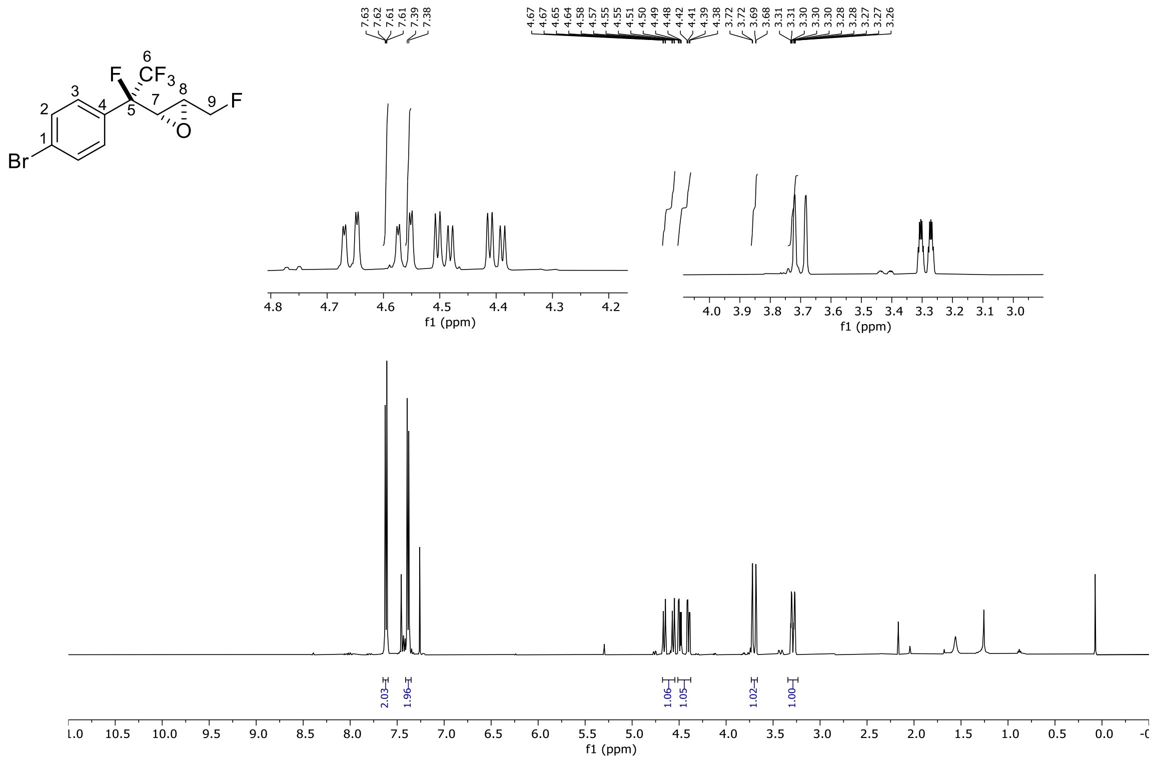

^{13}C NMR (126 MHz, $CDCl_3$)

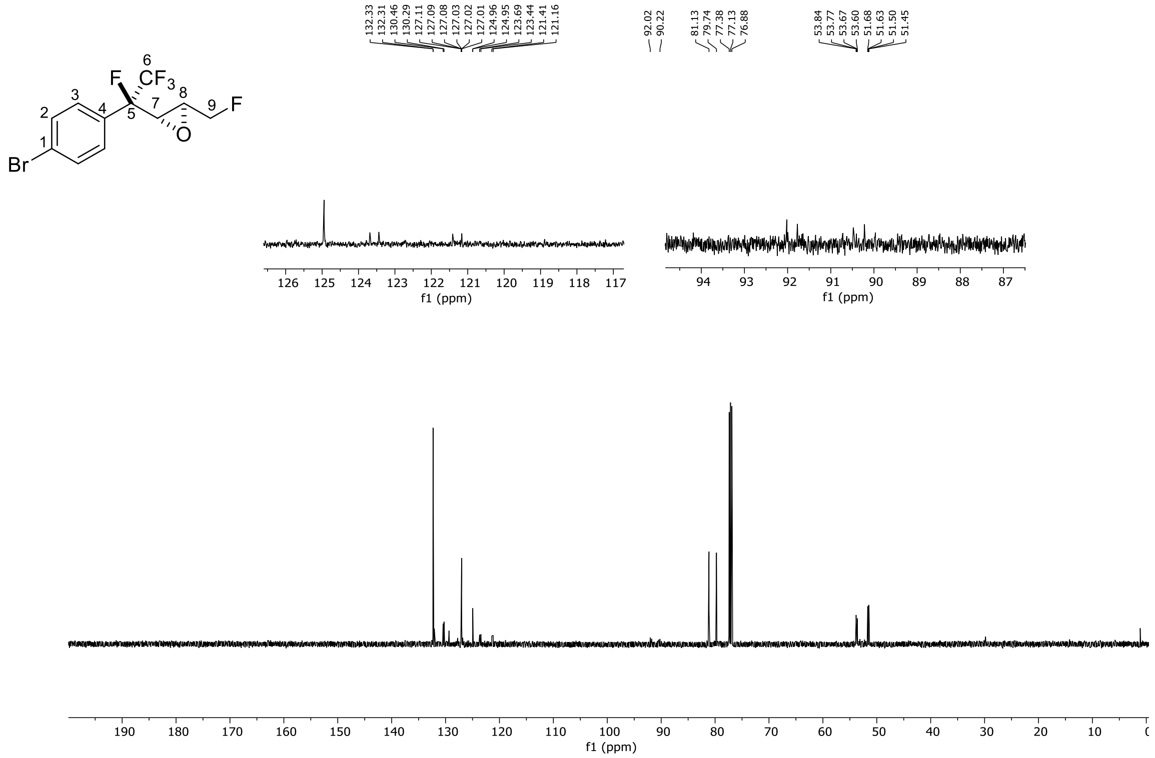

^{19}F NMR (376 MHz, $CDCl_3$)

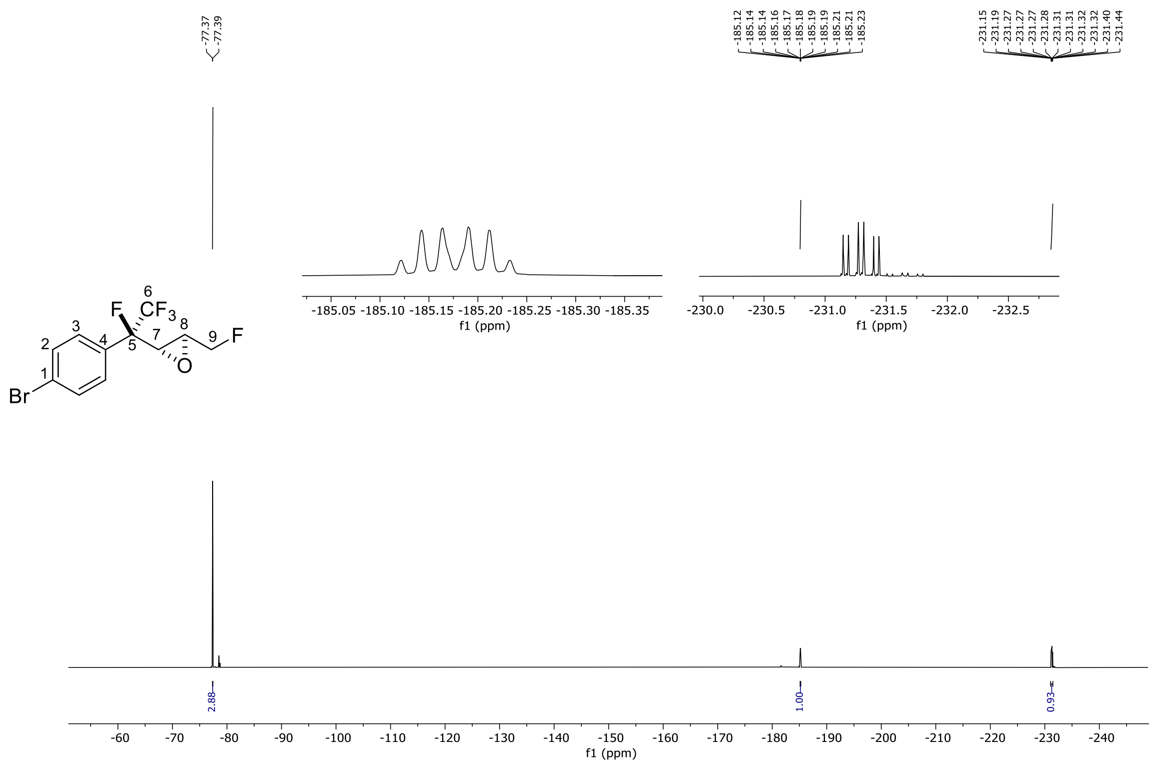

^{19}F{^{1}H} NMR (376 MHz, $CDCl_3$)

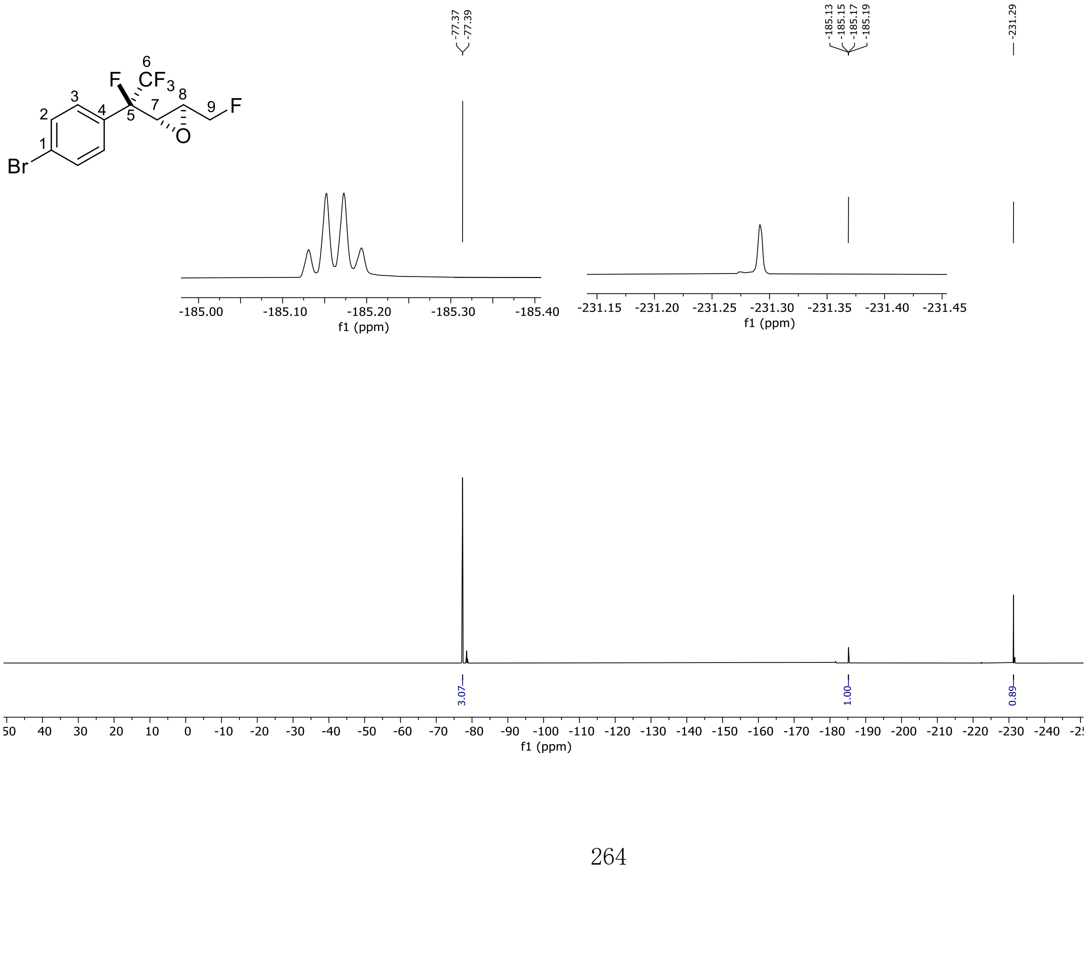

(*E*)-1-Bromo-4-(5-bromo-1,1,1,2-tetrafluoropent-3-en-2-yl)benzene (4d)

^{1}H NMR (500 MHz, $CDCl_3$)

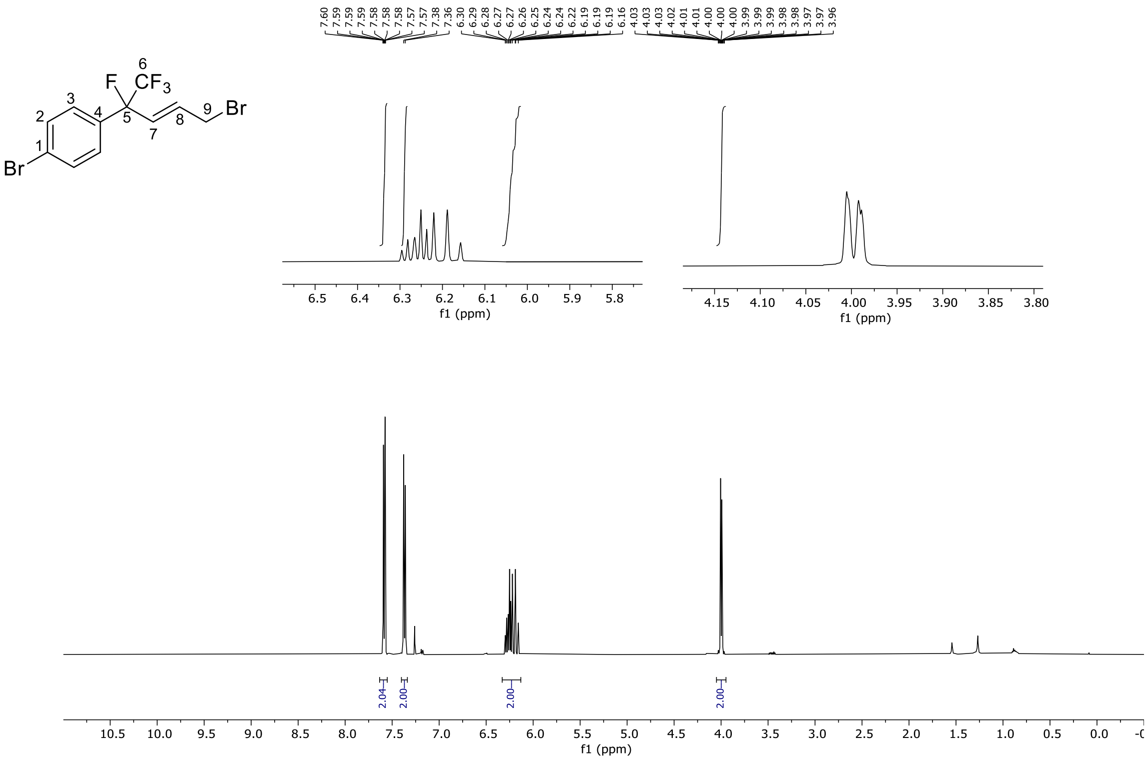

^{13}C NMR (126 MHz, $CDCl_3$)

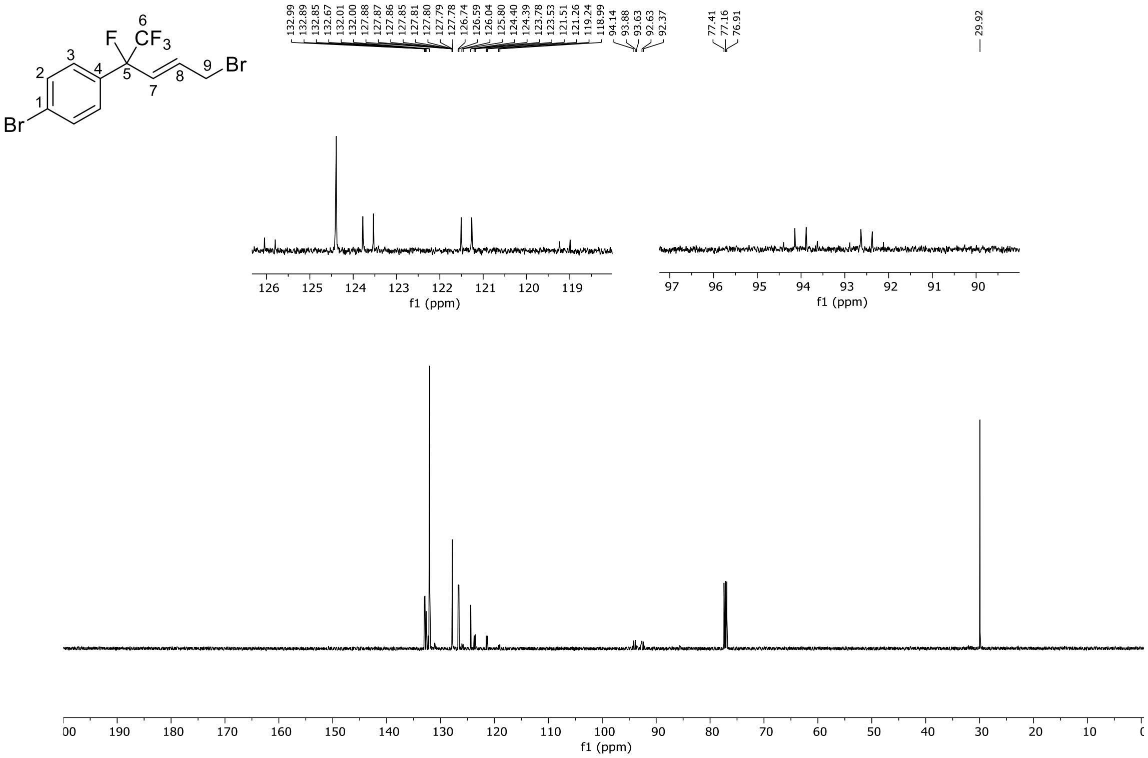

^{19}F NMR (470 MHz, $CDCl_3$)

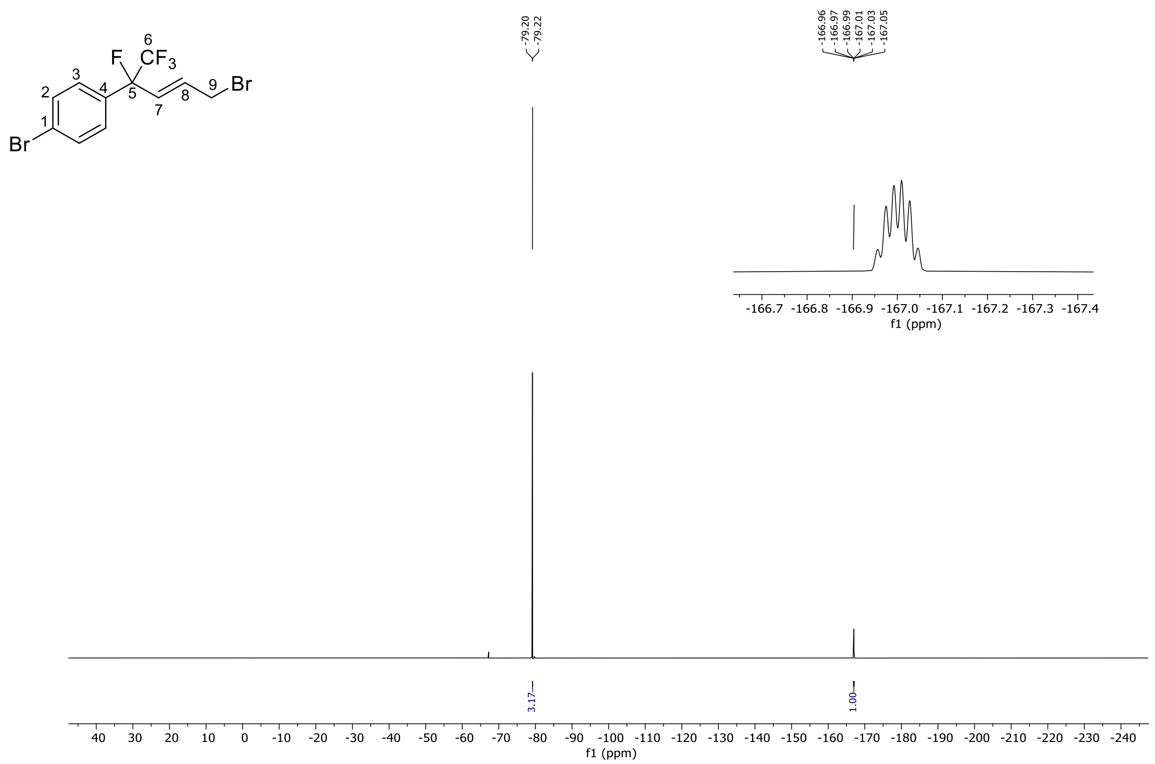

^{19}F{^{1}H} NMR (470 MHz, $CDCl_3$)

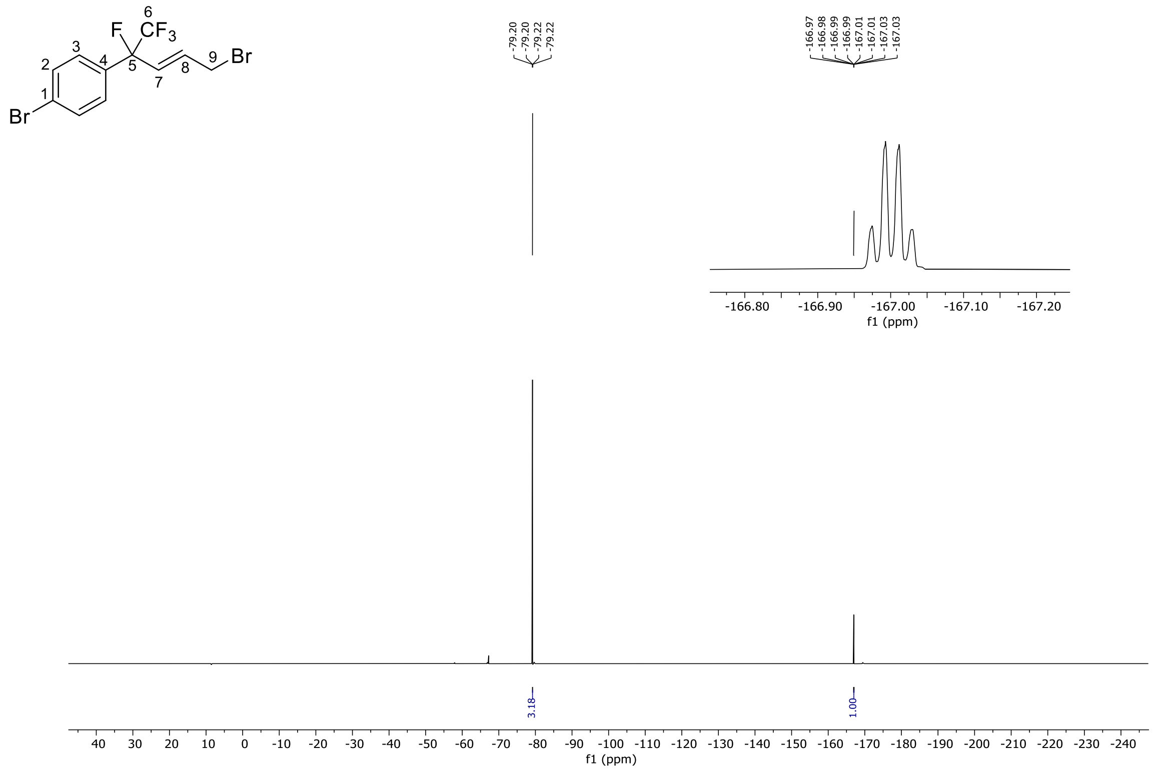

(*E*)-4-(4-Bromophenyl)-4,5,5,5-tetrafluoropent-2-enoic acid (4e)

^{1}H NMR (500 MHz, $CDCl_3$)

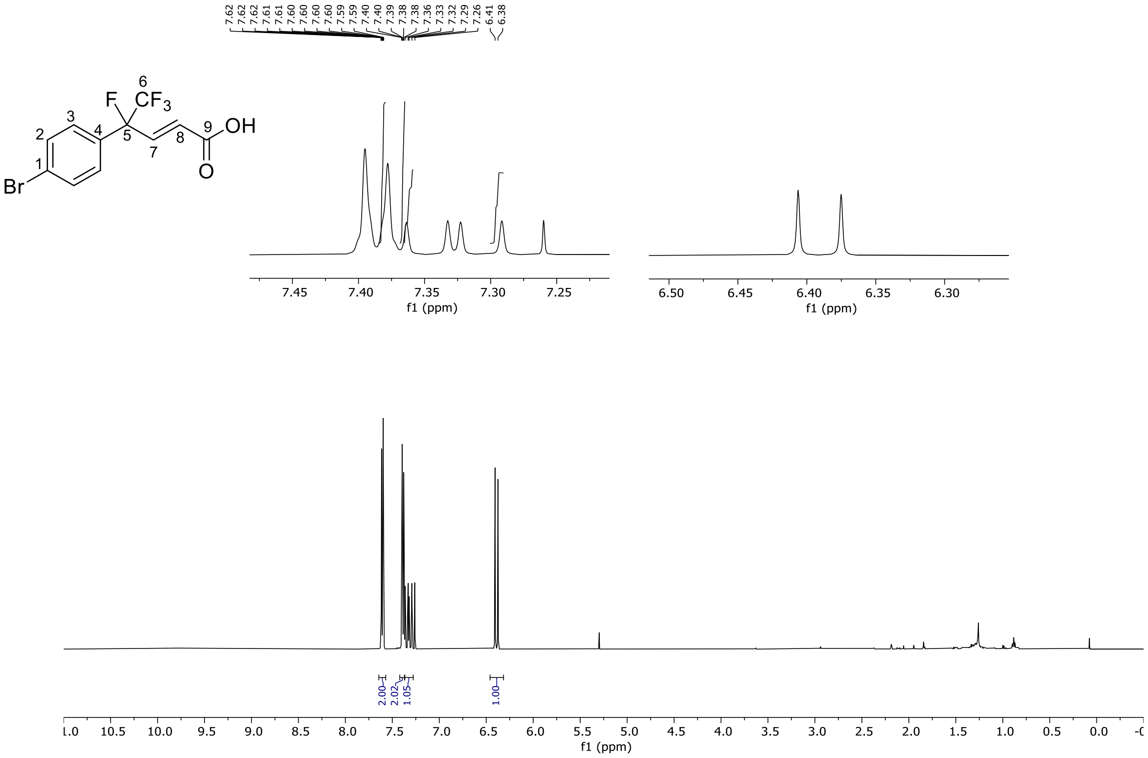

^{13}C NMR (126 MHz, $CDCl_3$)

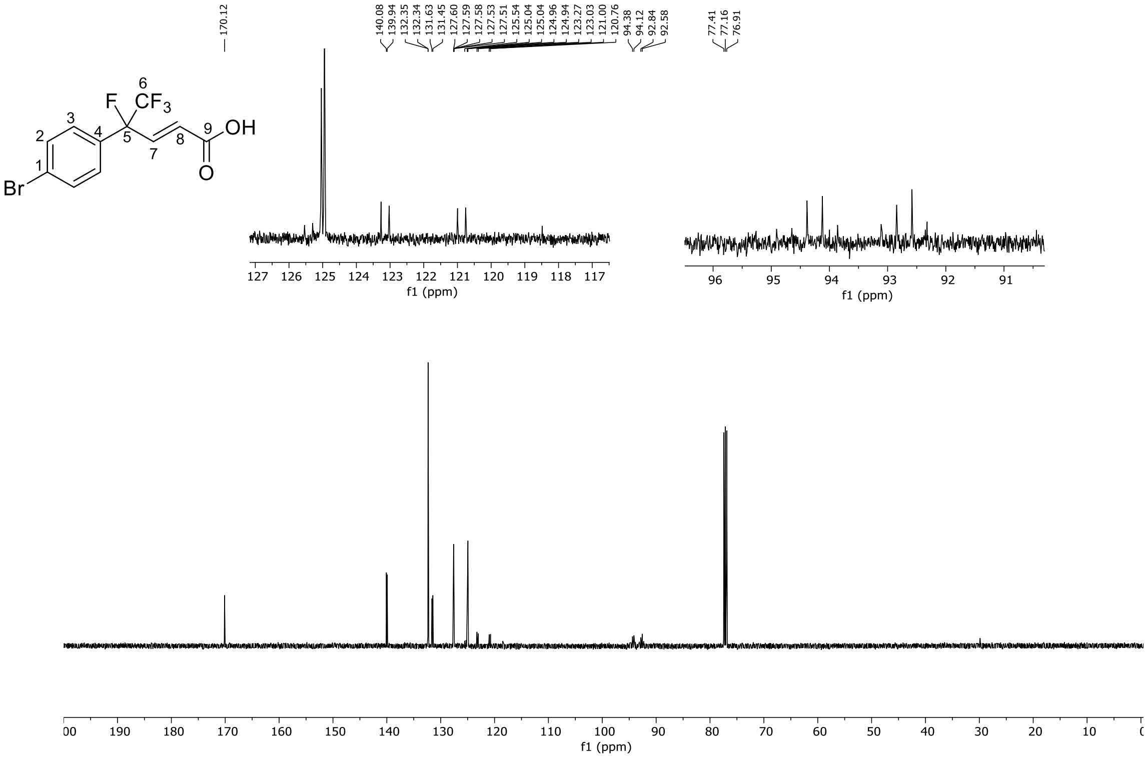

^{19}F NMR (470 MHz, $CDCl_3$)

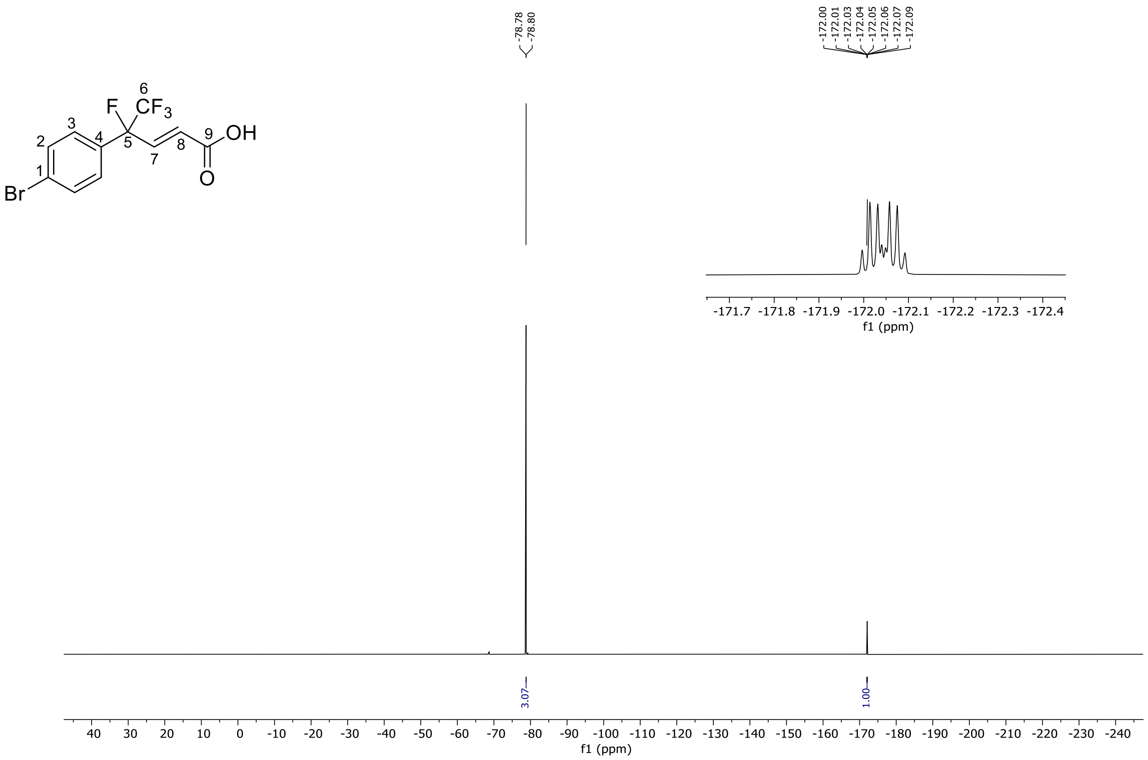

^{19}F{^{1}H} NMR (470 MHz, $CDCl_3$)

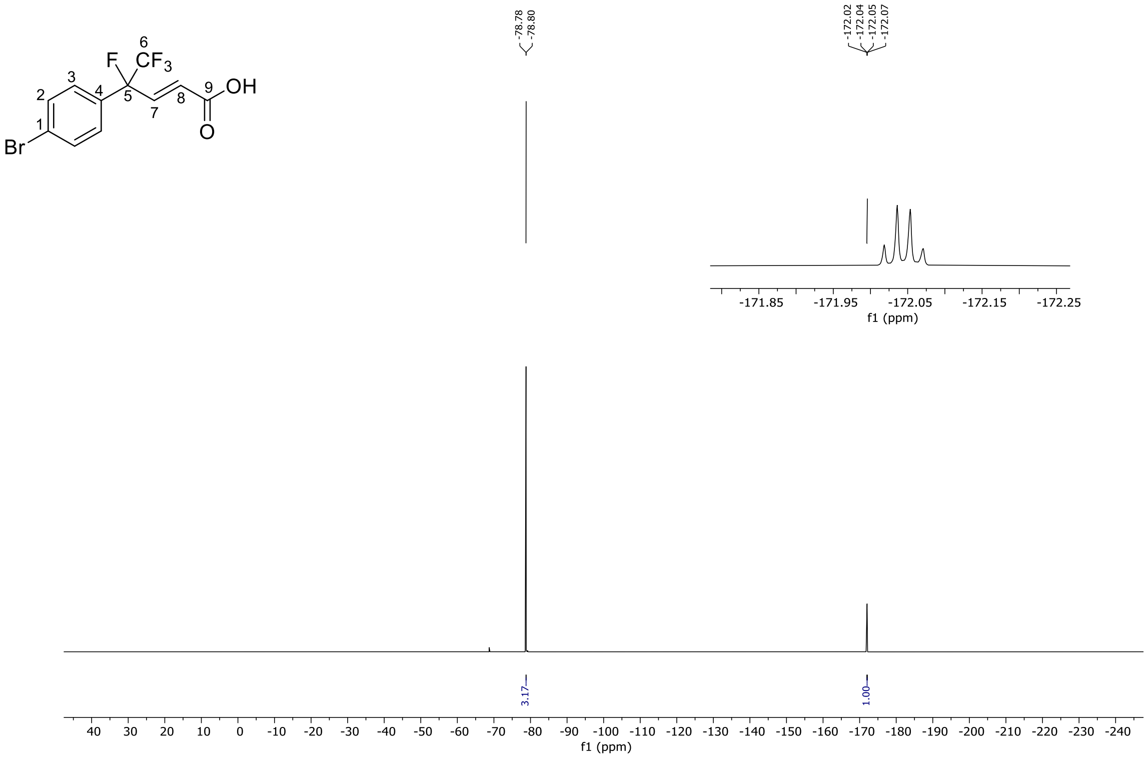

8. References

[1] J. Häfliger, K. Livingstone, C. G. Daniliuc, R. Gilmour, *Chem. Sci.* **2021**, *12*, 6148-6152.

[2] H. Cheng, Y. Pei, F. Leng, J. Li, A. Liang, D. Zou, Y. Wu, Y. Wu, *Tetrahedron Lett.* **2013**, *54*, 4483-4486.

[3] B. M. Trost, L. Debien, *J. Am. Chem. Soc.* **2015**, *137*, 11606-11609.

[4] H. Chen, D. Anand, L. Zhou, *Asian Journal of Organic Chemistry* **2019**, *8*, 661-664.

[5] L. Becerra-Figueroa, E. Brun, M. Mathieson, L. J. Farrugia, C. Wilson, J. Prunet, D. Gamba-Sánchez, *Org. Biomol. Chem.* **2017**, *15*, 301-305.

[6] T. Konno, T. Takehana, M. Mishima, T. Ishihara, *J. Org. Chem.* **2006**, *71*, 3545-3550.

[7] P. Poutrel, M. V. Ivanova, X. Pannecoucke, P. Jubault, T. Poisson, *Chem. Eur. J.* **2019**, *25*, 15262-15266.

[8] J. Wu, H. Wu, X. Li, X. Liu, Q. Zhao, G. Huang, C. Zhang, *Angew. Chem. Int. Ed.* **2021**, *60*, 20376-20382.

[9] C. B. Kelly, M. A. Mercadante, E. R. Carnaghan, M. J. Doherty, D. C. Fager, J. J. Hauck, A. E. MacInnis, L. J. Tilley, N. E. Leadbeater, *Eur. J. Org. Chem.* **2015**, *2015*, 4071-4076.

[10] Z. Xie, G. Li, G. Zhao, J. Wang, *Chin. J. Chem* . **2010**, *28*, 1212-1216.

[11] X. Wang, Y. Xu, Y. Deng, Y. Zhou, J. Feng, G. Ji, Y. Zhang, J. Wang, *Chem. Eur. J.* **2014**, *20*, 961-965.

[12] I. Protasova, B. Bulat, N. Jung, S. Bräse, *Org. Lett.* **2017**, *19*, 34-37.

[13] K. Futatsugi, A. C. Smith, M. Tu, B. Raymer, K. Ahn, S. B. Coffey, M. S. Dowling, D. P. Fernando, J. A. Gutierrez, K. Huard, J. Jasti, A. S. Kalgutkar, J. D. Knafels, J. Pandit, K. D. Parris, S. Perez, J. A. Pfefferkorn, D. A. Price, T. Ryder, A. Shavnya, I. A. Stock, A. S. Tsai, G. J. Tesz, B. A. Thuma, Y. Weng, H. M. Wisniewska, G. Xing, J. Zhou, T. V. Magee, *J. Med. Chem.* **2020**, *63*, 13546-13560.

[14] B. Potter, E. K. Edelstein, J. P. Morken, *Org. Lett.* **2016**, *18*, 3286-3289.

[15] J. Filmon, D. Grée, R. Grée, *J. Fluorine Chem.* **2001**, *107*, 271-273.

[16] P. Bravo, E. Piovosi, G. Resnati, *J. Chem. Soc., Perkin Trans. 1* **1989**, 1201-1208.

[17] P. J. Kocienski, G. Cernigliaro, G. Feldstein, *J. Org. Chem.* **1977**, *42*, 353-355.

[18] T. R. Hoye, M. Hu, *J. Am. Chem. Soc.* **2003**, *125*, 9576-9577.

[19] Bruker AXS (**2021**) *APEX4 Version 2021.4-0, SAINT Version 8.40B* and *SADABS Bruker AXS area detector scaling and absorption correction Version 2016/2*, Bruker AXS Inc., Madison, Wisconsin, USA.

[20] G. M. Sheldrick, *Acta Cryst.,* **2015**, *A71,* 3-8.

[21] G. M. Sheldrick, *Acta Cryst.,* **2015**, *C71,* 3-8.

[22] Bruker AXS (**1998**) *XP* – Interactive molecular graphics, Version 5.1, Bruker AXS Inc., Madison, Wisconsin, USA.
